# Study on the chirality of gyroid photonic crystals in butterfly wing scales

**DOI:** 10.1038/s41598-025-05750-2

**Published:** 2025-07-01

**Authors:** Masayuki Inoue, Kai Saito, Hinao Aoyama, Haruya Inoue, Ryosuke Ohnuki, Shinya Yoshioka

**Affiliations:** Department of Physics and Astronomy, Faculty of Science and Technology, Tokyo University of Science, Yamazaki, Noda, 278-8510 Japan

**Keywords:** *Teinopalpus imperialis*, *Parides sesostris*, Gyroid photonic crystal, Chirality selection, Crystal orientation, Biophysics, Structural biology, Materials science, Optics and photonics

## Abstract

Some brilliantly colored butterflies are known to possess gyroid photonic crystals in the wing scale. As the gyroid structure is inherently chiral, it is an intriguing question whether the structure has enantiomeric purity or not, considering that biomolecules exhibit homochirality. It has been previously reported for a few lycaenid species that both enantiomeric forms of the gyroid structure are found in multidomain photonic crystals. In this study, we evaluated the chirality of gyroid crystals in the wing scales of the papilionid butterfly *Teinopalpus imperialis* and found only one enantiomeric form (LH-type) in more than 200 gyroid crystals examined. In another papilionid butterfly, *Parides sesostris*, the populations of the two enantiomeric forms were found to be highly unbalanced. Because the gyroid crystals of these two butterflies are known to have a strong orientation preference along the surface normal of the scale, we suggest that crystal orientation-controlled development is related to chirality selection. Our findings provide insights into chirality-selected gyroid synthesis in self-organization processes.

## Introduction

The origin of the homochirality of biological molecules is one of the mysteries in science^1–3^. Only the L-type enantiomeric form is predominantly observed in natural amino acids, whereas the D-type form is observed in sugar molecules. On a macroscopic scale, some creatures have chiral shapes, such as the shell of a snail and the vines of morning glory^4,5^. How is one of the two possible enantiomeric forms created? Such chiral morphogenesis poses interdisciplinary questions that span the fields of biology, chemistry, and physics. Between the microscopic biomolecules and the macroscopic shapes, a subwavelength-sized chiral structure exists in the wing scale of some butterflies. The wing scale contains a three-dimensional (3D) periodic network of cuticle, which serves as a natural example of a photonic crystal. This periodic structure generates brilliant structural colors through optical interference, which are usually enhanced by a dark pigmented background^6–8^. The exact structure of the cuticle network is identified as a gyroid photonic crystal^9–11^. As the gyroid structure is chiral, its mirror image cannot be superimposed on the original image by translation and rotation processes. Therefore, the wing scale of a butterfly has inspired researchers to develop optical materials that are sensitive to circular polarization^12–14^. Turner et al.^15,16^ fabricated a gyroid structure using the direct laser writing method and demonstrated that it functions as a beam splitter that separates different circular polarizations.

The gyroid surface is a type of triply periodic minimal surface that is defined as a surface having zero mean curvature at any point on the surface. The minimal surface of the gyroid separates the 3D space into two bicontinuous regions, and, when one region is filled with the cuticle, the obtained cuticle network becomes an approximation for the photonic crystal in the butterfly wing scale. When the other region is filled, the network corresponds to the mirror image of the first image. These two forms are distinguishable and correspond to the two enantiomeric forms of the gyroid structure. Consequently, a question naturally arises: whether the gyroid crystals in the butterfly wing scale are left-handed (LH) or right-handed (RH). Previous studies have reported that, for some lycaenid butterflies, both chiralities exist in an individual scale. The wing scale does not comprise a single crystal spreading over the entire scale but consists of many small crystals with different crystal orientations, and some of them are LH-gyroid while the others are RH-gyroid^17–19^. However, the LH-gyroid is found more frequently than the RH-gyroid, indicating that some factors break the symmetry between the two enantiomeric forms during the development of the gyroid crystal. The homochirality of biological molecules has been suggested to be a factor, although detailed mechanisms are still unknown. Understanding the chiral development process is a challenging issue because there are no effective methods that directly probe the in vivo development with the spatial resolution of a sub-wavelength scale.

In this study, we evaluated the chirality of the gyroid crystals present in the wings of the papilionid butterfly *Teinopalpus imperialis* and report that only LH-gyroids were observed in all the 218 crystals examined. This is in contrast to the results of previous studies on lycaenid butterflies, in which both chiralities of the gyroid were observed^17–19^. The butterfly *T. imperialis* has a strong preference for crystal orientation^20^, wherein $\langle 111\rangle$ crystal orientations are preferred along the surface normal of the scale. Therefore, we investigated another papilionid butterfly, *Parides sesostris*, which has a similar orientation preference in the $\langle 110\rangle$ direction, and found that the populations of the two enantiomeric forms are highly unbalanced. In this paper, we present the experimental results of these two papilionid butterflies as well as the results of a previously studied lycaenid butterfly, *Callophrys rubi*, for comparison and discuss the possible development process that correlates the chirality selection with crystal orientation.

## Determination of chirality

**Fig. 1:**
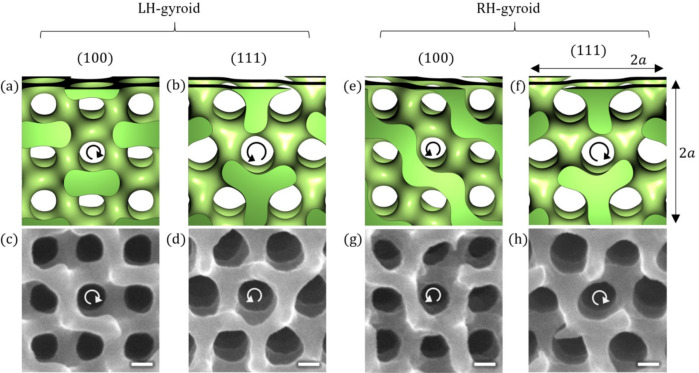
Gyroid structure. (**a**) and (**b**) show the LH gyroid corresponding to (100) and (111) planes, respectively. The arrangement of through holes appears square-like in (**a**) and hexagonal in (**b**), corresponding to the symmetry of the crystal direction. Notably, differently handed screws are observed depending on the directions. (**c**) and (**d**) show the corresponding scanning electron microscopy (SEM) images obtained for the scale of *C. rubi*. (**e**) and (**f**) show the RH gyroid corresponding to (100) and (111) planes, respectively. (**g**) and (**h**) show the SEM images obtained for the scale of *C. rubi*. The structural model was created using Mathematica (version 14.0; Wolfram Research, https://www.wolfram.com/mathematica/). Scale bar: 100 nm for (**c**), (**d**), (**g**), and (**h**).

The gyroid surface can be approximated using the function *f *(*x*, *y*, *z*) that consists of several sinusoidal functions as follows^21^:

1 $$\begin{aligned} f(x,y,z)= \sin \frac{2\pi }{a}x \cos \frac{2\pi }{a}y+\sin \frac{2\pi }{a}y \cos \frac{2\pi }{a}z+\sin \frac{2\pi }{a}z\cos \frac{2\pi }{a}x, \end{aligned}$$

where *x*, *y*, *z* are Cartesian coordinates, and the parameter *a* is the lattice constant of the cubic unit cell. The surface defined by equation $f(x,y,z)=0$ is a good approximation of the minimal surface of the gyroid, which separates the 3D space into two regions with the same volume. The cuticle network inside the butterfly wing scale can be approximated by $f(x,y,z) <t$  (or $f(x,y,z) >t$ ), where the region in the 3D space that satisfies this inequality is filled with the cuticle; therefore, the parameter *t* is related to the volume fraction of the cuticle. For $t=0$, both regions have a volume fraction of 0.5. When one side is filled by a material, the symmetry of the structure is different from that of the gyroid minimal surface. This structure is called a single gyroid, and in this paper we call it the gyroid structure. In the butterfly wing scale, the volume fraction of the cuticle has been reported to be less than 0.5, indicating $t \ne 0$^9^. Thus, to be more precise, the cuticle surface is better approximated by a constant-mean-curvature surface, which separates the space into two regions of unequal volumes, rather than a minimal surface with zero mean curvature. In the case of butterfly wing scales, the lattice constant *a* has been reported to be approximately 300 nm^22,23^. Following previous studies^24^, we defined the LH-gyroid when the structure can be modeled by $f(x,y,z) <t$. In the LH-gyroid, the LH screw of the cuticle network is oriented along the $\langle 111\rangle$ direction of the cubic crystal (Fig. 1b,d). When the space is filled with the opposite inequality $f(x,y,z) >t$, the RH screw of the cuticle is oriented along the $\langle 111\rangle$ direction (Fig. 1f,h); therefore, this network is defined as the RH-gyroid. However, the opposite-handed cuticle screw is observed for different crystal orientations: along the $\langle 100\rangle$ direction, the RH screw of the cuticle is found in the LH gyroid (Fig. 1a,c), whereas the LH screw is found in the RH-gyroid (Fig. 1e,g). These intricate structural features are well summarized in a previous study^24^.

**Fig. 2:**
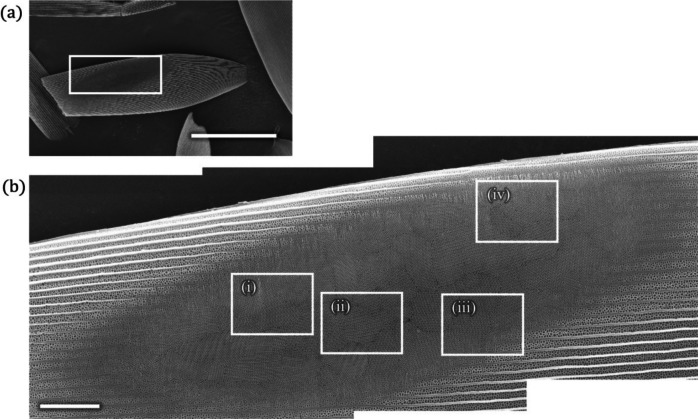
Green scale on the ventral side of *T. imperialis*. An Ar-ion beam is used to mill off the upper surface of a scale. A region approximately indicated by the white rectangle in (**a**) is observed under higher magnification in (**b**). Longitudinally running ridges and honeycombs are removed in the oval area, and the gyroid crystals are exposed. It is noted that three SEM images are composed to obtain a large image with better spatial resolution. Four rectangular regions in (**b**) are observed in detail, as shown in Fig. 3. Scale bar: (**a**) $100\,\upmu \hbox{m}$ and (**b**) $10\,\upmu \hbox{m}$.

Previous studies used 3D methods such as electron tomography and X-ray nanotomography to determine the chirality of the cuticle network^11,18,25^. These methods are powerful because they can fully characterize complex 3D networks. However, a 2D observation using scanning electron microscopy (SEM) can also be used to determine the handedness of the cuticle screw when the surface normal is nearly along the $\langle 111\rangle$ or $\langle 100\rangle$ direction. The process of determining the handedness using SEM observations has already been reported^10,17^. The butterfly *T. imperialis* is advantageous from this aspect because the gyroid crystals have a strong orientation preference in which the $\langle 111\rangle$ direction is along the surface normal^20^. Therefore, if the upper scale structures, called ridges and honeycombs, covering the gyroid crystal are removed, the cuticle screw along the $\langle 111\rangle$ direction can be observed directly. In this study, we utilized an apparatus called a cross section polisher for this purpose, in which a broad Ar ion beam mills off the upper scale structures (Fig. 2). This apparatus is highly useful because smooth sections can be prepared, allowing us to accurately observe the structures (see the Experimental section for details). After exposing the gyroid crystals, the surface was observed with the SEM. It is often necessary to tilt the sample during SEM observations because a slight tilt makes it easier to determine the handedness of cuticle screws along the through holes.

The obtained SEM images were carefully examined to determine the handedness of the cuticle screw. This process depended on the image quality. In some cases, it was easy to determine the handedness, as shown in Fig. 1c,d,g,h. In other cases, we carefully checked the variation in the gray level of the image along assumed LH- or RH-spirals around the through holes; if the assumed handedness is correct, the gray level decreases gradually and becomes darker along the screw. When we were unable to determine the handedness confidently, we removed the image from the analysis.

## Results

**Fig. 3:**
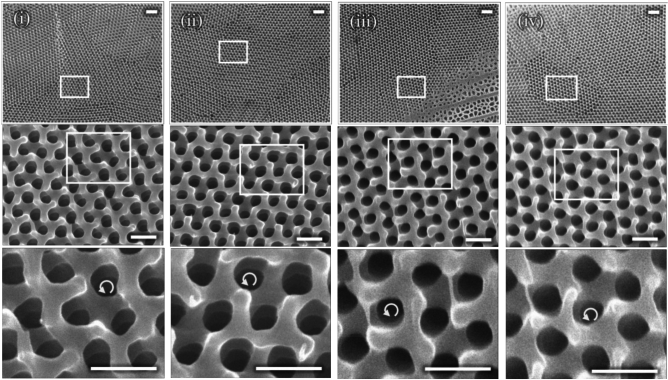
Chirality of gyroid crystals. Four rectangular regions in Fig. 2b are observed, which are denoted as (**i**)–(**iv**). In the top-row images, we can observe that the surface structure of gyroid crystal domains has a different texture. The rectangular region is observed under high magnification, as shown in middle-row images, where the cuticle network with the hexagonal arrangement of through holes is observed. The cuticle screw is examined for chirality determination, and the images are shown in the bottom row. Curled arrows indicate the LH screw of the cuticle network. Scale bars from top to bottom: $1\,\upmu \hbox{m}$, 400 nm, and 400 nm.

We first investigated the green scale of *T. imperialis* on the ventral side. The upper scale structures were removed via Ar-ion beam milling from the oval region shown in Fig. 2, and the gyroid crystals were exposed. Four rectangular regions were closely inspected, as shown in Fig. 3. The surface structure consisted of the hexagonal arrangement of through holes corresponding to the (111) plane, consistent with that reported in a previous study^20^. The LH cuticle screw could be clearly observed under high magnification, as shown in Fig. 3, which allowed us to determine that the chirality of these gyroids is LH based on a comparison with the modeling (Fig. 1b). We determined the chirality of the gyroid from one domain to another and unexpectedly found that all 29 gyroid crystals in the scale shown in Fig. 2 were LH. The identified crystal domains are shown in Fig. S1, and Fig. S2 shows the SEM images of other crystals used for chirality determination. Our observations were expanded to different green scales, and, consequently, we found all the examined 84 gyroid crystals from the other four ventral scales were LH (Fig. S2 and Table S1). Furthermore, we examined another individual of *T. imperialis* and found that 14 gyroid crystals from two ventral scales were LH. Therefore, 127 (=29+84+14) gyroid crystals were found to be LH. In addition, we observed structurally colored green scales on the dorsal side. They were smaller than those observed on the ventral side (Fig. S3). However, the color-causing microstructure is commonly the multidomain gyroid crystals, and the chirality was found to be LH for all 91 domains from 10 scales (see the SEM images in Fig. S4). In summary, we investigated 218 crystal domains from 17 scales (7 and 10 scales on the ventral and dorsal sides, respectively) from two individuals and found that the chirality of all gyroid crystals was LH; the results are summarized in Table S1.

We note that the number of gyroid crystals observed per scale depends largely on the orientation of the sectioning plane. When the plane was nearly parallel to the surface of the scale, many gyroid crystals were exposed and could be examined. In contrast, when the plane was significantly tilted, fewer crystals were revealed, and the chirality could only be determined for a limited number of them.

**Fig. 4:**
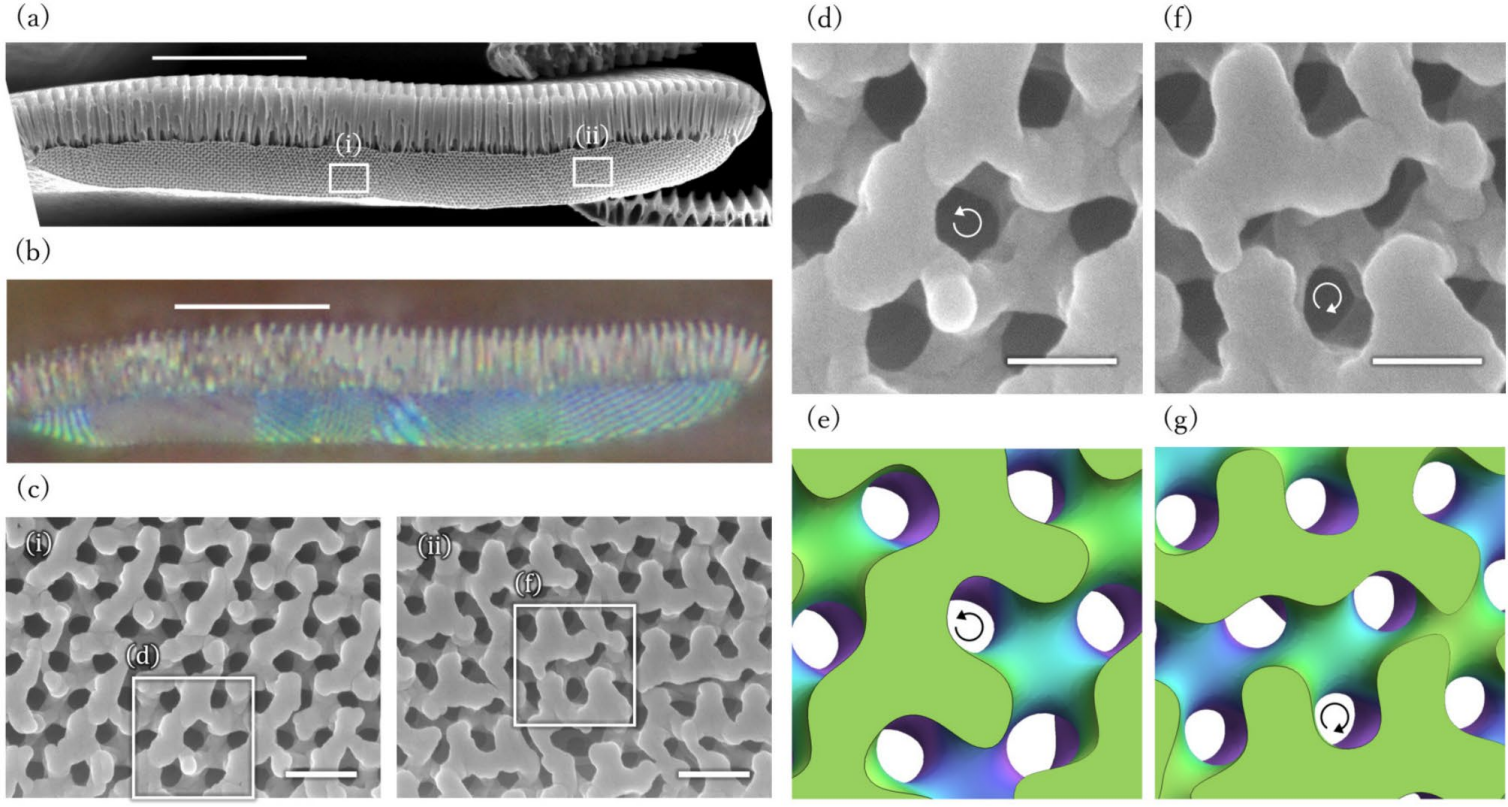
Gyroid chirality of *Parides sesostris*. (**a**) SEM image showing the cross section of the scale. (**b**) Optical image of the scale corresponding to (**a**). (**c**) SEM images of rectangular regions (**i**) and (**ii**) shown in (**a**). (**d**) Close-up SEM image of the LH-gyroid domain in (**c**), and (**e**) its corresponding model. (**f**) Close-up SEM image of the RH-gyroid domain in (**c**), and (**g**) its corresponding model. Curled arrows indicate the LH and RH screws of the cuticle network along the $\langle 111\rangle$ direction. In panels (**e**) and (**g**), the surface color varies from green to purple as a function of surface height. Scale bars: (**a**, **b**) $10\,\upmu \hbox{m}$, (**c**) 400 nm and (**d**, **f**) 200 nm.

The above findings of *T. imperialis* motivated us to examine another butterfly, *P. sesostris*, which has also been reported to have a strong preference for crystal orientation. In the scales of this butterfly, the gyroid crystals were arranged such that the $\langle 110\rangle$ direction was aligned with the surface normal^26^. As this direction is not one along which the cuticle screw can be seen, the frontal section is not effective in this butterfly. Thus, we prepared the transverse cross section of the scales via ion beam milling (Fig. 4). A smooth cross section was obtained, as shown in Fig. 4a, where the crystal domains clearly correspond to optical micrographs with differently oriented stripes (Fig. 4b). Judging from the surface texture, seven crystal domains are observed in this cross section. When the orientation of the exposed crystal is along the $\langle 111\rangle$ or $\langle 100\rangle$ direction, we can determine the chirality as shown in Fig. 4c–g. The crystal domains (i) and (ii), shown in Fig. 4a, oriented along the $\langle 111\rangle$ direction, were determined to be LH- and RH-gyroid, respectively, based on comparisons with the gyroid models shown in Fig. 4. e,g. Fig. S5 presents comparisons between other regions of the SEM images and structural models, further supporting the above determination of chirality. We performed similar observations on other scales and found 81 LH-gyroids and 10 RH-gyroids among 91 crystals from 40 scales from two individuals. The LH:RH ratio was calculated to be 0.89:0.11 (see Fig. S6 for the SEM images of these observations and Table S2 for the summary of scales, domains, and the determined gyroid chiralities).

A lycaenid butterfly, the green hairstreak butterfly *Callophrys rubi*, was also examined for comparison. We milled off the upper scale structure using ion beam milling, as shown in Fig. S7. Some ridges remained due to the slightly shallow ion beam milling process. However, many gyroid crystals were observed with different crystal orientations. In agreement with the findings reported in a previous study^27^, crystals with a $\langle 100\rangle$ orientation were found more frequently than other orientations. Detailed SEM observations identified 21 LH-gyroids and 10 RH-gyroids among 31 crystals from one scale (see Fig. S8 for the SEM images). Therefore, the ratio of the two enantiomers was calculated to be LH:RH = 0.68:0.32.

## Discussion

Previous studies reported that LH-gyroids were found with a higher probability than RH-gyroids for two lycaenid butterflies^17–19^. For the butterfly *C. rubi*, Mille et al. ^17^ reported that LH- and RH-gyroids were found in a ratio 21:3 (= 0.88:0.12) for 24 crystals using SEM, whereas Winter et al.^18^ reported an LH:RH ratio of 10:2 (= 0.83:0.17) for 12 crystals using electron tomography. In the present study, we obtained an LH:RH ratio of 0.68:0.32 for 31 gyroid crystals from *C. rubi*. The percentage of the LH-gyroid was slightly lower than that reported in the above two studies. For the lycaenid butterfly *Thecla opisena*, Wilts et al.^19^ reported an LH:RH ratio of 35:12 (= 0.74:0.26) using high-resolution X-ray tomography.

The present study reported strong chirality selection in the butterfly *T. imperialis*: only LH-gyroid crystals were observed among 218 gyroid crystals. One may immediately expect a circular polarization effect based on this finding. However, a previous study has already rejected this expectation by measuring the circularly polarized reflectance^24^. Several possible reasons have been discussed in detail, and one major reason is the crystal orientation. Although the $\langle 100\rangle$ is expected to have a different circular polarization reflectance, the gyroid crystals of *T. imperialis* have a preference for the $\langle 111\rangle$ direction along the surface normal of the scale. Therefore, enantiomeric purity is not aimed at least for optical purposes, but may be a byproduct of other biological processes. However, the findings of this study provide insights into the chirality-selected morphogenesis of the photonic structure.

Here, we review the development of the wing scale following the pioneering works of Ghiradella^28,29^. The scale-forming cell first produces a membrane sac, in which the scale structure is formed by the extracellular cuticle deposition. The formation of scale structures consists of two stages. First, extracellular ornaments such as ridges, cross-ribs, and microribs are produced on the top surface of the scale. The second stage begins with the invagination of the plasma membrane, which later becomes the pillar (also called trabecula) after cuticle secretion. This structural component connects the upper scale structures and the bottom layer of the scale. The second stage is considered to be the stage at which the gyroid structure is formed. Saranathan et al.^10^ considered that the plasma and smooth endoplasmic membranes are folded to form the precursor of the gyroid. One channel is connected to the extracellular space where the nascent cuticle is secreted. Winter et al.^18^ suggested the co-folding model, in which cuticle secretion occurs simultaneously with the folding process. This model is supported by the chiral imbalance of the gyroid found in a lycaenid butterfly^19^, as the molecular chirality of the material can affect the folding process and result in the break in symmetry between the two enantiomeric forms. That is, we speculate that the molecular chirality causes a slight energy difference between the two types of the gyroid, and owing to this difference, the LH-type is more favorably produced possibly according to the Boltzmann factor in statistical physics, although the question of how far this process is from equilibrium remains.

Considering the enantiomeric purity of the gyroid in *T. imperialis* and the large imbalance in *P. sesostris*, we speculate that the crystal orientation is another factor that affects chirality. Singer et al. suggested a layer-by-layer crystal growth process as a possible origin for the orientation preference along the $\langle 111\rangle$ direction in *T. imperialis*^20^. In fact, some SEM images obtained in this study indicate that the gyroid crystal develops from the upper scale structures rather than the bottom cuticle layer, as shown in Fig. S9a and Movie S1. In the longitudinal section, many air voids can be observed between the gyroid crystal and the bottom layer, whereas the gyroid crystal appears to be continuously connected to the upper scale structures (panel (iii) in Fig. 3). In addition, Fig. S9b shows that, near the basal part of the scale, the cuticle network exists only in the upper scale part. Based on the detailed structural observations of *T. opisena*, Wilts et al. also reported that the gyroid crystals develop from the upper laminae of the scale^19^.

If the layer-by-layer crystal growth from the upper lamina is the case, the two-dimensional arrangement of the invaginating points should have a threefold symmetry such that the upper surface of the gyroid becomes the (111) plane in the wing scale of *T. imperialis*. We speculate that, owing to such regularly arranged invagination points, the membrane folding is more significantly affected by the molecular chirality than the crystal growth from point nucleation; when a crystal grows from a point nucleus, it is thought that the way of membrane folding at the very first stage determines the chirality of the crystal. However, if the crystal grows from a plane, the chirality selection occurs simultaneously for many invagination points, in which the probability of the LH-type gyroid is enhanced. The different orientation preferences of *P. sesostris* may be related to the slight increase in the RH-gyroid population.

A recent study reported that a species of longhorn beetle has I-WP minimal surface-based photonic crystals in the scale^30–32^. Amorphous I-WP-like networks have also been found in different longhorn beetles^33^. The I-WP minimal surface is known as an unbalanced minimal surface in which neither the two interconnected spaces divided by the surface have the same volume nor are related by the symmetry operation. Interestingly, one specific channel is always occupied by the cuticle to form the network in multidomain photonic crystals. This fact seems to indicate that the membrane is folded such that the channel specifically becomes the extracellular space where the cuticle is secreted. This is similar to the case of *T. imperialis*, where only the LH-gyroid space becomes extracellular. The longhorn beetle has a preference for the (110) plane orientation^30^. Therefore, the selection of the specific channel may be related to the control of the two-dimensional pattern of the invaginating points, which results in the selection of crystal orientation. Further studies are strongly encouraged to examine whether the above speculations are true or not.

Recently, a novel imaging method called speckle-correlation reflection phase microscopy has been applied to the in vivo development of the wing scale of a butterfly with a lateral resolution of approximately 500 nm^34^. The time-lapse data for the ridge formation has been well analyzed using an elastic buckling model^35^. Furthermore, another study applied super-resolution structured illumination to observe the development of the honeycomb lattice in the scales of Papilio butterflies^36^. The development and application of such state-of-the-art microscopy techniques are crucial for better elucidation of the in vivo development of photonic structures.

In conclusion, we reported that only one enantiomeric form of gyroid crystal was found in the wing scale of *T. imperialis*. We also found that the chirality ratio of *P. sesostris* is highly unbalanced. As the gyroid crystals of these butterflies are known to have a strong orientation preference, the crystal orientation is suggested as another factor that affects the chirality of the gyroid during the development of the scale. These findings provide valuable insights into the chirality selection during the gyroid synthesis in self-organizing processes, which is important for the application of optical materials sensitive to circular polarizations.

**Fig. 5:**
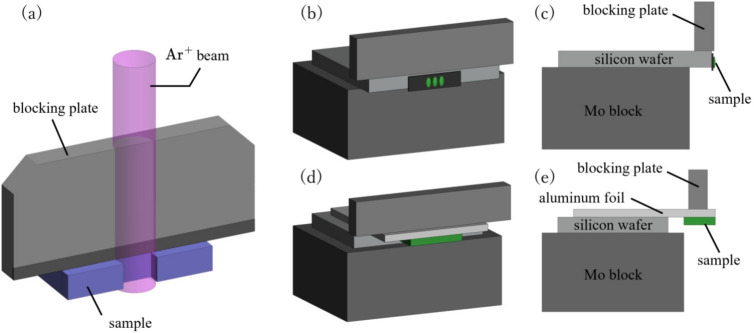
Experimental setup for ion beam milling. (**a**) Illustration of the general experimental setup. (**b**) and (**c**) show the setup for the case of *T. imperialis* to obtain the frontal section of the scale. (**d**) and (**e**) show the setup for the case of *P. sesostris* to obtain the cross section of the scale. The structural model was created using Mathematica (version 14.0; Wolfram Research, https://www.wolfram.com/mathematica/).

## Methods

The butterfly specimens used in this study were purchased from an online supplier. The gyroid crystals are located in the lower part of the scale. Therefore, the upper scale structures such as ridges and honeycombs must be removed for direct observation. We employed an apparatus called a cross section polisher (JEOL, IB-19530CP) for this purpose. This apparatus uses a broad $\text {Ar}^{+}$ beam to mill the surface (Fig. 5a). For the frontal section of *T. imperialis* scales, several wing scales were first placed on a piece of carbon tape, the surface of which was set nearly parallel to the $\text {Ar}^{+}$ beam (Fig. 5b,c). It was important to place the sample slightly outside the blocking plate made of Mo so that only a-few-$\mu$m-thick surface was exposed to the beam to shave off the upper scale structure. This process was performed using a camera equipped with the apparatus. However, it was difficult to perfectly adjust the sample position. Therefore, we placed several scales on a piece of carbon tape for a better chance of obtaining a good section. An example of the section obtained in this manner is shown in Fig. 2. For the cross section of *P. sesostris*, a small piece of the wing of approximately $1.0\times 5.0\,{\text {mm}}^2$ with several scales was glued to an aluminum foil, and the edge of the sample was exposed to the ion beam (Fig. 5d,e) for preparing the cross section (Fig. 4). The accelerating voltage was 3.5 kV, and the milling time was 8 h. A simple tape-peeling method similar to that used in a previous study^19^ was also used to expose the gyroid crystal for some scales: the bottom cuticle layer of the scale was removed using a piece of adhesive tape. After the gyroid structure was exposed, the surface structure was observed with an SEM (JEOL JCM-6000) under low magnification and another SEM (JEOL JSM-6500F) for chirality determination. The gonio and rotation stages in the sample chamber were often used to tilt the sample by several degrees and observe the screw axis of the cuticle network.

## Supplementary Information

Supplementary Information 1.

Supplementary Information 2.

Supplementary Information 3.

Supplementary Information 4.

Supplementary Information 5.

Supplementary Information 6.

## Data Availability

All data needed to draw the conclusions in the paper are present in the paper and the supporting information.

## References

[1] Wagnière, G. H. *On chirality and the universal asymmetry: Reflections on image and mirror image* (Wiley, New York, 2007).

[2] Kitagawa, Y., Segawa, H. & Ishii, K. Magneto-chiral dichroism of organic compounds. *Angew. Chem. Int. Ed.* **50**, 8993–8993 (2011). 10.1002/anie.201101809 21796747

[3] Cowan, J. A. & Furnstahl, R. J. Origin of chirality in the molecules of life. *ACS Earth Space Chem.* **6**, 2575–2581 (2022).

[4] Maderspacher, F. Snail chirality: The unwinding. *Curr. Biol.* **26**, R215–R217 (2016). 26954445 10.1016/j.cub.2016.02.008

[5] Nakamura, M. & Hashimoto, T. Mechanistic insights into plant chiral growth. *Symmetry* **12**, 2056 (2020).

[6] Hyde, S. T. & Schröder-Turk, G. E. Geometry of interfaces: Topological complexity in biology and materials. *Interface Focus* **2**, 529–538 (2012).

[7] Poladian, L., Wickham, S., Lee, K. & Large, Maryanne CJ. Iridescence from photonic crystals and its suppression in butterfly scales. *J. R. Soc. Interface* **6**, S233–S242 (2009). 18980932 10.1098/rsif.2008.0353.focus PMC2706480

[8] Kertész, K. et al. Gleaming and dull surface textures from photonic-crystal-type nanostructures in the butterfly *Cyanophrys remus*. *Phys. Rev. E* **74**, 021922 (2006). 10.1103/PhysRevE.74.021922 17025487

[9] Michielsen, K. & Stavenga, D. Gyroid cuticular structures in butterfly wing scales: Biological photonic crystals. *J. R. Soc. Interface* **5**, 85–94 (2008). 17567555 10.1098/rsif.2007.1065 PMC2709202

[10] Saranathan, V. et al. Structure, function, and self-assembly of single network gyroid (I4132) photonic crystals in butterfly wing scales. *Proc. Natl. Acad. Sci. U.S.A.* **107**, 11676–11681 (2010). 20547870 10.1073/pnas.0909616107 PMC2900708

[11] Schröder-Turk, G. E. et al. The chiral structure of porous chitin within the wing-scales of *Callophrys rubi*. *J. Struct. Biol.* **174**, 290–295 (2011). 21272646 10.1016/j.jsb.2011.01.004

[12] Saba, M. et al. Circular dichroism in biological photonic crystals and cubic chiral nets. *Phys. Rev. Lett.* **106**, 103902 (2011). 21469792 10.1103/PhysRevLett.106.103902

[13] Goi, E., Cumming, B. P. & Gu, M. Gyroid “srs’’ networks: Photonic materials beyond nature. *Adv. Opt. Mater.* **6**, 1800485 (2018).

[14] Kilchoer, C. et al. Strong circular dichroism in single gyroid optical metamaterials. *Adv. Opt. Mater.* **8**, 1902131 (2020).

[15] Turner, M. D. et al. Miniature chiral beamsplitter based on gyroid photonic crystals. *Nat. Photon.* **7**, 801–805 (2013).

[16] Turner, M. D., Schröder-Turk, G. E. & Gu, M. Fabrication and characterization of three-dimensional biomimetic chiral composites. *Opt. Express* **19**, 10001–10008 (2011). 21643258 10.1364/OE.19.010001

[17] Mille, C., Tyrode, E. C. & Corkery, R. W. 3D Titania photonic crystals replicated from gyroid structures in butterfly wing scales: Approaching full band gaps at visible wavelengths. *RSC Adv.* **3**, 3109–3117 (2013).

[18] Winter, B., Dieker, C., Schröder-Turk, G. E., Mecke, K. & Spiecker, E. Coexistence of both gyroid chiralities in individual butterfly wing scales of *Callophrys rubi*. *Proc. Natl. Acad. Sci. U.S.A.* **112**, 12911–12916 (2015). 26438839 10.1073/pnas.1511354112 PMC4620911

[19] Wilts, B. D. et al. Butterfly gyroid nanostructures as a time-frozen glimpse of intracellular membrane development. *Sci. Adv.* **3**, e1603119 (2017). 28508050 10.1126/sciadv.1603119 PMC5406134

[20] Singer, A. et al. Domain morphology, boundaries, and topological defects in biophotonic gyroid nanostructures of butterfly wing scales. *Sci. Adv.* **2**, e1600149 (2016). 27386575 10.1126/sciadv.1600149 PMC4928966

[21] Wohlgemuth, M., Yufa, N., Hoffman, J. & Thomas, E. L. Triply periodic bicontinuous cubic microdomain morphologies by symmetries. *Macromolecules* **34**, 6083–6089 (2001).

[22] Wilts, B. D., Michielsen, K., De Raedt, H. & Stavenga, D. G. Iridescence and spectral filtering of the gyroid-type photonic crystals in *Parides sesostris* wing scales. *Interface Focus* **2**, 681–687 (2012). 24098853 10.1098/rsfs.2011.0082 PMC3438581

[23] Michielsen, K., De Raedt, H. & Stavenga, D. G. Reflectivity of the gyroid biophotonic crystals in the ventral wing scales of the Green Hairstreak butterfly, *Callophrys rubi*. *J. R. Soc. Interface* **7**, 765–771 (2010). 19828506 10.1098/rsif.2009.0352 PMC2874226

[24] Saba, M., Wilts, B. D., Hielscher, J. & Schröder-Turk, G. E. Absence of circular polarisation in reflections of butterfly wing scales with chiral gyroid structure. *Mater. Today Proc.* **1**, 193–208 (2014).

[25] Wilts, B. D., Giraldo, M. A. & Stavenga, D. G. Unique wing scale photonics of male Rajah Brooke’s birdwing butterflies. *Front. Zool.* **13**, 36 (2016). 27525030 10.1186/s12983-016-0168-7 PMC4983073

[26] Yoshioka, S., Fujita, H., Kinoshita, S. & Matsuhana, B. Alignment of crystal orientations of the multi-domain photonic crystals in *Parides sesostris* wing scales. *J. R. Soc. Interface* **11**, 20131029 (2014). 24352678 10.1098/rsif.2013.1029 PMC3899871

[27] Corkery, R. W. & Tyrode, E. C. On the colour of wing scales in butterflies: Iridescence and preferred orientation of single gyroid photonic crystals. *Interface Focus* **7**, 20160154 (2017). 28630678 10.1098/rsfs.2016.0154 PMC5474040

[28] Ghiradella, H. Structure and development of iridescent butterfly scales: Lattices and laminae. *J. Morphol.* **202**, 69–88 (1989). 29865680 10.1002/jmor.1052020106

[29] Ghiradella, H. Hairs, bristles, and scales. In *Microscopic anatomy of invertebrates* (eds F. N. Harrison & M. Locke) Vol. 11A, 257–289 (Insecta, Wiley-Liss, 1998).

[30] Kobayashi, Y., Ohnuki, R. & Yoshioka, S. Discovery of I-WP minimal-surface-based photonic crystal in the scale of a longhorn beetle. *J. R. Soc. Interface* **18**, 20210505 (2021). 34753307 10.1098/rsif.2021.0505 PMC8580427

[31] Ohnuki, R., Kobayashi, Y. & Yoshioka, S. Polarization-dependent reflection of I-WP minimal-surface-based photonic crystal. *Phys. Rev. E* **106**, 014123 (2022). 35974583 10.1103/PhysRevE.106.014123

[32] Bauernfeind, V. et al. Not only a matter of disorder in I-WP minimal surface-based photonic networks: Diffusive structural color in *Sternotomis amabilis* longhorn beetles. *Mater. Today Adv.* **23**, 100524 (2024).

[33] Bauernfeind, V., Djeghdi, K., Gunkel, I., Steiner, U. & Wilts, B. Photonic amorphous I-WP-like networks create angle-independent colors in *Sternotomis virescens* longhorn beetles. *Adv. Funct. Mater.* **34**, 2302720 (2023).

[34] McDougal, A. D., Kang, S., Yaqoob, Z., So, P. T. C. & Kolle, M. In vivo visualization of butterfly scale cell morphogenesis in *Vanessa cardui*. *Proc. Natl. Acad. Sci. U.S.A.* **118**, e2112009118 (2021). 34845021 10.1073/pnas.2112009118 PMC8670486

[35] Totz, J. F. et al. Cell membrane buckling governs early-stage ridge formation in butterfly wing scales. *Cell Rep. Phys. Sci.* **5**, 102063 (2024). 40084173 10.1016/j.xcrp.2024.102063 PMC11905118

[36] Seah, K. S. & Saranathan, V. Hierarchical morphogenesis of swallowtail butterfly wing scale nanostructures. *Elife* **12**, RP89082 (2023). 37768710 10.7554/eLife.89082 PMC10538957

