# Supplementary material for: Study on the chirality of gyroid photonic crystals in butterfly wing scales
Source: Sci Rep. 2025 Jul 1;15:20968. doi: 10.1038/s41598-025-05750-2 (PMC12215952; doi:10.1038/s41598-025-05750-2)
Supplement: Supplementary file 1 — Supplementary Information 1. [file 41598_2025_5750_MOESM1_ESM.pdf]

Supporting Information

Study on the chirality of gyroid photonic crystals in butterfly wing scales

Masayuki Inoue, Kai Saito, Hinao Aoyama, Haruya Inoue, Ryosuke Ohnuki*, Shinya Yoshioka*

Department of Physics and Astronomy, Faculty of Science and Technology, Tokyo University of Science, Yamazaki, Noda, 278-8510, Japan.

[*] Corresponding Author E-mail:
R.O.: r_ohnuki@rs.tus.ac.jp, S.Y.: syoshi@rs.tus.ac.jp

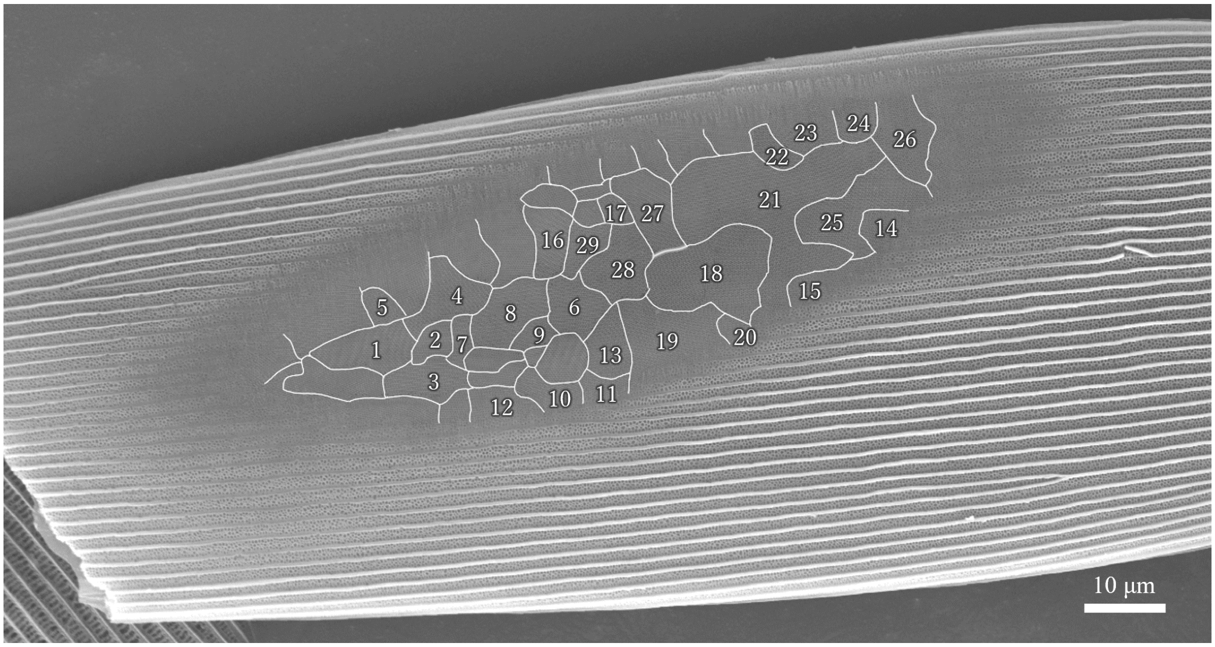

Figure S1 Boundaries between the gyroid crystals of the scale shown in Fig. 2. The domains are indicated by numbers of which the gyroid chirality is determined. The scale shown corresponds to scale No.2 in Fig. S2. Domain boundaries are drawn as boundaries between different textures of the surface structure. As some domains are significantly larger than the others, they may be separated into different crystal domains.

Figure S2 PDF file (FigureS2.pdf) SEM images of the ventral scales of *T. imperialis*.

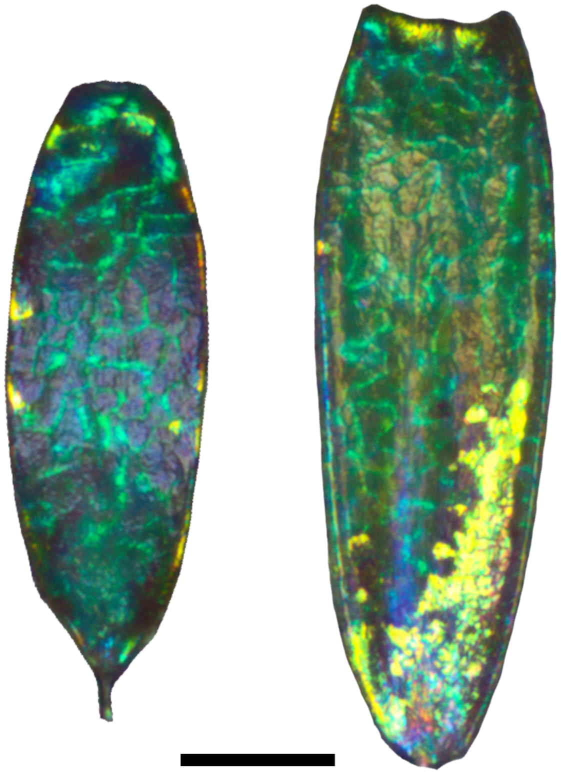

Figure S3 Green scales of *T. imperialis* taken from the dorsal (left) and ventral (scale) sides of the wing. The photographs are taken from the underside of the scale. Scale bar: 50 μm

Figure S4 PDF file(FigureS4.pdf). SEM images of dorsal scales of *T. imperialis*.

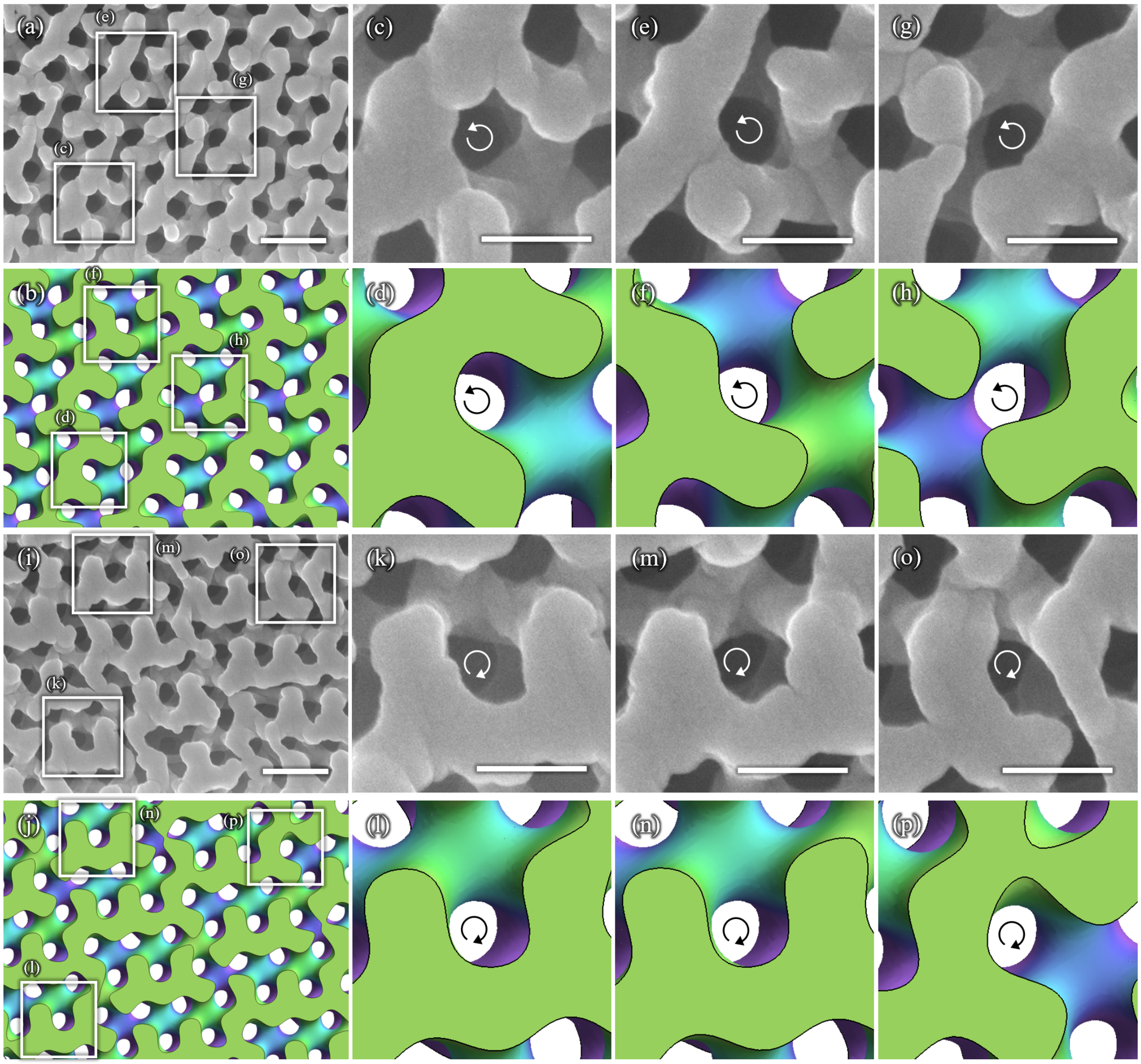

Figure S5 Chirality evaluation of *P. sesostris*. (a) SEM image and (b) model of an LH gyroid domain. Image (a) is the same as Fig. 4(c)(i) in the main text. (c,e,g) Close-up SEM images and (d,f,h) corresponding models. (i) SEM image and (j) model of an RH gyroid domain. Image (i) is the same as Fig. 4(c)(ii) in the main text. (k,m,o) Close-up SEM images and (l,n,p) corresponding models. The surface color of the models varies from green to purple as a function of surface height. Curled arrows indicate the LH and RH helices of the cuticle network along the ⟨111⟩ direction. Scale bars: (a,i) 400 nm; (c,e,g,k,m,o) 200 nm.

Figure S6 PDF file(FigureS6.pdf).
SEM images of *P. sesostris* scales used for chirality determination.

Table S1 Summary of the chirality investigation of *Teinopalpus imperialis*. The method column indicates the method used to expose the gyroid. CP: cross section polisher; TP: tape peeling. All the investigated domains have the [111] crystal orientation with the left-handed (LH) screw of the cuticle (LH-gyroid).

ventral or dorsal	specimen No.	scale No.	Number of domains examined	[111]lh	method
ventral	1	1	45	45	CP
ventral	1	2	29	29	CP
ventral	1	3	21	21	TP
ventral	1	4	10	10	TP
ventral	1	5	8	8	TP
ventral	2	6	13	13	CP
ventral	2	7	1	1	CP
		subtotal	127	127	
dorsal	1	1	21	21	CP
dorsal	1	2	17	17	CP
dorsal	1	3	5	5	CP
dorsal	1	4	5	5	CP
dorsal	1	5	7	7	CP
dorsal	1	6	4	4	CP
dorsal	1	7	4	4	CP
dorsal	1	8	7	7	CP
dorsal	1	9	11	11	CP
dorsal	1	10	10	10	CP
		subtotal	91	91	
total			218	218	

Table S2 Summary of the chirality investigation of *Parides sesostris*. Crystal domains with the [111] orientation with the left-handed screw and the [100] orientation with the right-handed screw are assigned to the LH-gyorid.

specimen No.	scale No.	Number of domains examined	LH gyroid		RH gyroid	
			[111]lh	[100]rh	[111]rh	[100]lh
1	1	5	1	3	1	0
1	2	1	1	0	0	0
1	3	2	2	0	0	0
1	4	2	2	0	0	0
1	5	1	1	0	0	0
1	6	1	1	0	0	0
1	7	4	2	2	0	0
1	8	1	0	1	0	0
1	9	4	1	3	0	0
1	10	1	1	0	0	0
1	11	6	2	3	1	0
1	12	3	3	0	0	0
1	13	3	1	1	1	0
1	14	4	3	1	0	0
2	1	1	0	1	0	0
2	2	2	1	1	0	0
2	3	2	0	2	0	0
2	4	1	1	0	0	0
2	5	1	1	0	0	0
2	6	2	0	2	0	0
2	7	1	0	1	0	0
2	8	1	1	0	0	0
2	9	3	3	0	0	0
2	10	3	1	1	1	0
2	11	3	2	0	1	0
2	12	4	2	1	1	0
2	13	1	0	1	0	0
2	14	2	2	0	0	0
2	15	2	2	0	0	0
2	16	2	1	1	0	0
2	17	2	0	0	2	0
2	18	3	1	1	1	0
2	19	2	1	1	0	0
2	20	2	2	0	0	0
2	21	1	1	0	0	0
2	22	2	2	0	0	0
2	23	1	1	0	0	0
2	24	2	1	1	0	0
2	25	3	2	1	0	0
2	26	4	2	1	1	0
total		91	51	30	10	0

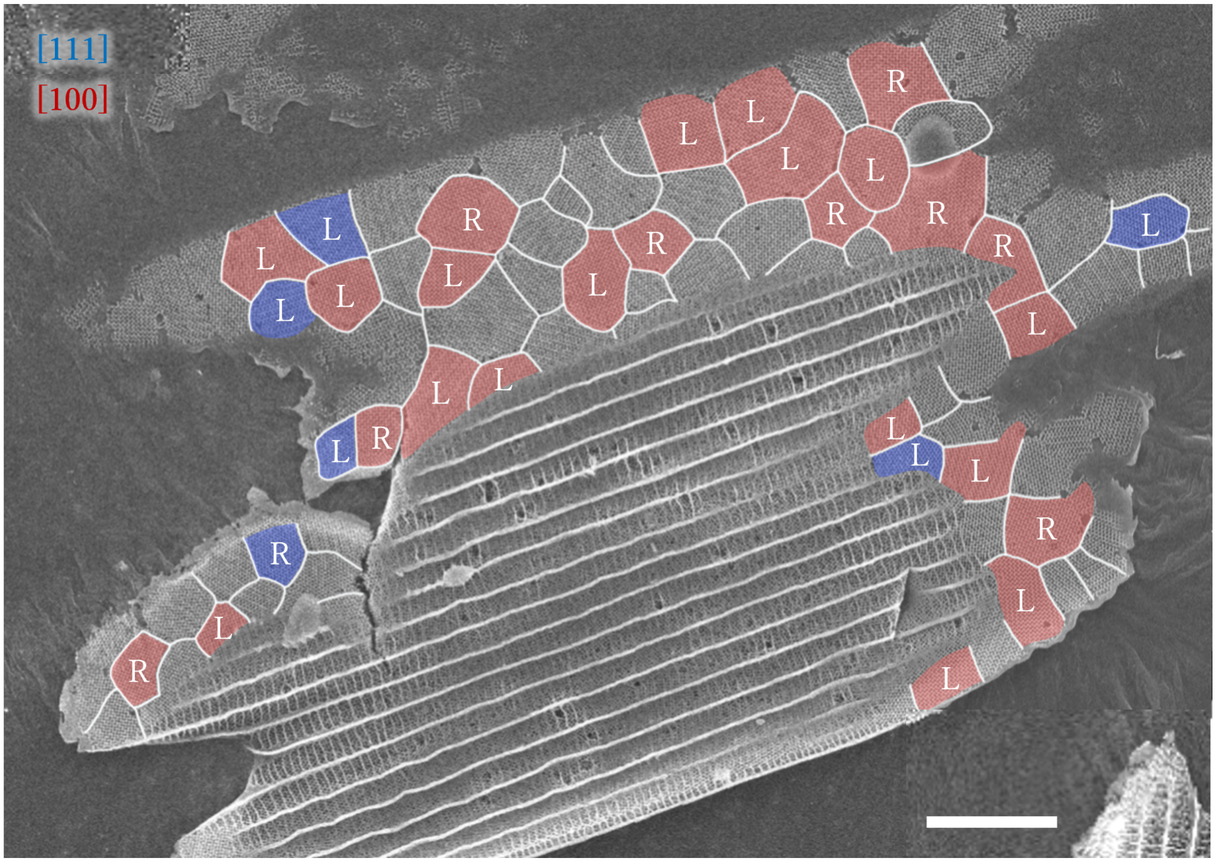

Figure S7 A scale of *Callophrys rubi*. The upper scale structures are partially removed by ion beam milling. Blue and red colors indicate the orientation of the crystal in ⟨111⟩ and ⟨100⟩ directions, respectively. Alphabets L and R indicate chirality. In crystal domains without colors, chirality was not determined because crystal orientations other than ⟨111⟩ or ⟨100⟩ are along the surface normal. Scale bar: 10 μm.

Figure S8 PDF file (FigureS8.pdf)
SEM images of *C. rubi* scales used for chirality determination.

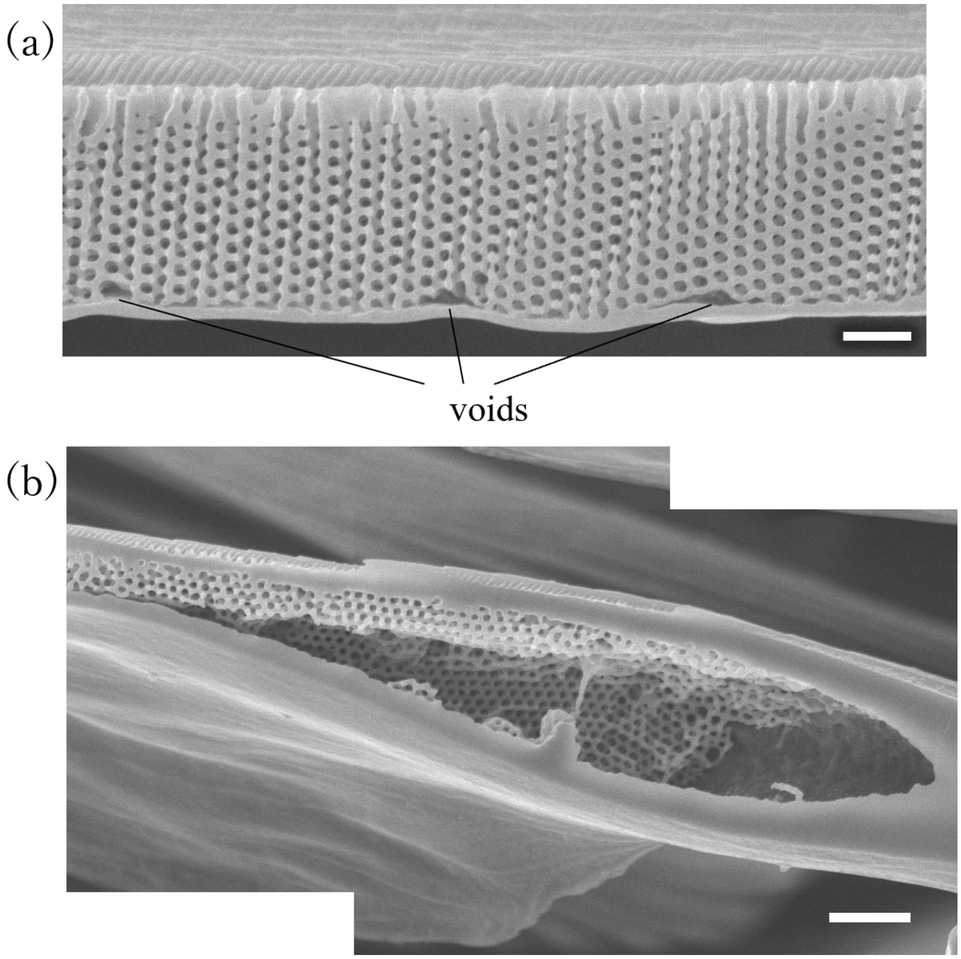

Figure S9 Longitudinal section of a scale of *T. imperialis*. In (a), air regions are observed between the gyroid crystals and the bottom layer. (b) The section near the root of the scale shows that a cuticle network is observed only in the upper cuticle layer. It is noted that two SEM images are composed to obtain a larger image. Scale bar: (a) 1 μm and (b) 2 μm.

Movie S1 (MovieS1.mp4) Entire longitudinal section of a scale of *T. imperialis*.
