# Supplementary material for: Study on the chirality of gyroid photonic crystals in butterfly wing scales
Source: Sci Rep. 2025 Jul 1;15:20968. doi: 10.1038/s41598-025-05750-2 (PMC12215952; doi:10.1038/s41598-025-05750-2)

specimen No. 1
scale No. 1

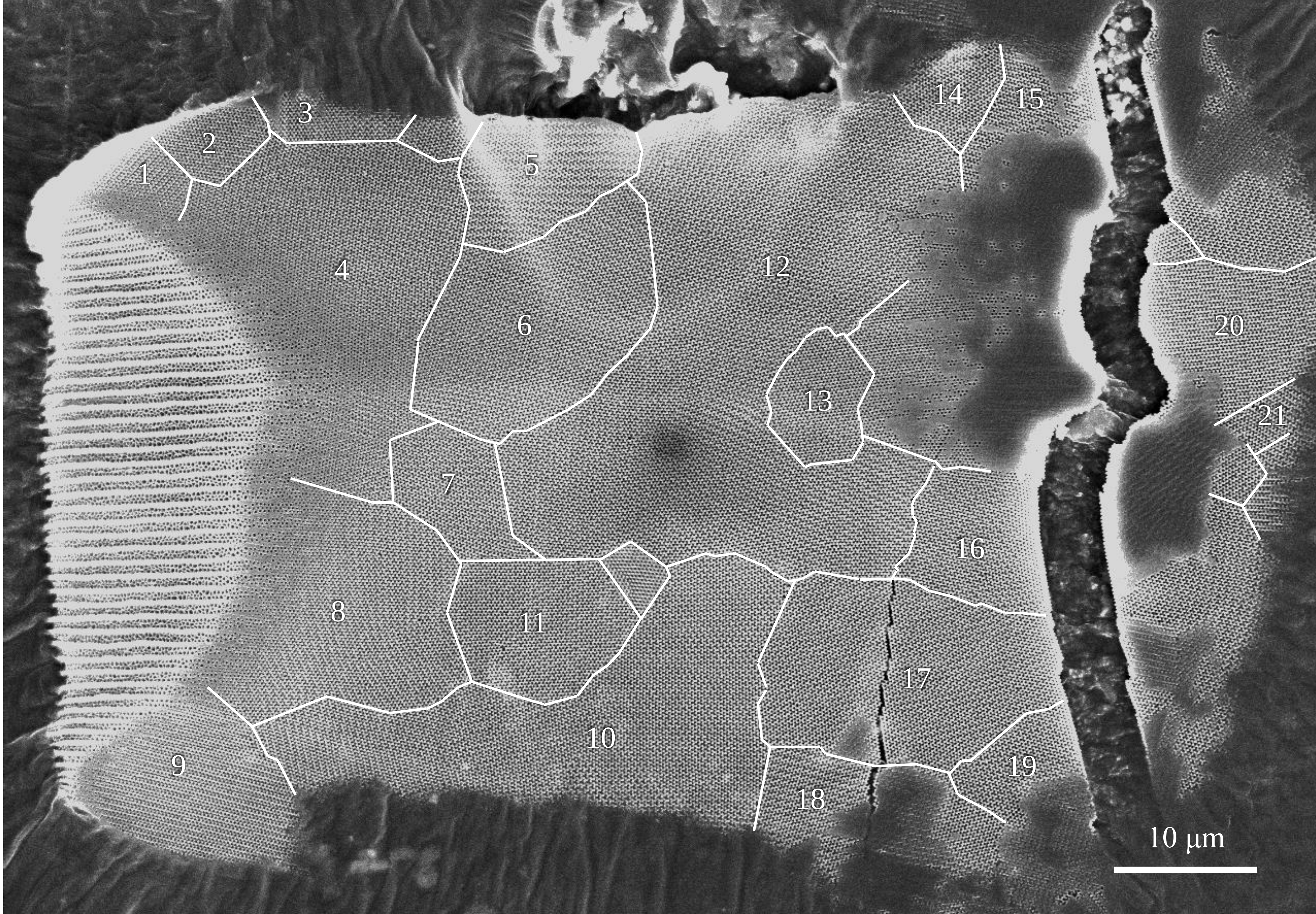

specimen No. 1
scale No. 1
domain No. 1
LH

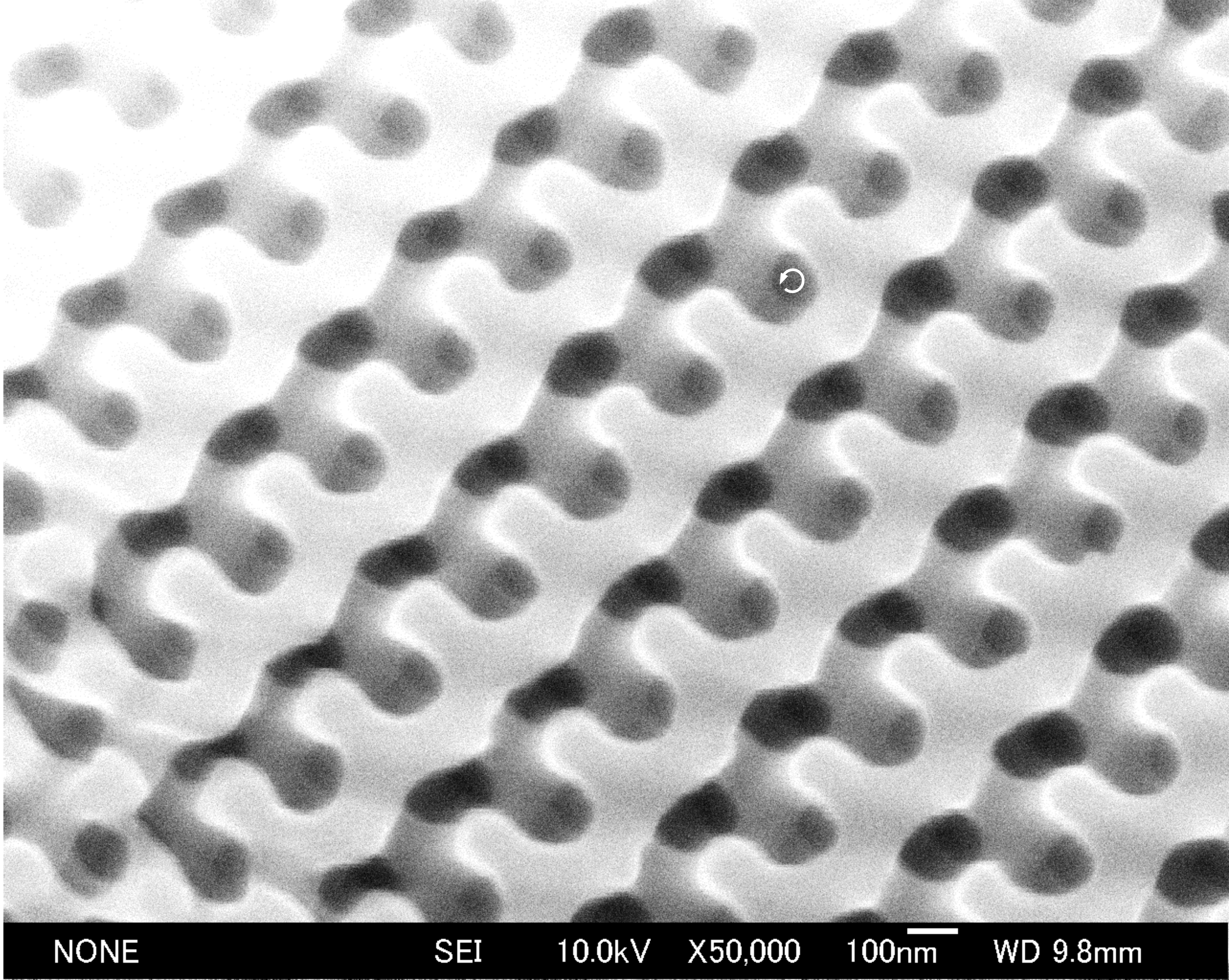

specimen No. 1
scale No. 1
domain No. 2
LH

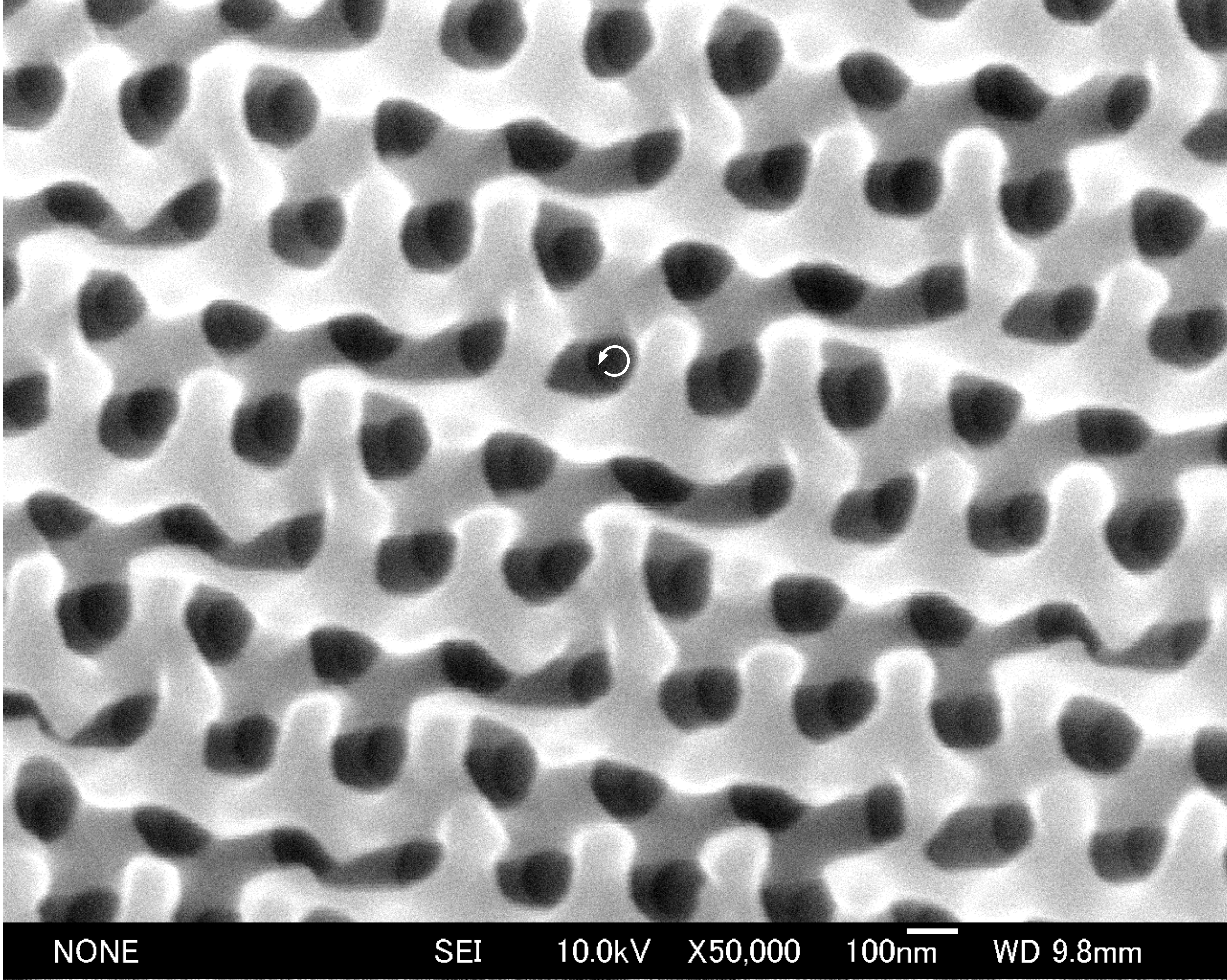

specimen No. 1
scale No. 1
domain No. 3
LH

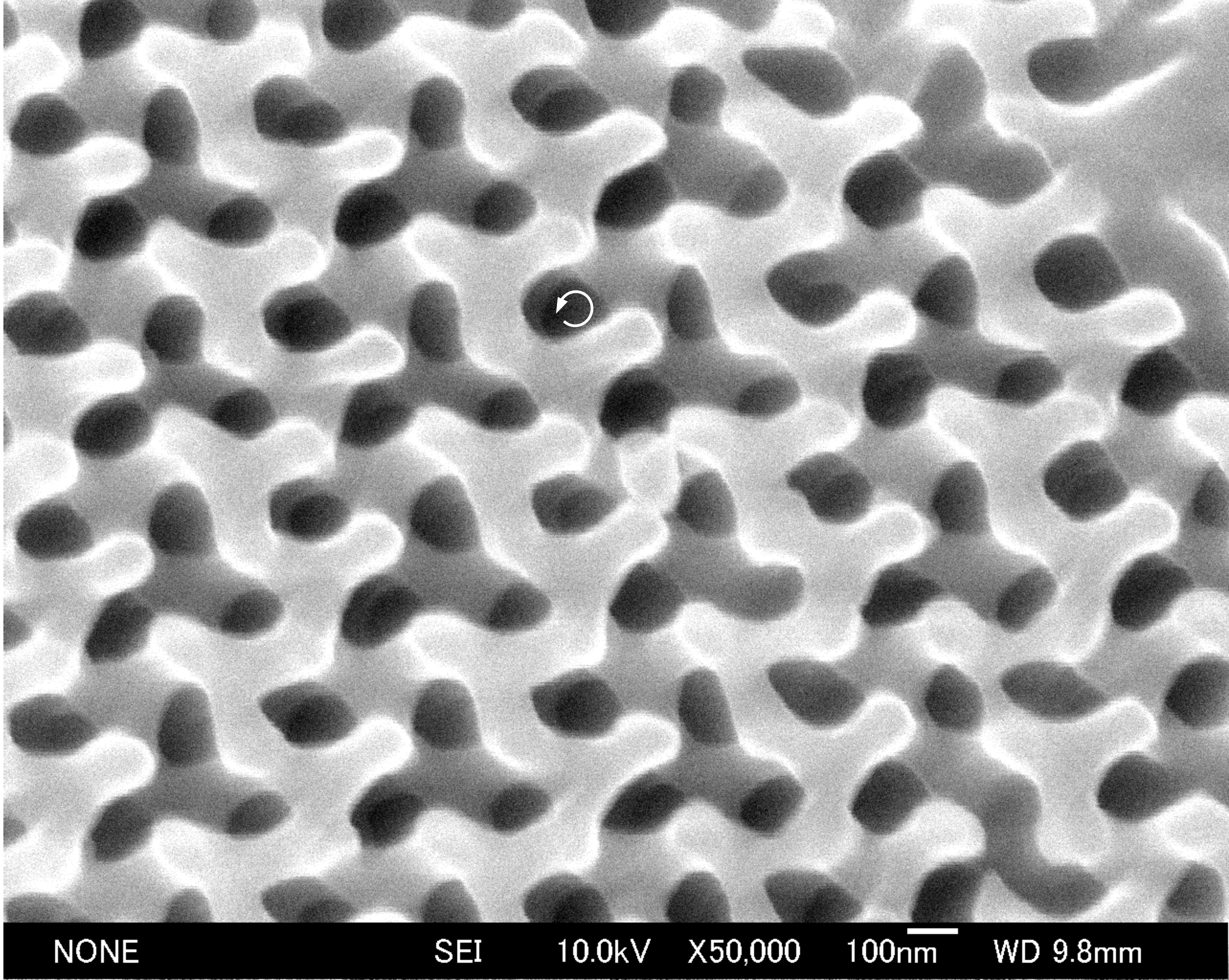

specimen No. 1
scale No. 1
domain No. 4
LH

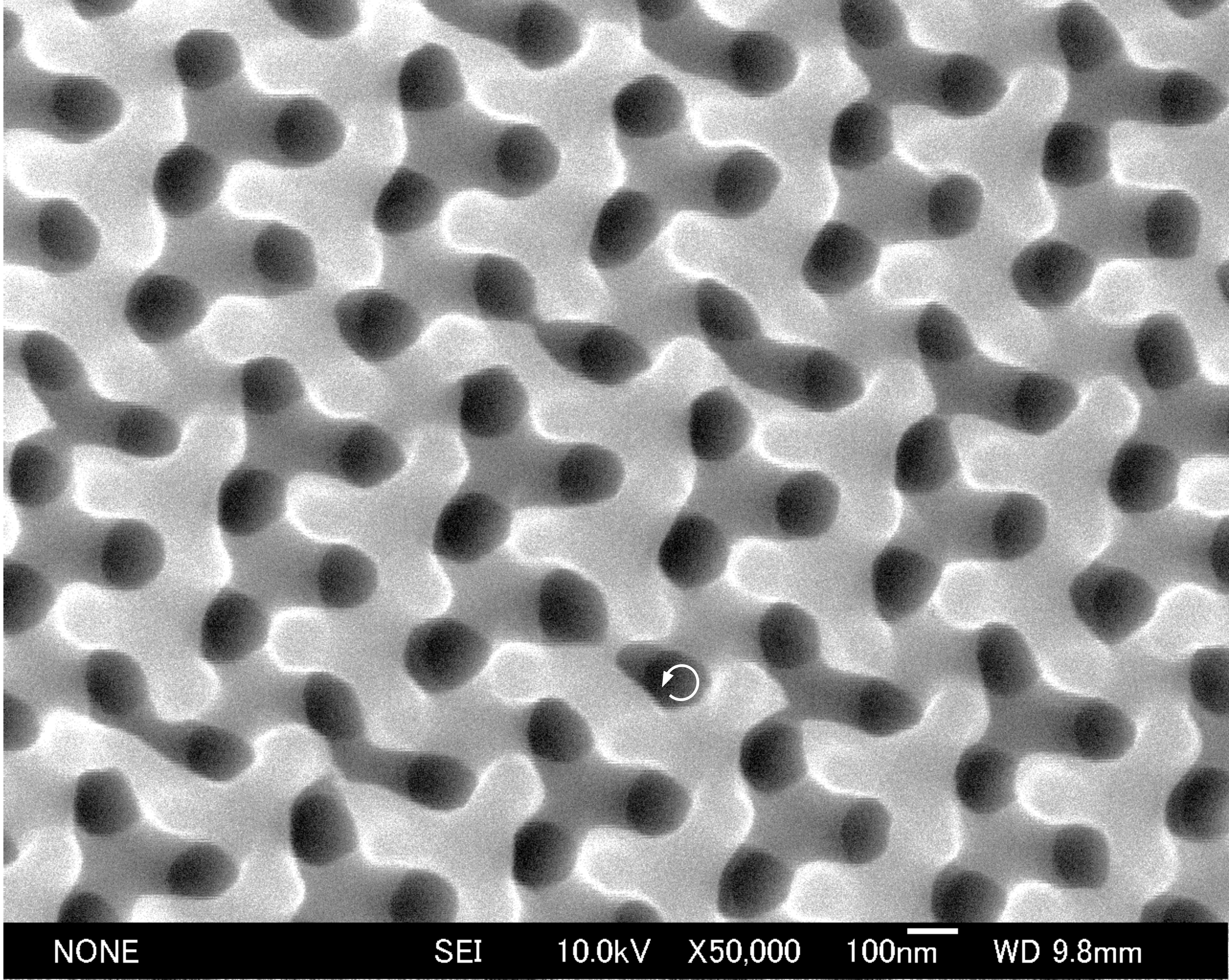

specimen No. 1
scale No. 1
domain No. 5
LH

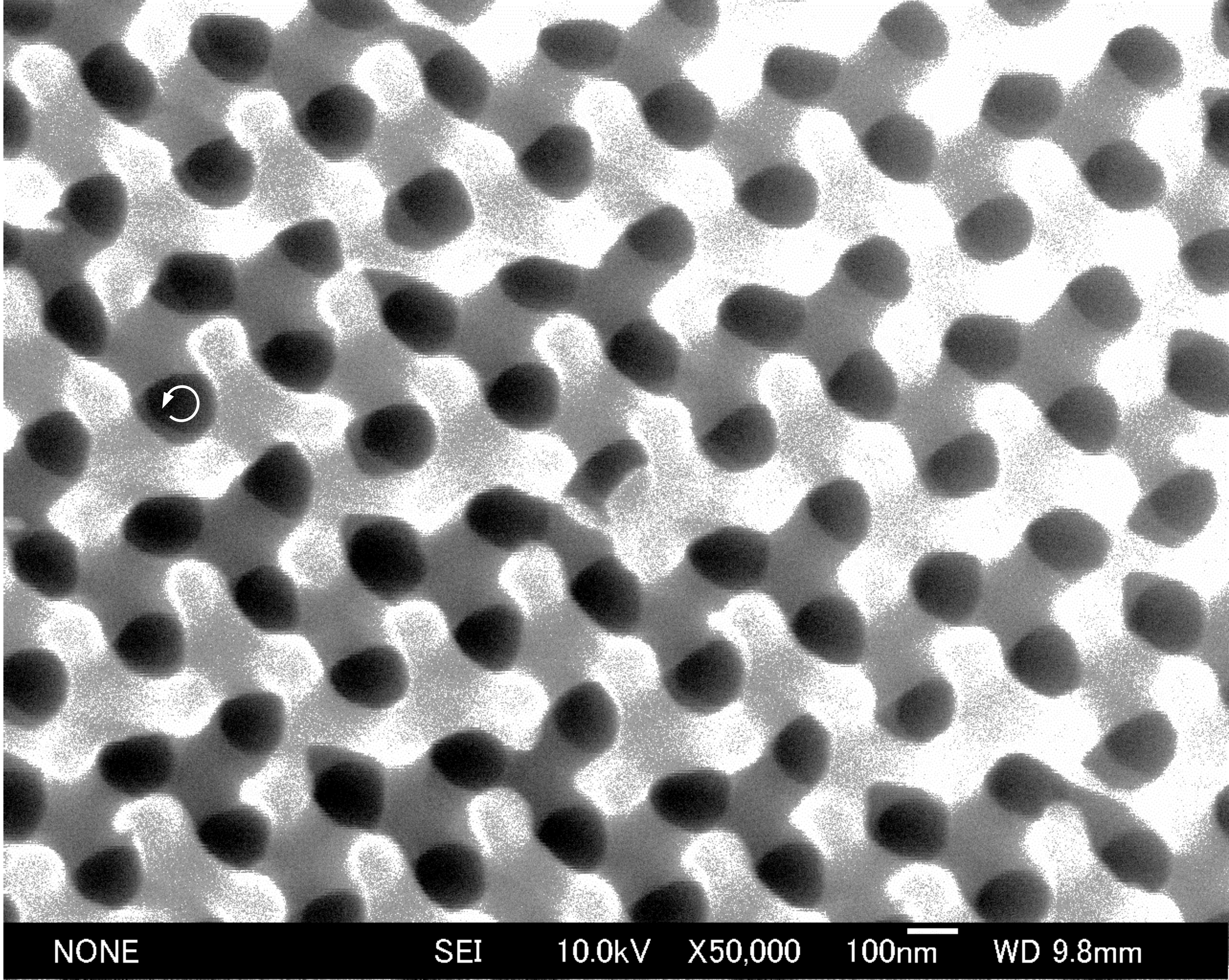

specimen No. 1
scale No. 1
domain No. 6
LH

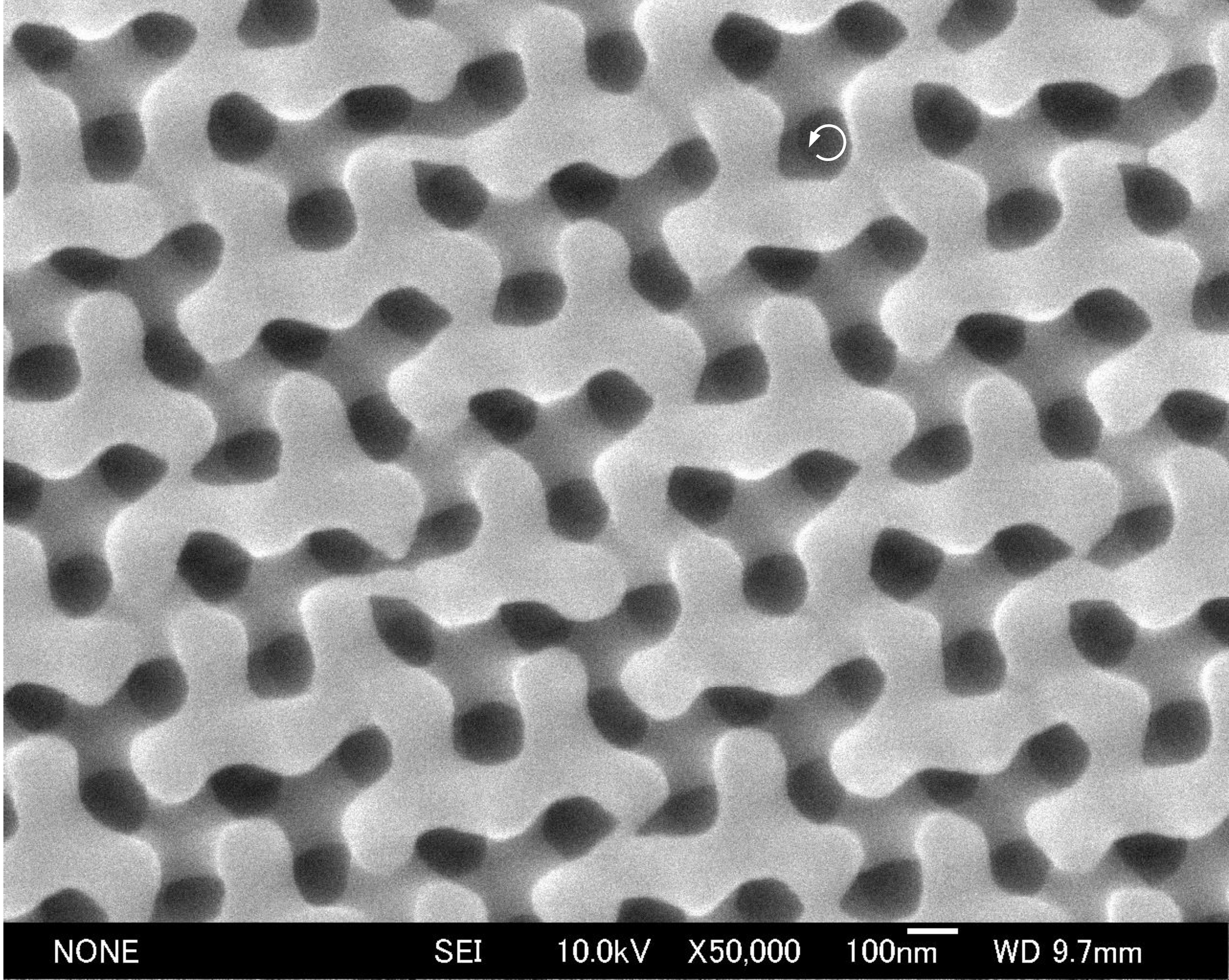

specimen No. 1
scale No. 1
domain No. 7
LH

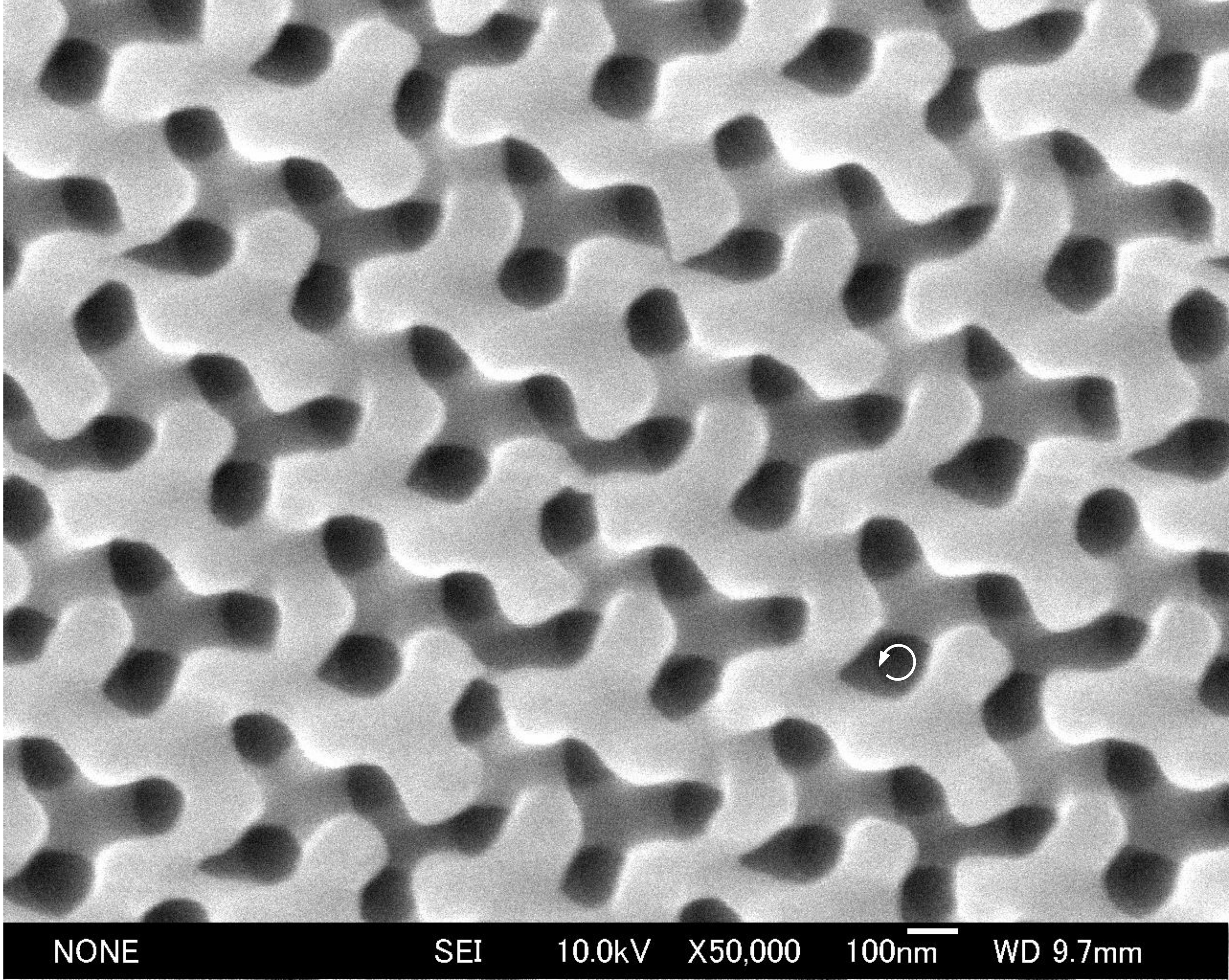

specimen No. 1
scale No. 1
domain No. 8
LH

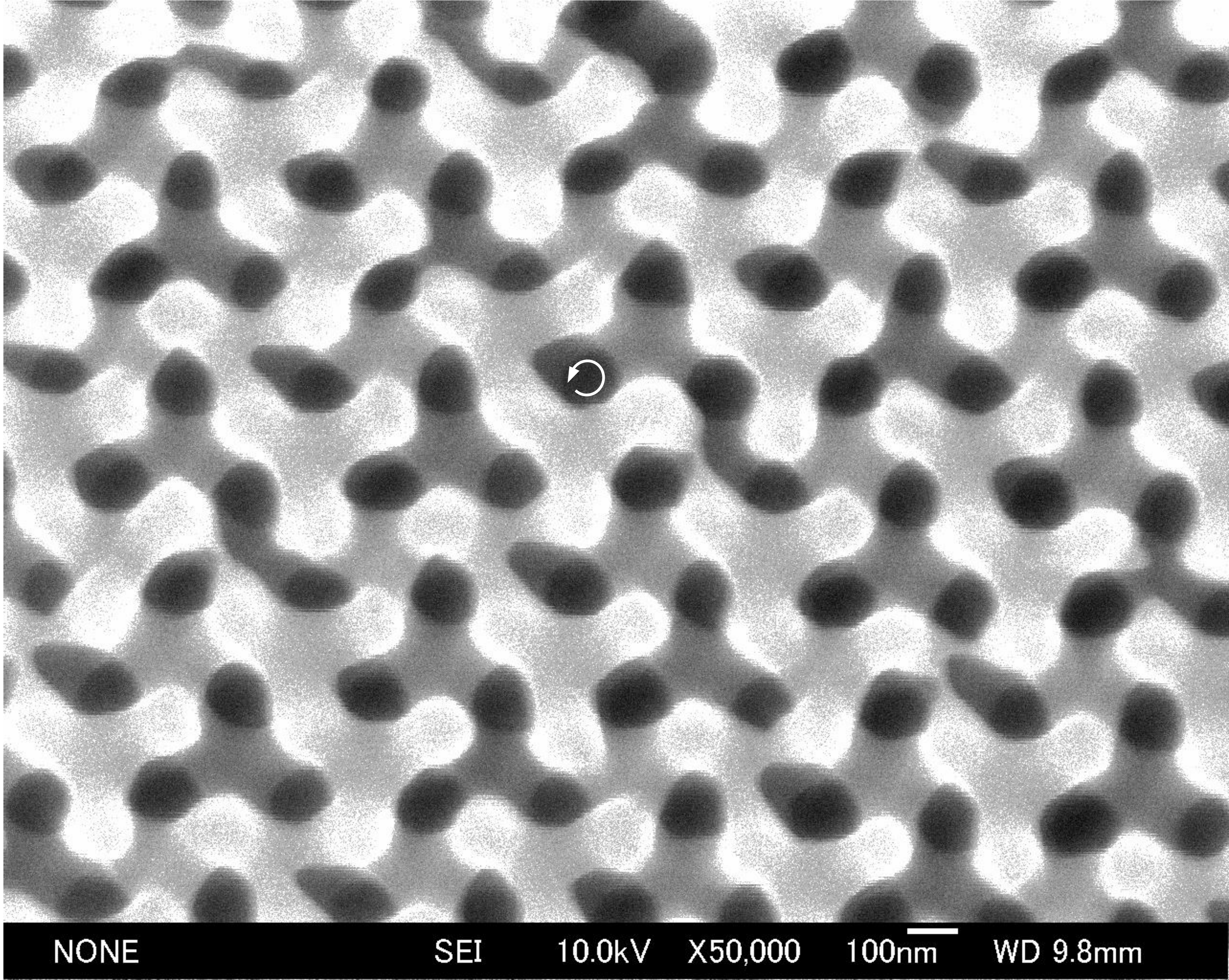

specimen No. 1
scale No. 1
domain No. 9
LH

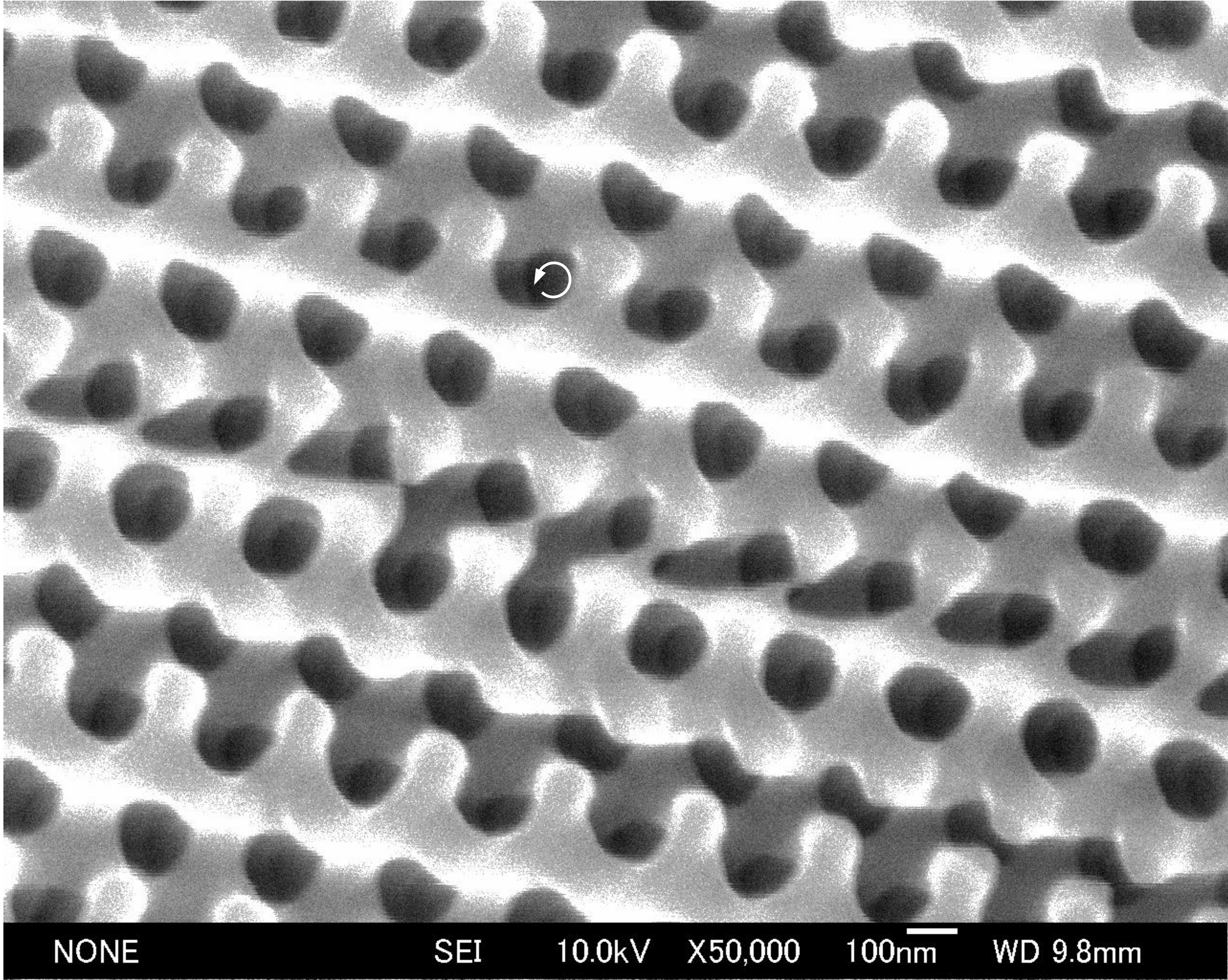

specimen No. 1
scale No. 1
domain No. 10
LH

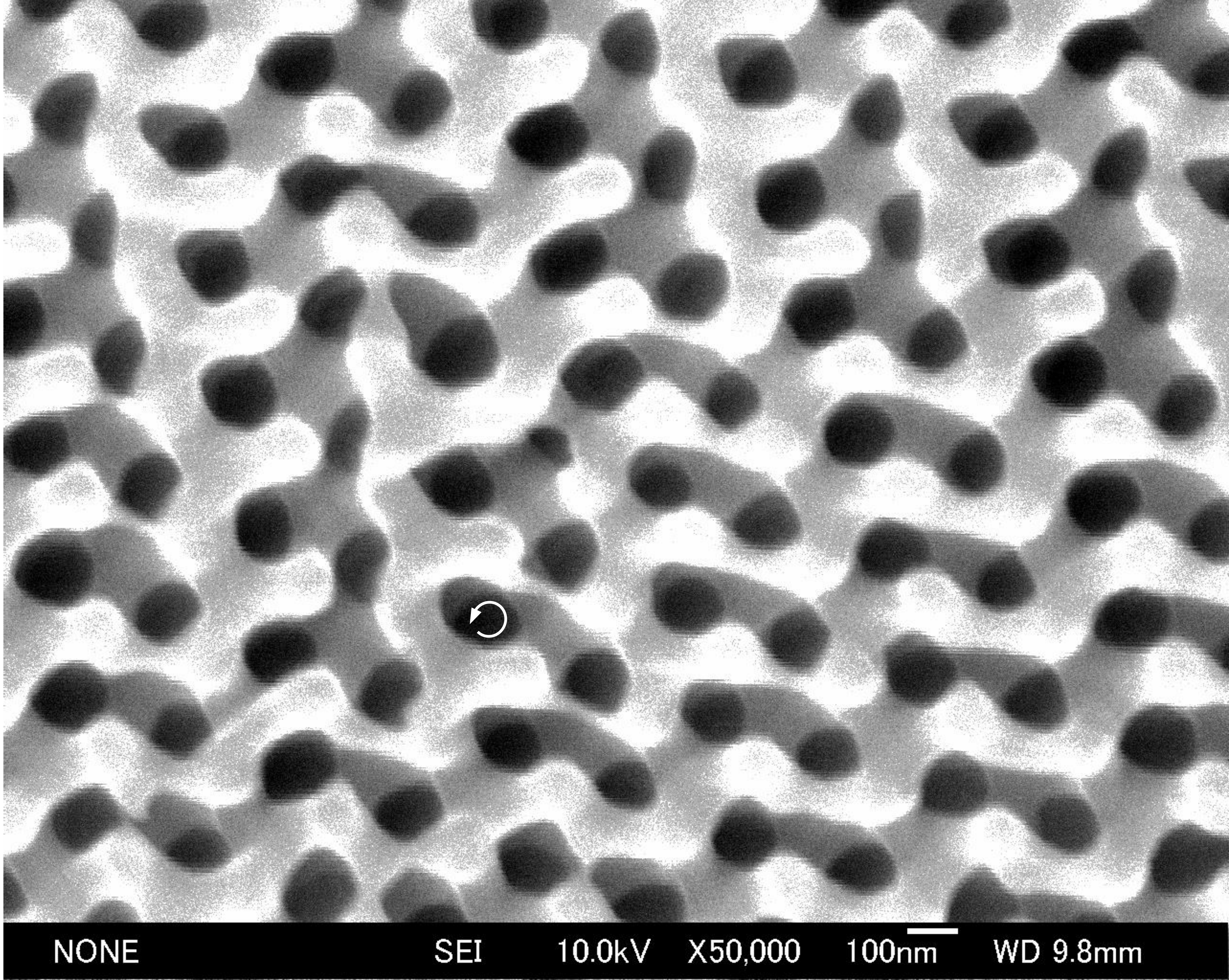

specimen No. 1
scale No. 1
domain No. 11
LH

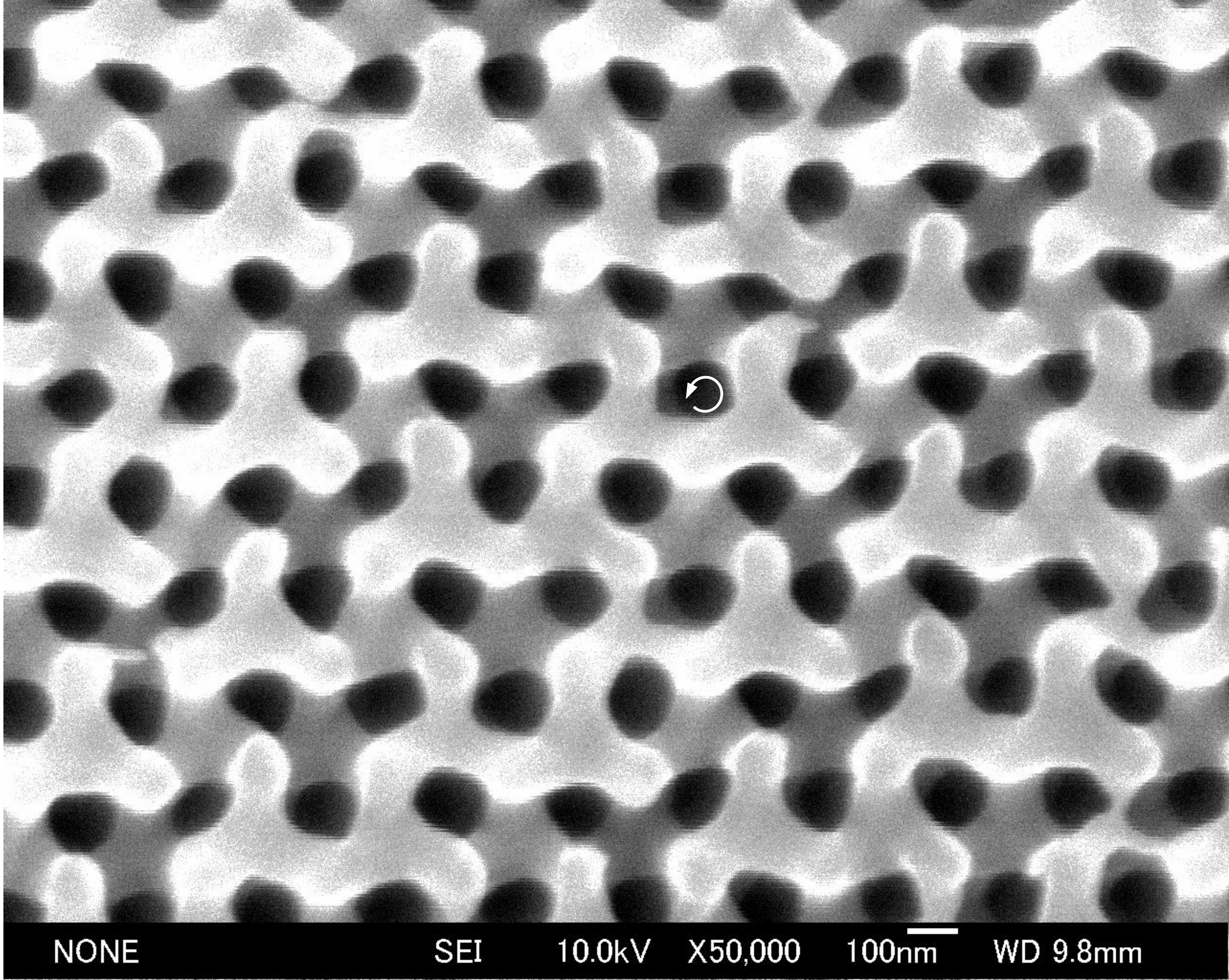

specimen No. 1
scale No. 1
domain No. 12
LH

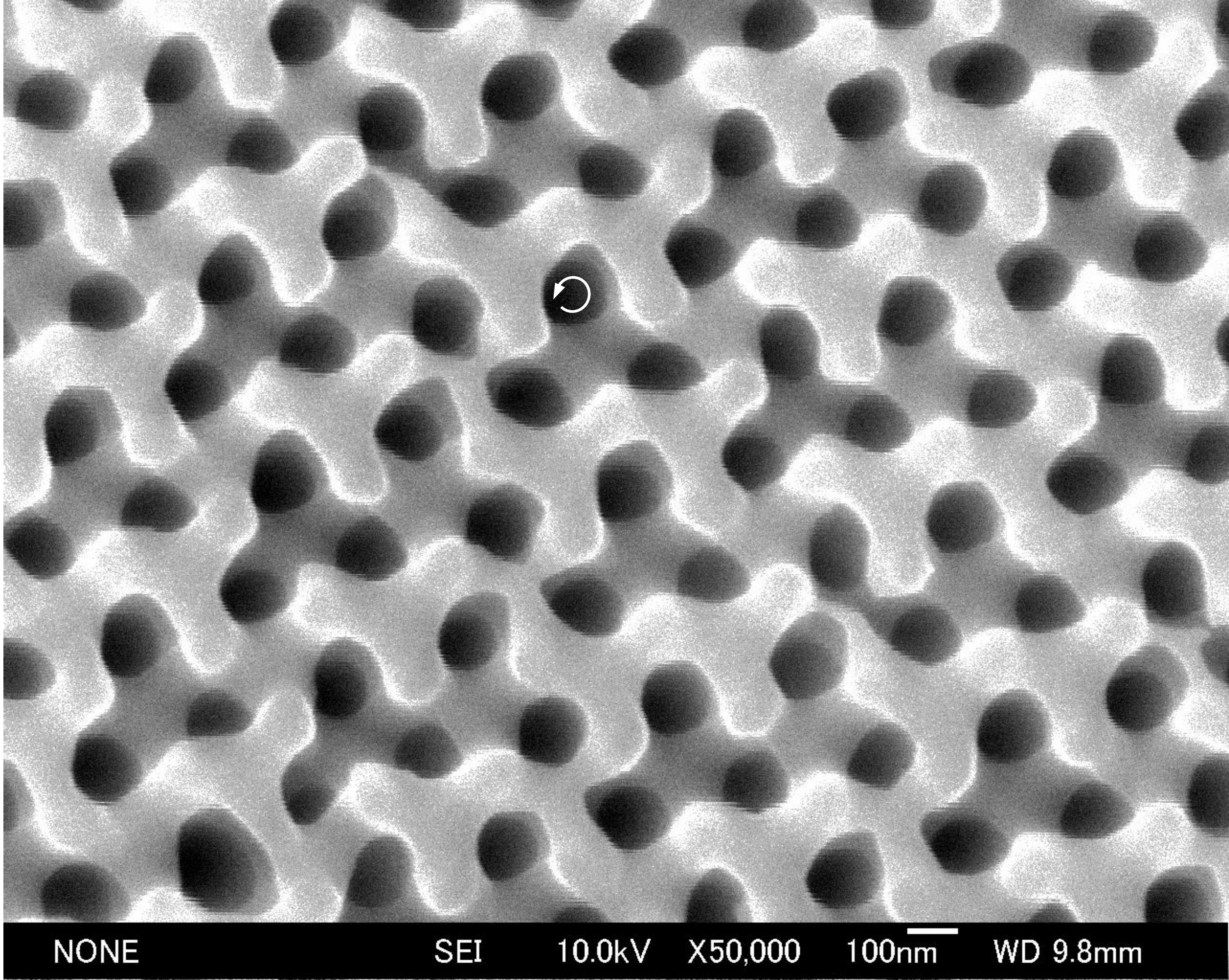

specimen No. 1
scale No. 1
domain No. 13
LH

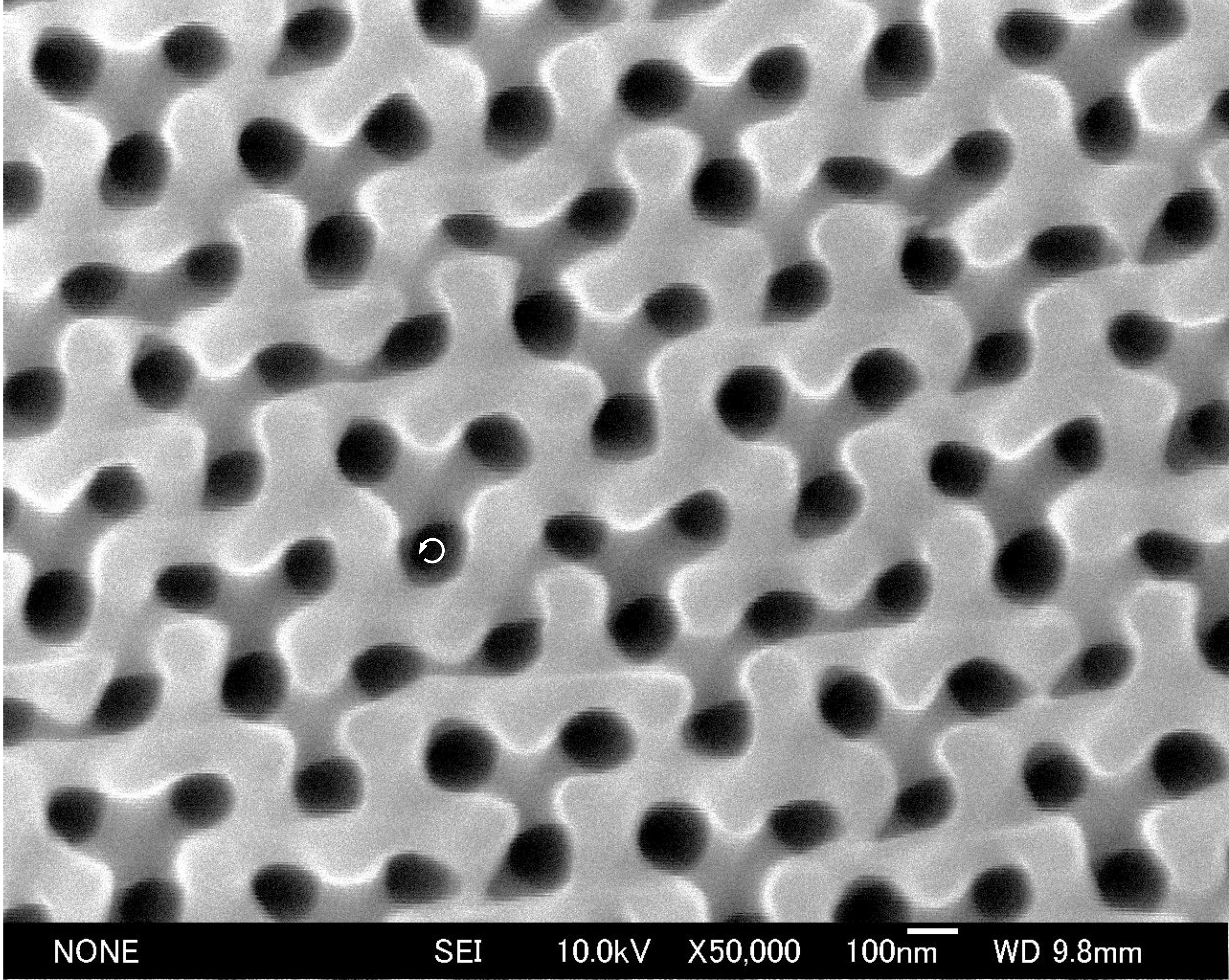

specimen No. 1
scale No. 1
domain No. 14
LH

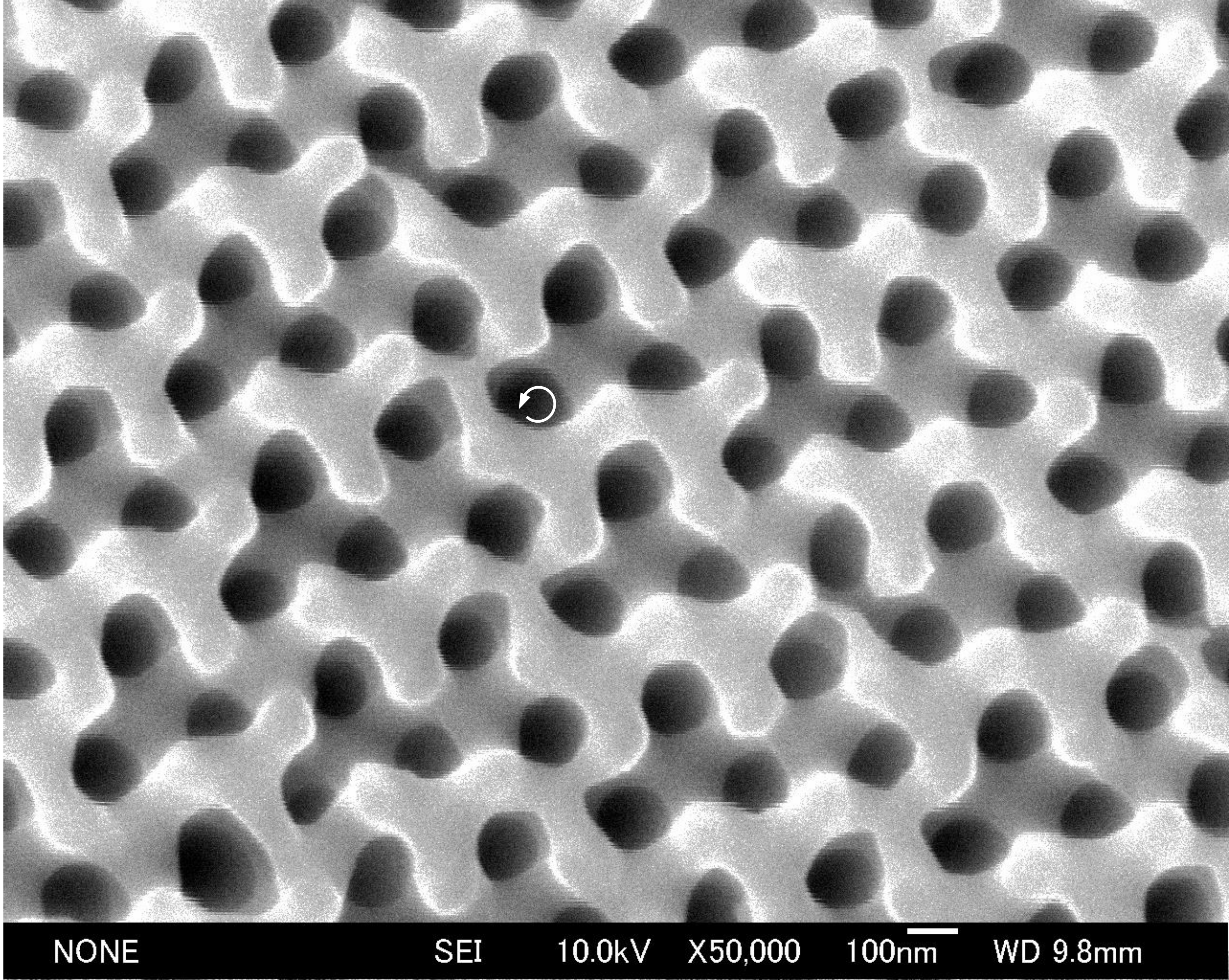

specimen No. 1
scale No. 1
domain No. 15
LH

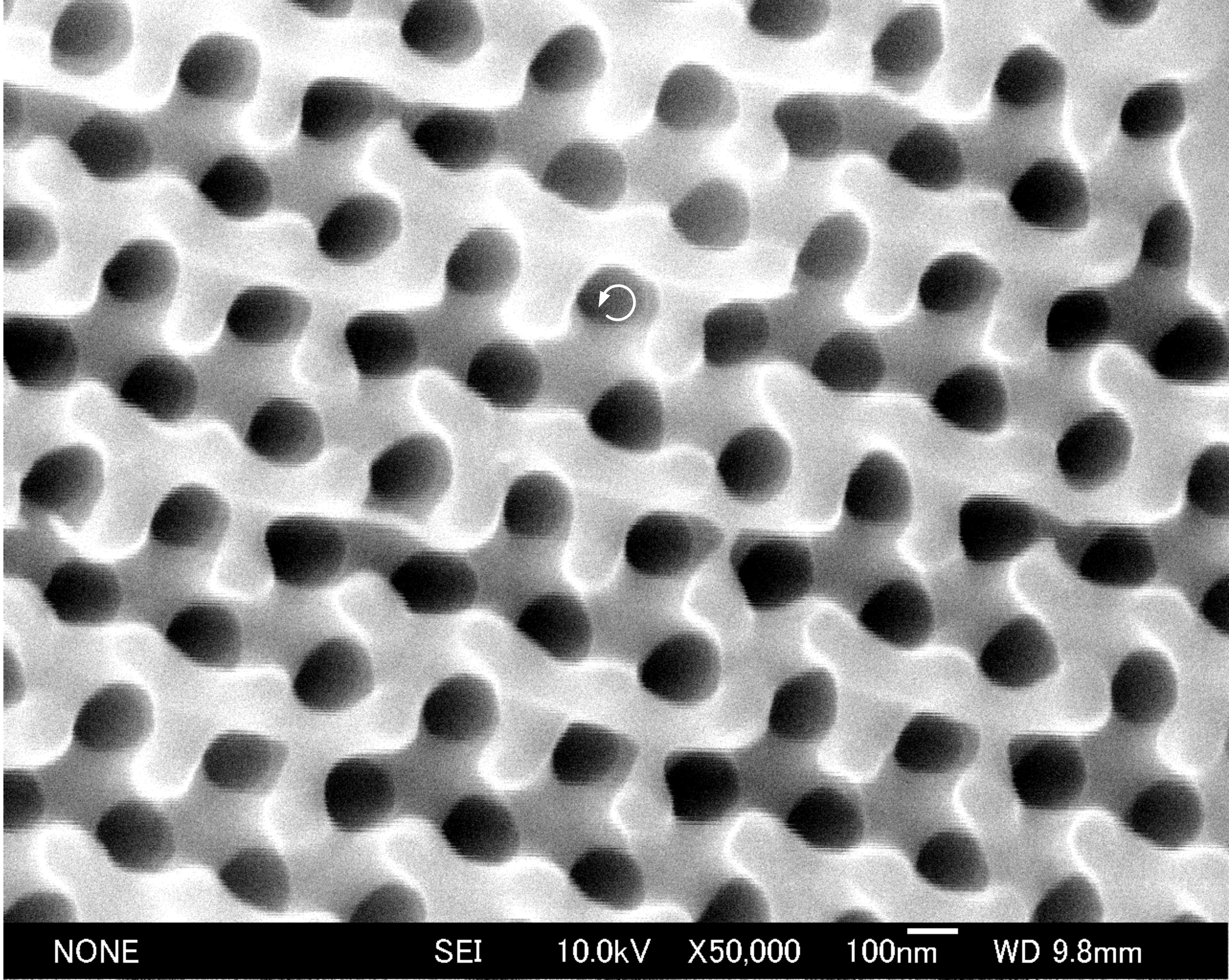

specimen No. 1
scale No. 1
domain No. 16
LH

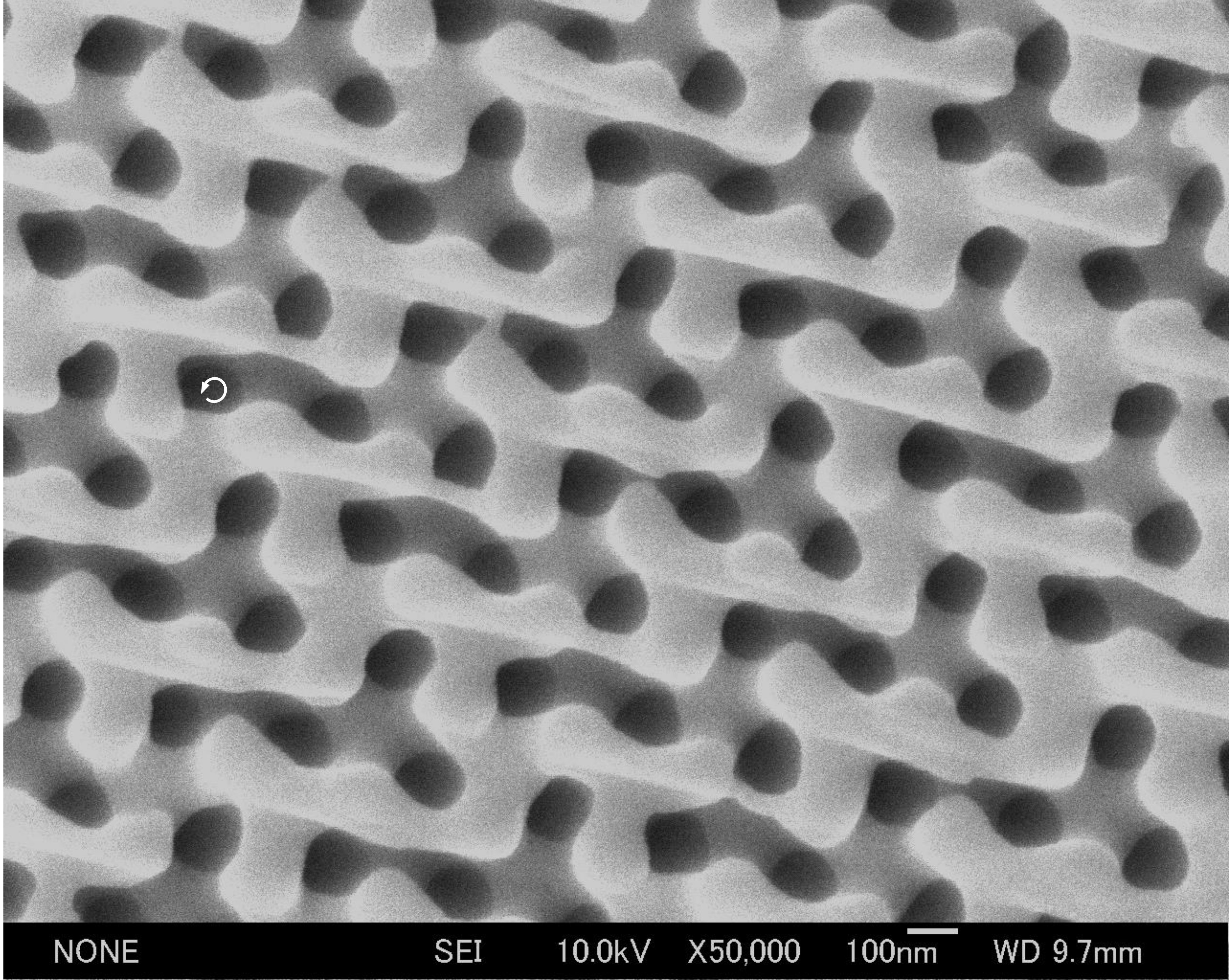

specimen No. 1
scale No. 1
domain No. 17
LH

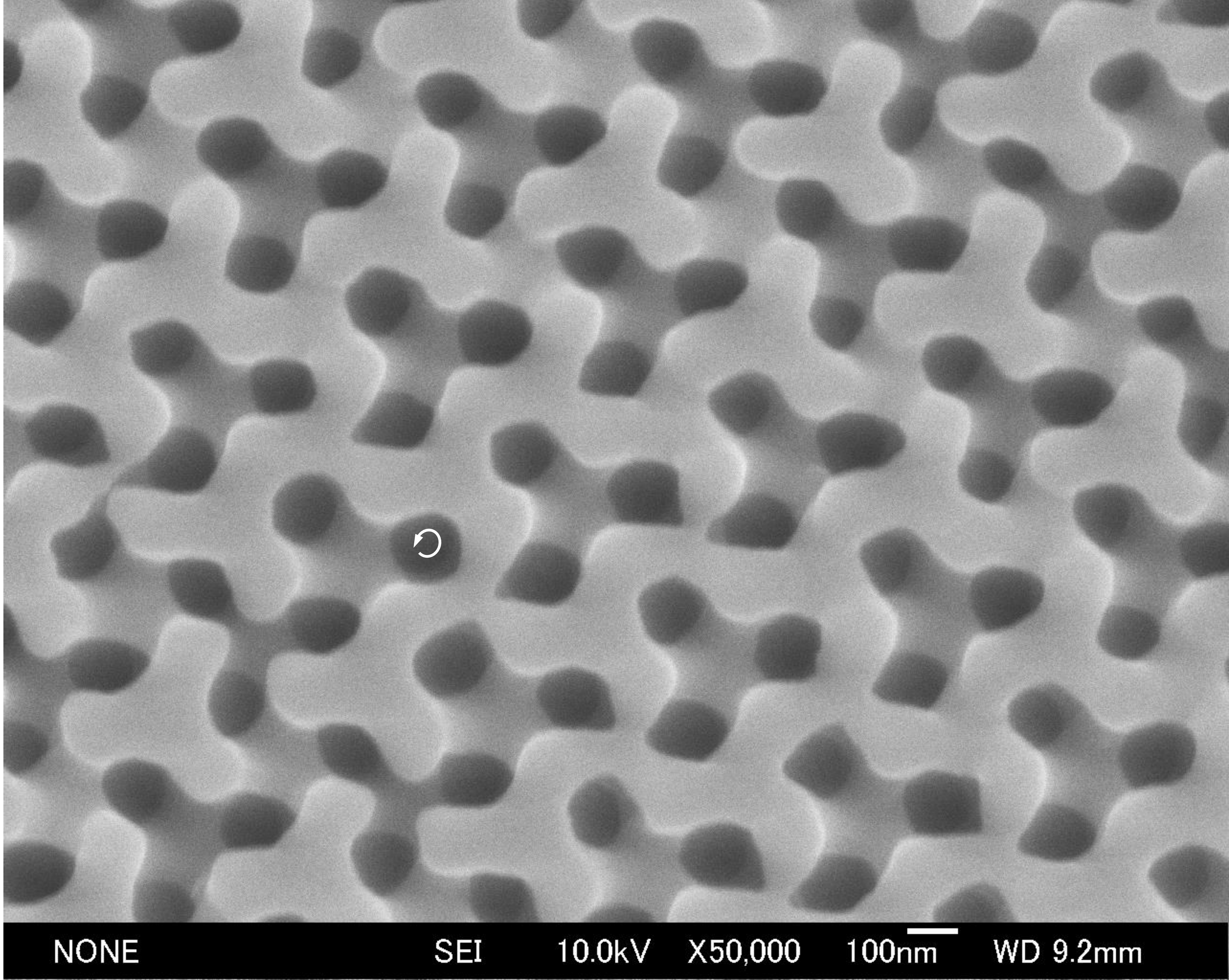

specimen No. 1
scale No. 1
domain No. 18
LH

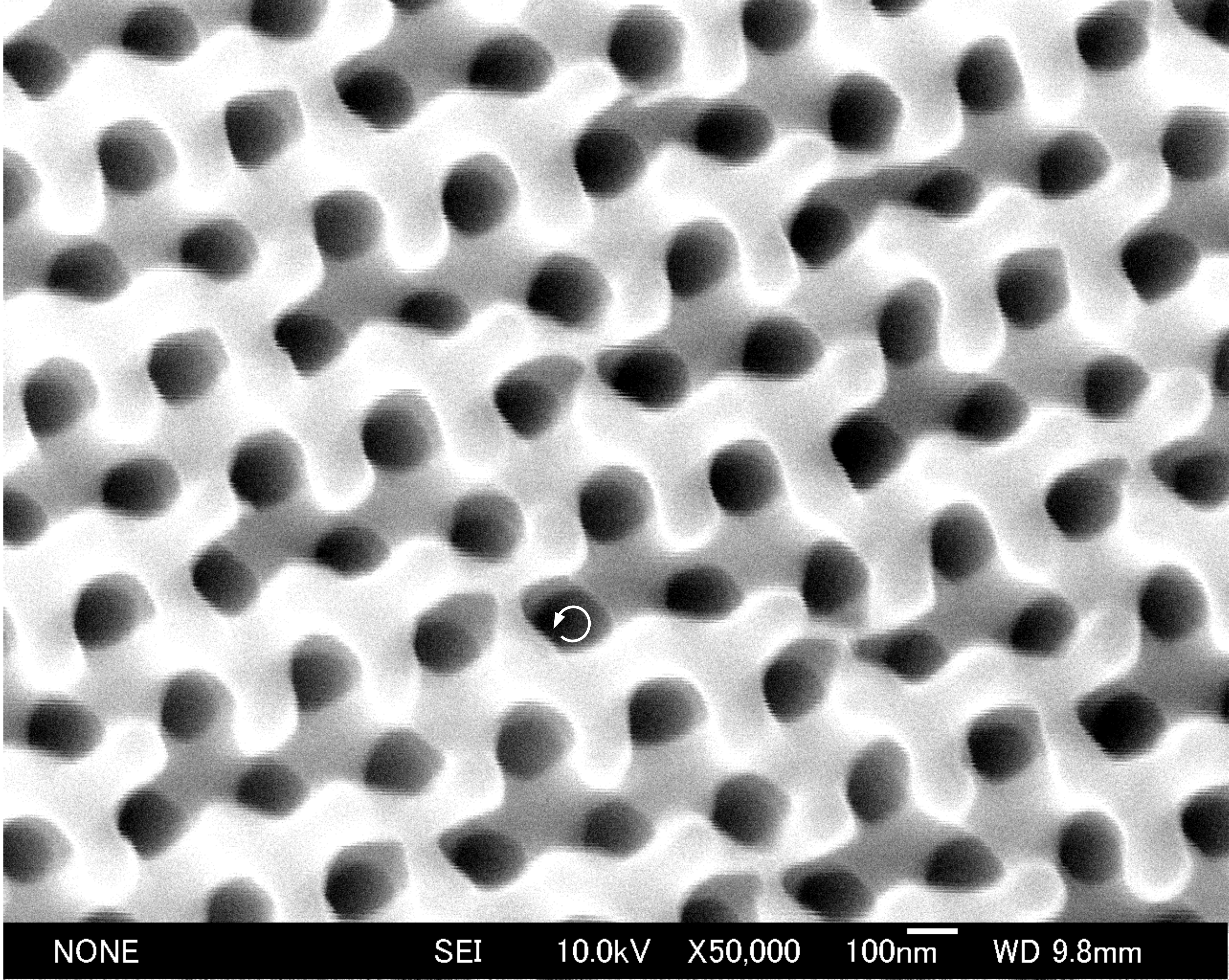

specimen No. 1
scale No. 1
domain No. 19
LH

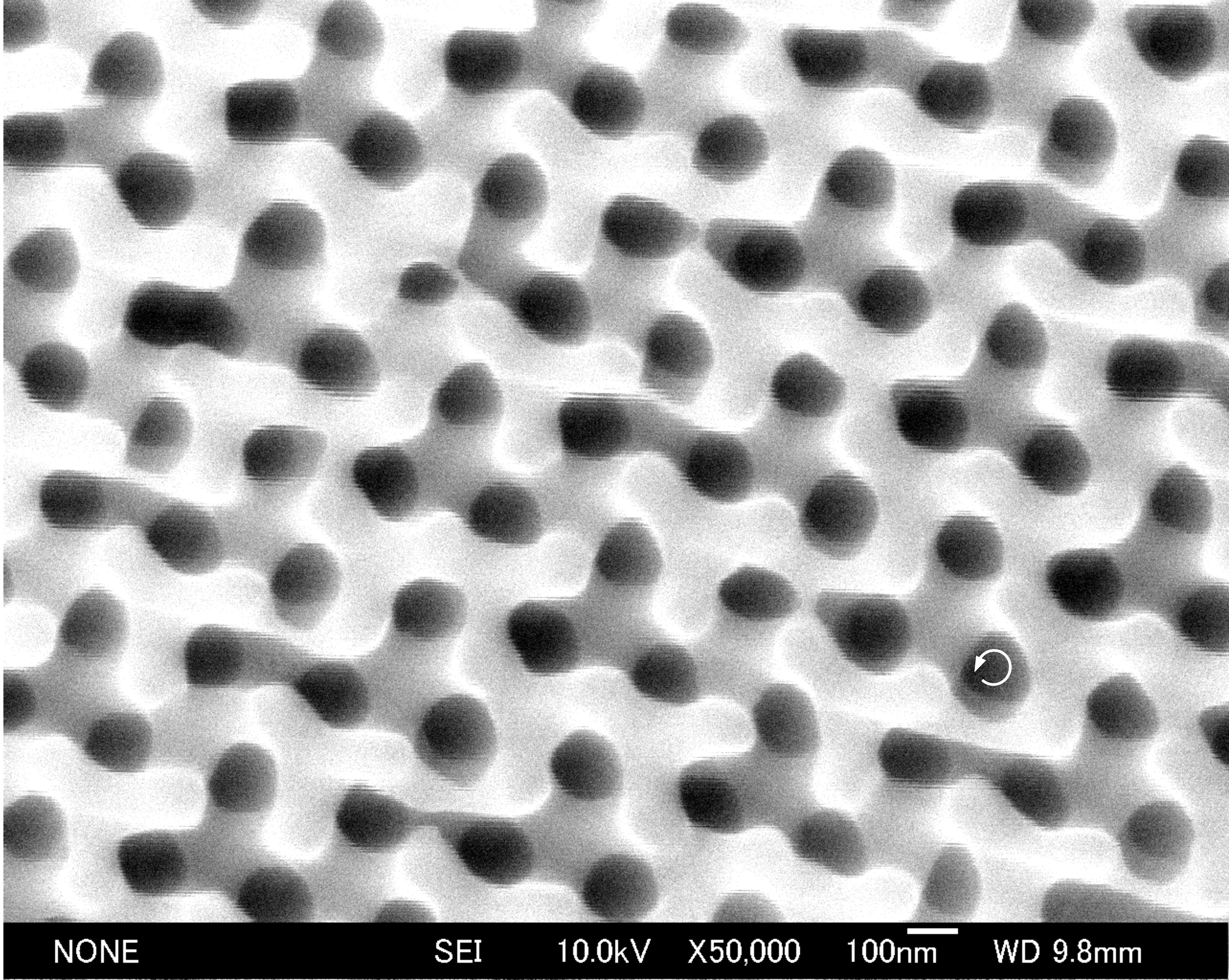

specimen No. 1
scale No. 1
domain No. 20
LH

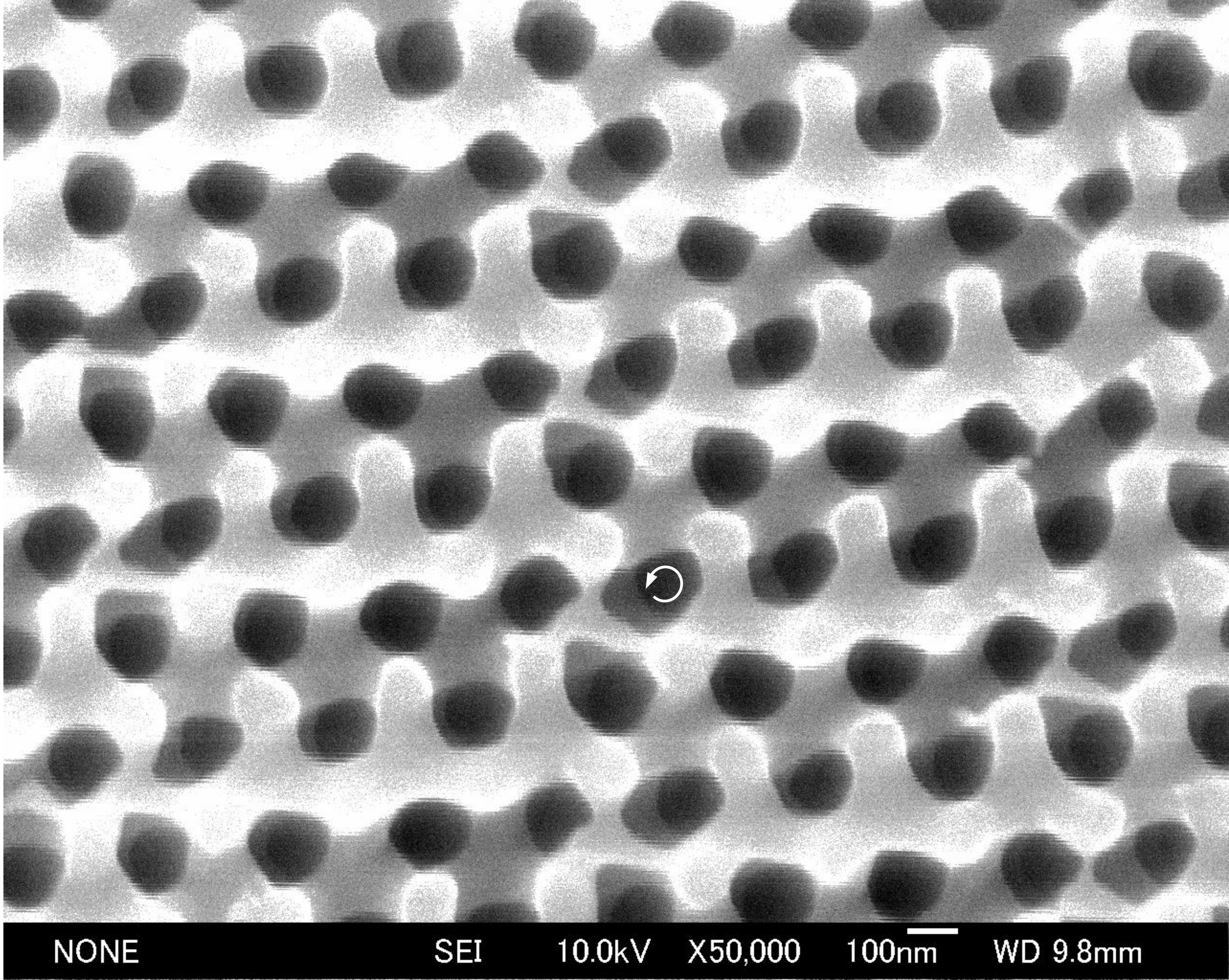

specimen No. 1
scale No. 1
domain No. 21
LH

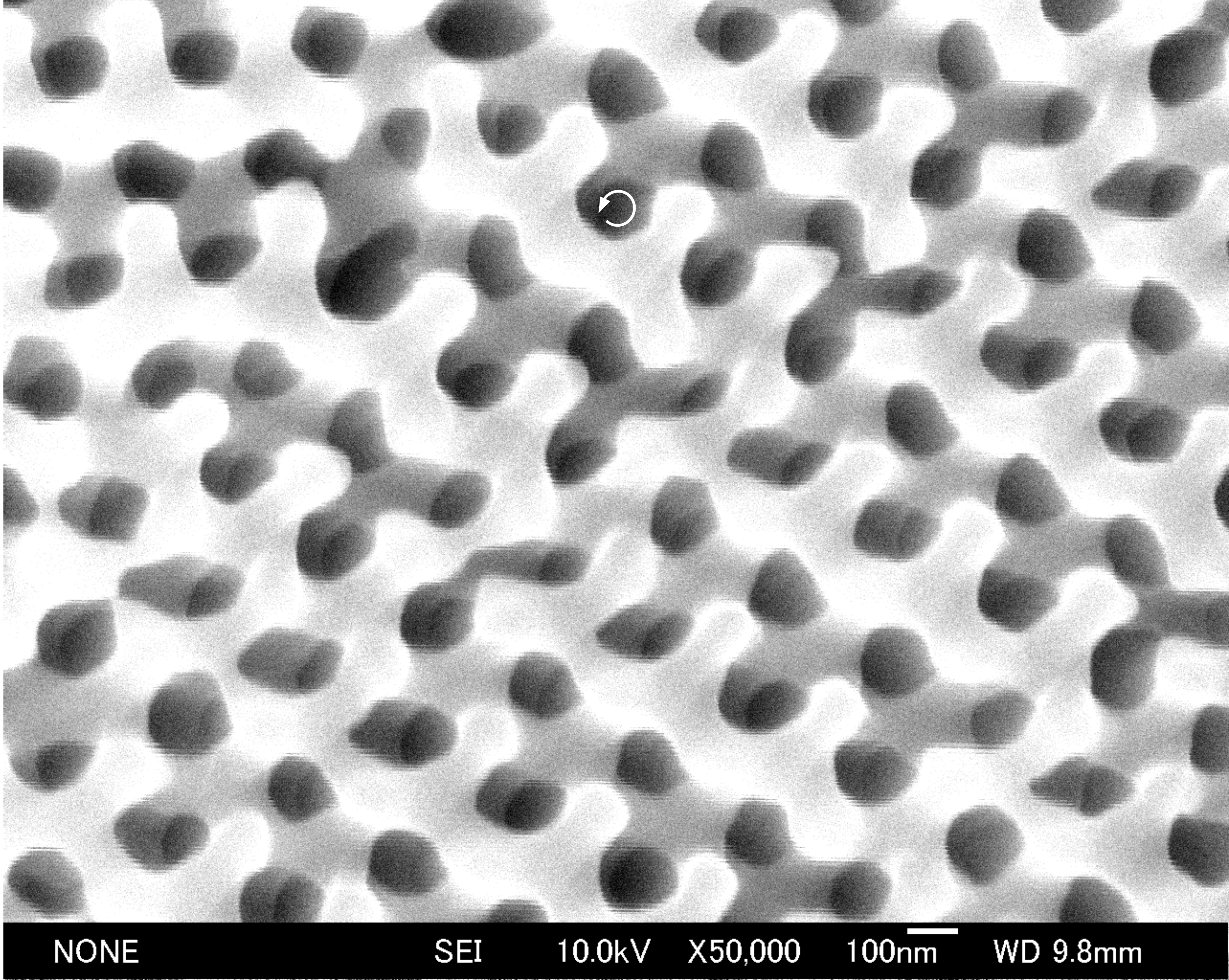

specimen No. 1
scale No. 2

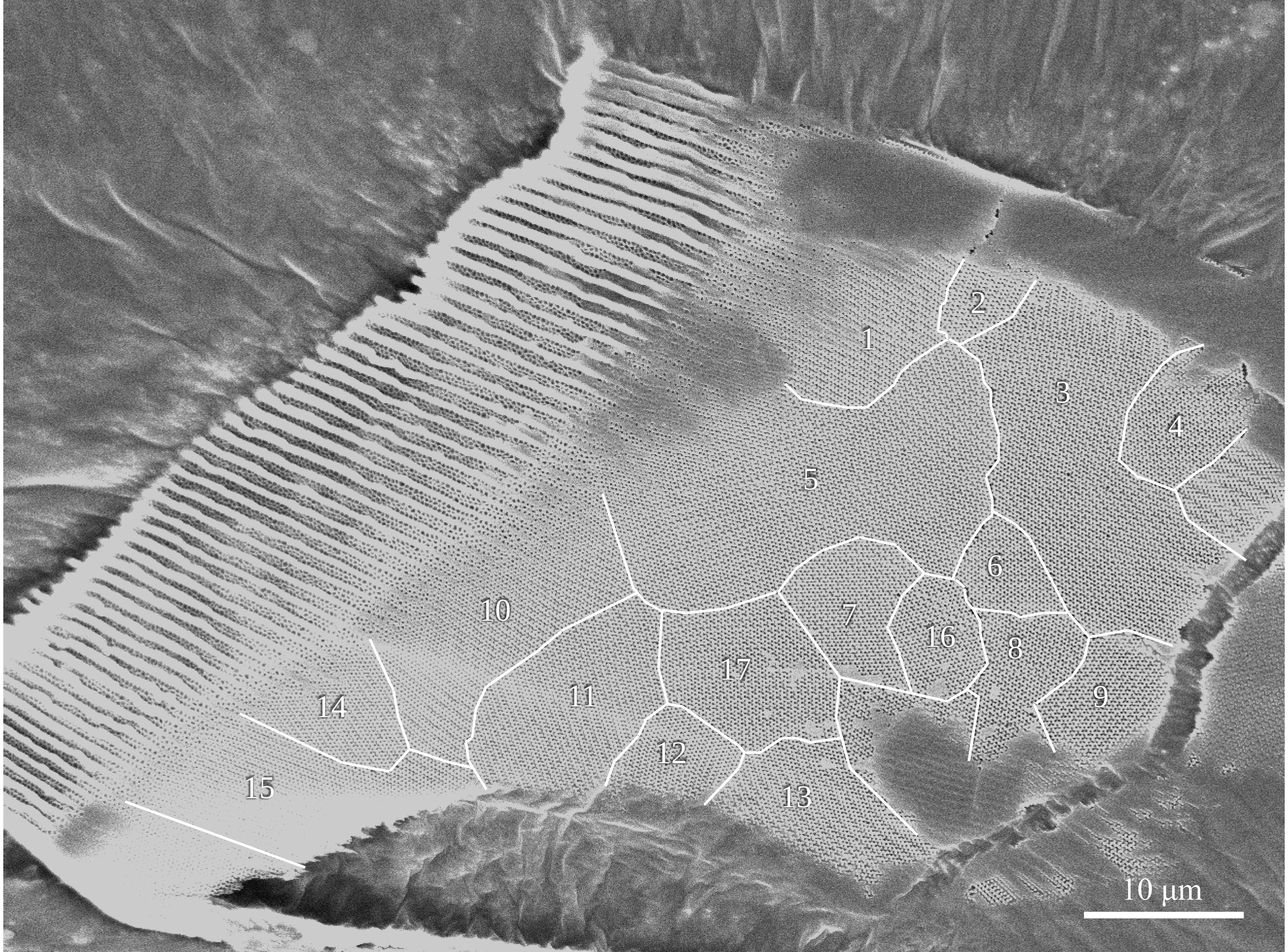

specimen No. 1
scale No. 2
domain No. 1
LH

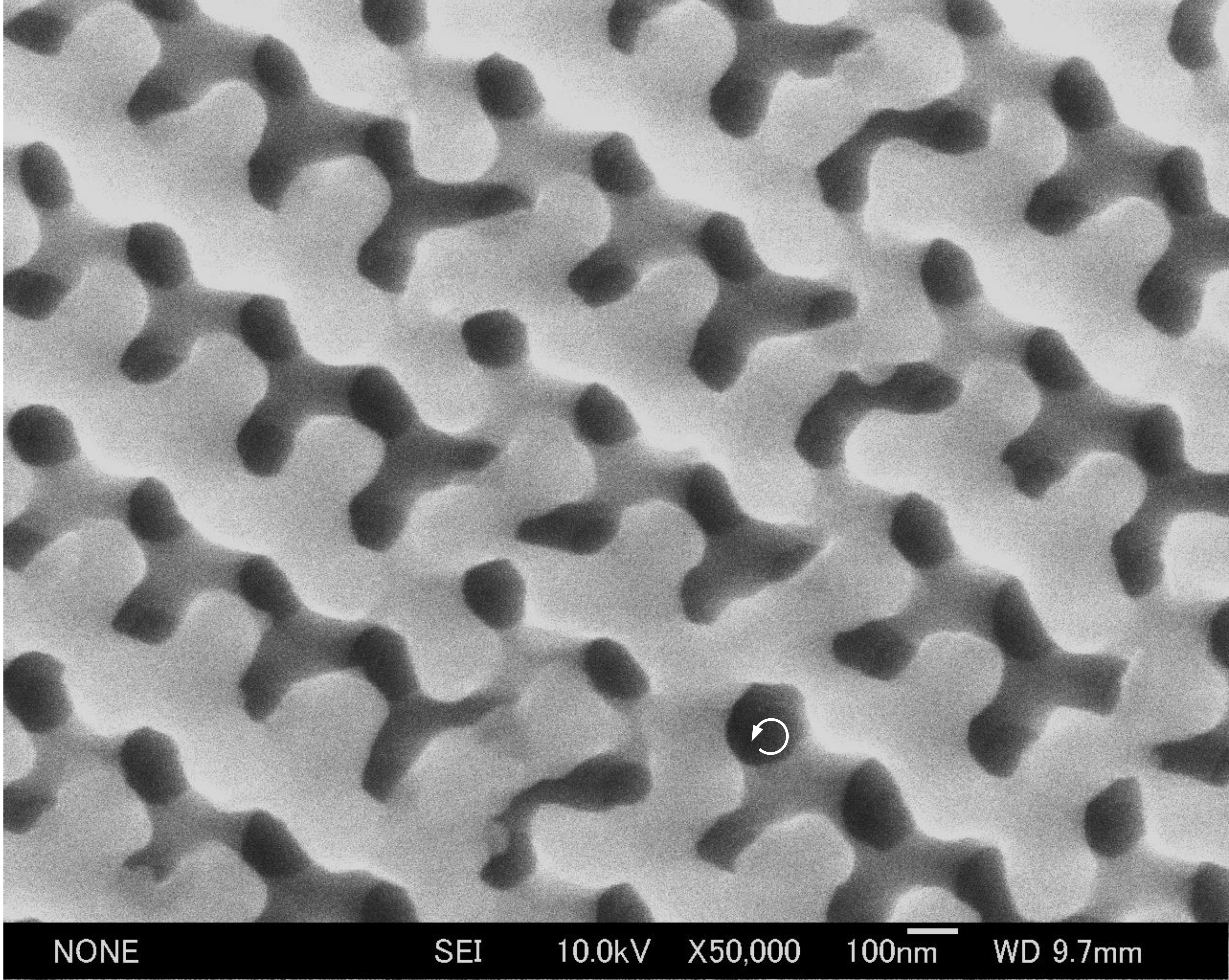

specimen No. 1
scale No. 2
domain No. 2
LH

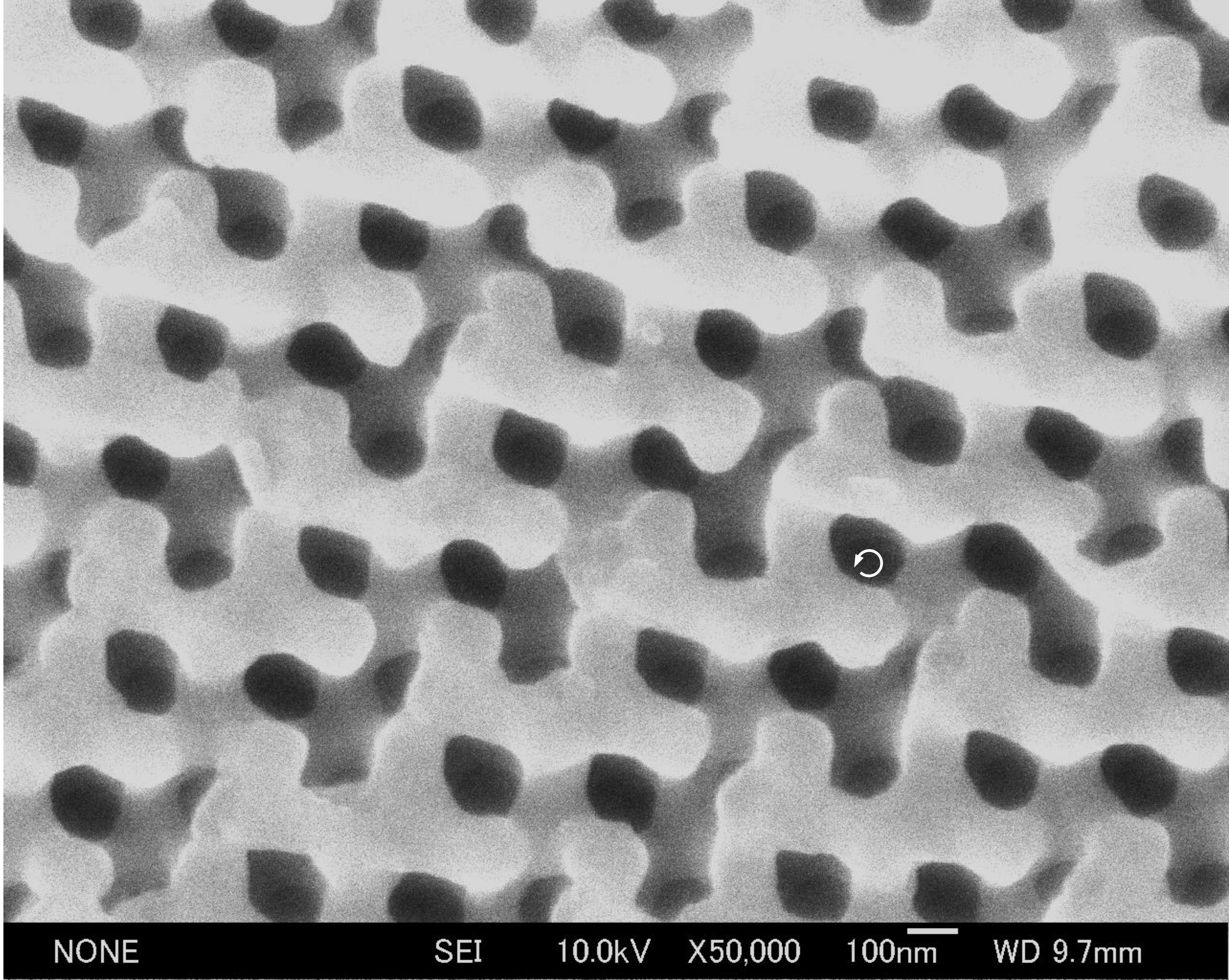

specimen No. 1
scale No. 2
domain No. 3
LH

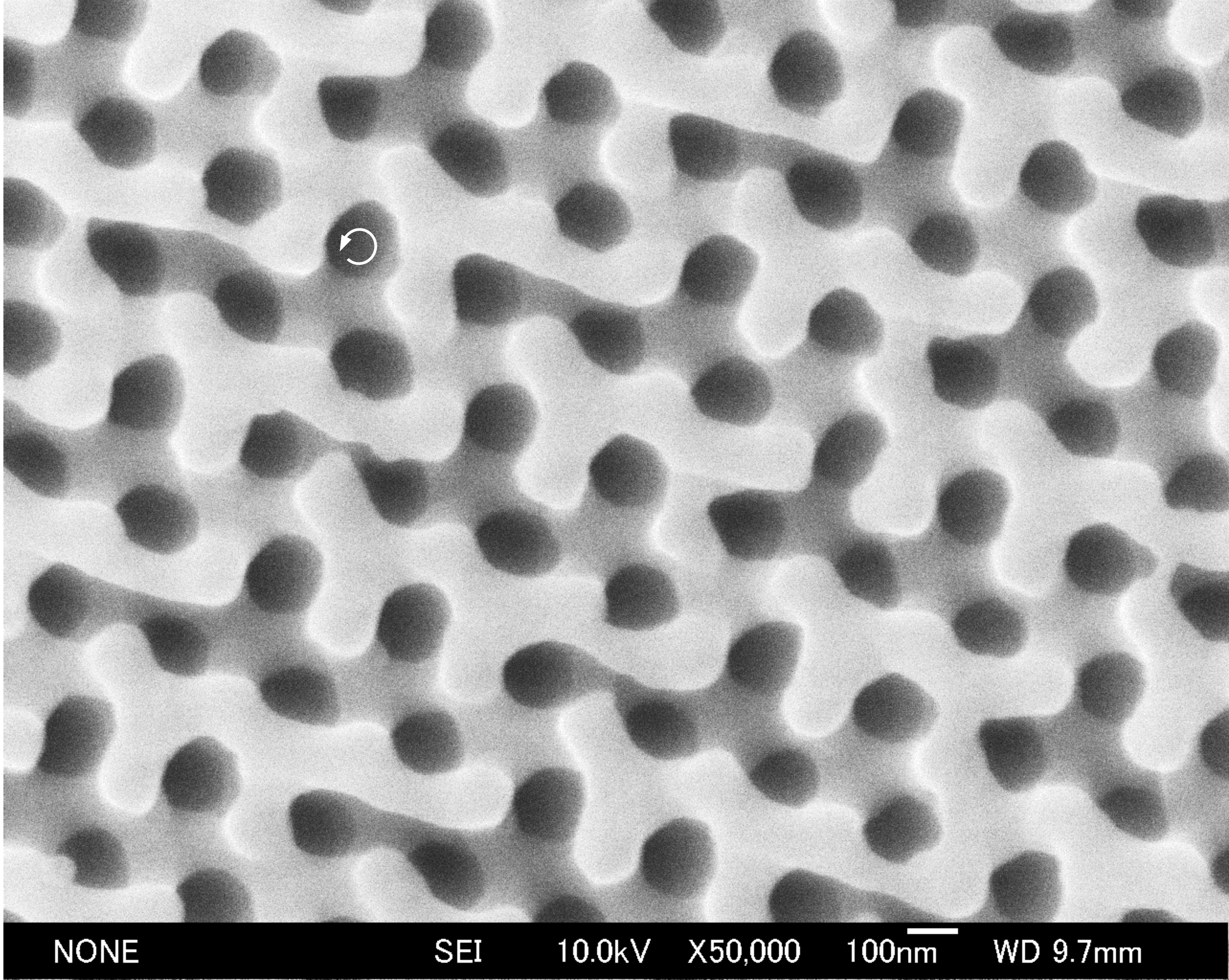

specimen No. 1
scale No. 2
domain No. 4
LH

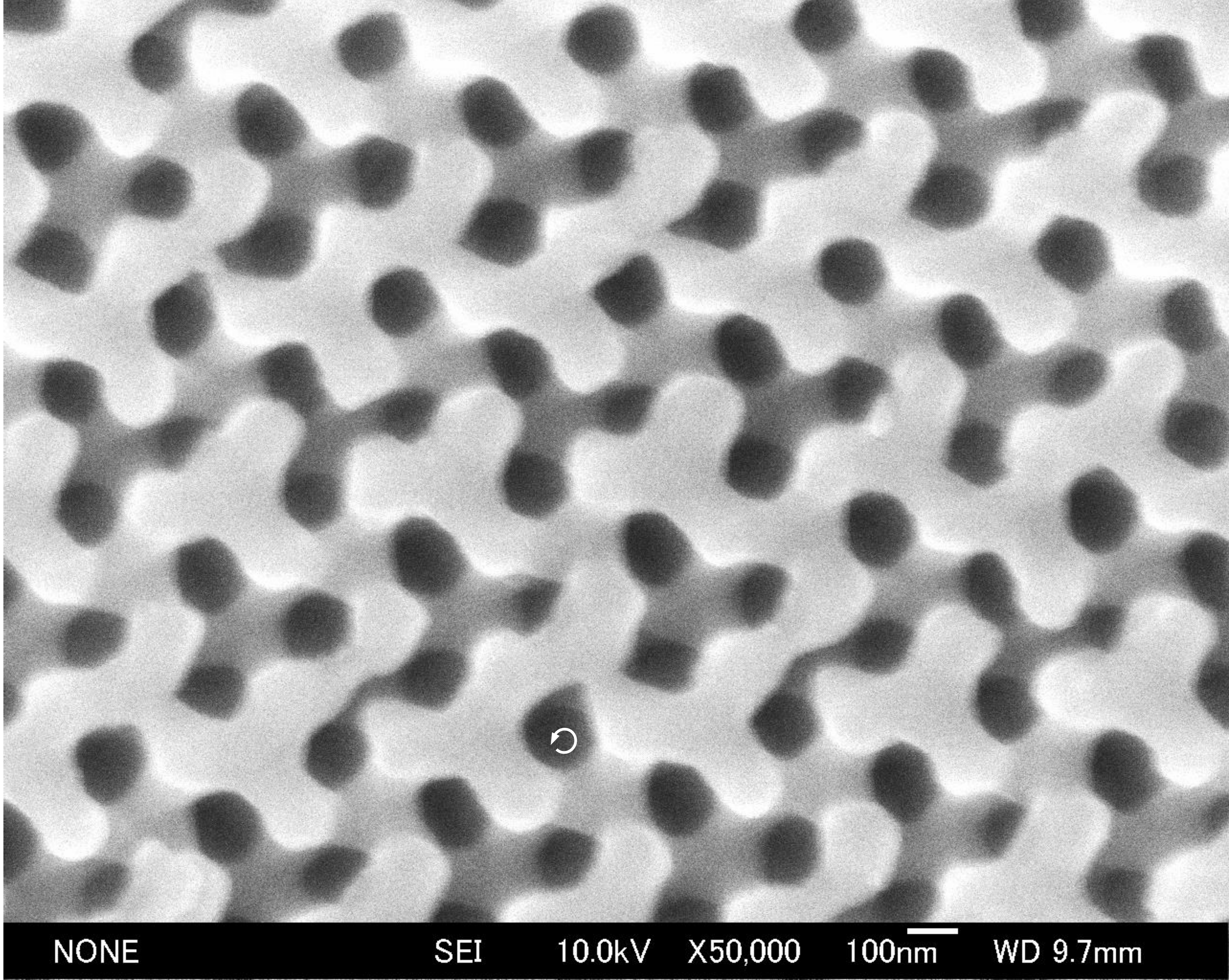

specimen No. 1
scale No. 2
domain No. 5
LH

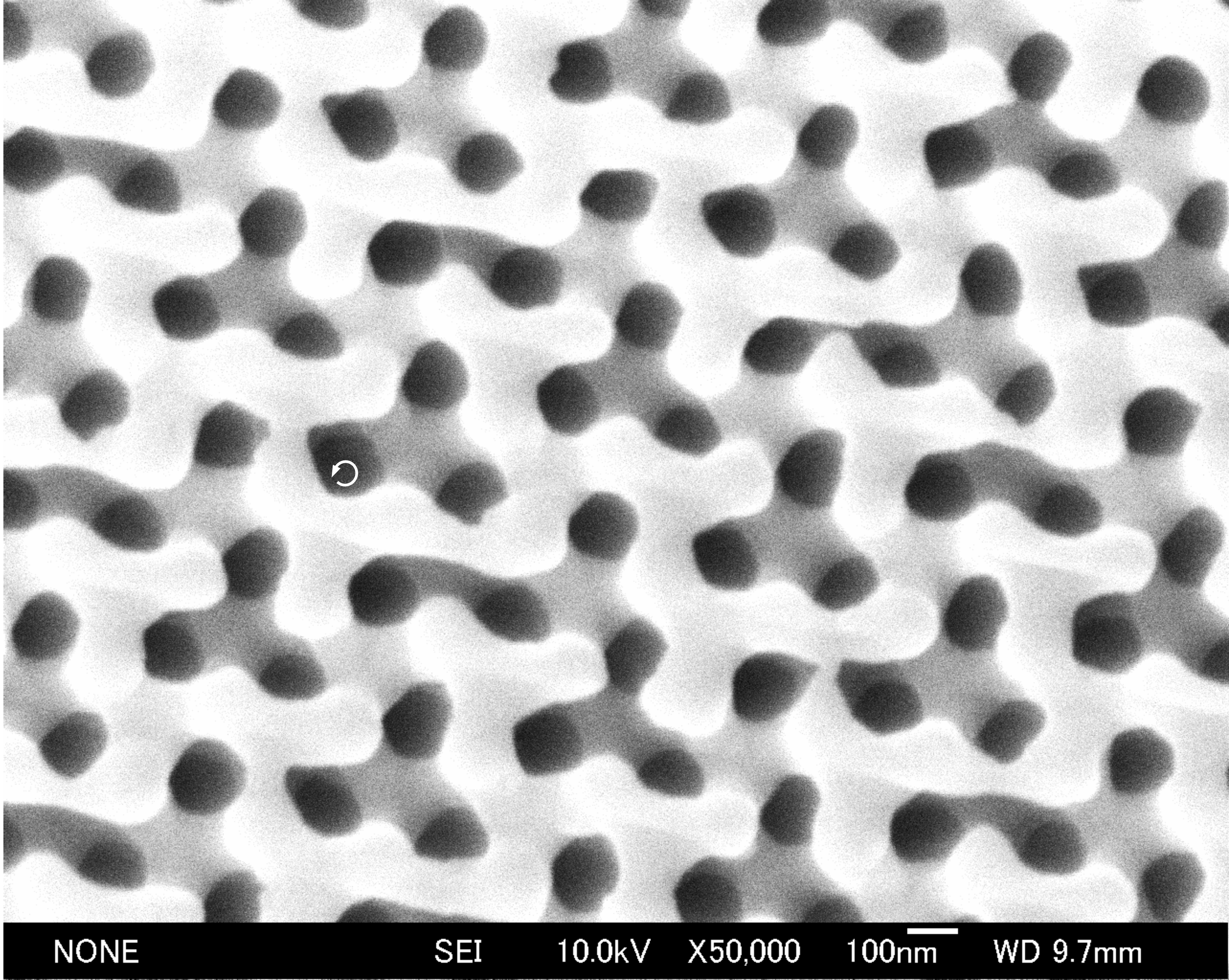

specimen No. 1
scale No. 2
domain No. 6
LH

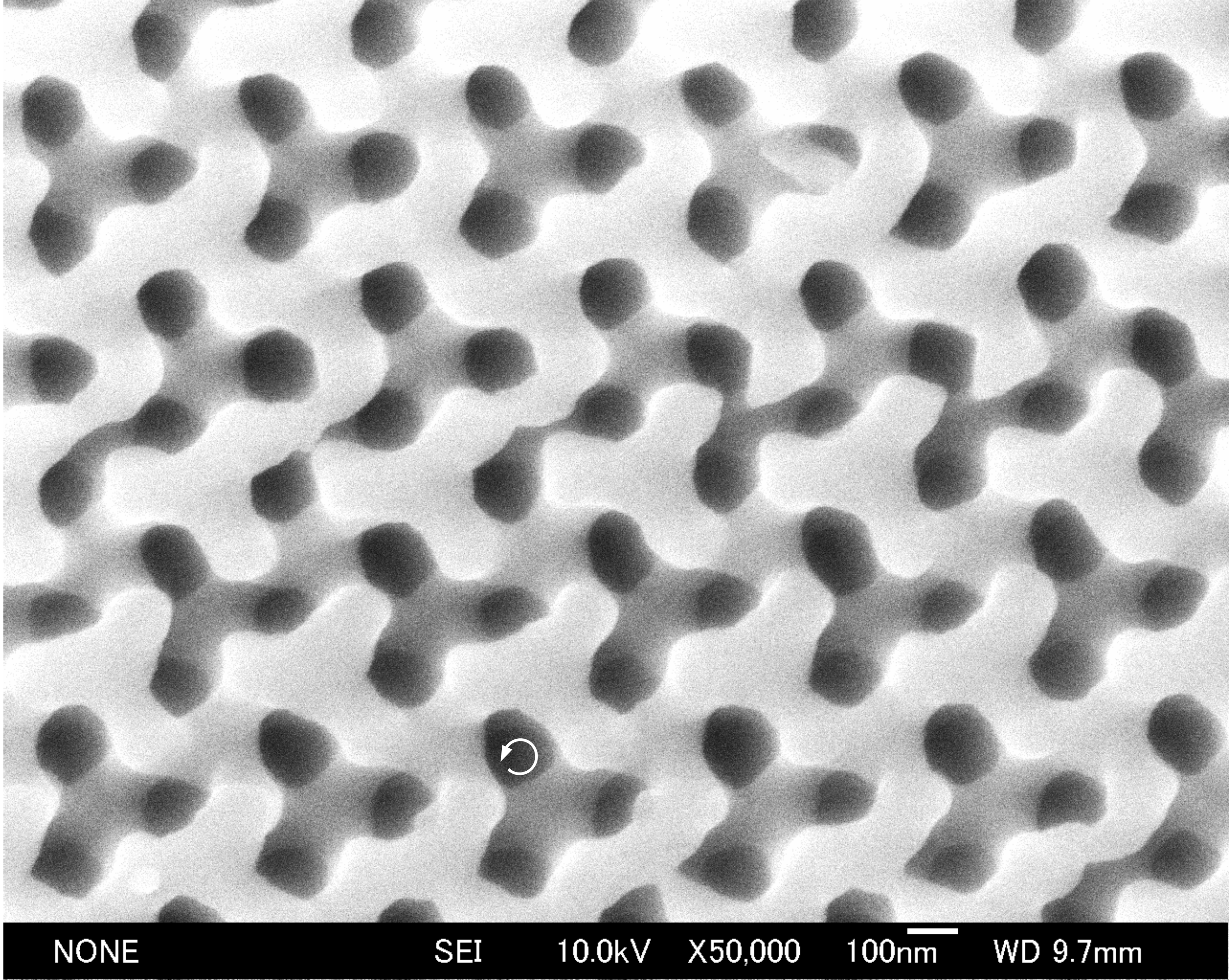

specimen No. 1
scale No. 2
domain No. 7
LH

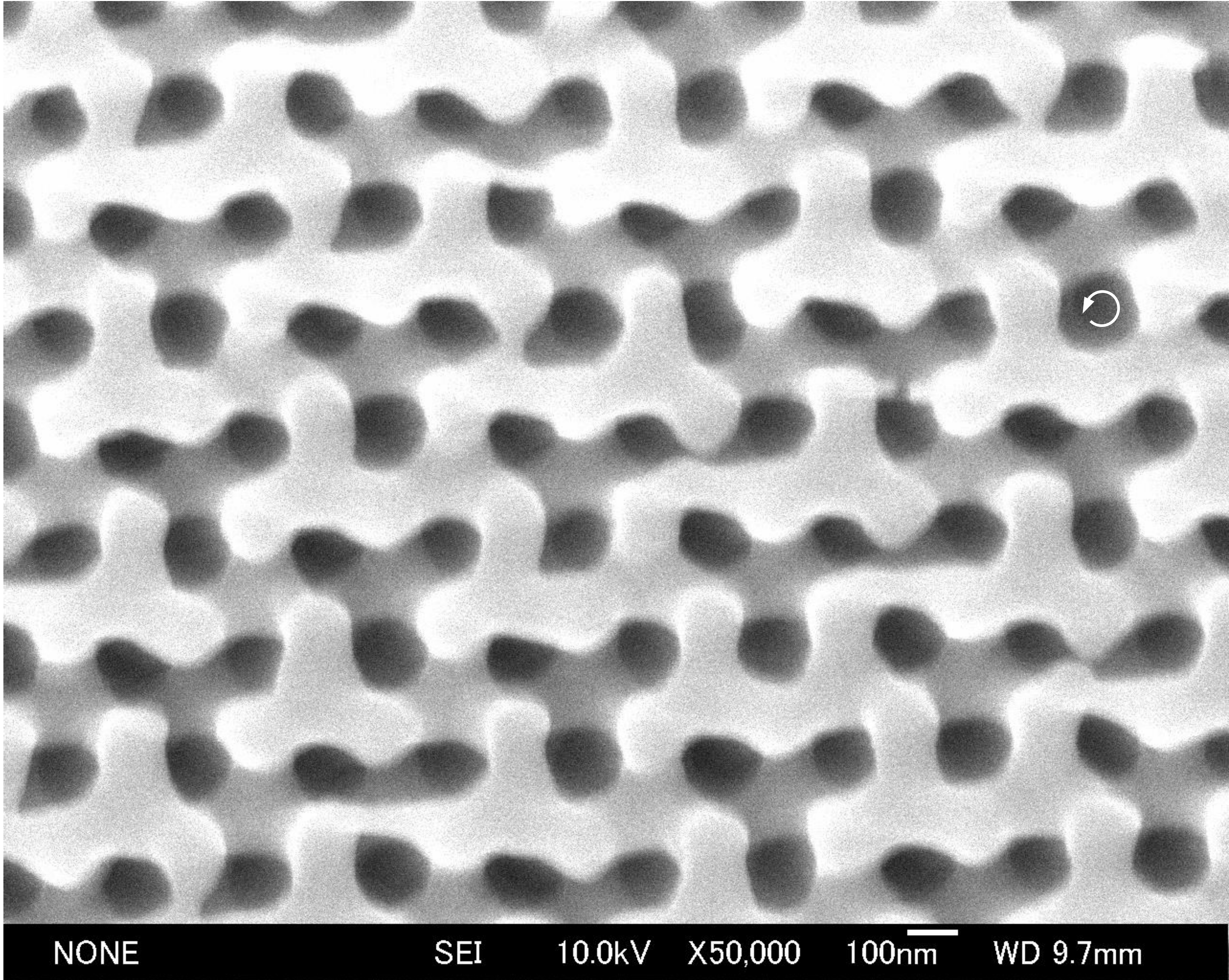

specimen No. 1
scale No. 2
domain No. 8
LH

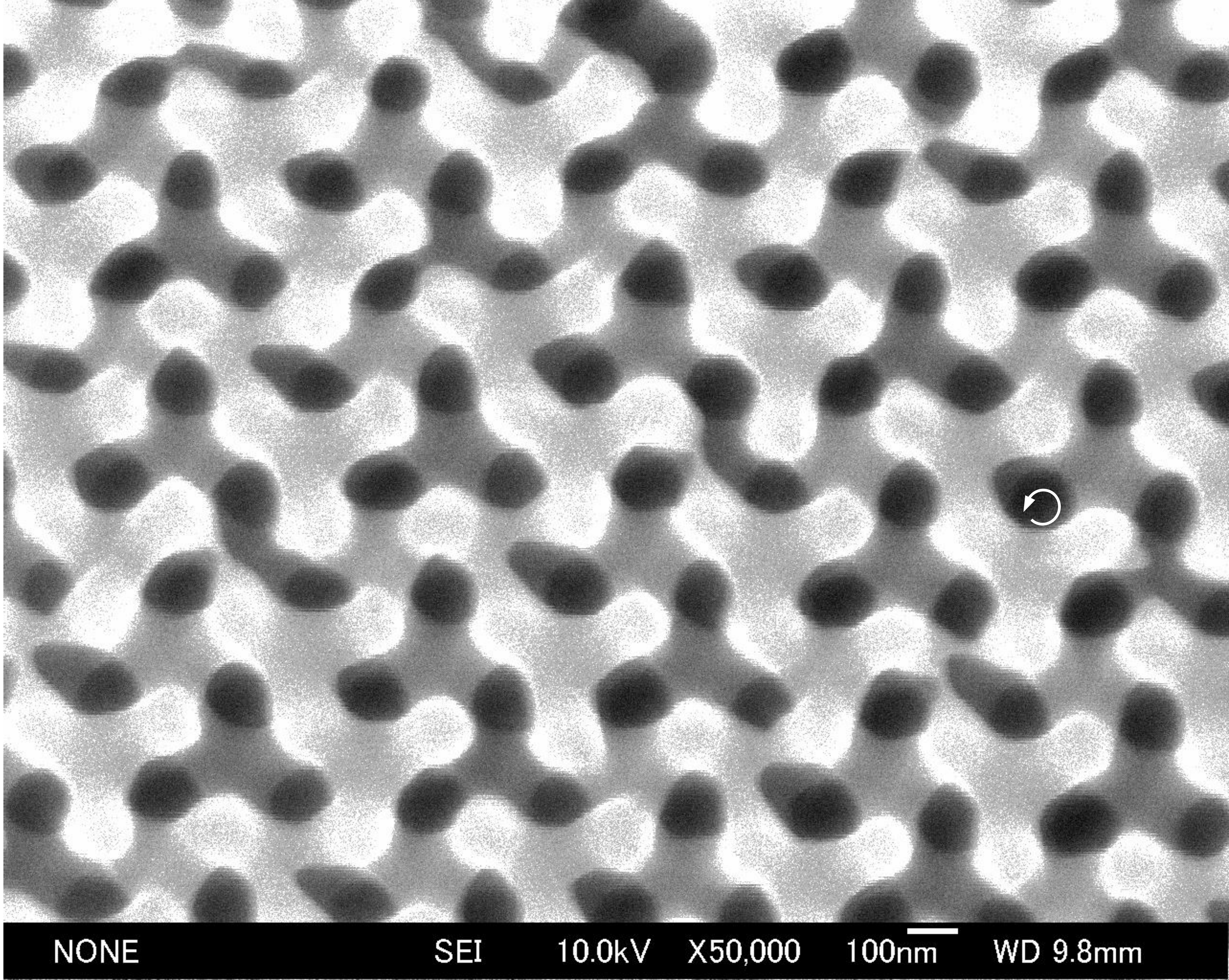

specimen No. 1
scale No. 2
domain No. 9
LH

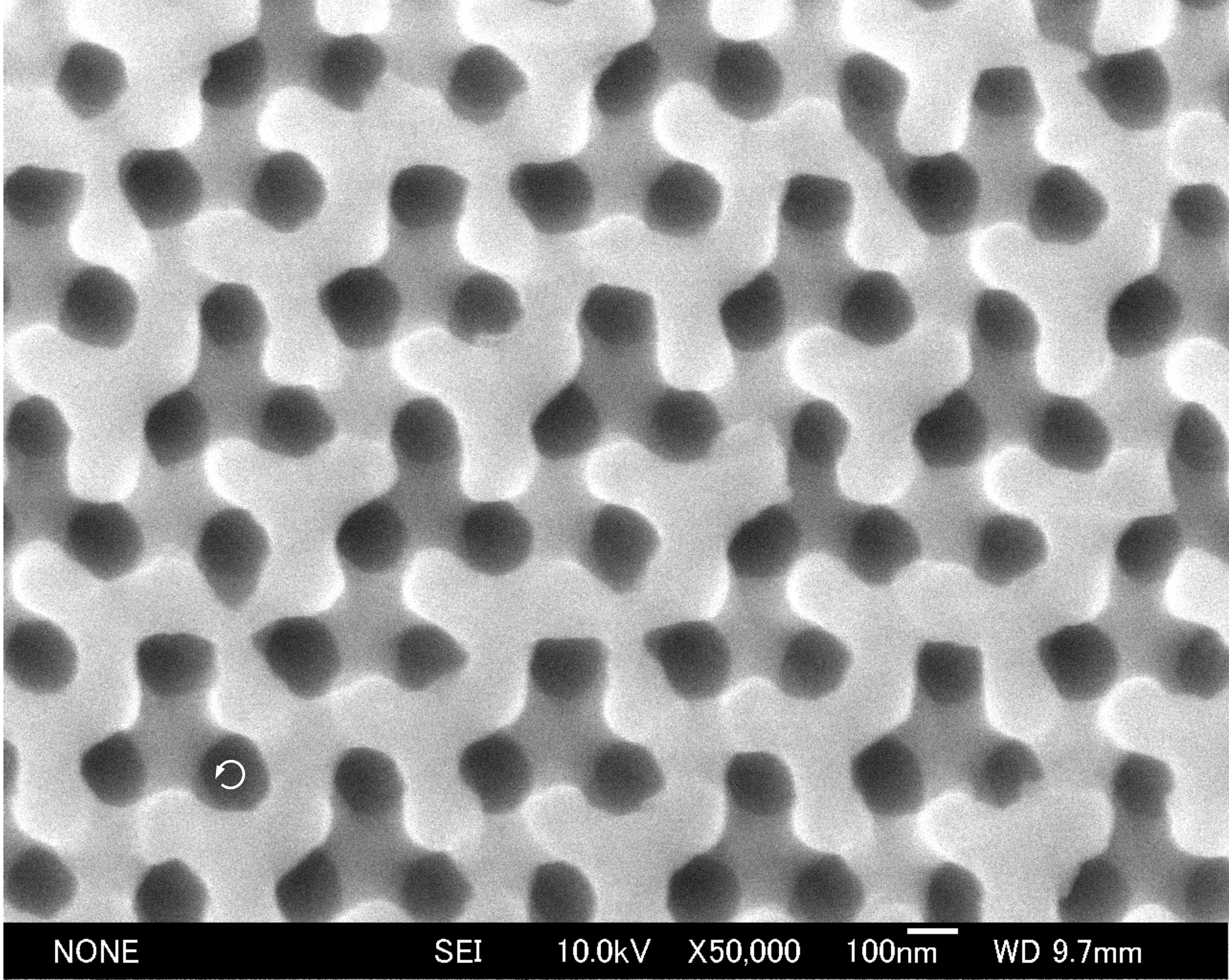

specimen No. 1
scale No. 2
domain No. 10
LH

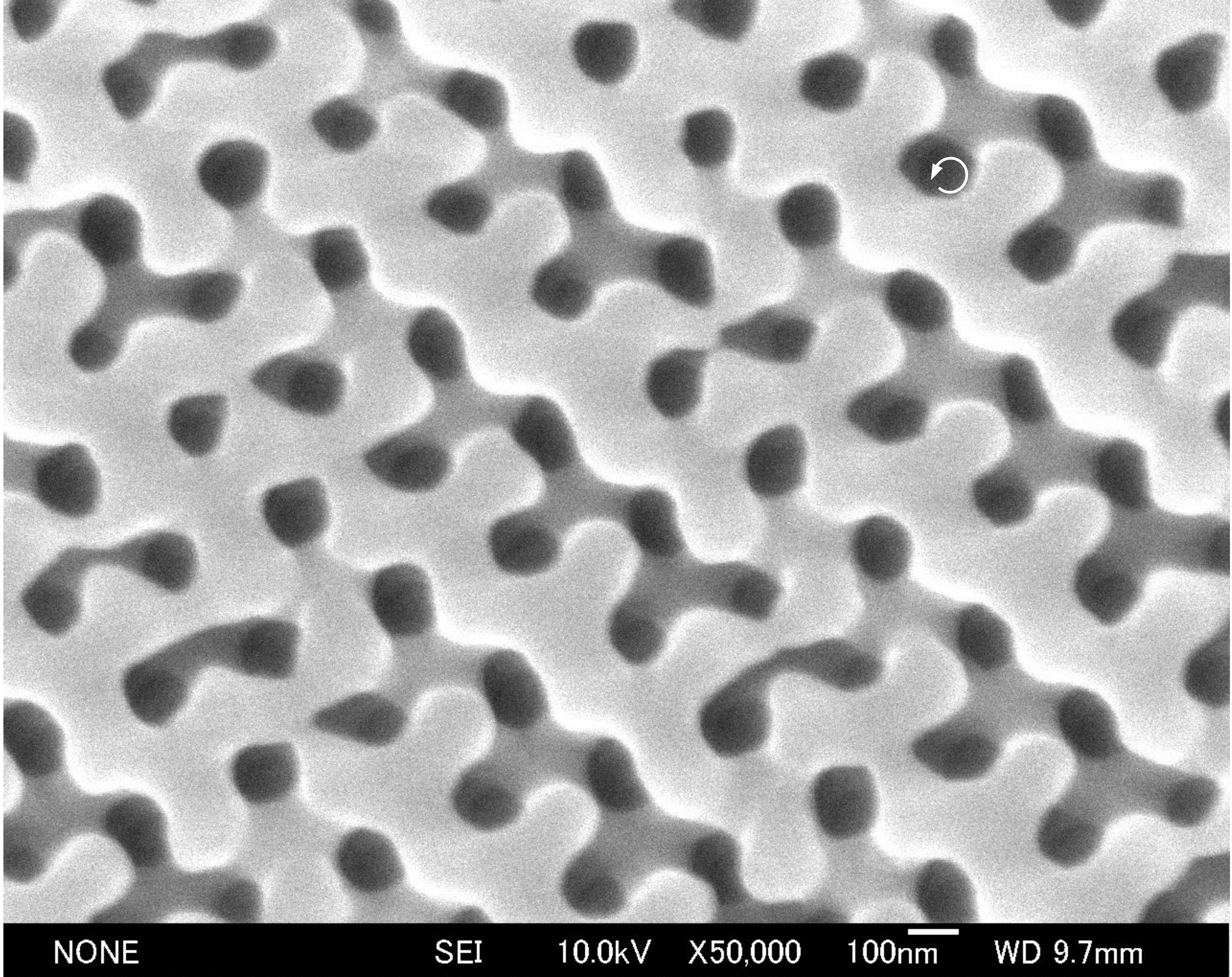

specimen No. 1
scale No. 2
domain No. 11
LH

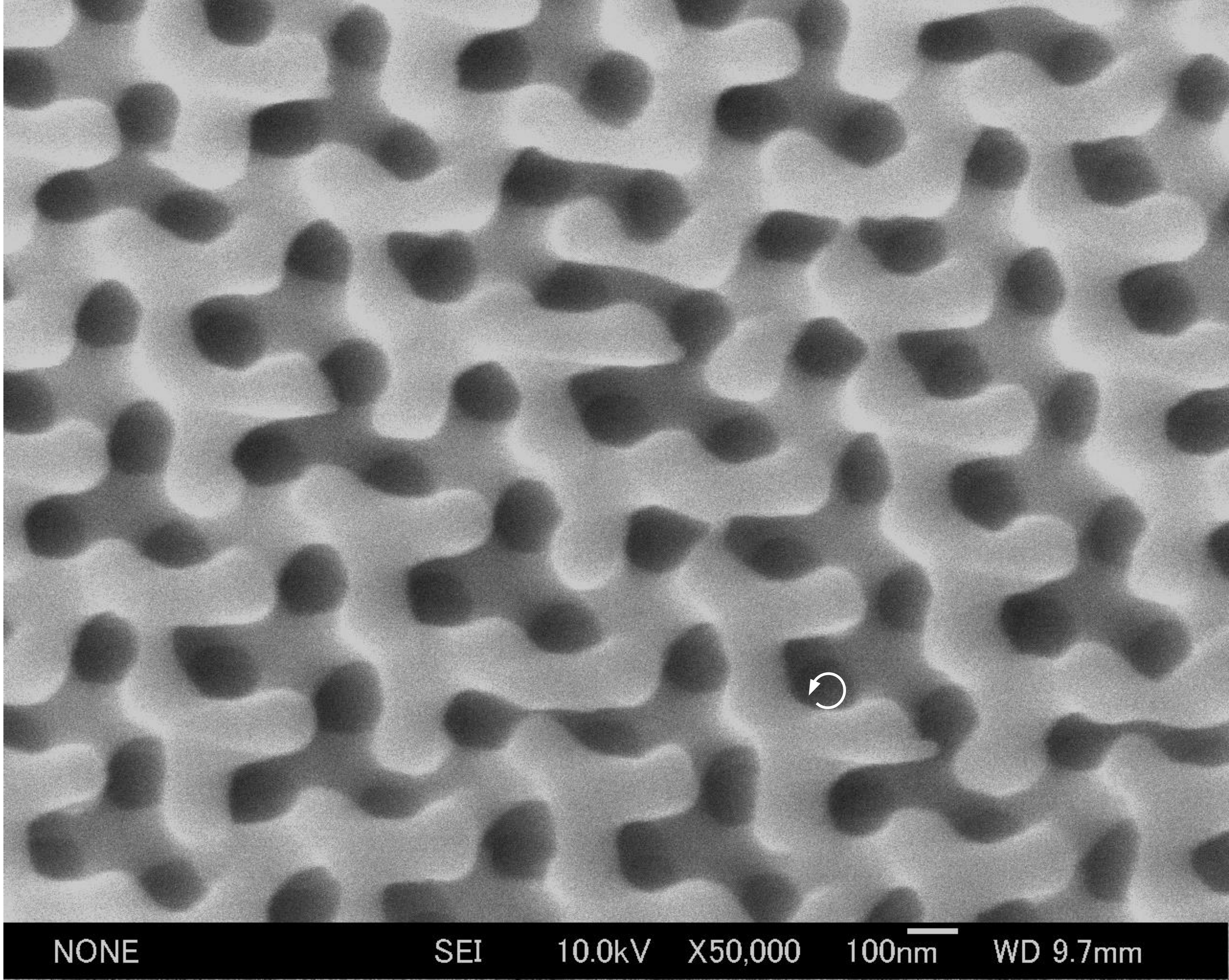

specimen No. 1
scale No. 2
domain No. 12
LH

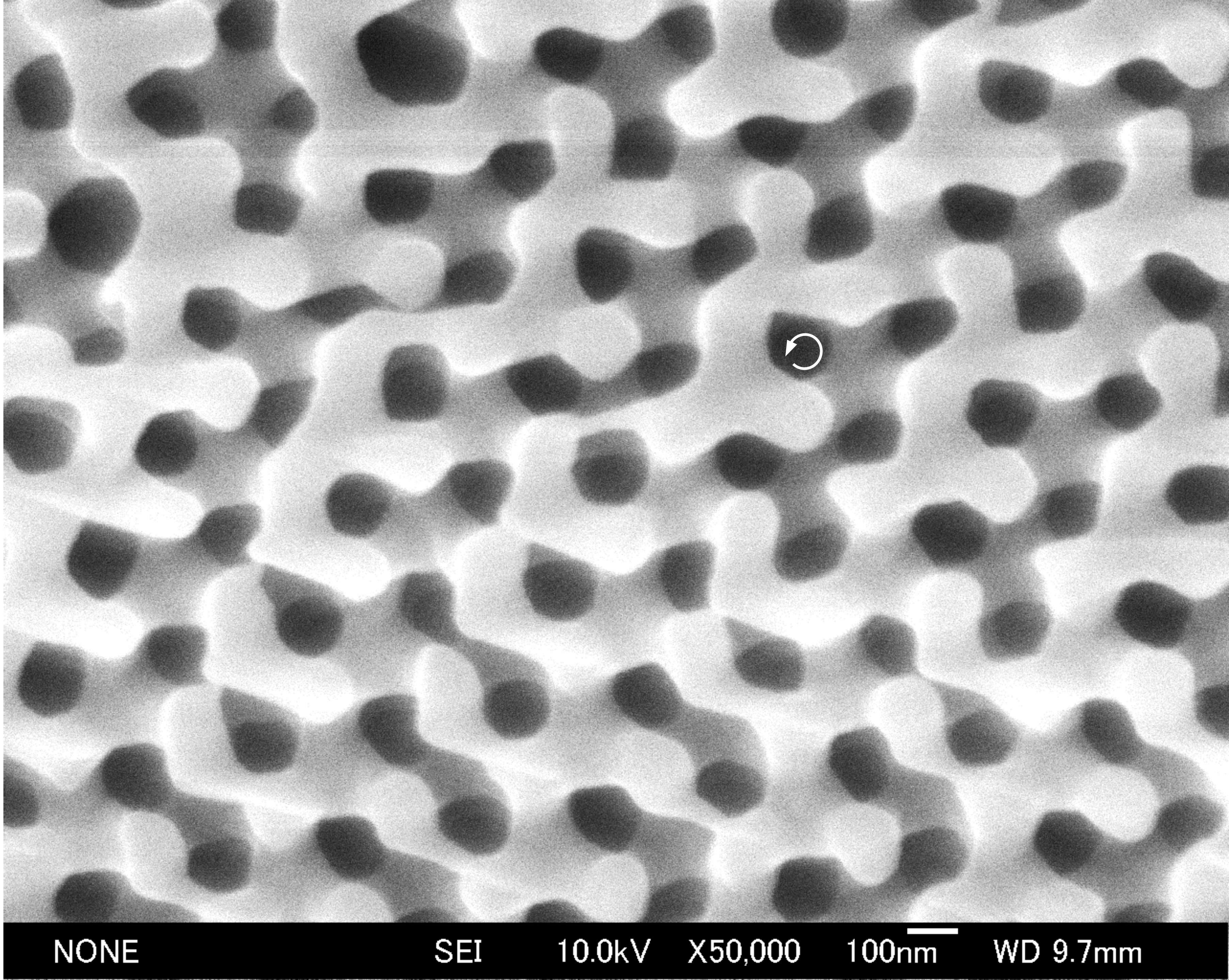

specimen No. 1
scale No. 2
domain No. 13
LH

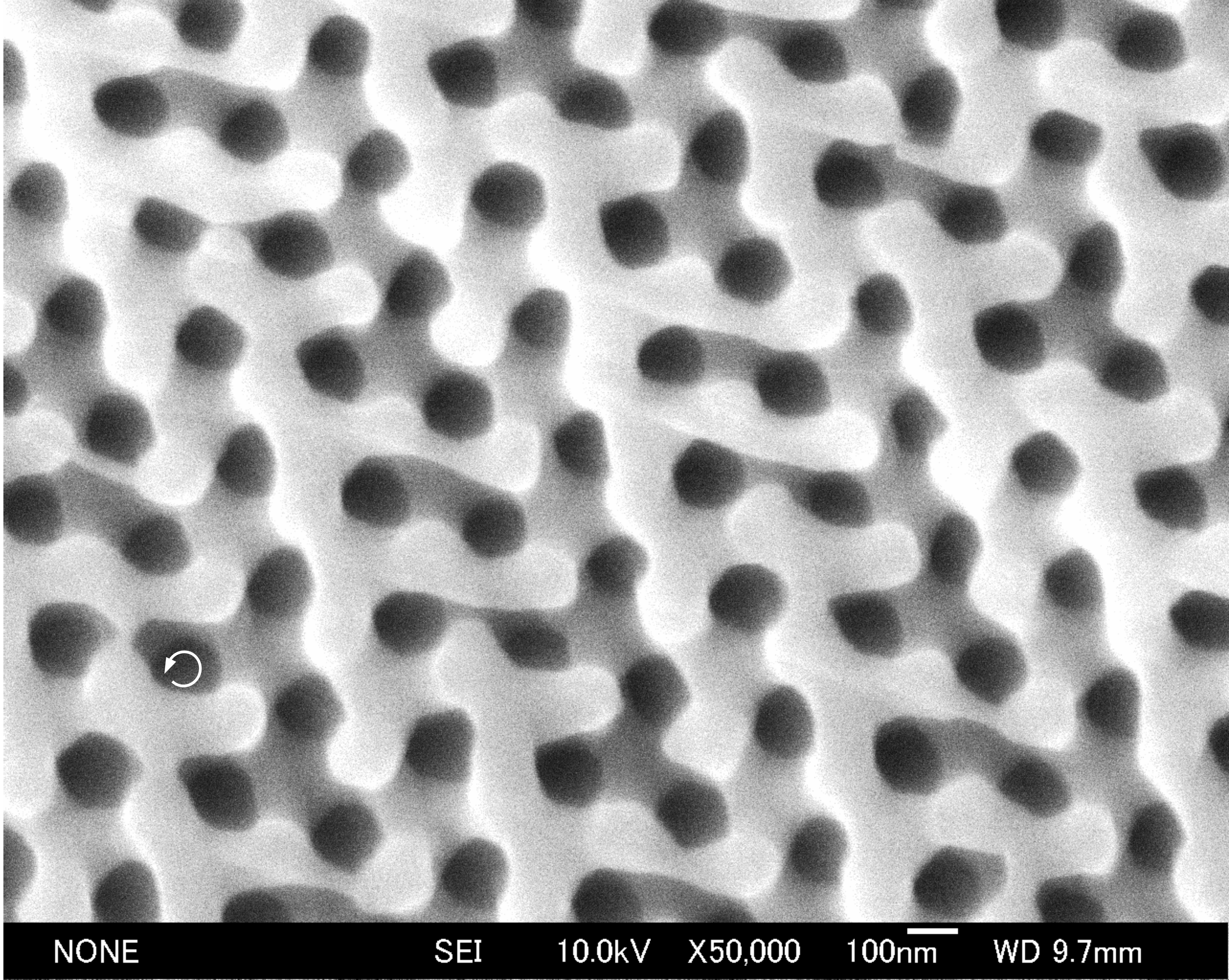

specimen No. 1
scale No. 2
domain No. 14
LH

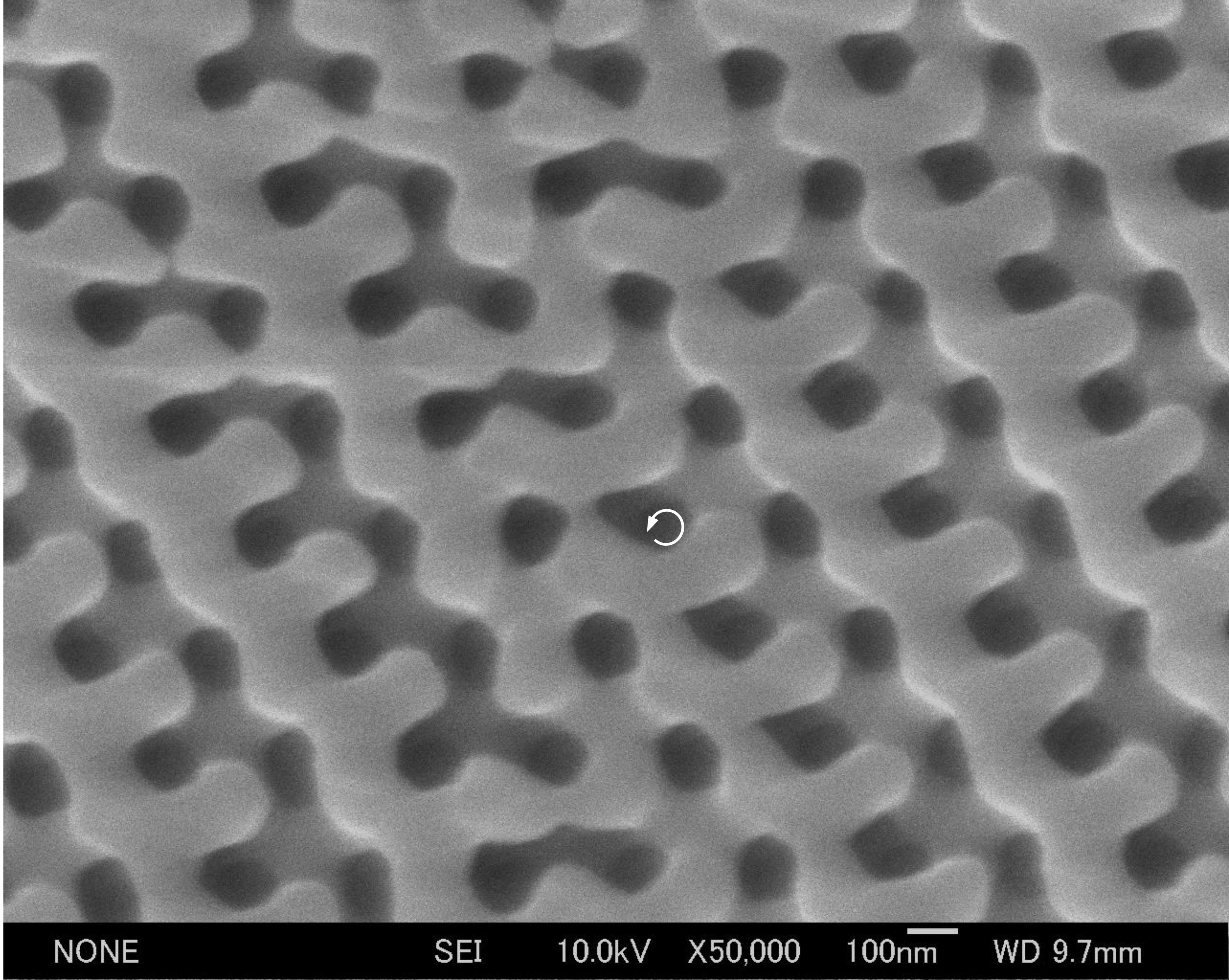

specimen No. 1
scale No. 2
domain No. 15
LH

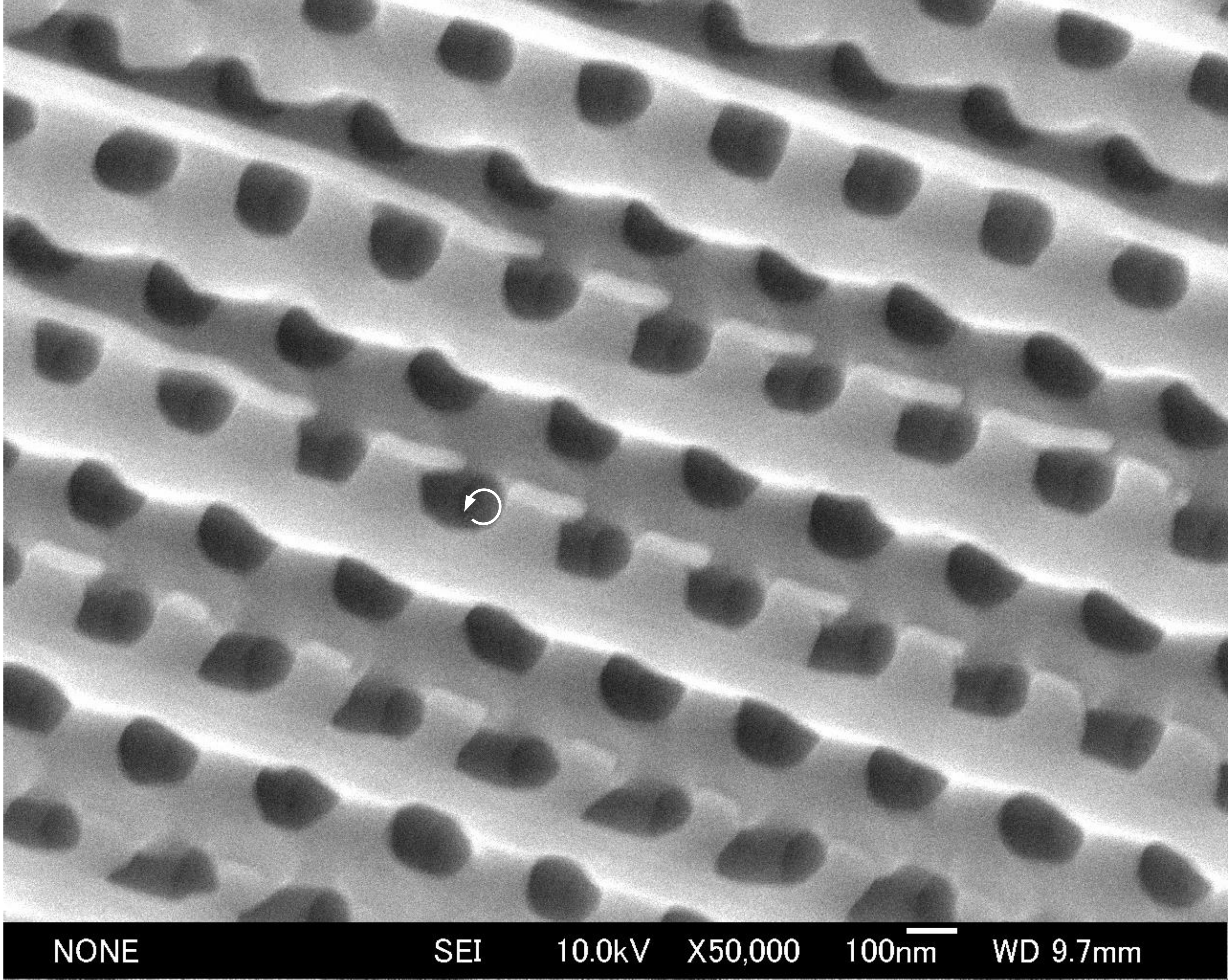

specimen No. 1
scale No. 2
domain No. 16
LH

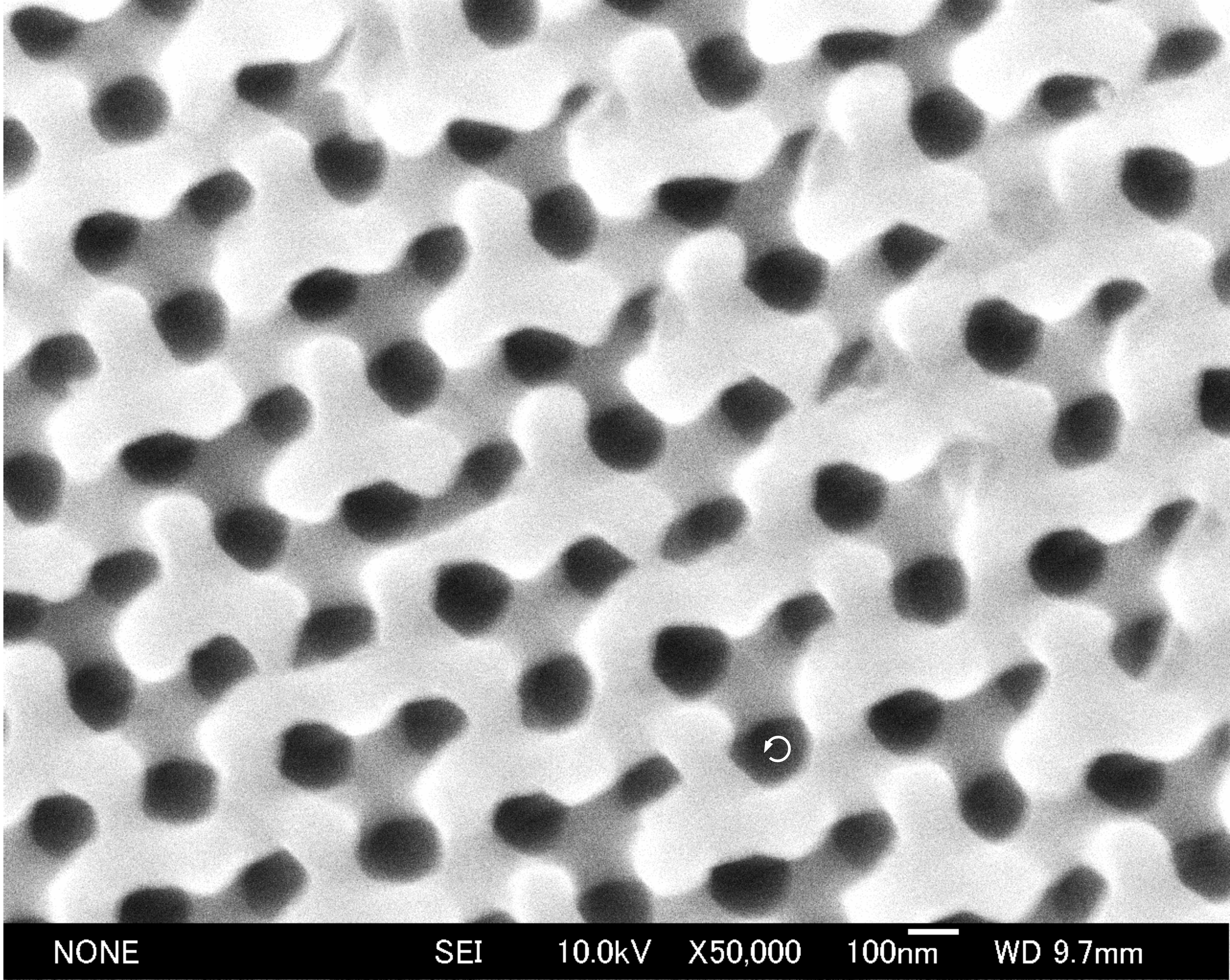

specimen No. 1
scale No. 2
domain No. 17
LH

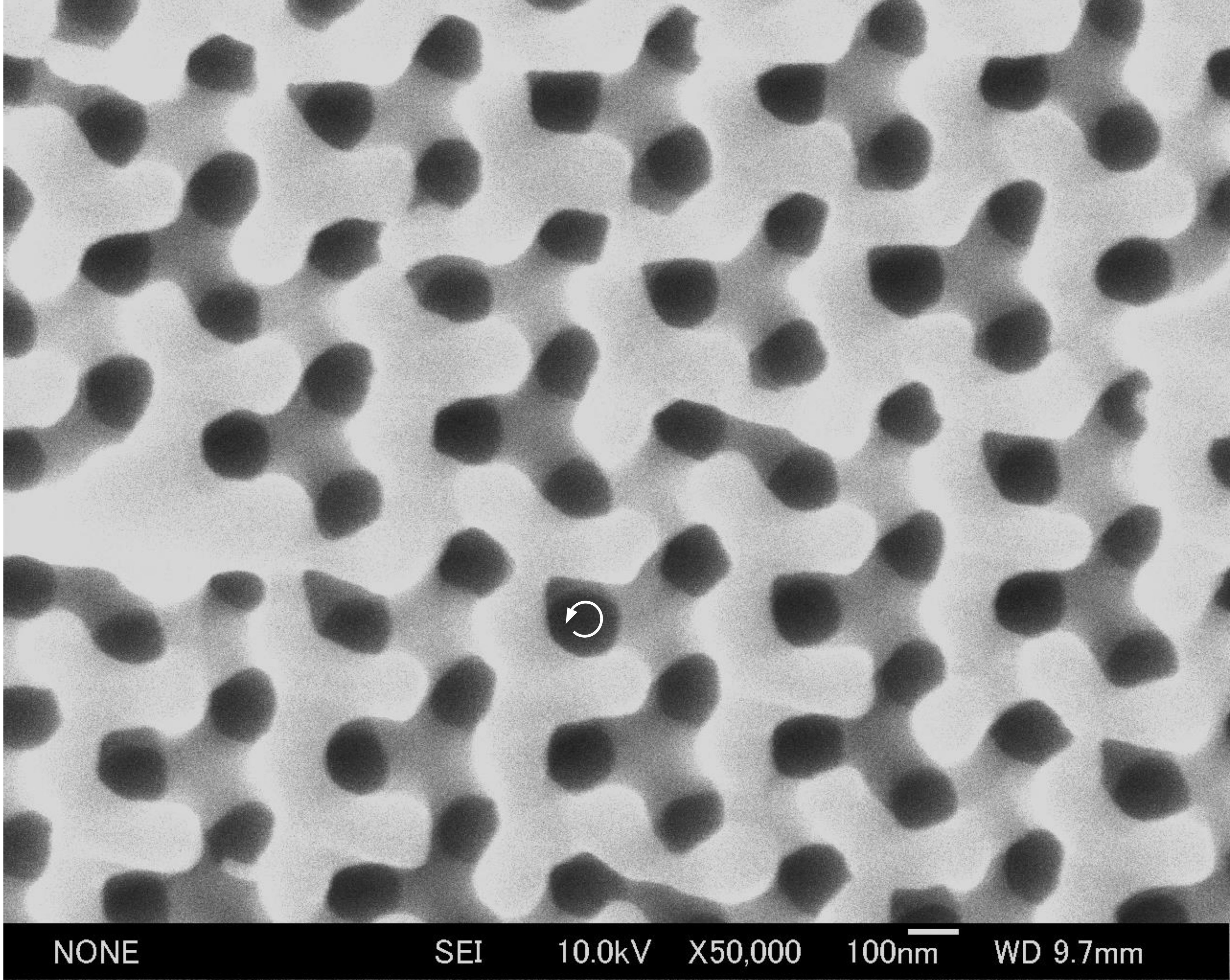

specimen No. 1
scale No. 3

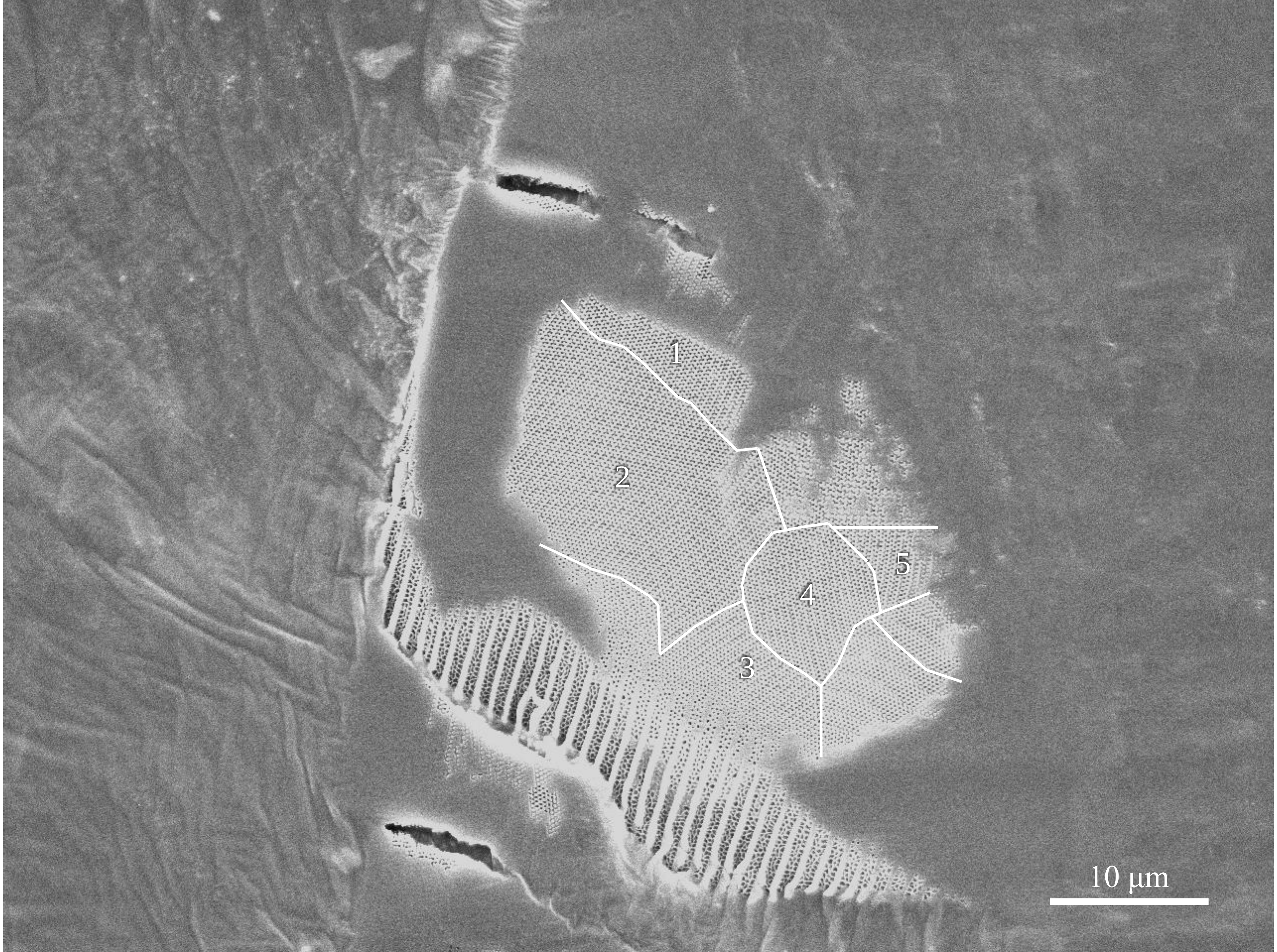

specimen No. 1
scale No. 3
domain No. 1
LH

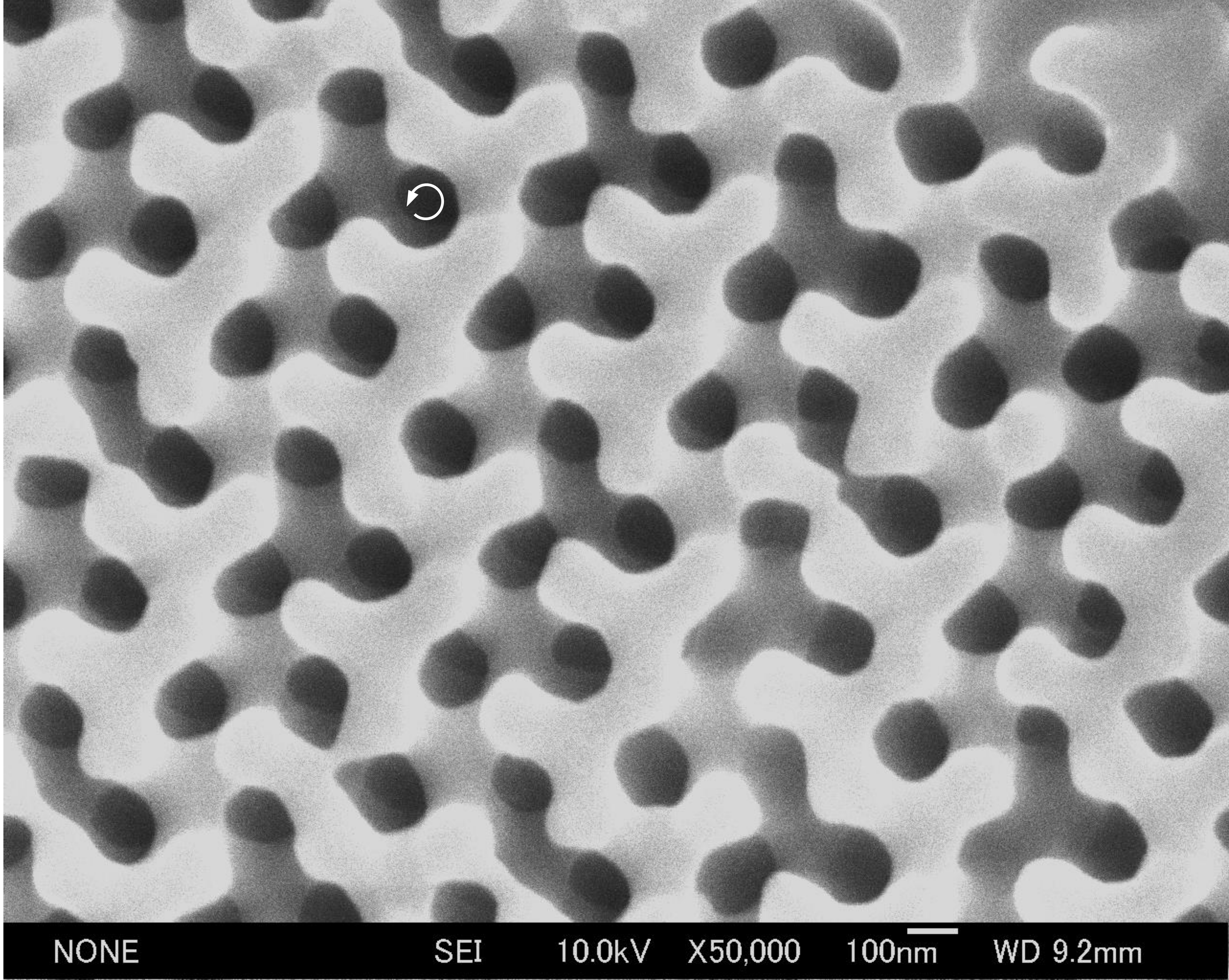

specimen No. 1
scale No. 3
domain No. 2
LH

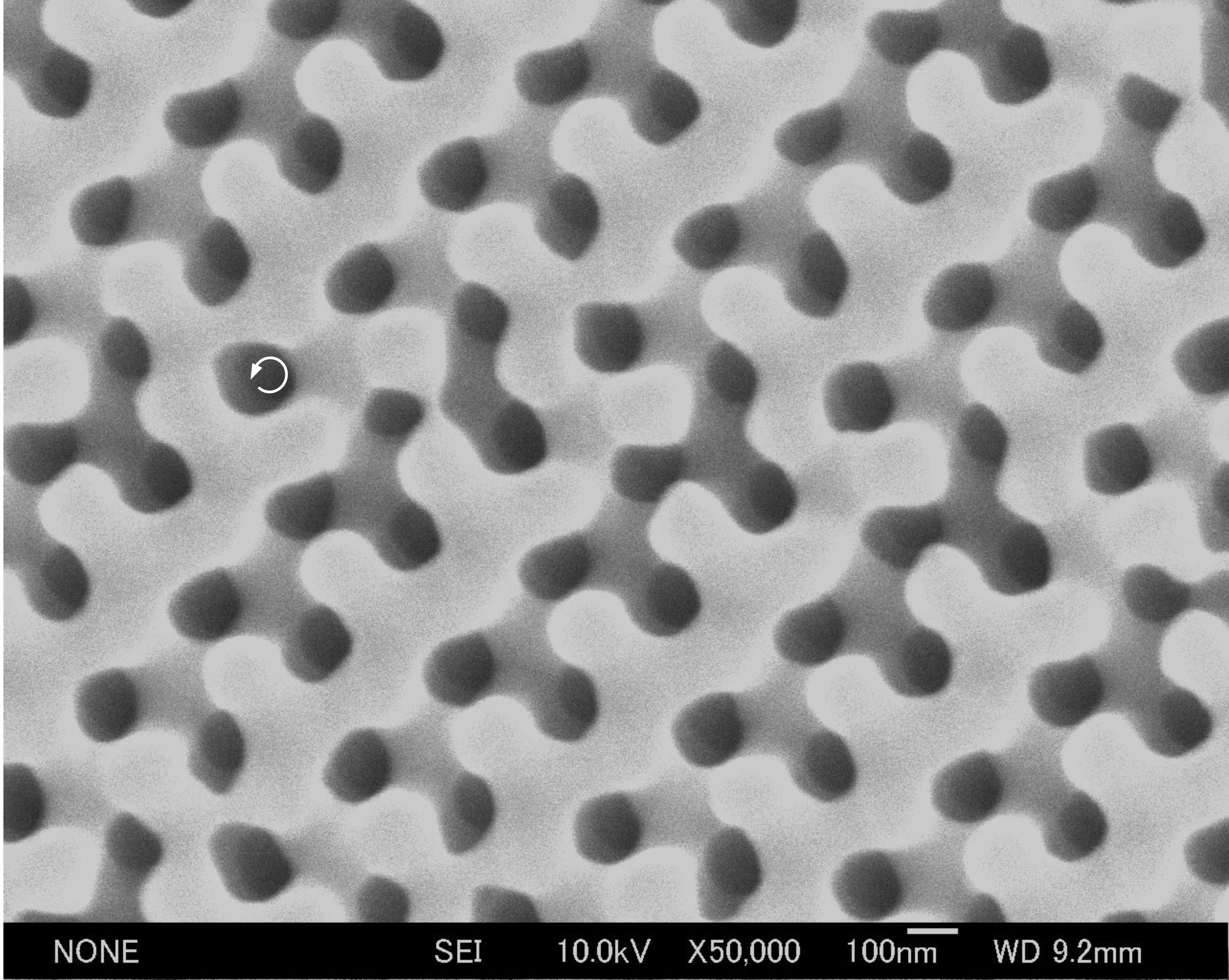

specimen No. 1
scale No. 3
domain No. 3
LH

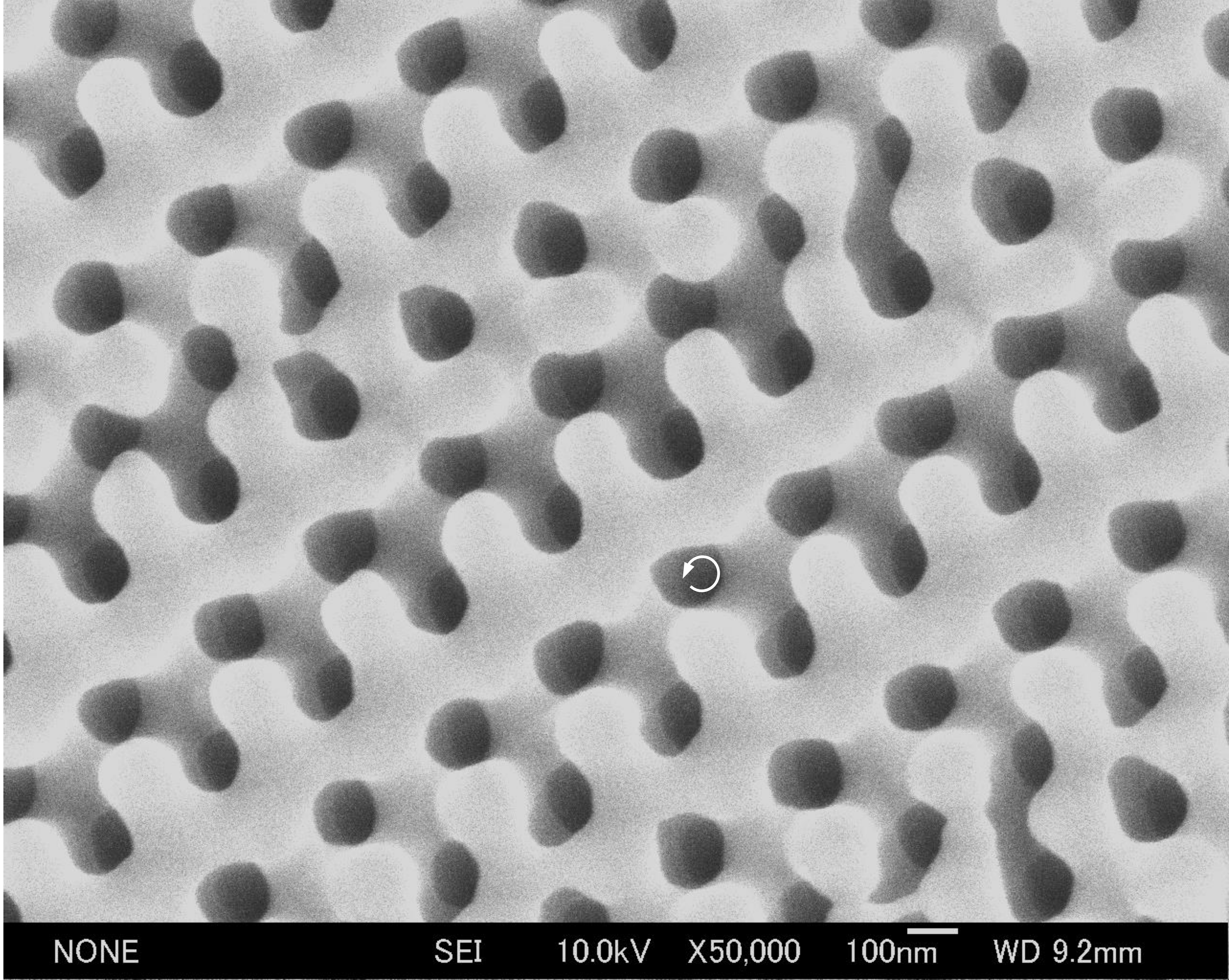

specimen No. 1
scale No. 3
domain No. 4
LH

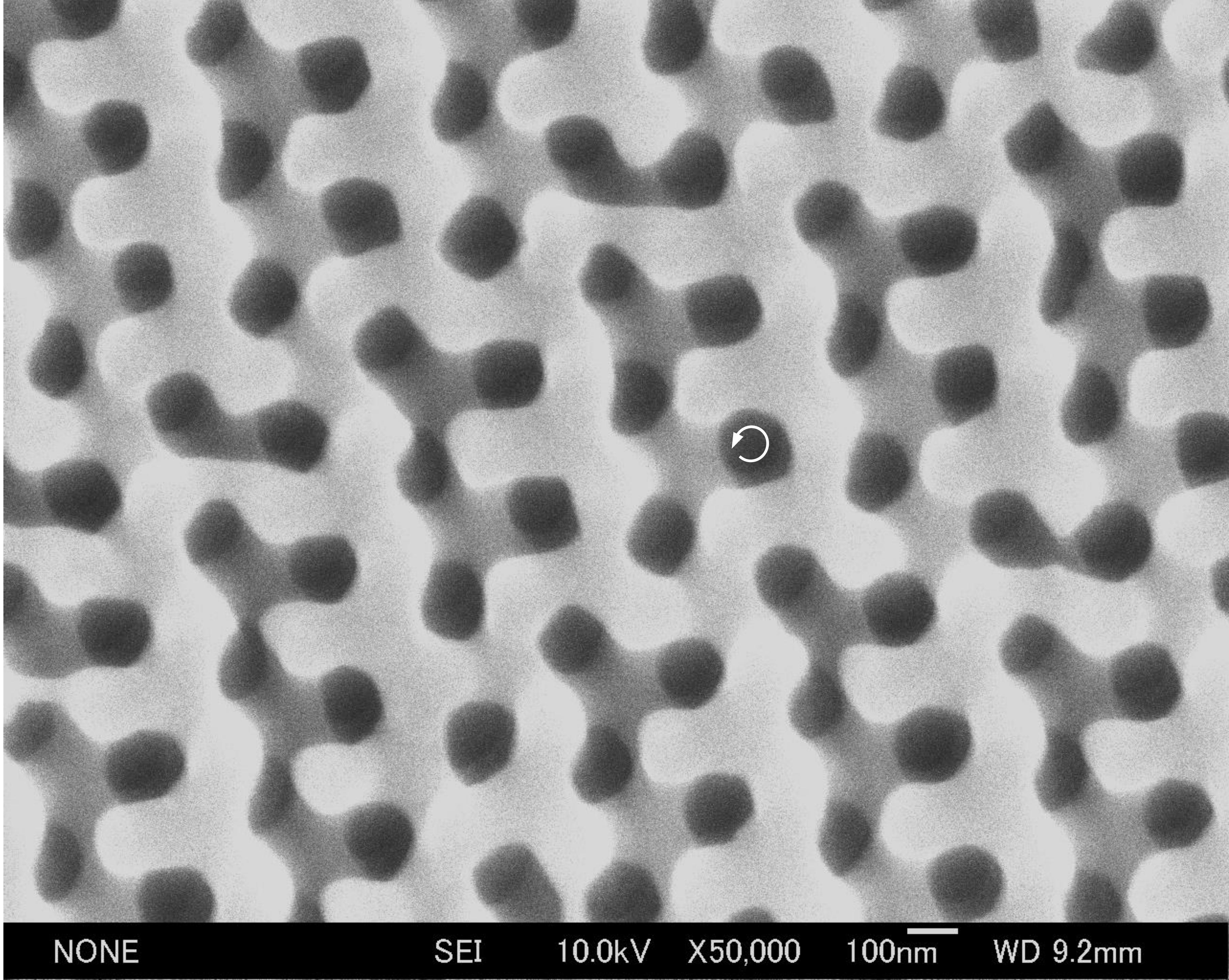

specimen No. 1
scale No. 3
domain No. 5
LH

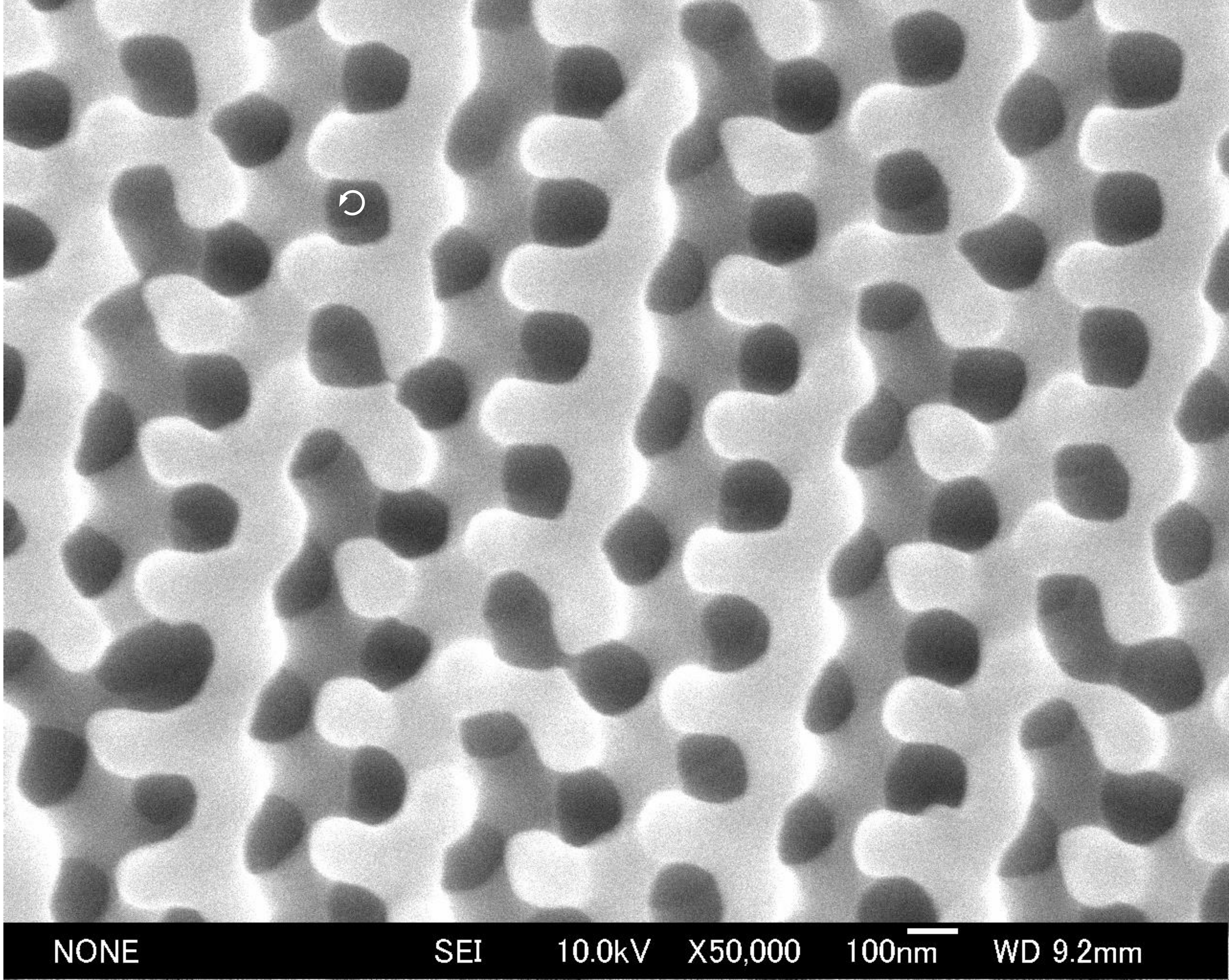

specimen No. 1
scale No. 4

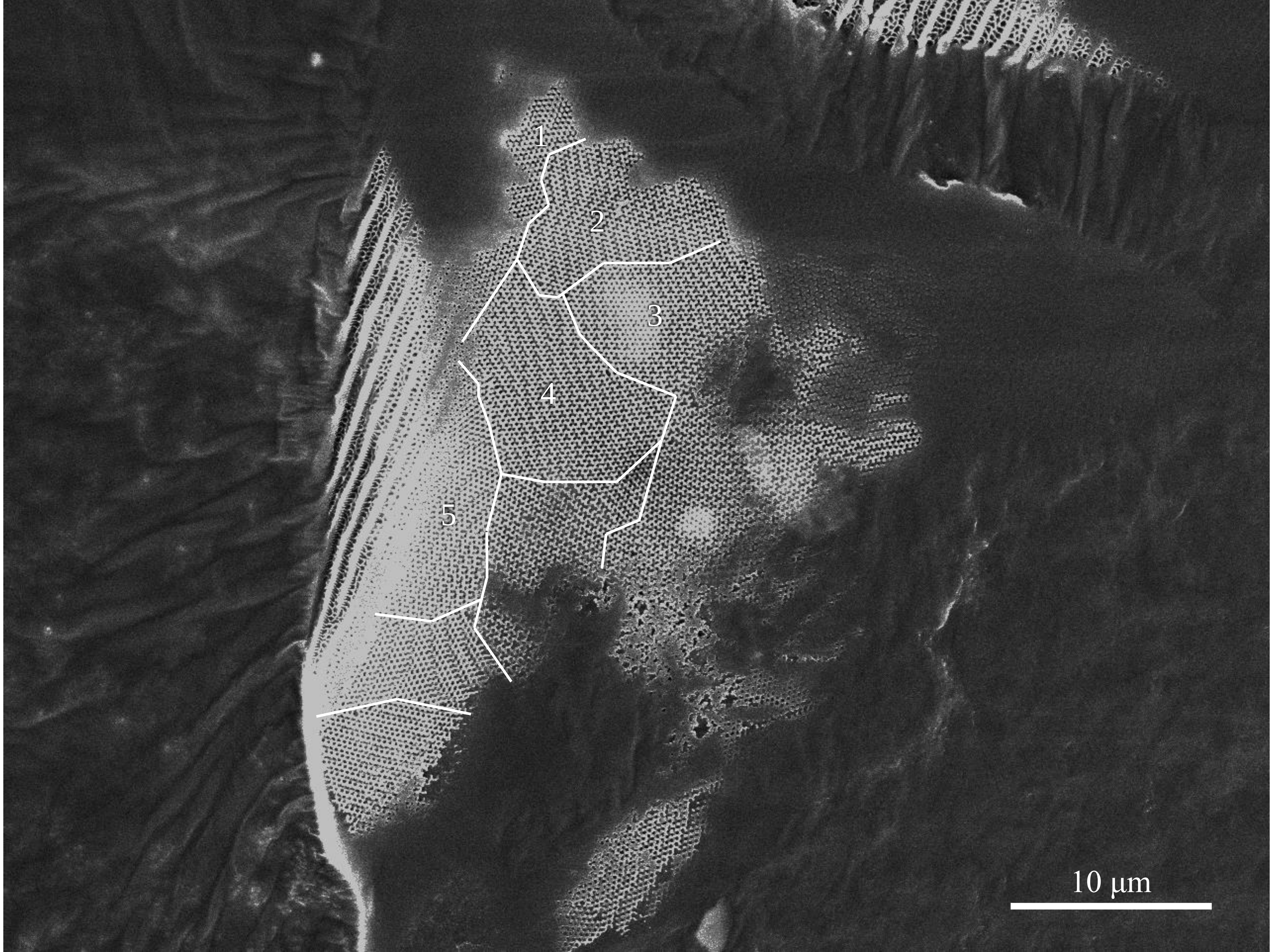

specimen No. 1
scale No. 4
domain No. 1
LH

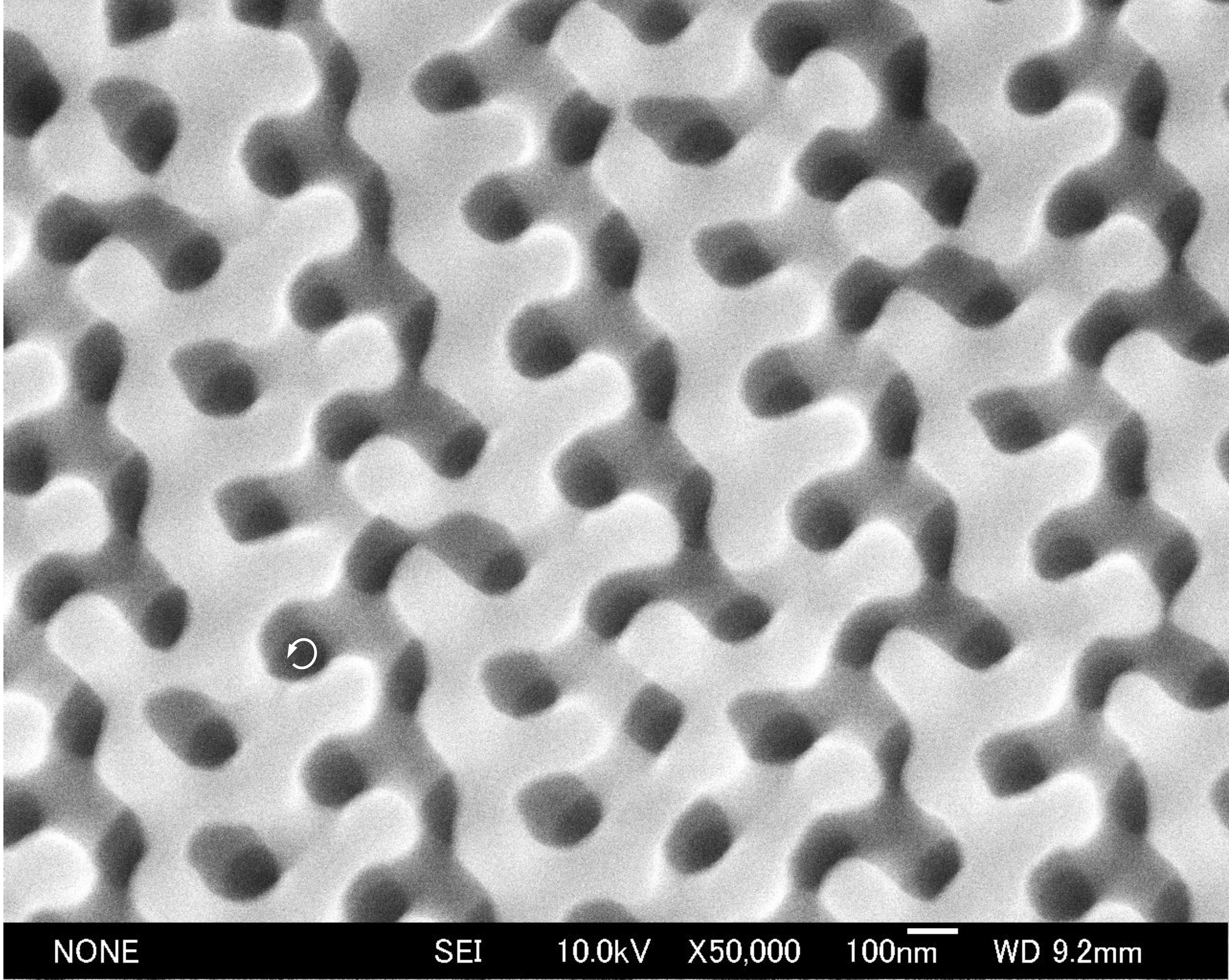

specimen No. 1
scale No. 4
domain No. 2
LH

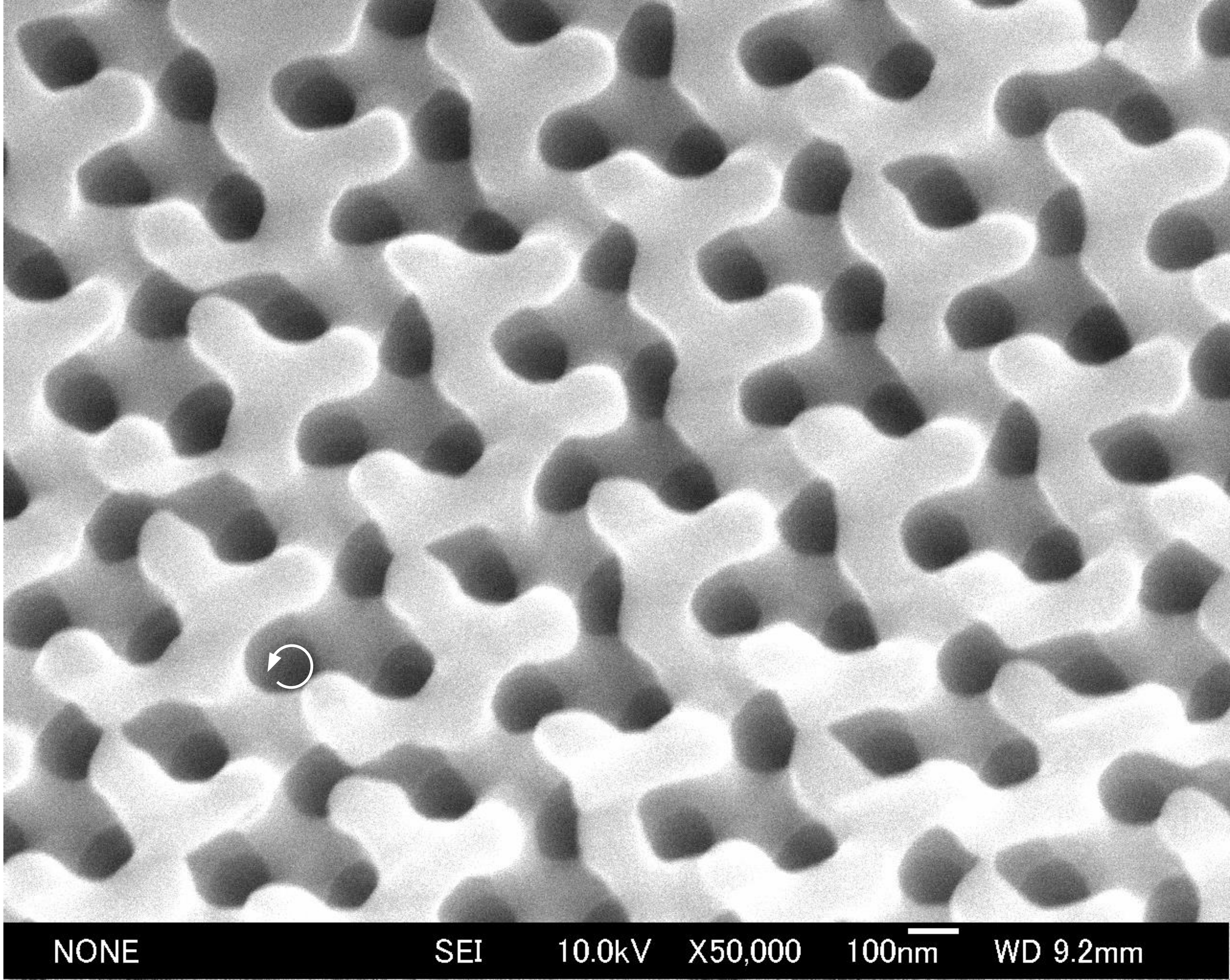

specimen No. 1
scale No. 4
domain No. 3
LH

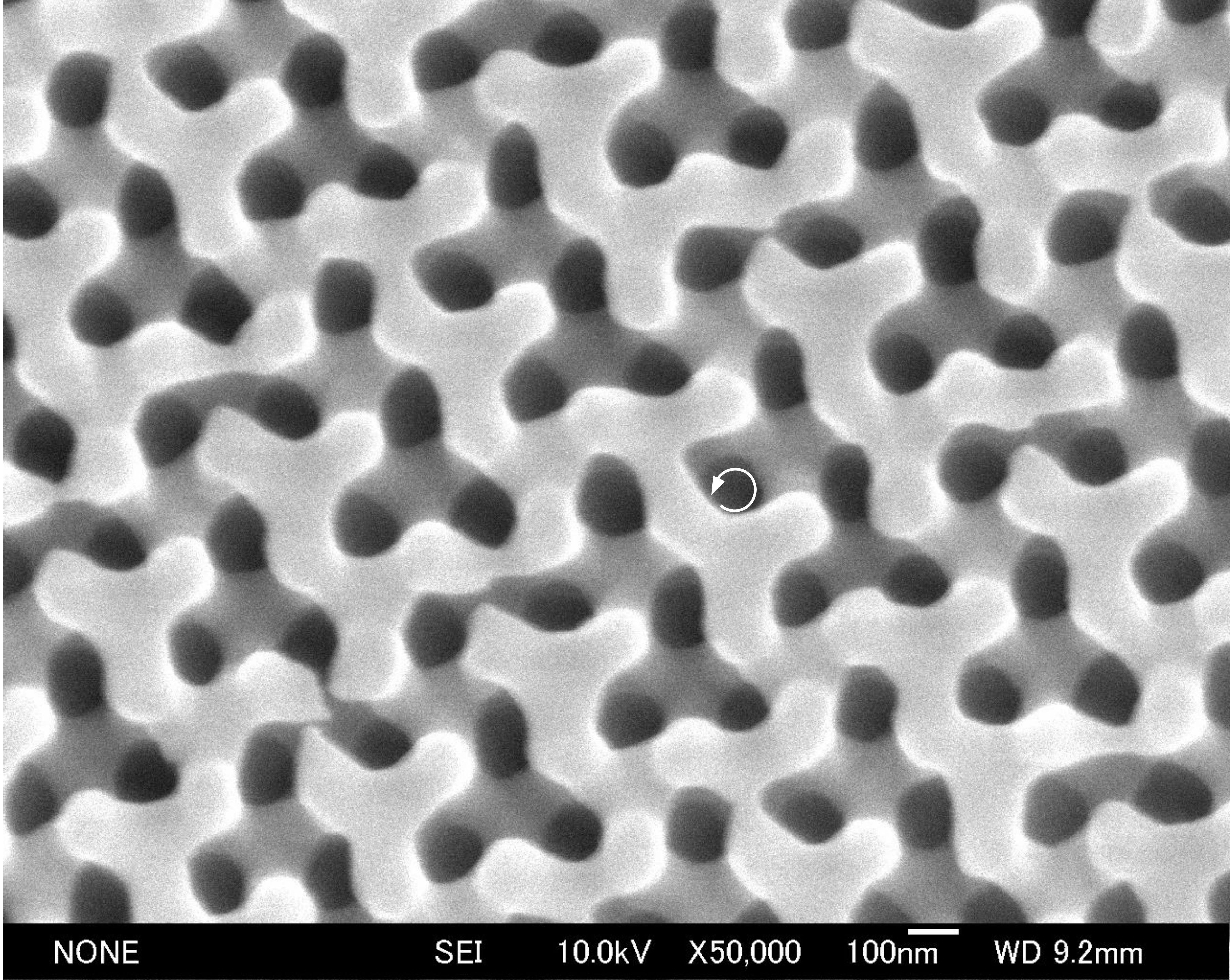

specimen No. 1
scale No. 4
domain No. 4
LH

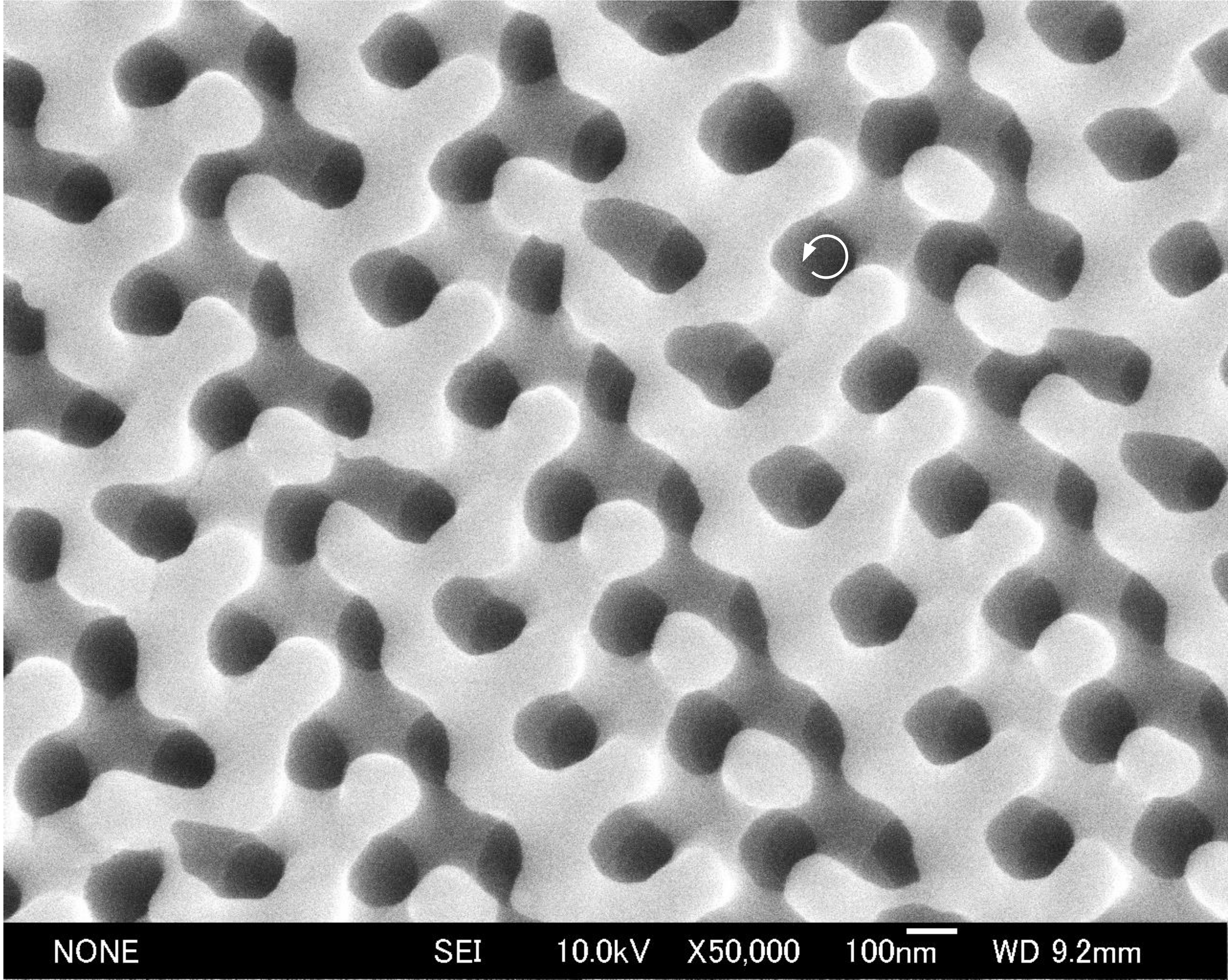

specimen No. 1
scale No. 4
domain No. 5
LH

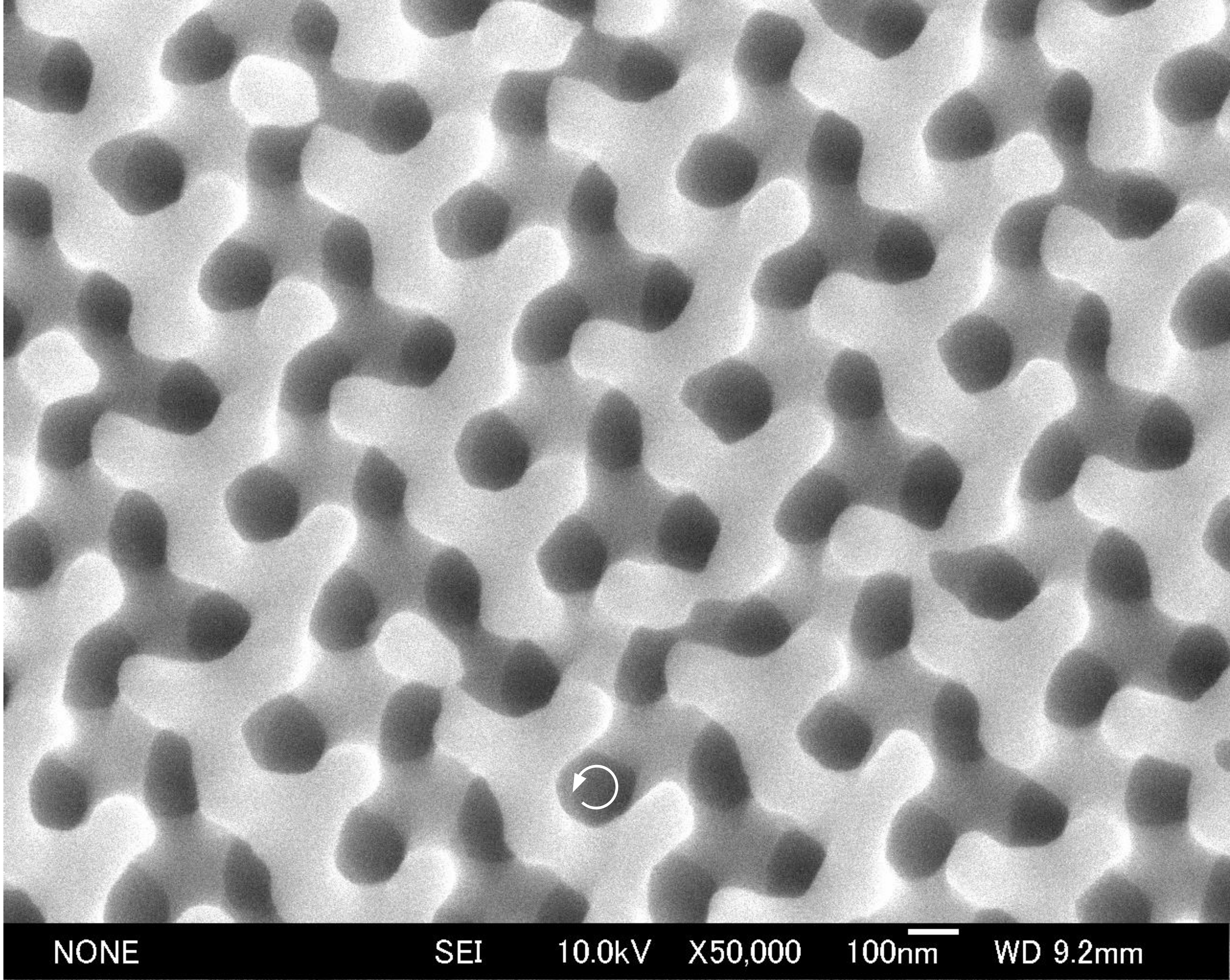

specimen No. 1
scale No. 5

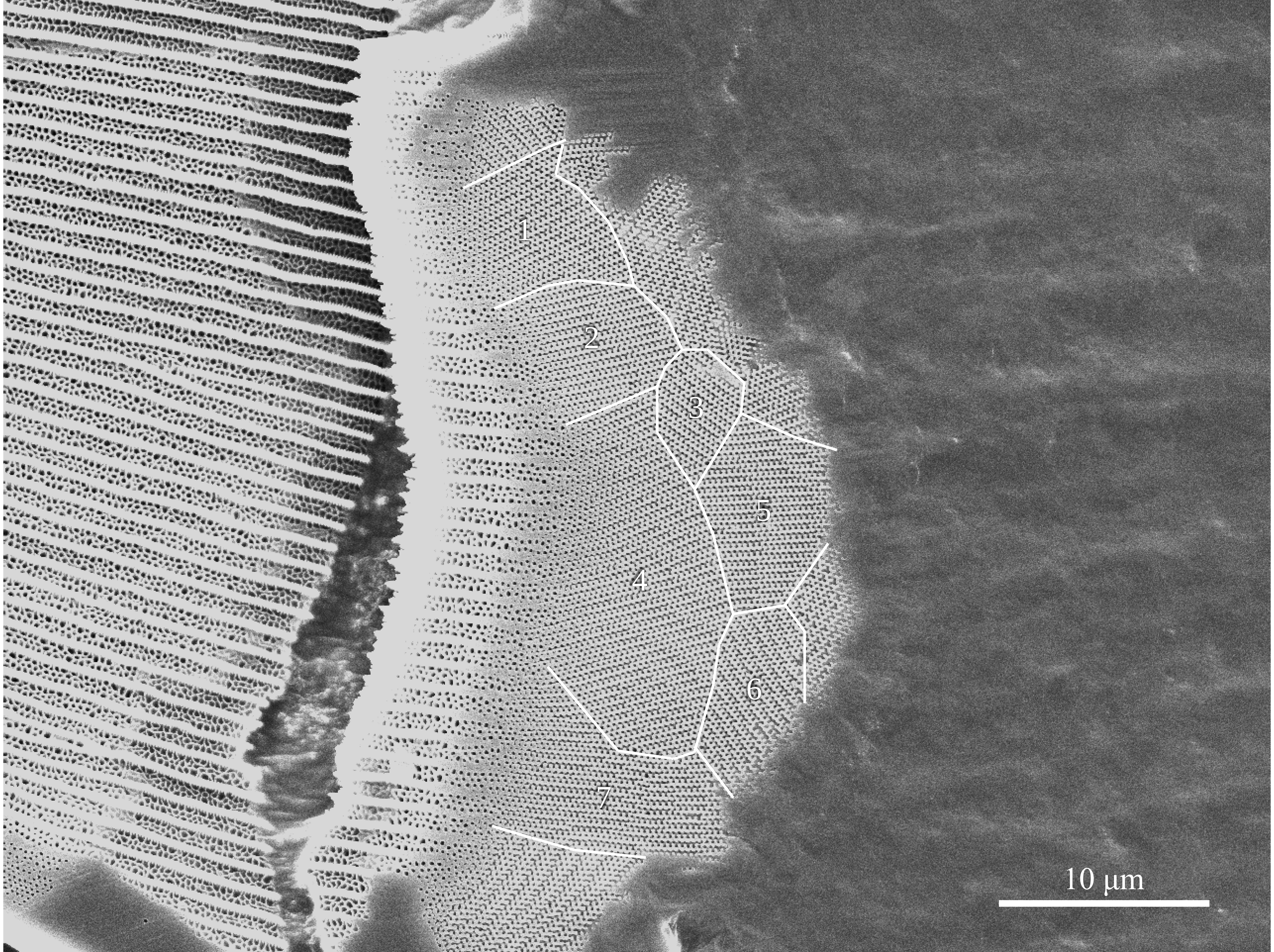

specimen No. 1
scale No. 5
domain No. 1
LH

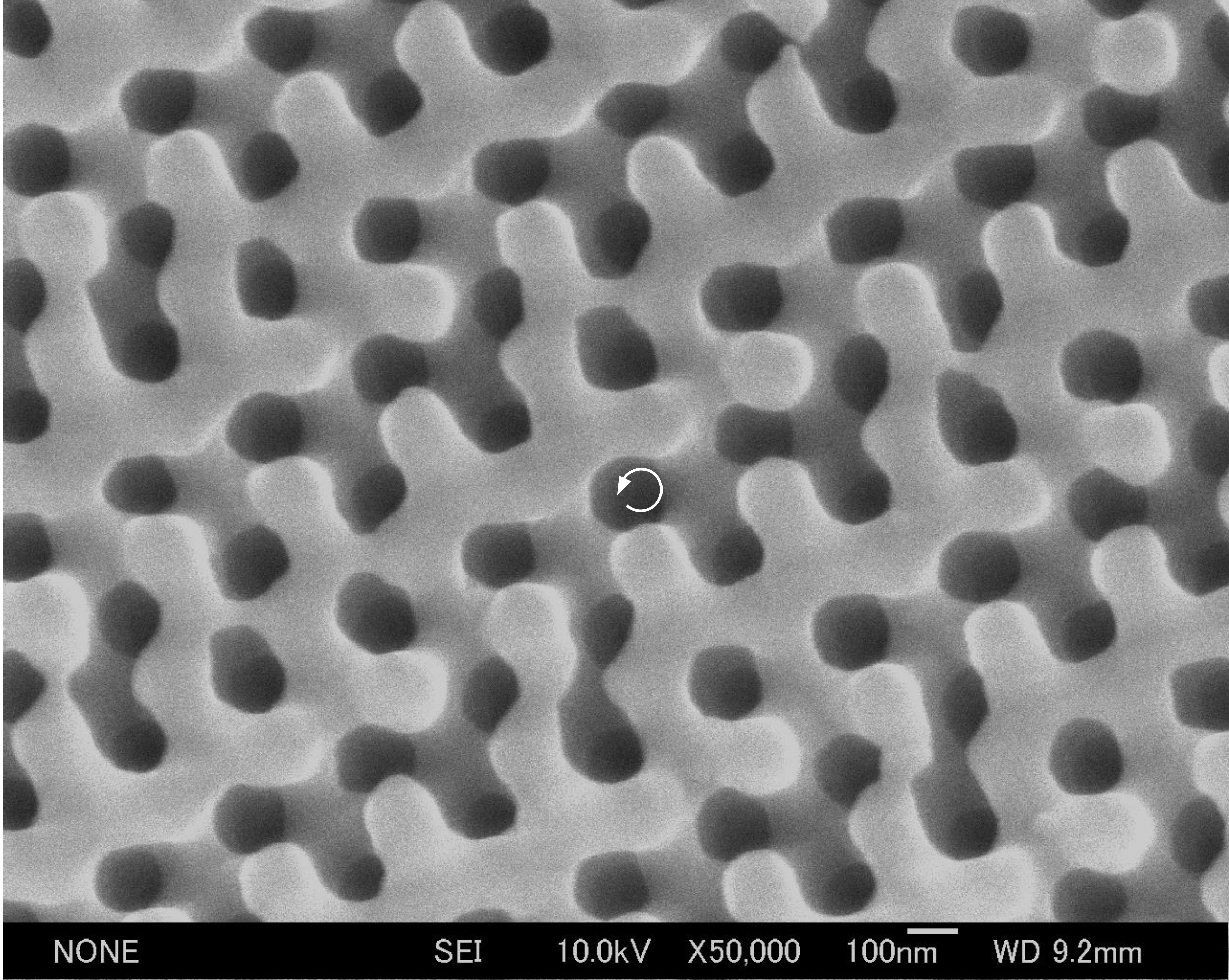

specimen No. 1
scale No. 5
domain No. 2
LH

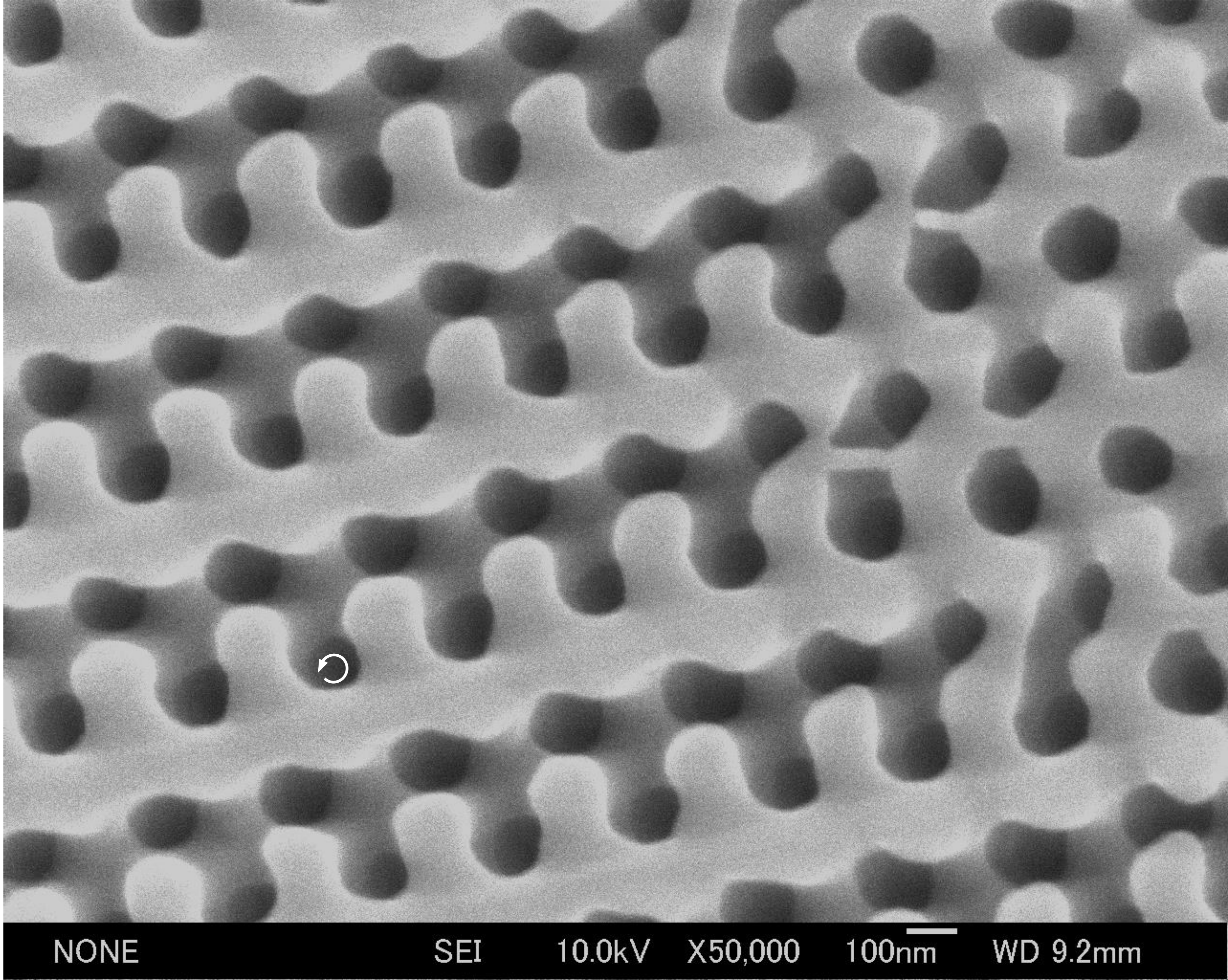

specimen No. 1
scale No. 5
domain No. 3
LH

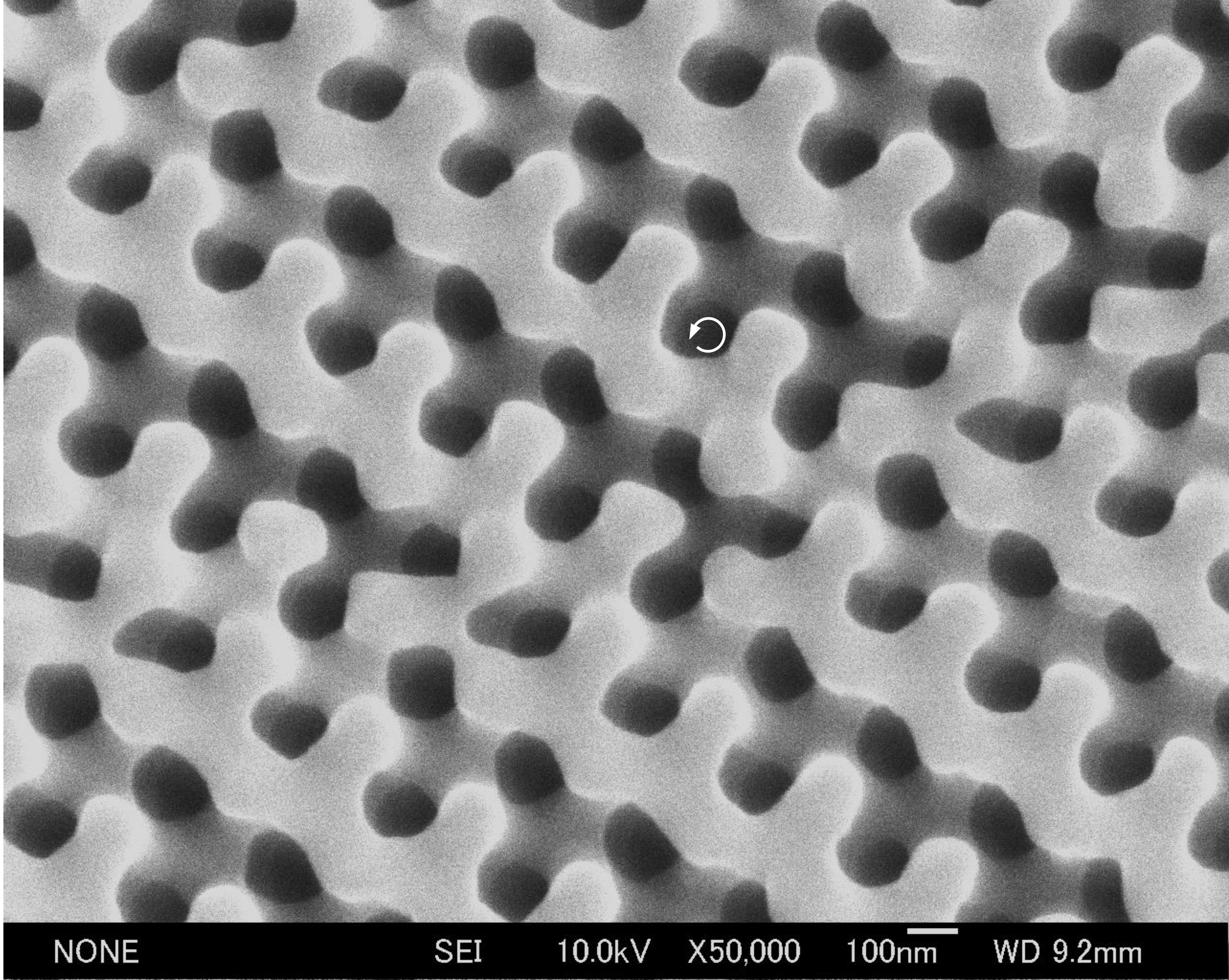

specimen No. 1
scale No. 5
domain No. 4
LH

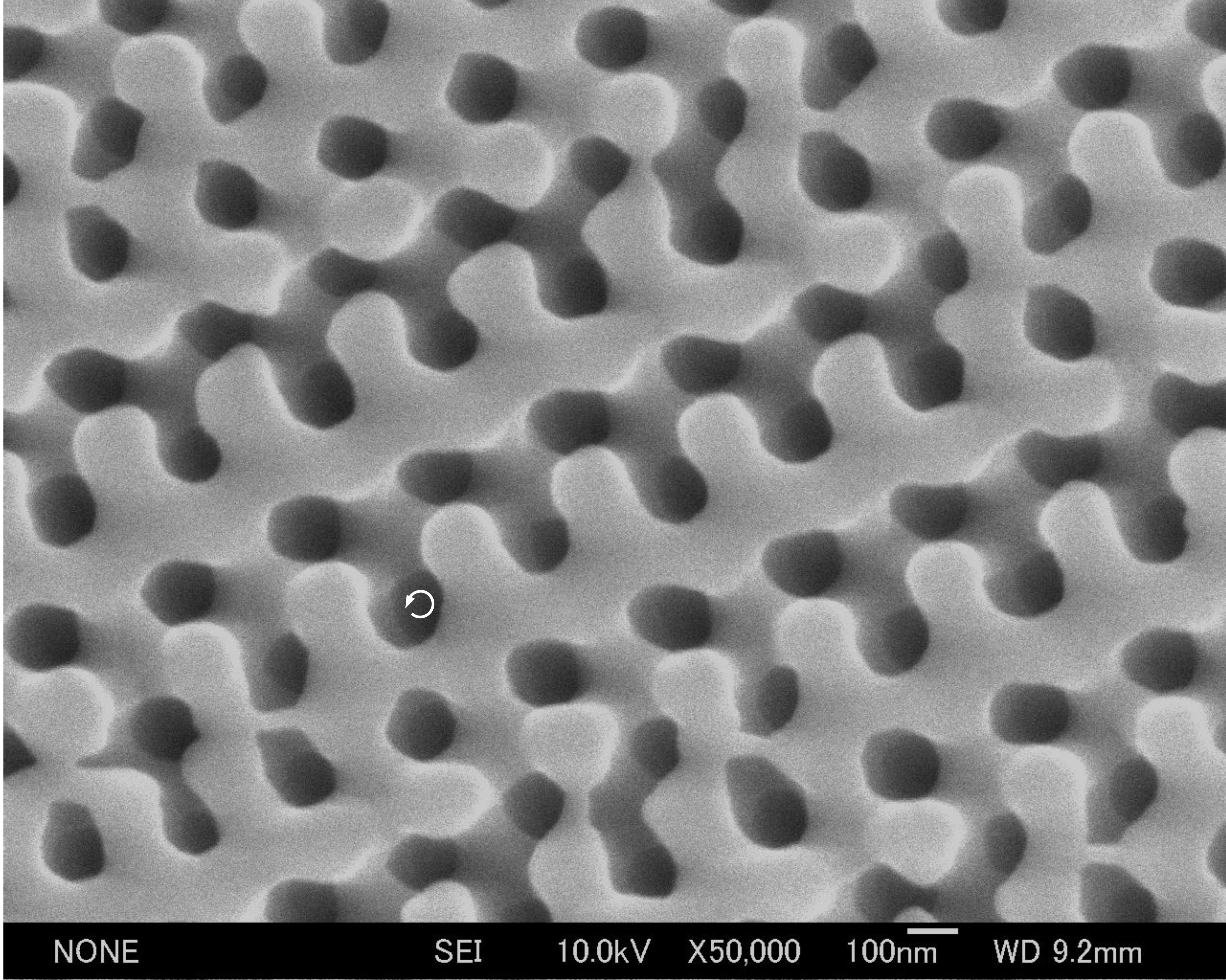

specimen No. 1
scale No. 5
domain No. 5
LH

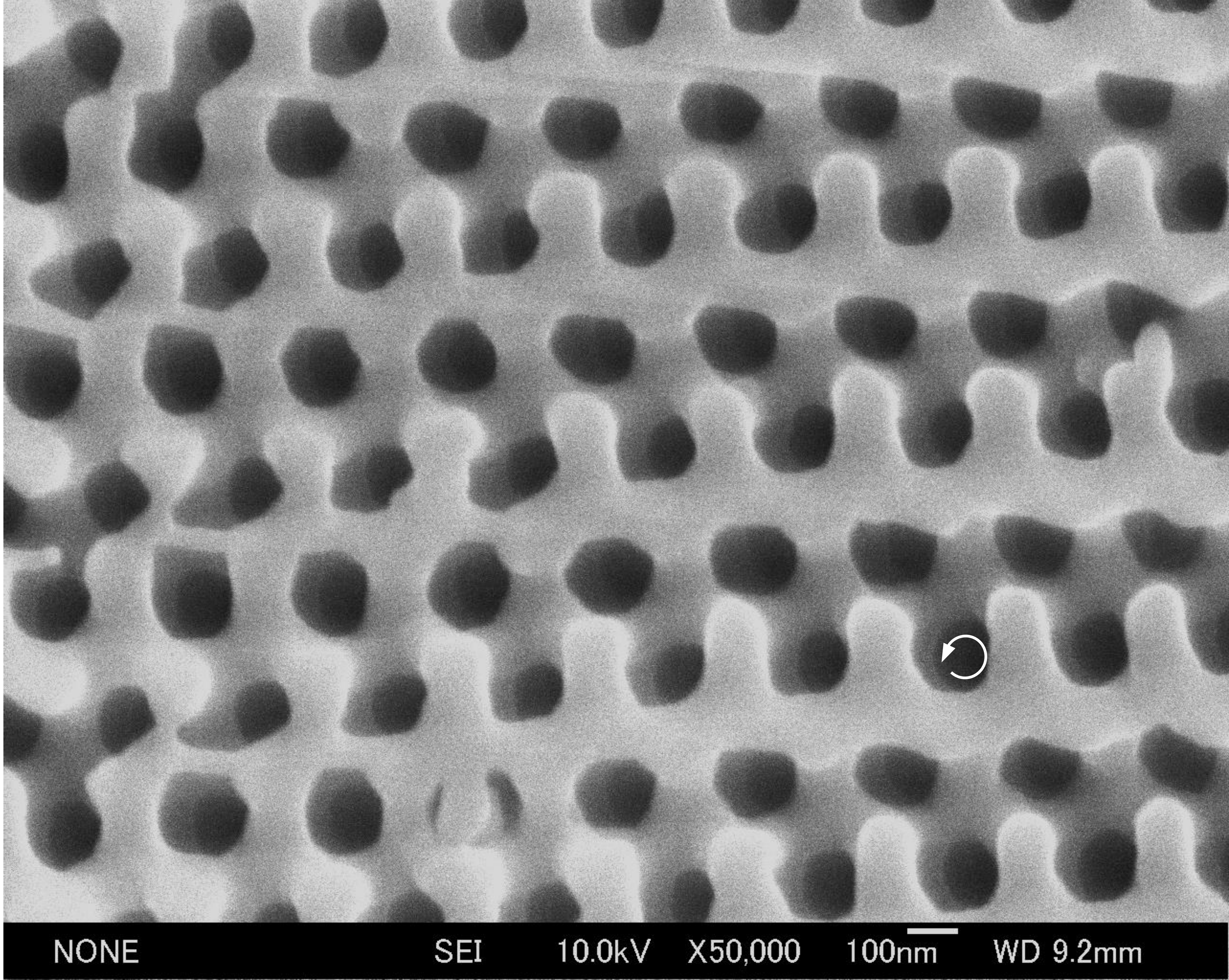

specimen No. 1
scale No. 5
domain No. 6
LH

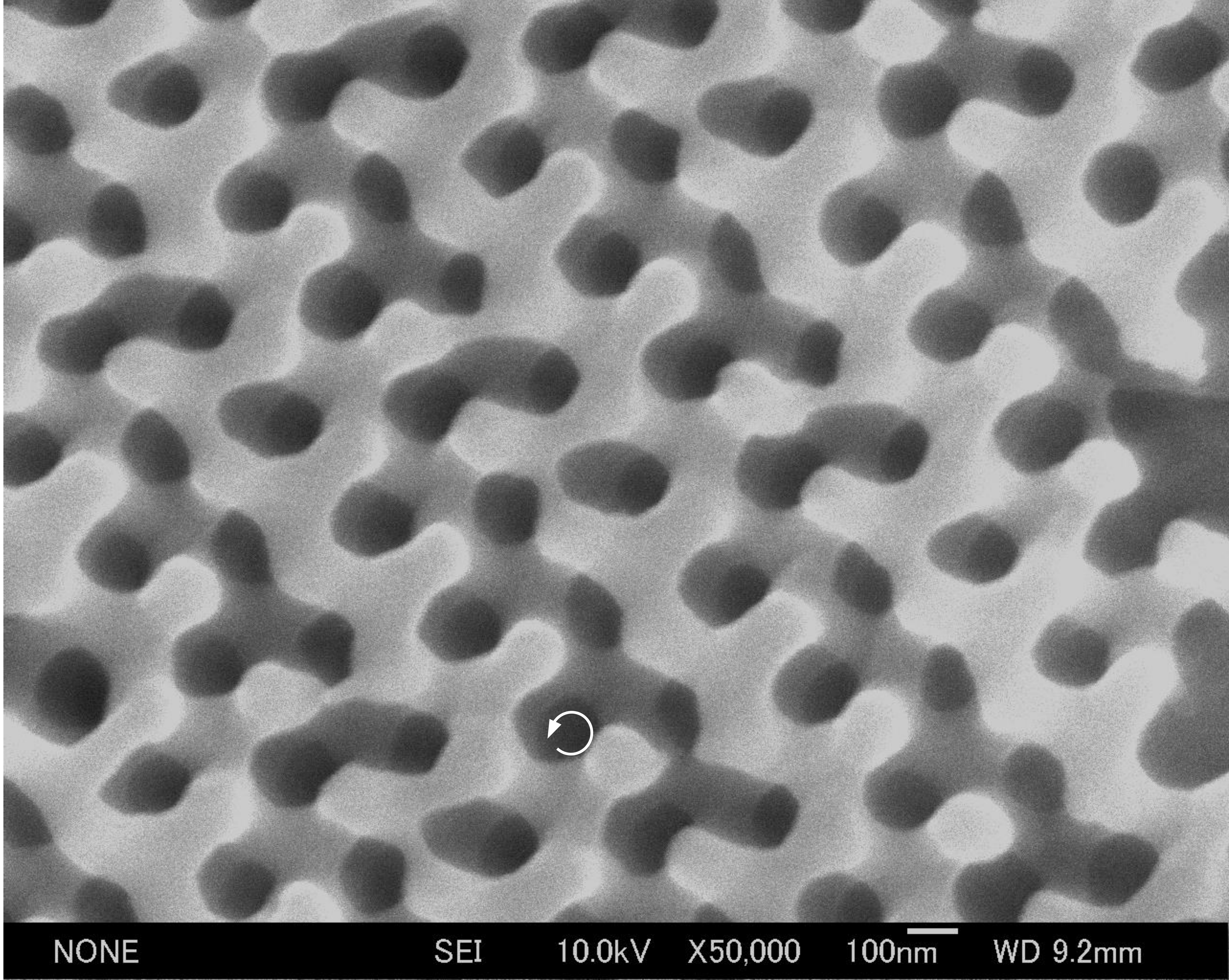

specimen No. 1
scale No. 5
domain No. 7
LH

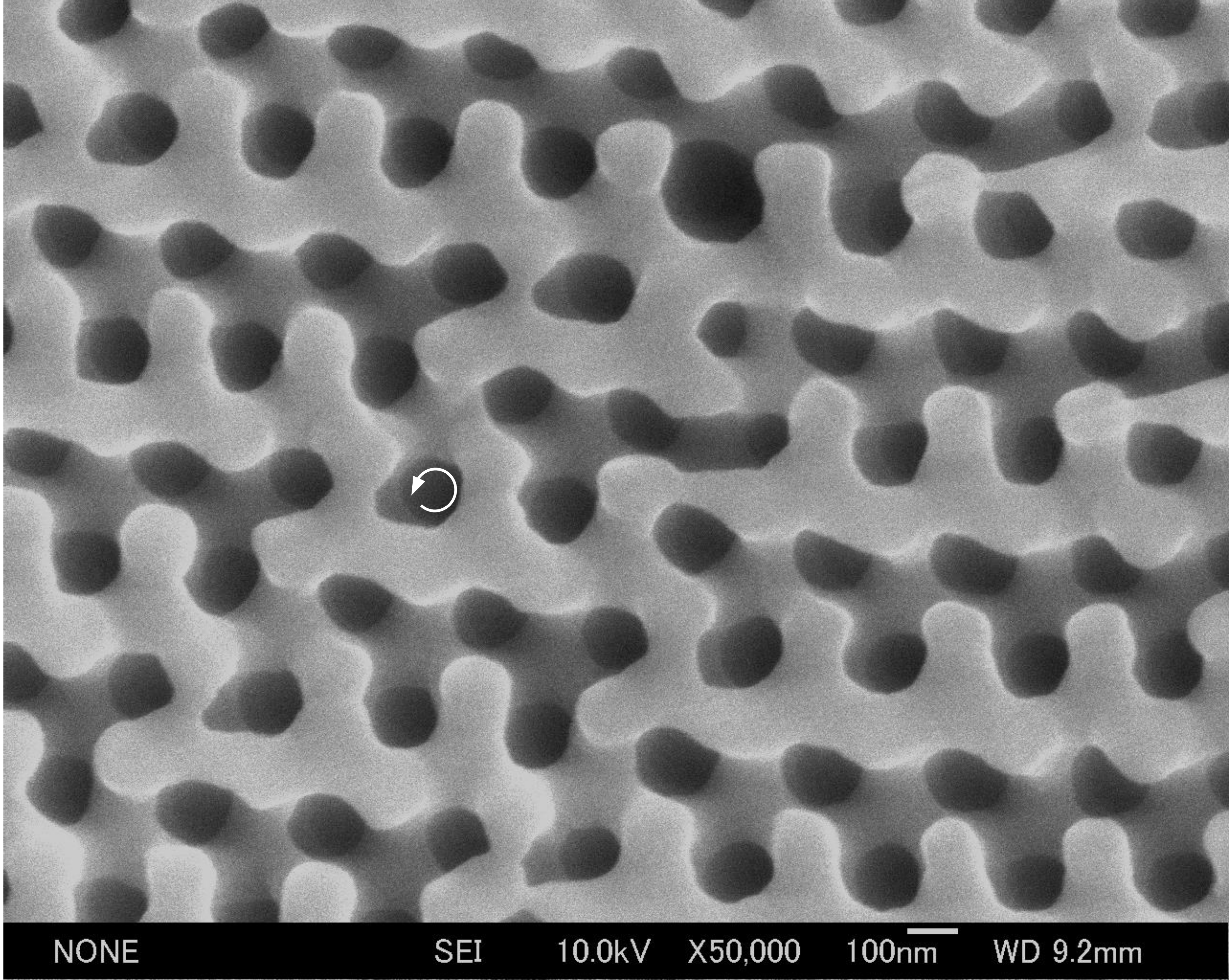

specimen No. 1
scale No. 6

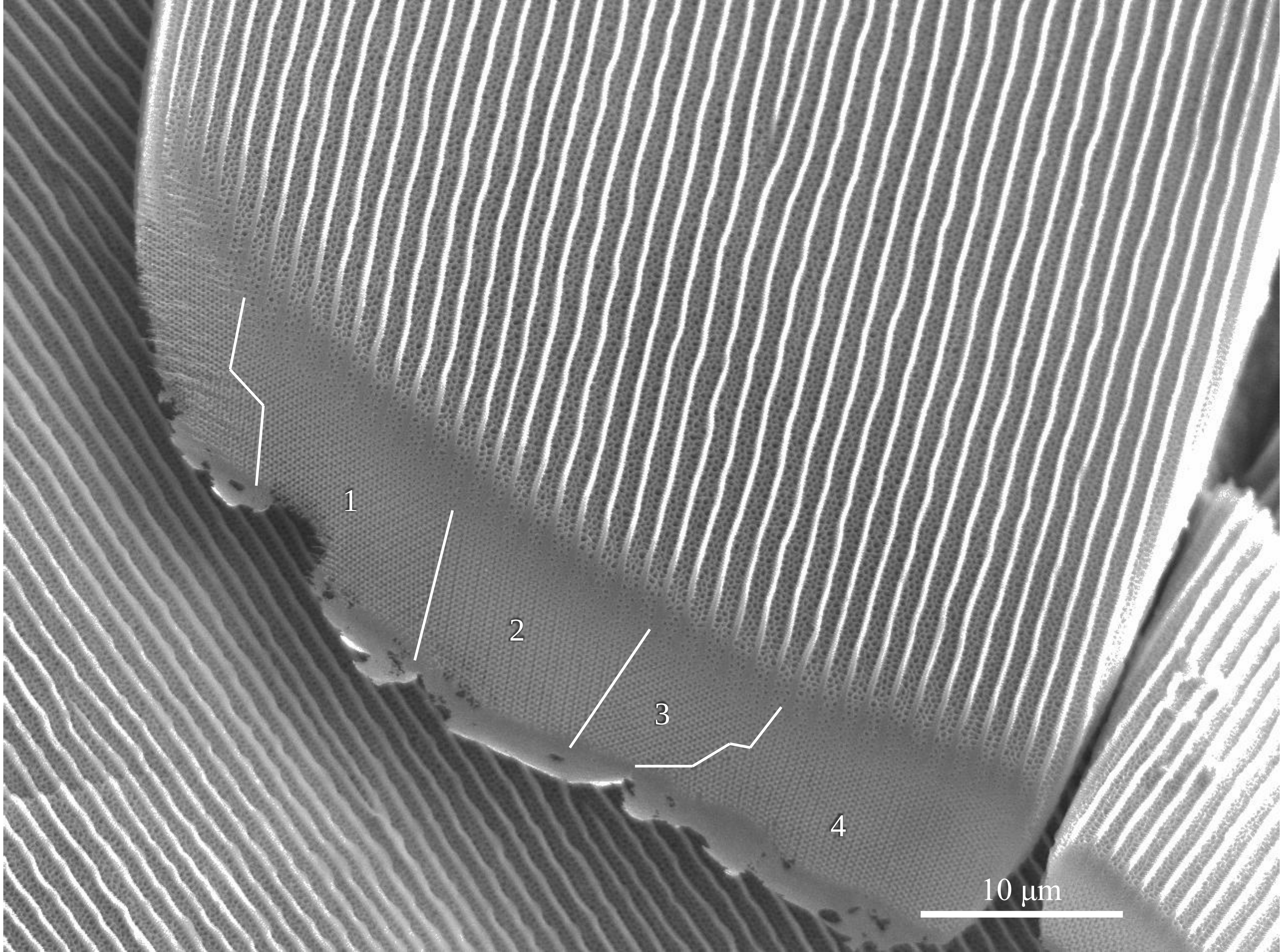

specimen No. 1
scale No. 6
domain No. 1
LH

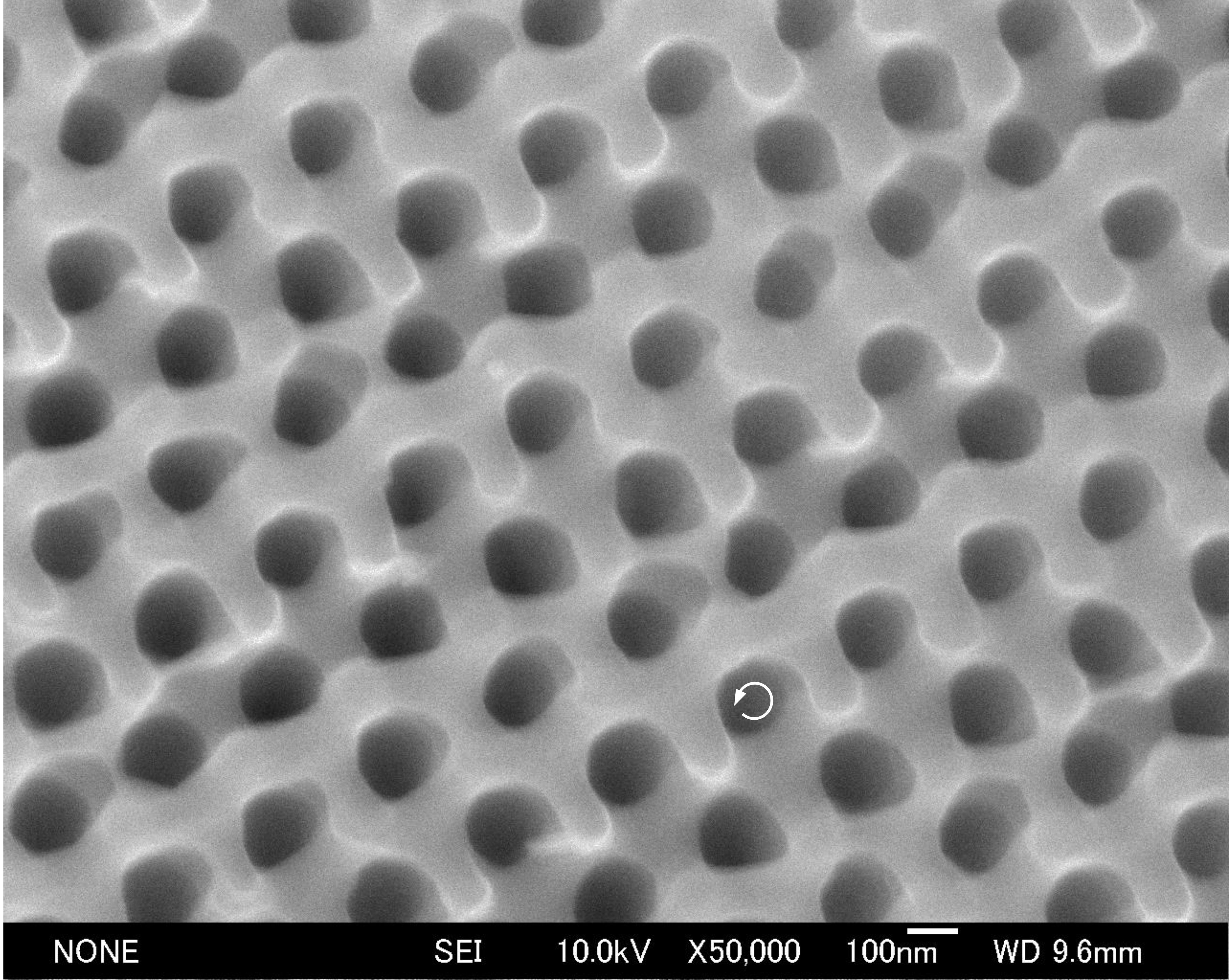

specimen No. 1
scale No. 6
domain No. 2
LH

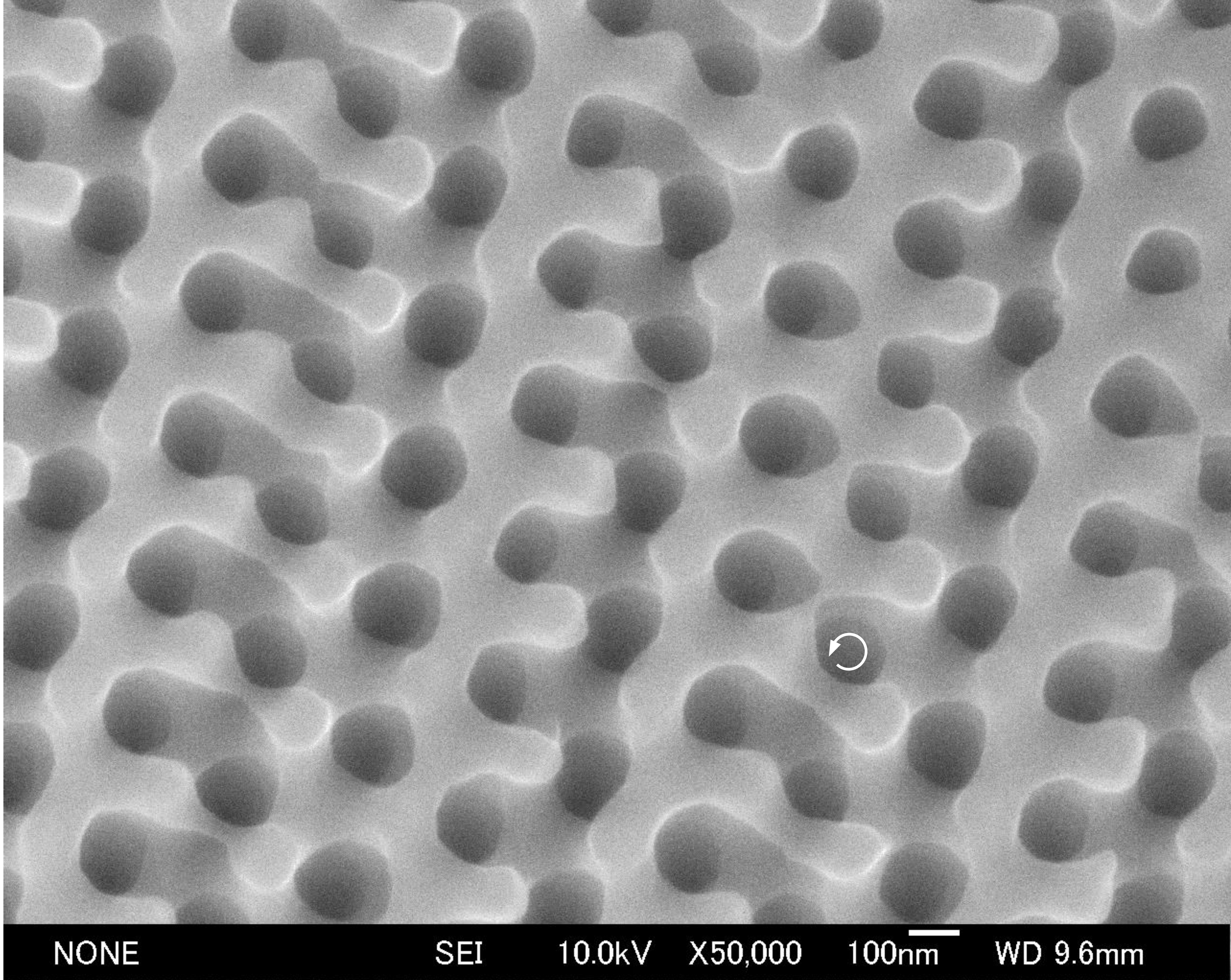

specimen No. 1
scale No. 6
domain No. 3
LH

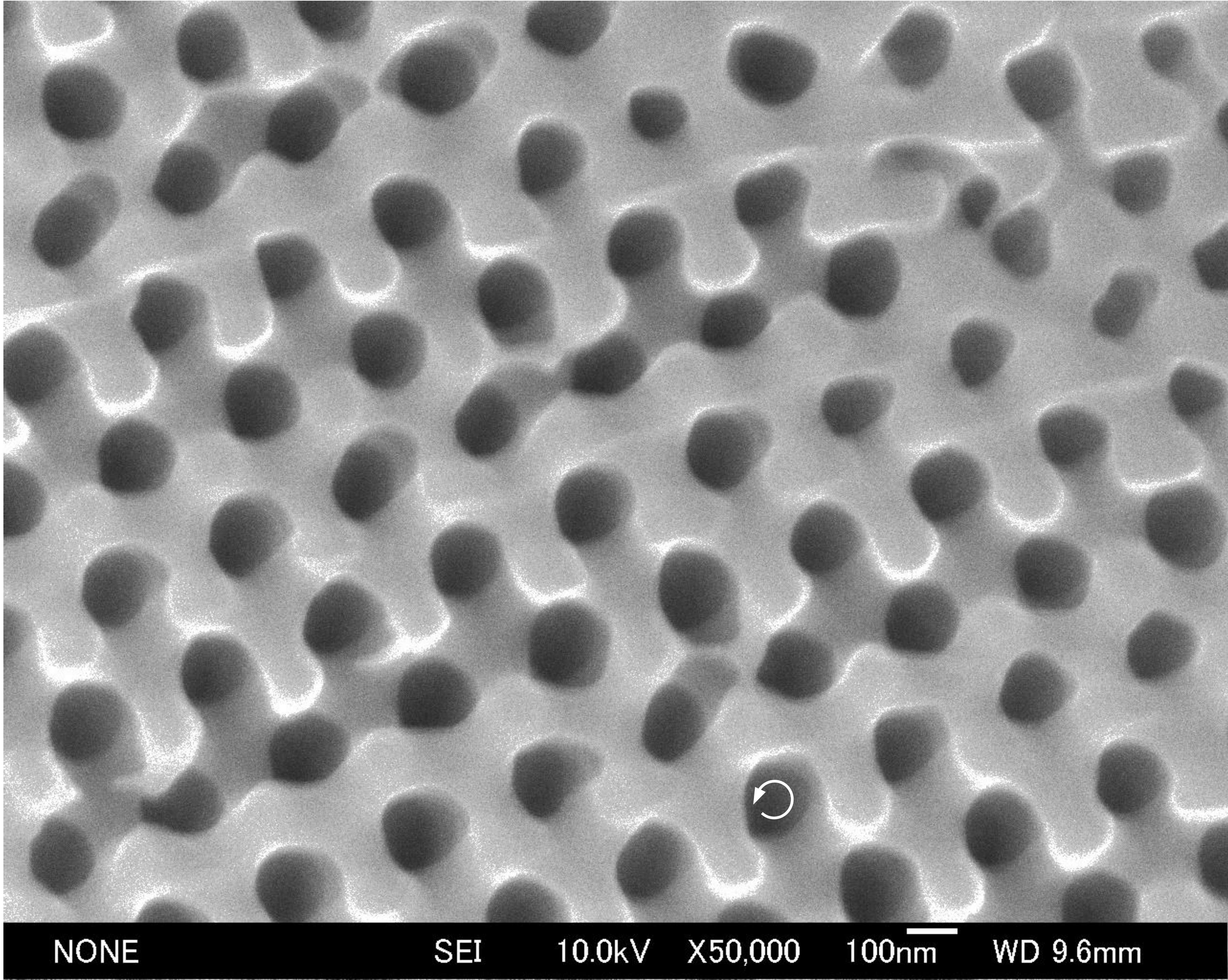

specimen No. 1
scale No. 6
domain No. 4
LH

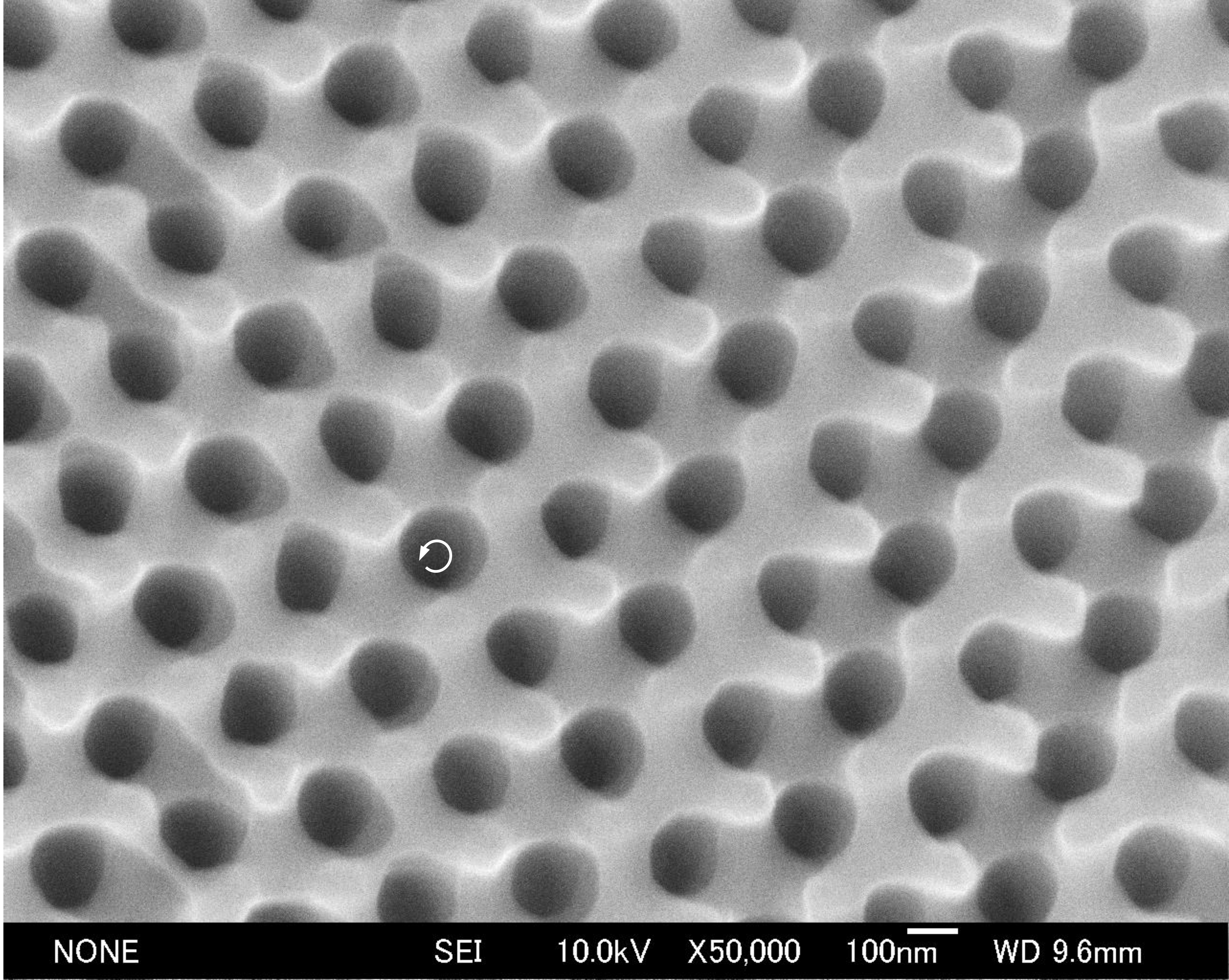

specimen No. 1
scale No. 7

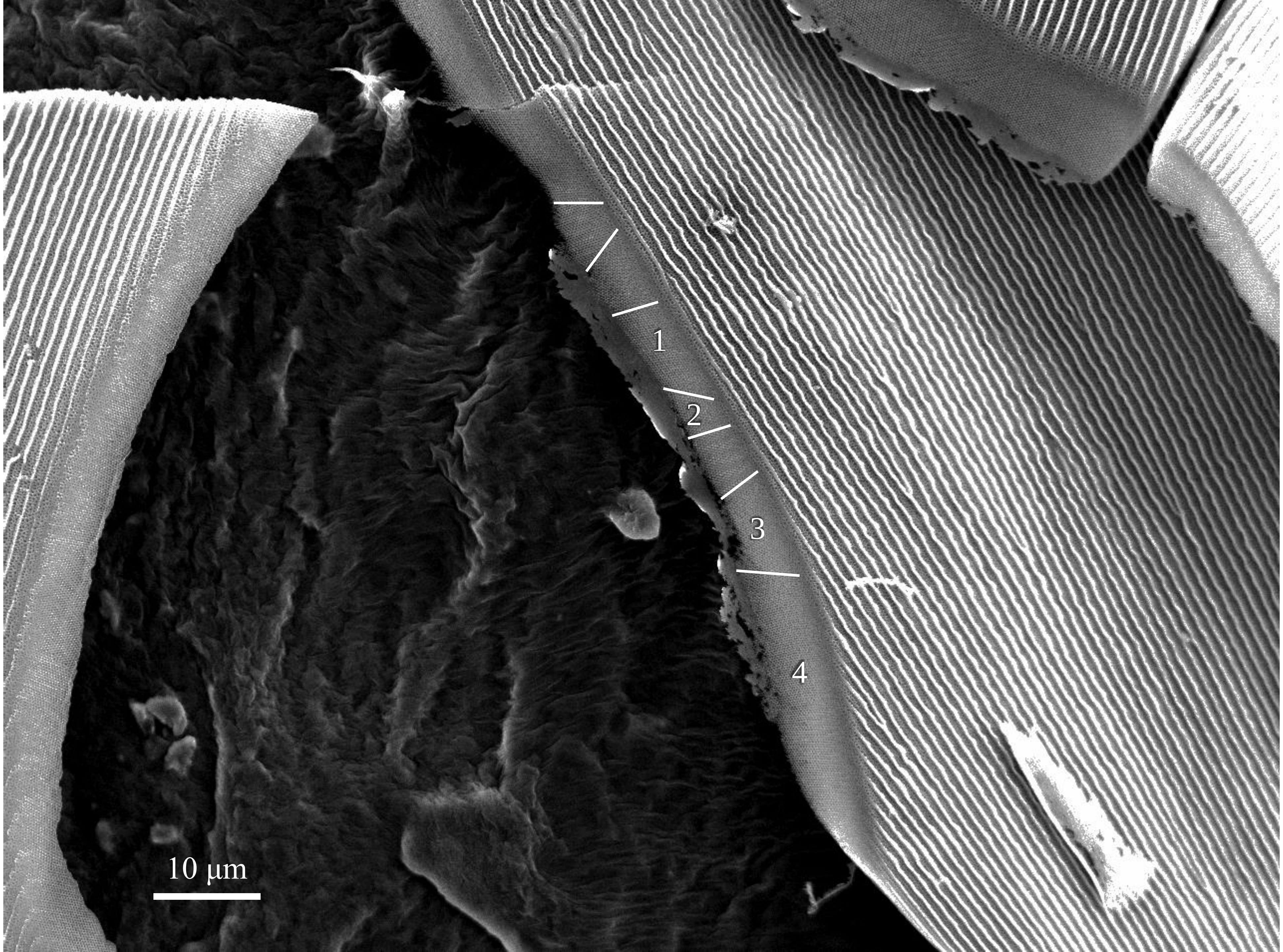

specimen No. 1
scale No. 7
domain No. 1
LH

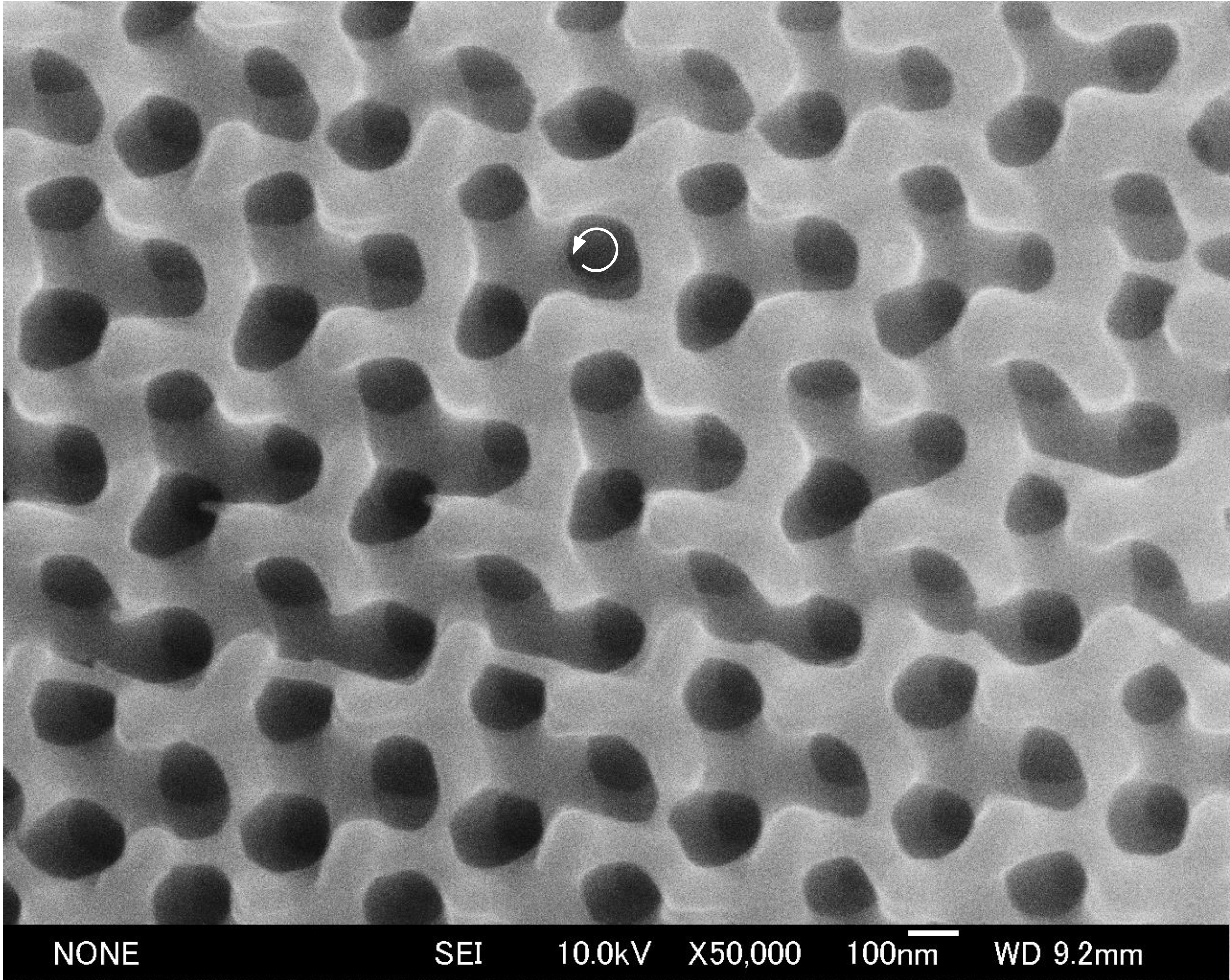

specimen No. 1
scale No. 7
domain No. 2
LH

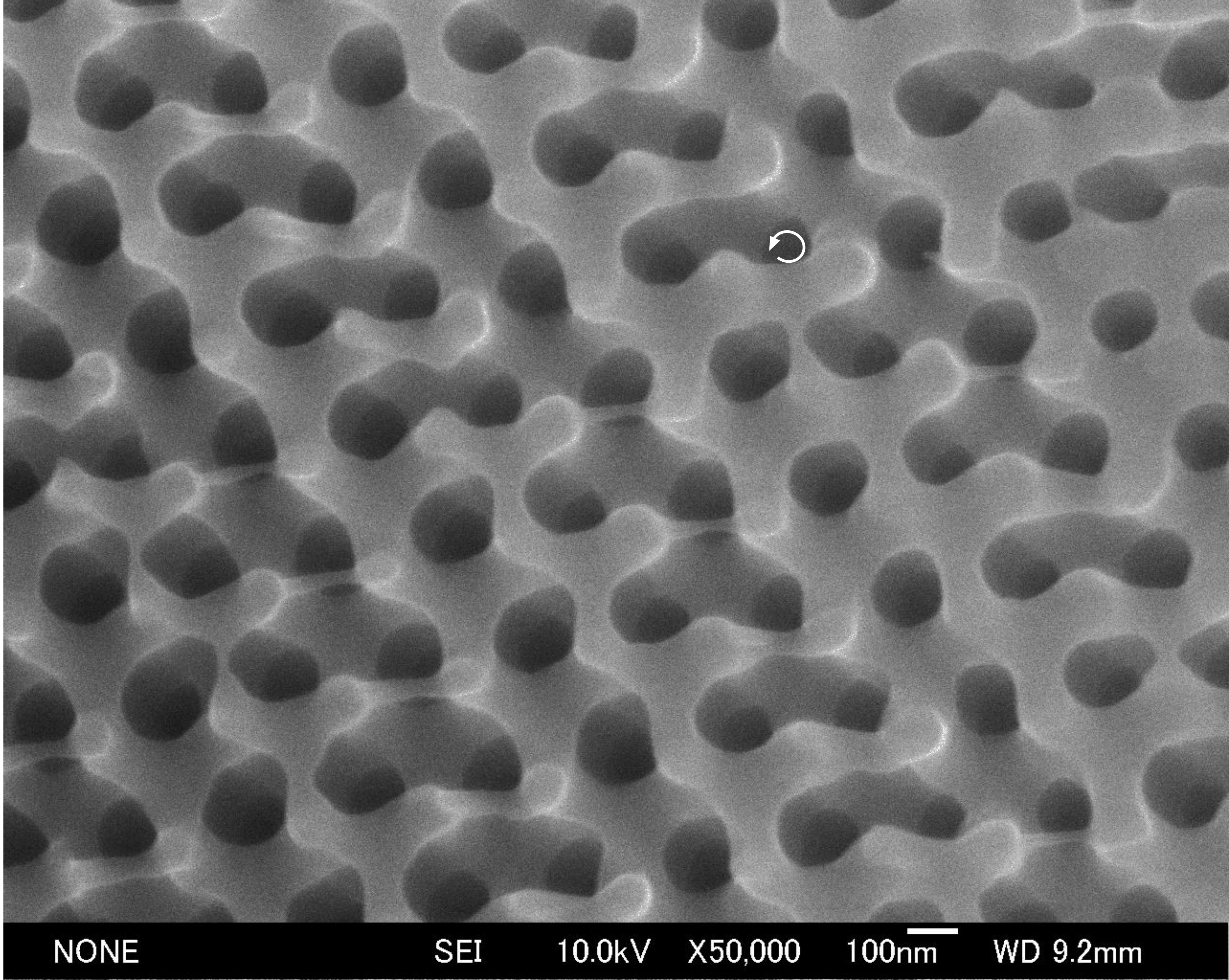

specimen No. 1
scale No. 7
domain No. 3
LH

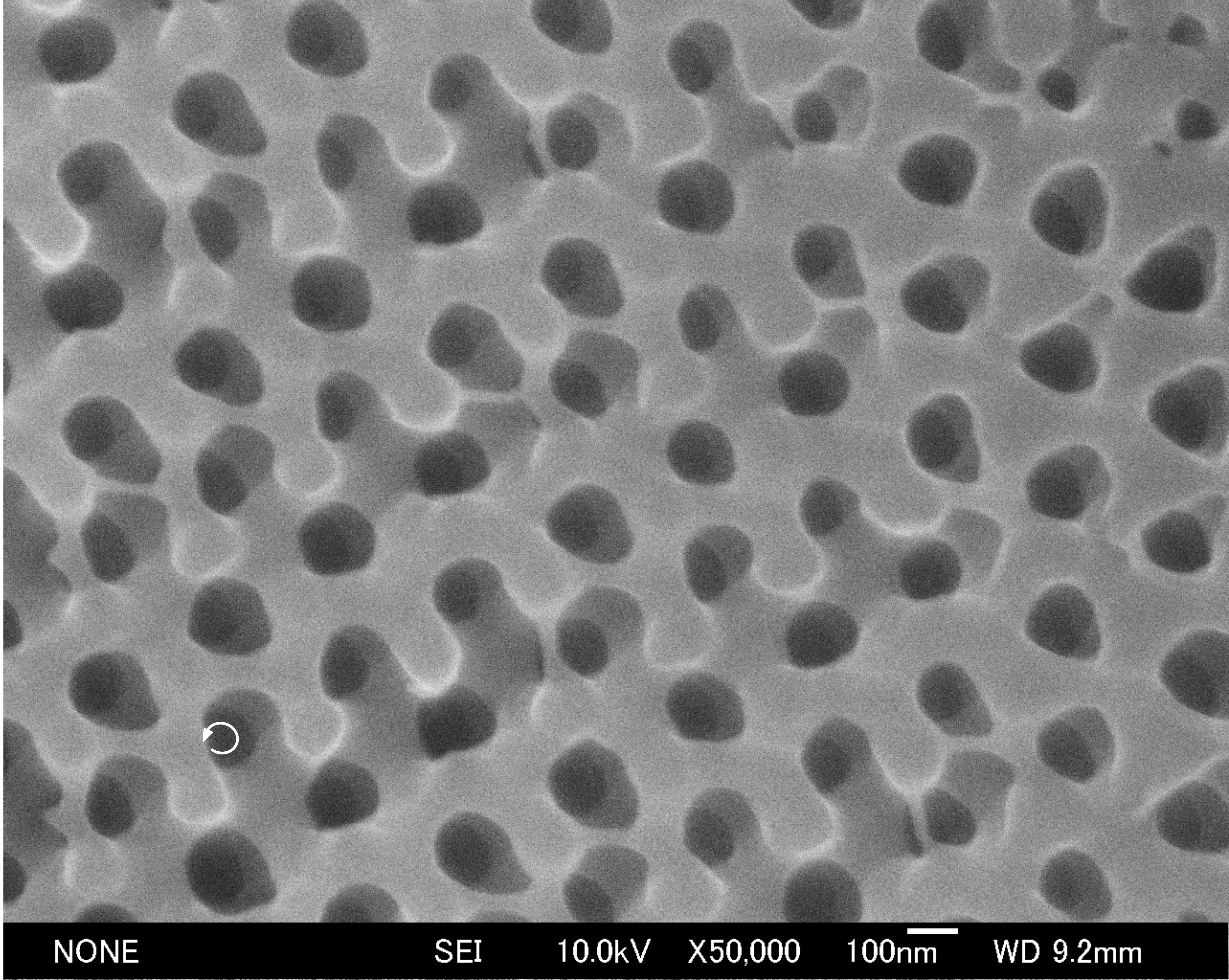

specimen No. 1
scale No. 7
domain No. 4
LH

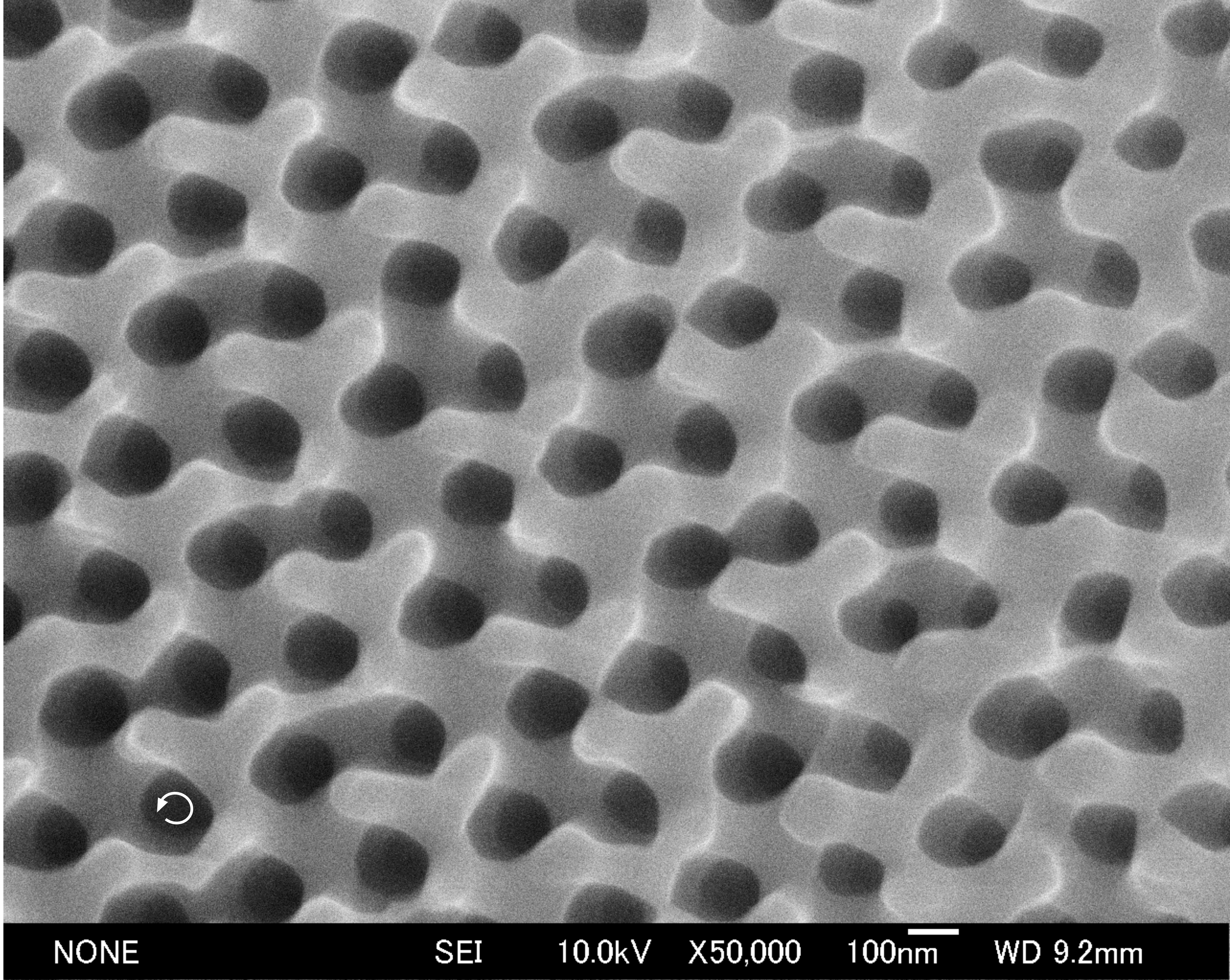

specimen No. 1
scale No. 8

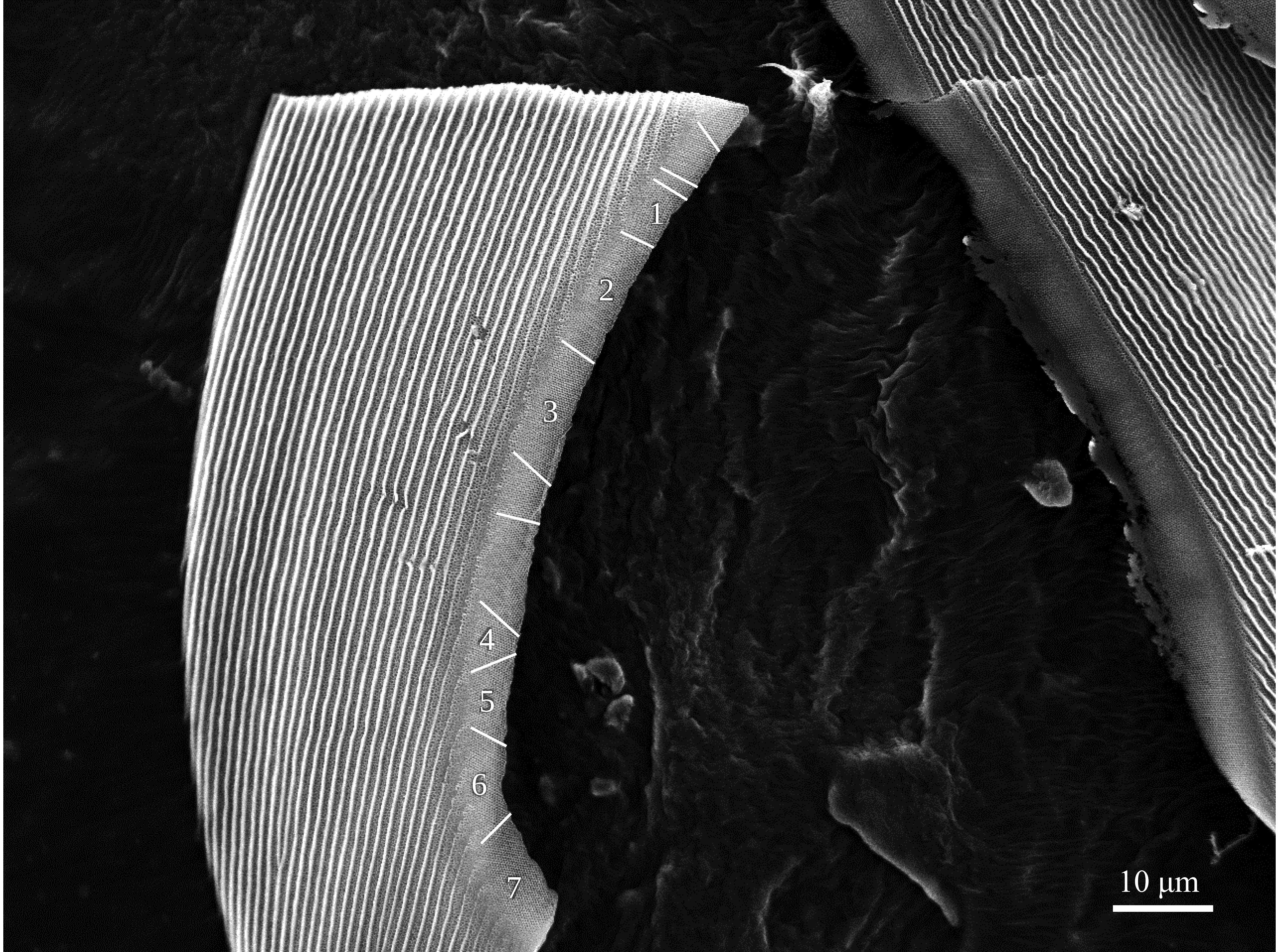

specimen No. 1
scale No. 8
domain No. 1
LH

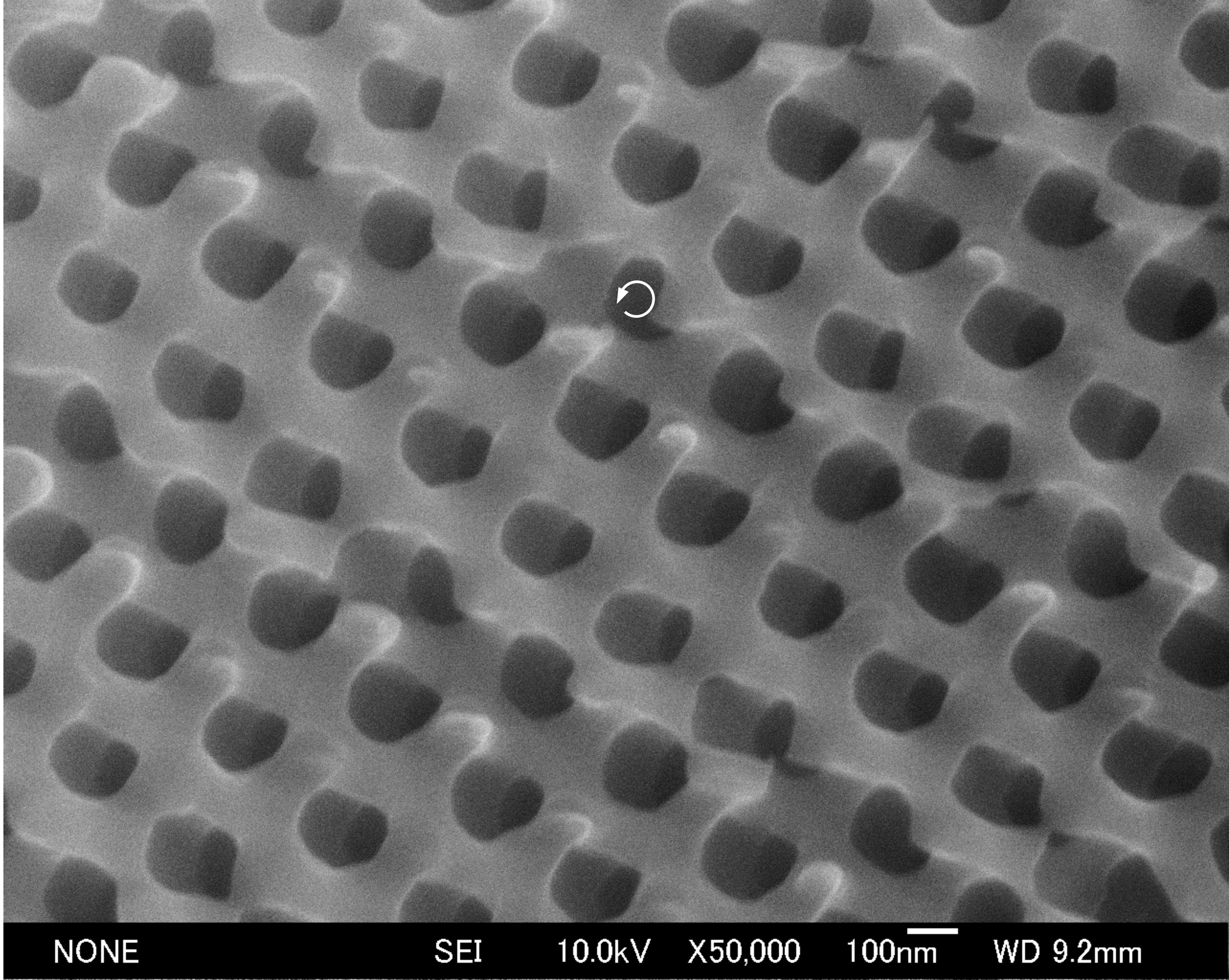

specimen No. 1
scale No. 8
domain No. 2
LH

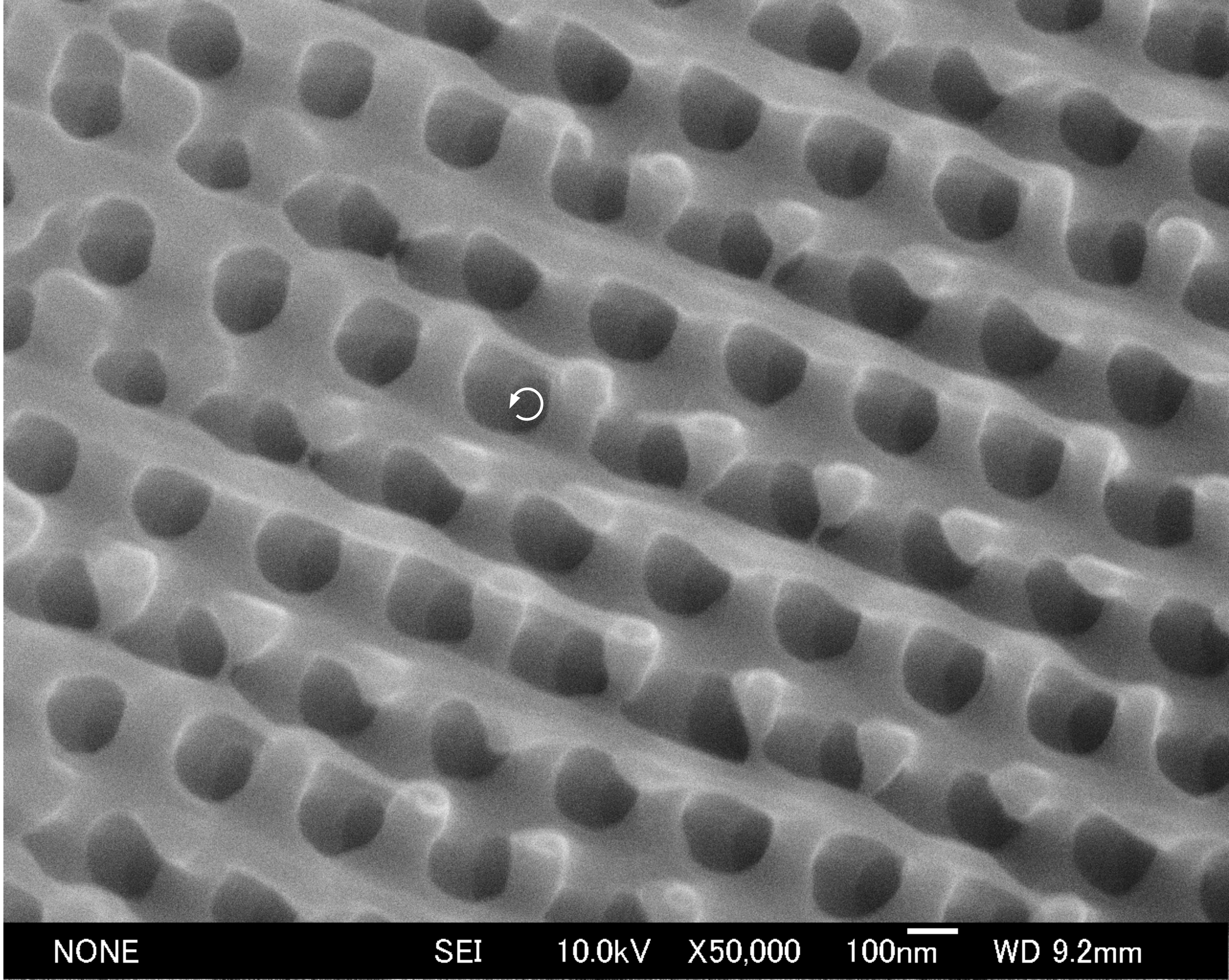

specimen No. 1
scale No. 8
domain No. 3
LH

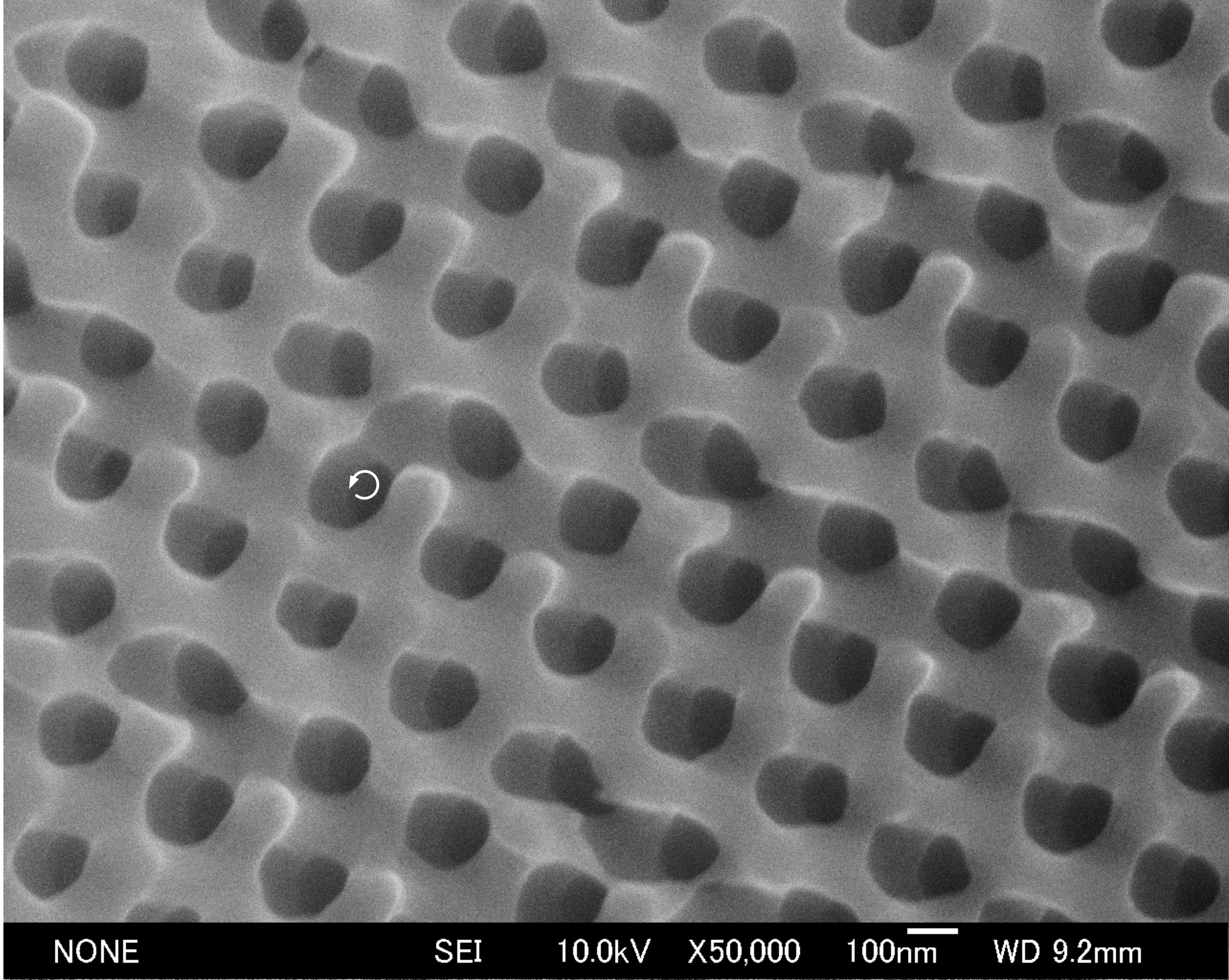

specimen No. 1
scale No. 8
domain No. 4
LH

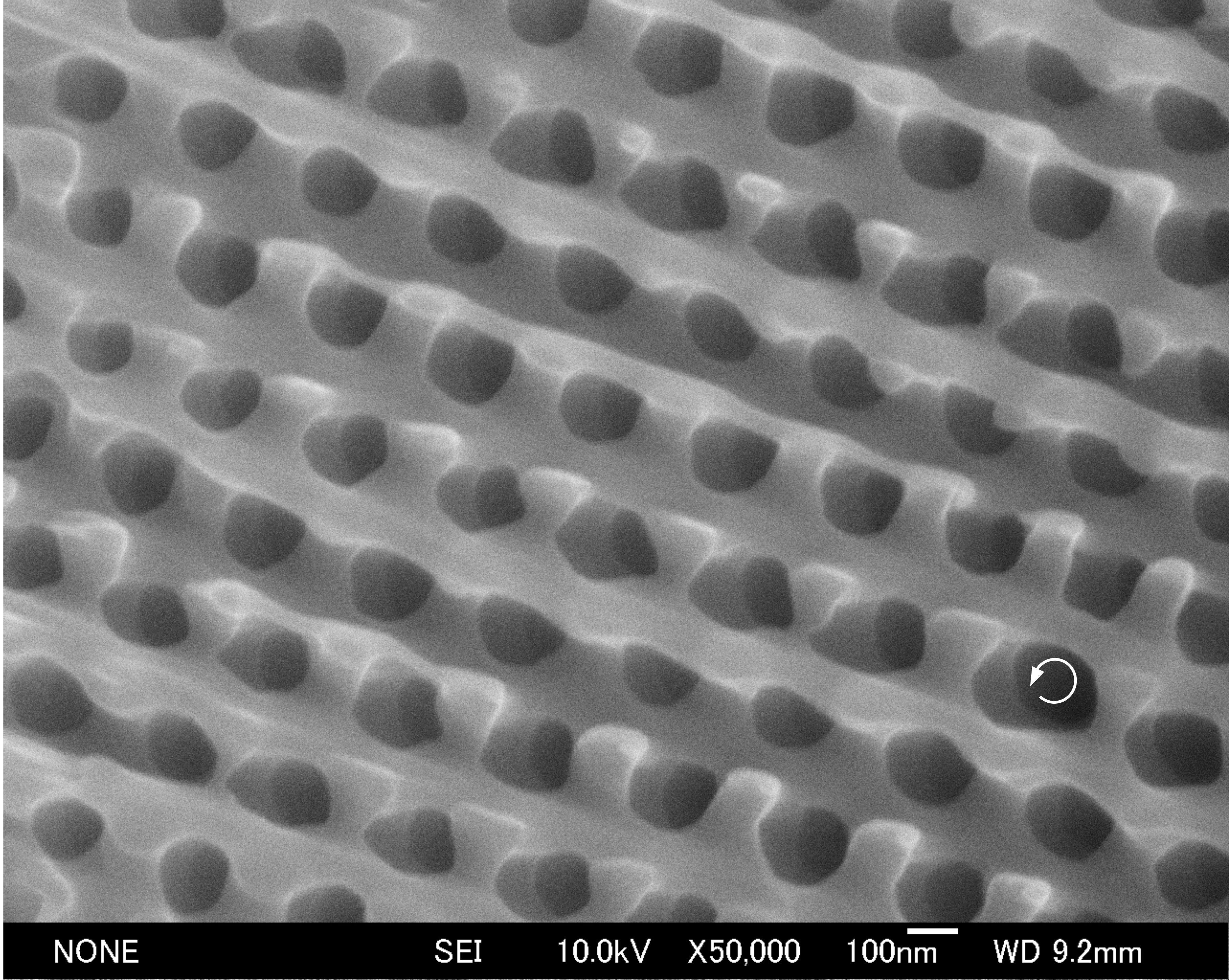

specimen No. 1
scale No. 8
domain No. 5
LH

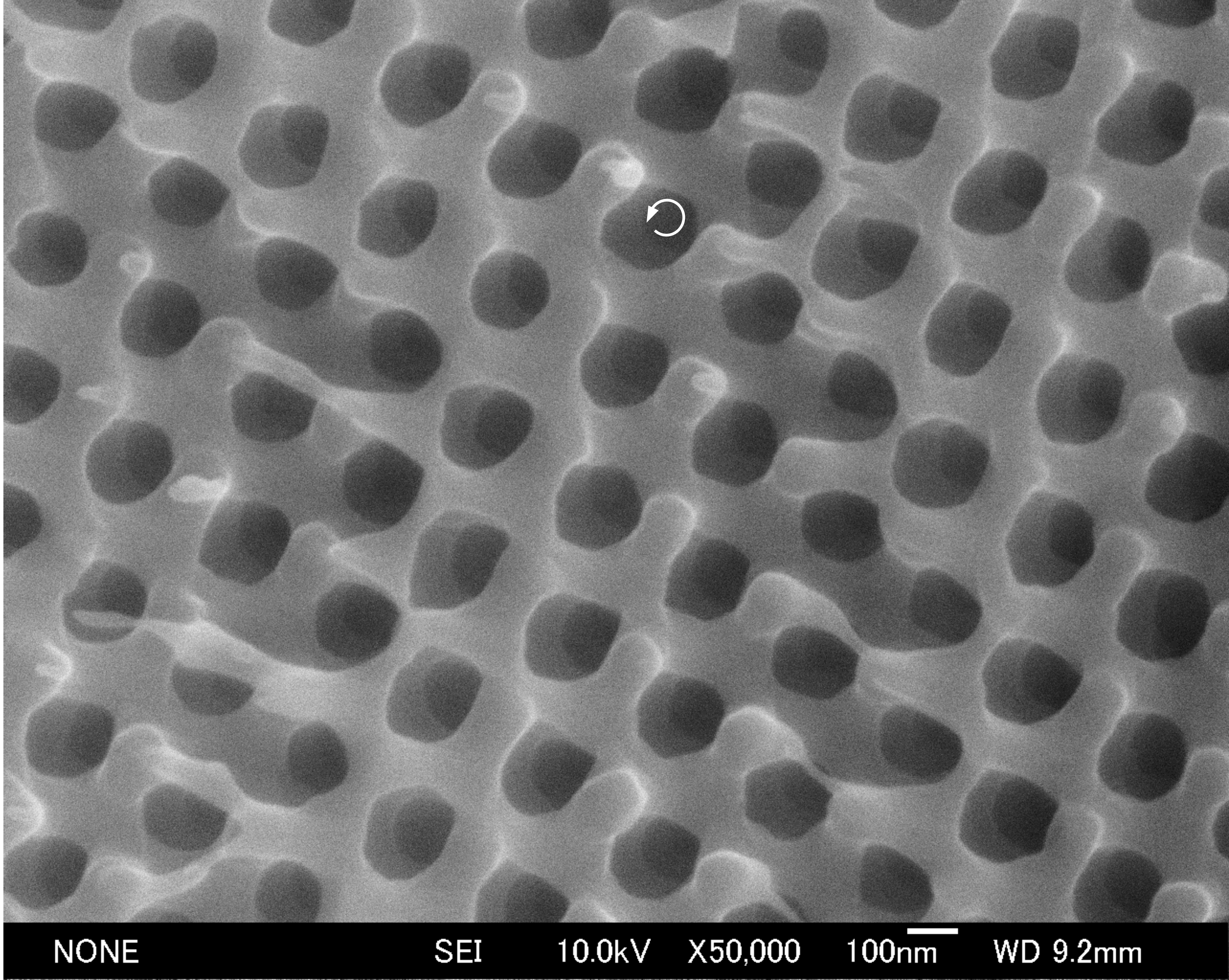

specimen No. 1
scale No. 8
domain No. 6
LH

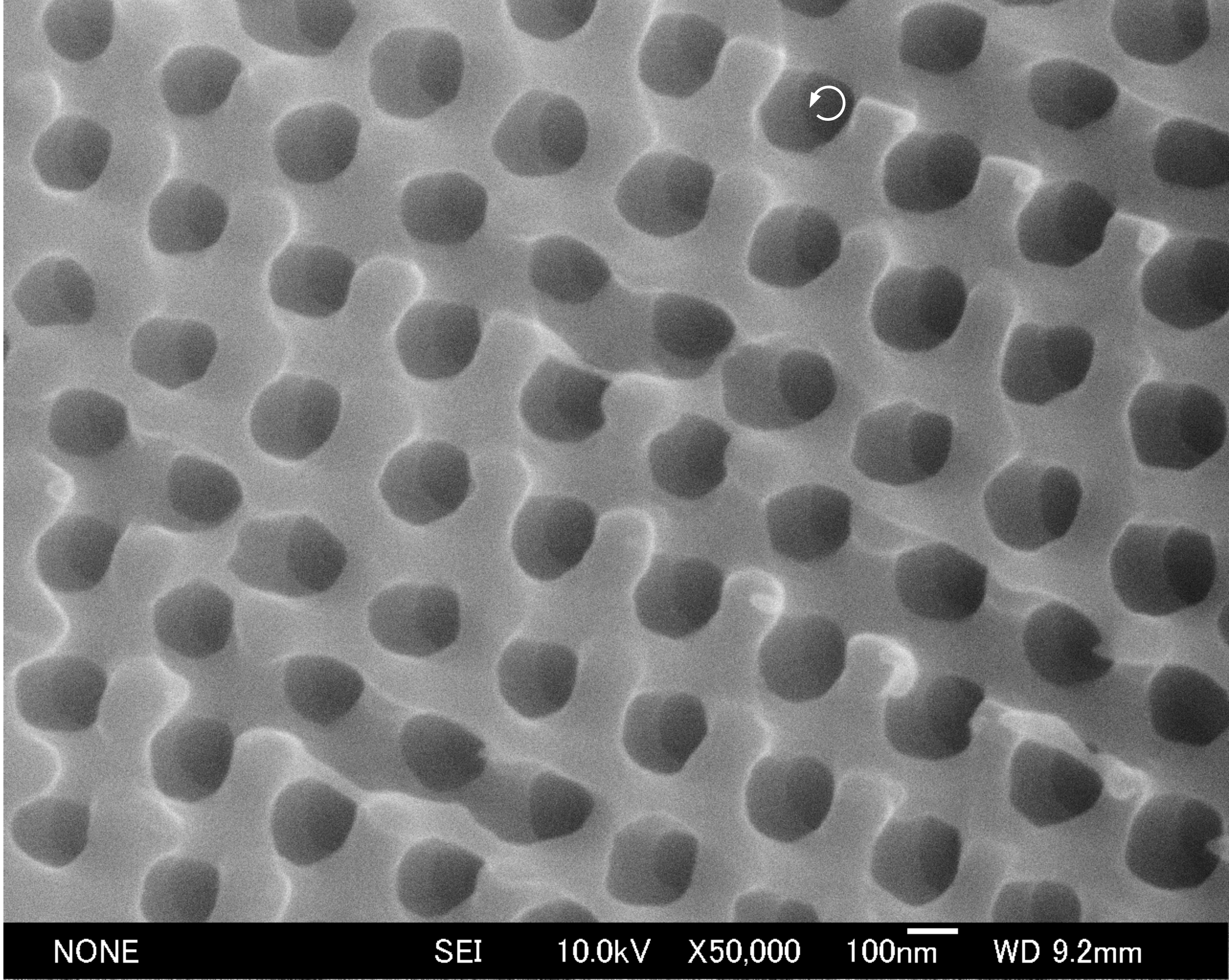

specimen No. 1
scale No. 8
domain No. 7
LH

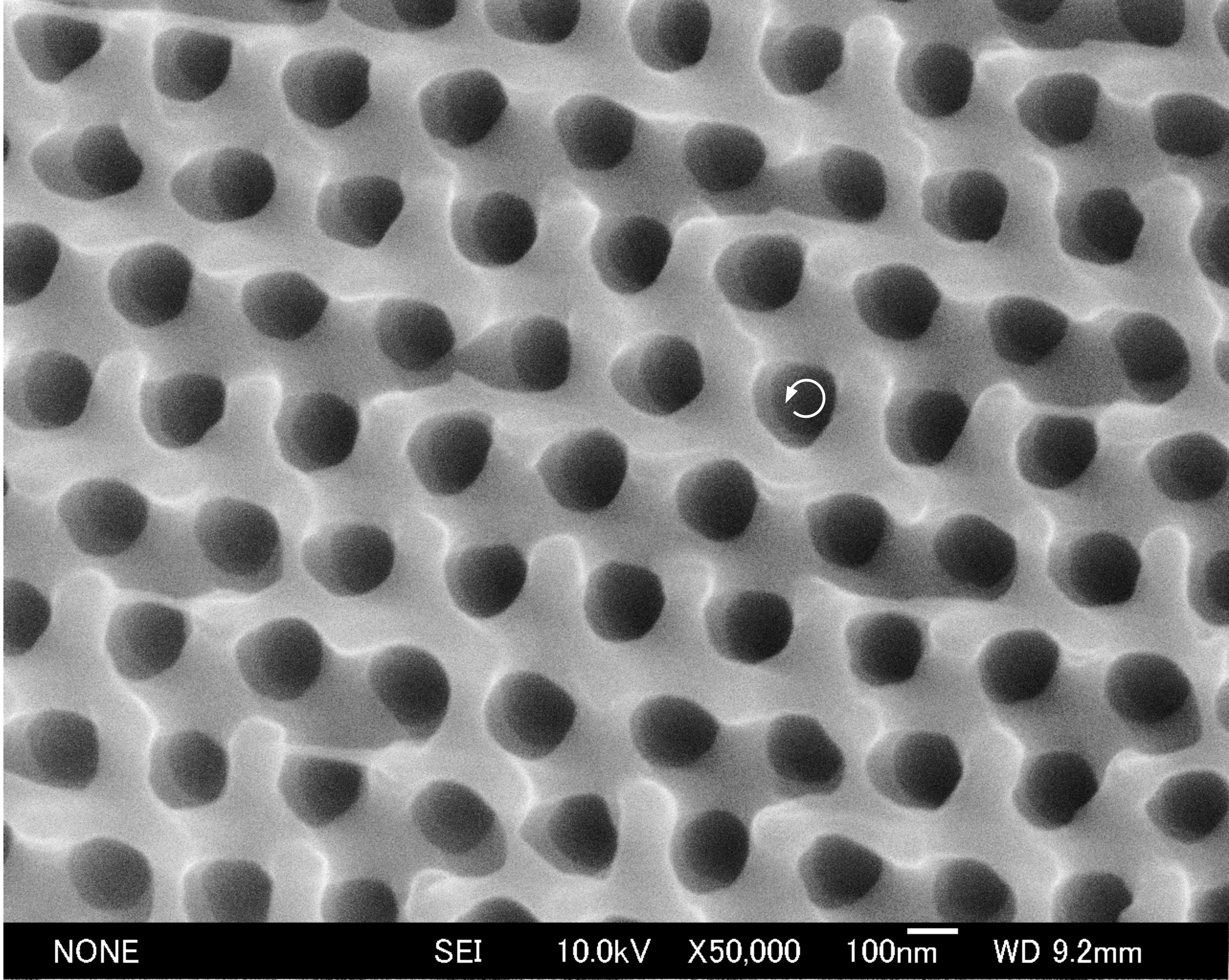

specimen No. 1
scale No. 9

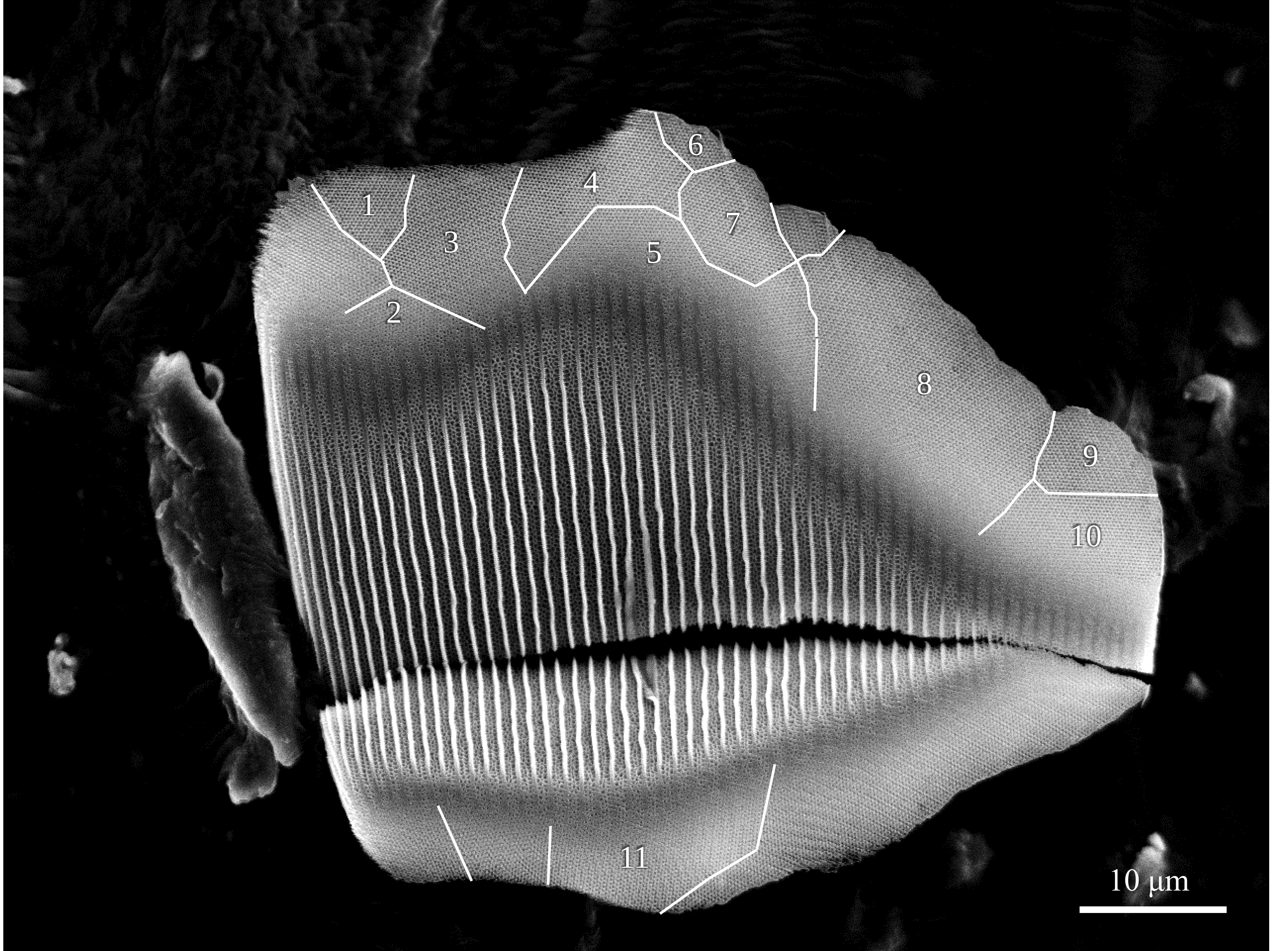

specimen No. 1
scale No. 9
domain No. 1
LH

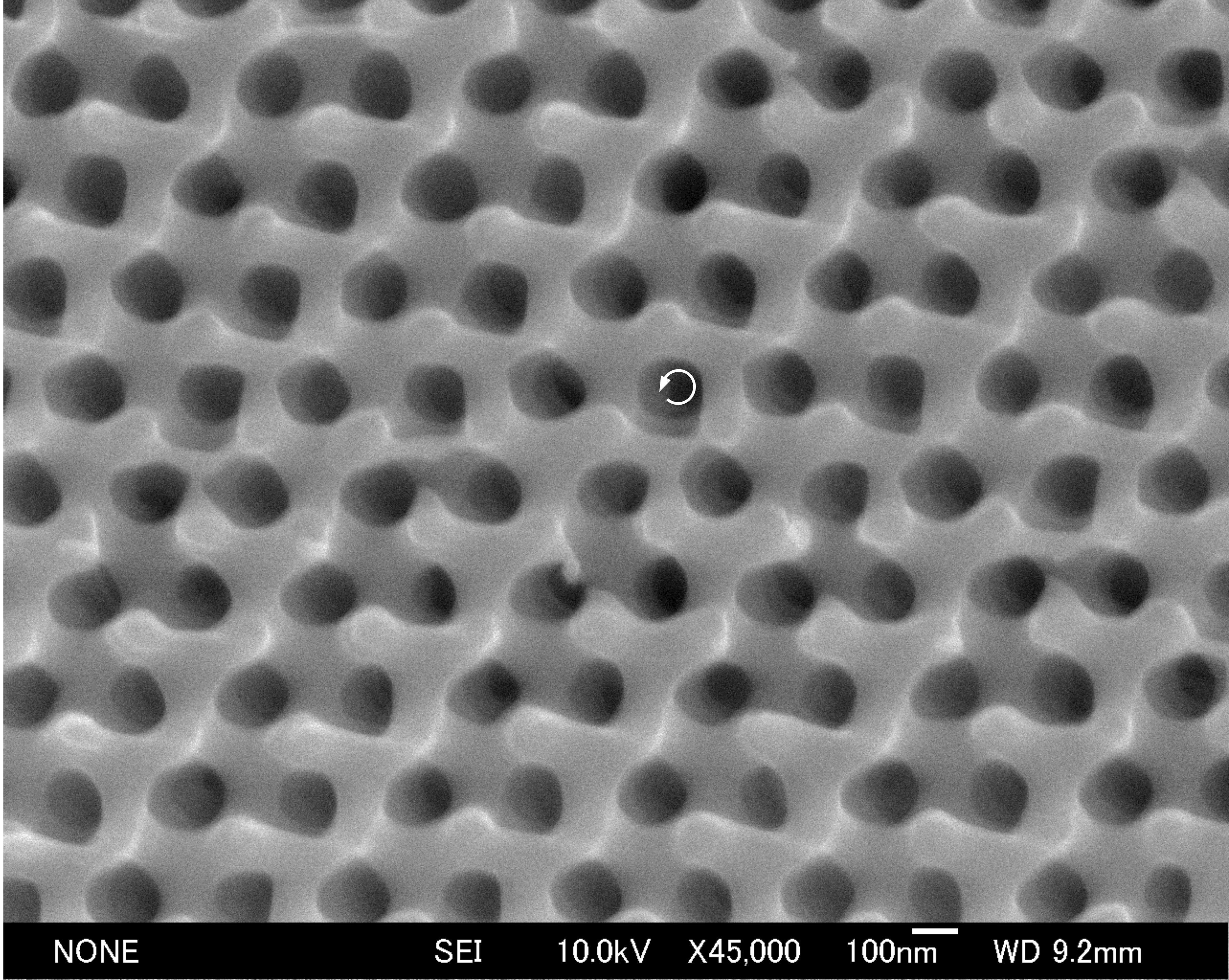

specimen No. 1
scale No. 9
domain No. 2
LH

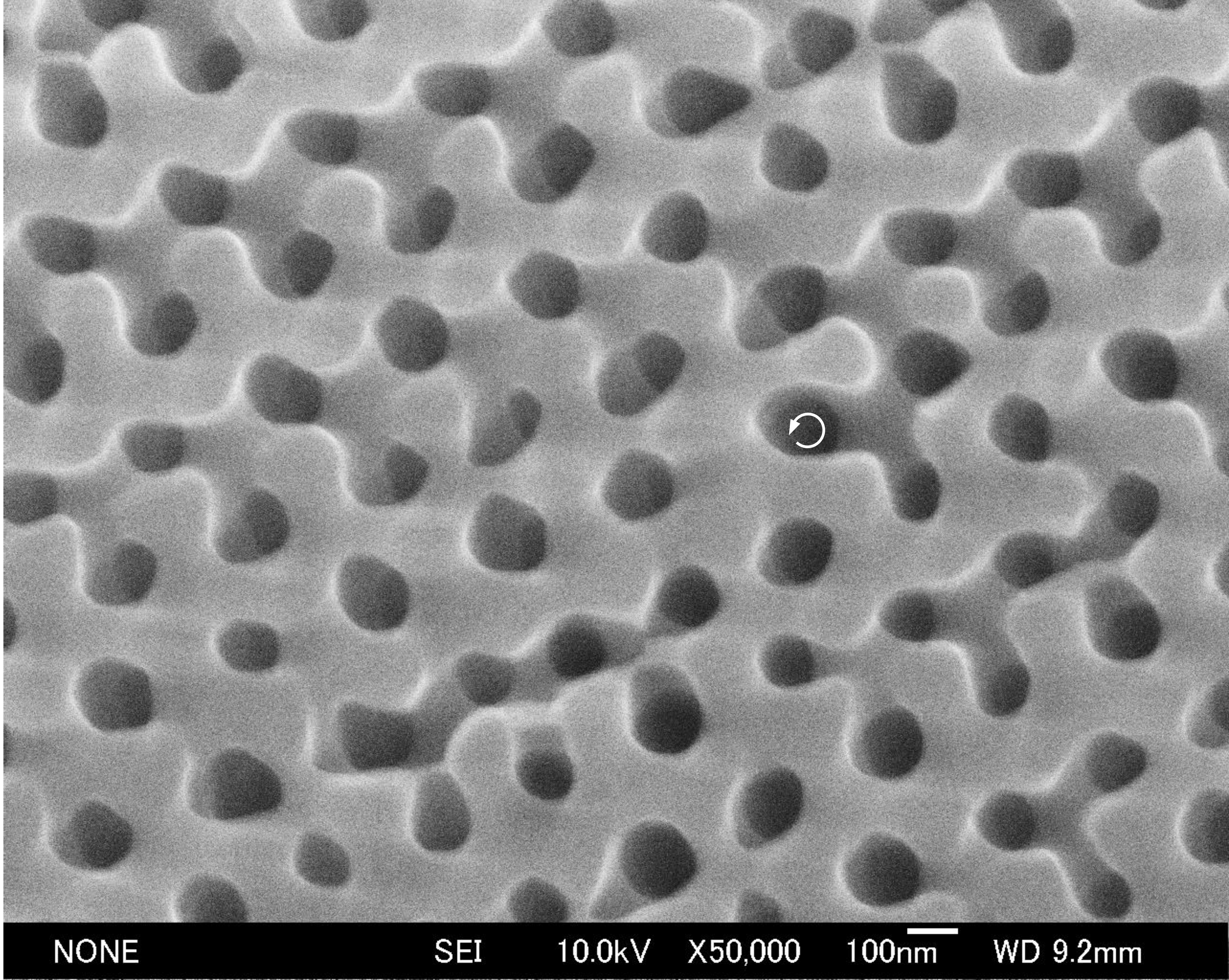

specimen No. 1
scale No. 9
domain No. 3
LH

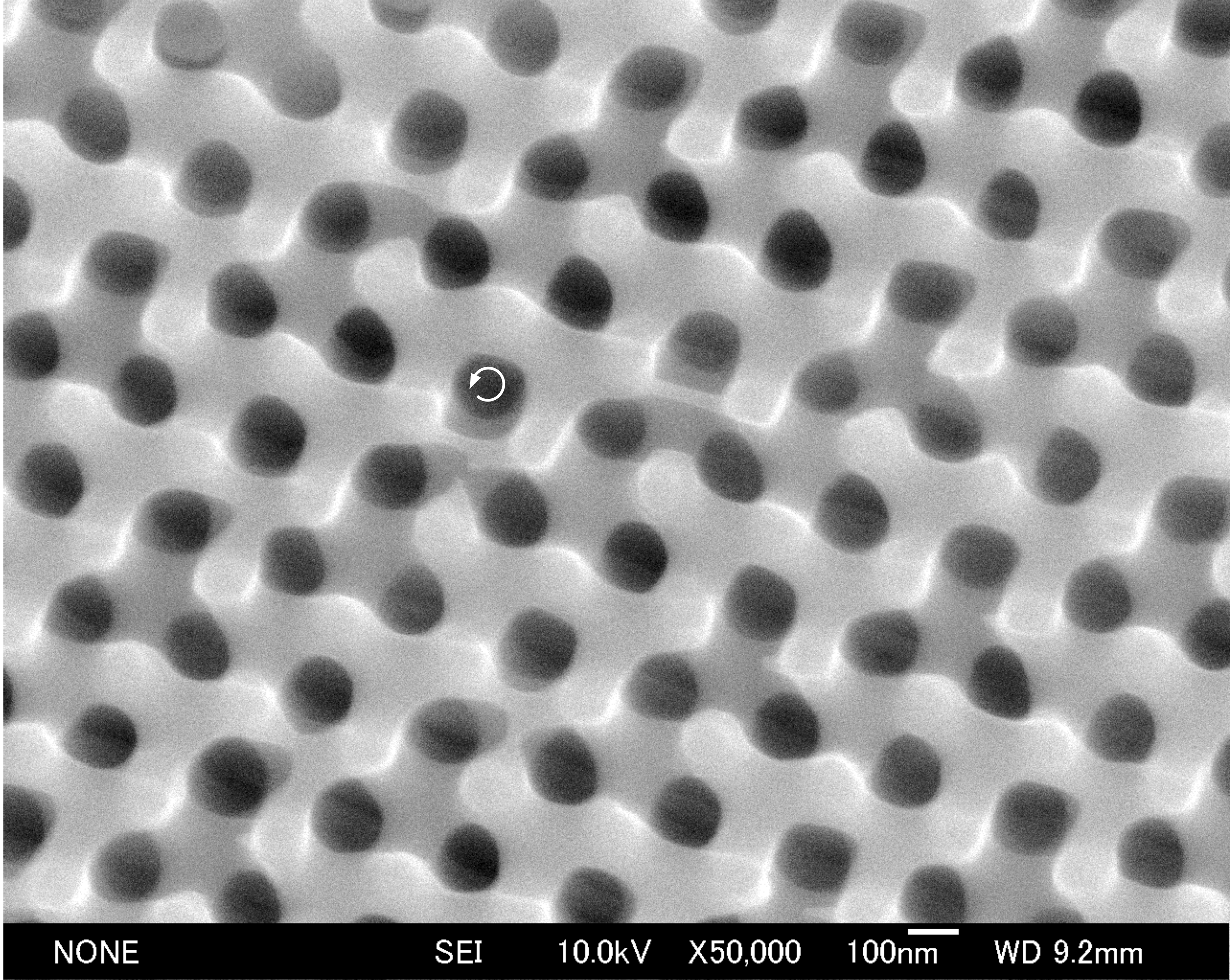

specimen No. 1
scale No. 9
domain No. 4
LH

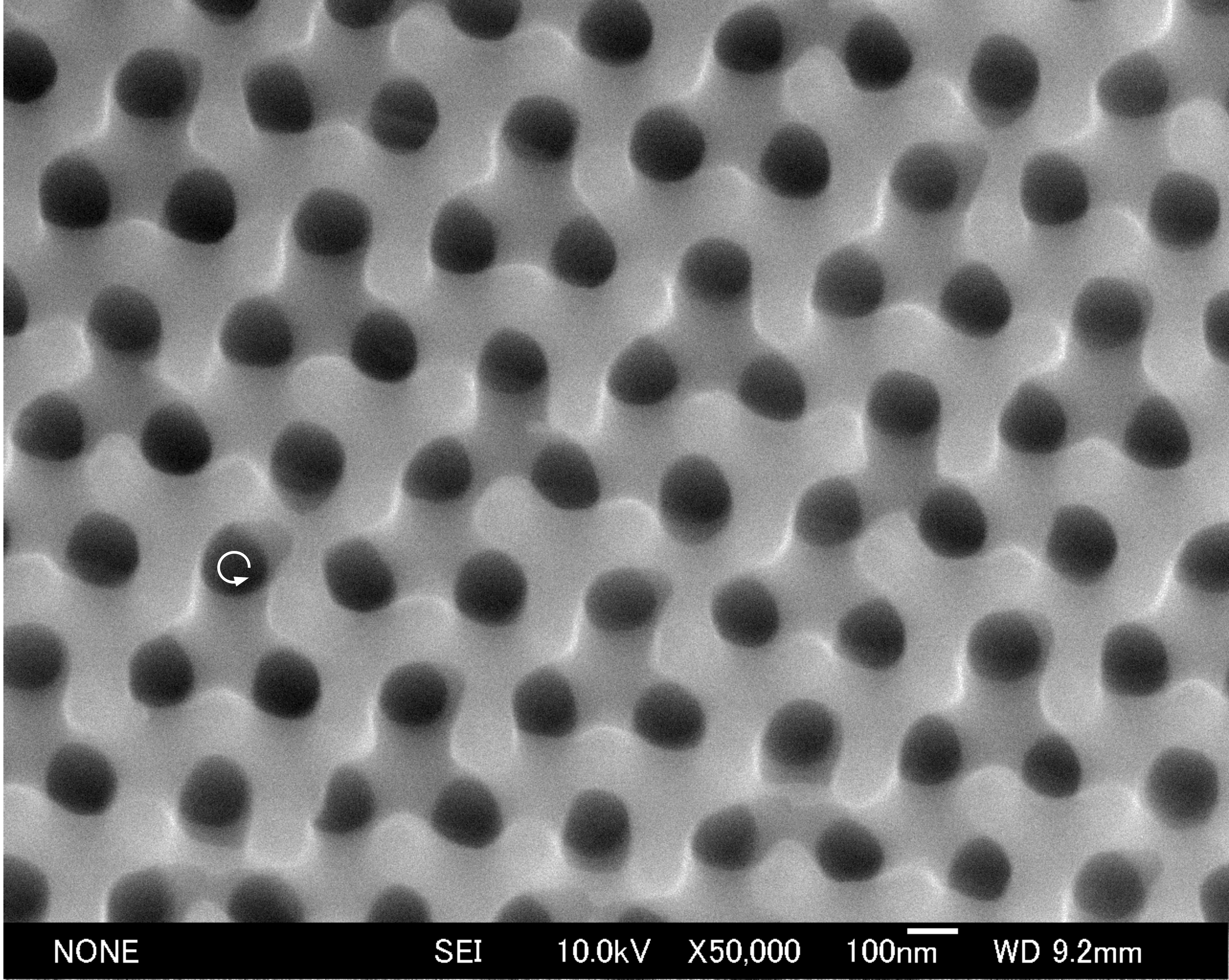

specimen No. 1
scale No. 9
domain No. 5
LH

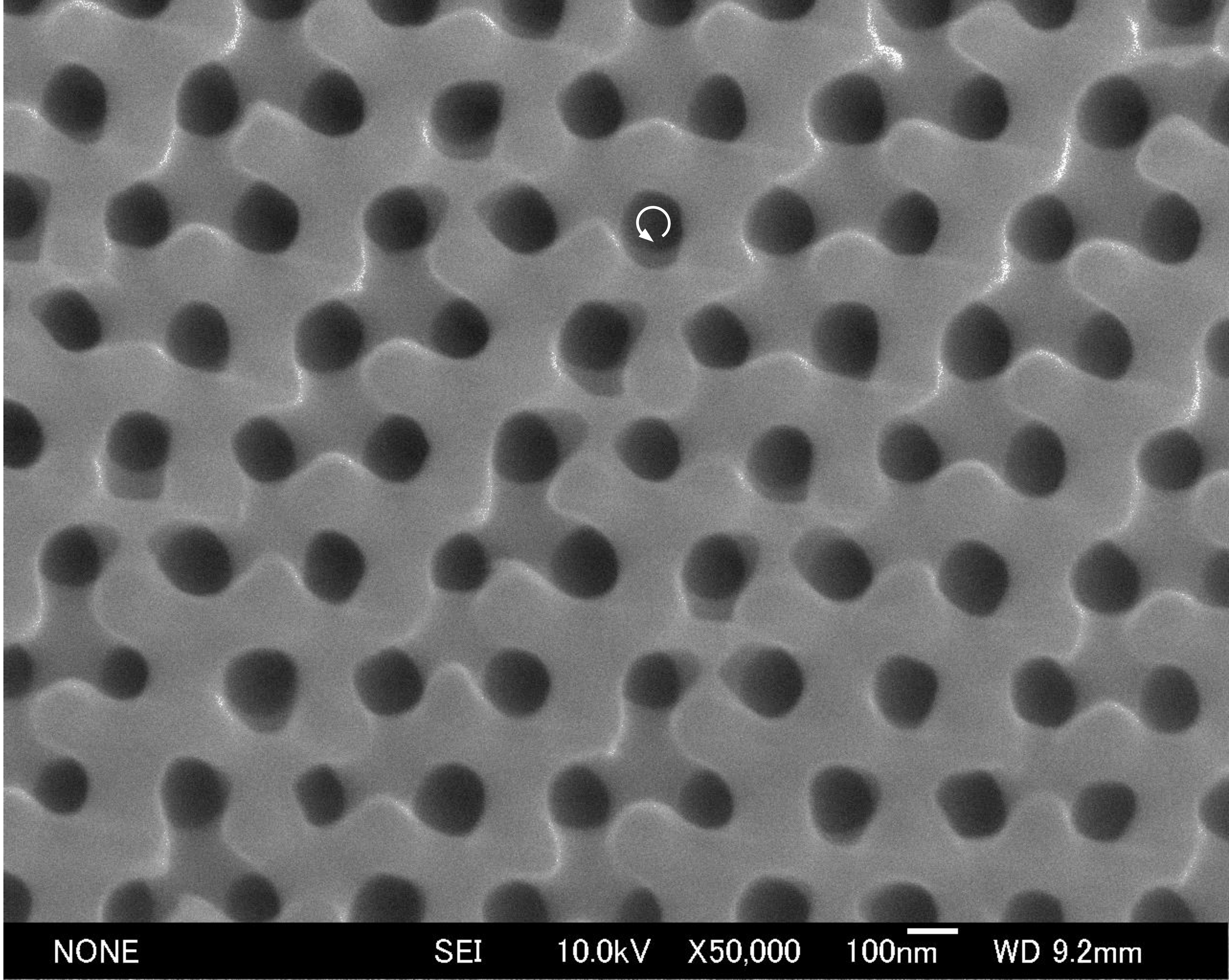

specimen No. 1
scale No. 9
domain No. 6
LH

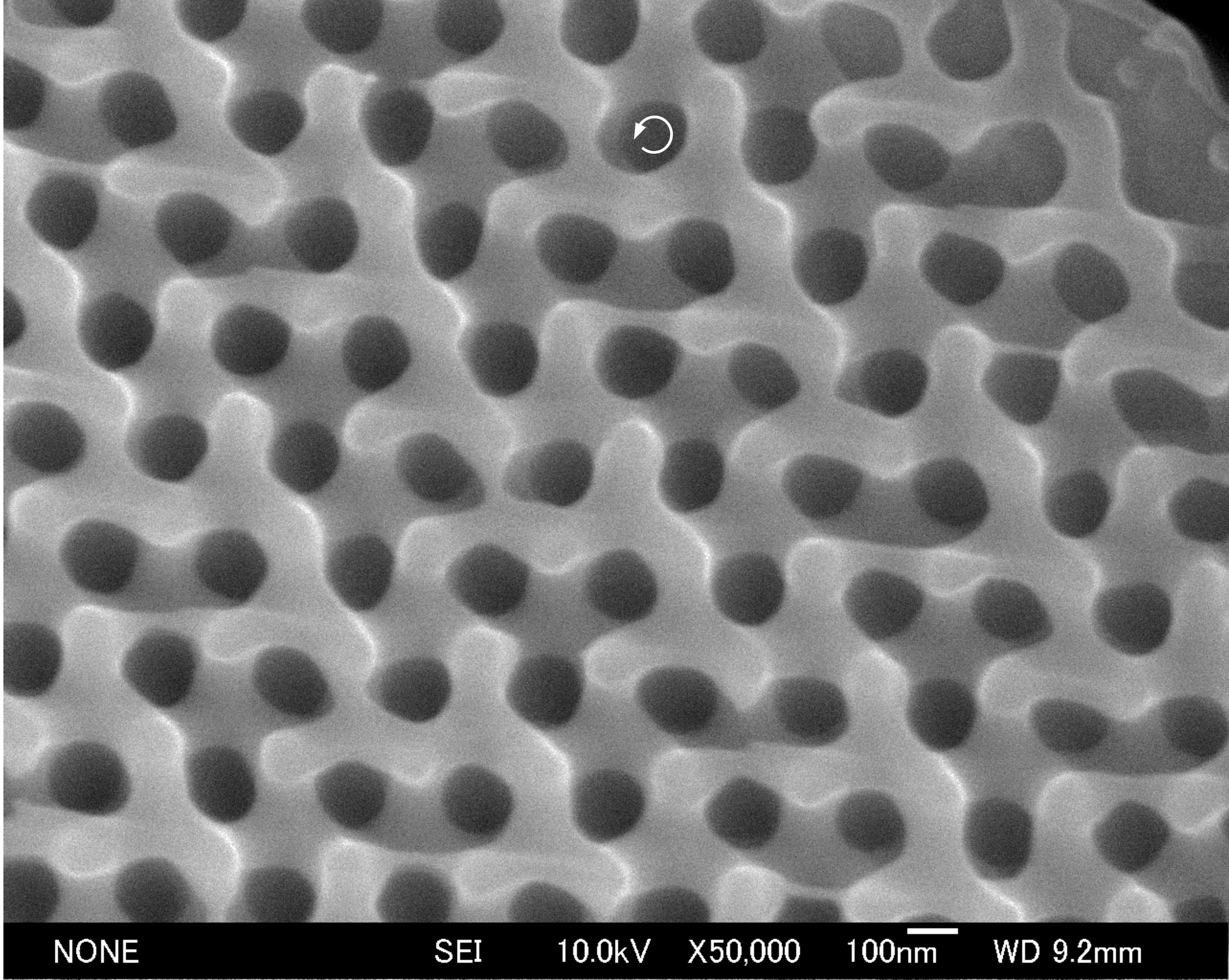

specimen No. 1
scale No. 9
domain No. 7
LH

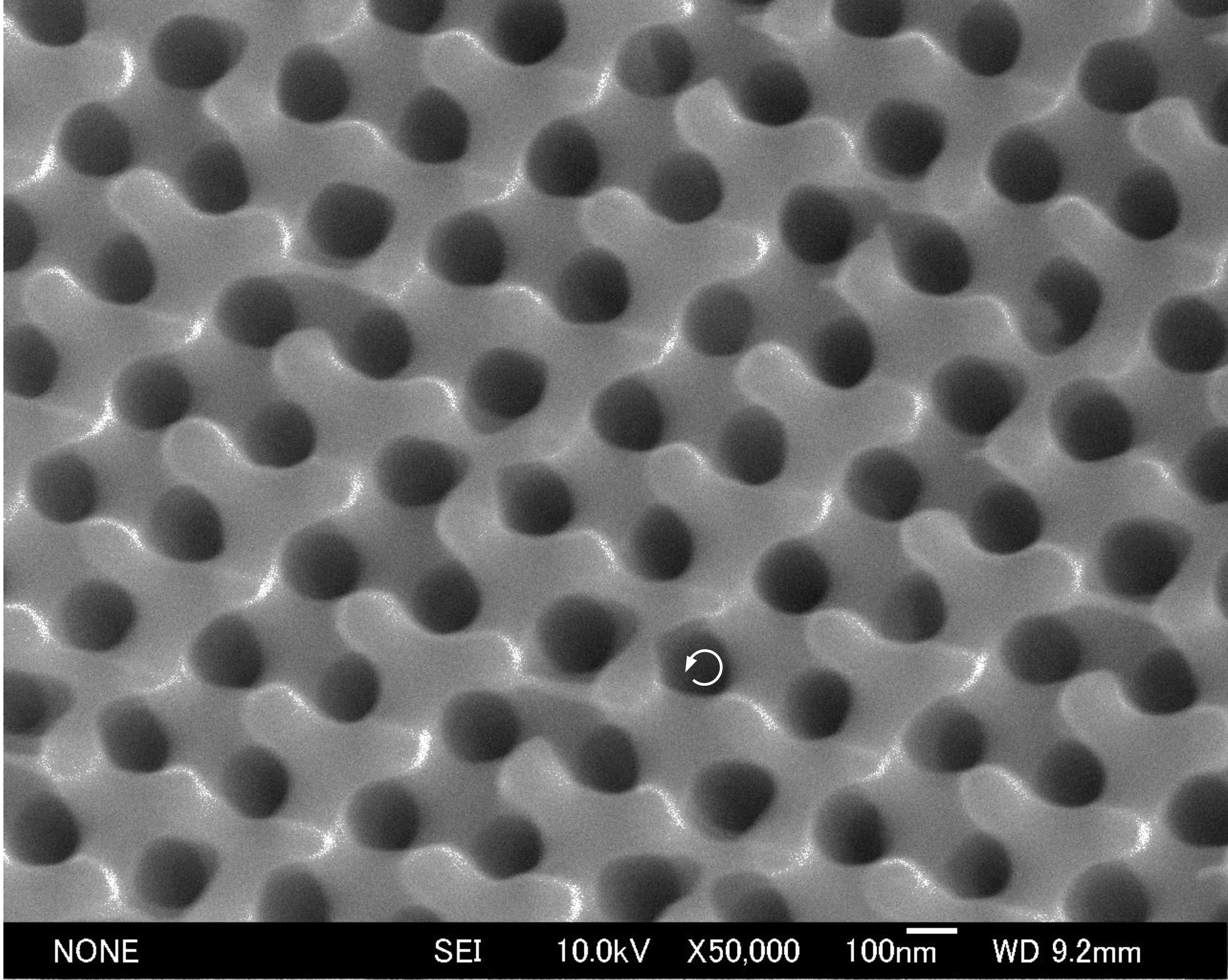

specimen No. 1
scale No. 9
domain No. 8
LH

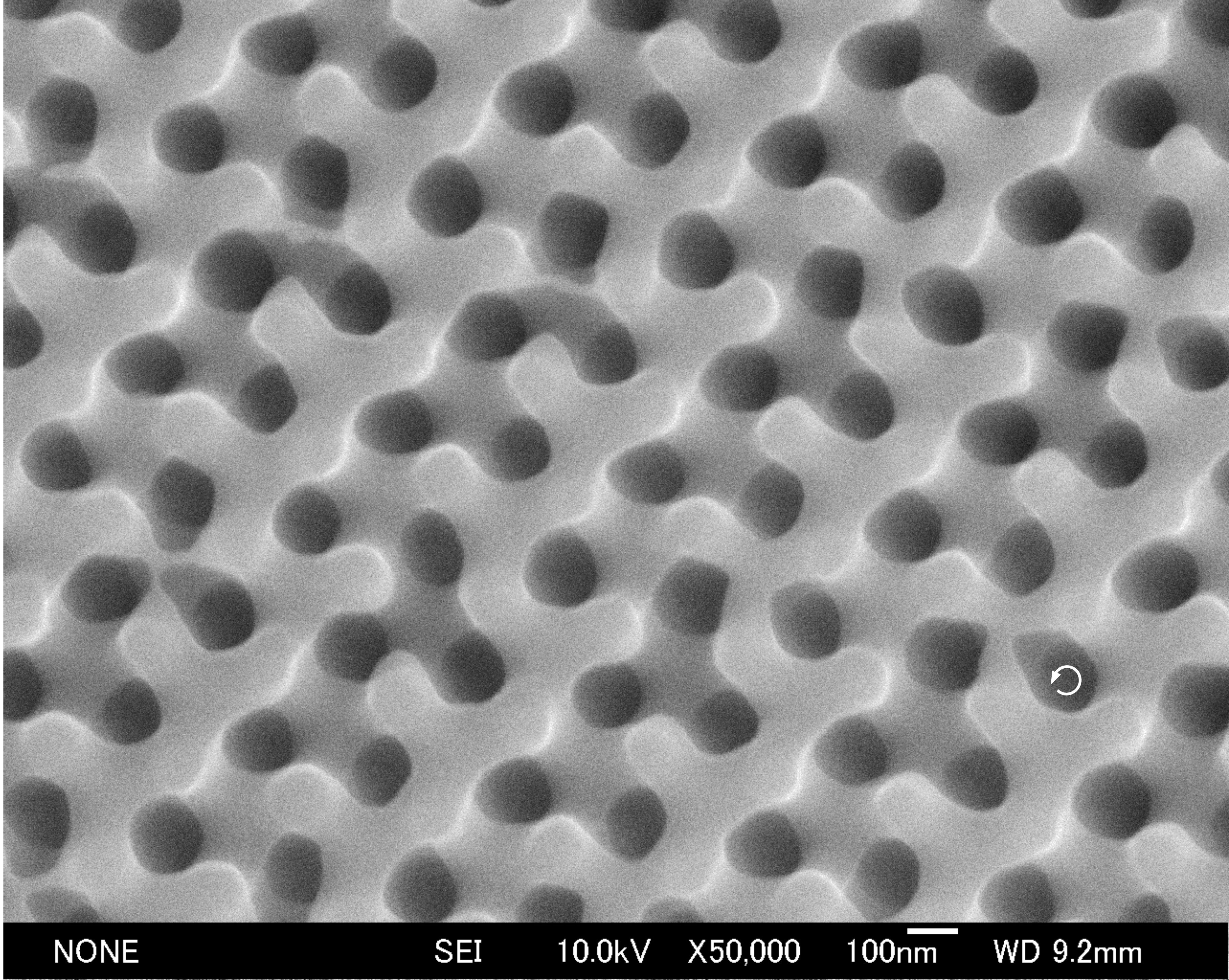

specimen No. 1
scale No. 9
domain No. 9
LH

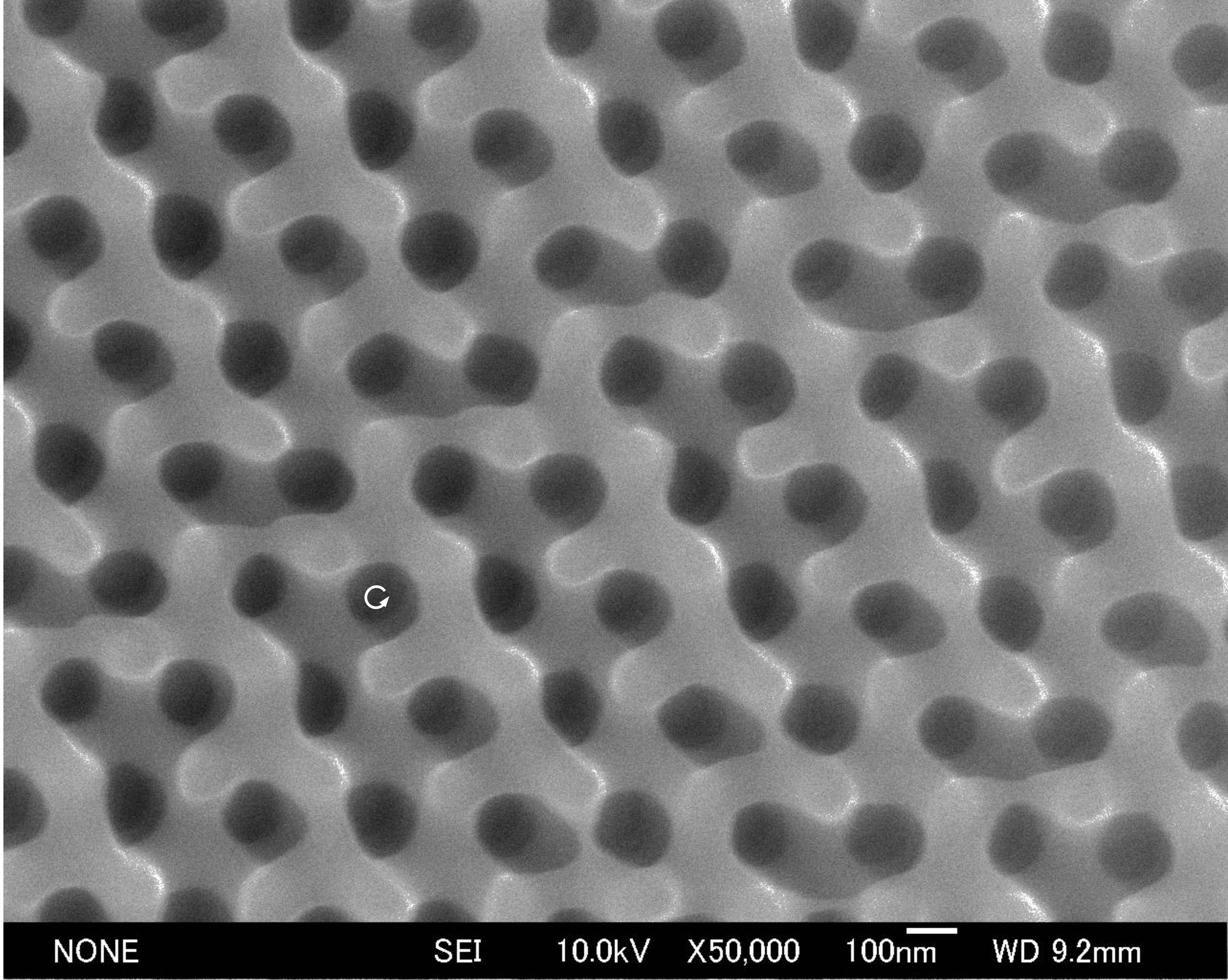

specimen No. 1
scale No. 9
domain No. 10
LH

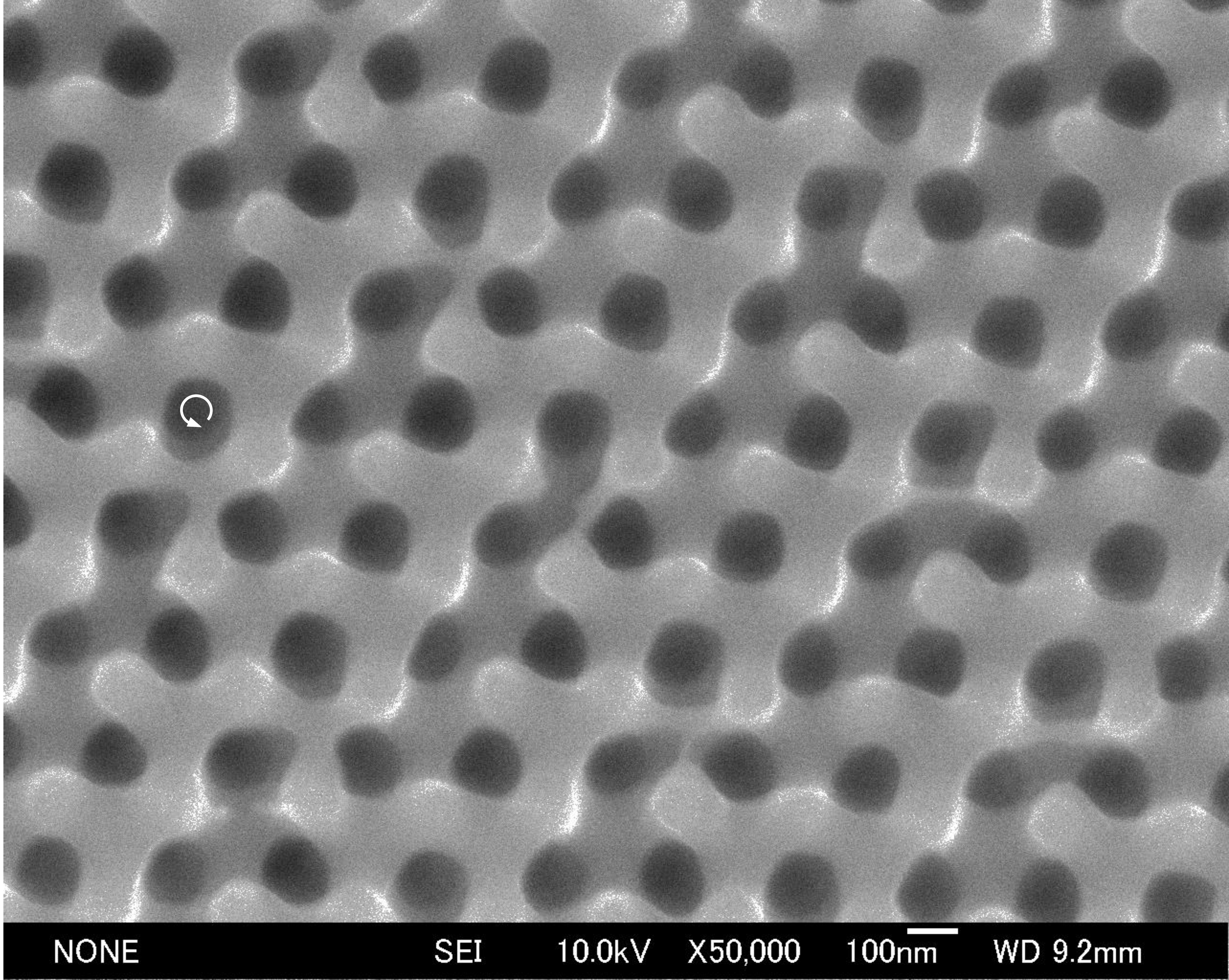

specimen No. 1
scale No. 9
domain No. 11
LH

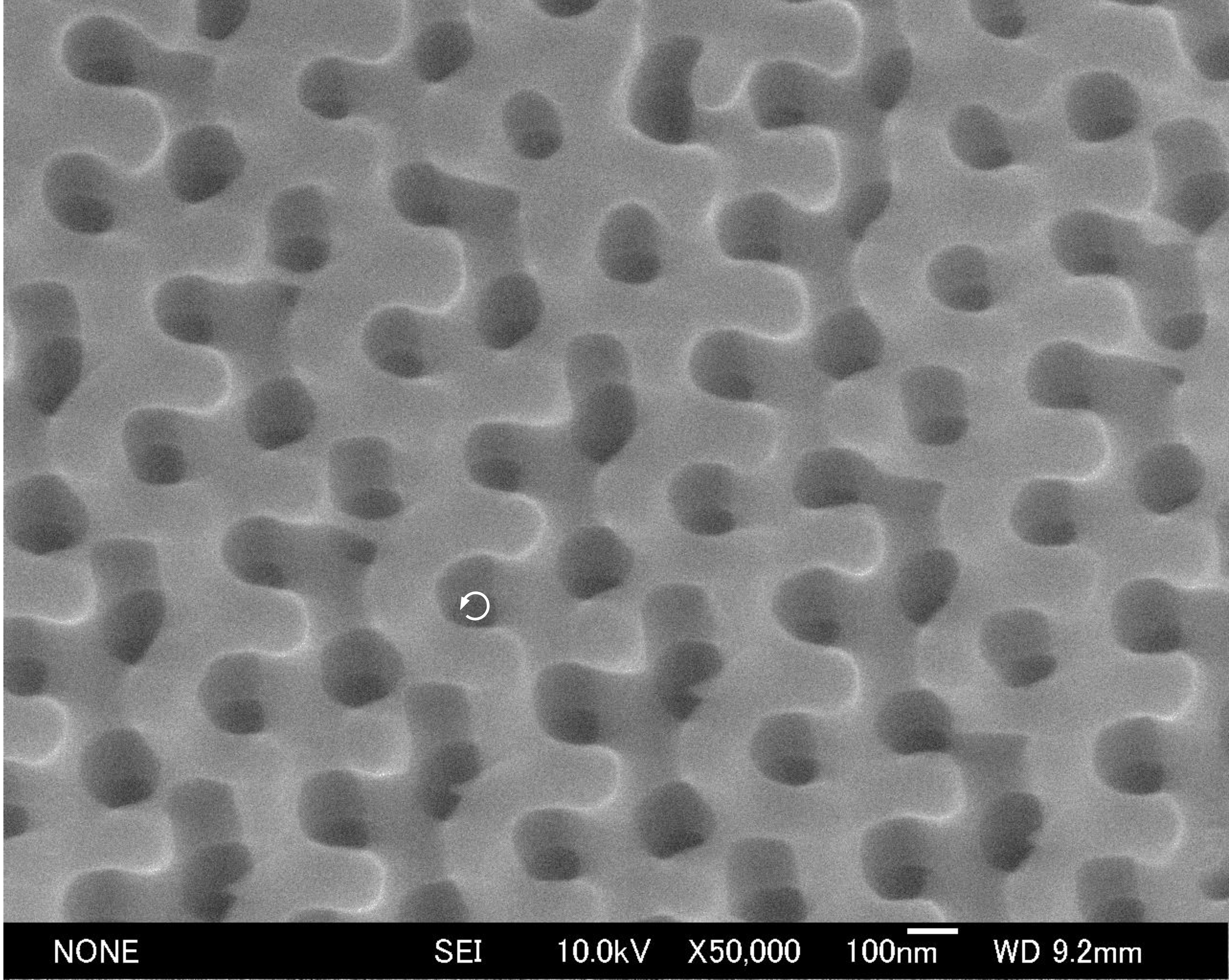

specimen No. 1
scale No. 10

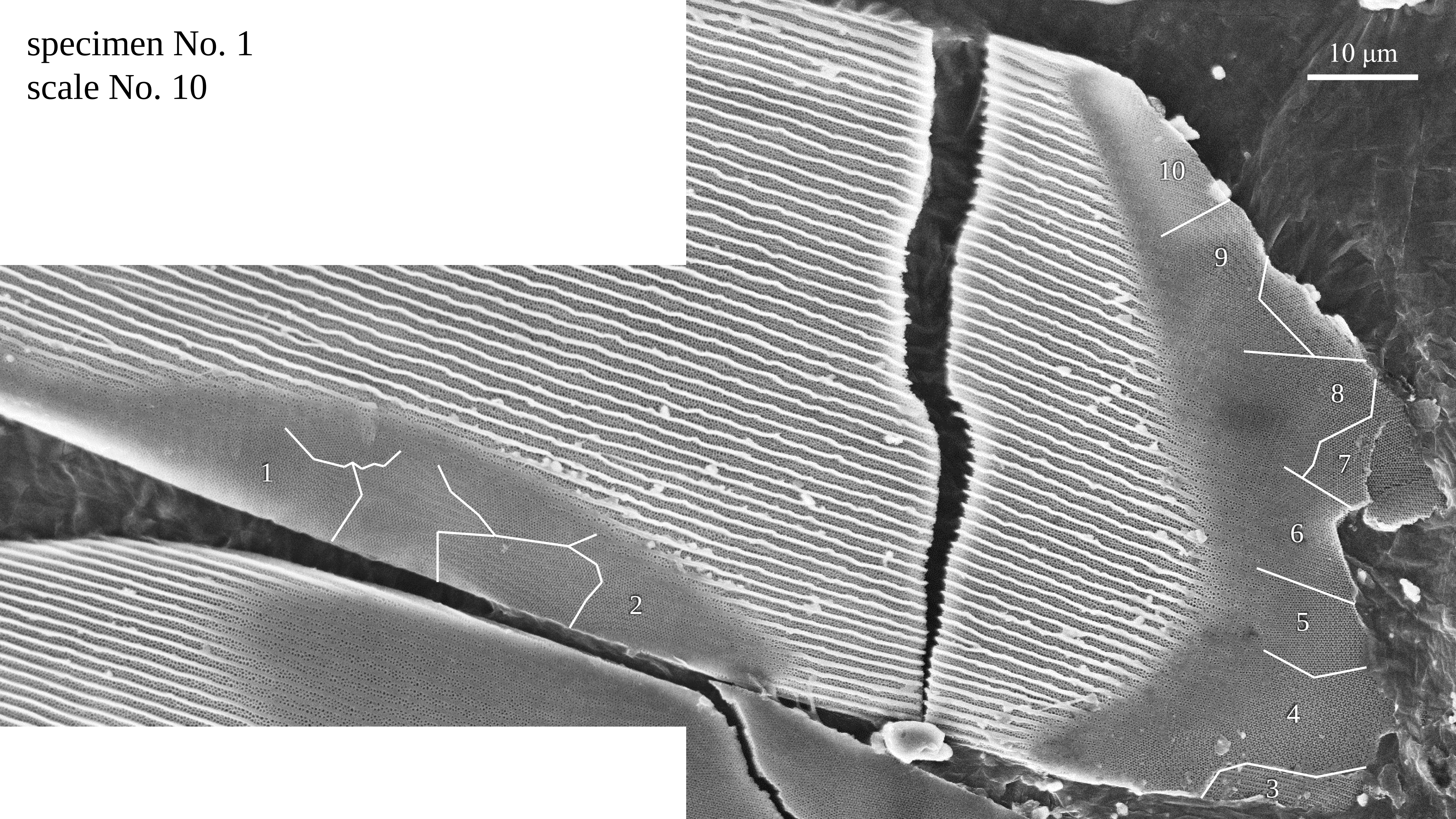

specimen No. 1
scale No. 10
domain No. 1
LH

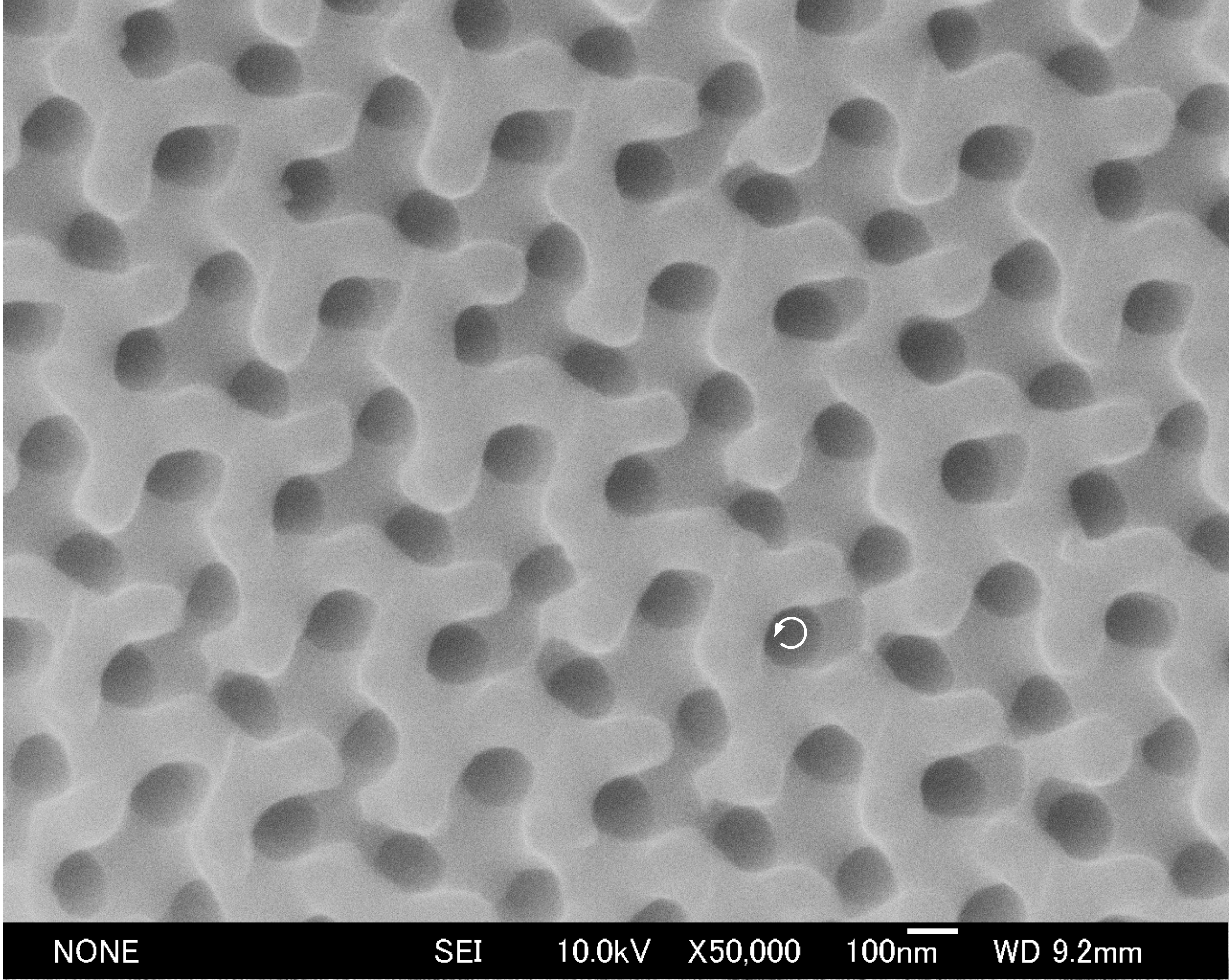

specimen No. 1
scale No. 10
domain No. 2
LH

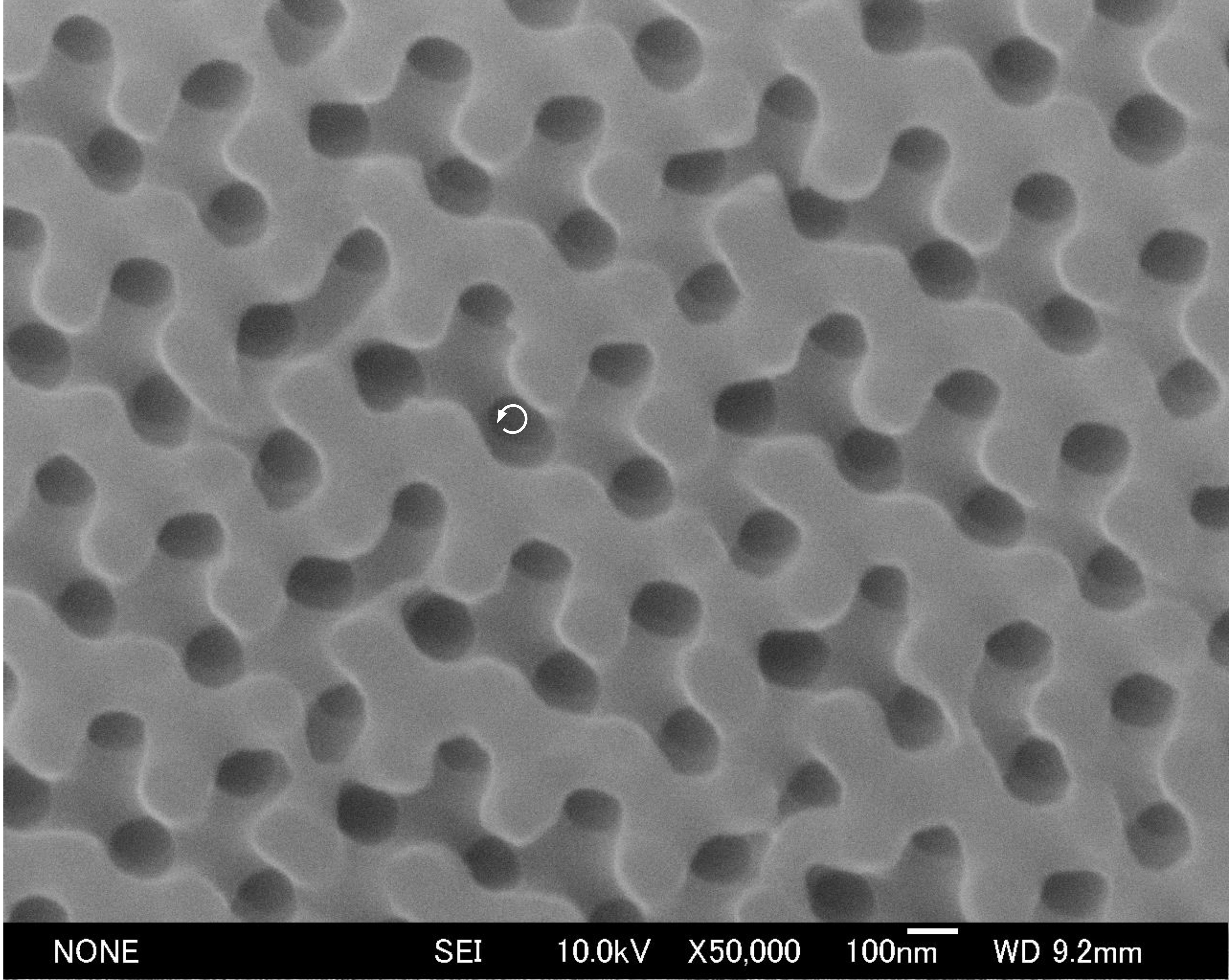

specimen No. 1
scale No. 10
domain No. 3
LH

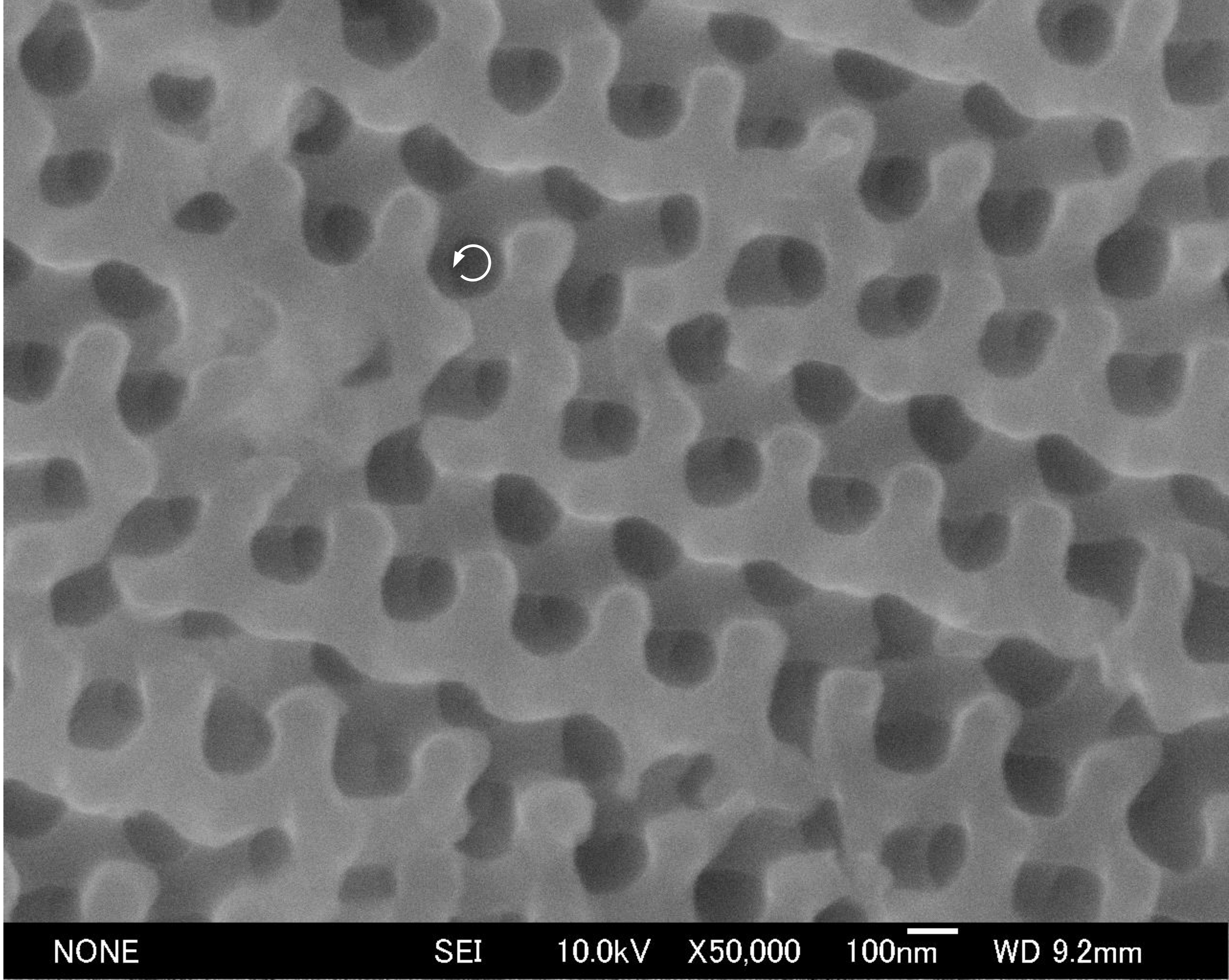

specimen No. 1
scale No. 10
domain No. 4
LH

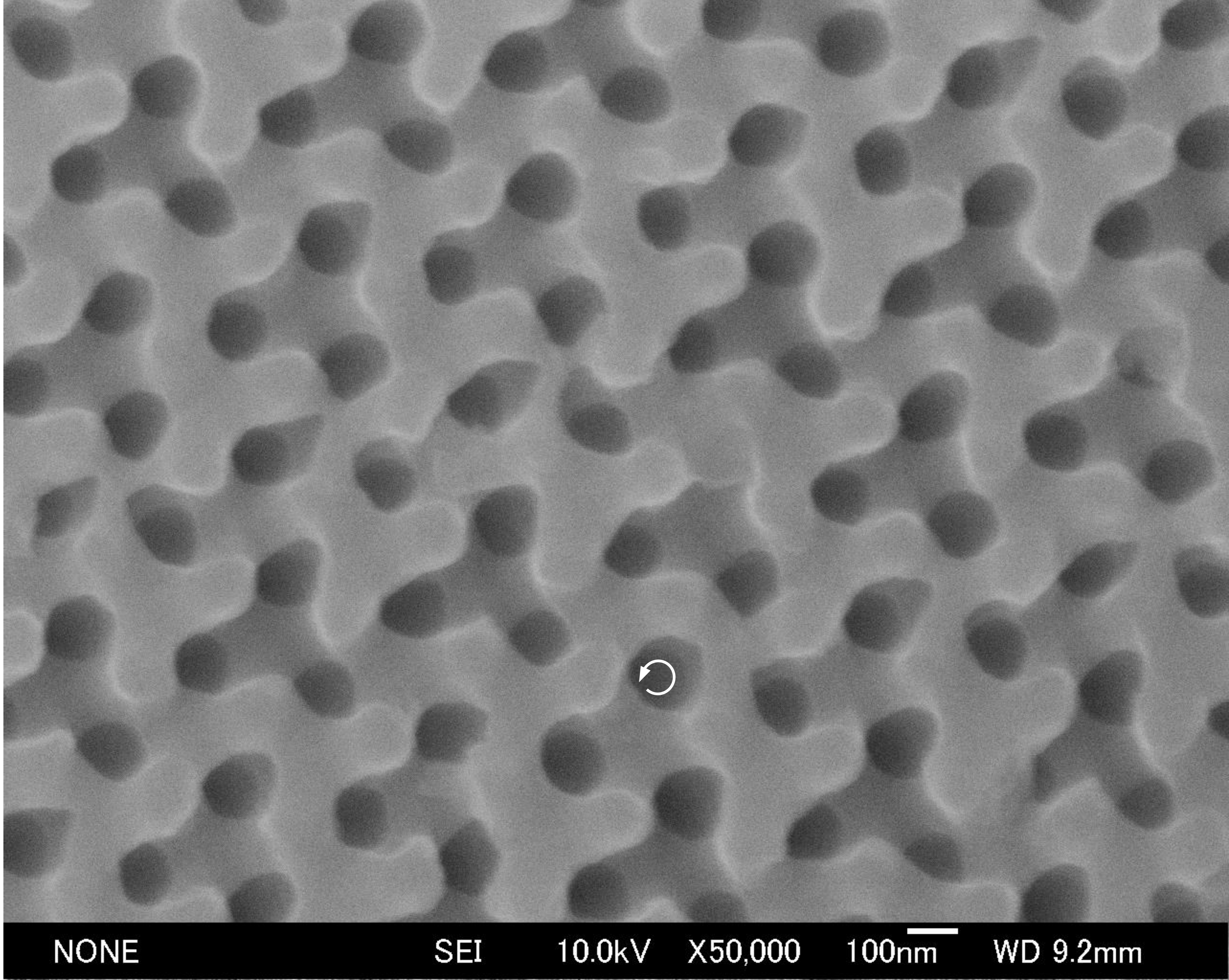

specimen No. 1
scale No. 10
domain No. 5
LH

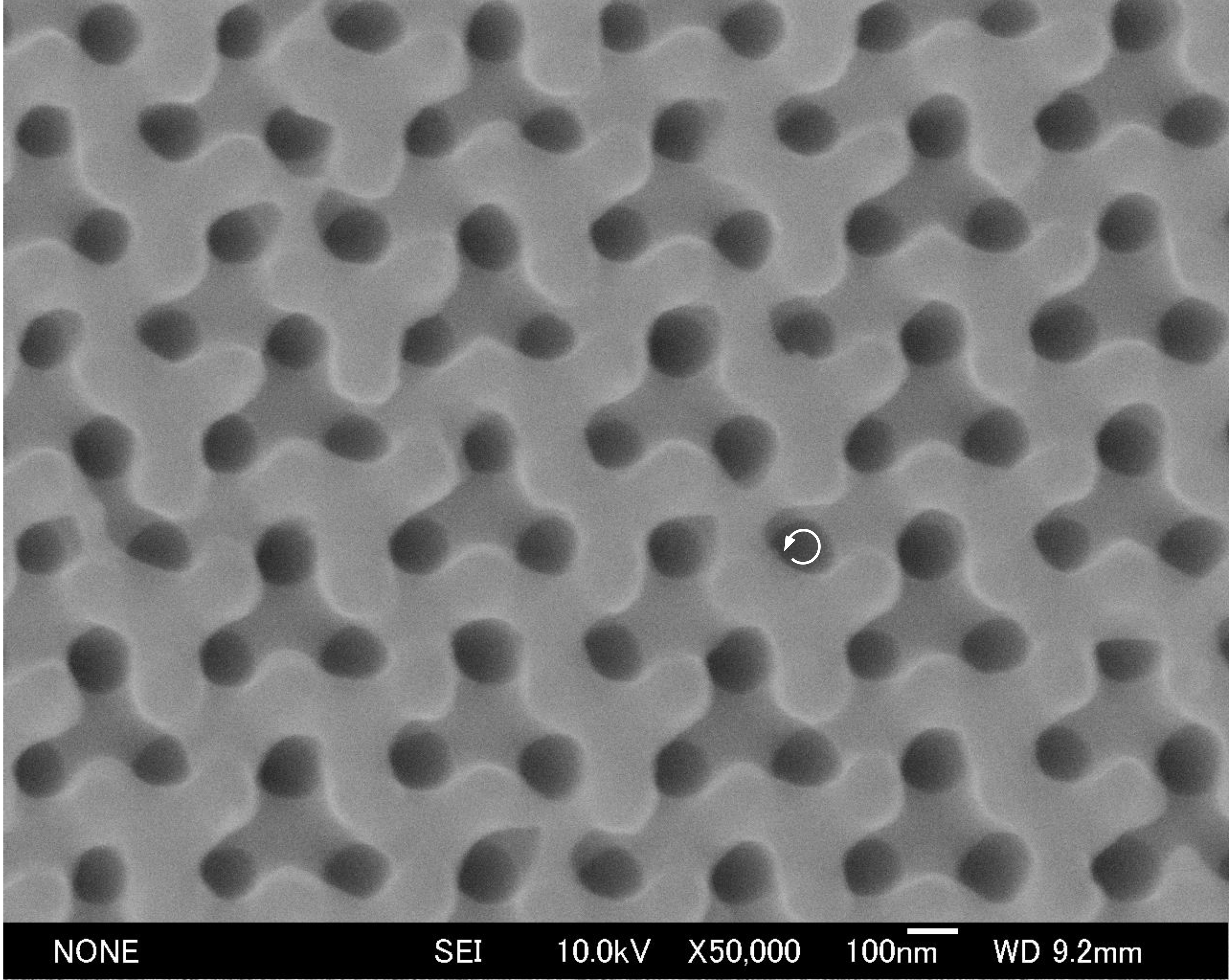

specimen No. 1
scale No. 10
domain No. 6
LH

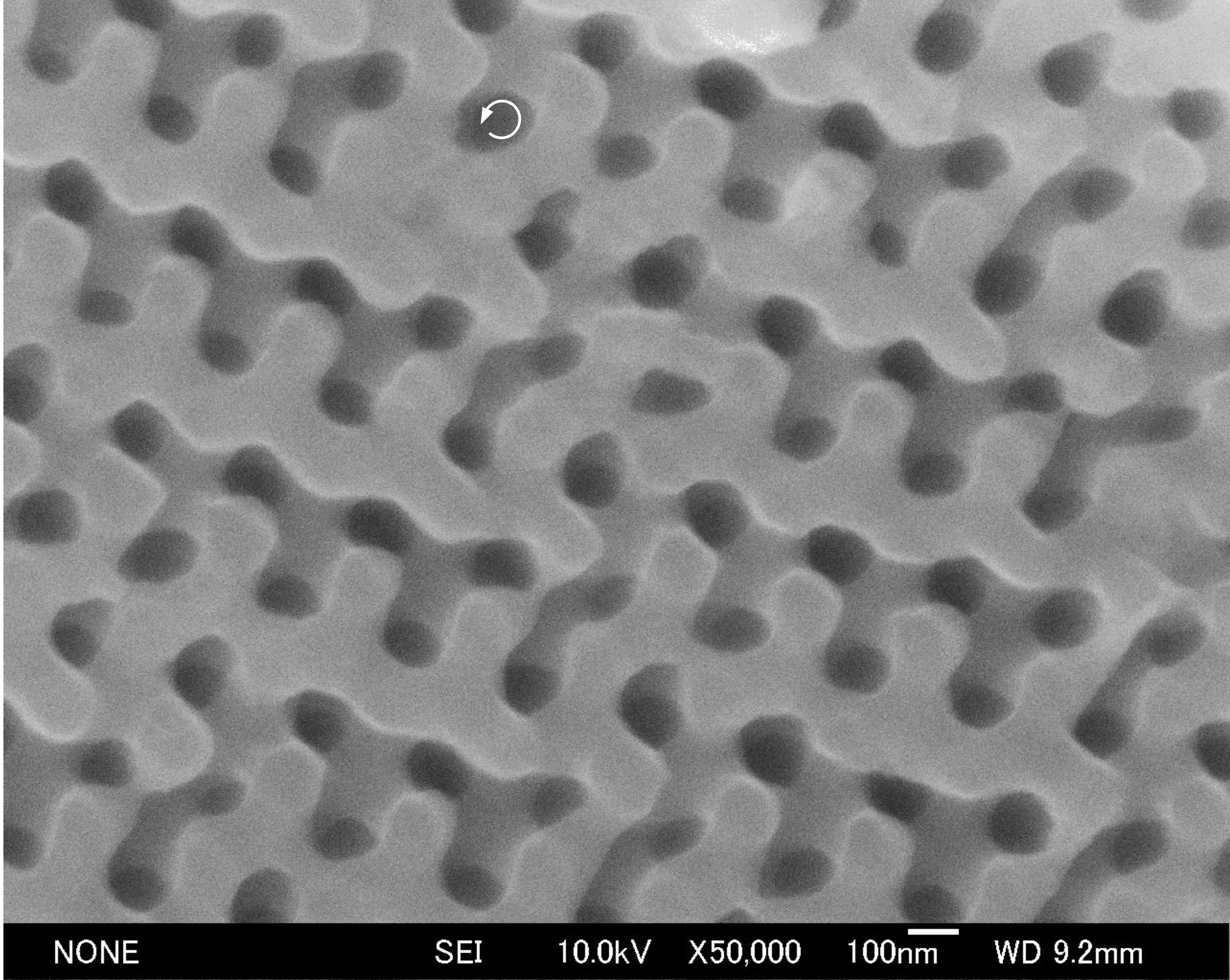

specimen No. 1
scale No. 10
domain No. 7
LH

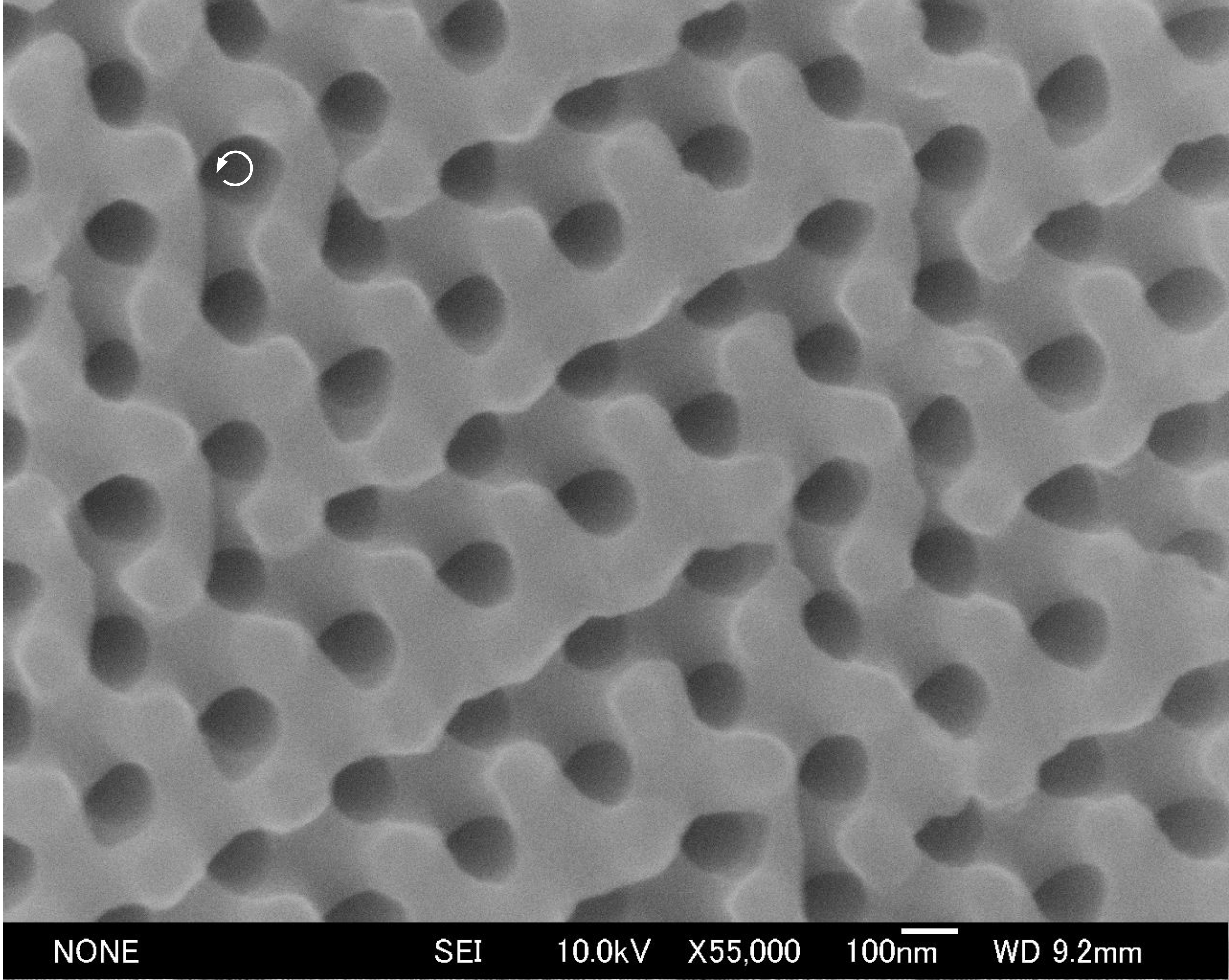

specimen No. 1
scale No. 10
domain No. 8
LH

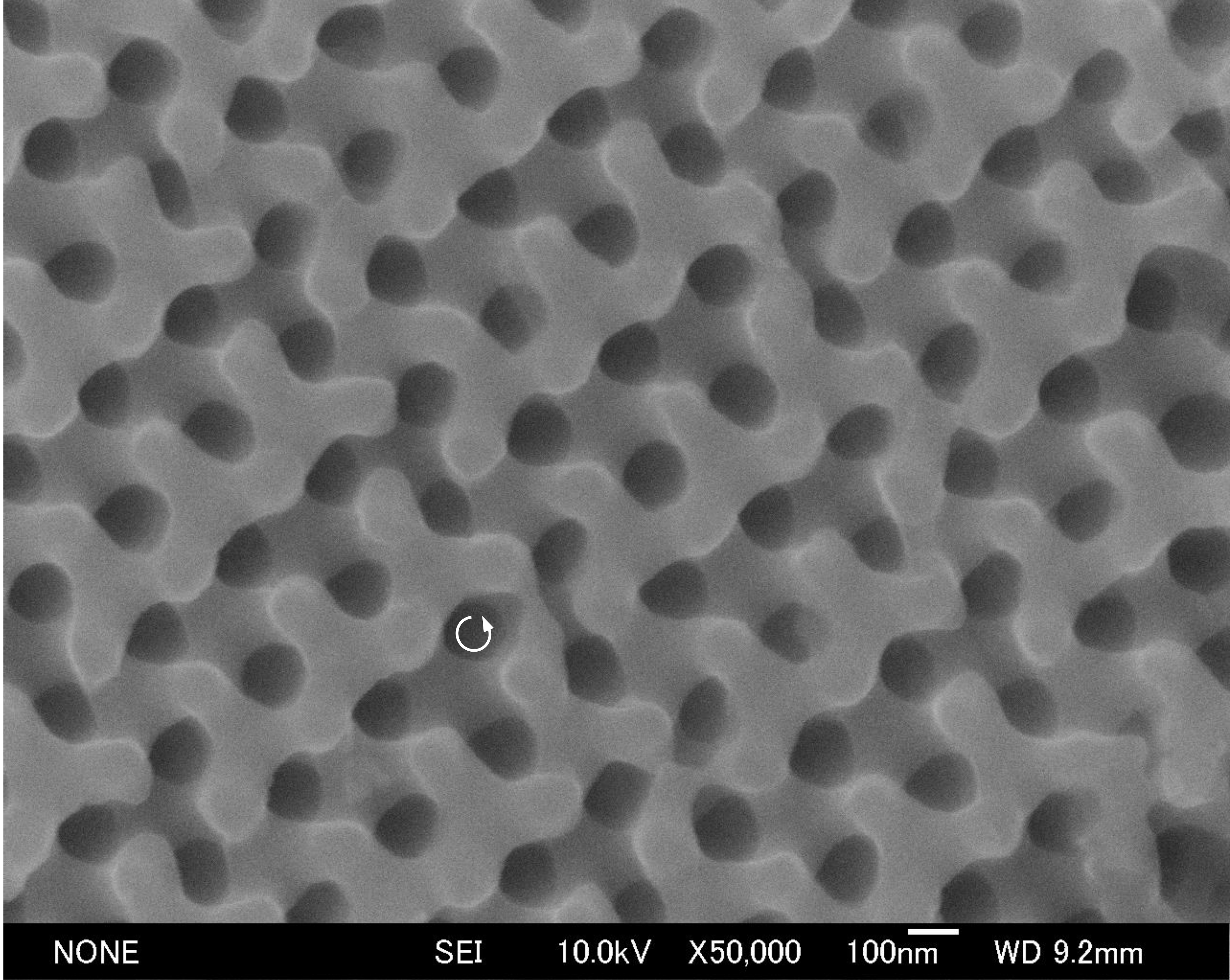

specimen No. 1
scale No. 10
domain No. 9
LH

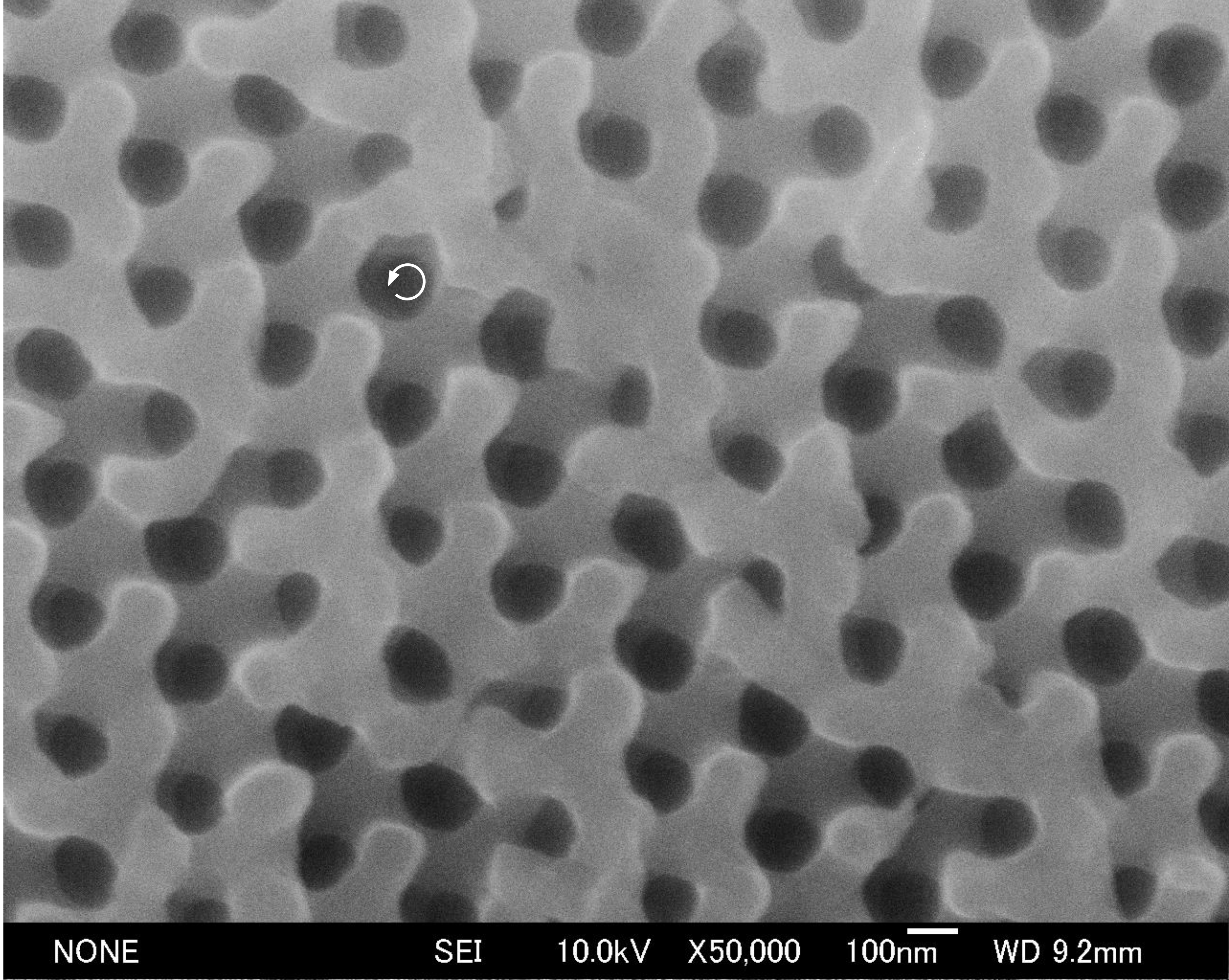

specimen No. 1
scale No. 10
domain No. 10
LH

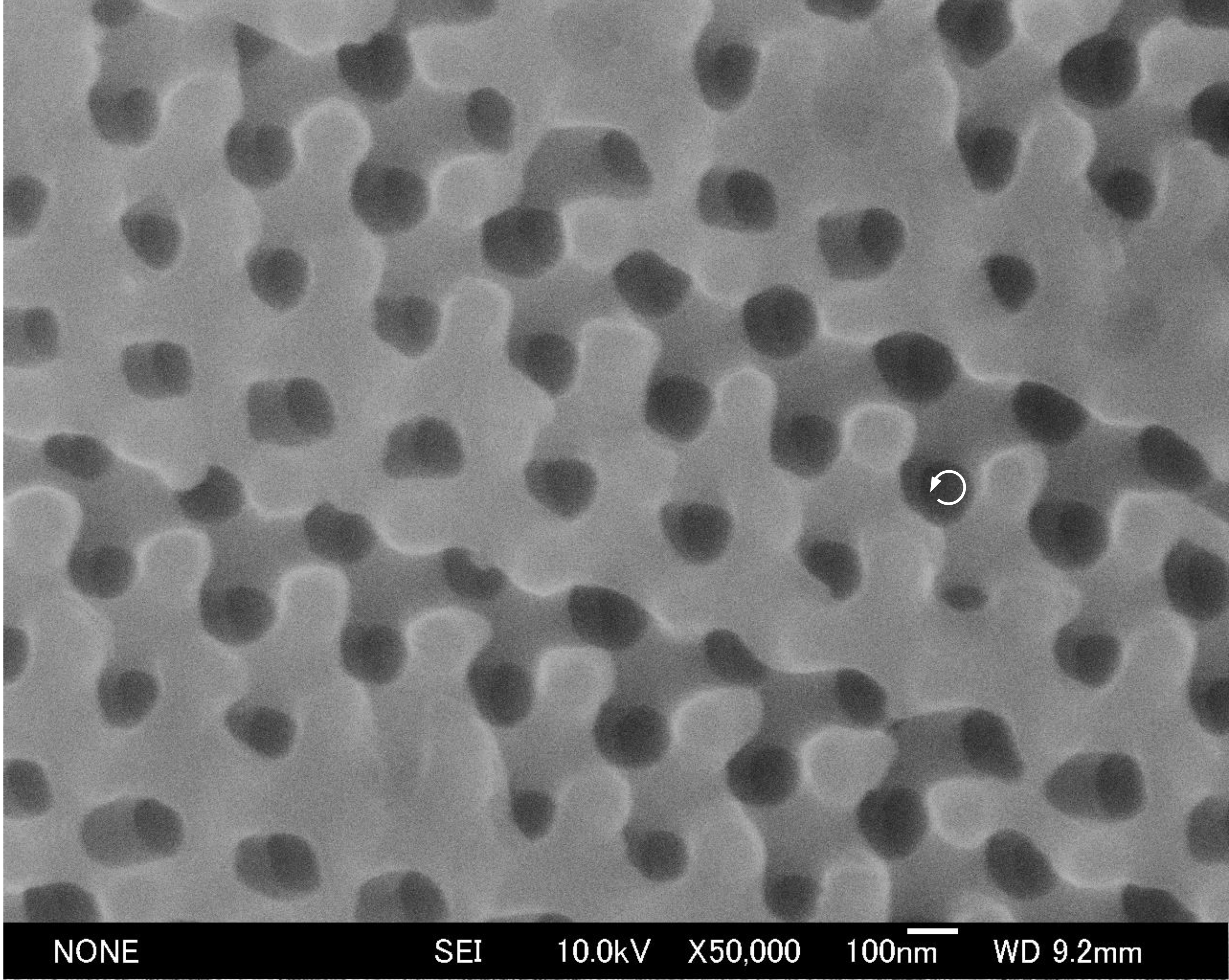

Supplement: Supplementary file 3 — Supplementary Information 3. [file 41598_2025_5750_MOESM3_ESM.pdf]
