# Supplementary material for: Study on the chirality of gyroid photonic crystals in butterfly wing scales
Source: Sci Rep. 2025 Jul 1;15:20968. doi: 10.1038/s41598-025-05750-2 (PMC12215952; doi:10.1038/s41598-025-05750-2)

specimen No. 1
scale No. 1

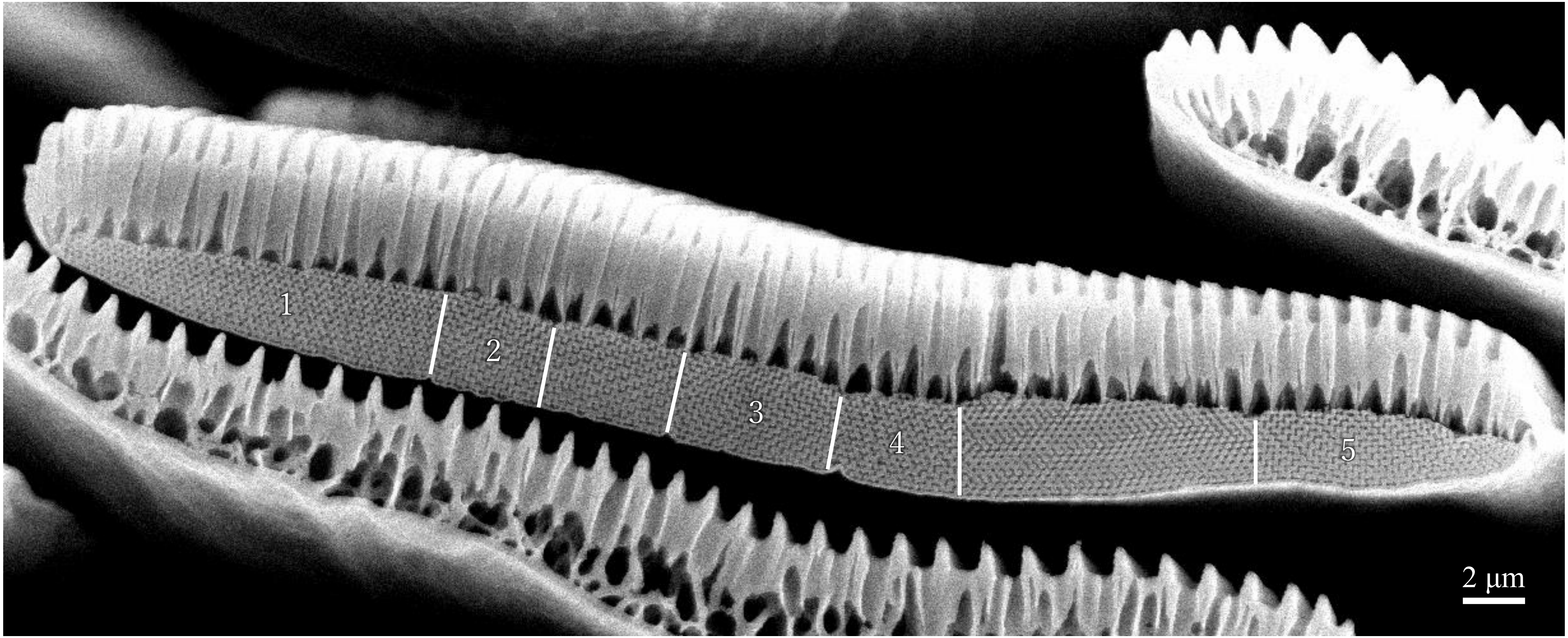

specimen No. 1
scale No. 1
domain No. 1
[111] lh spiral
LH gyroid

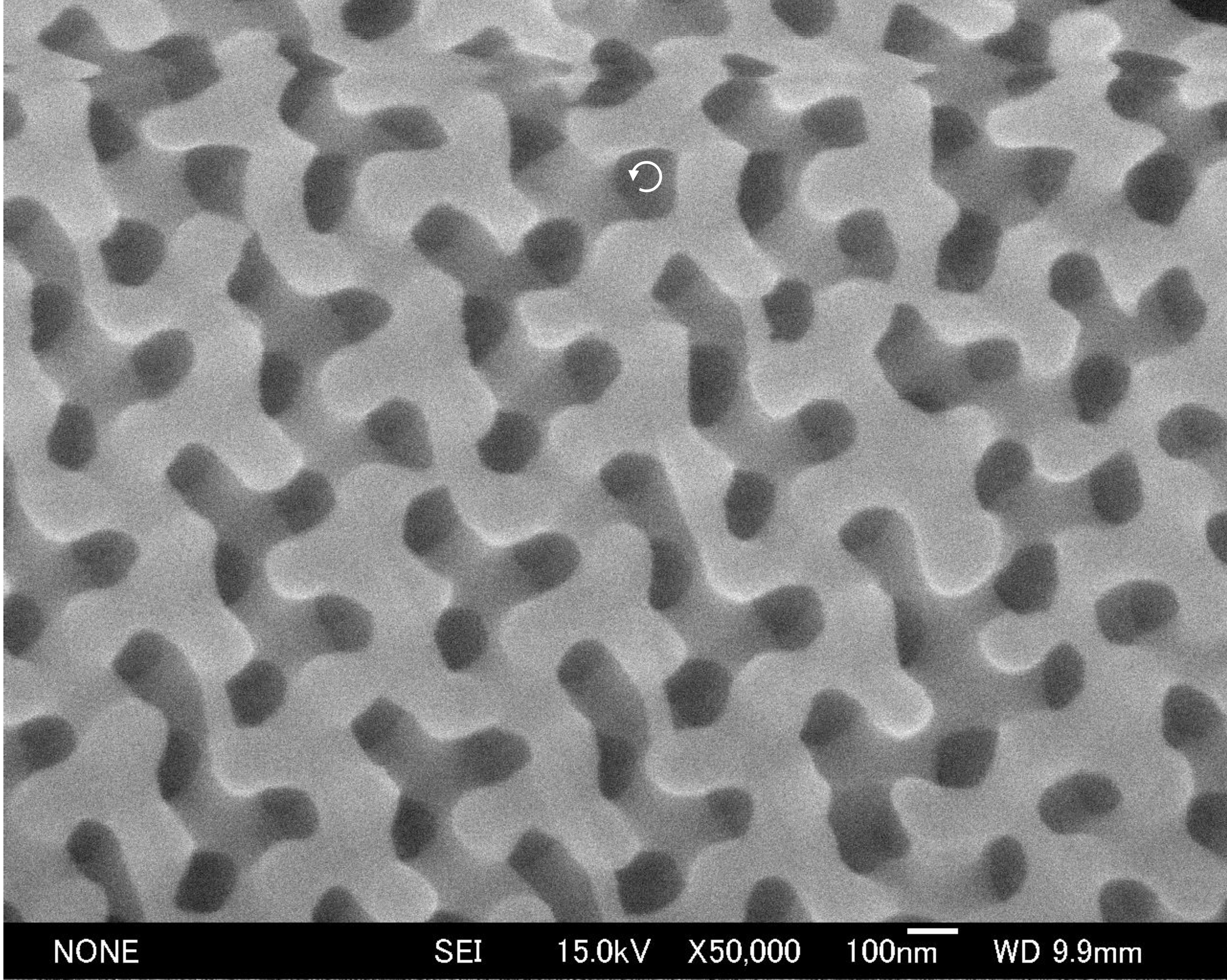

specimen No. 1
scale No. 1
domain No. 2
[100] rh spiral
LH gyroid

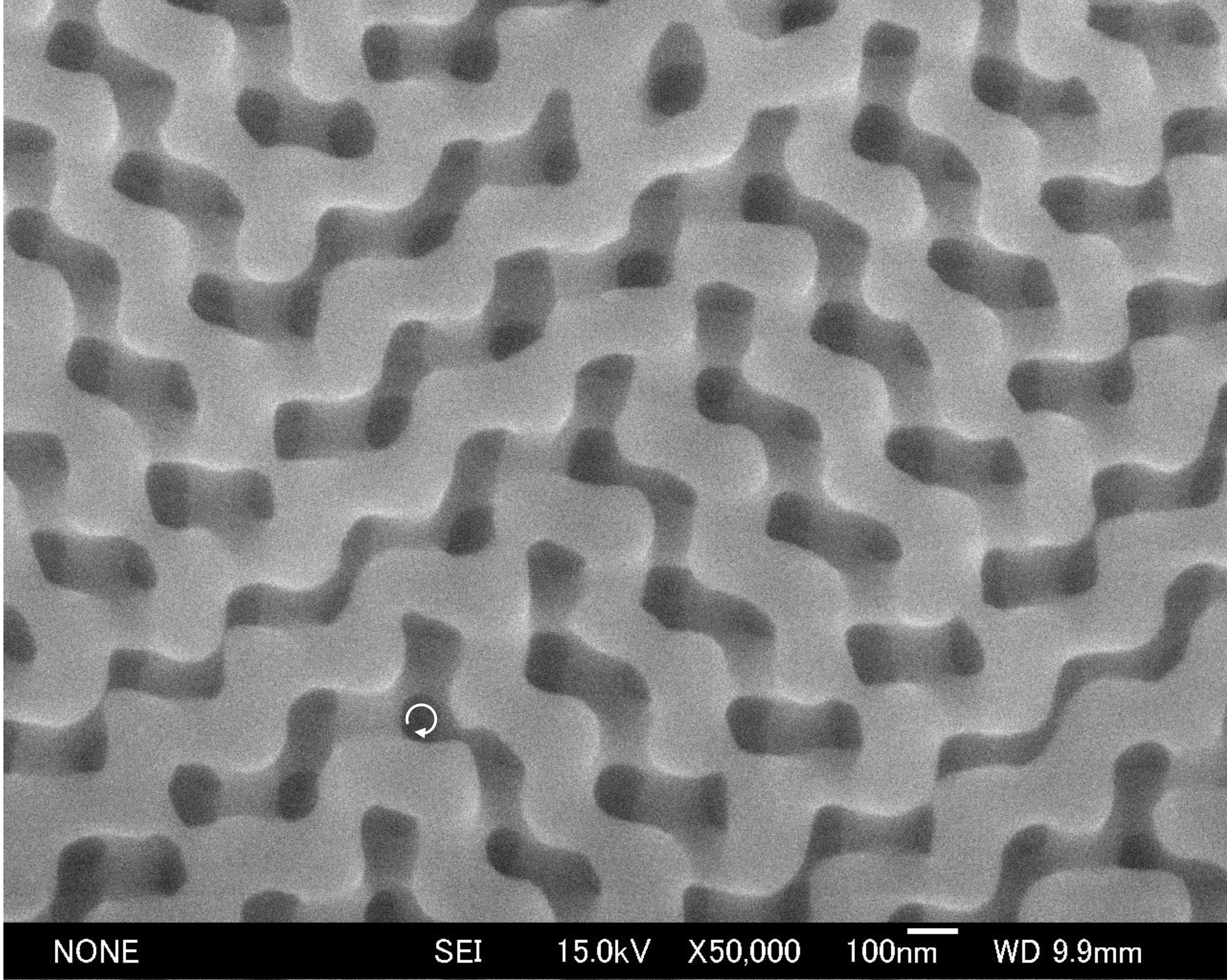

specimen No. 1
scale No. 1
domain No. 3
[100] rh spiral
LH gyroid

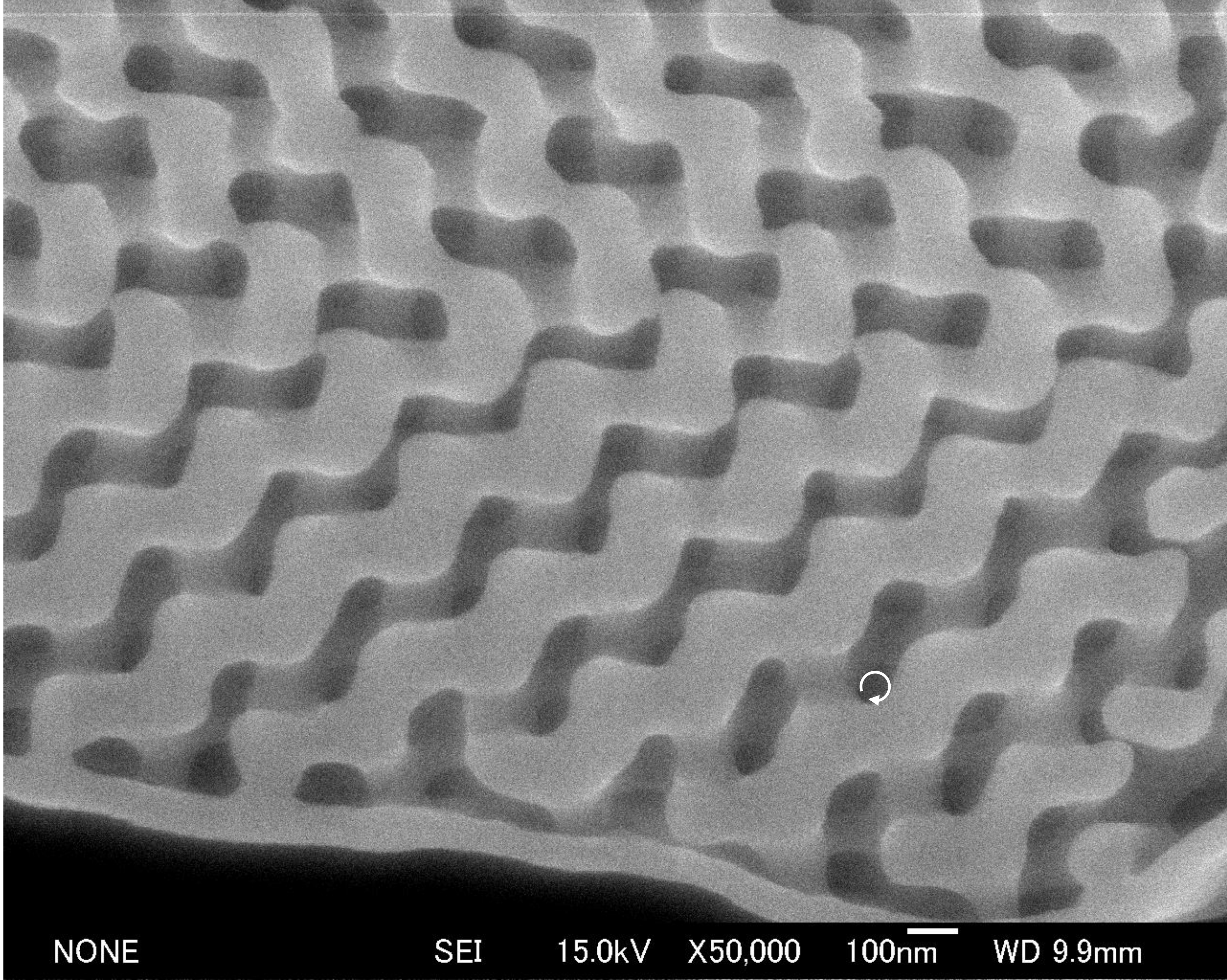

specimen No. 1
scale No. 1
domain No. 4
[111] rh spiral
RH gyroid

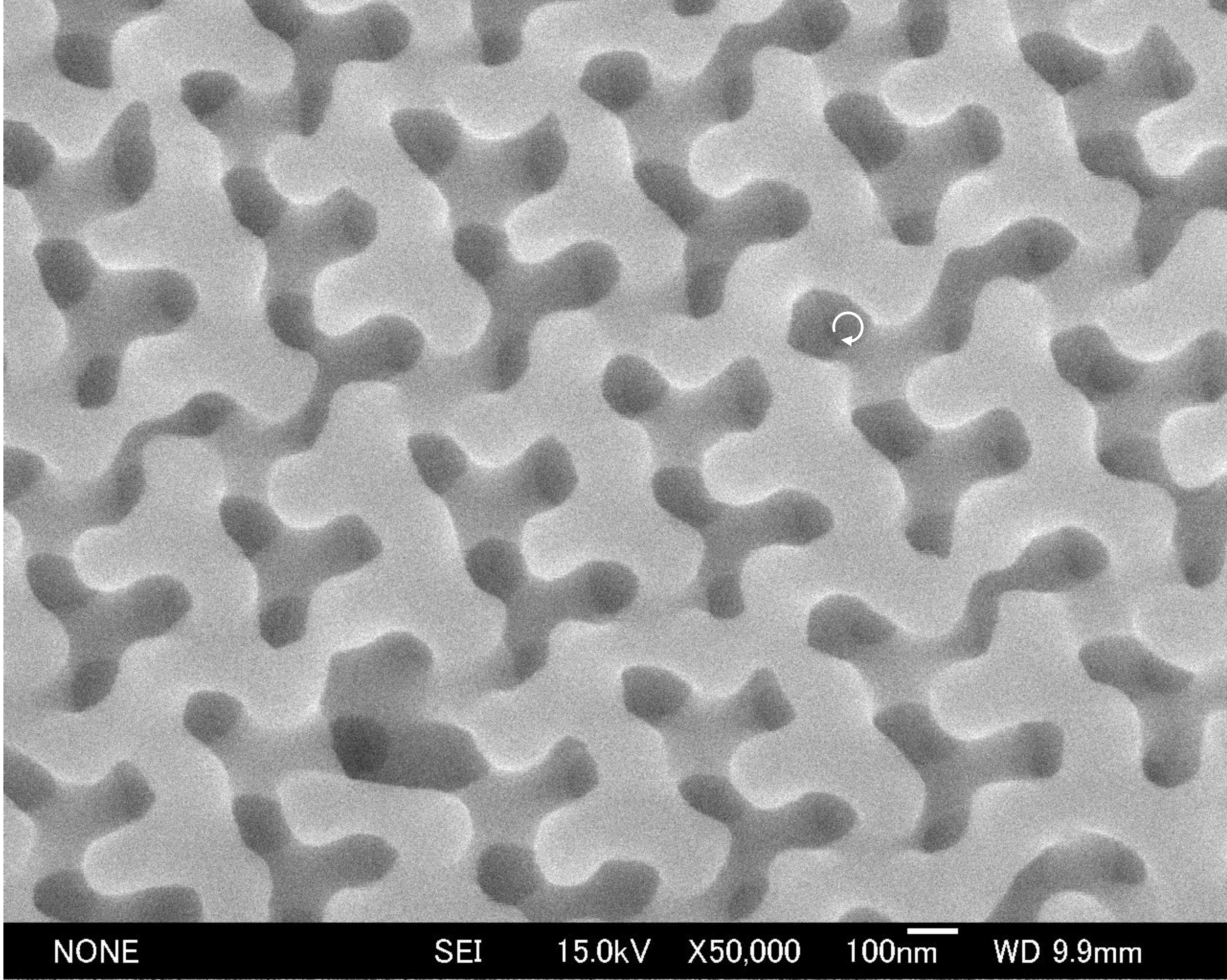

specimen No. 1
scale No. 1
domain No. 5
[100] rh spiral
LH gyroid

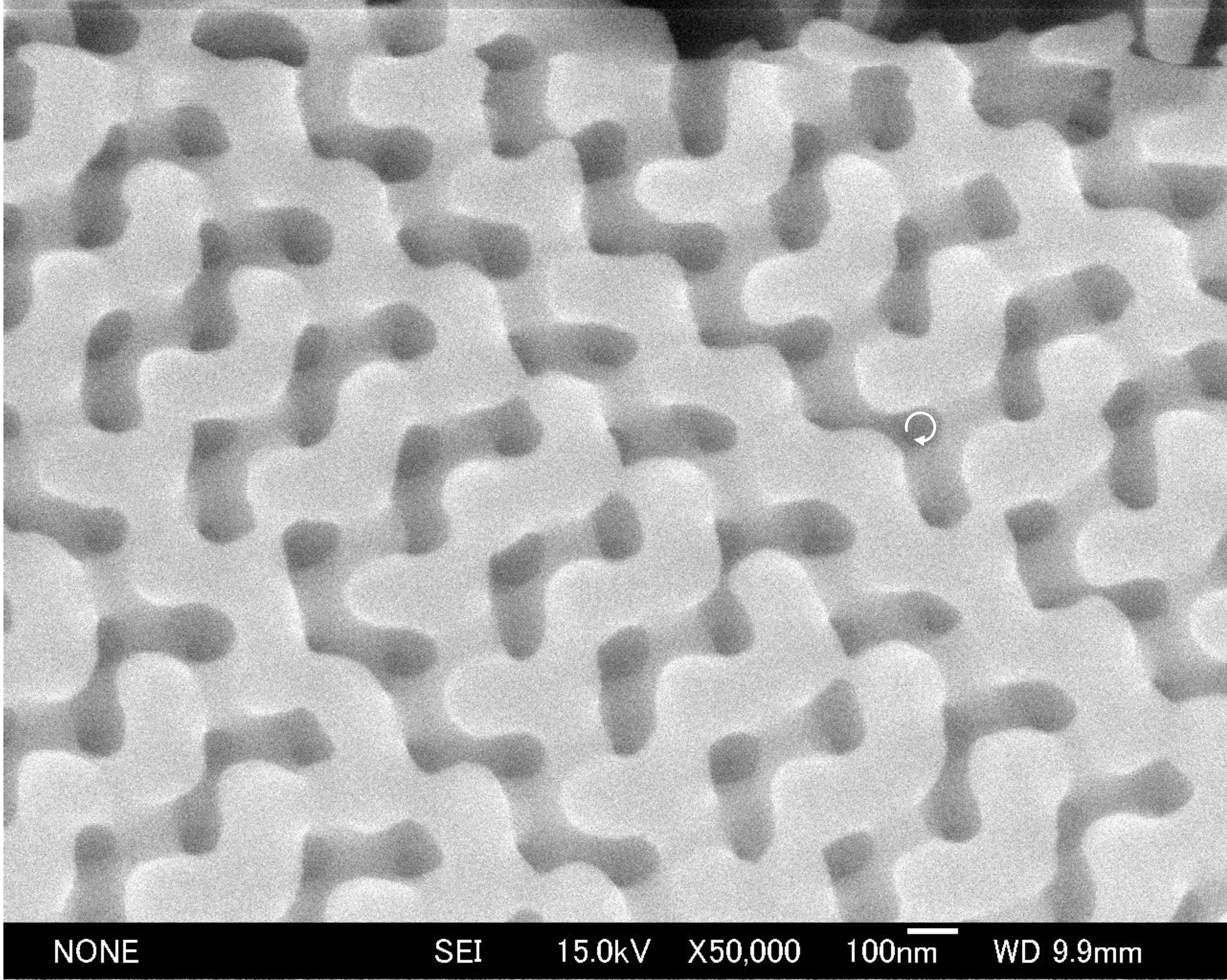

specimen No. 1
scale No. 2

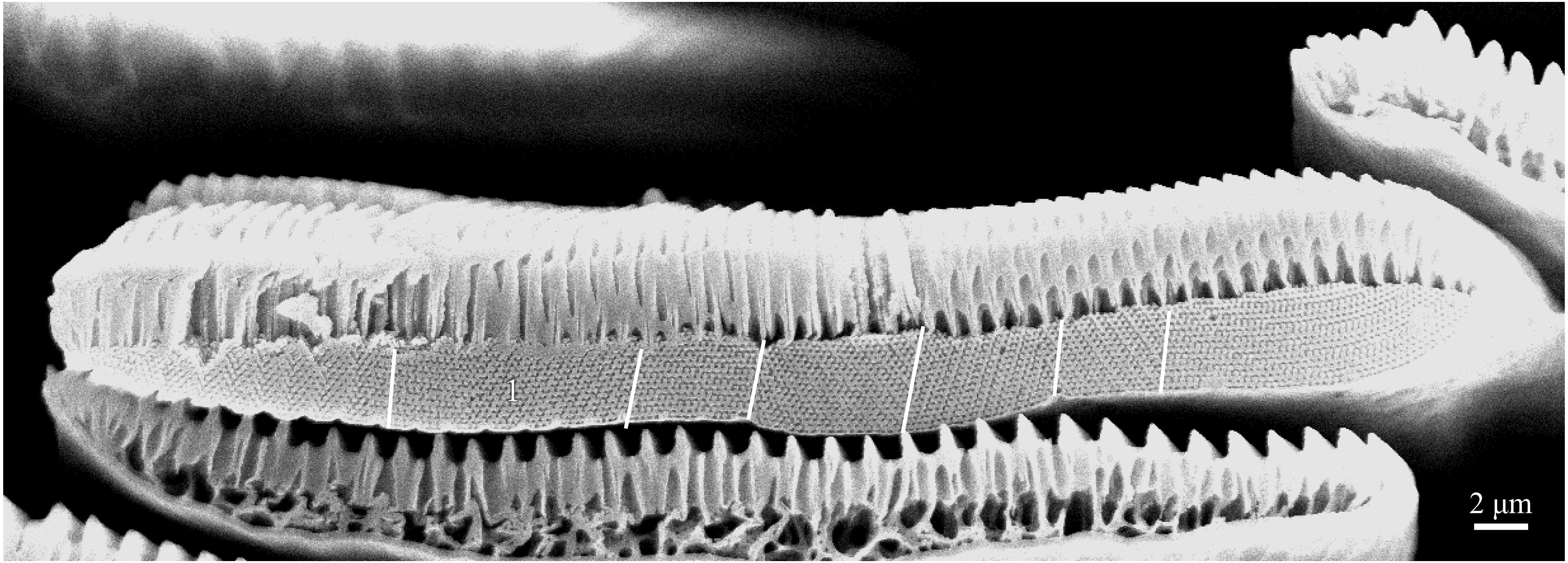

specimen No. 1
scale No. 2
domain No. 1
[111] lh spiral
LH gyroid

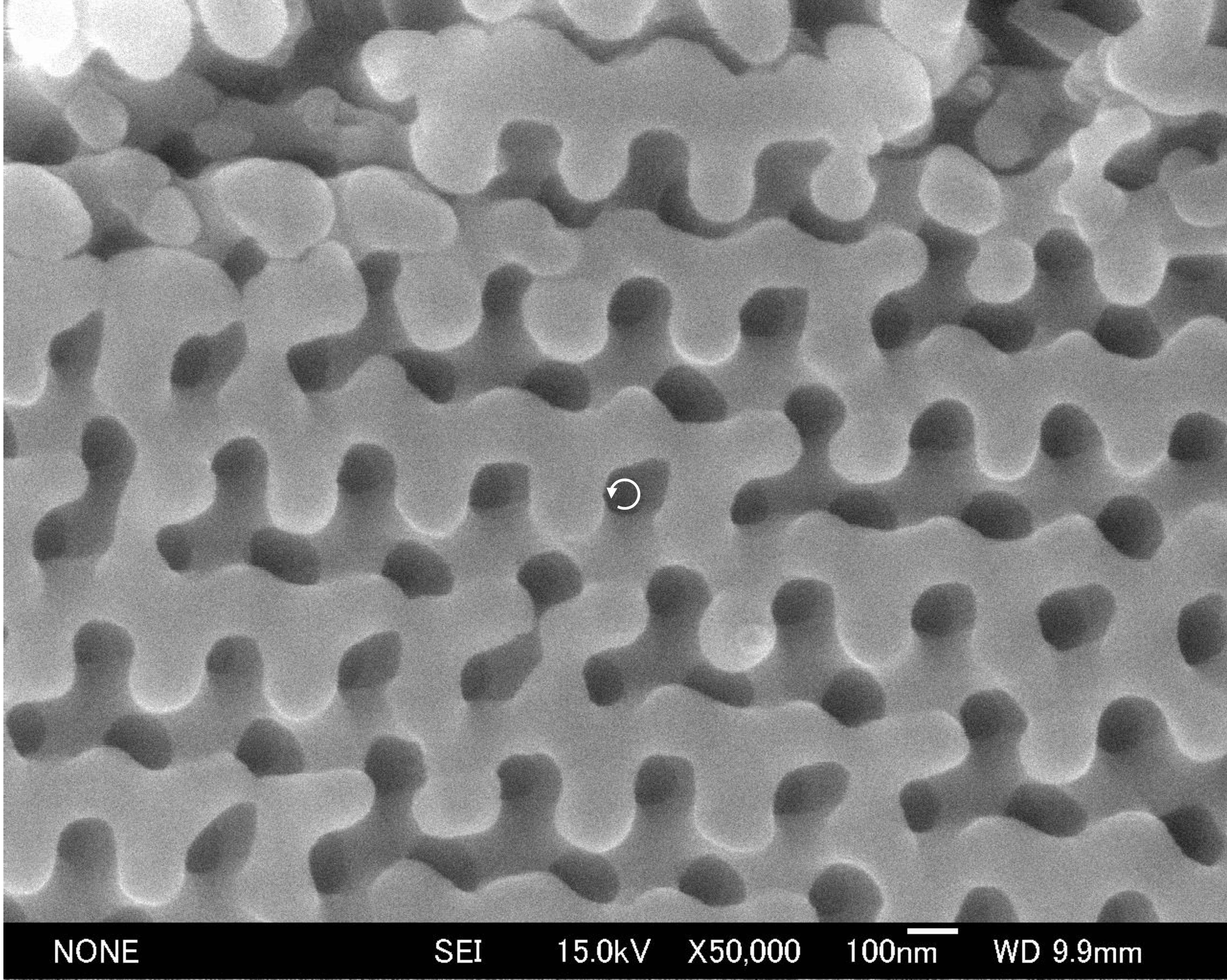

specimen No. 1
scale No. 3

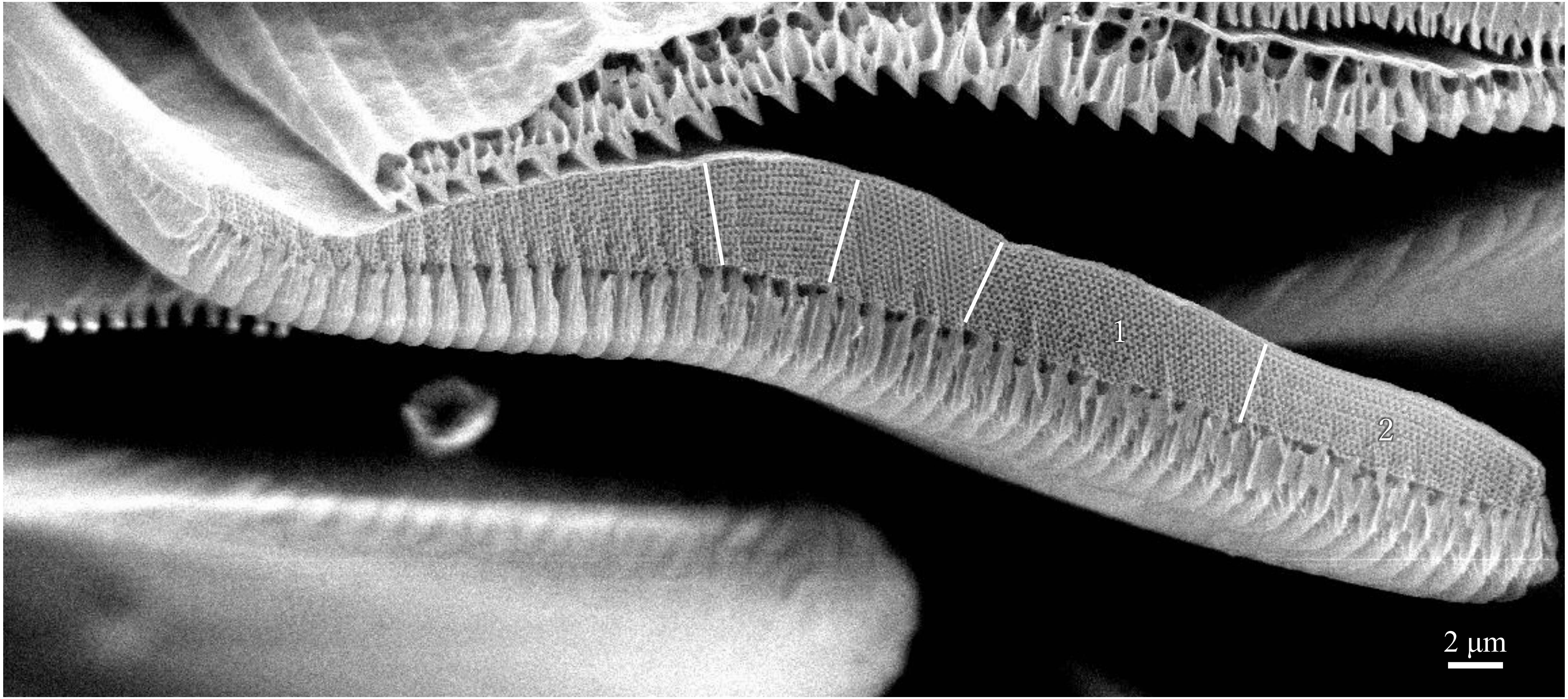

specimen No. 1
scale No. 3
domain No. 1
[111] lh spiral
LH gyroid

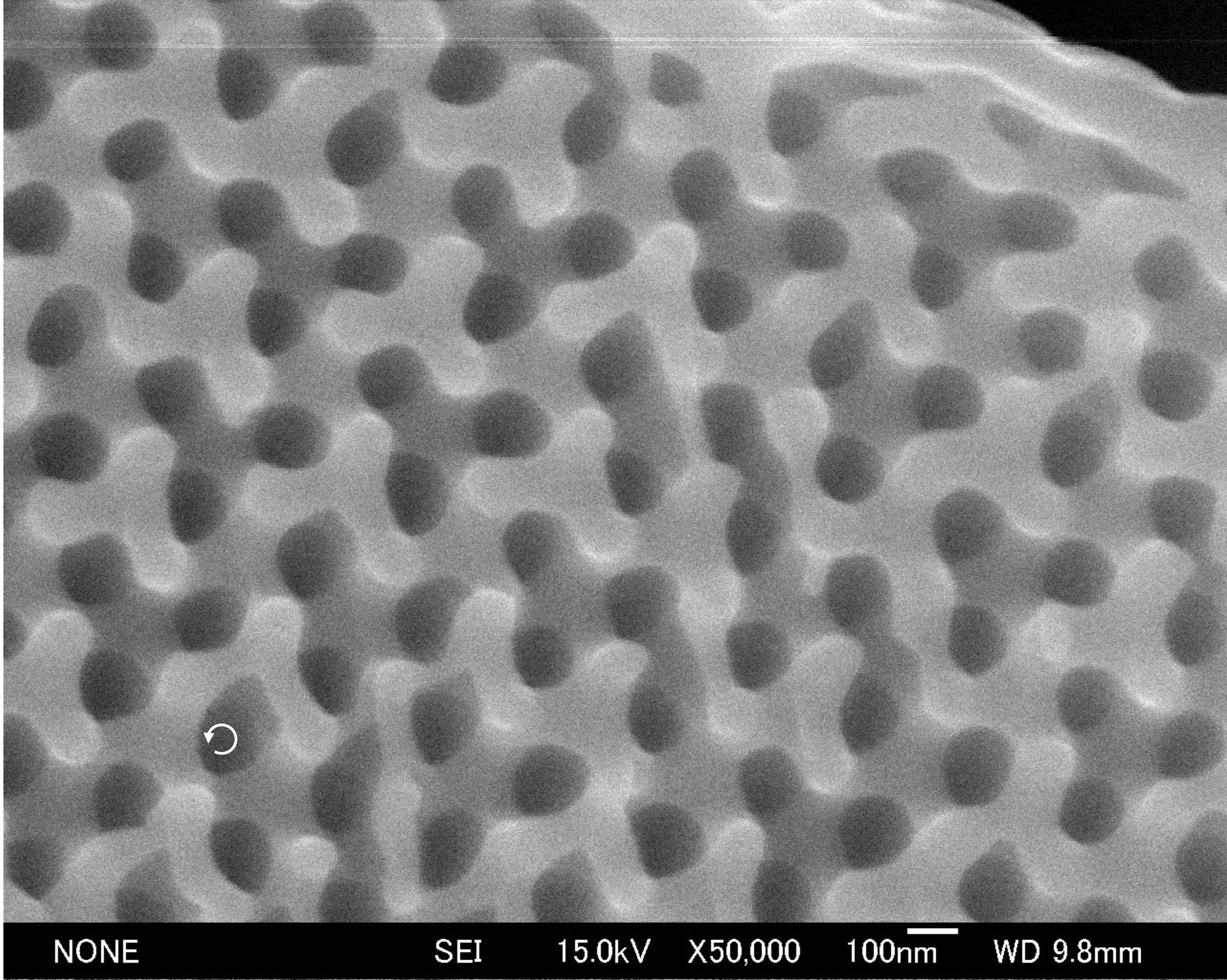

specimen No. 1
scale No. 3
domain No. 2
[111] lh spiral
LH gyroid

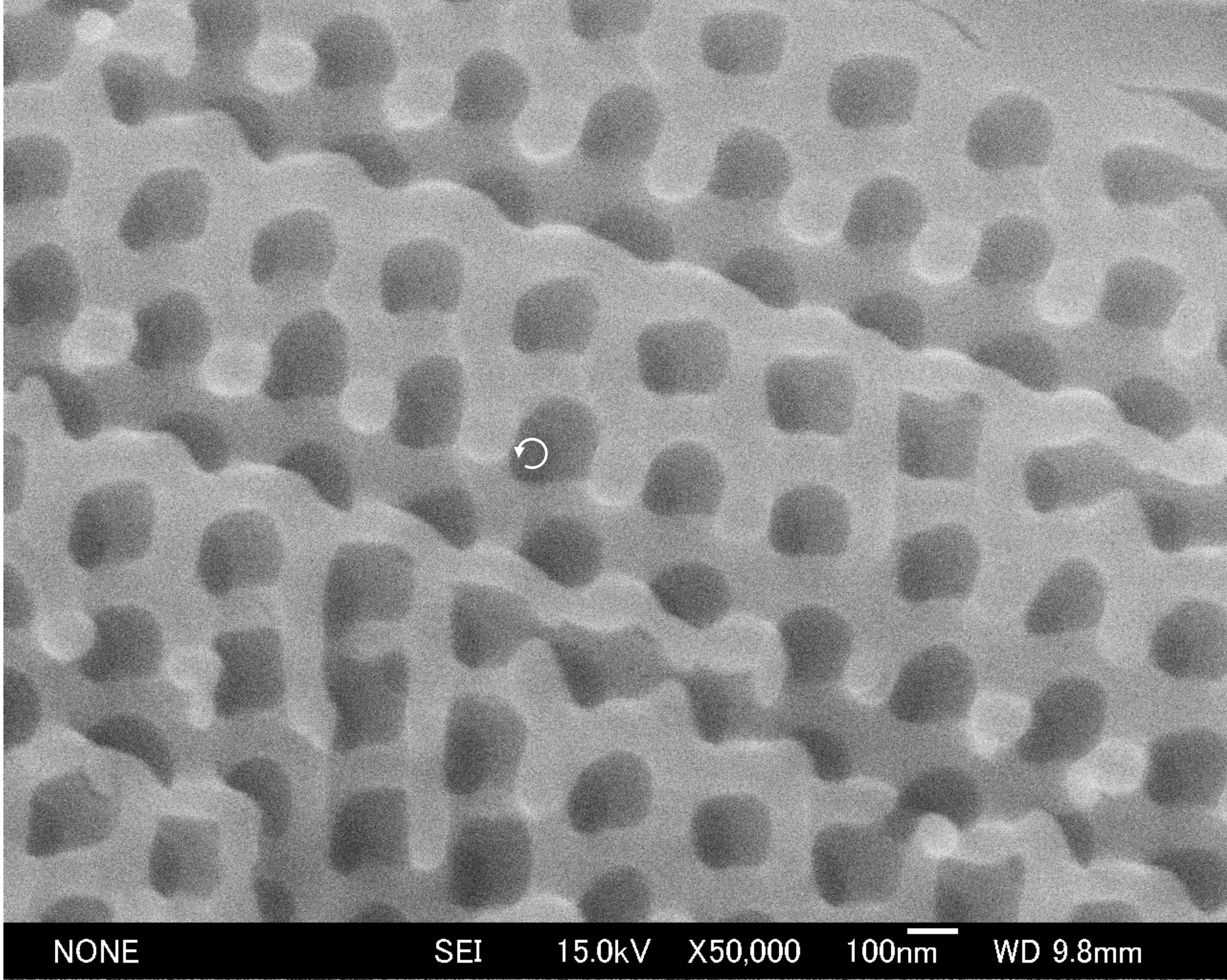

specimen No. 1
scale No. 4

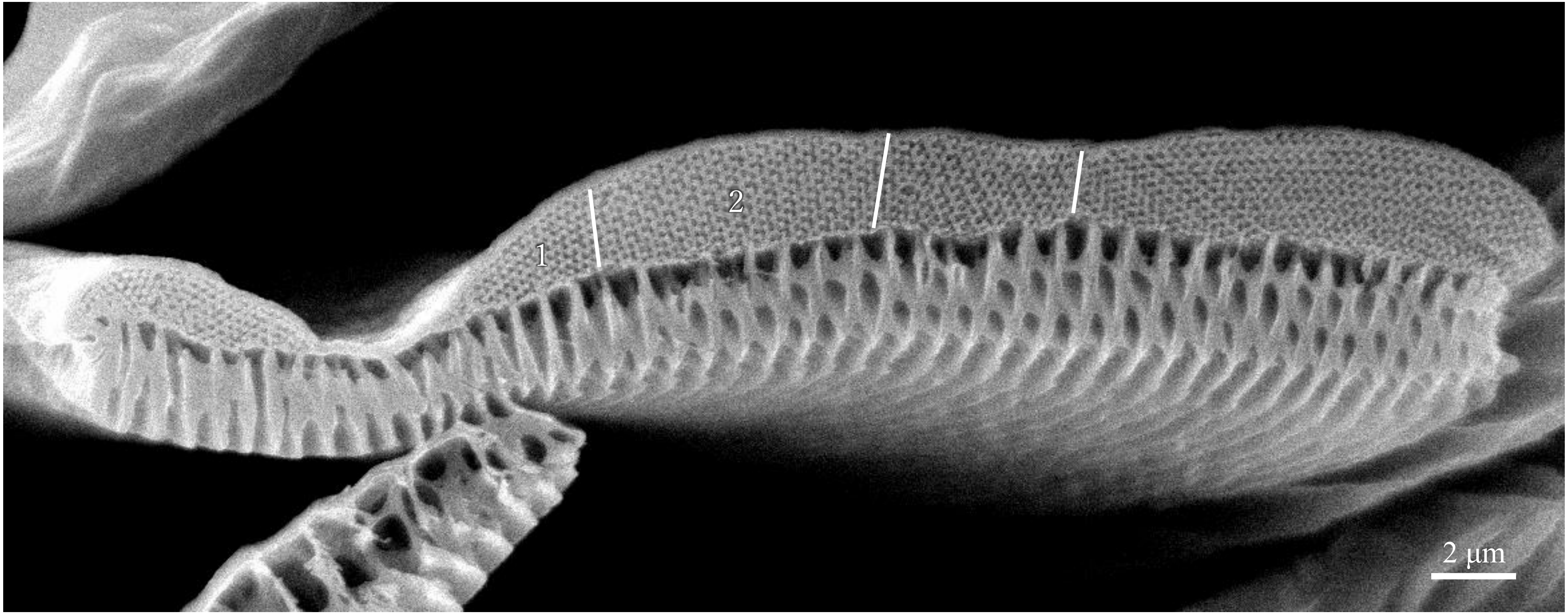

specimen No. 1
scale No. 4
domain No. 1
[111] lh spiral
LH gyroid

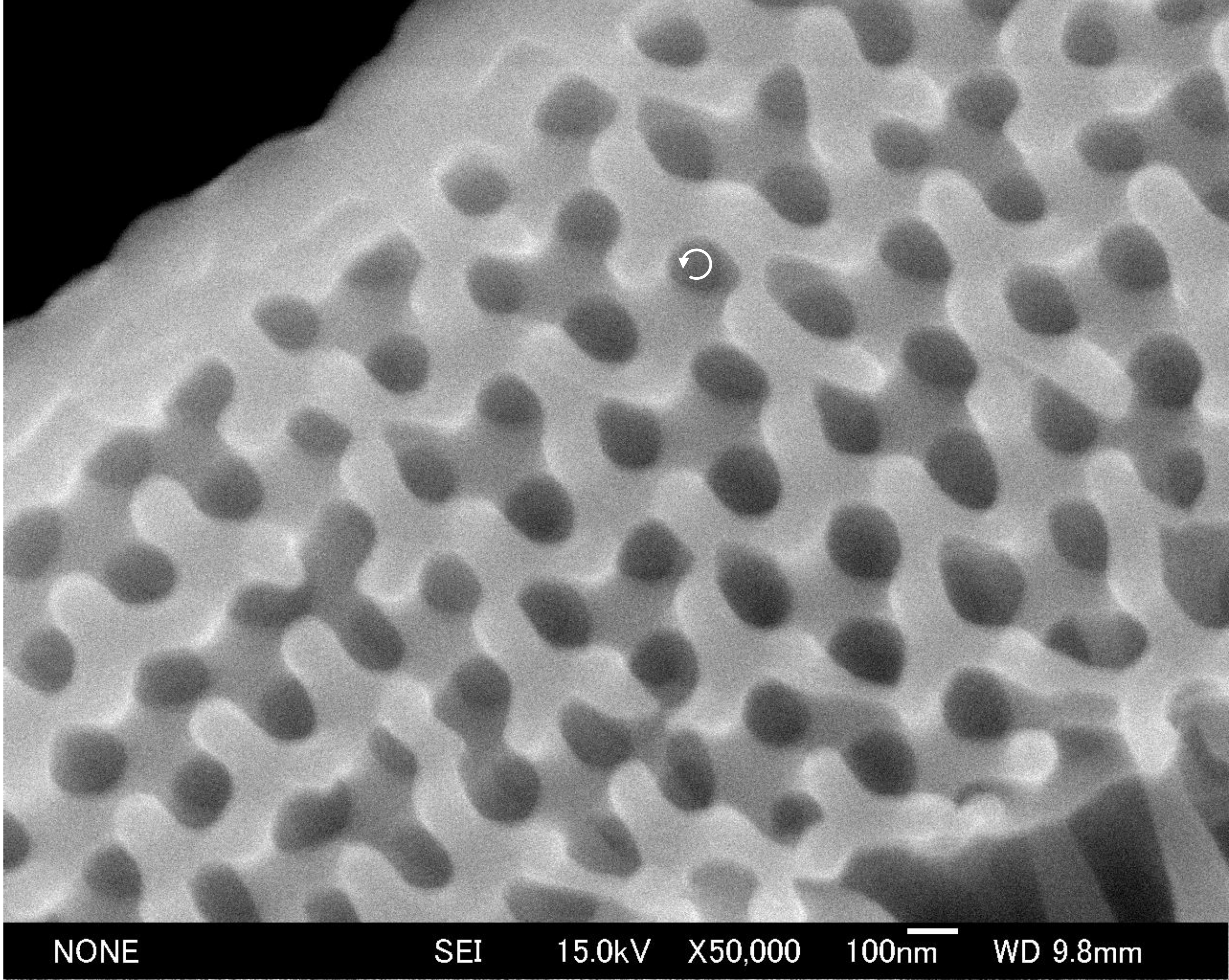

specimen No. 1
scale No. 4
domain No. 2
[111] lh spiral
LH gyroid

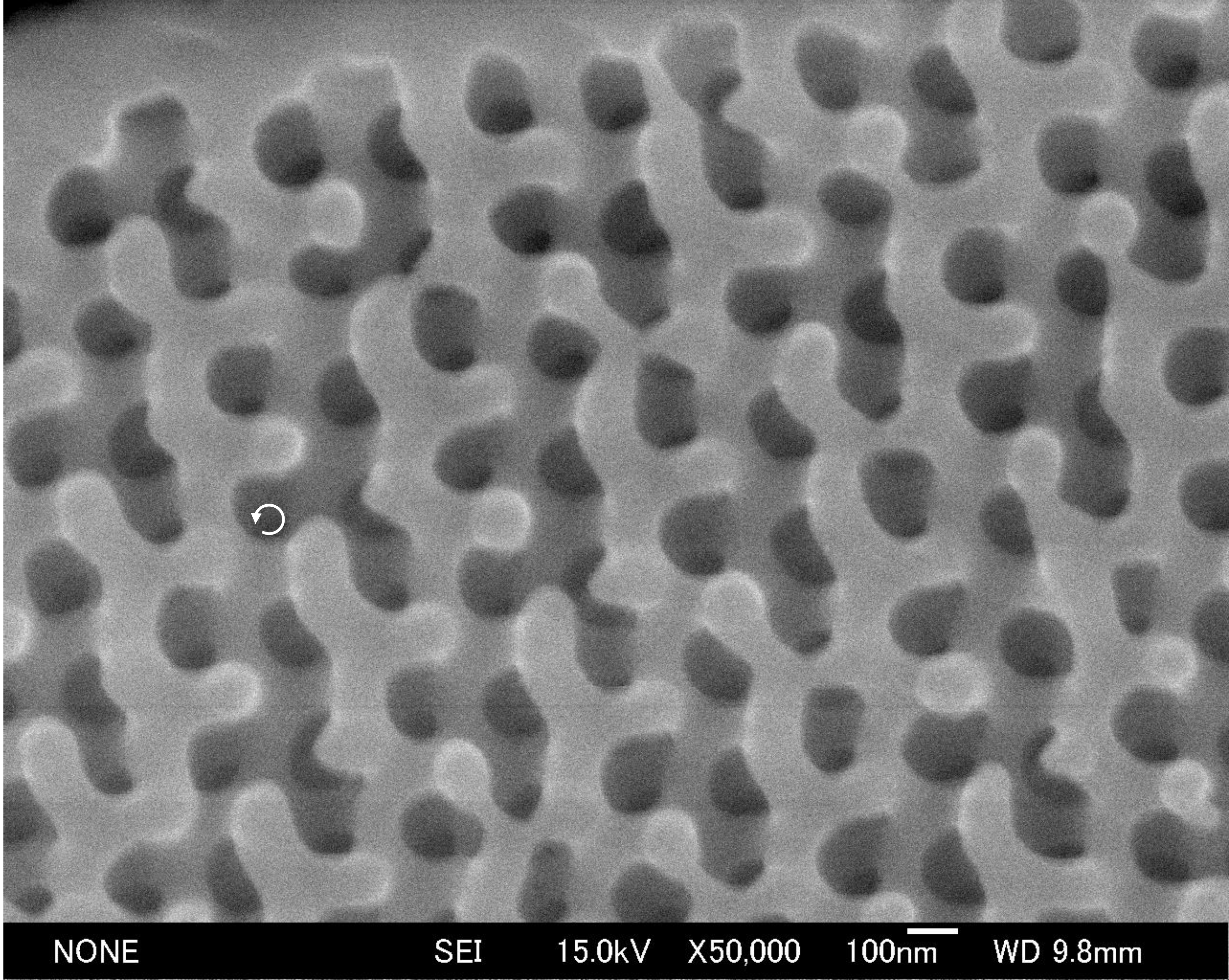

specimen No. 1
scale No. 5

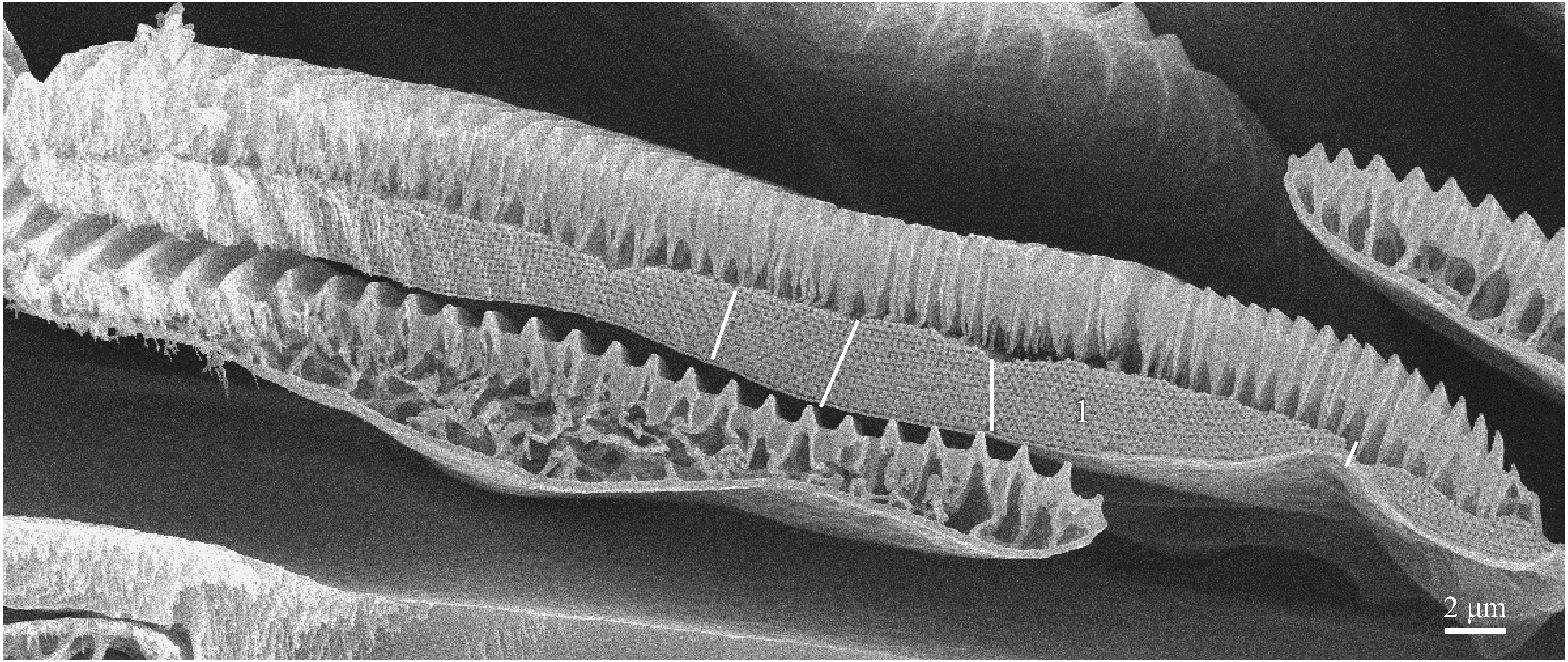

specimen No. 1
scale No. 5
domain No. 1
[111] lh spiral
LH gyroid

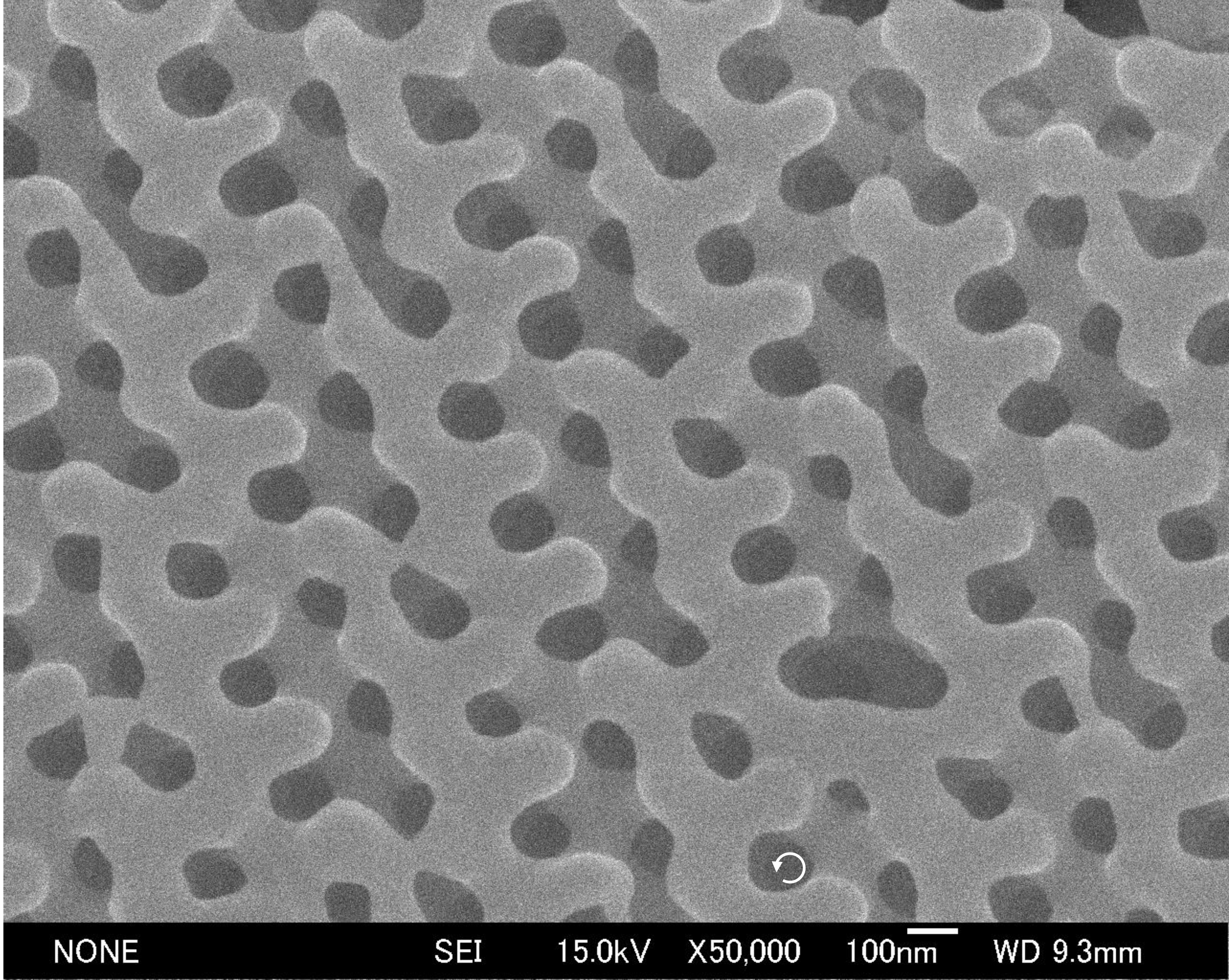

specimen No. 1
scale No. 6

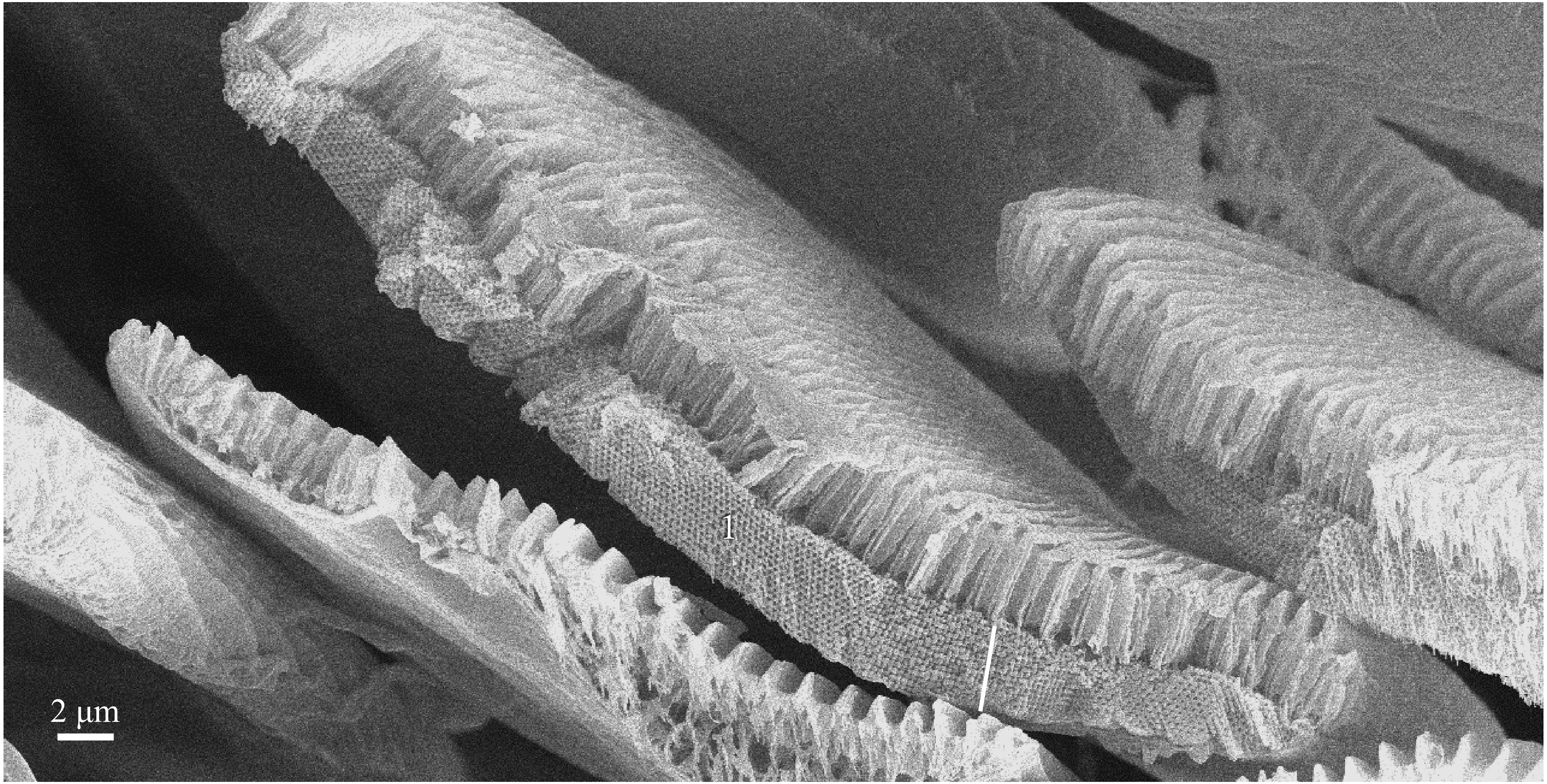

specimen No. 1
scale No. 6
domain No. 1
[111] lh spiral
LH gyroid

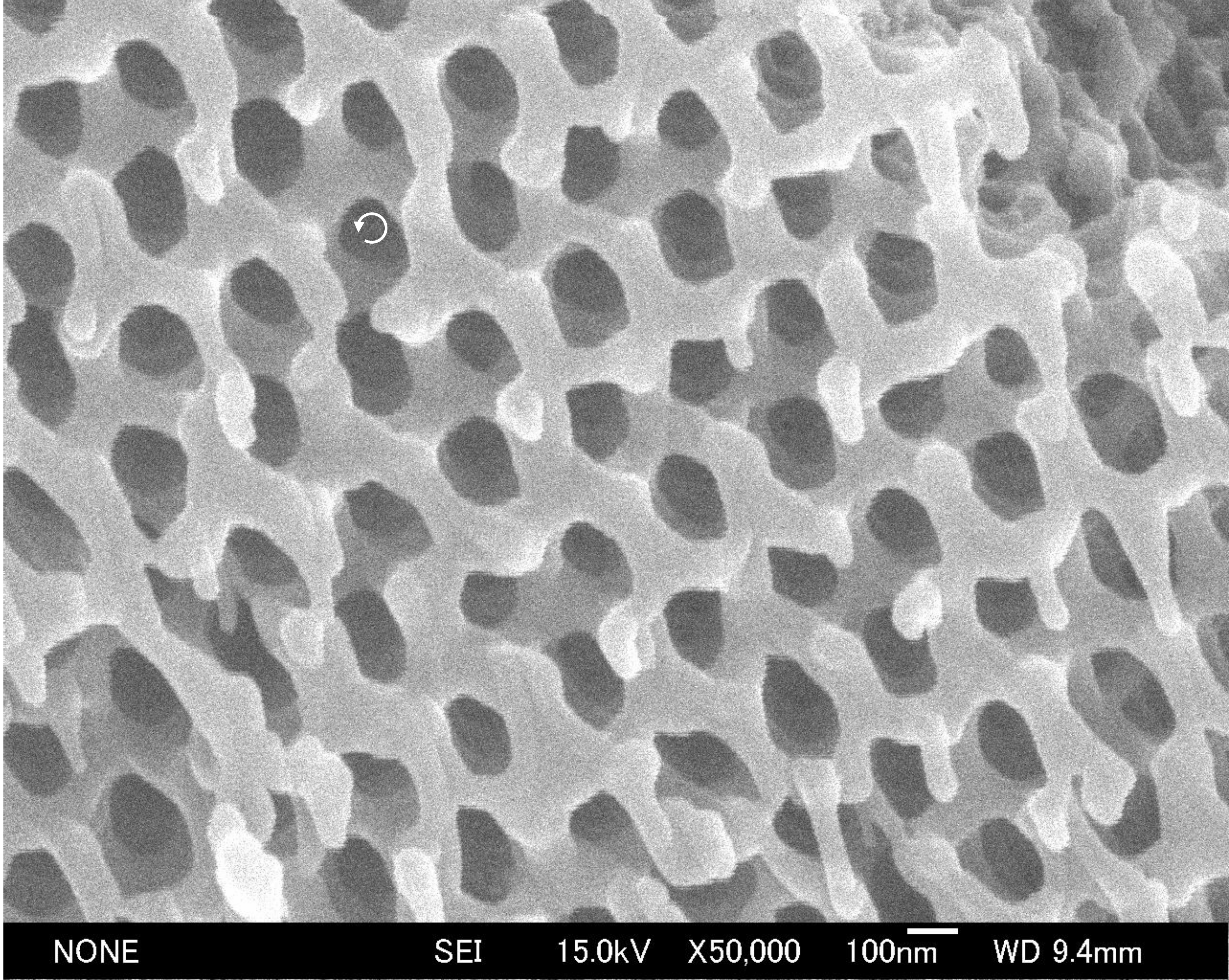

specimen No. 1
scale No. 7

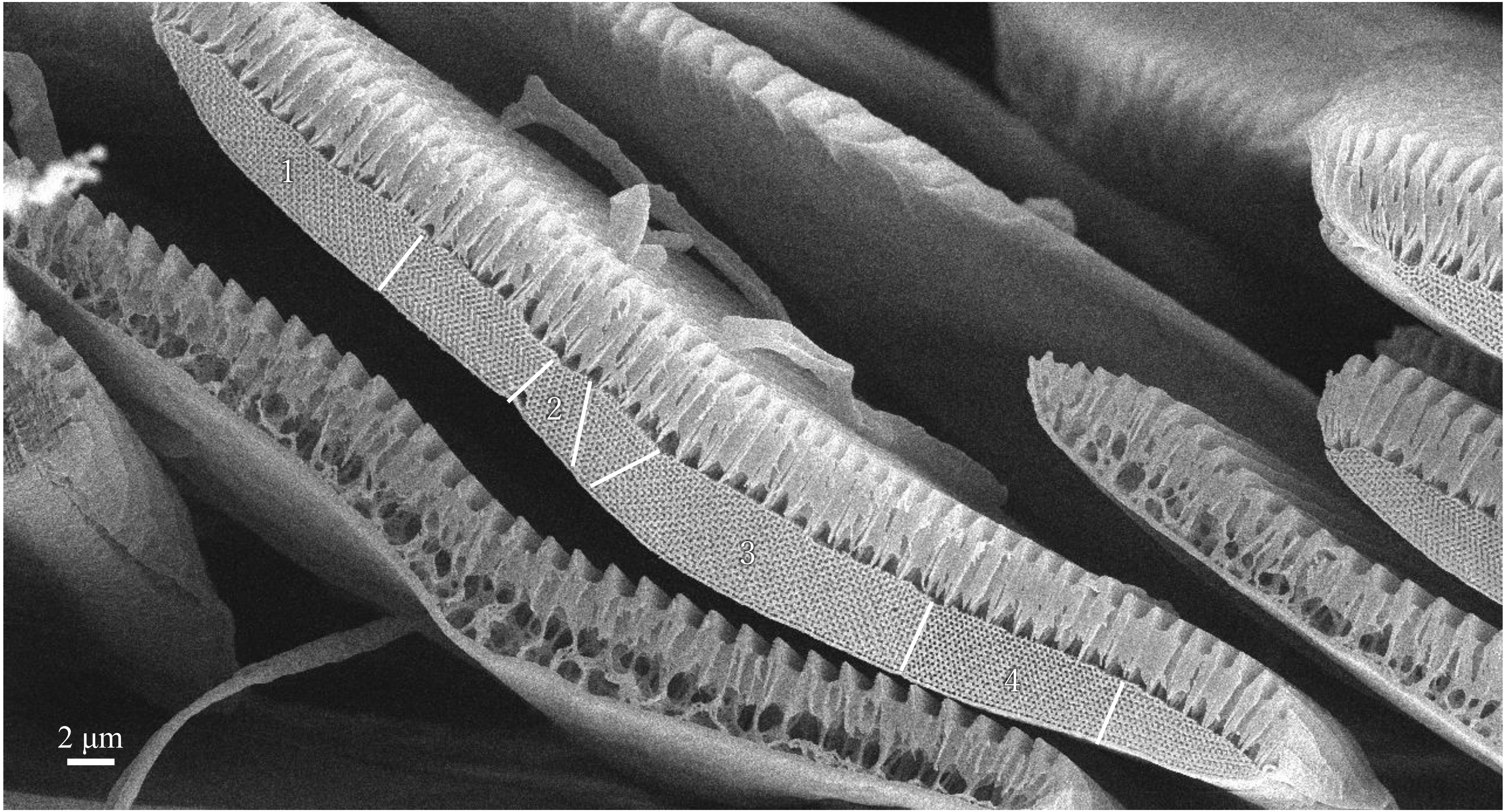

specimen No. 1
scale No. 7
domain No. 1
[100] rh spiral
LH gyroid

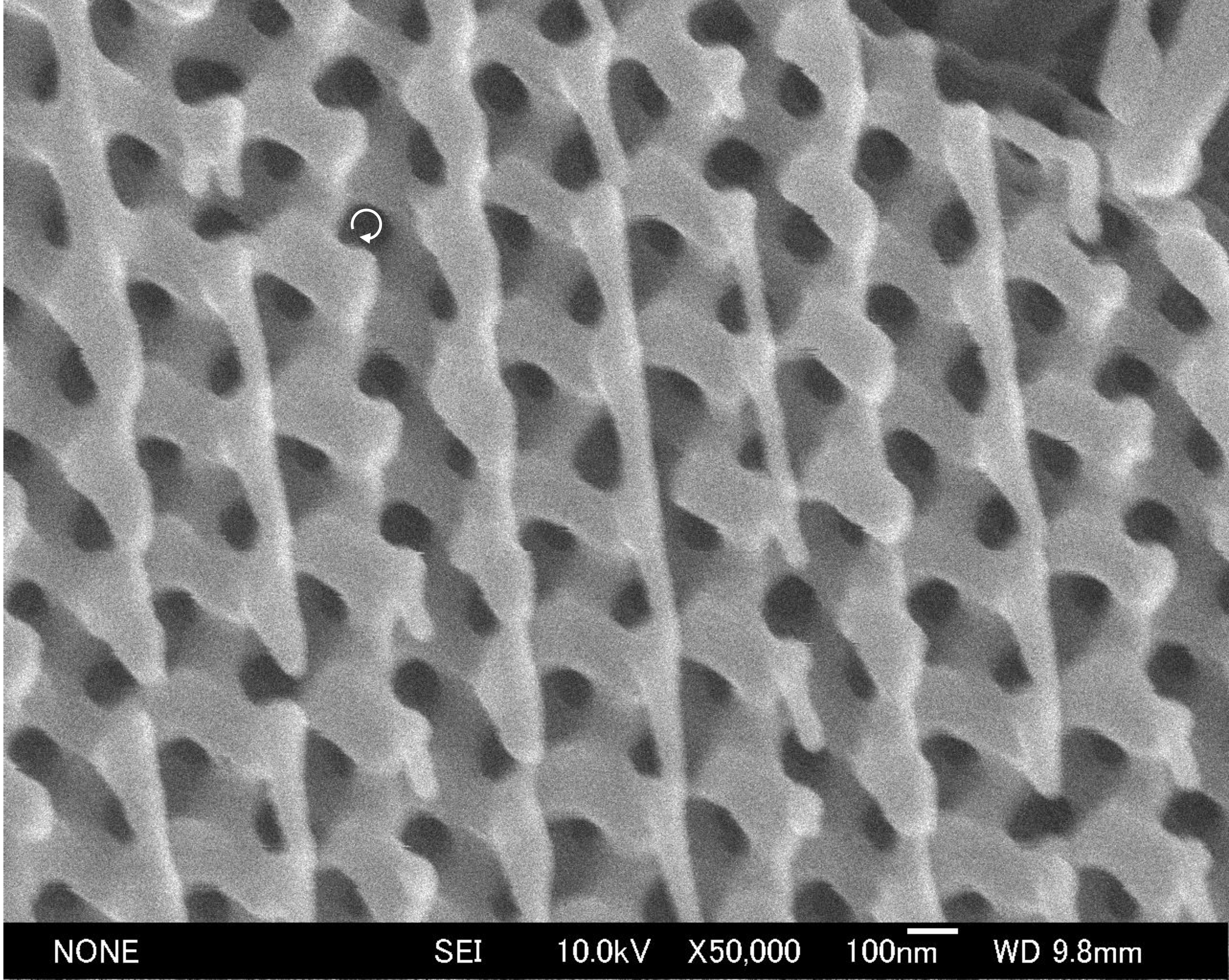

specimen No. 1
scale No. 7
domain No. 2
[111] lh spiral
LH gyroid

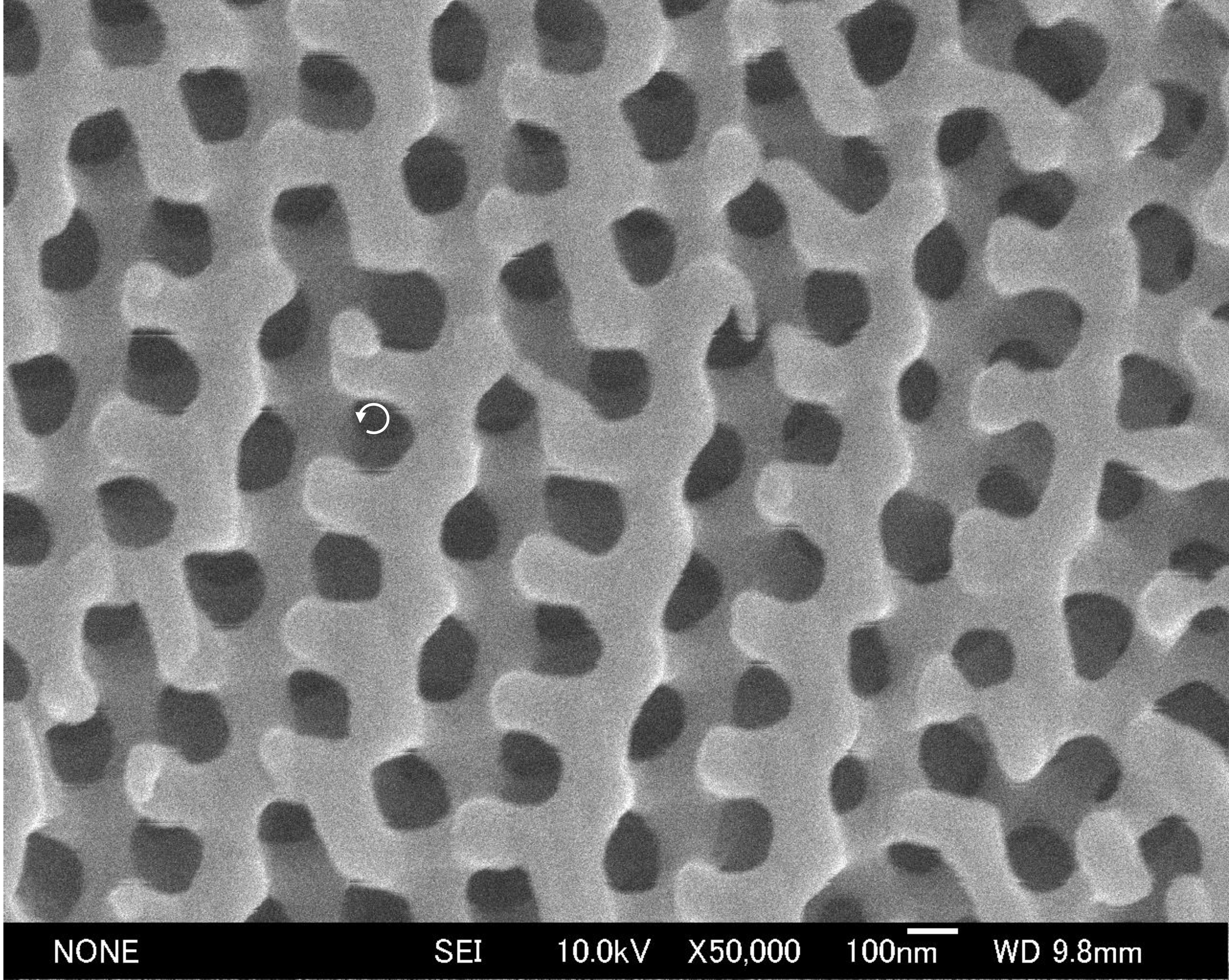

specimen No. 1
scale No. 7
domain No. 3
[100] rh spiral
LH gyroid

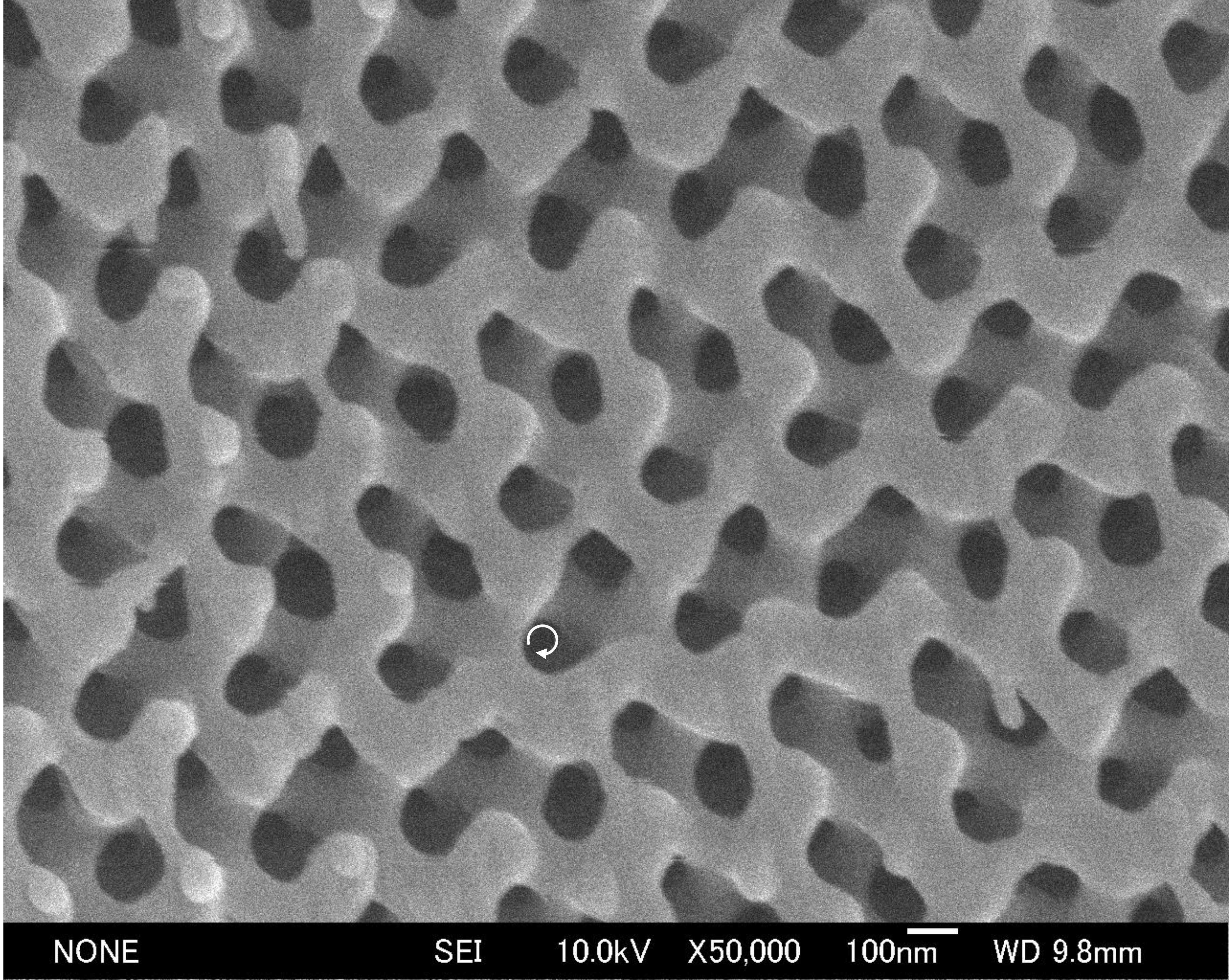

specimen No. 1
scale No. 7
domain No. 4
[111] lh spiral
LH gyroid

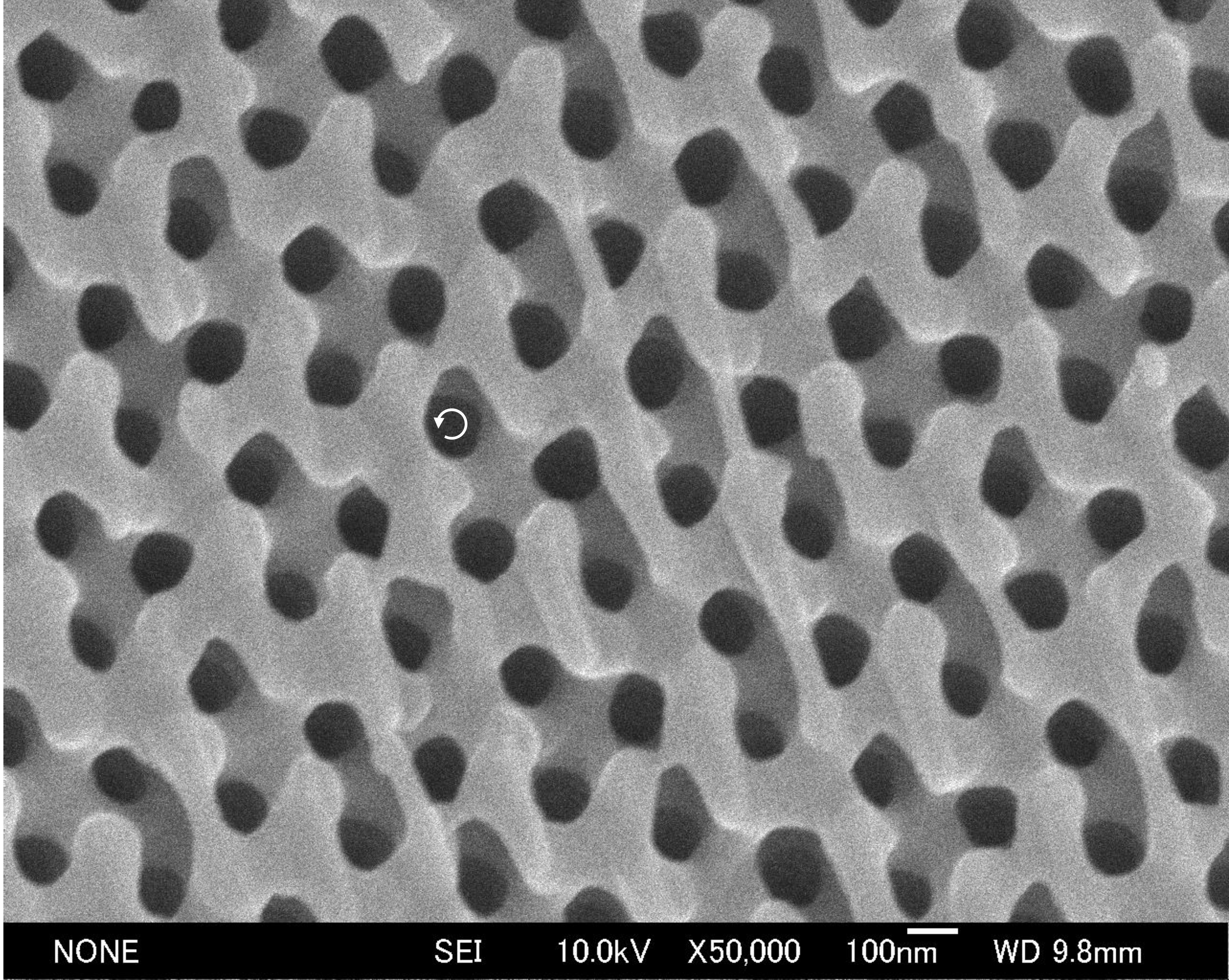

specimen No. 1
scale No. 8

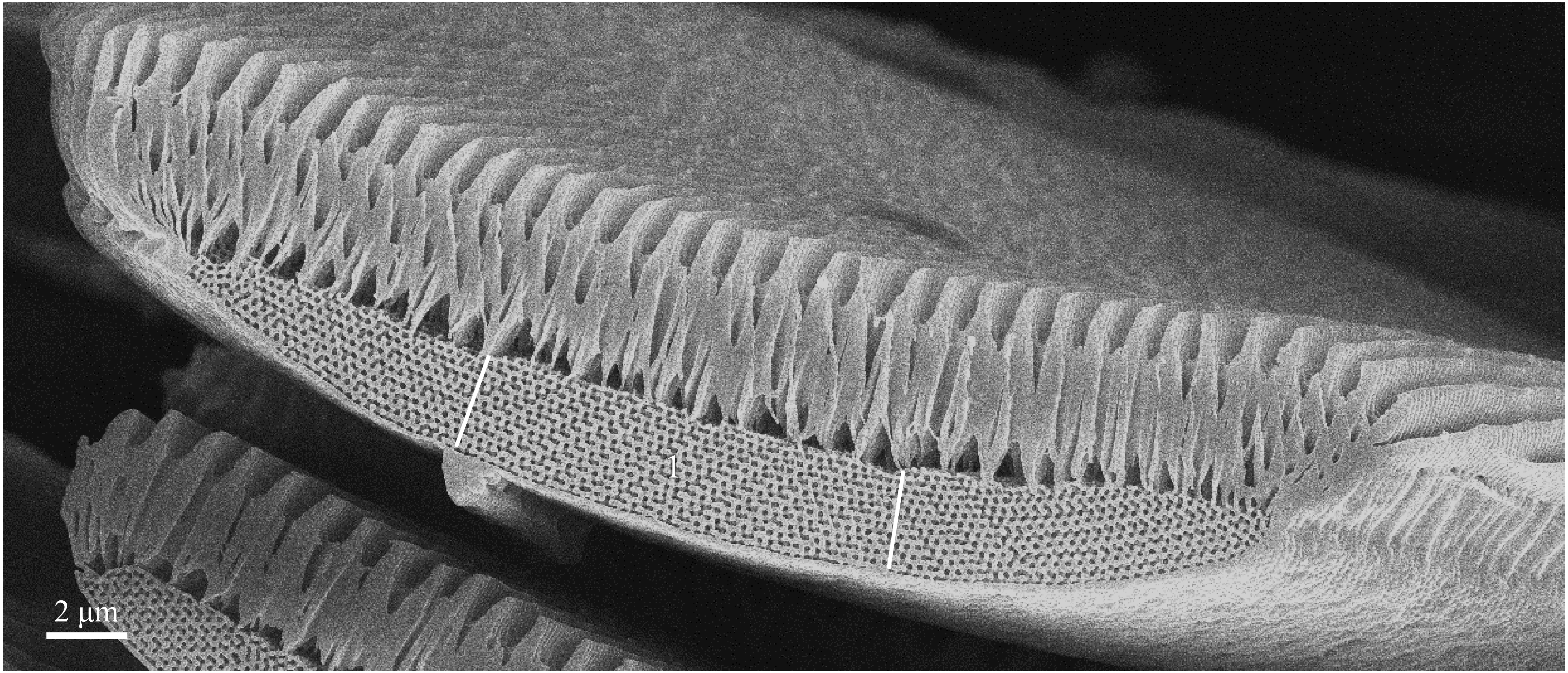

specimen No. 1
scale No. 8
domain No. 1
[100] rh spiral
LH gyroid

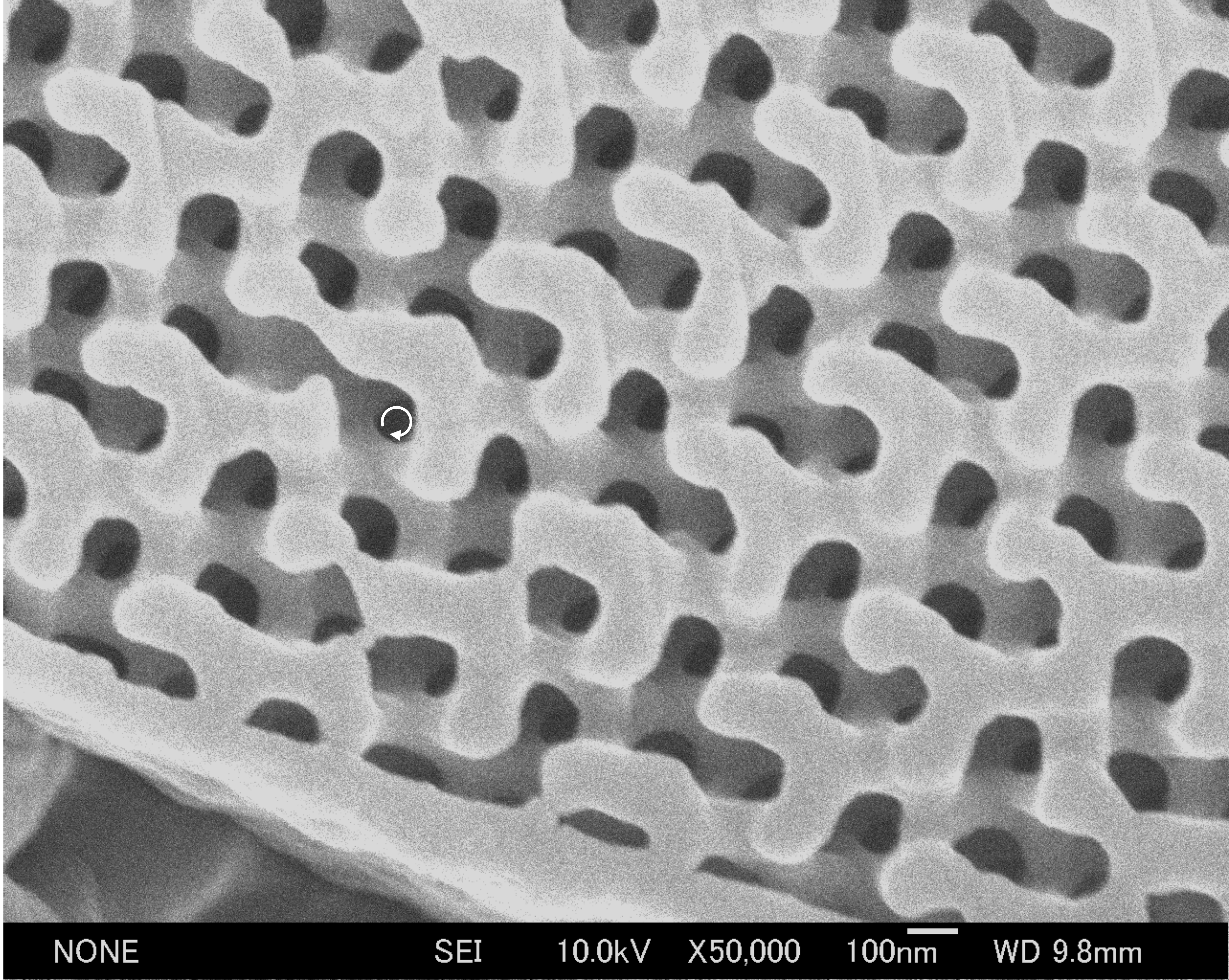

specimen No. 1
scale No. 9

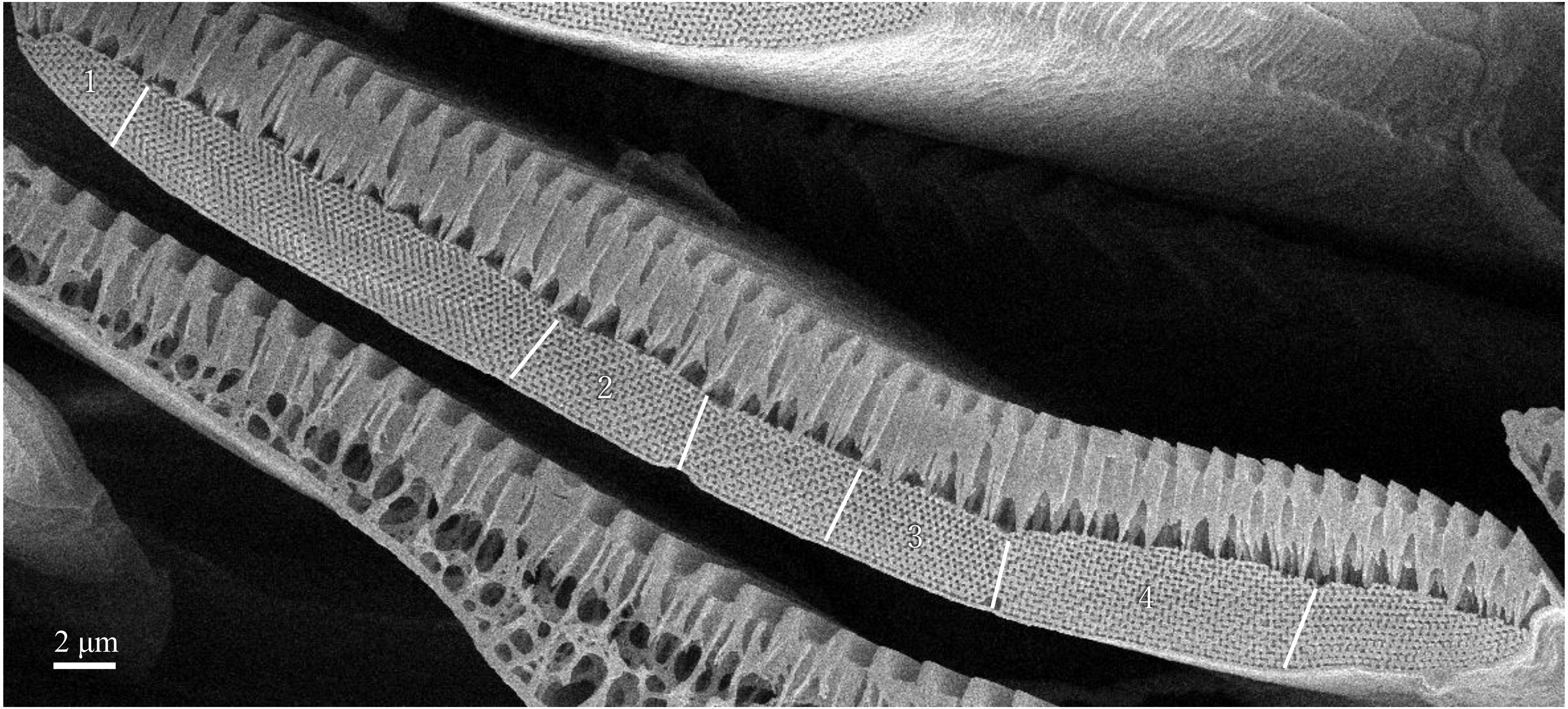

specimen No. 1
scale No. 9
domain No. 1
[100] rh spiral
LH gyroid

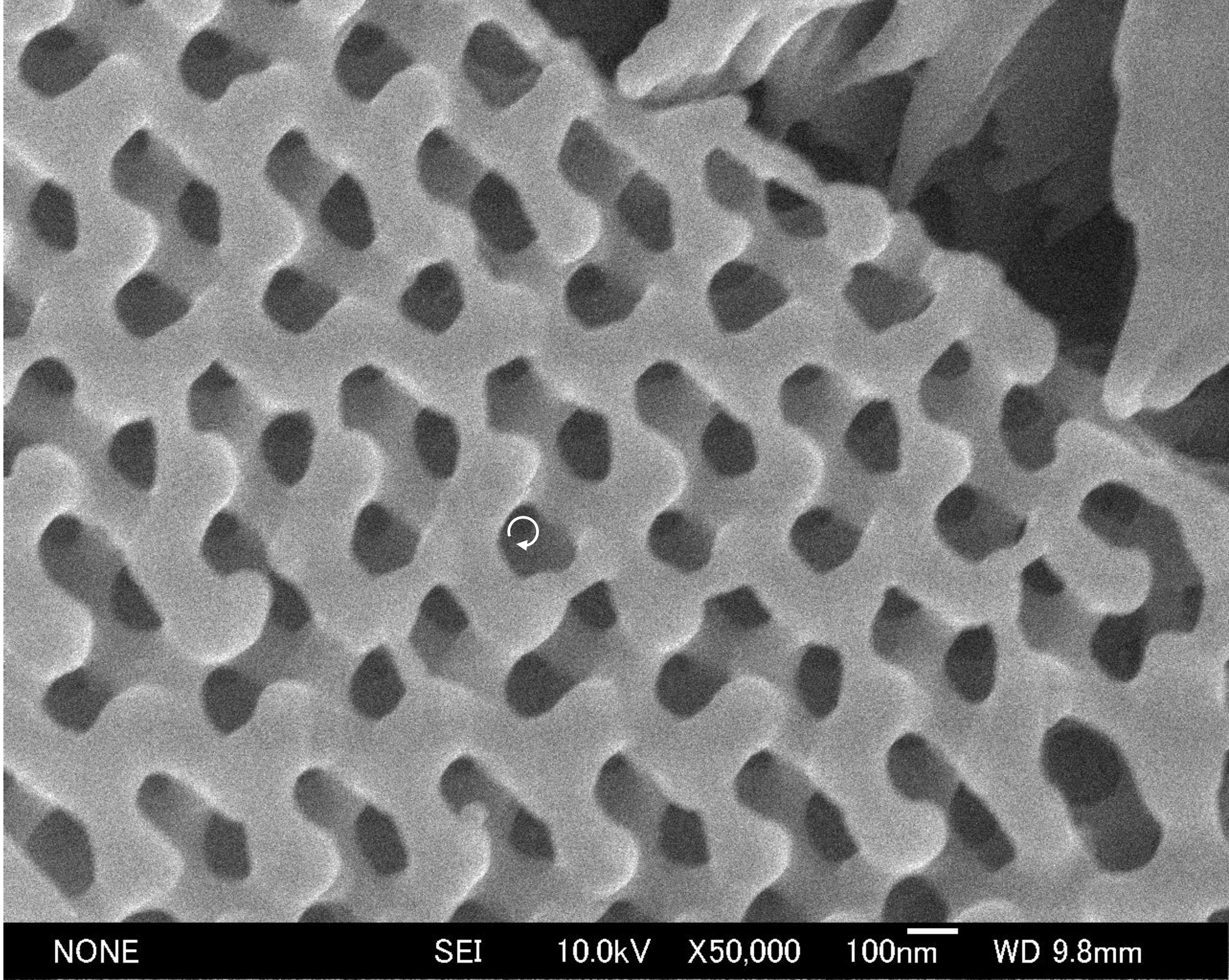

specimen No. 1
scale No. 9
domain No. 2
[100] rh spiral
LH gyroid

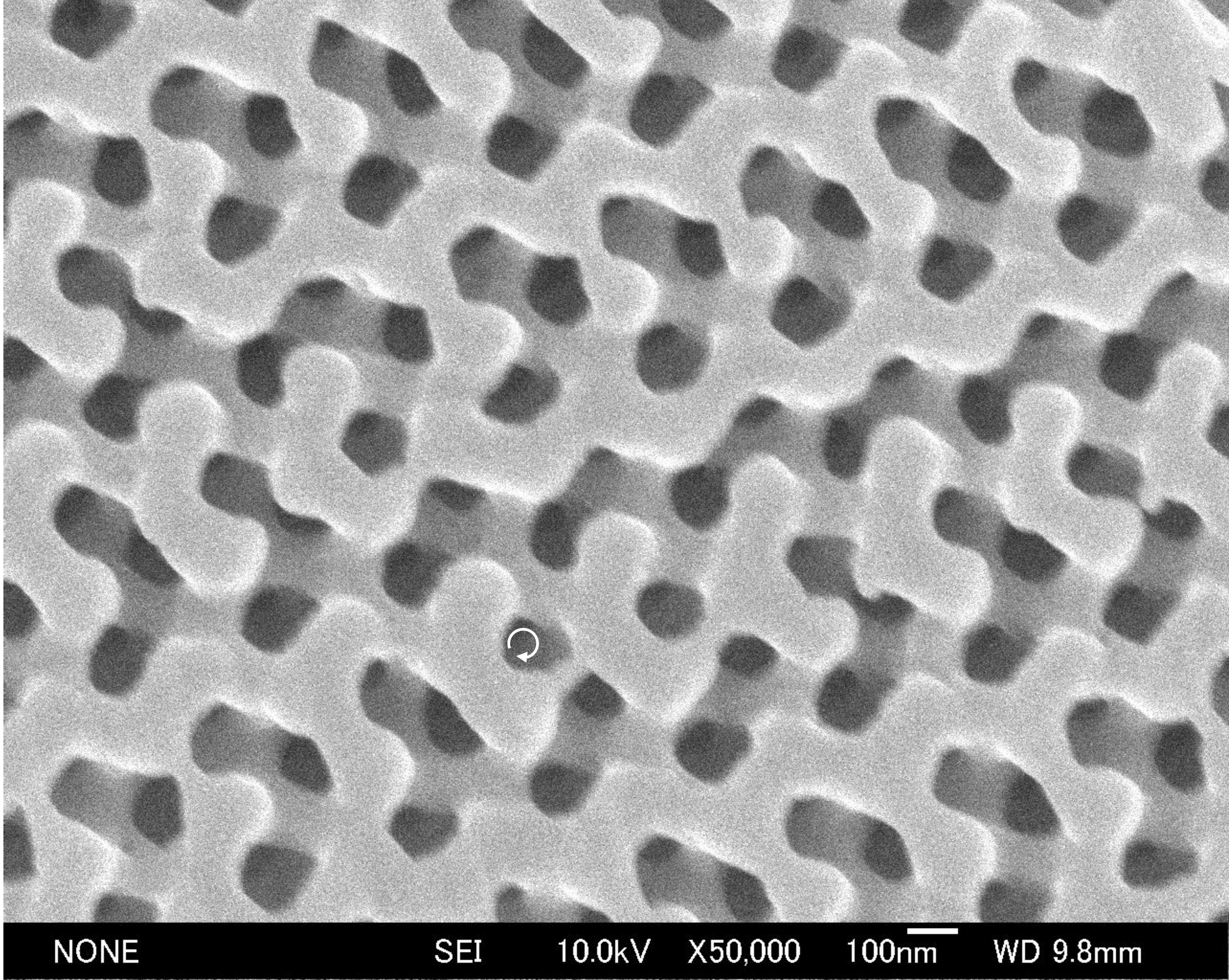

specimen No. 1
scale No. 9
domain No. 3
[111] lh spiral
LH gyroid

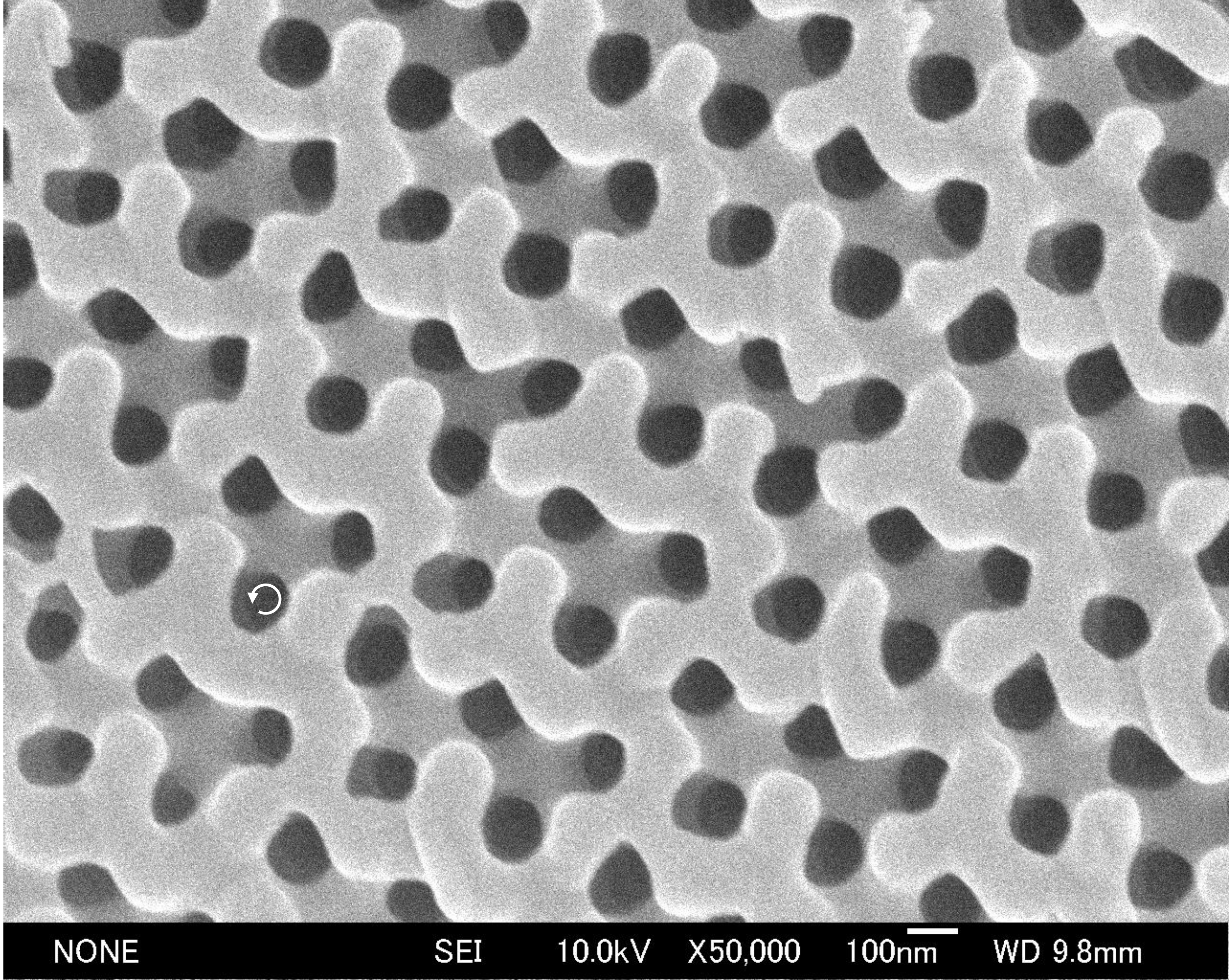

specimen No. 1
scale No. 9
domain No. 4
[100] rh spiral
LH gyroid

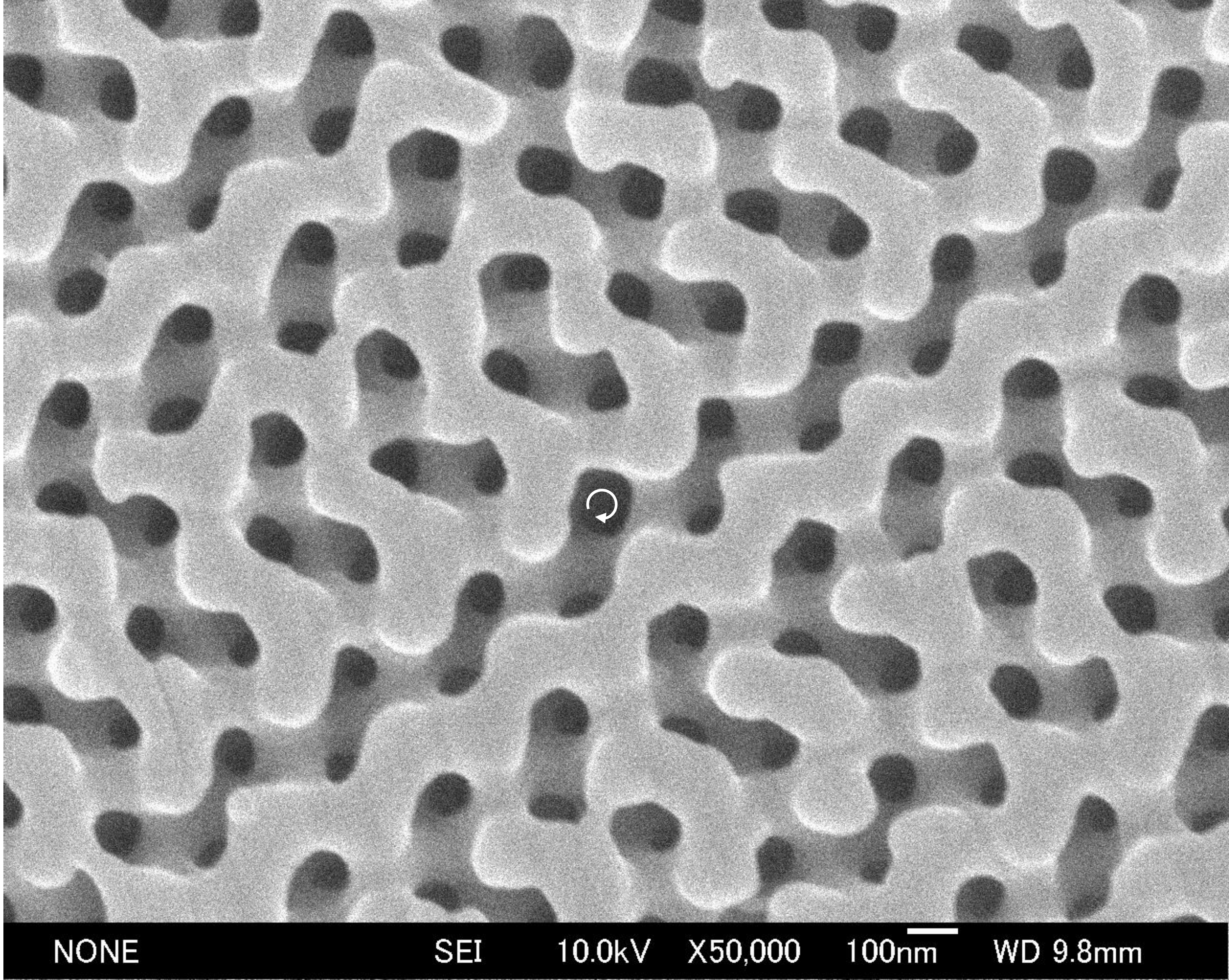

specimen No. 1
scale No. 10

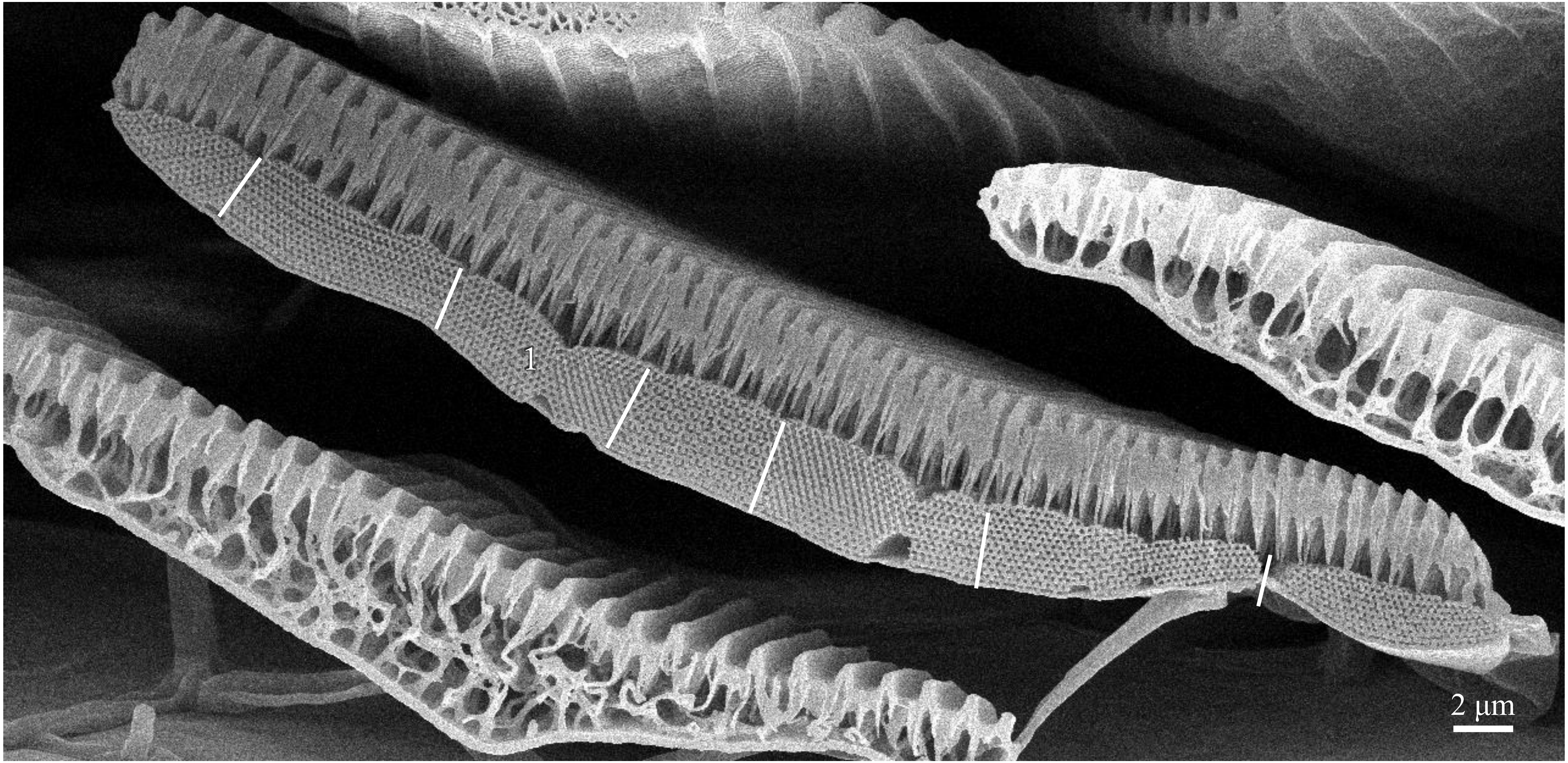

specimen No. 1
scale No. 10
domain No. 1
[111] lh spiral
LH gyroid

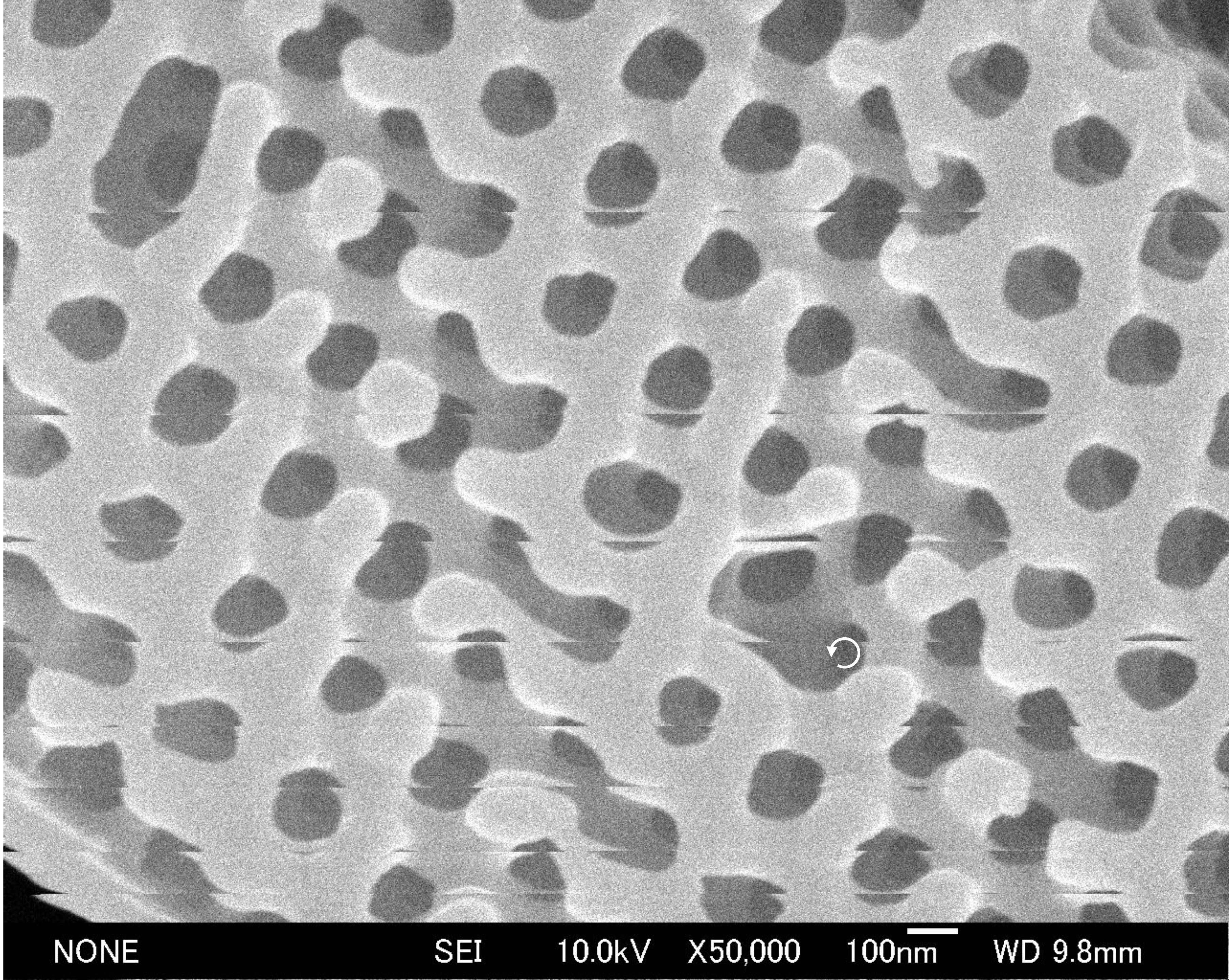

specimen No. 1
scale No. 11

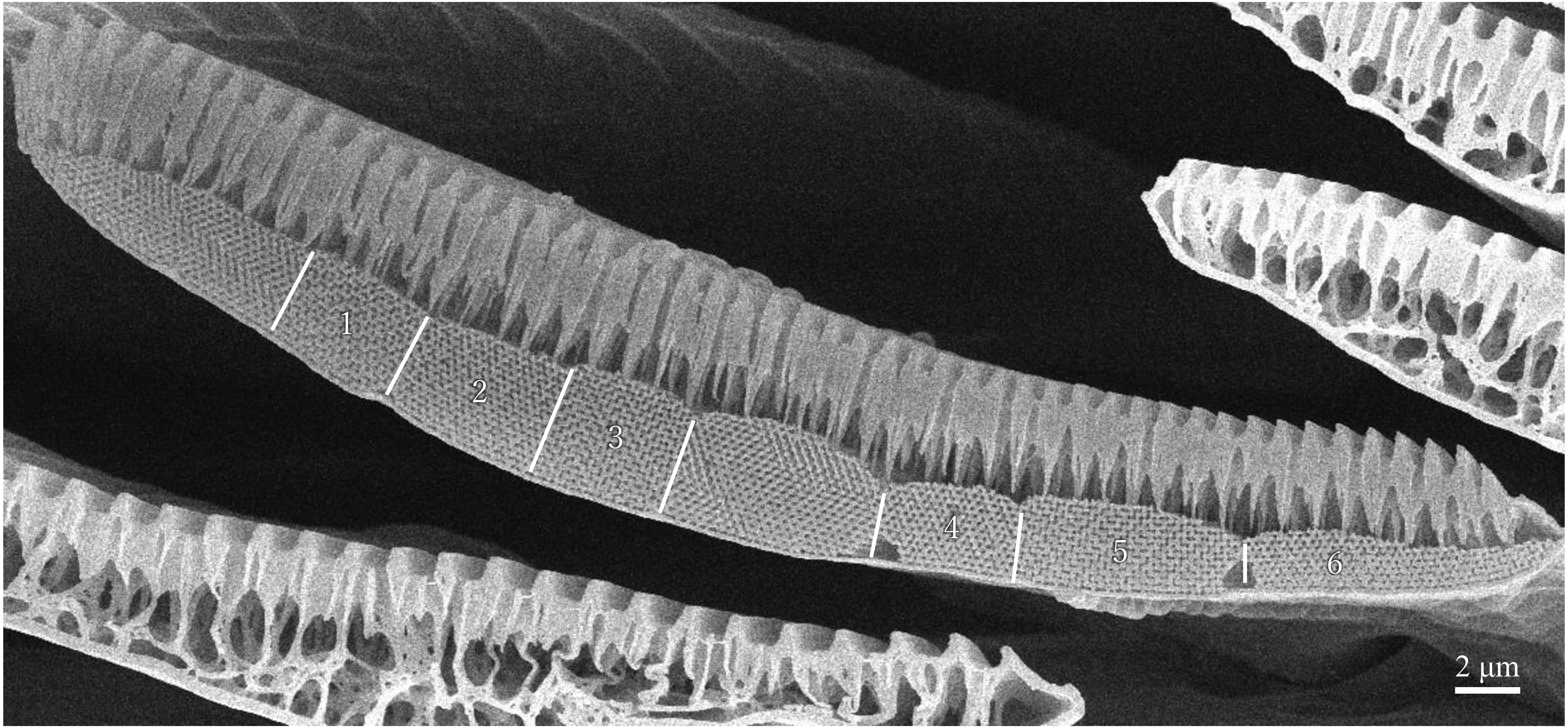

specimen No. 1
scale No. 11
domain No. 1
[100] rh spiral
LH gyroid

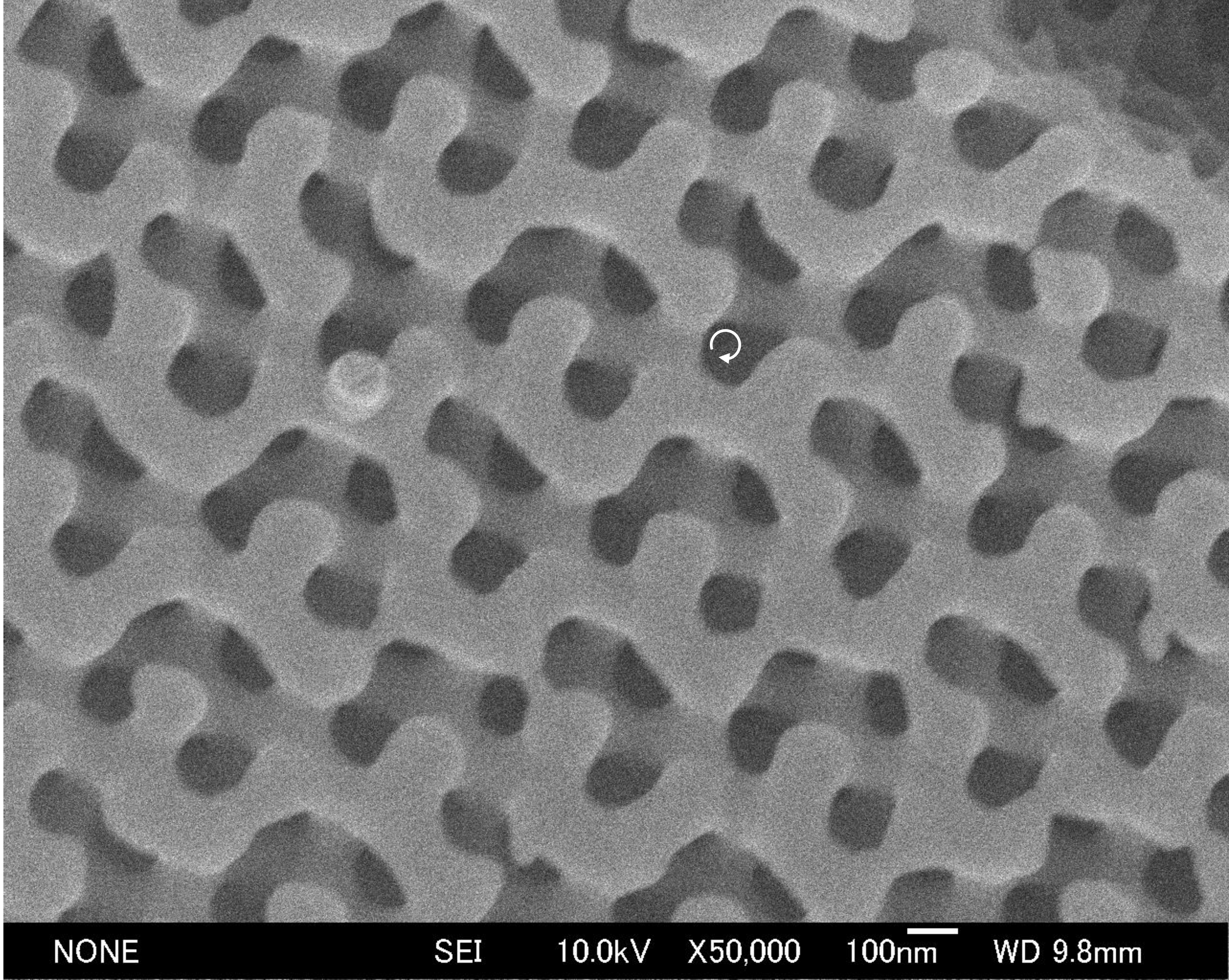

specimen No. 1
scale No. 11
domain No. 2
[111] rh spiral
RH gyroid

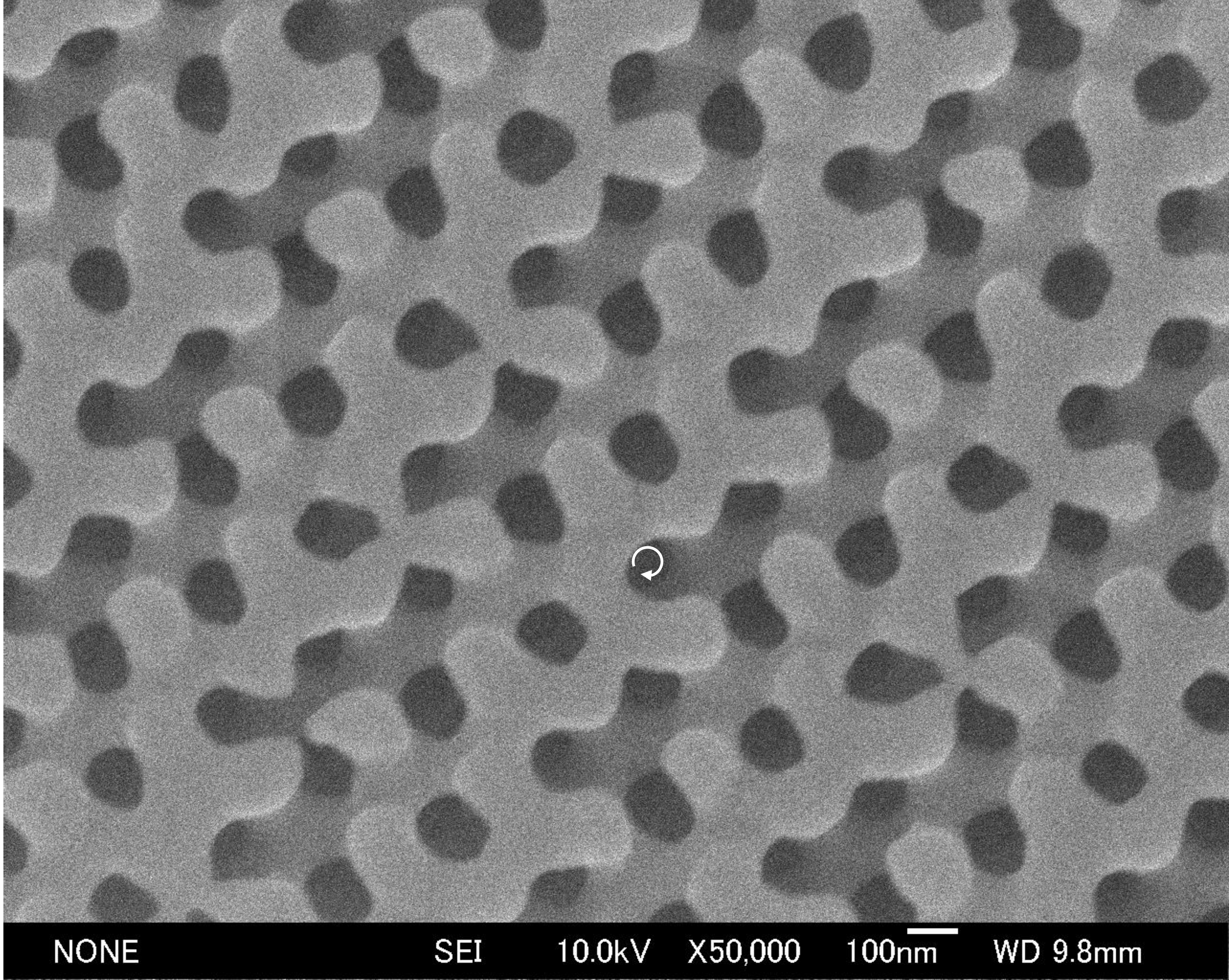

specimen No. 1
scale No. 11
domain No. 3
[100] rh spiral
LH gyroid

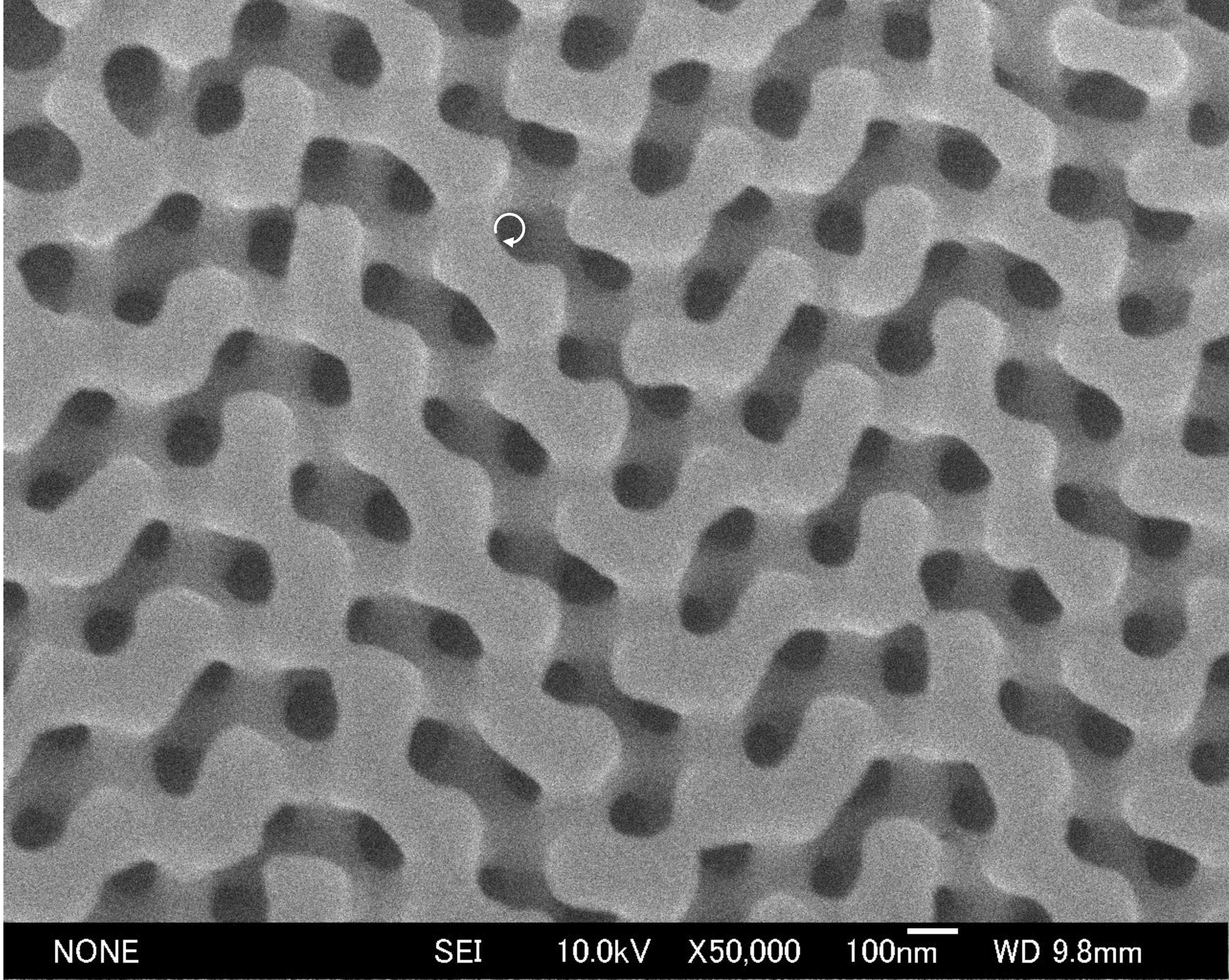

specimen No. 1
scale No. 11
domain No. 4
[111] lh spiral
LH gyroid

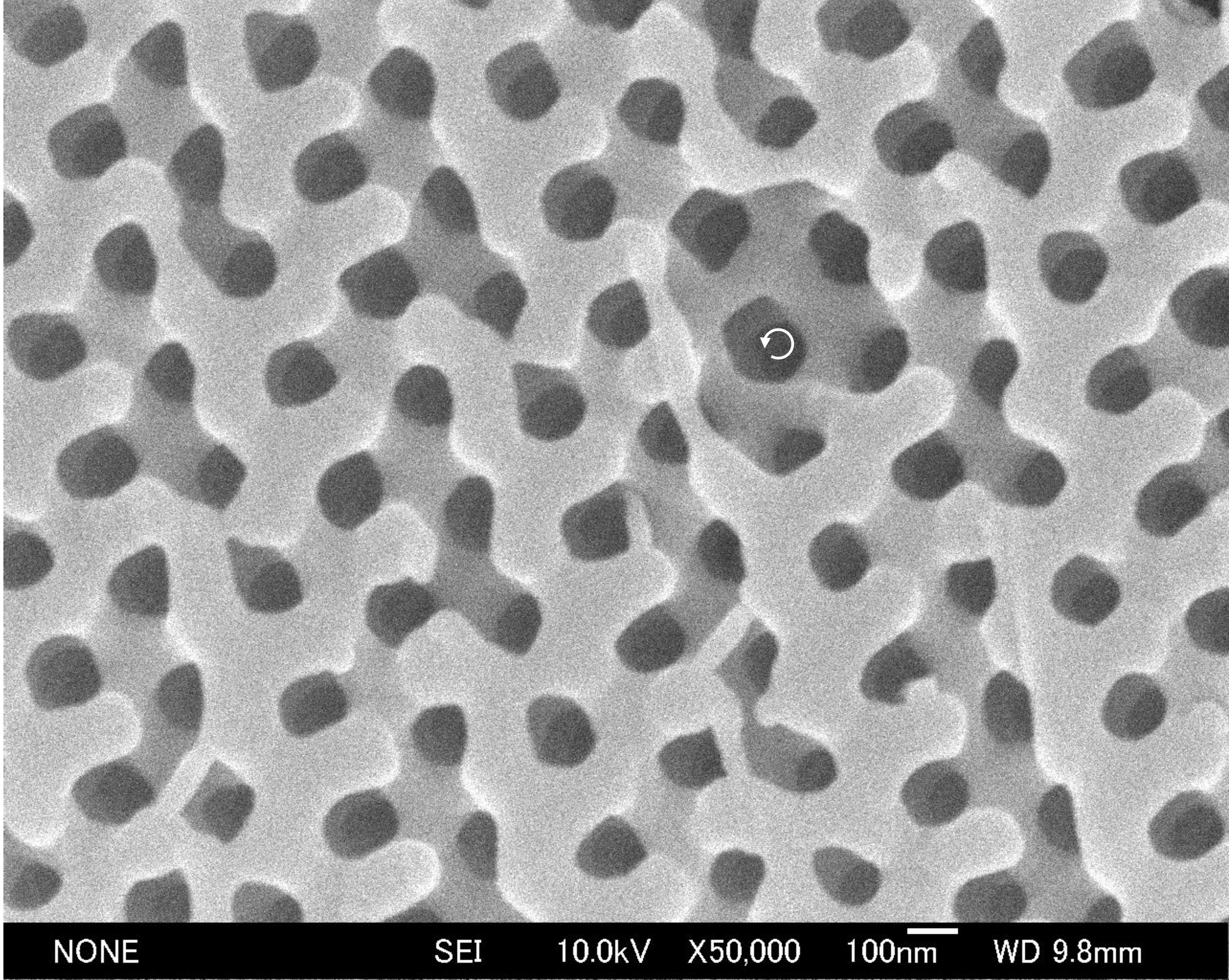

specimen No. 1
scale No. 11
domain No. 5
[100] rh spiral
LH gyroid

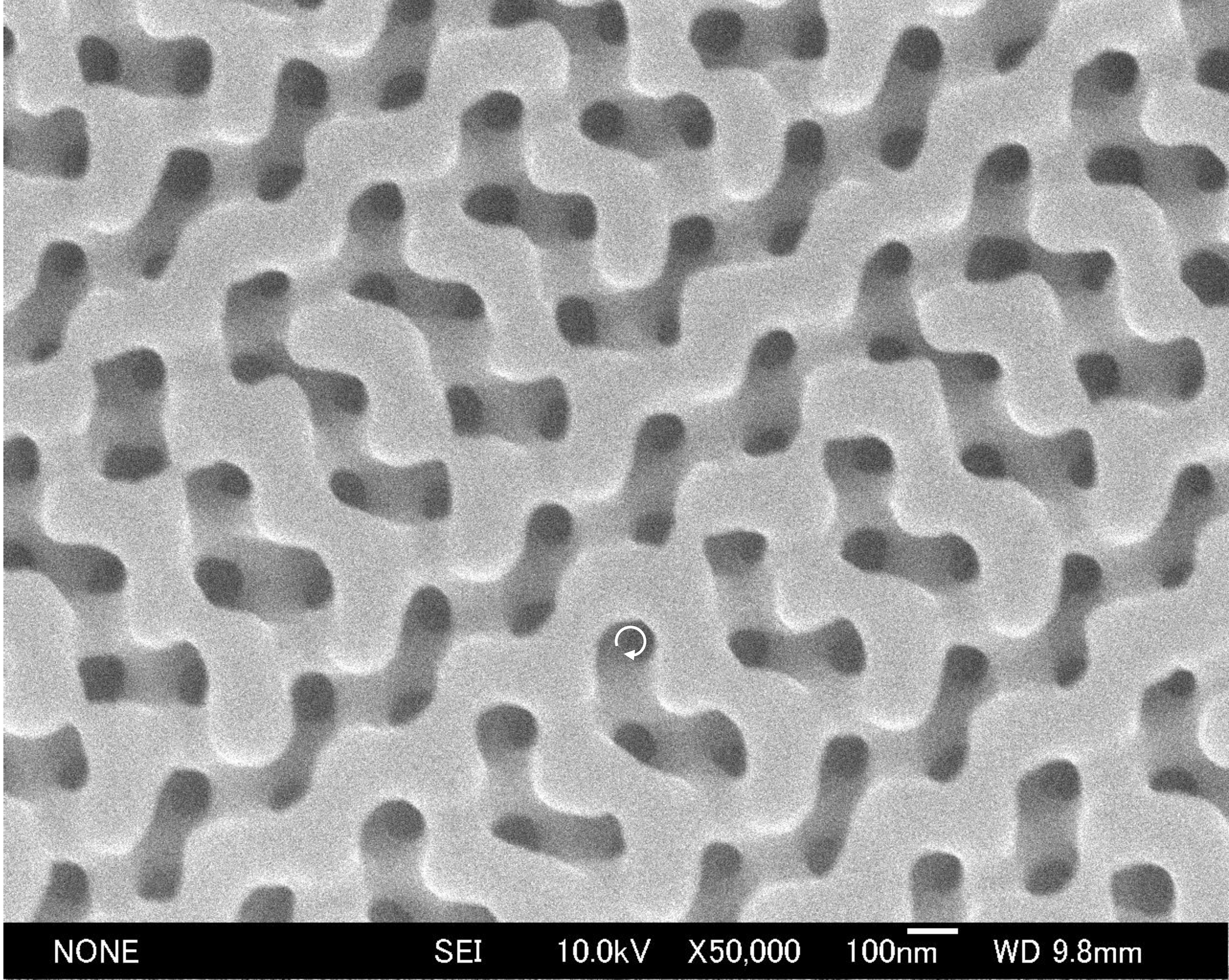

specimen No. 1
scale No. 11
domain No. 6
[111] lh spiral
LH gyroid

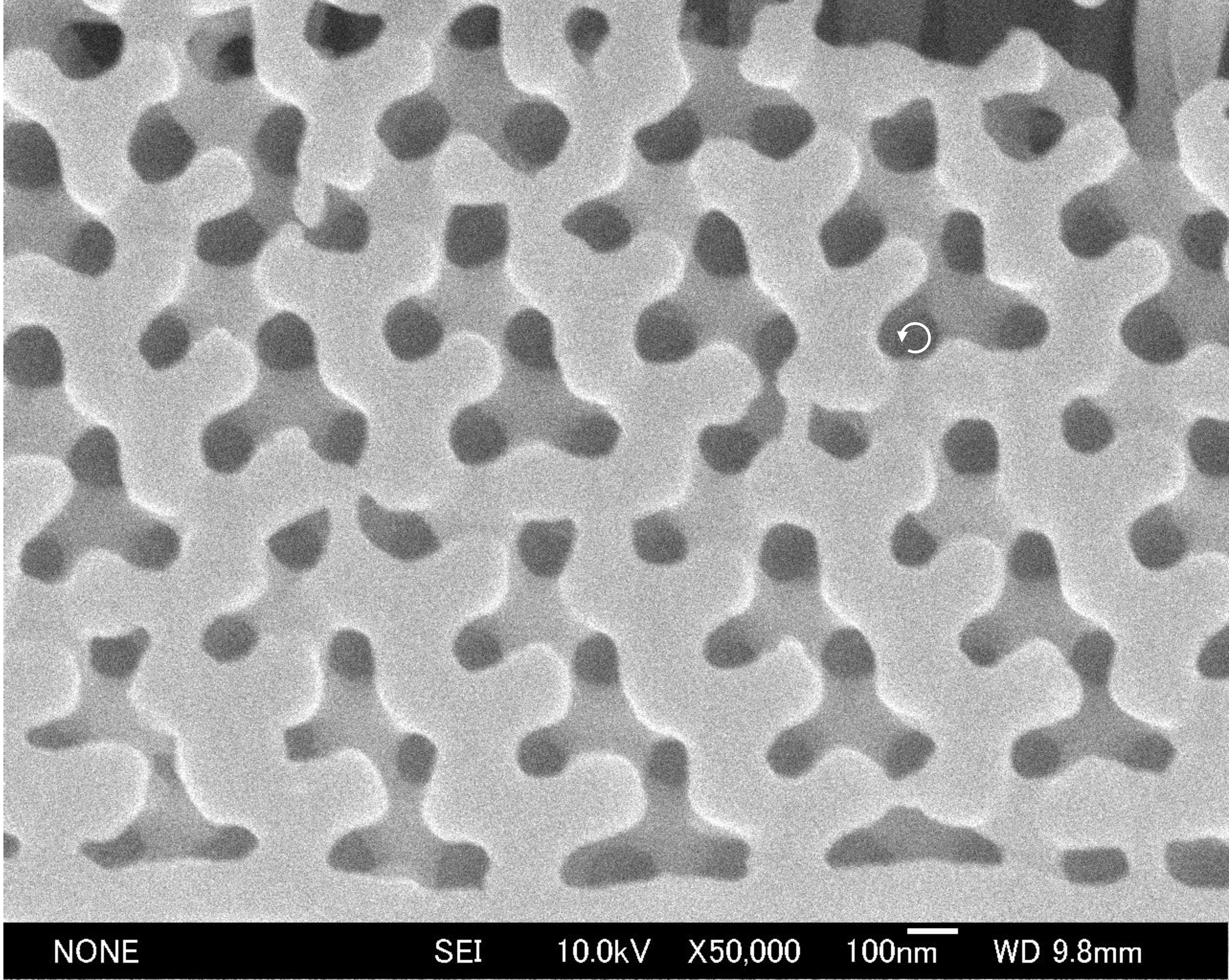

specimen No. 1
scale No. 12

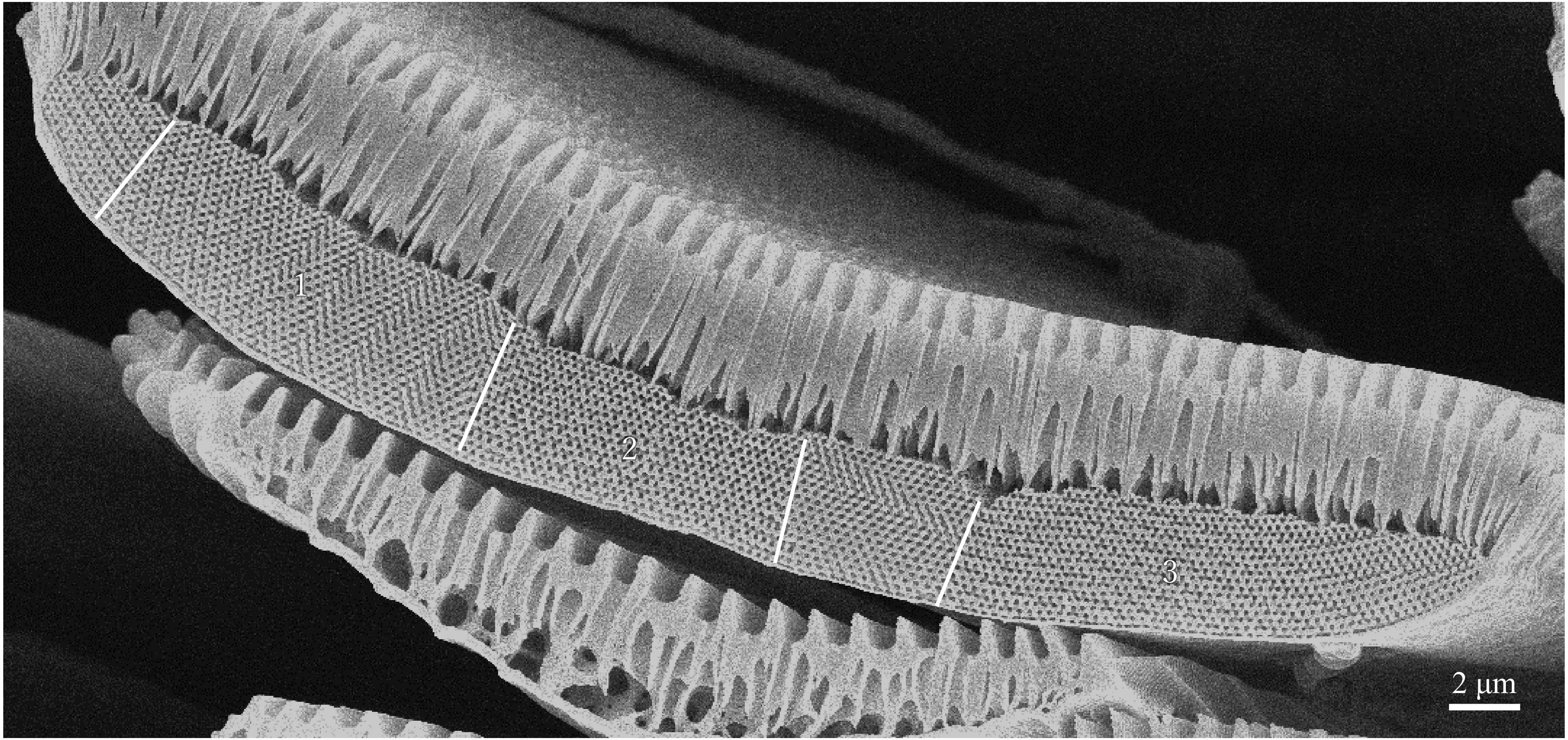

specimen No. 1
scale No. 12
domain No. 1
[111] lh spiral
LH gyroid

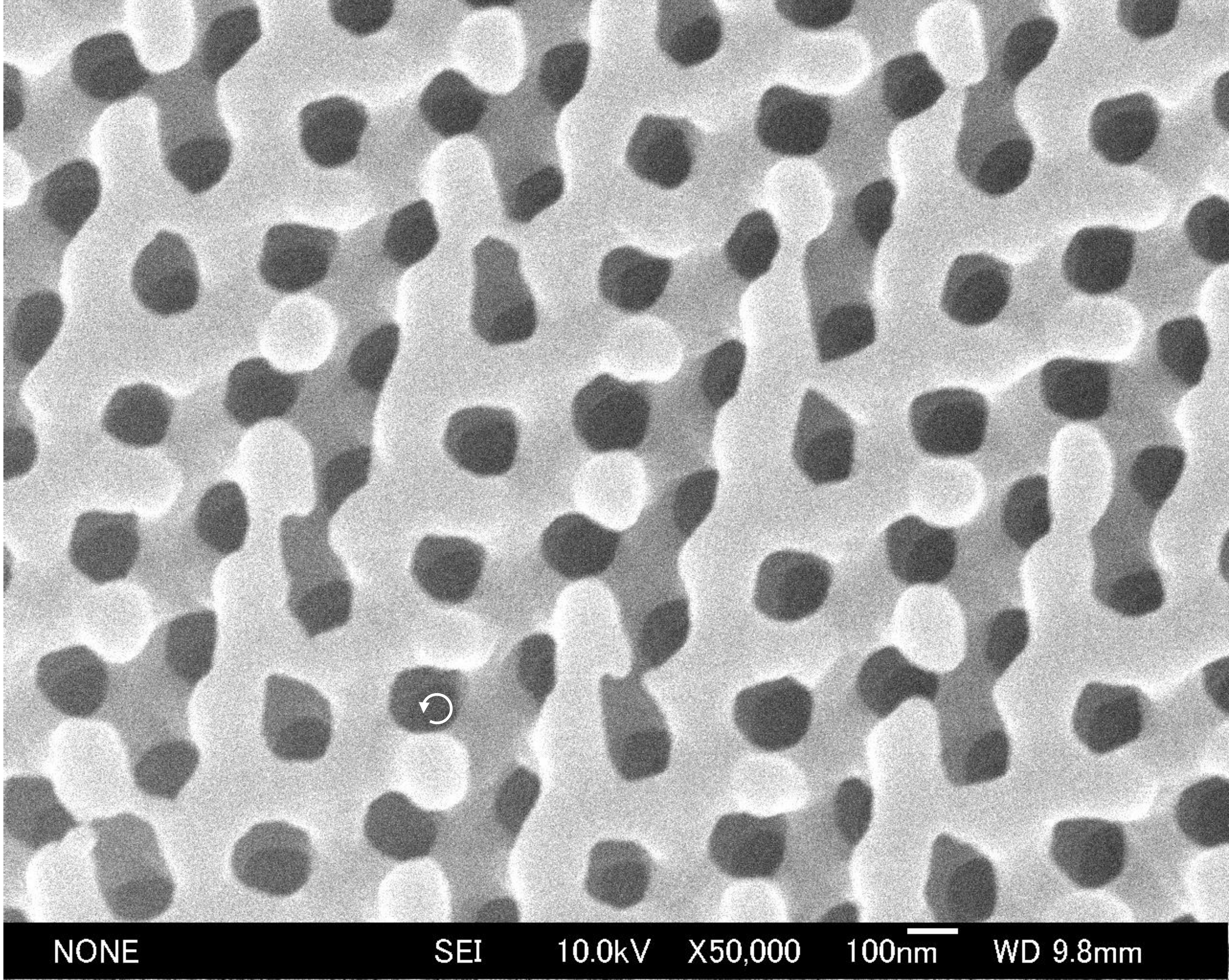

specimen No. 1
scale No. 12
domain No. 2
[111] lh spiral
LH gyroid

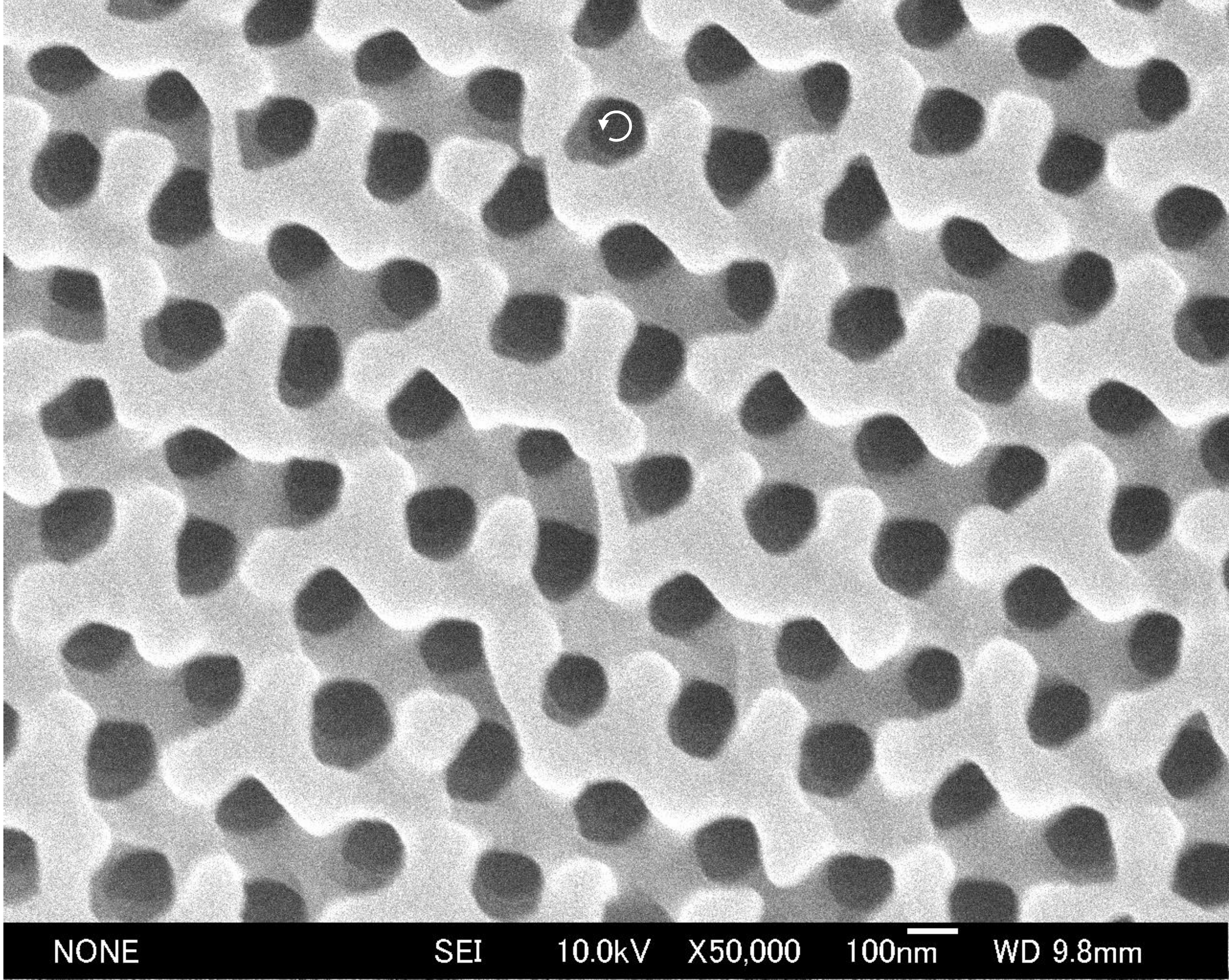

specimen No. 1
scale No. 12
domain No. 3
[111] lh spiral
LH gyroid

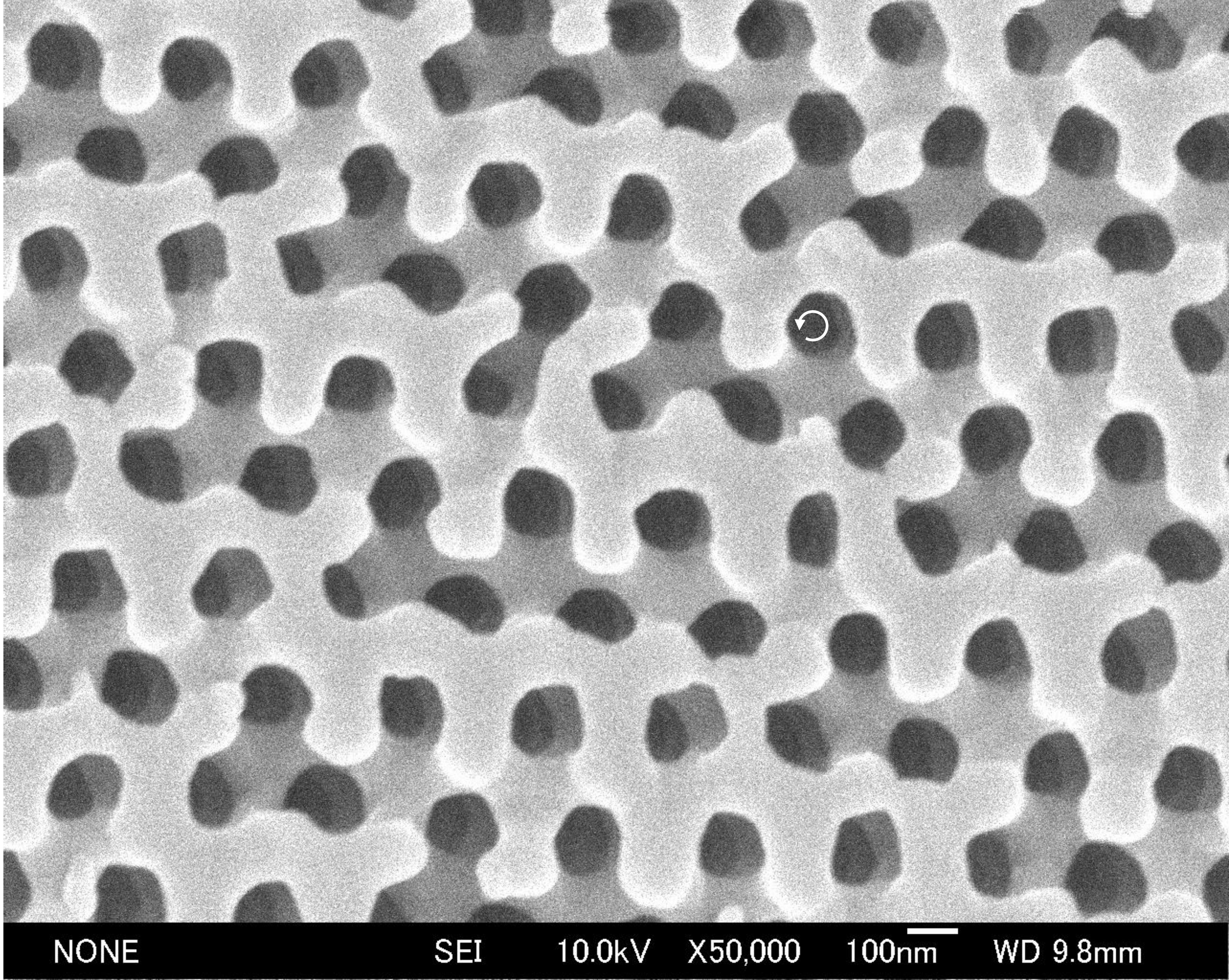

specimen No. 1
scale No. 13

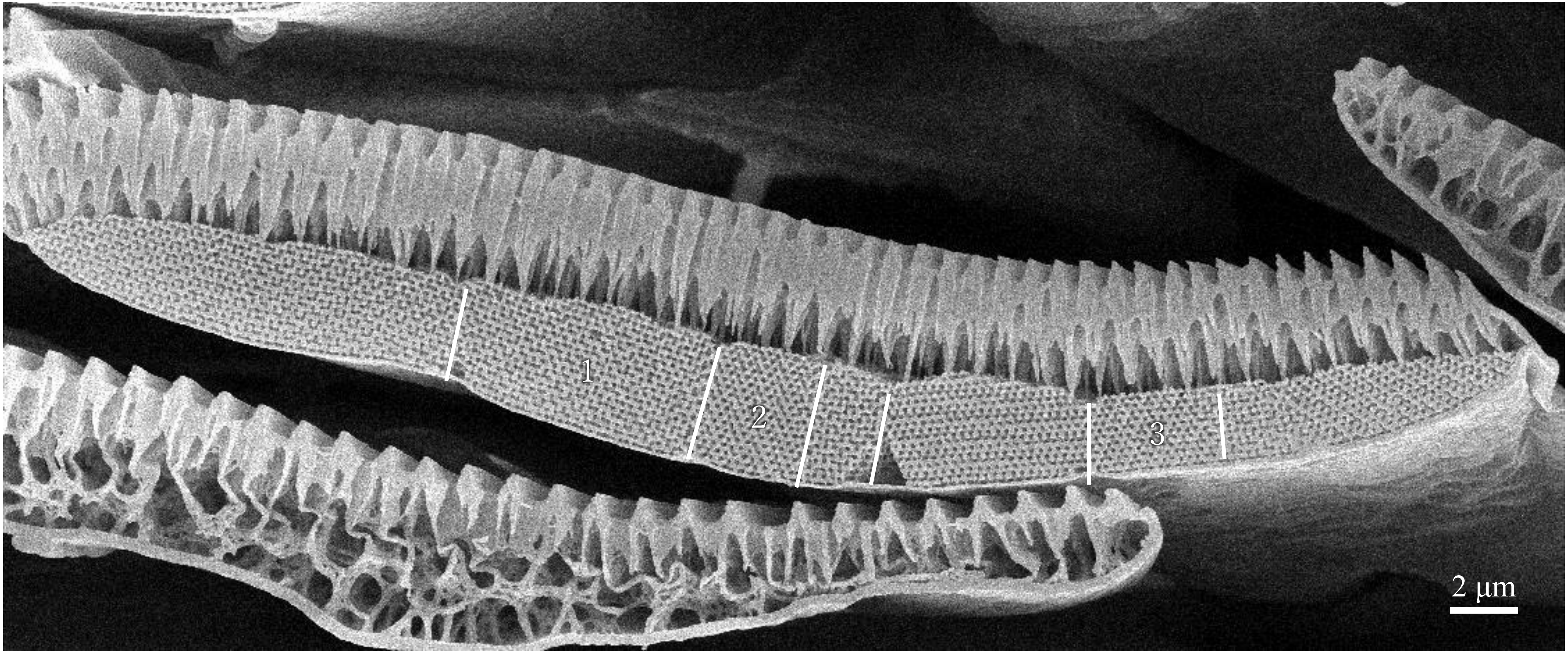

specimen No. 1
scale No. 13
domain No. 1
[100] rh spiral
LH gyroid

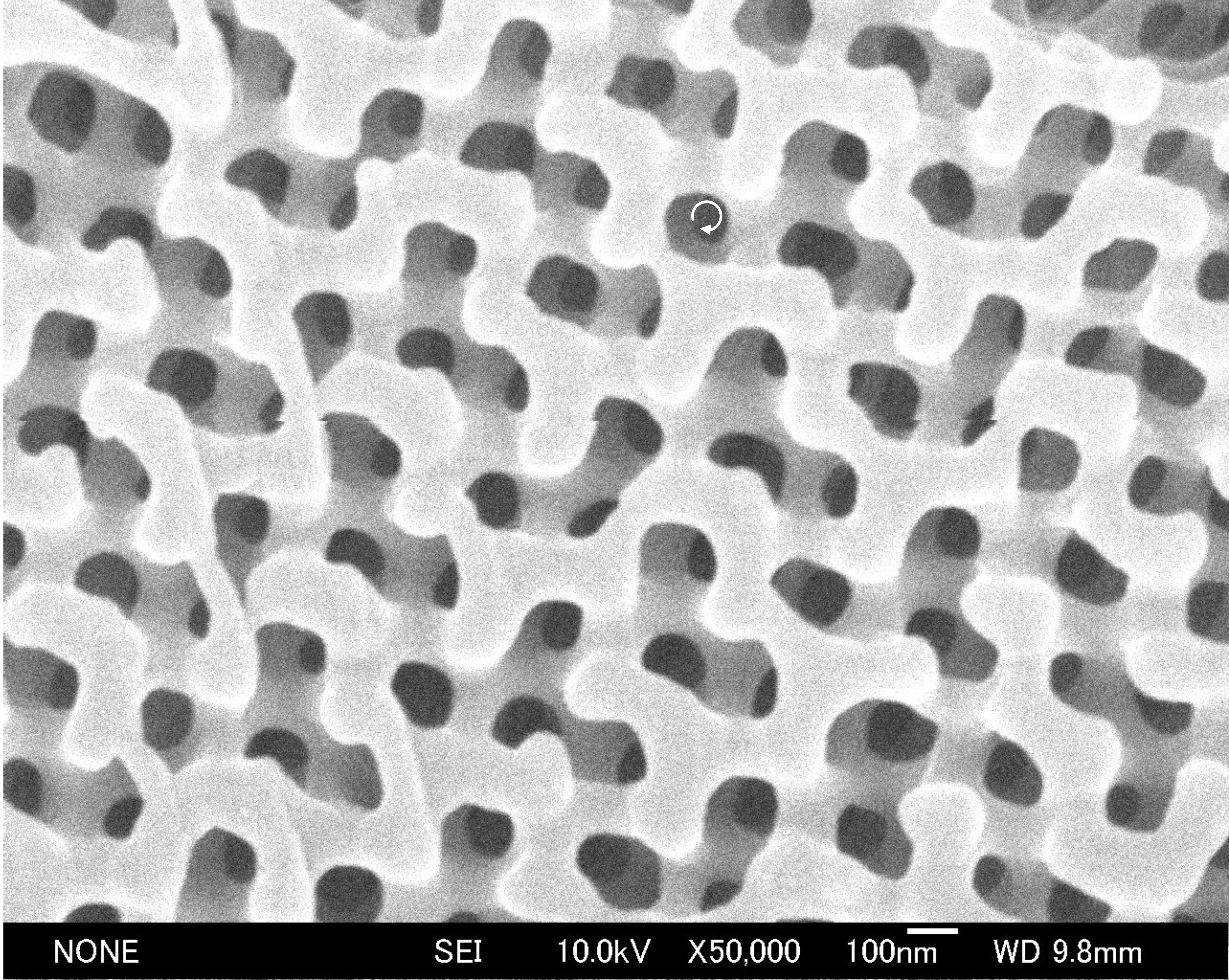

specimen No. 1
scale No. 13
domain No. 2
[111] lh spiral
LH gyroid

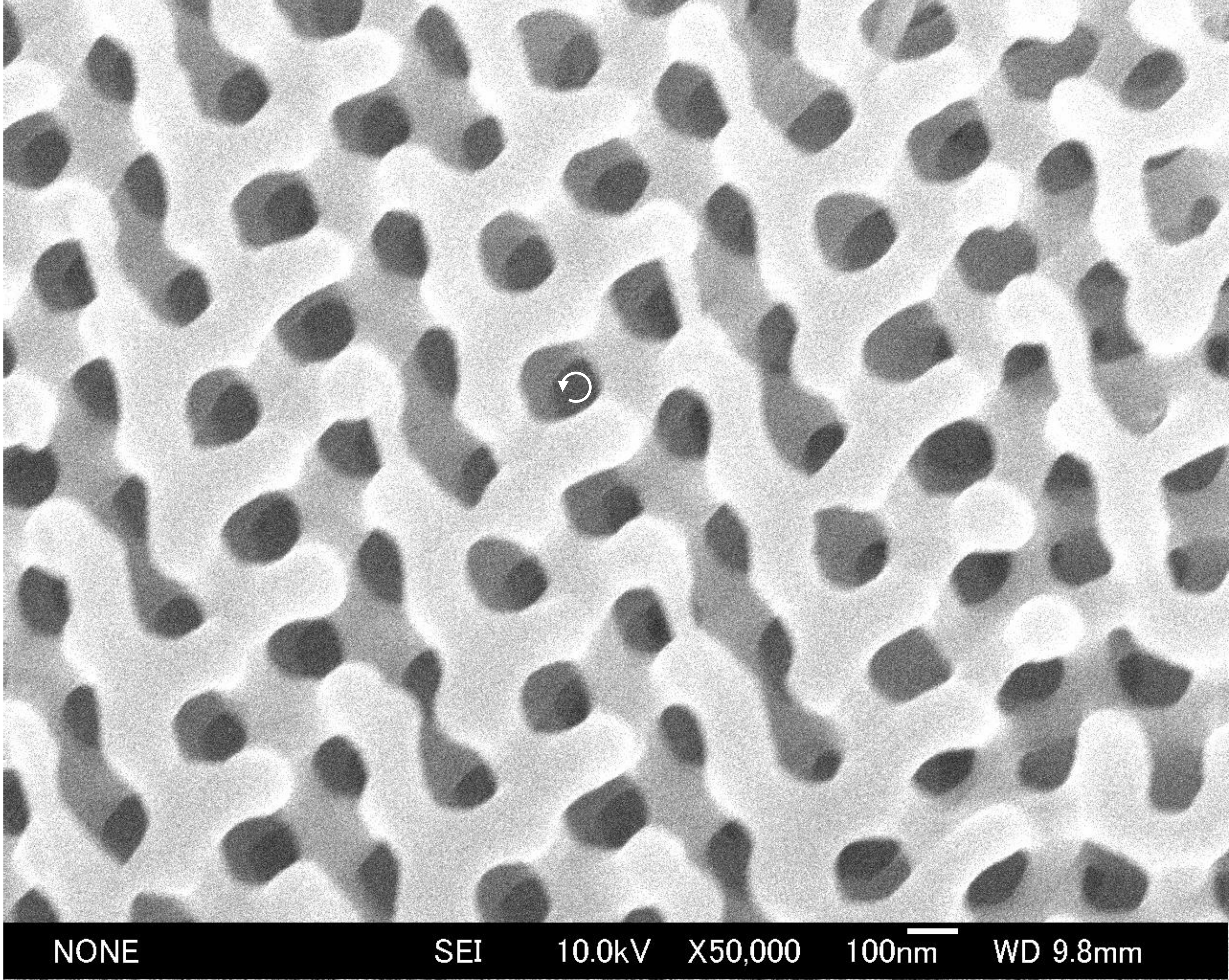

specimen No. 1
scale No. 13
domain No. 3
[111] rh spiral
RH gyroid

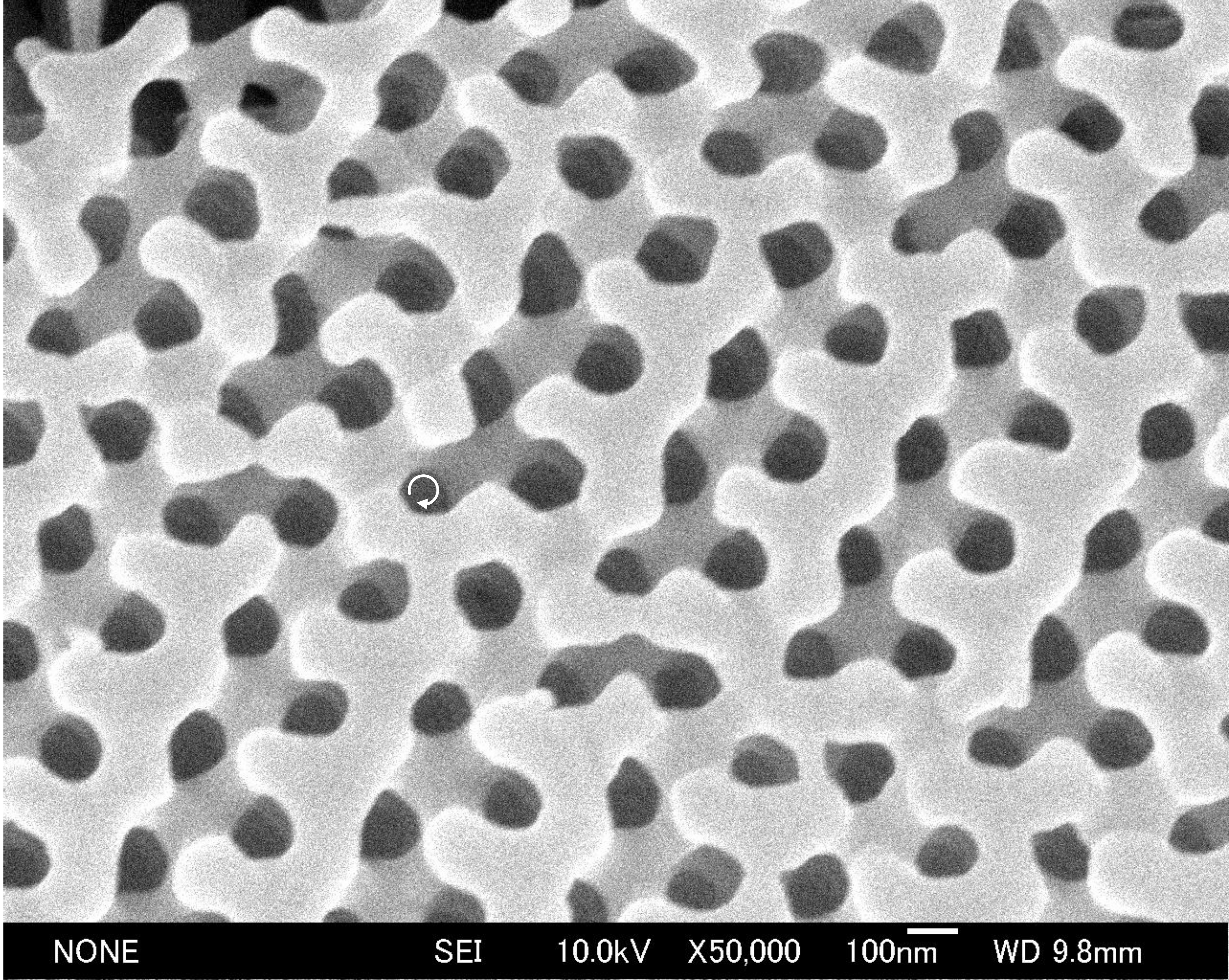

specimen No. 1
scale No. 14

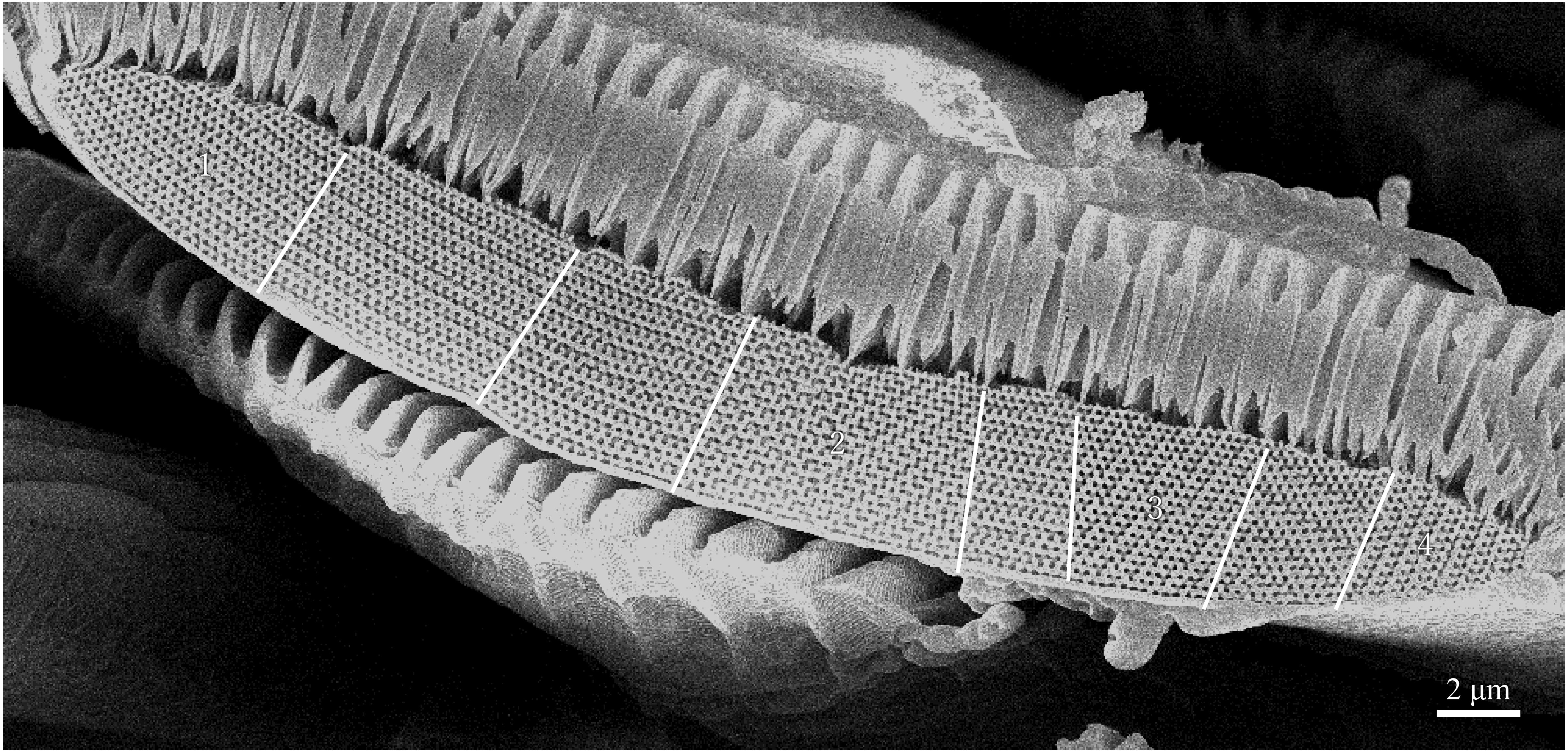

specimen No. 1
scale No. 14
domain No. 1
[111] lh spiral
LH gyroid

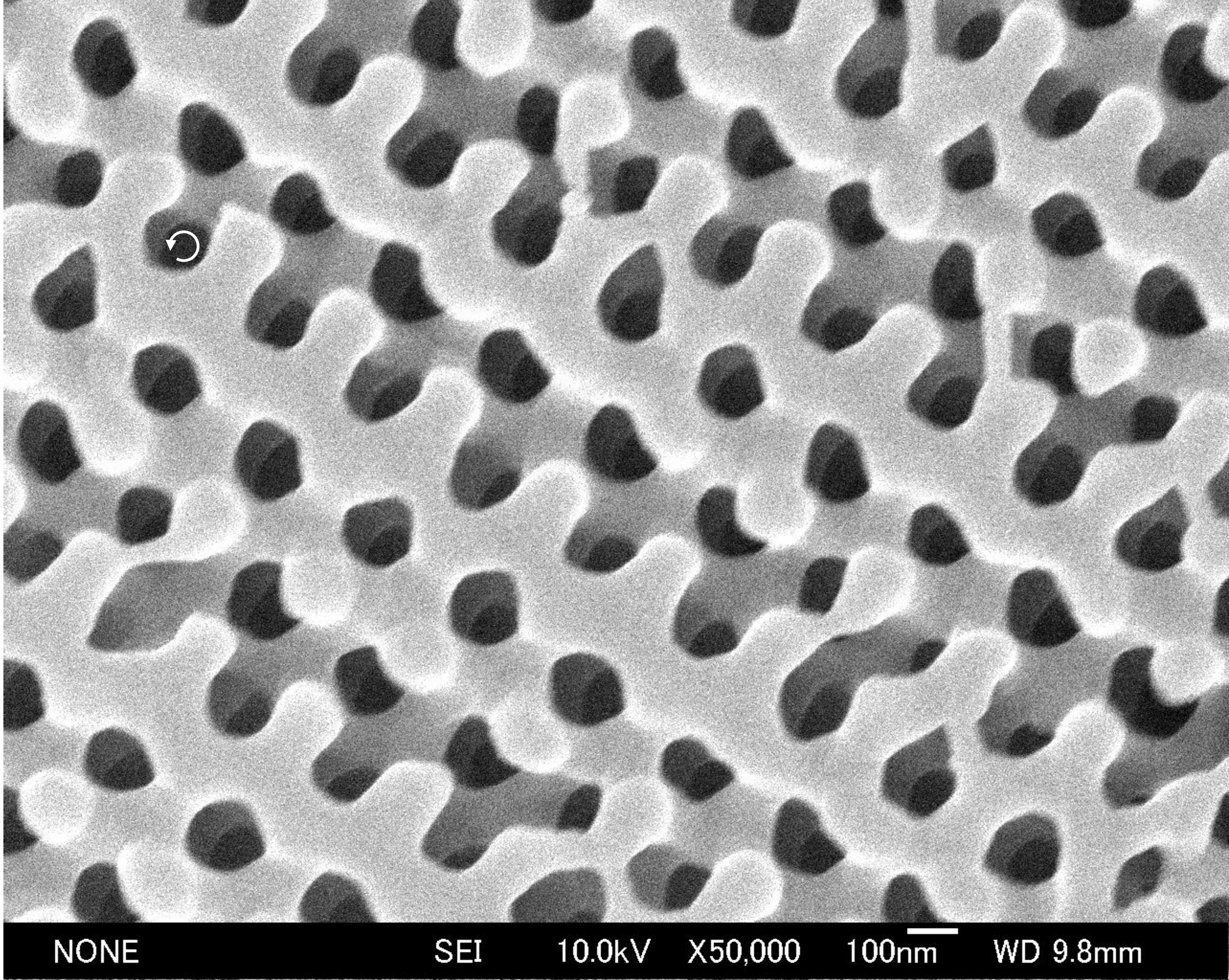

specimen No. 1
scale No. 14
domain No. 2
[100] rh spiral
LH gyroid

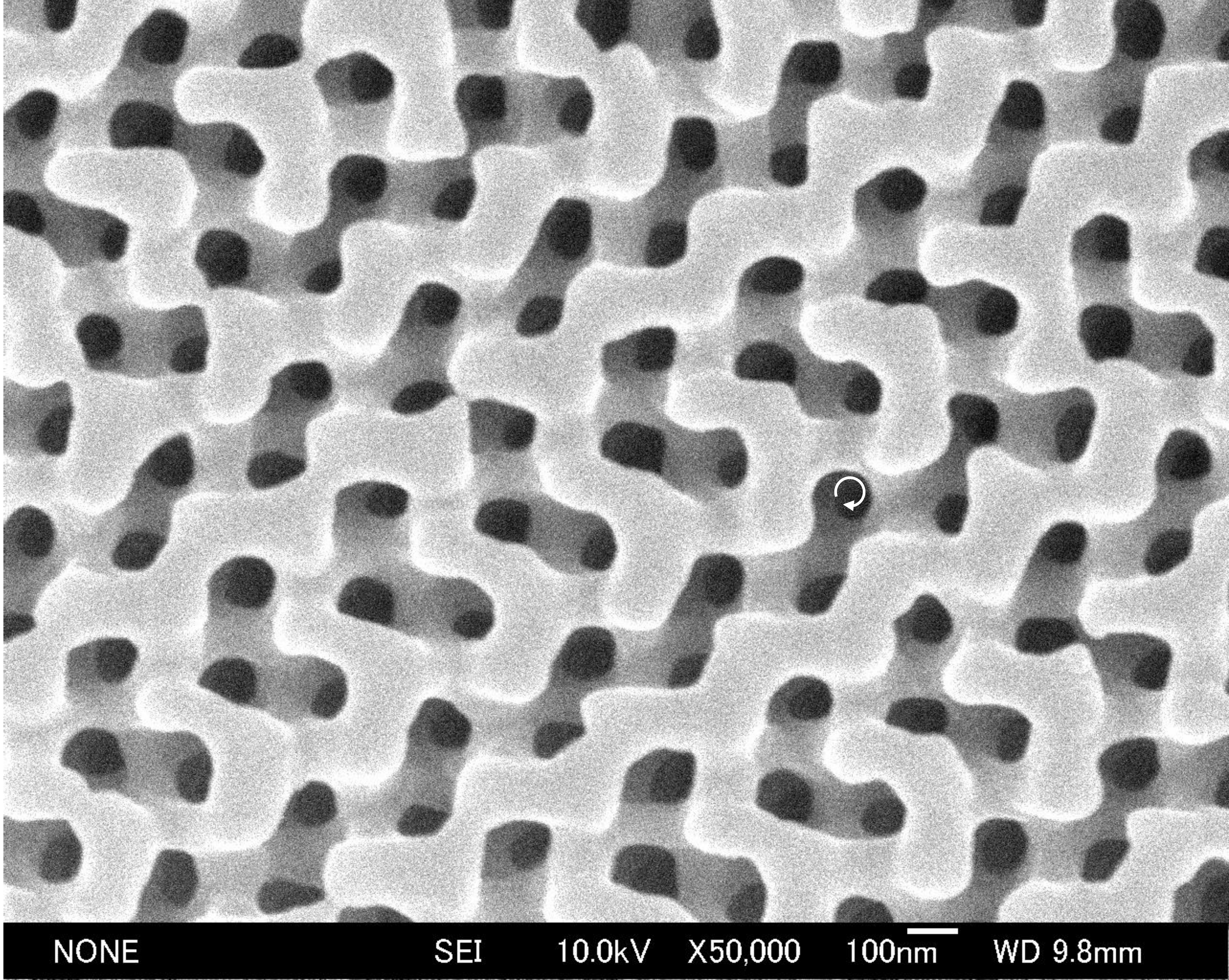

specimen No. 1
scale No. 14
domain No. 3
[111] lh spiral
LH gyroid

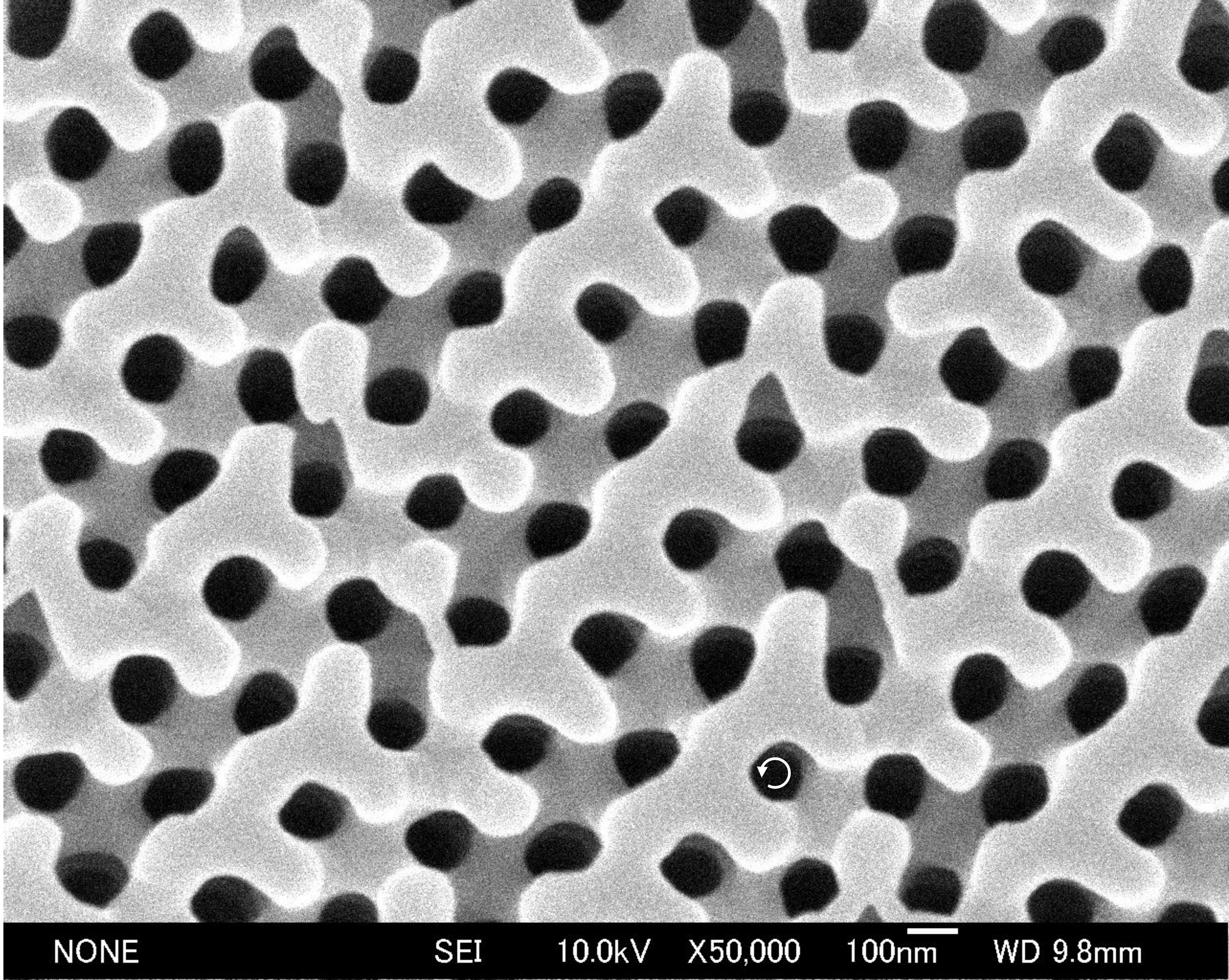

specimen No. 1
scale No. 14
domain No. 4
[111] lh spiral
LH gyroid

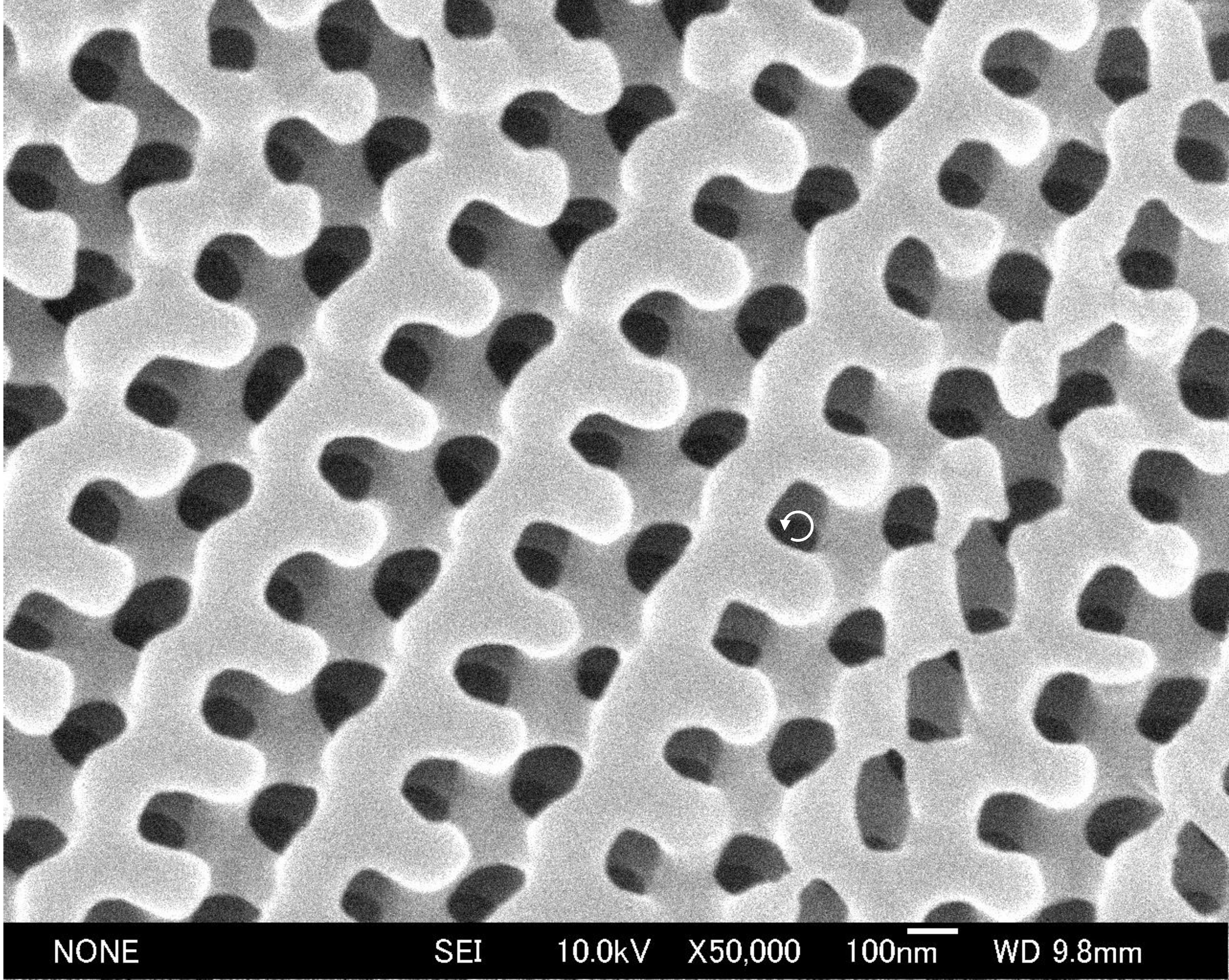

specimen No. 2
scale No. 1

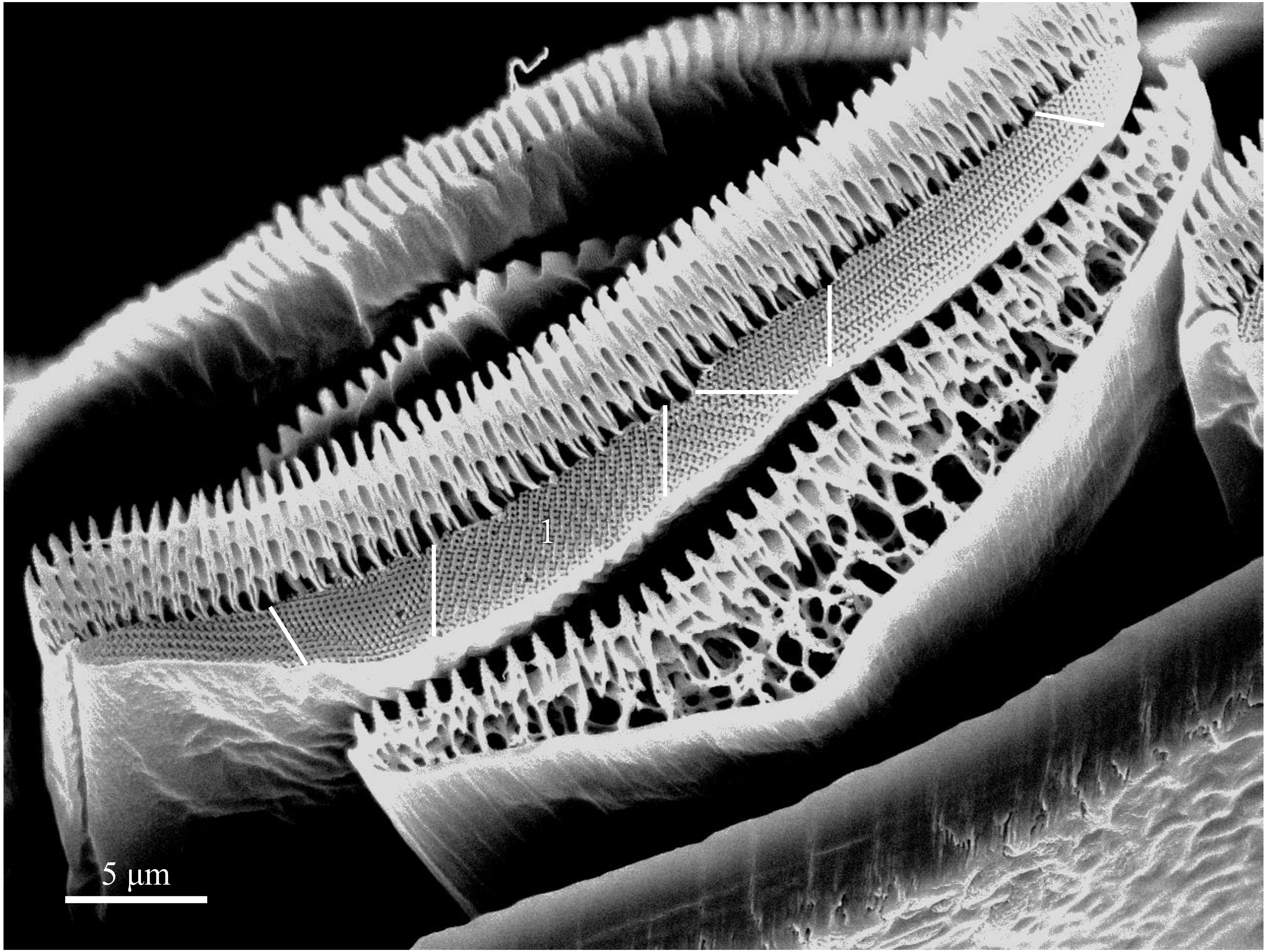

specimen No. 2
scale No. 1
domain No. 1
[100] rh spiral
LH gyroid

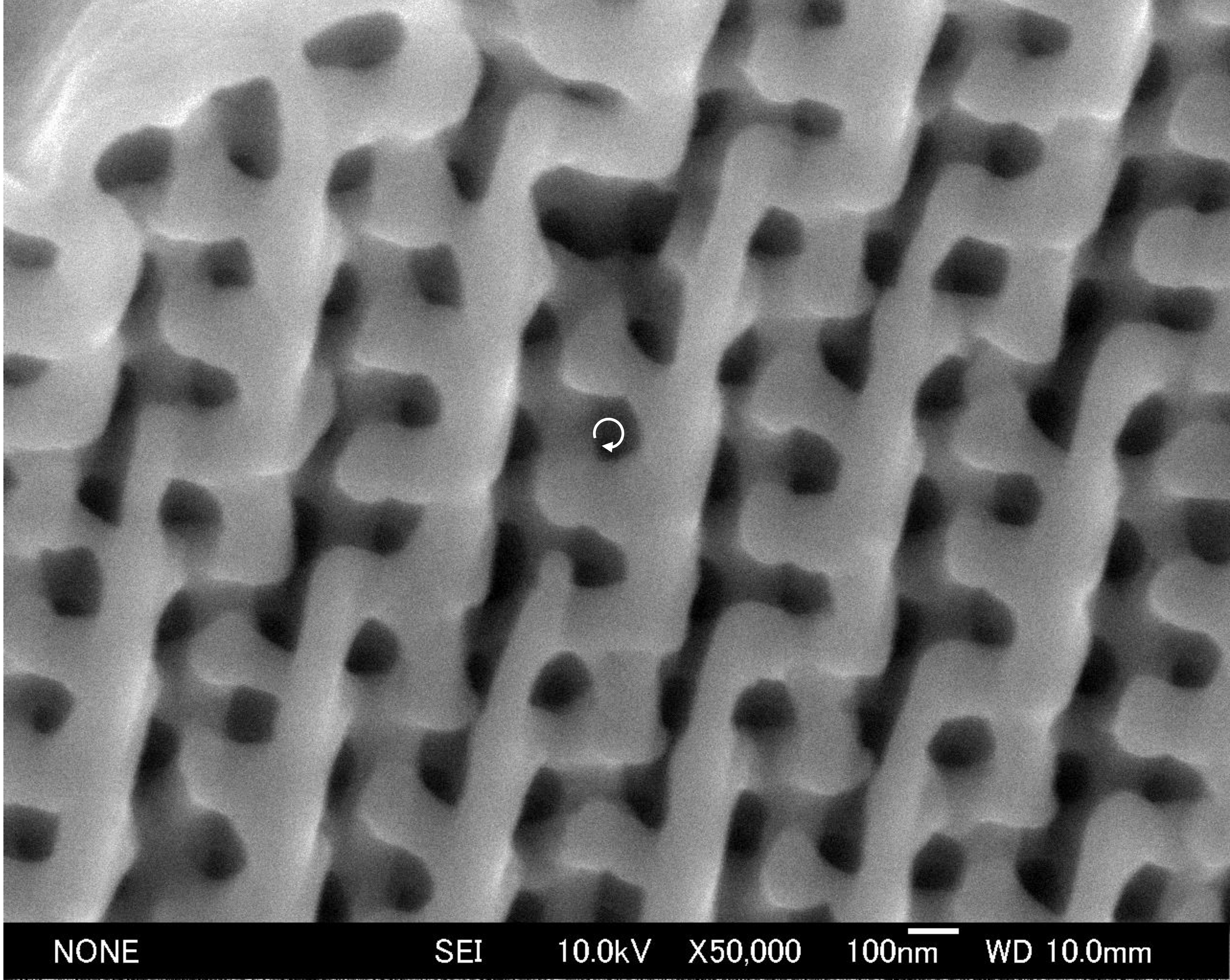

specimen No. 2
scale No. 2

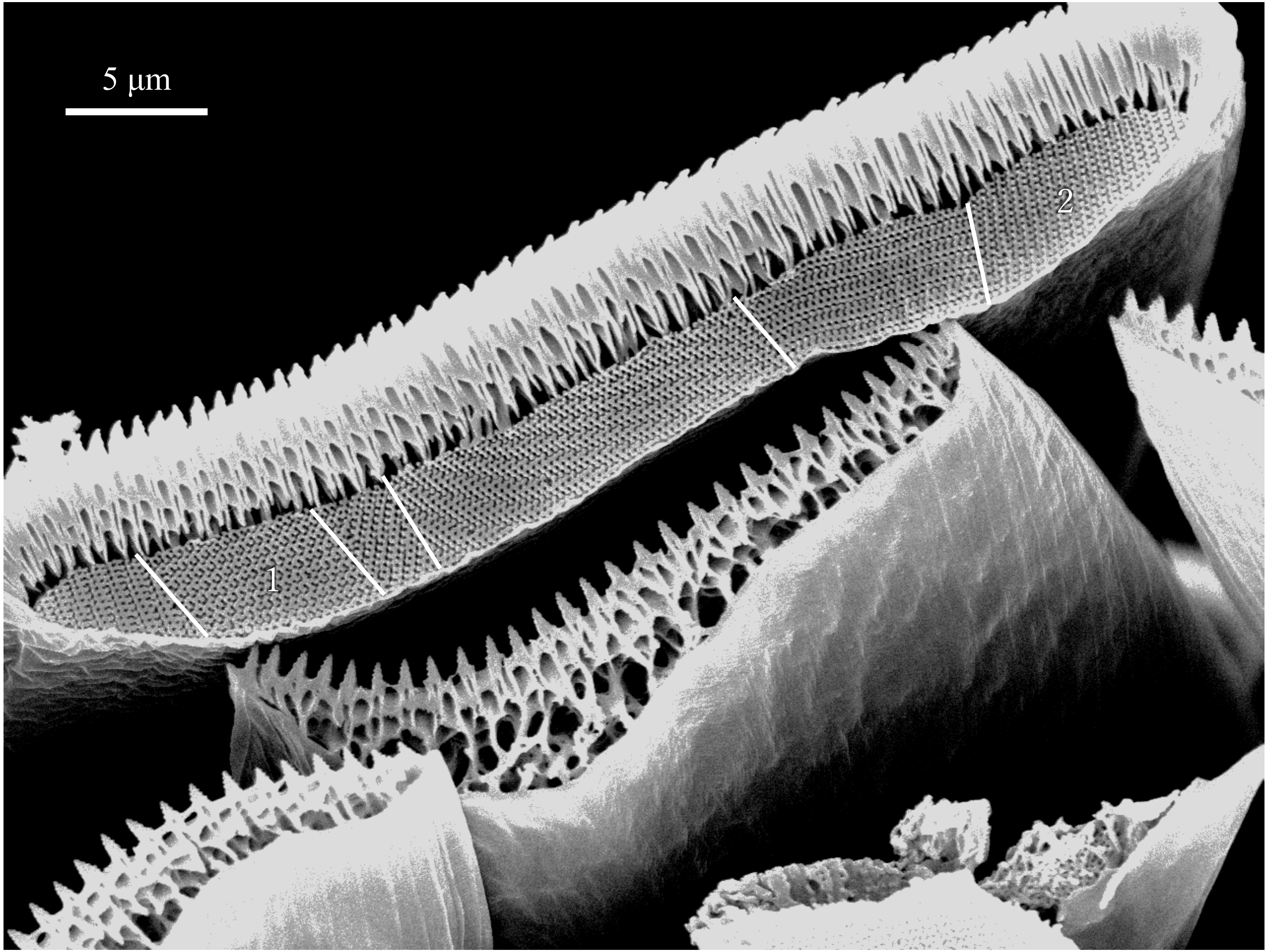

specimen No. 2
scale No. 2
domain No. 1
[100] rh spiral
LH gyroid

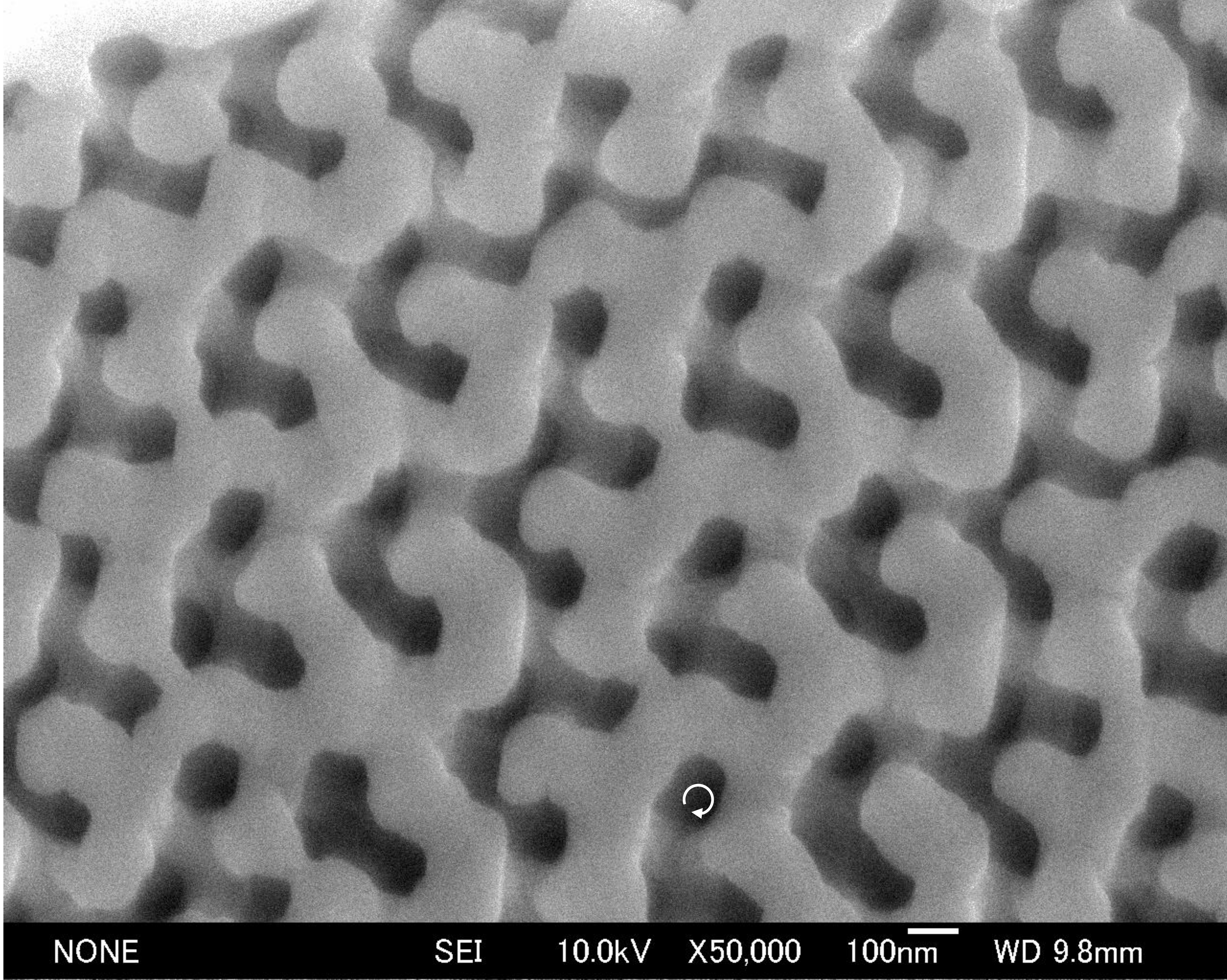

specimen No. 2
scale No. 2
domain No. 2
[111] lh spiral
LH gyroid

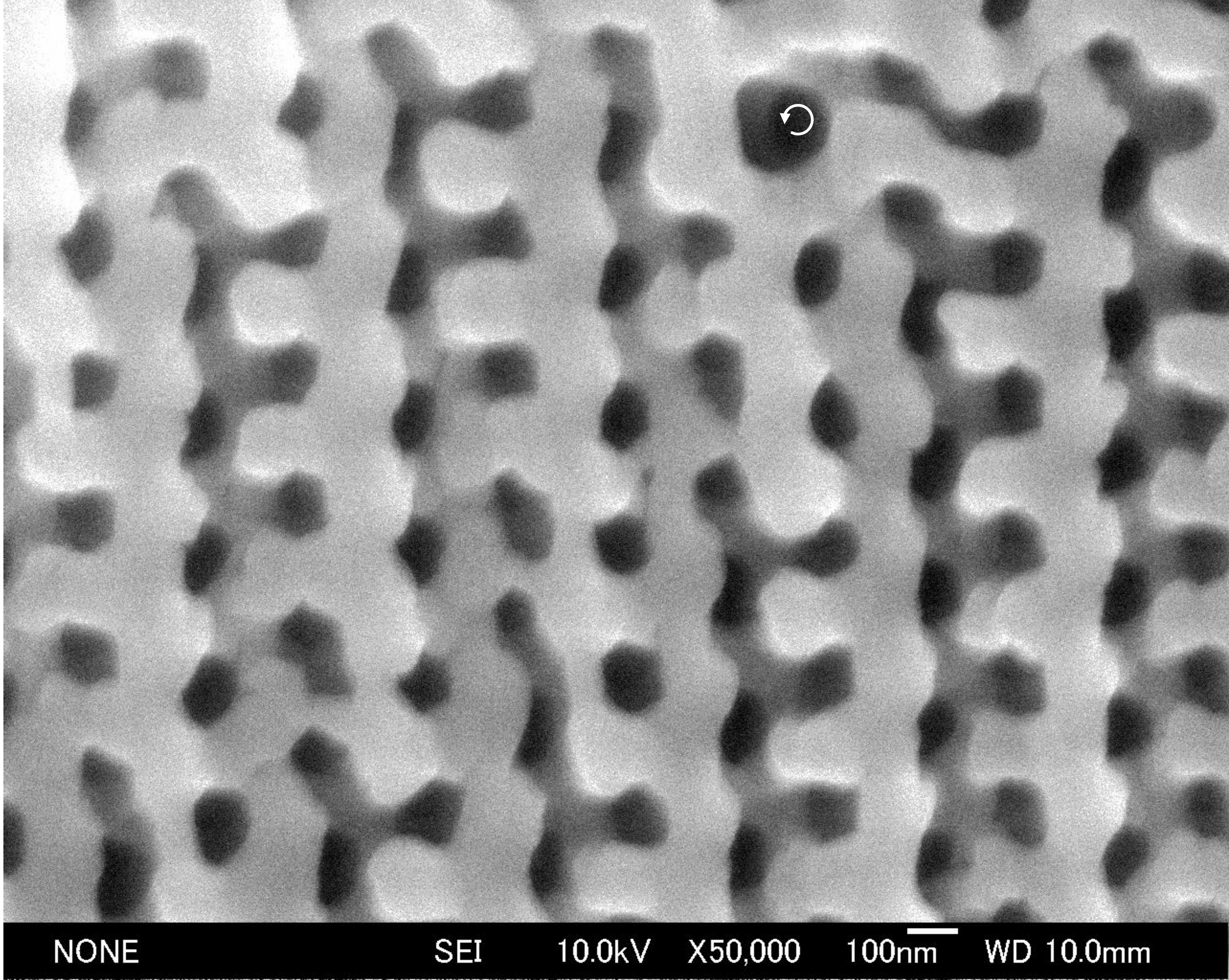

specimen No. 2
scale No. 3

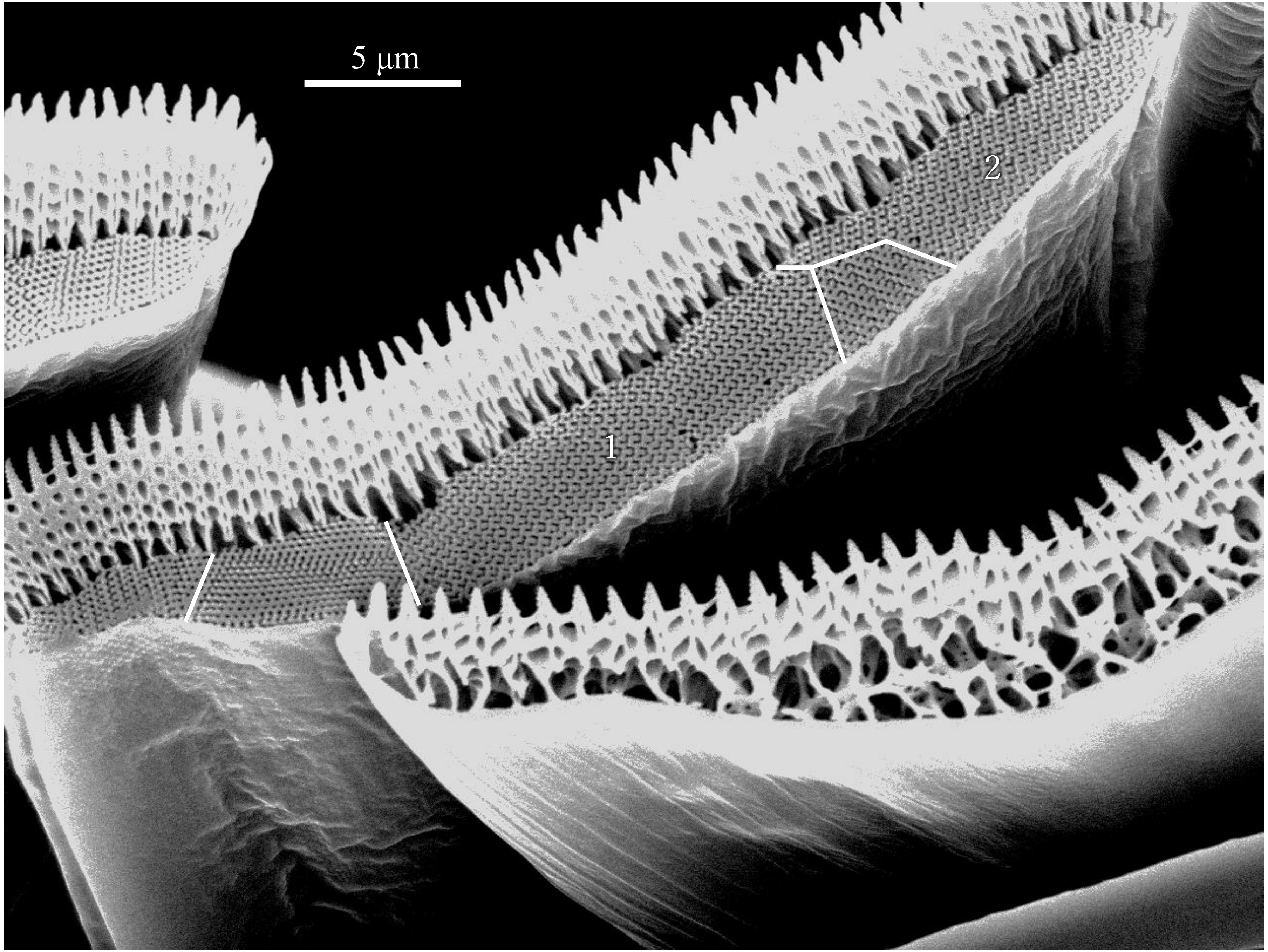

specimen No. 2
scale No. 3
domain No. 1
[100] rh spiral
LH gyroid

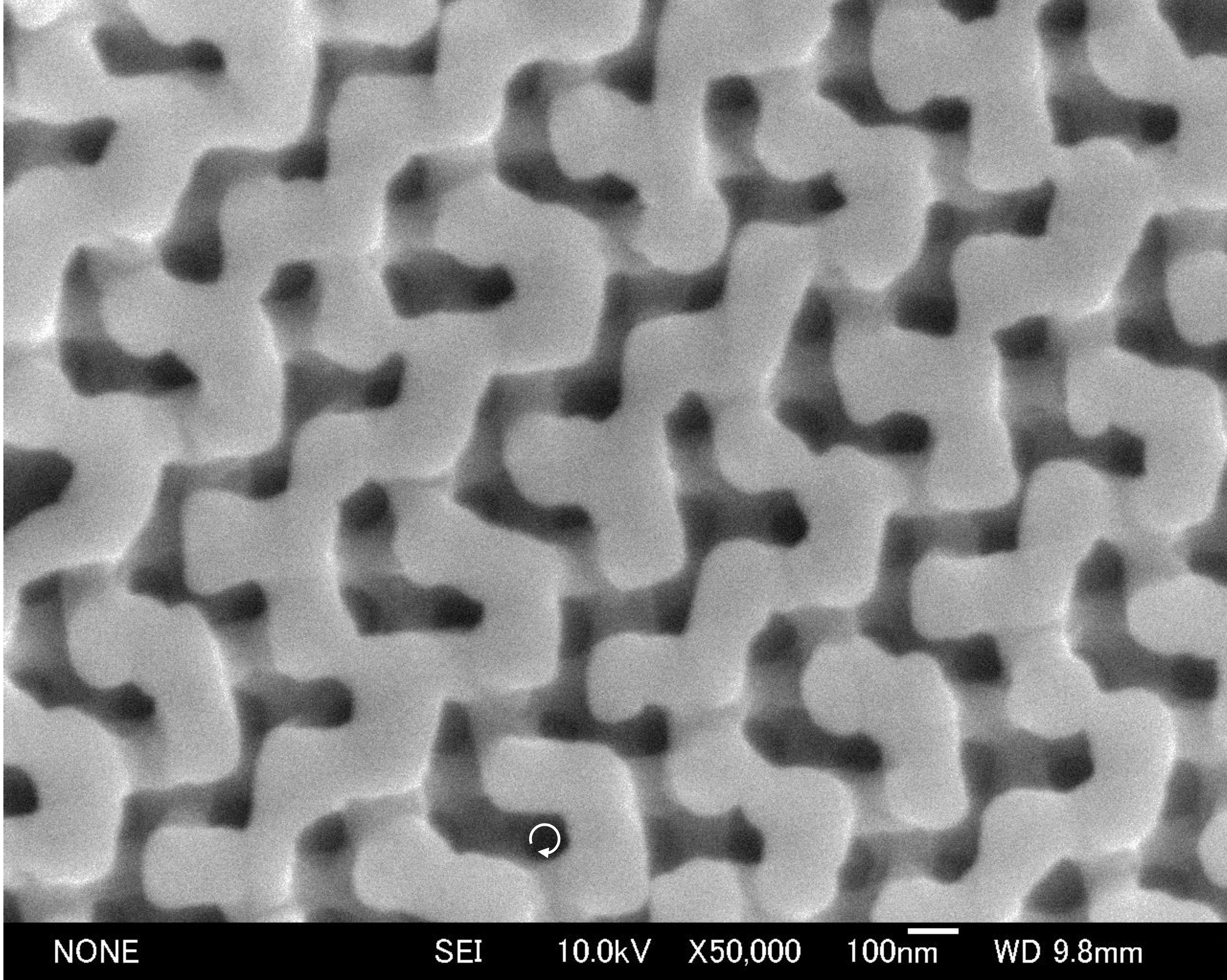

specimen No. 2
scale No. 3
domain No. 2
[100] rh spiral
LH gyroid

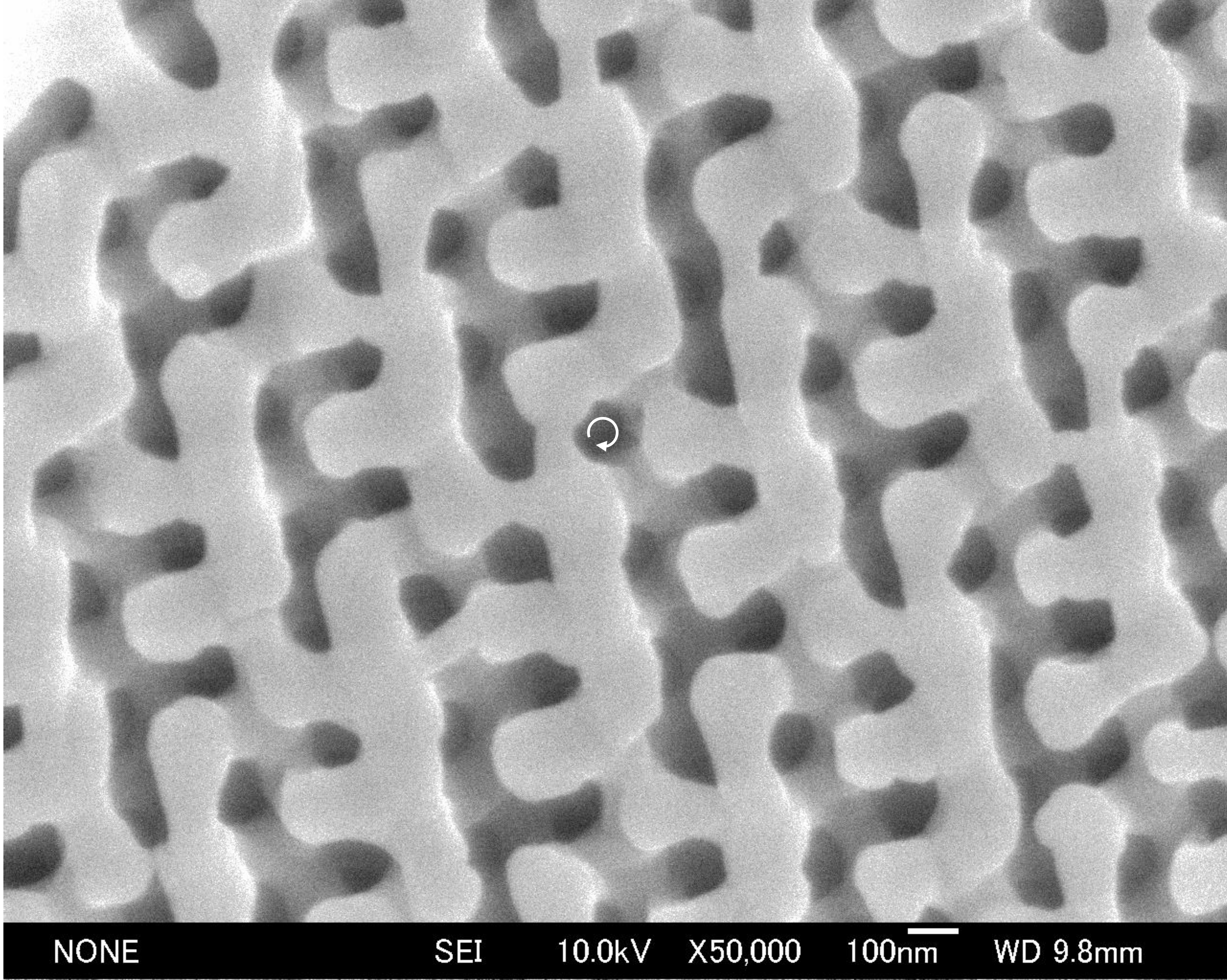

specimen No. 2
scale No. 4

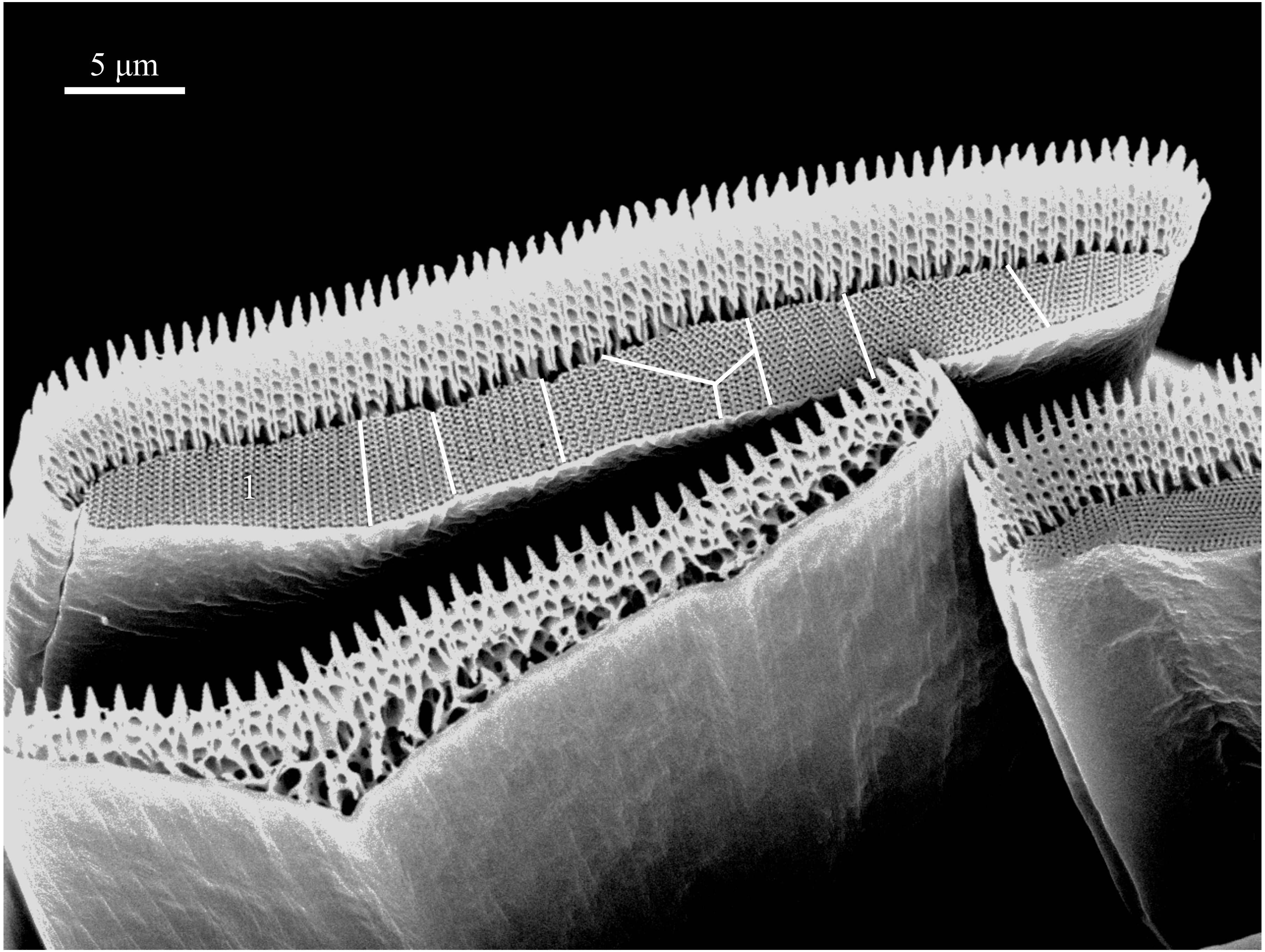

specimen No. 2
scale No. 4
domain No. 1
[111] lh spiral
LH gyroid

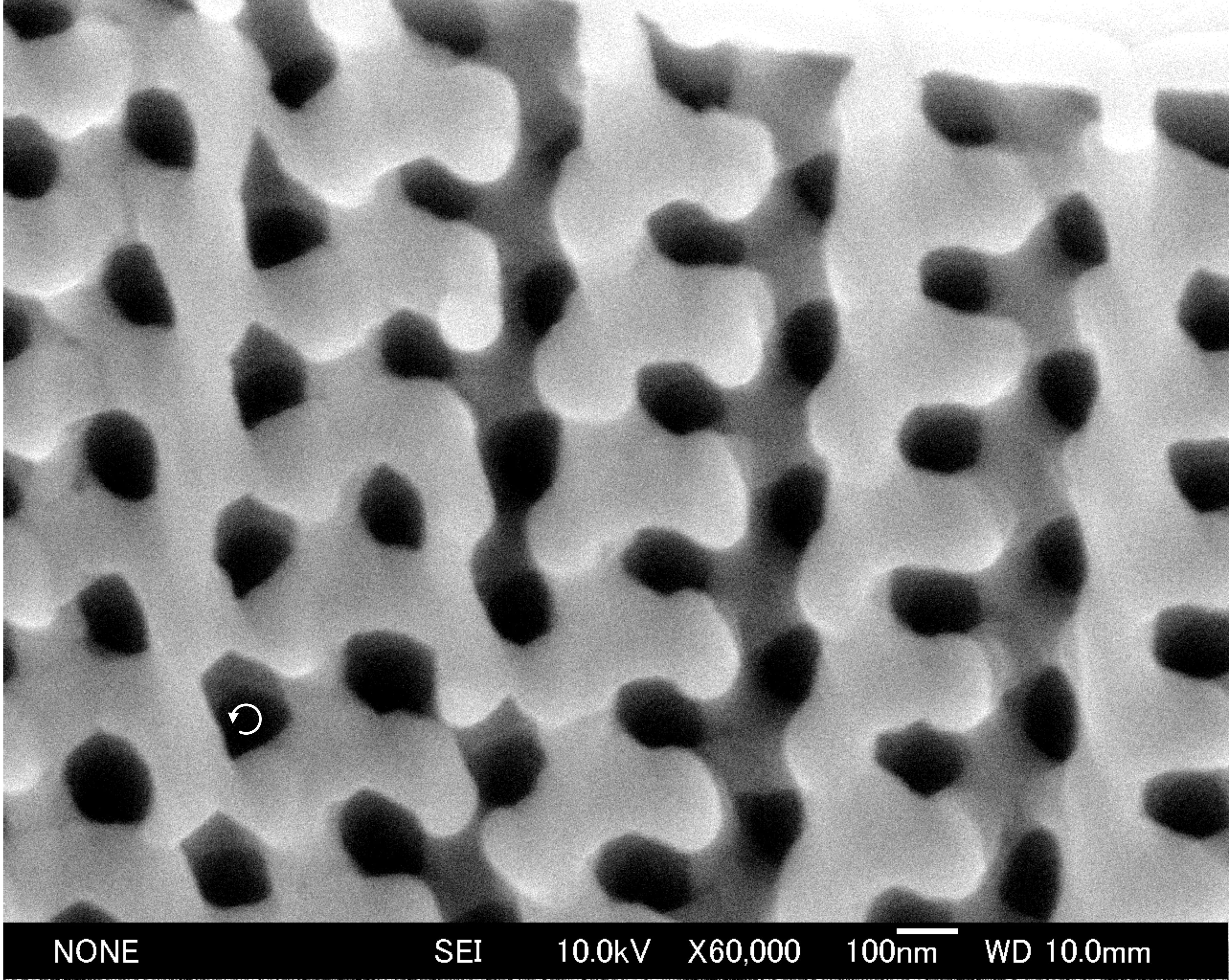

specimen No. 2
scale No. 5

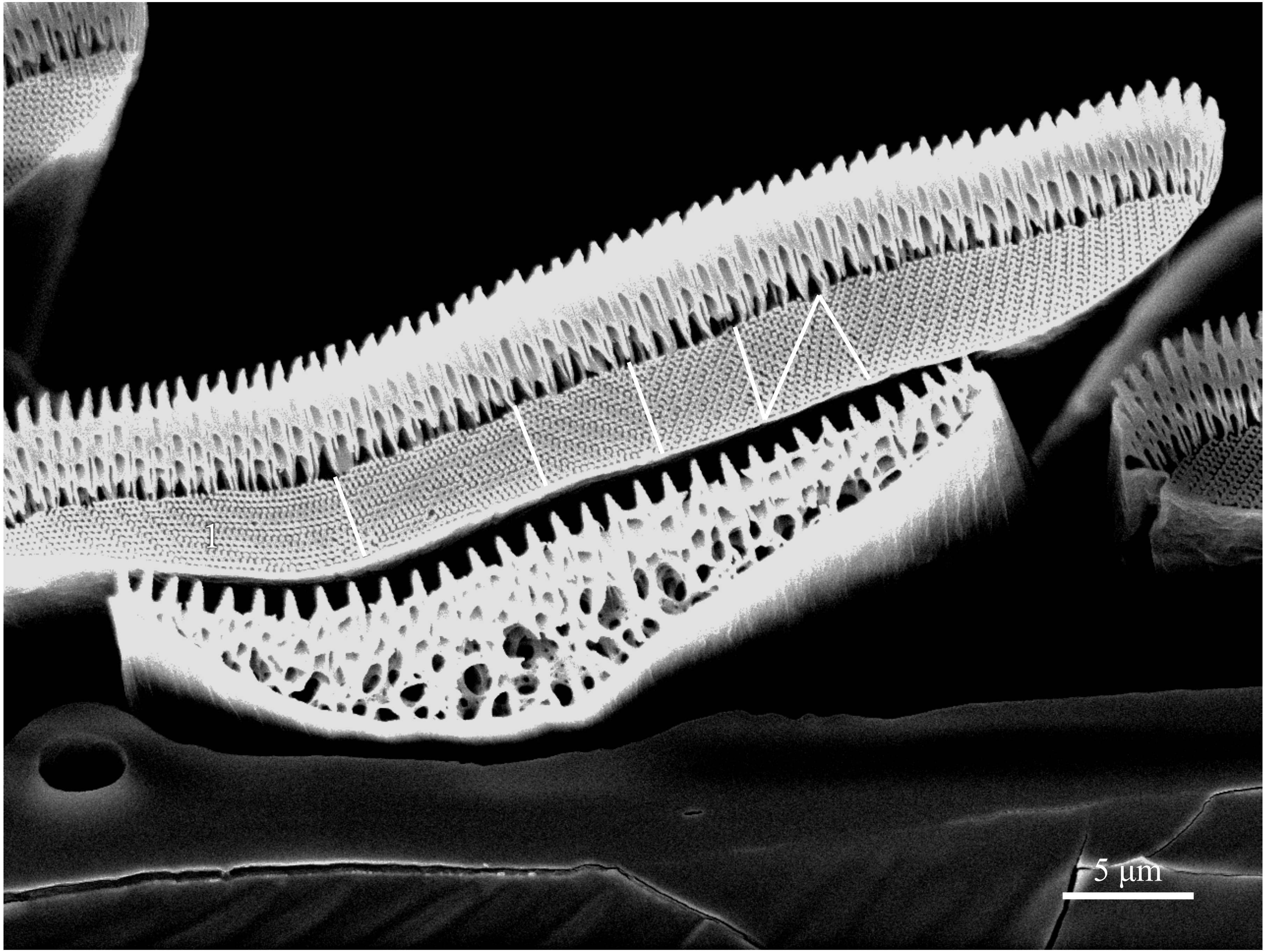

specimen No. 2
scale No. 5
domain No. 1
[111] lh spiral
LH gyroid

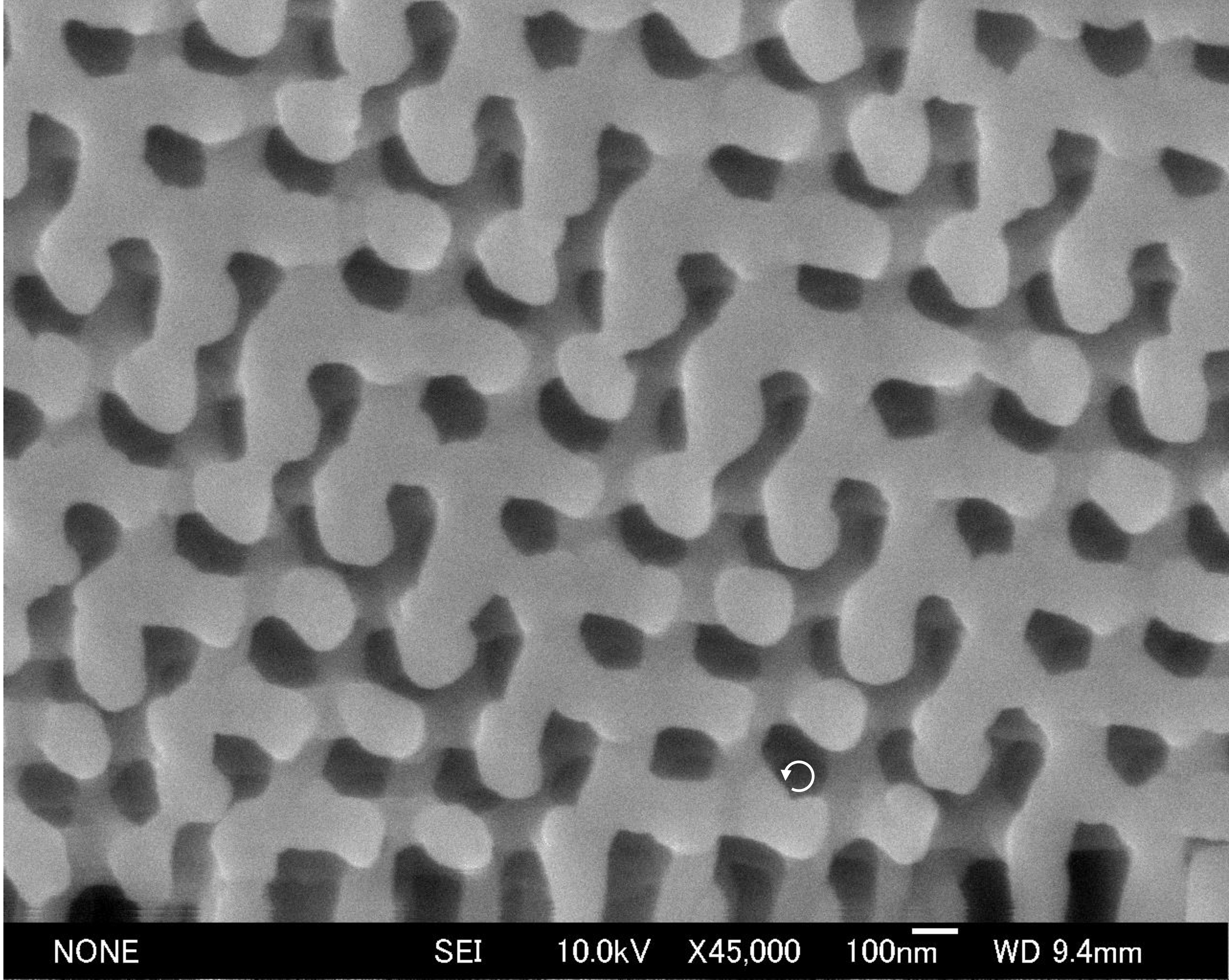

specimen No. 2
scale No. 6

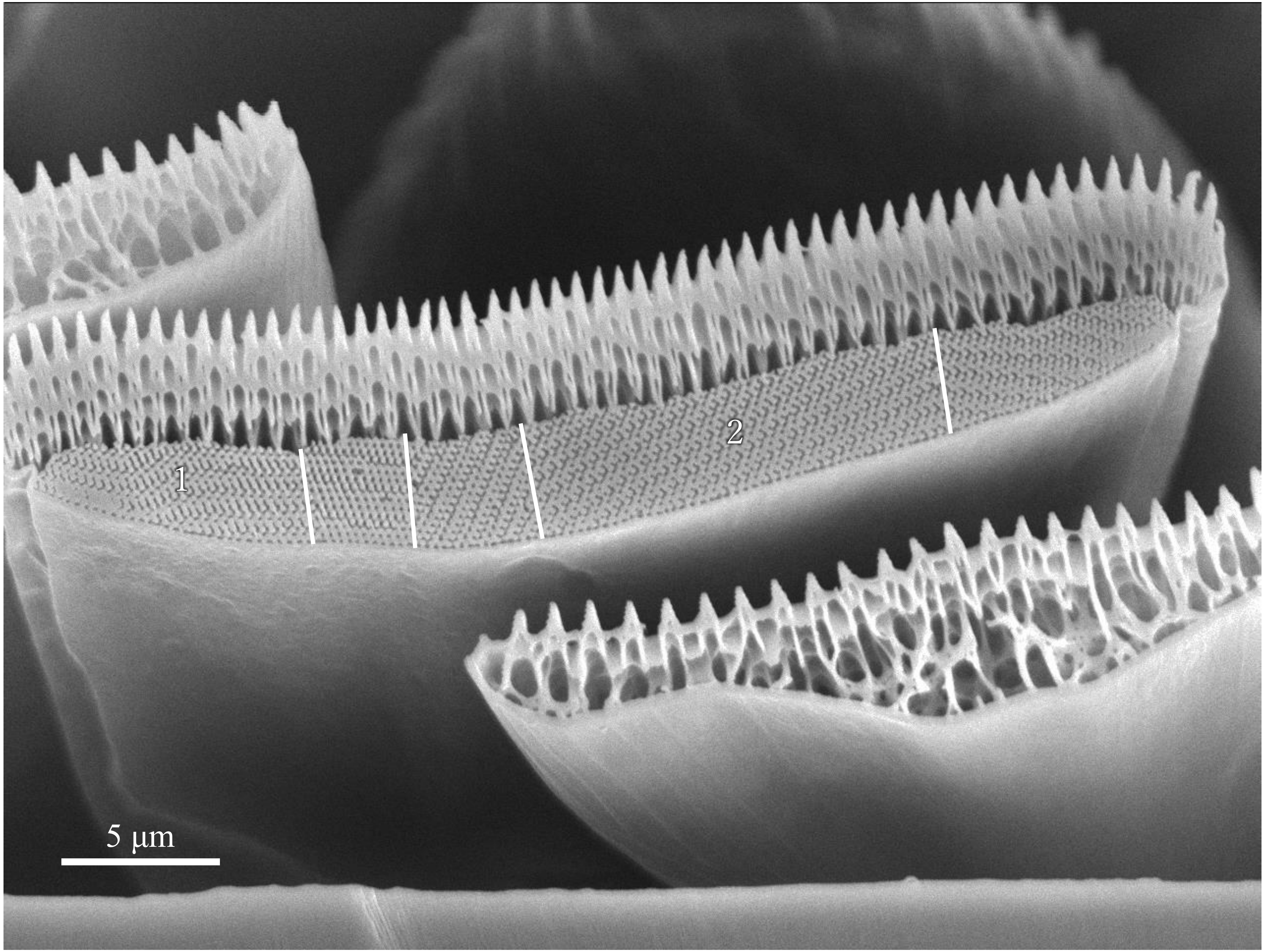

specimen No. 2
scale No. 6
domain No. 1
[100] rh spiral
LH gyroid

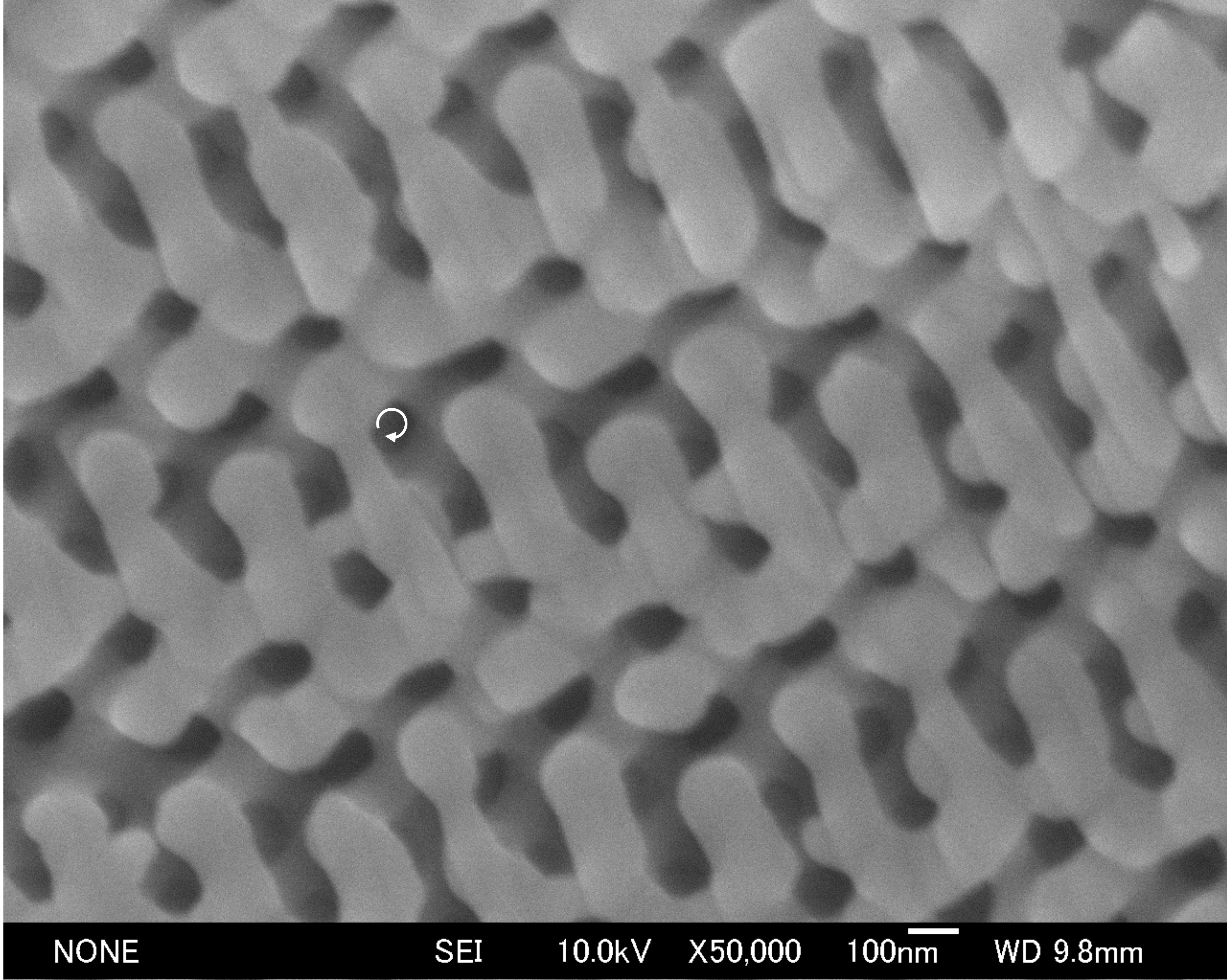

specimen No. 2
scale No. 6
domain No. 2
[100] rh spiral
LH gyroid

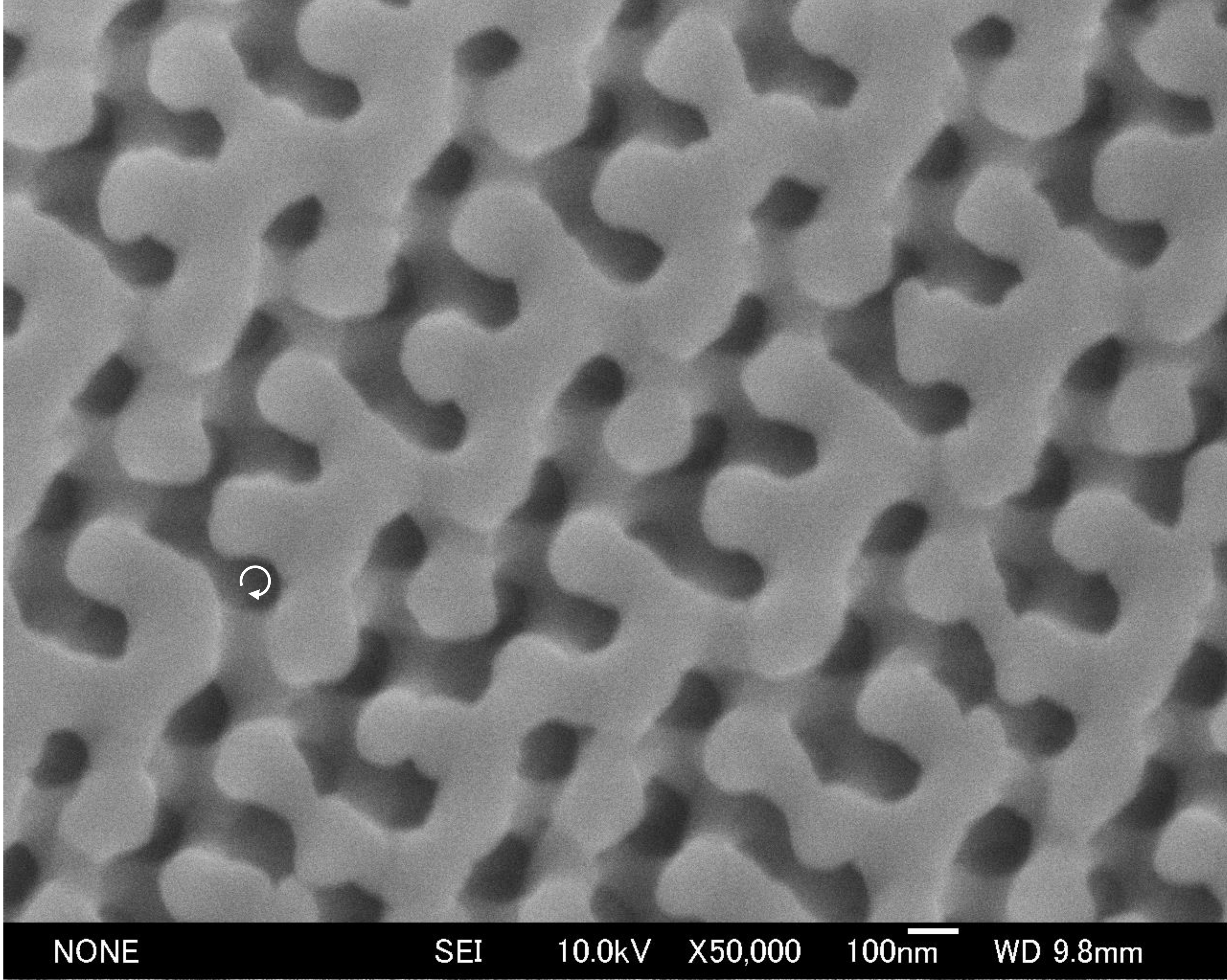

specimen No. 2
scale No. 7

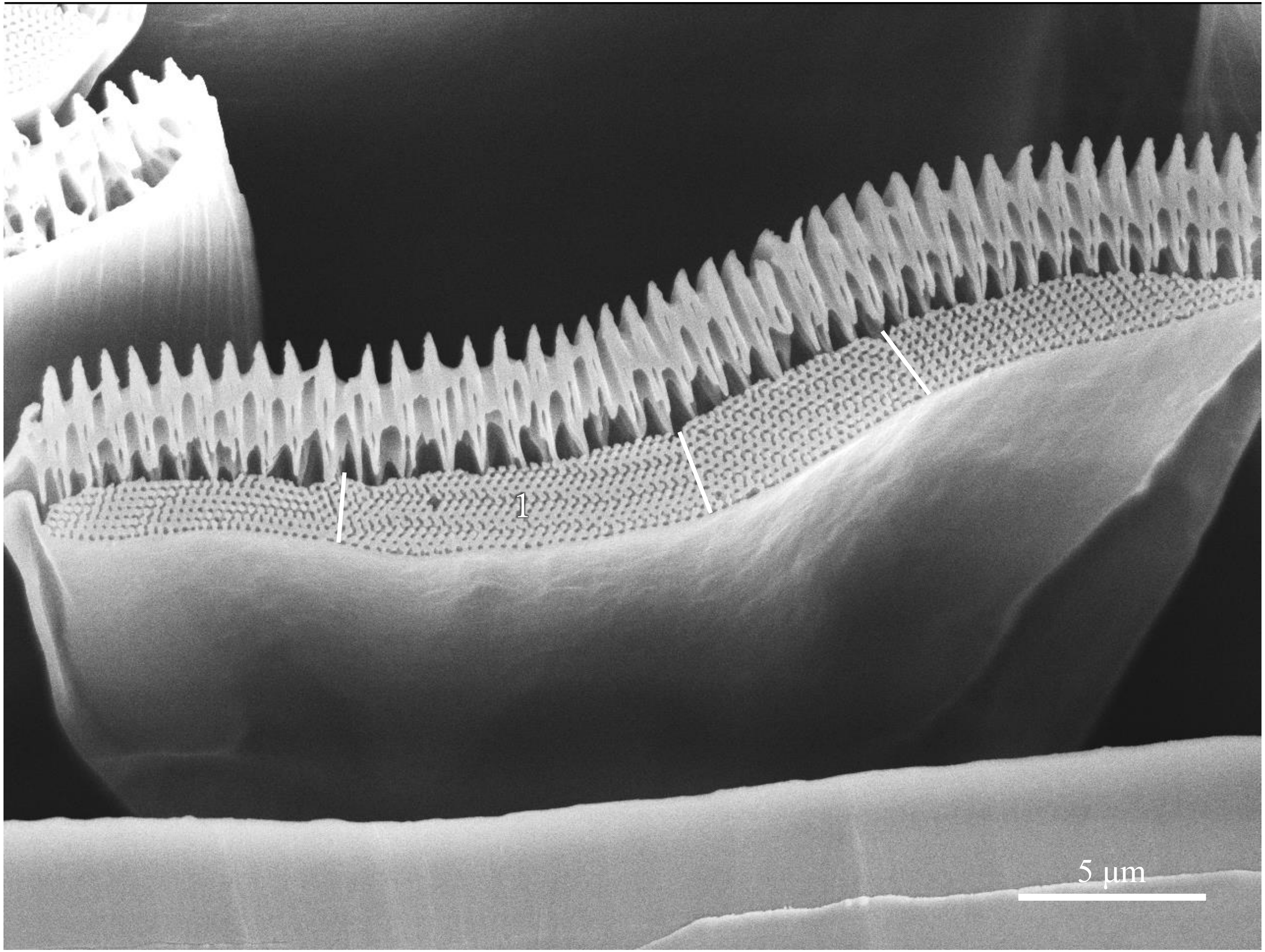

specimen No. 2
scale No. 7
domain No. 1
[100] rh spiral
LH gyroid

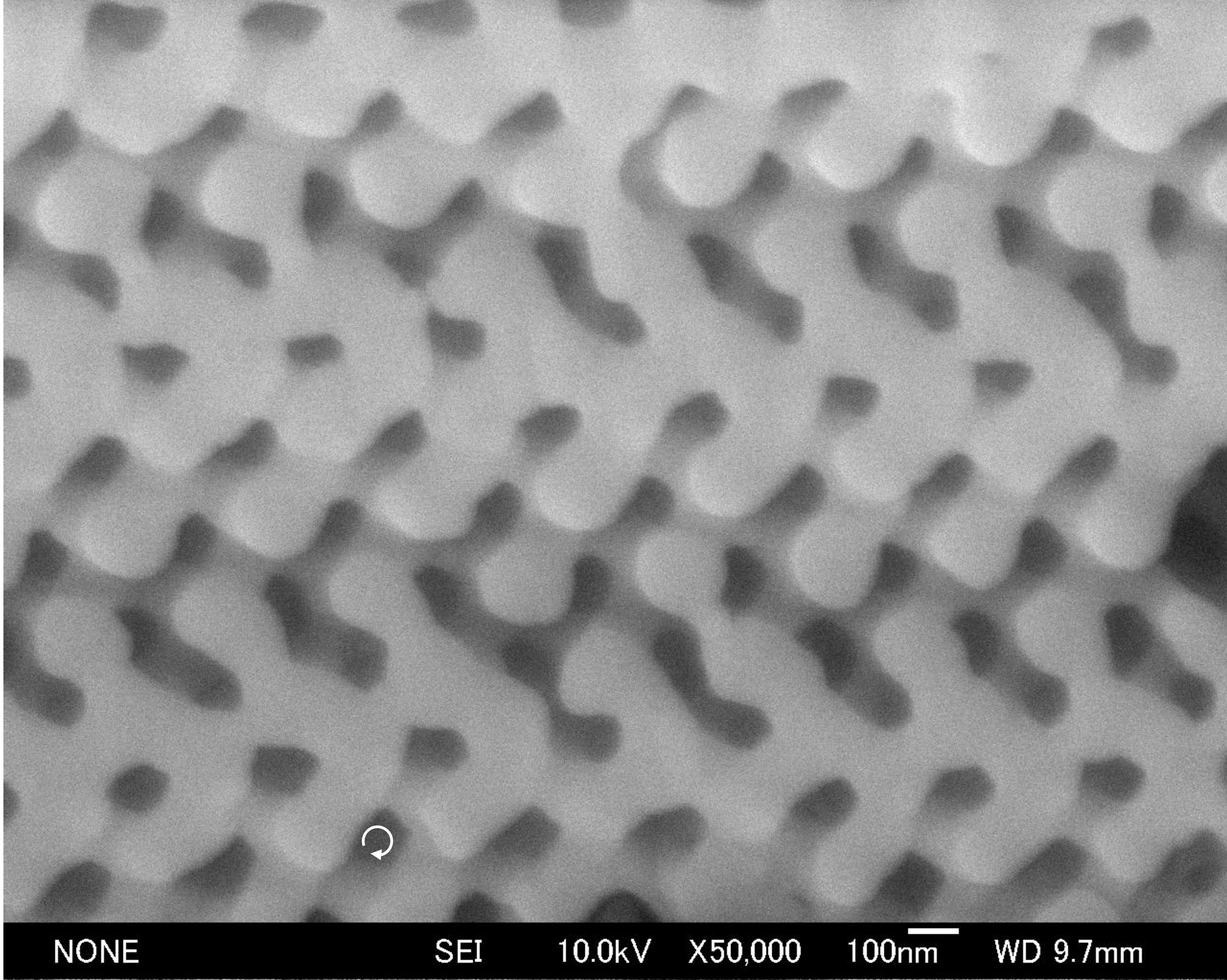

specimen No. 2
scale No. 8

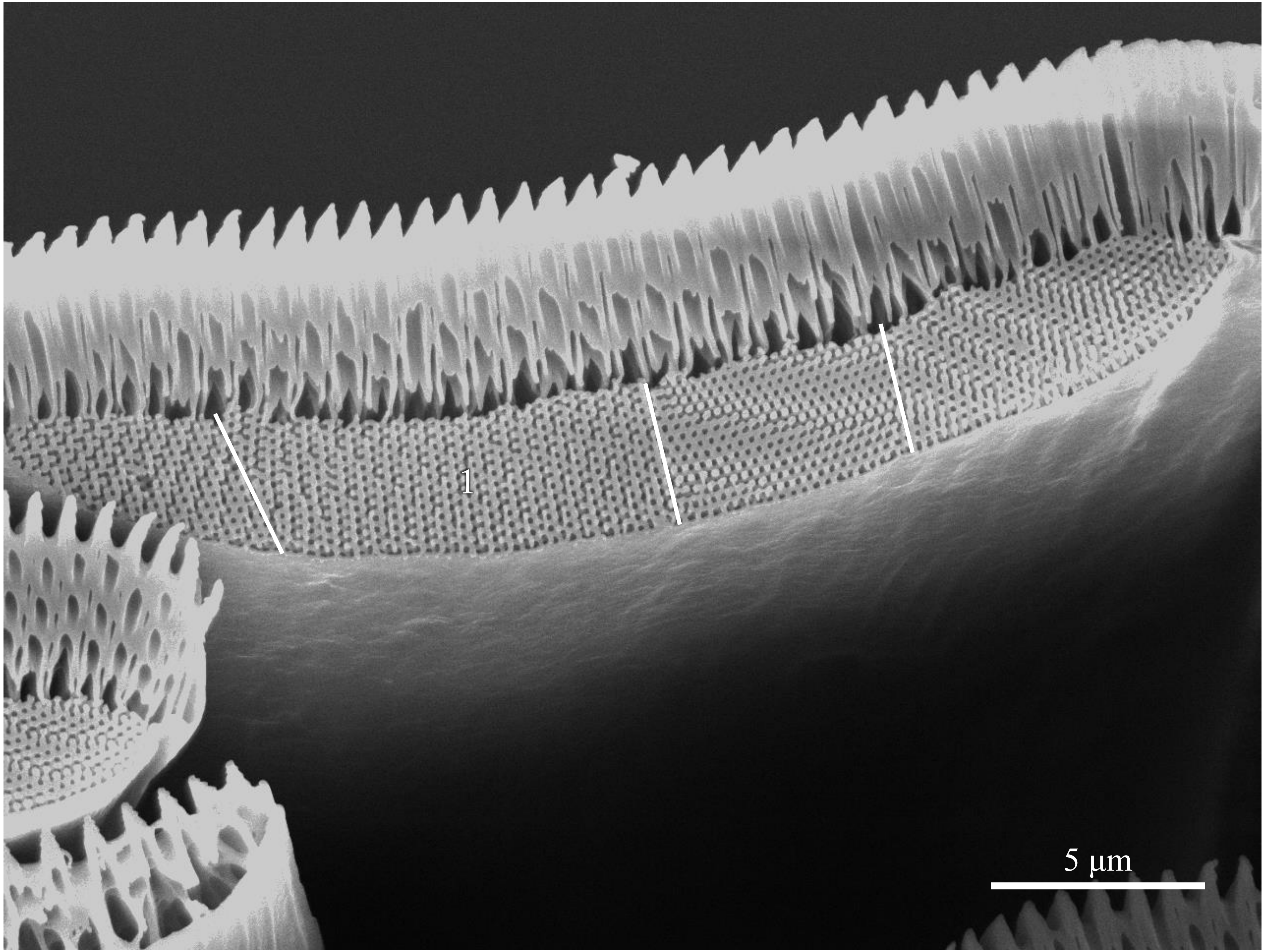

specimen No. 2
scale No. 8
domain No. 1
[111] lh spiral
LH gyroid

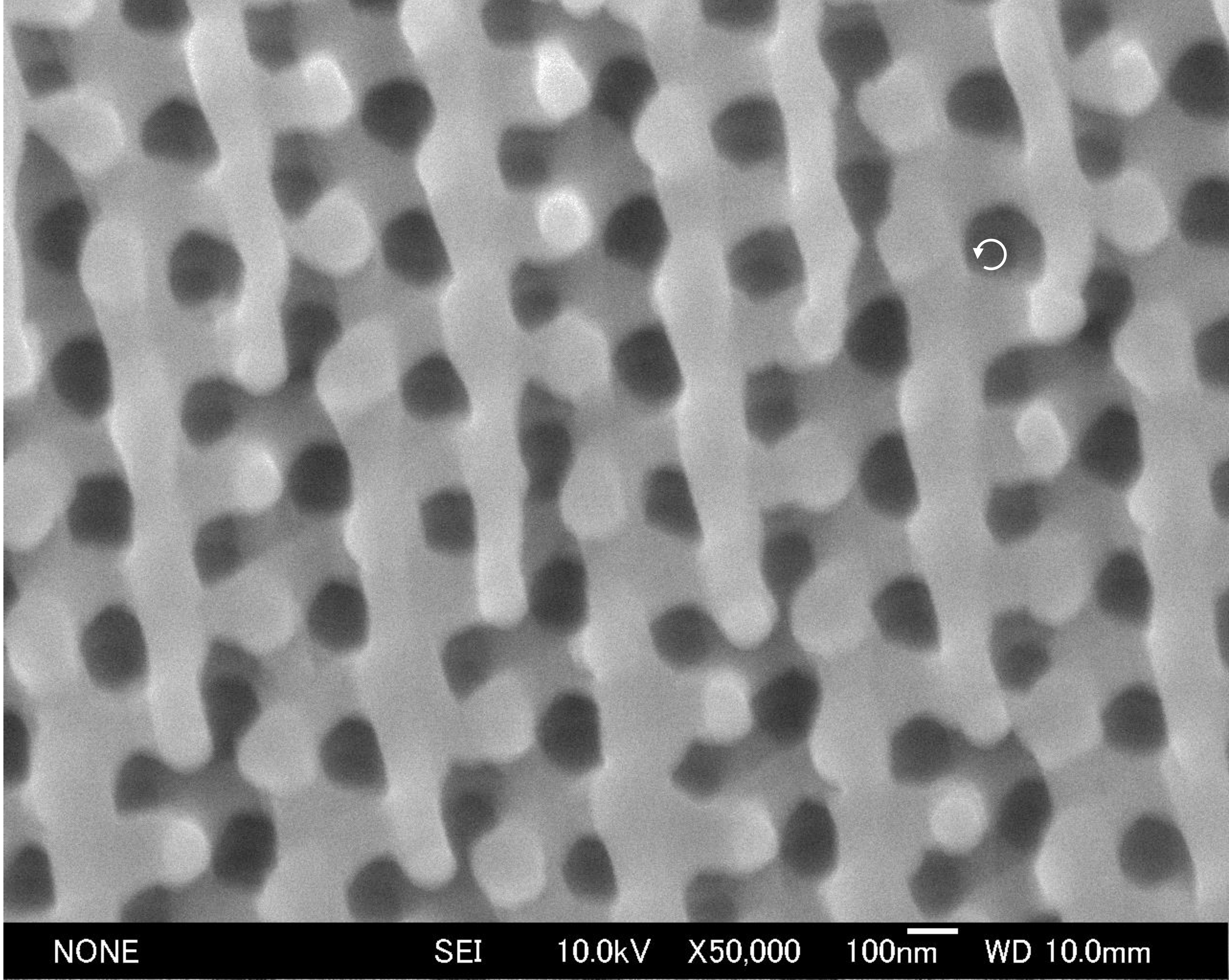

specimen No. 2
scale No. 9

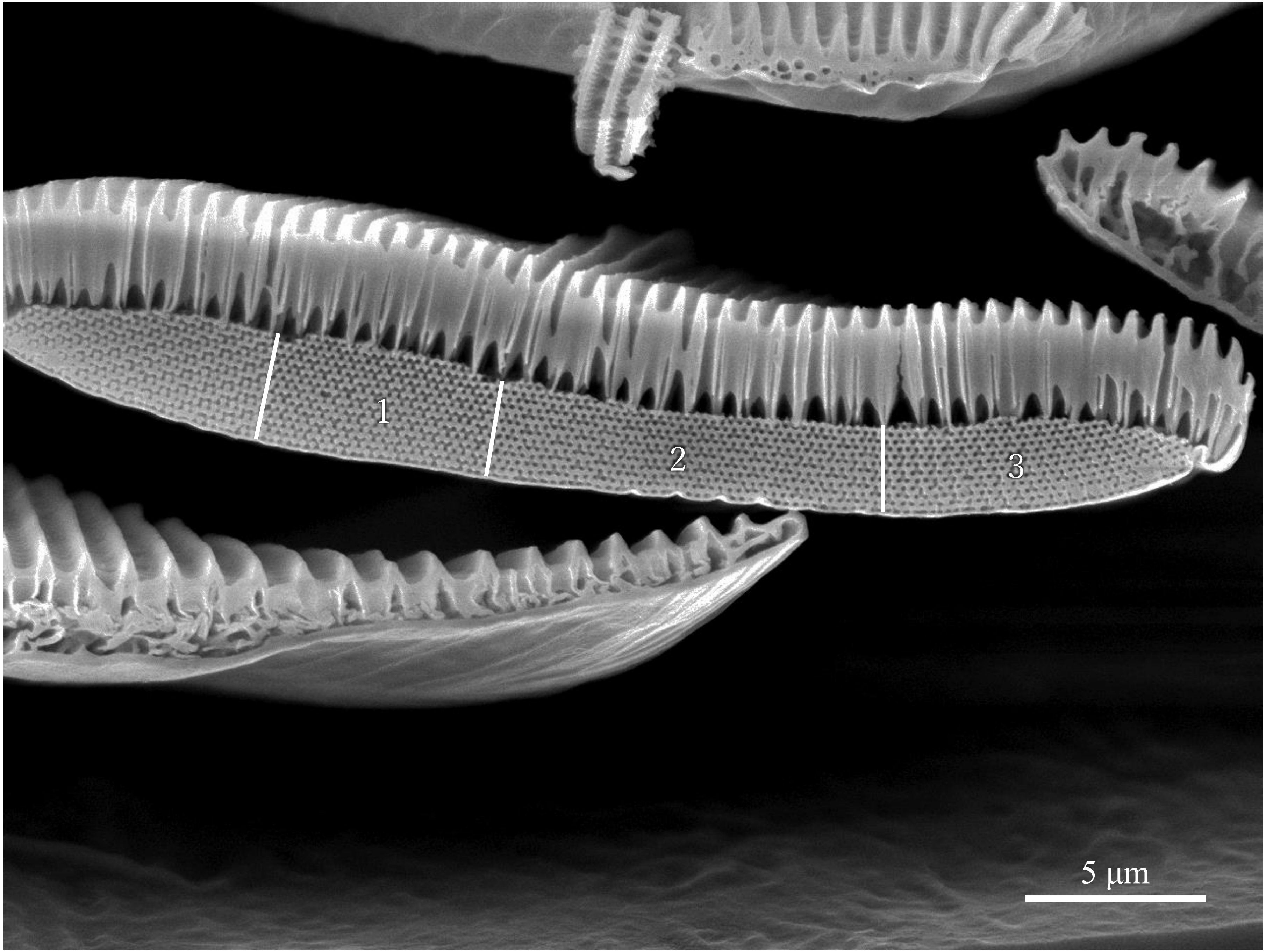

specimen No. 2
scale No. 9
domain No. 1
[111] lh spiral
LH gyroid

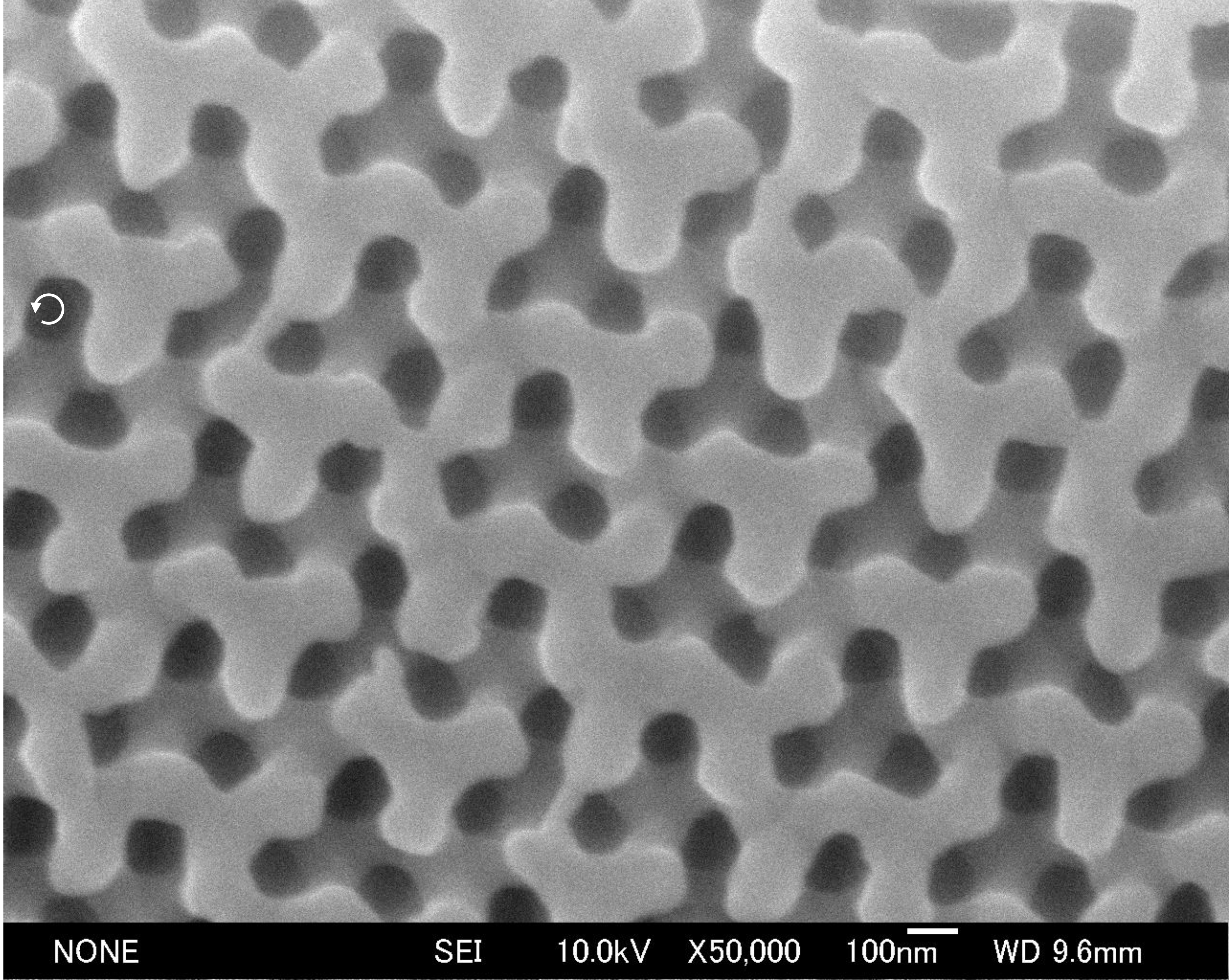

specimen No. 2
scale No. 9
domain No. 2
[111] lh spiral
LH gyroid

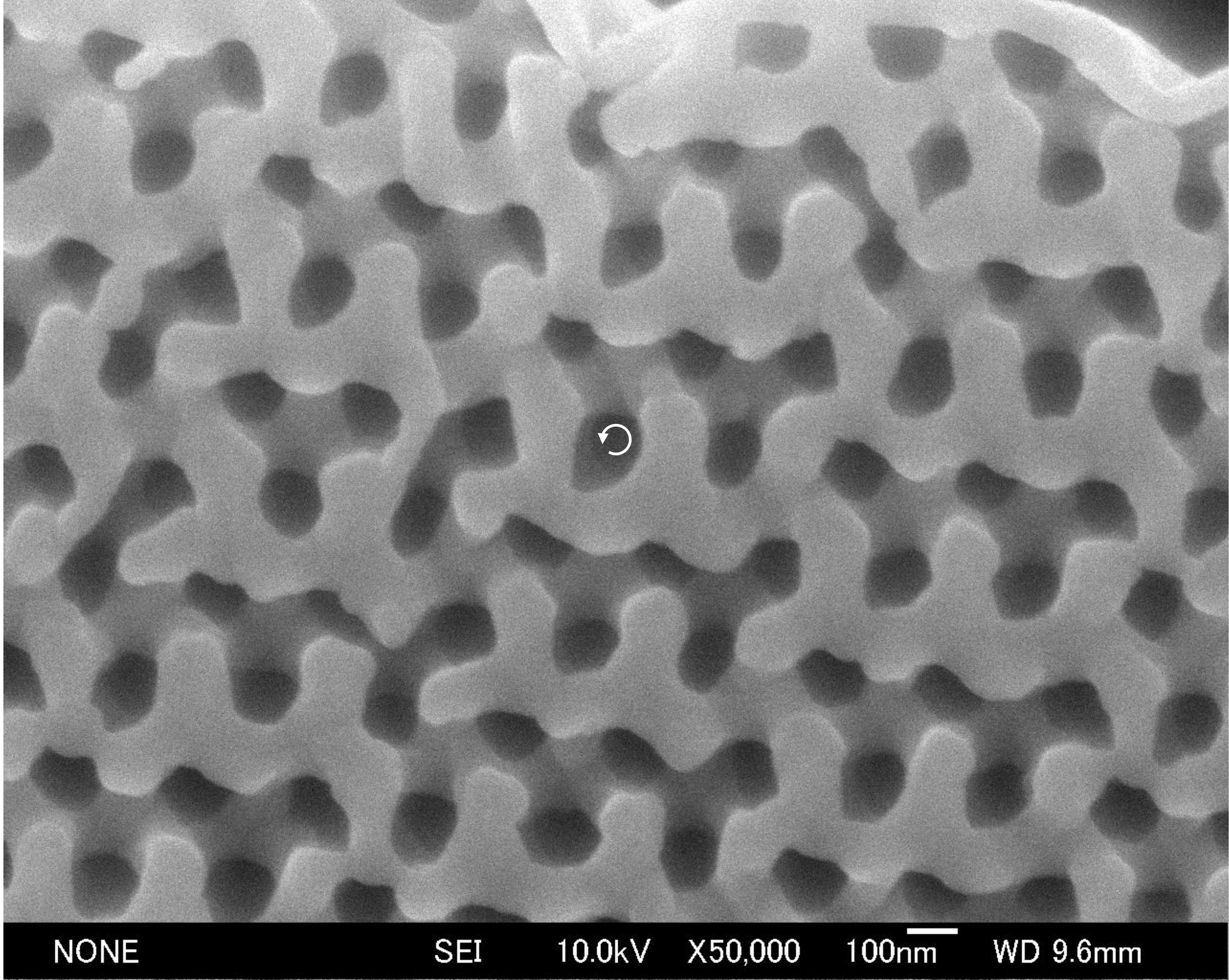

specimen No. 2
scale No. 9
domain No. 3
[111] lh spiral
LH gyroid

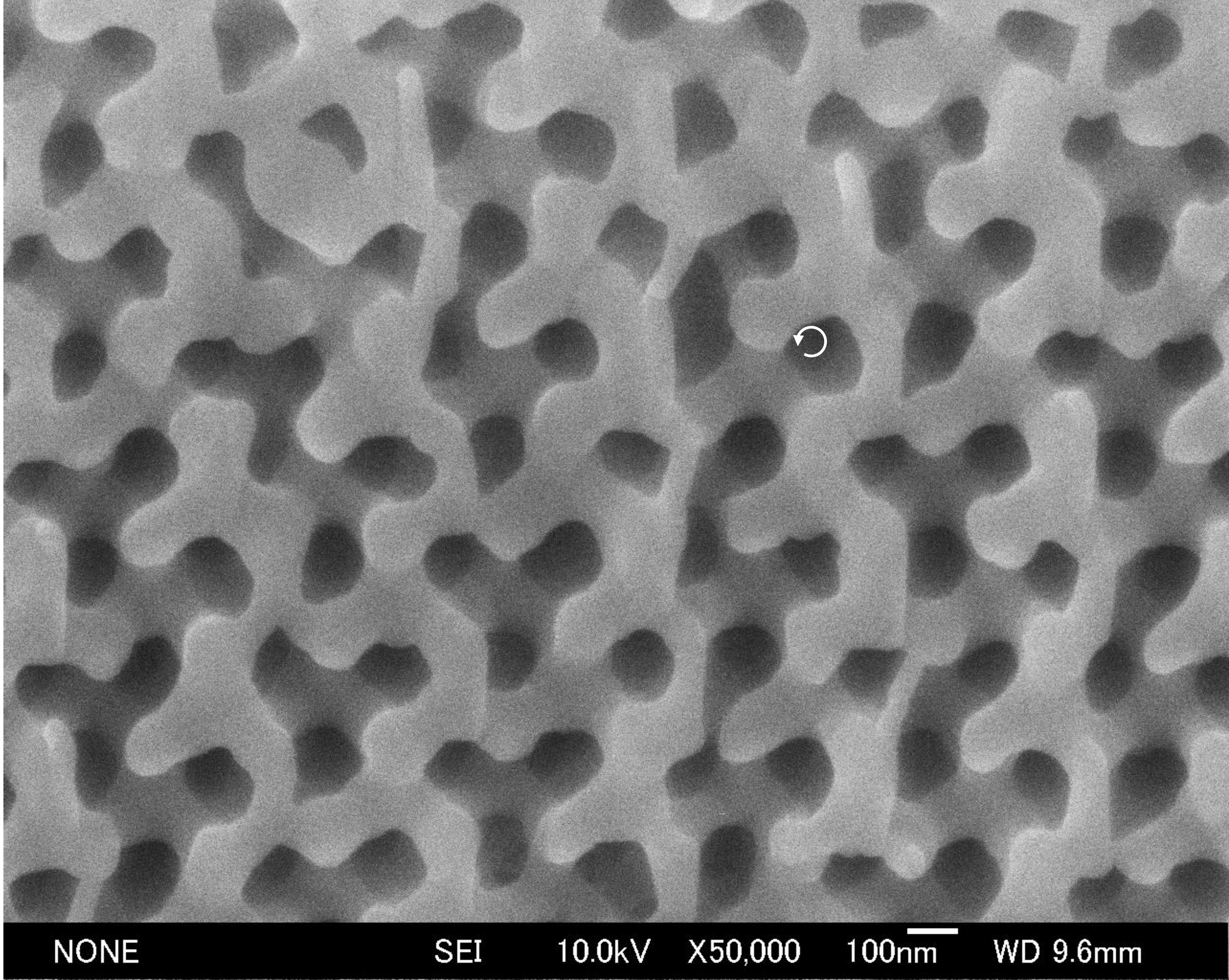

specimen No. 2
scale No. 10

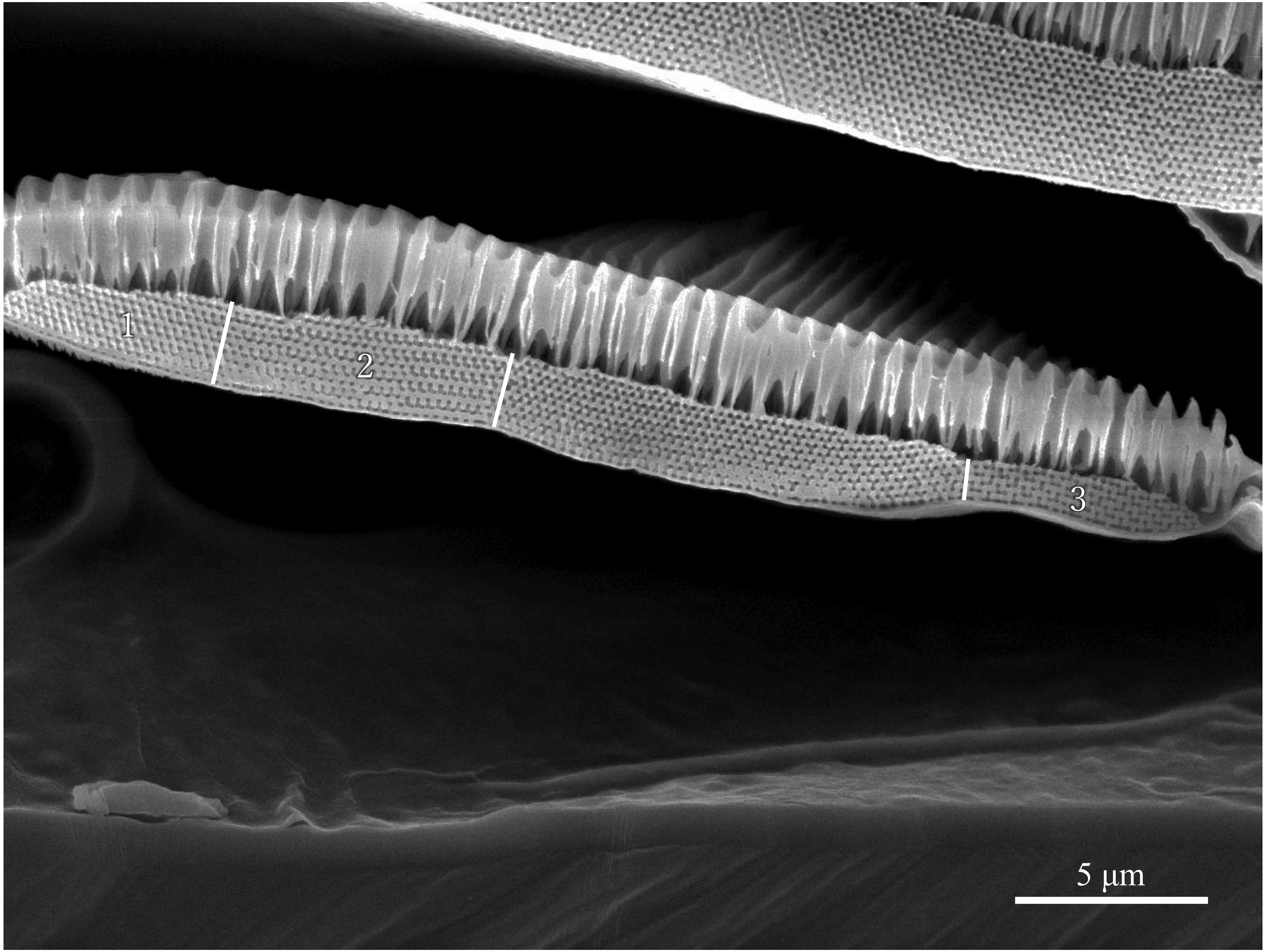

specimen No. 2
scale No. 10
domain No. 1
[111] lh spiral
LH gyroid

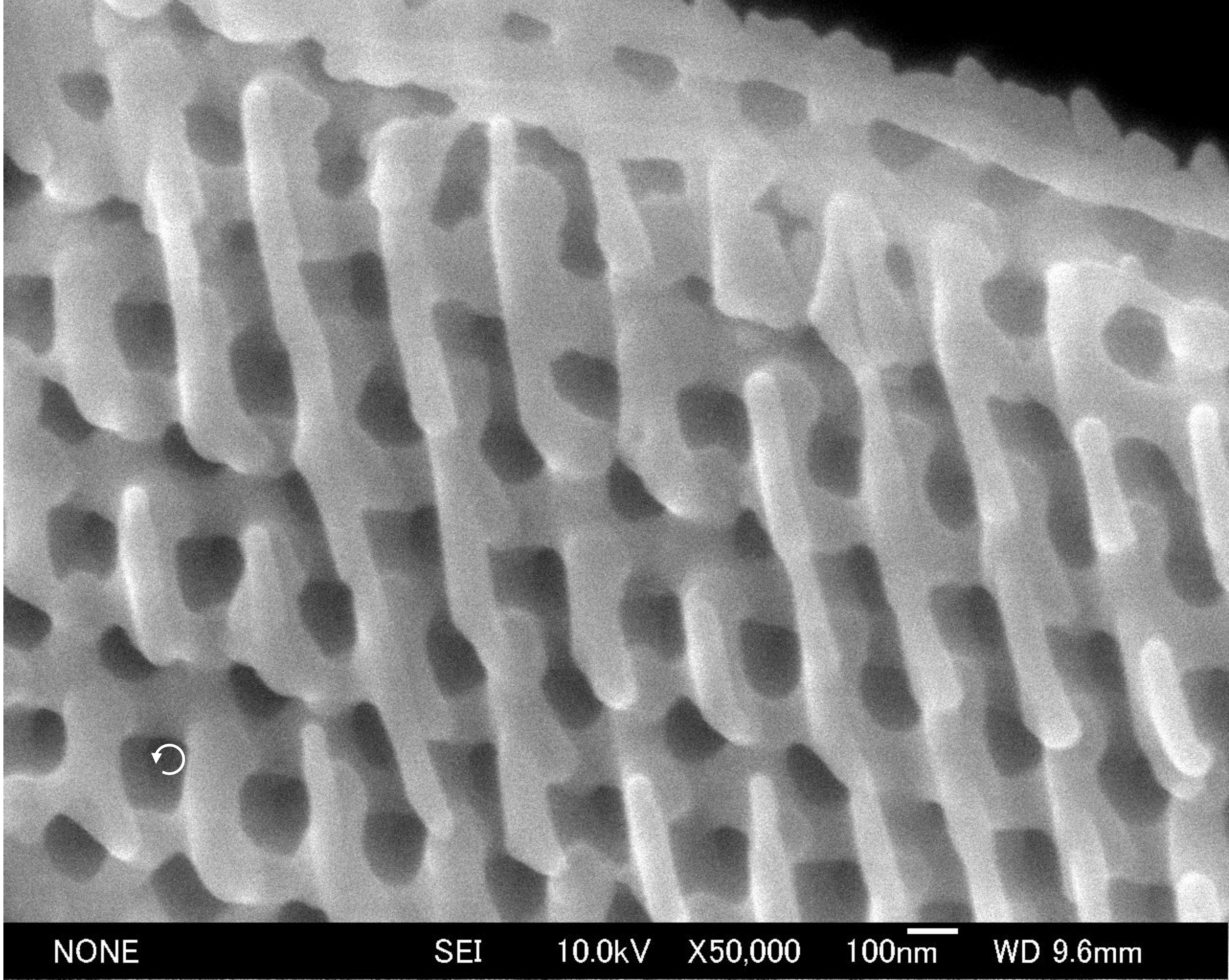

specimen No. 2
scale No. 10
domain No. 2
[111] rh spiral
RH gyroid

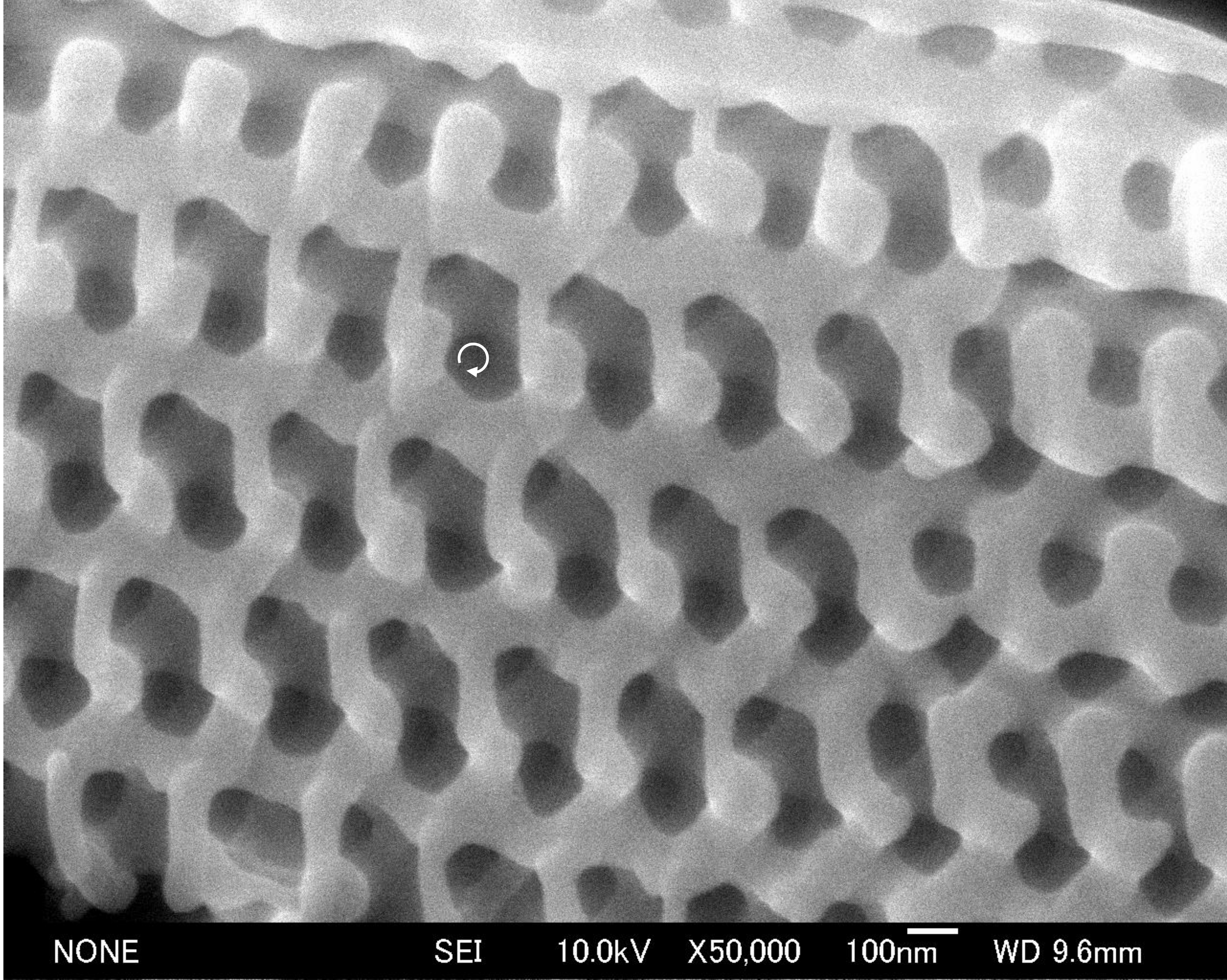

specimen No. 2
scale No. 10
domain No. 3
[100] rh spiral
LH gyroid

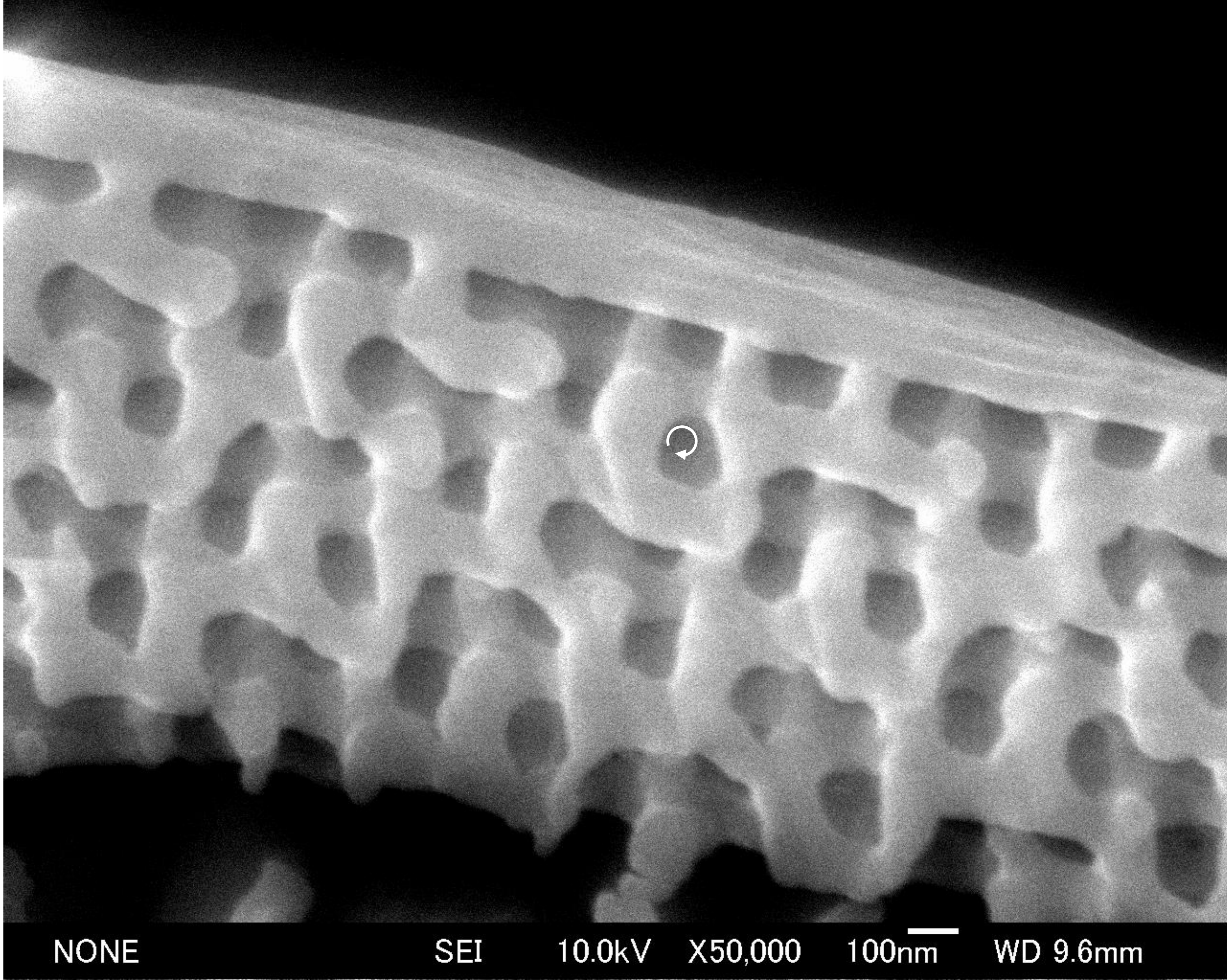

specimen No. 2
scale No. 11

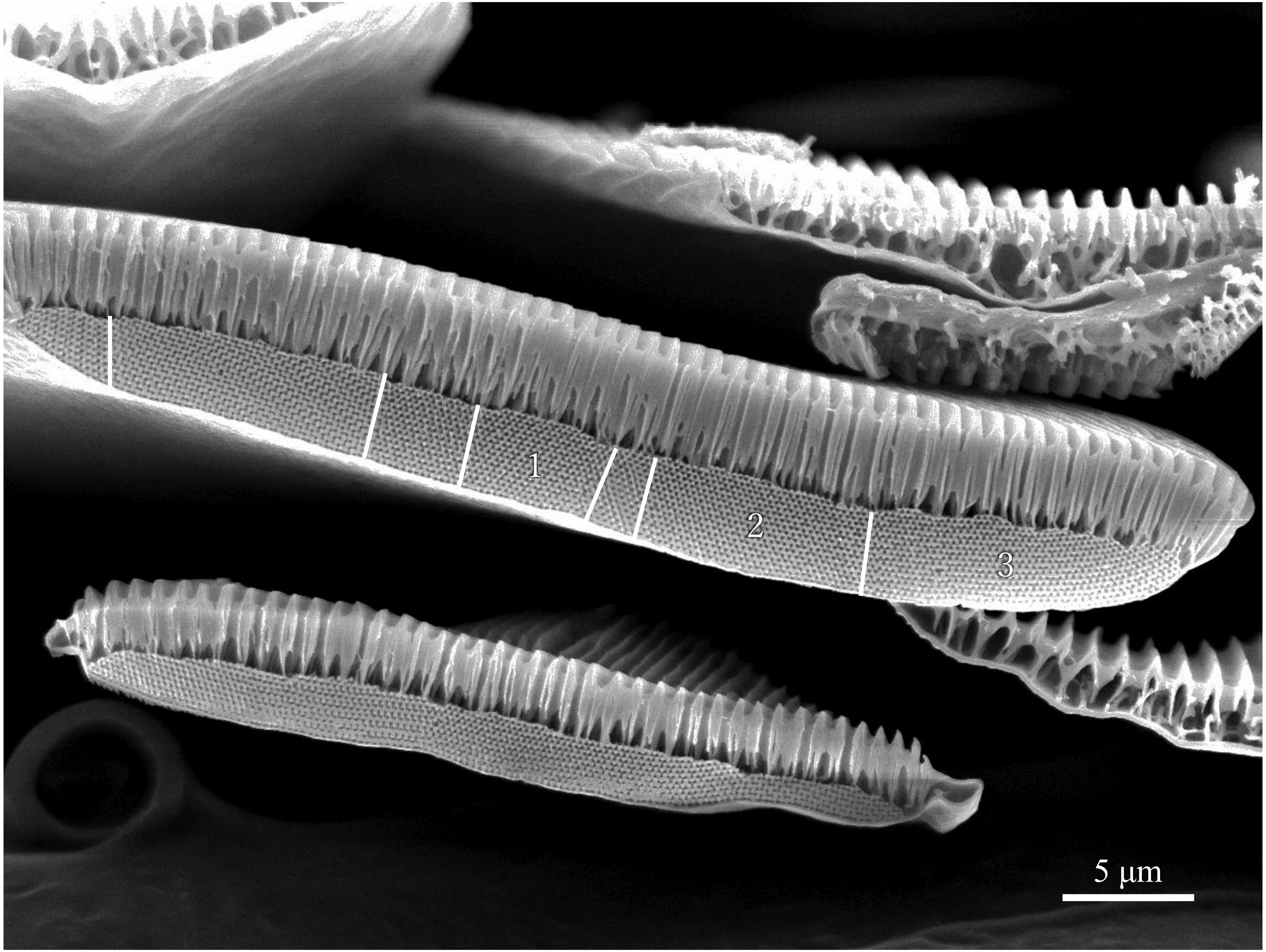

specimen No. 2
scale No. 11
domain No. 1
[111] lh spiral
LH gyroid

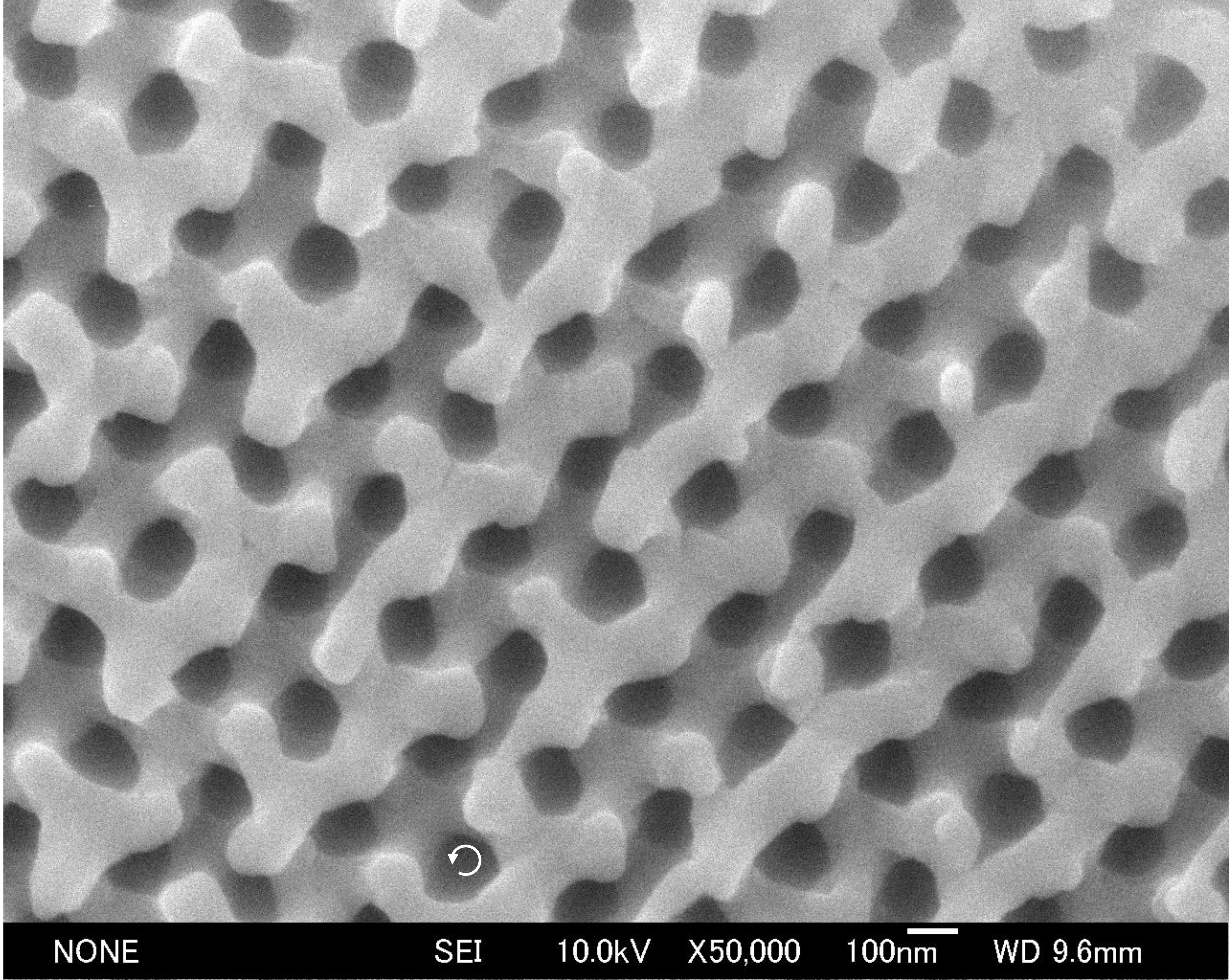

specimen No. 2
scale No. 11
domain No. 2
[111] lh spiral
LH gyroid

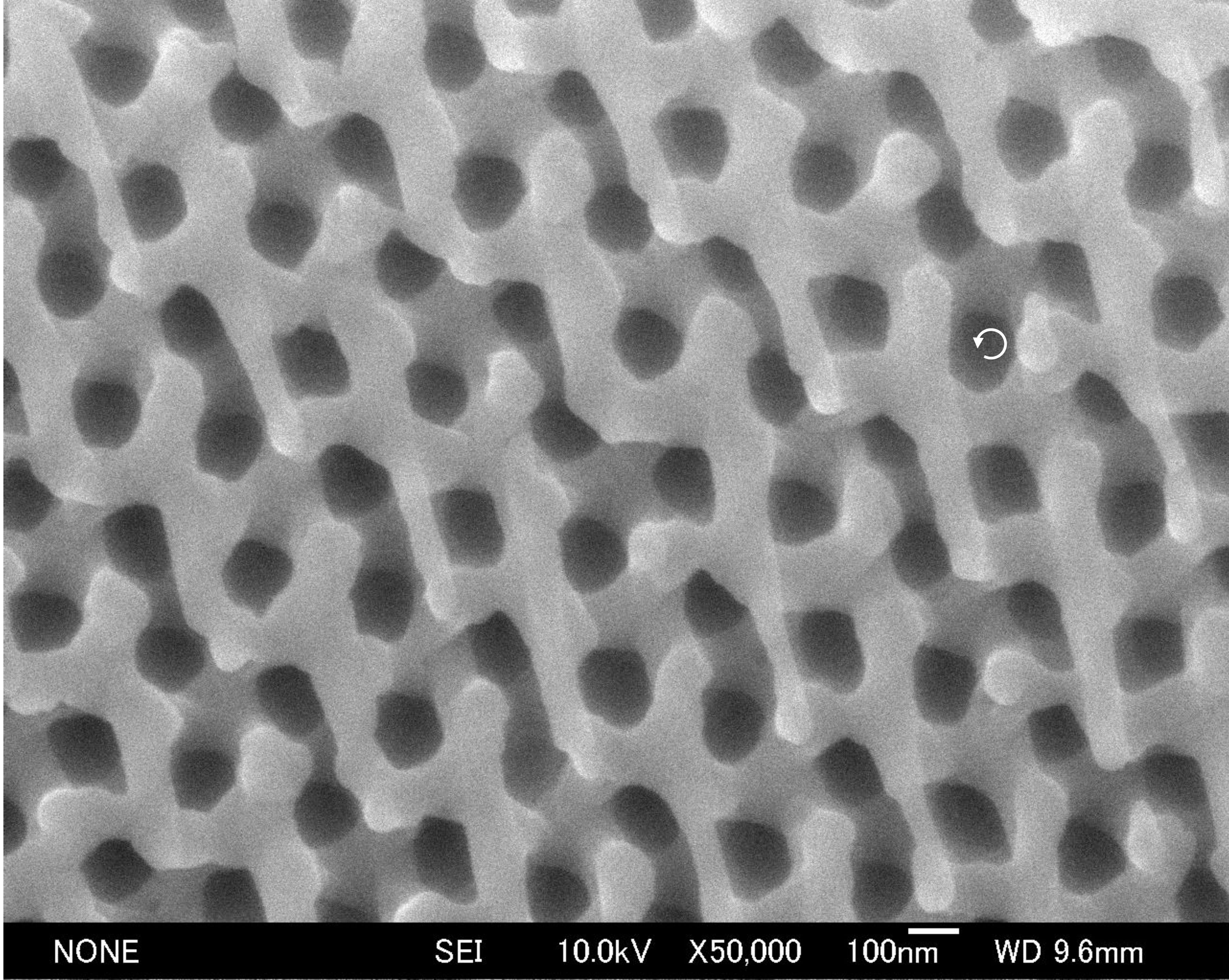

specimen No. 2
scale No. 11
domain No. 3
[111] rh spiral
RH gyroid

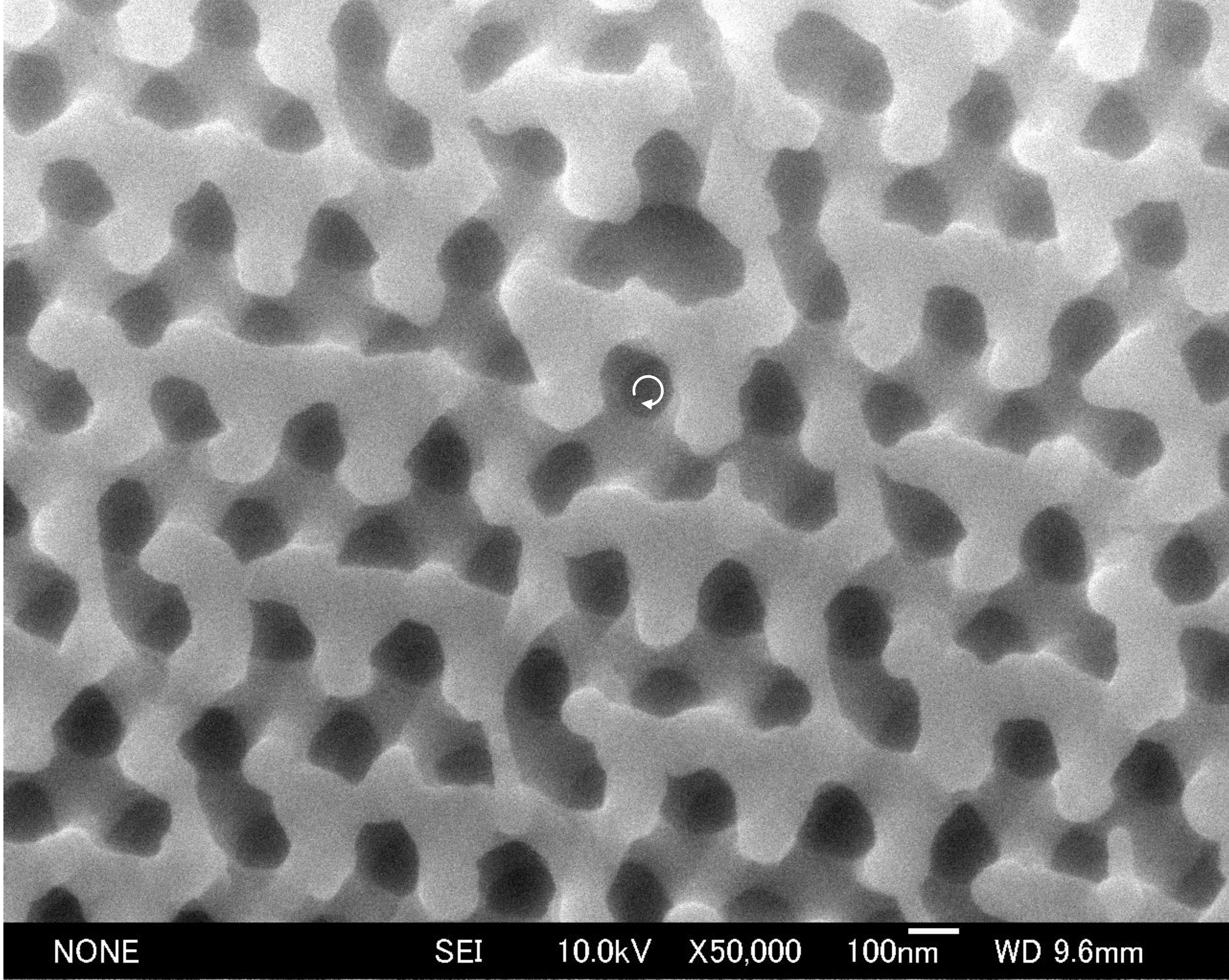

specimen No. 2
scale No. 12

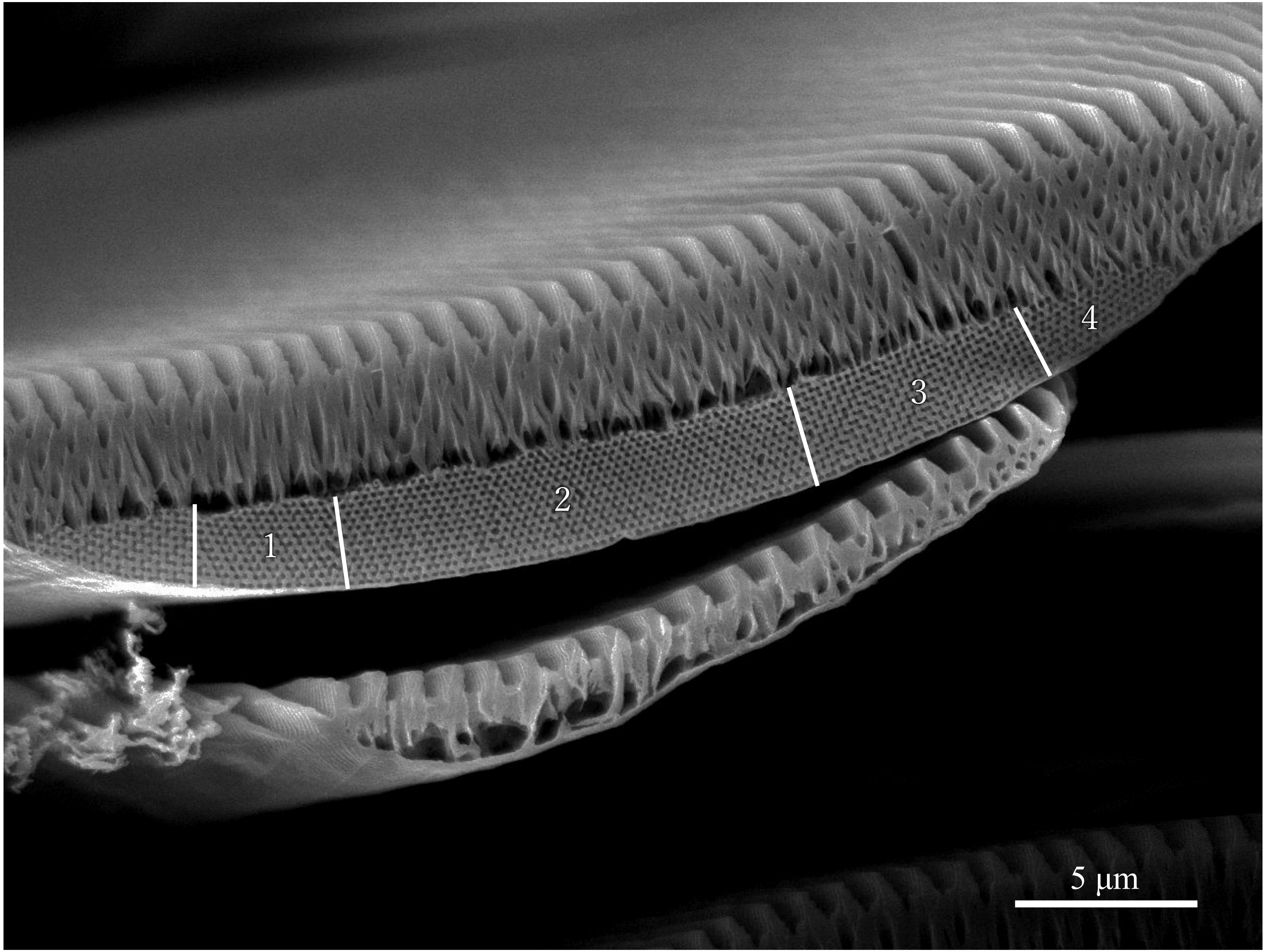

specimen No. 2
scale No. 12
domain No. 1
[111] rh spiral
RH gyroid

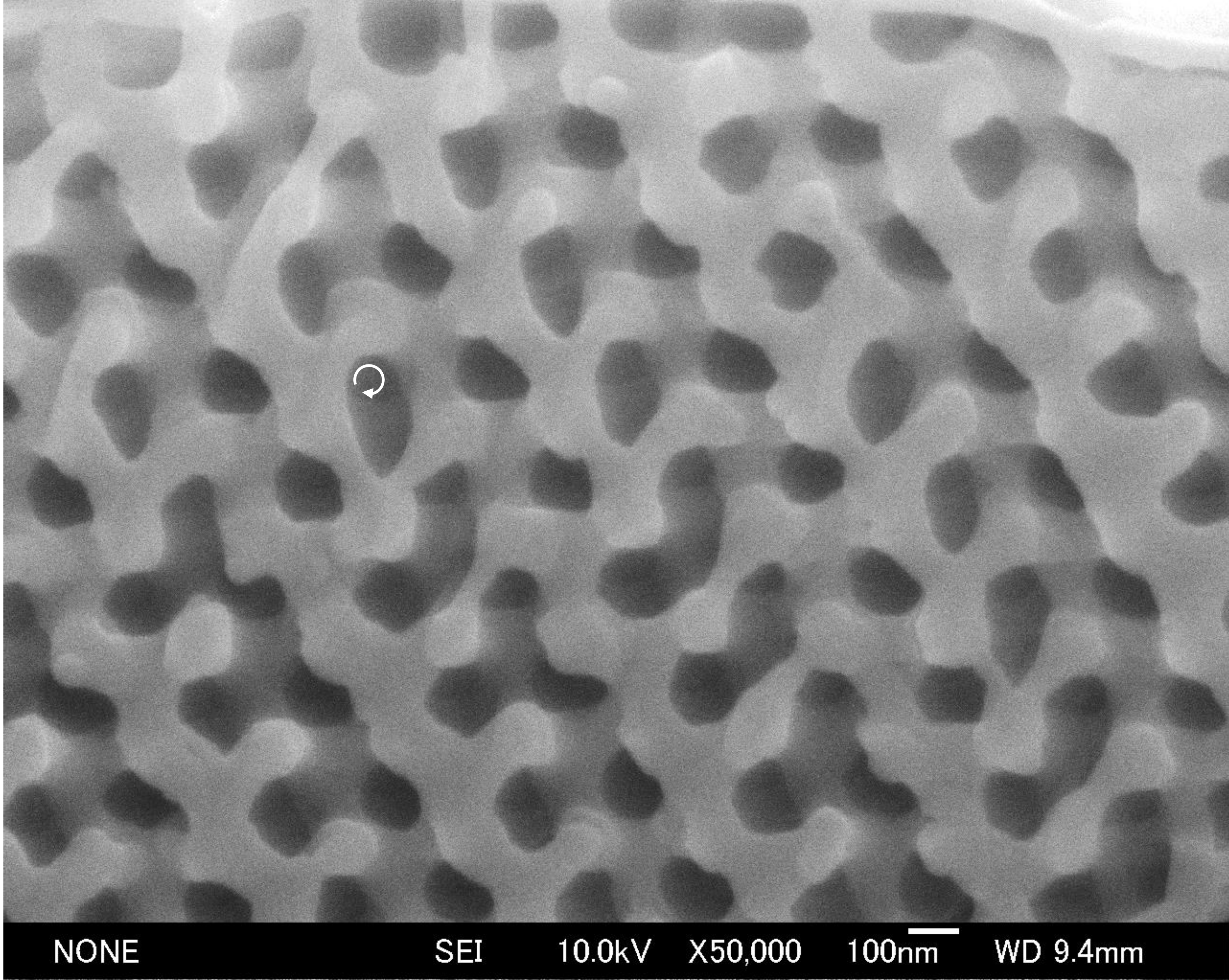

specimen No. 2
scale No. 12
domain No. 2
[111] lh spiral
LH gyroid

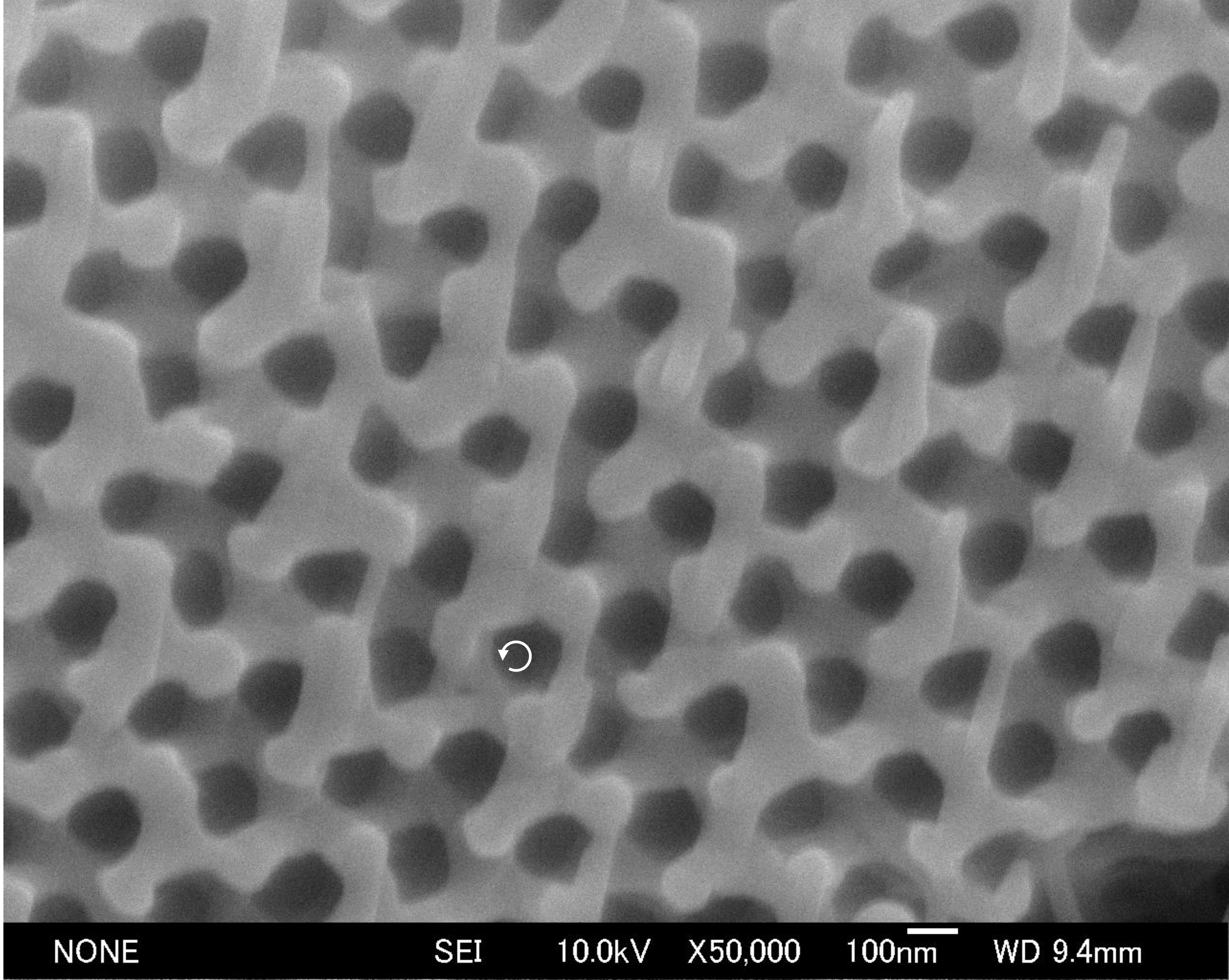

specimen No. 2
scale No. 12
domain No. 3
[100] rh spiral
LH gyroid

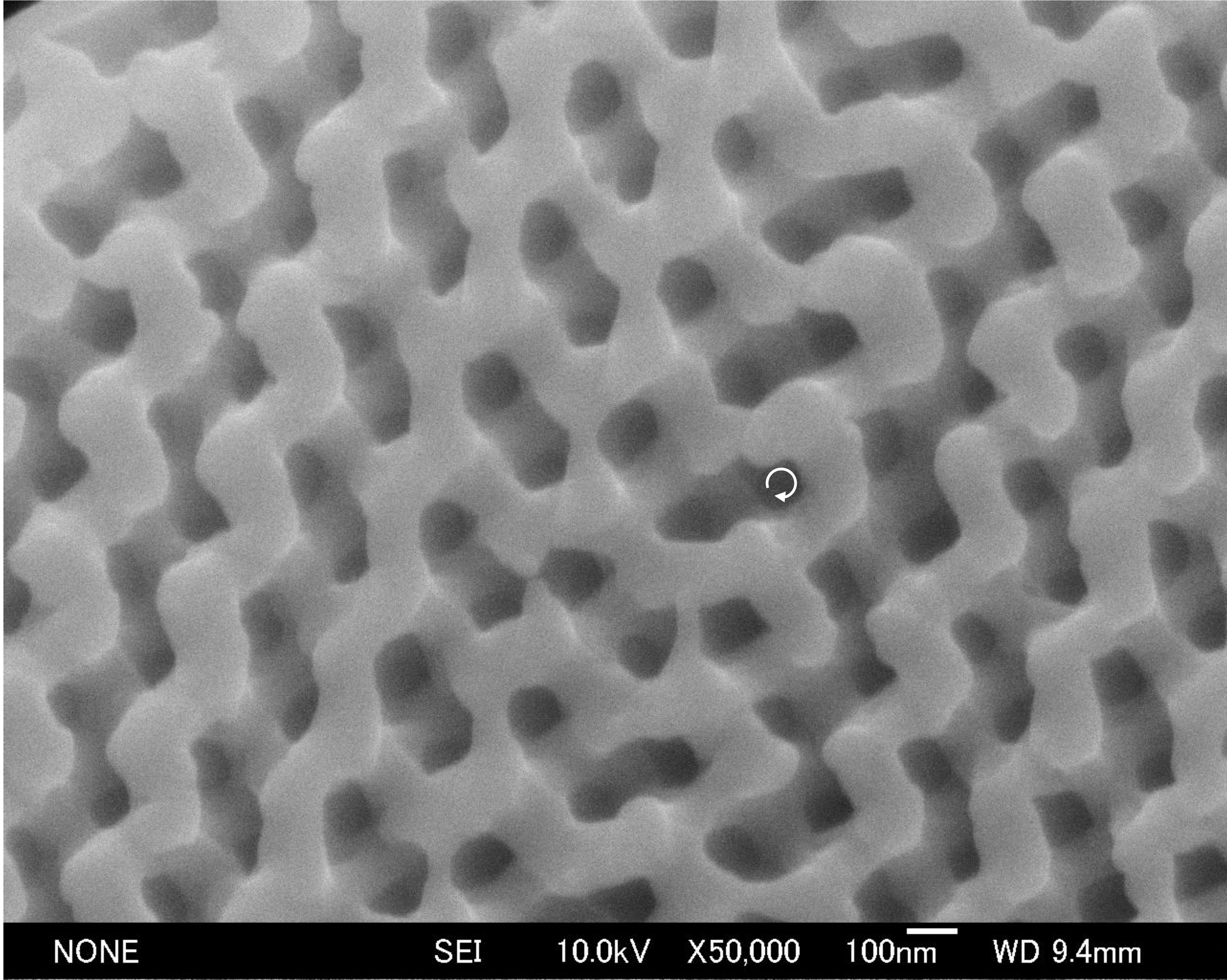

specimen No. 2
scale No. 12
domain No. 4
[111] lh spiral
LH gyroid

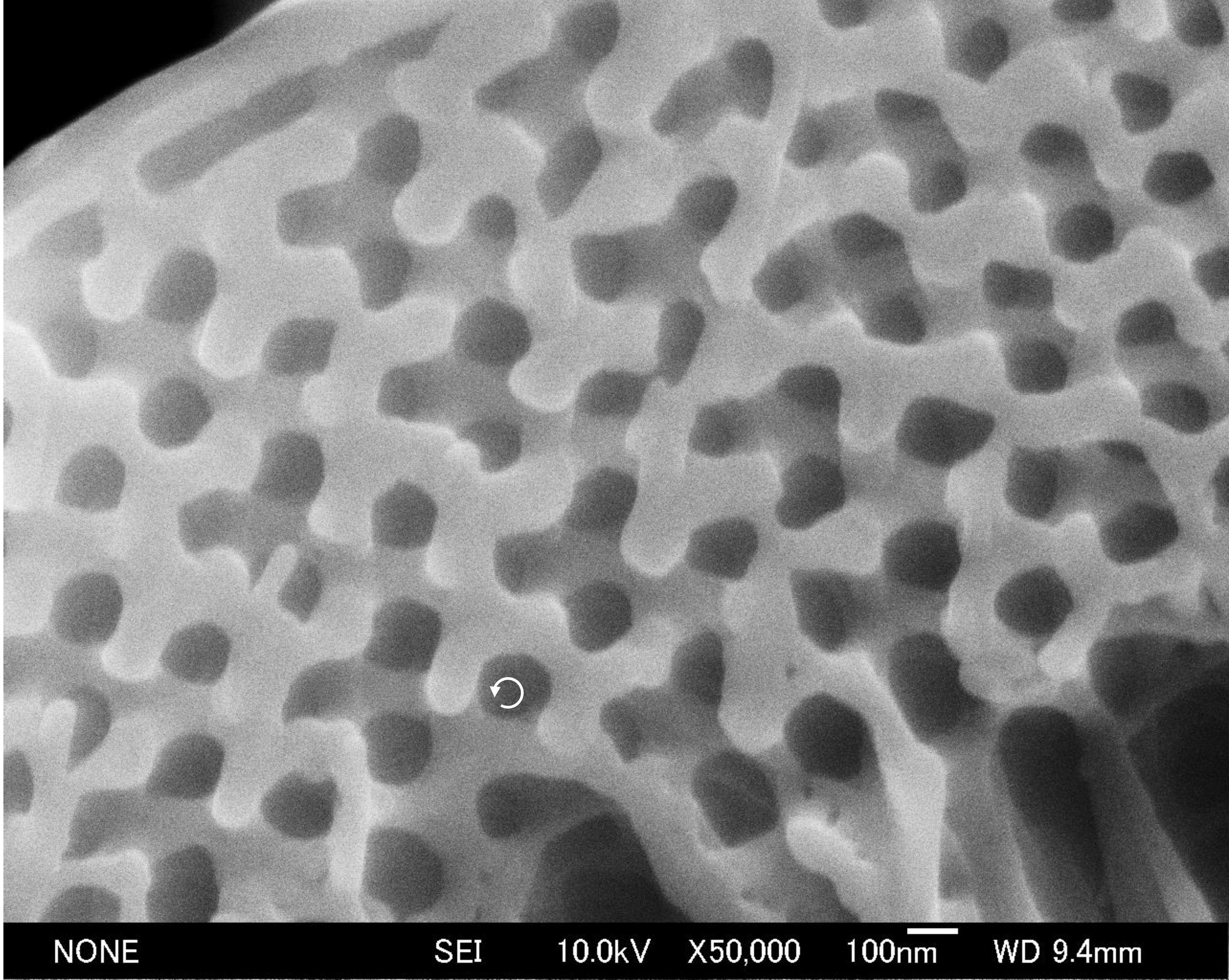

specimen No. 2
scale No. 13

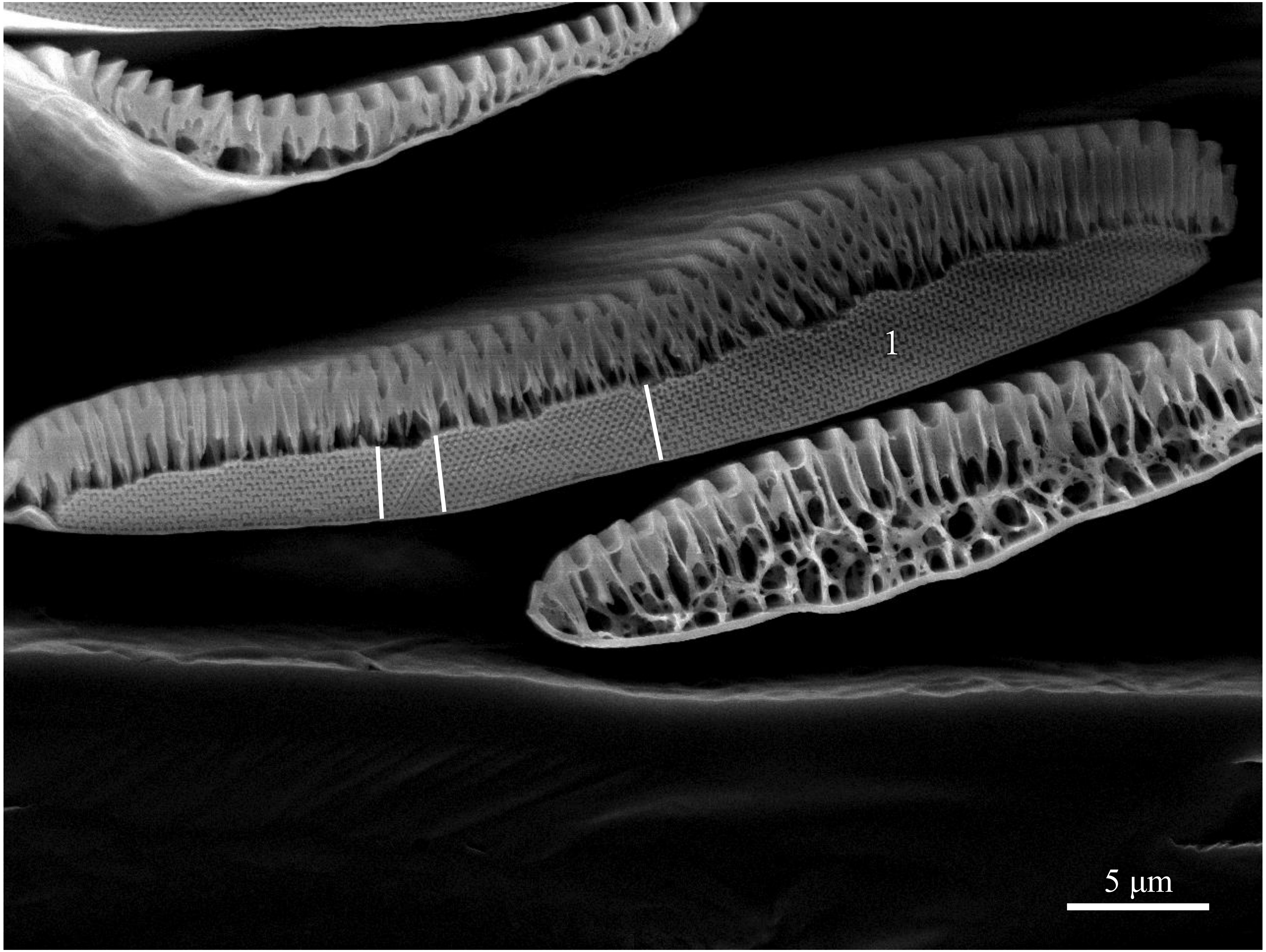

specimen No. 2
scale No. 13
domain No. 1
[100] rh spiral
LH gyroid

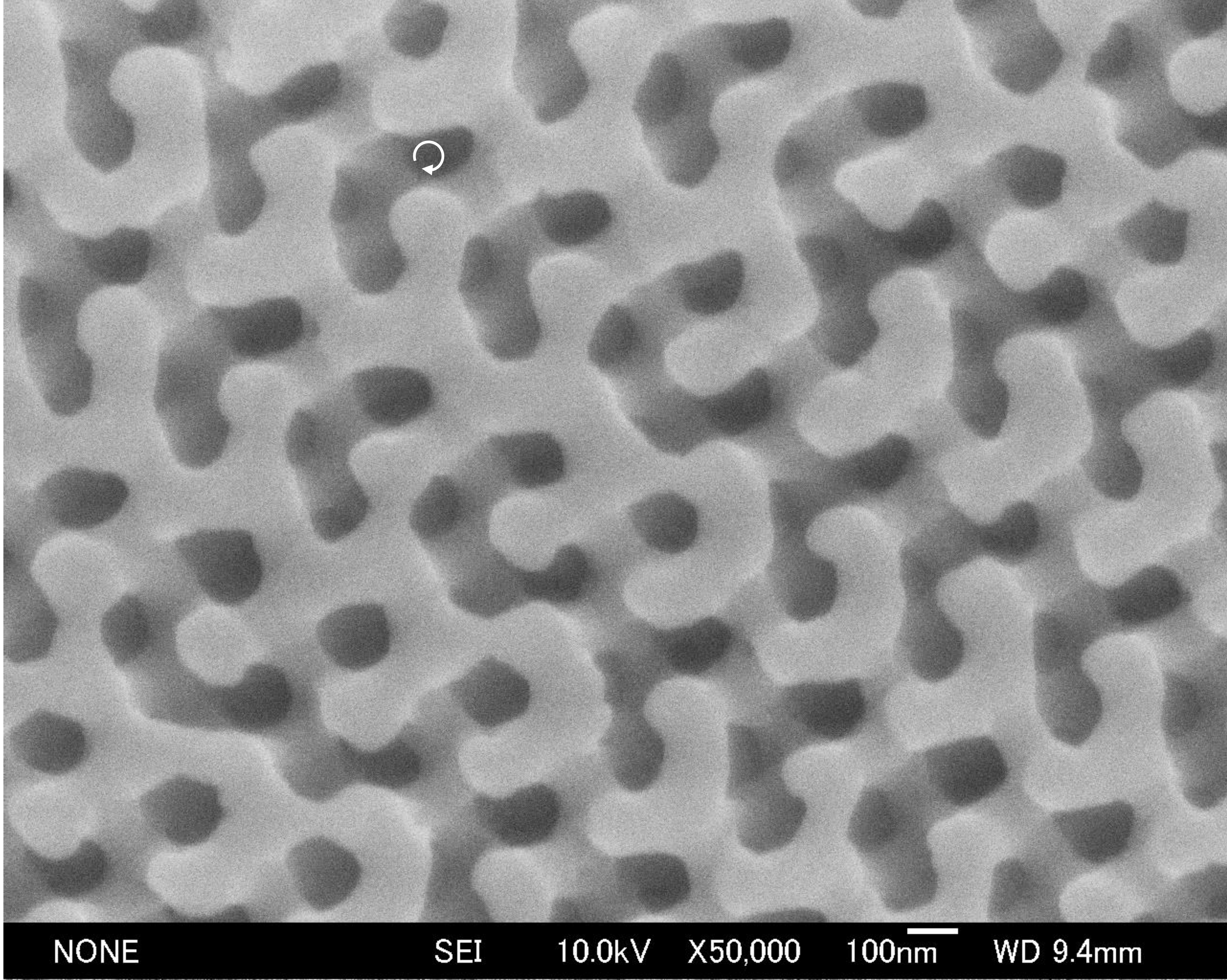

specimen No. 2
scale No. 14

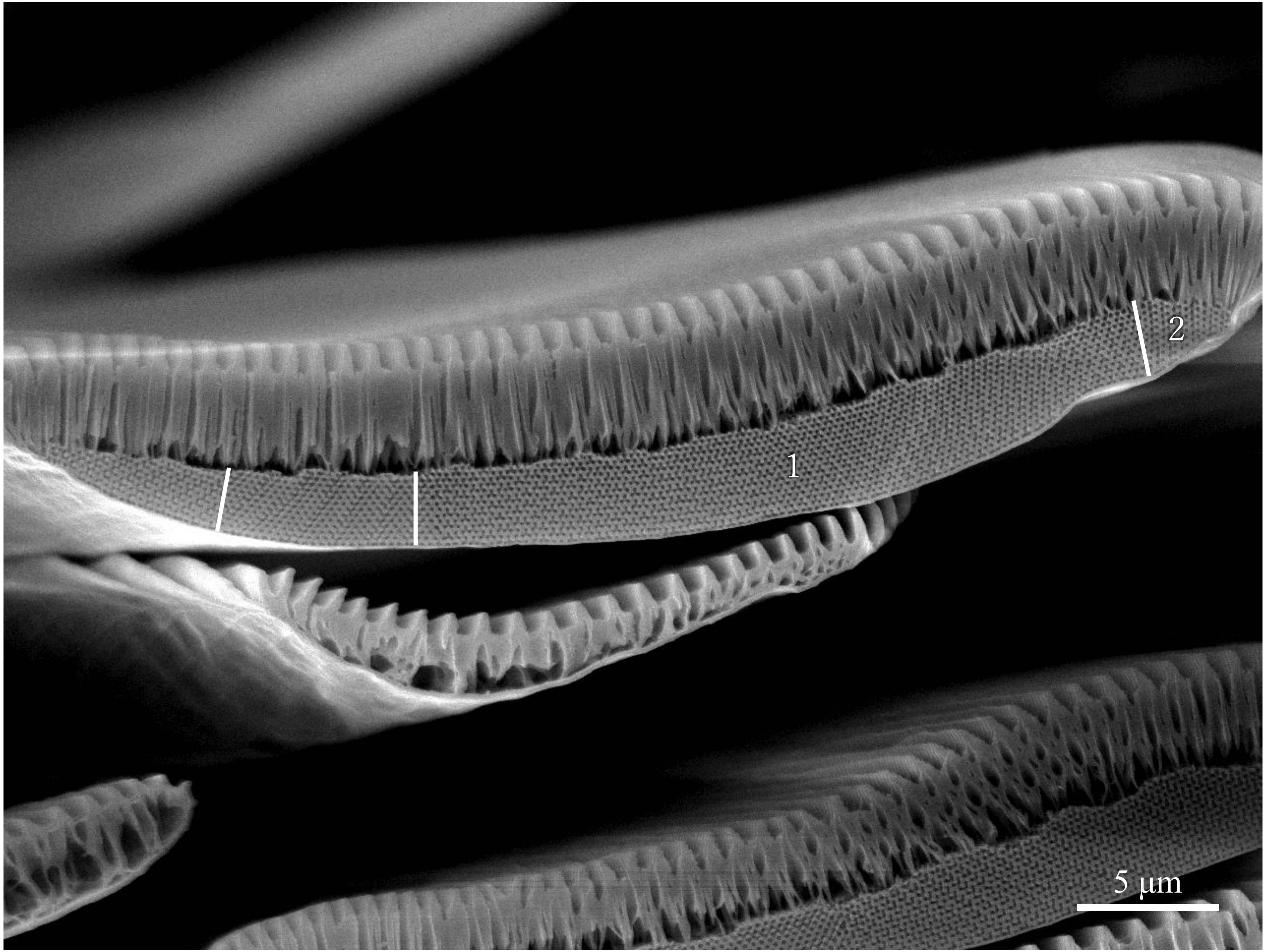

specimen No. 2
scale No. 14
domain No. 1
[111] lh spiral
LH gyroid

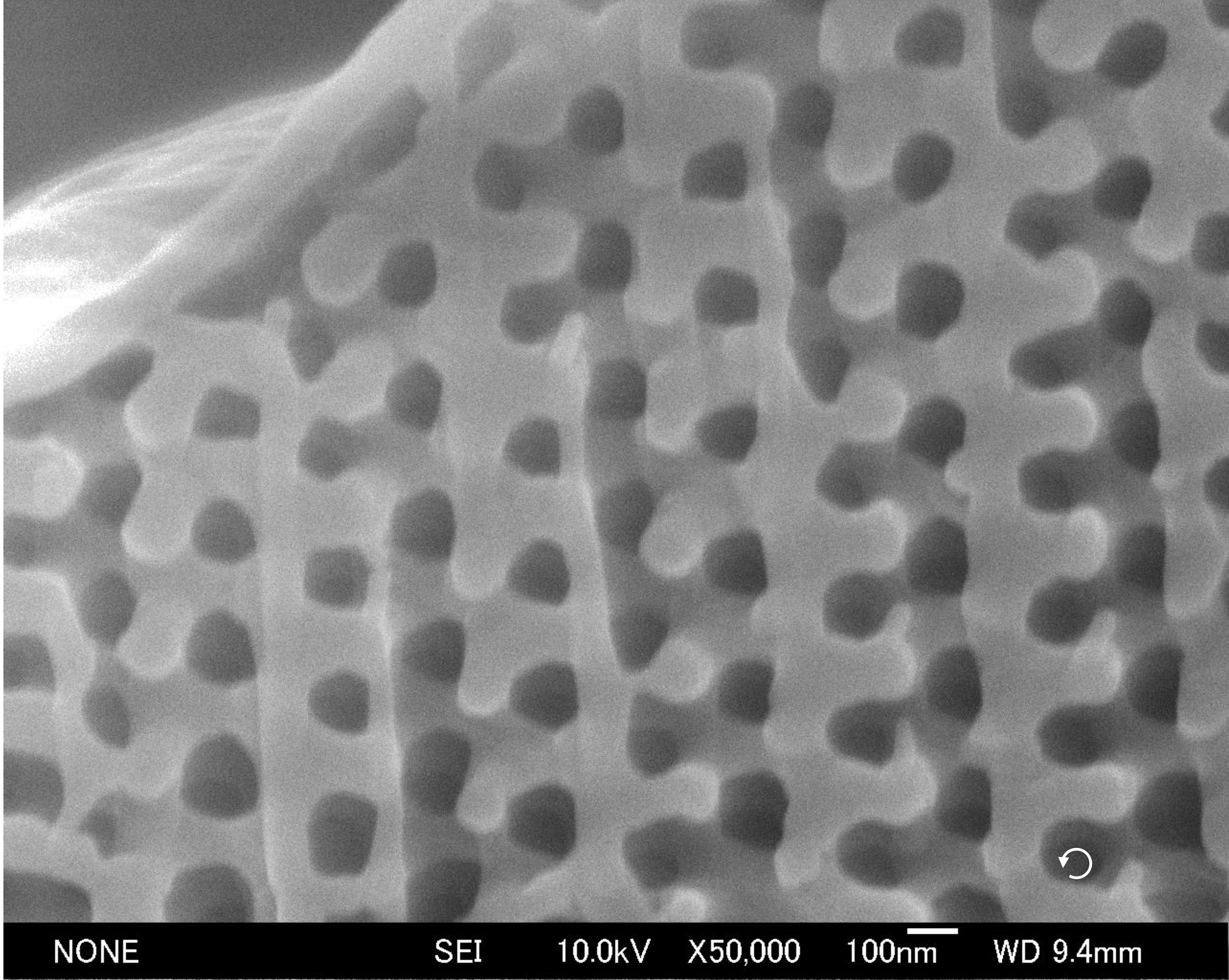

specimen No. 2
scale No. 14
domain No. 2
[111] lh spiral
LH gyroid

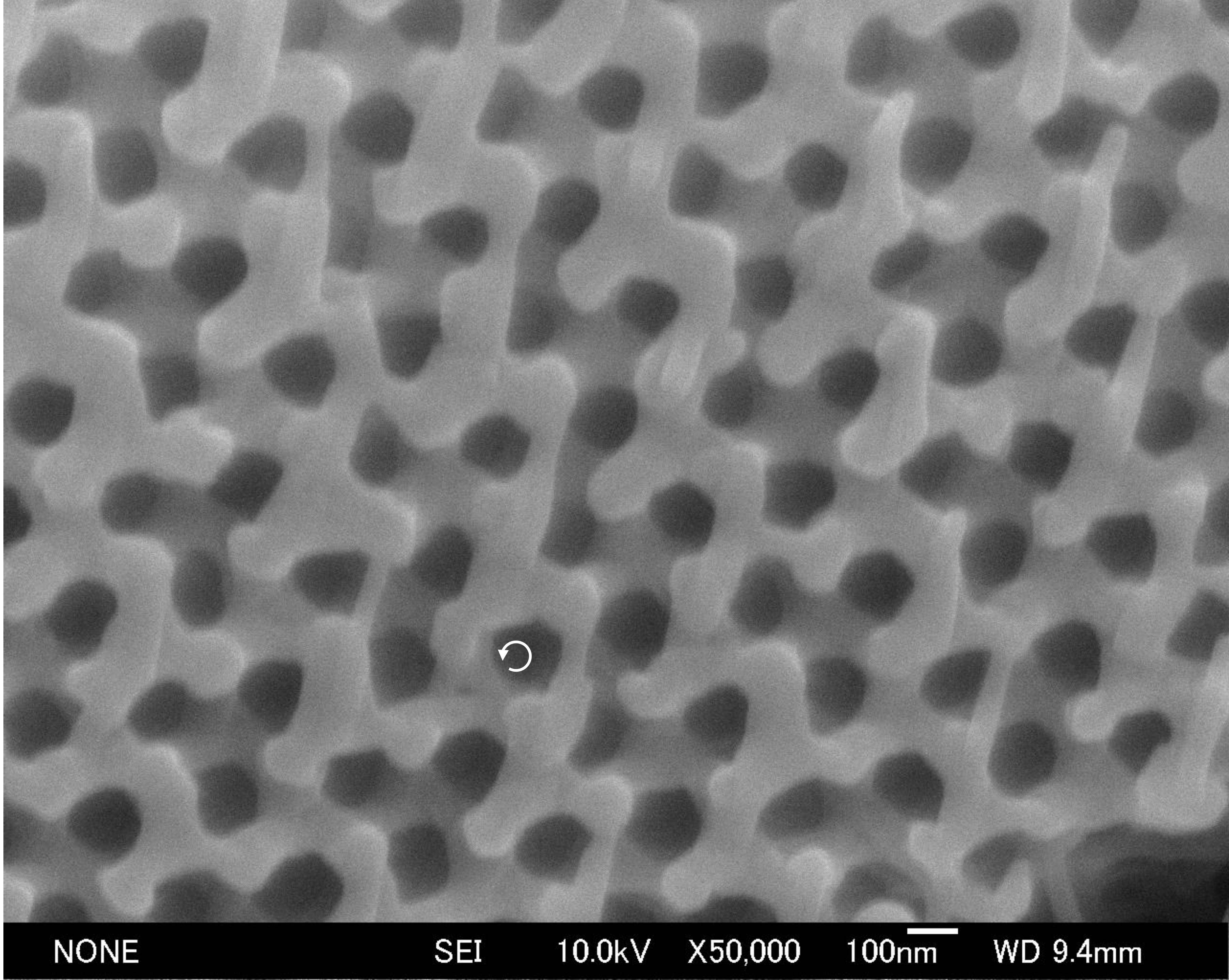

specimen No. 2
scale No. 15

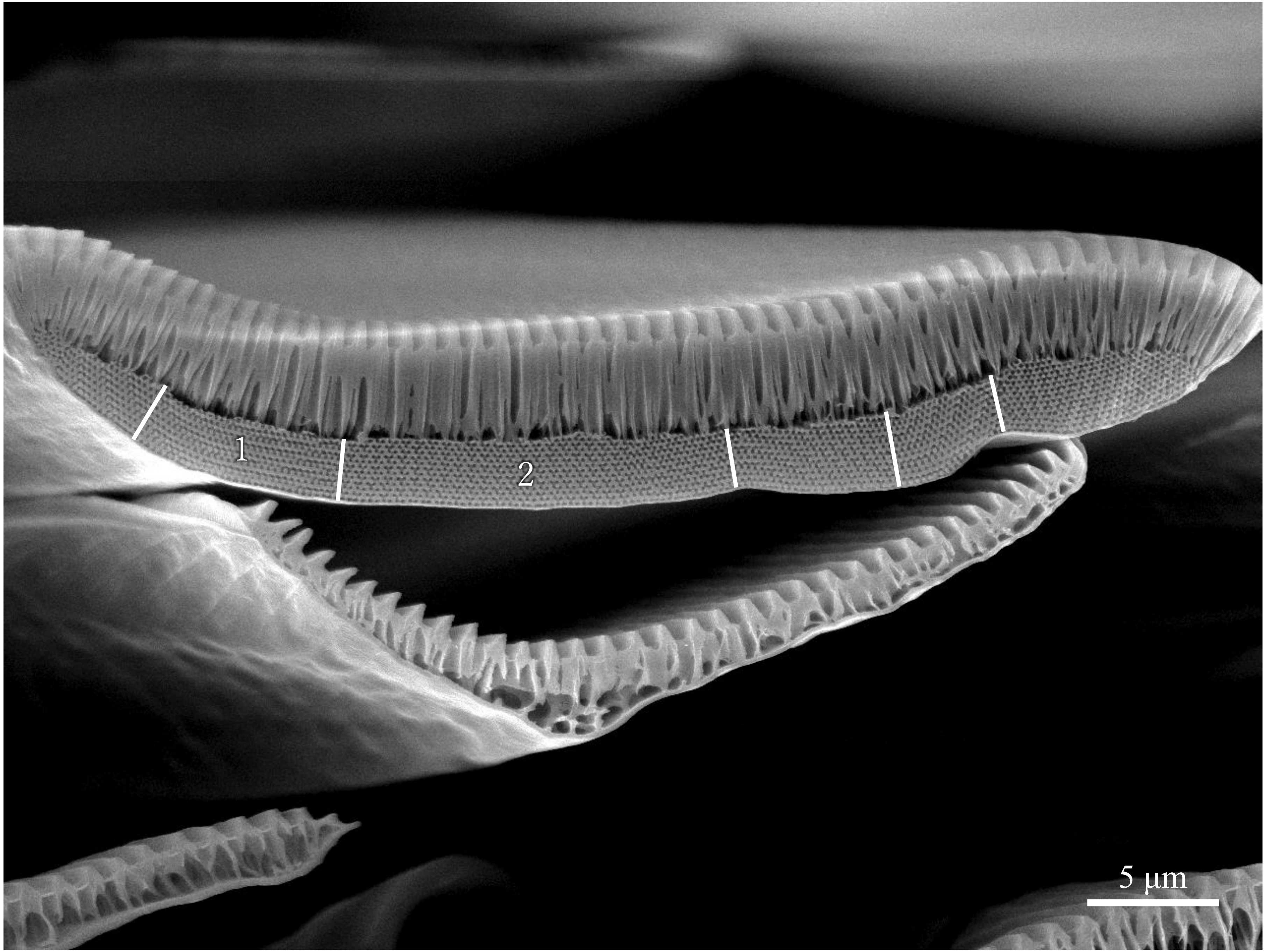

specimen No. 2
scale No. 15
domain No. 1
[111] lh spiral
LH gyroid

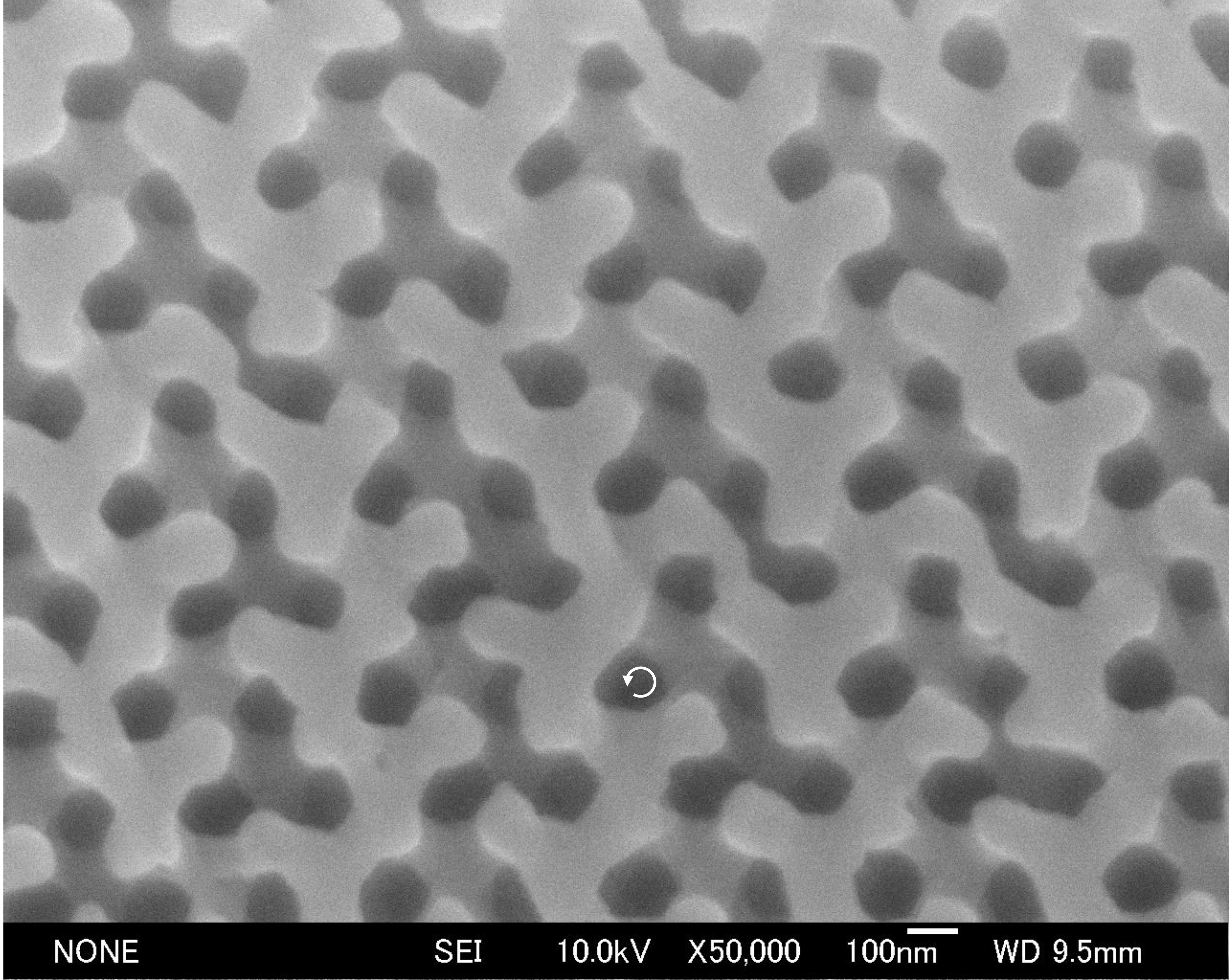

specimen No. 2
scale No. 15
domain No. 2
[111] lh spiral
LH gyroid

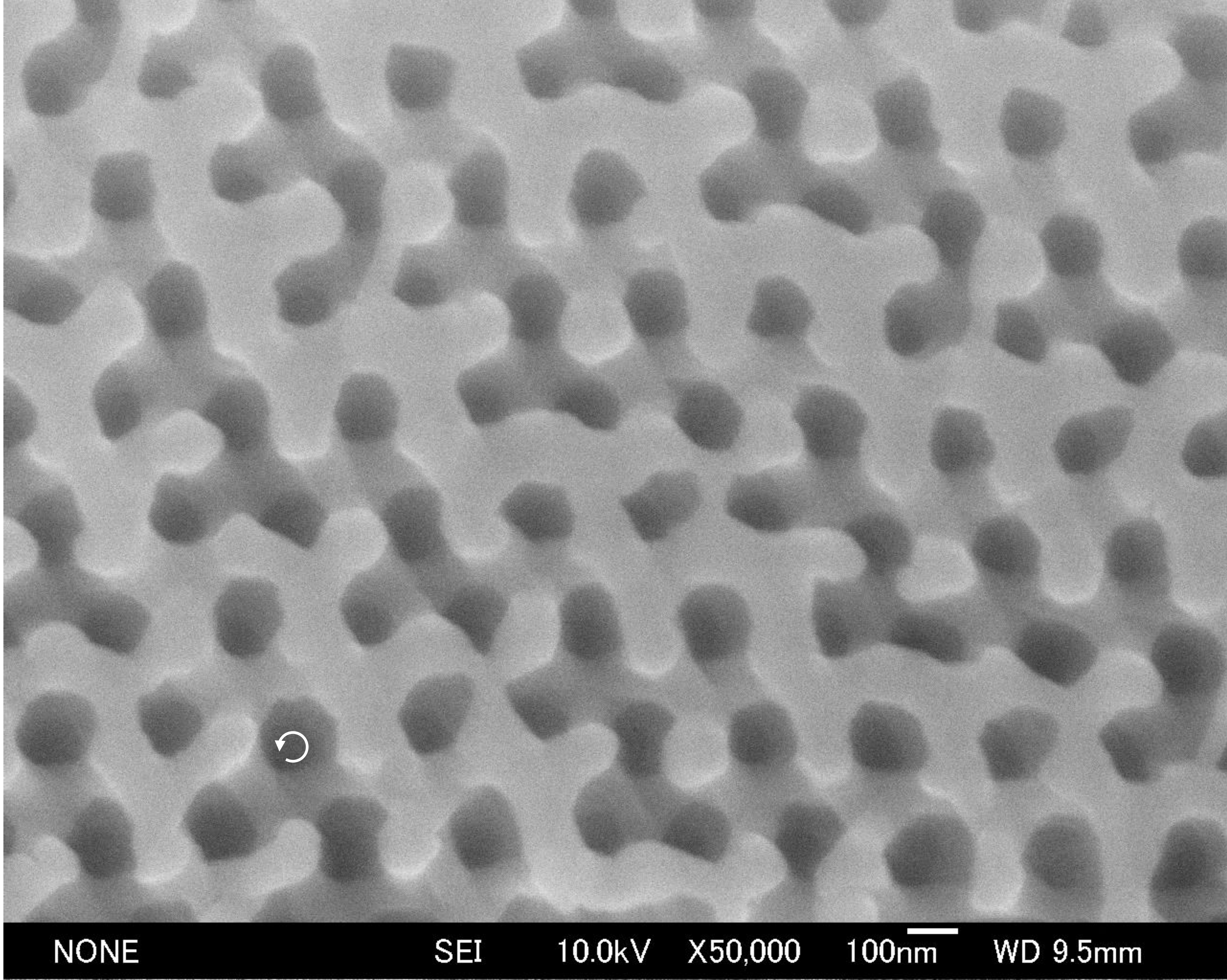

specimen No. 2
scale No. 16

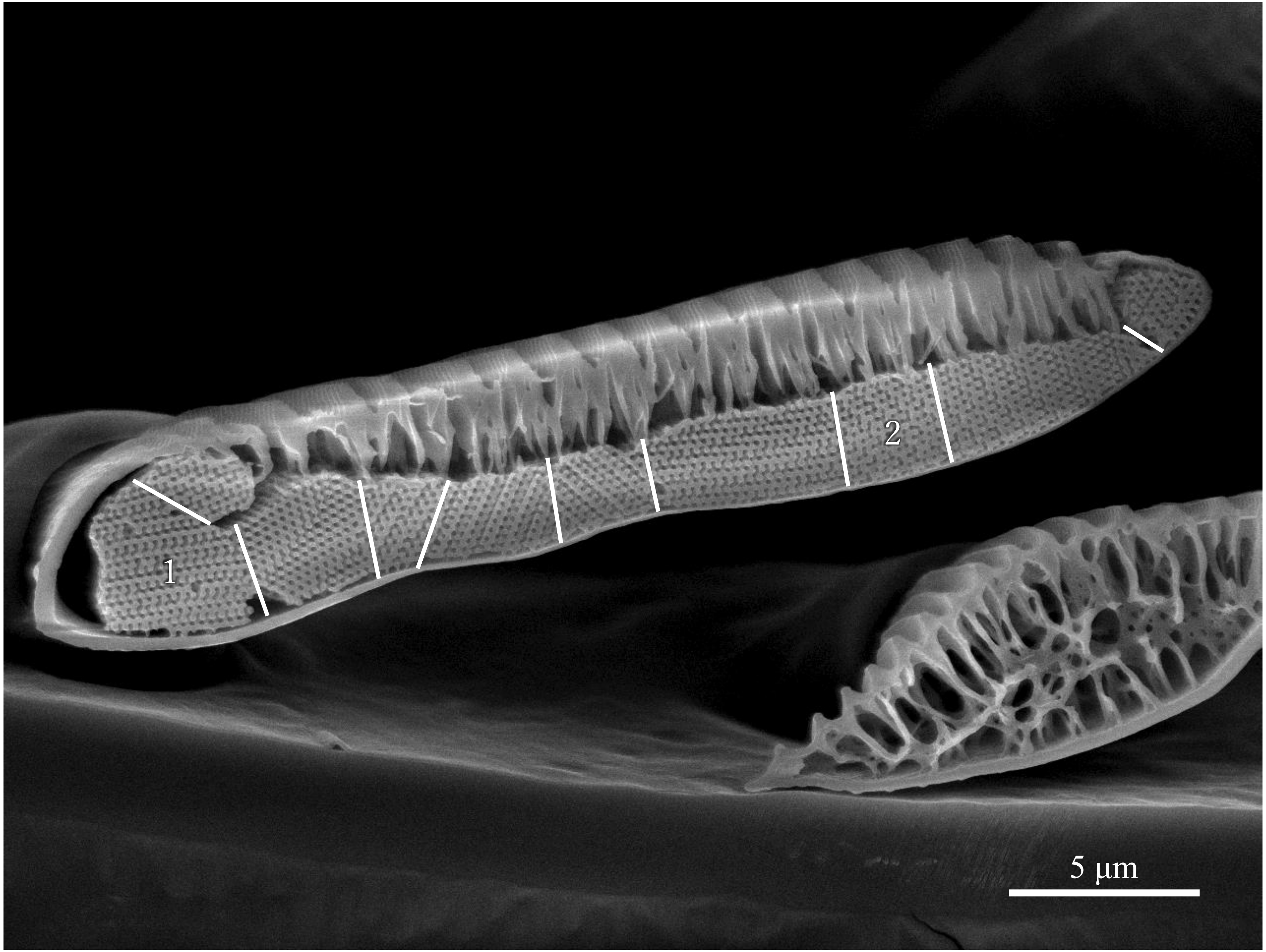

specimen No. 2
scale No. 16
domain No. 1
[111] lh spiral
LH gyroid

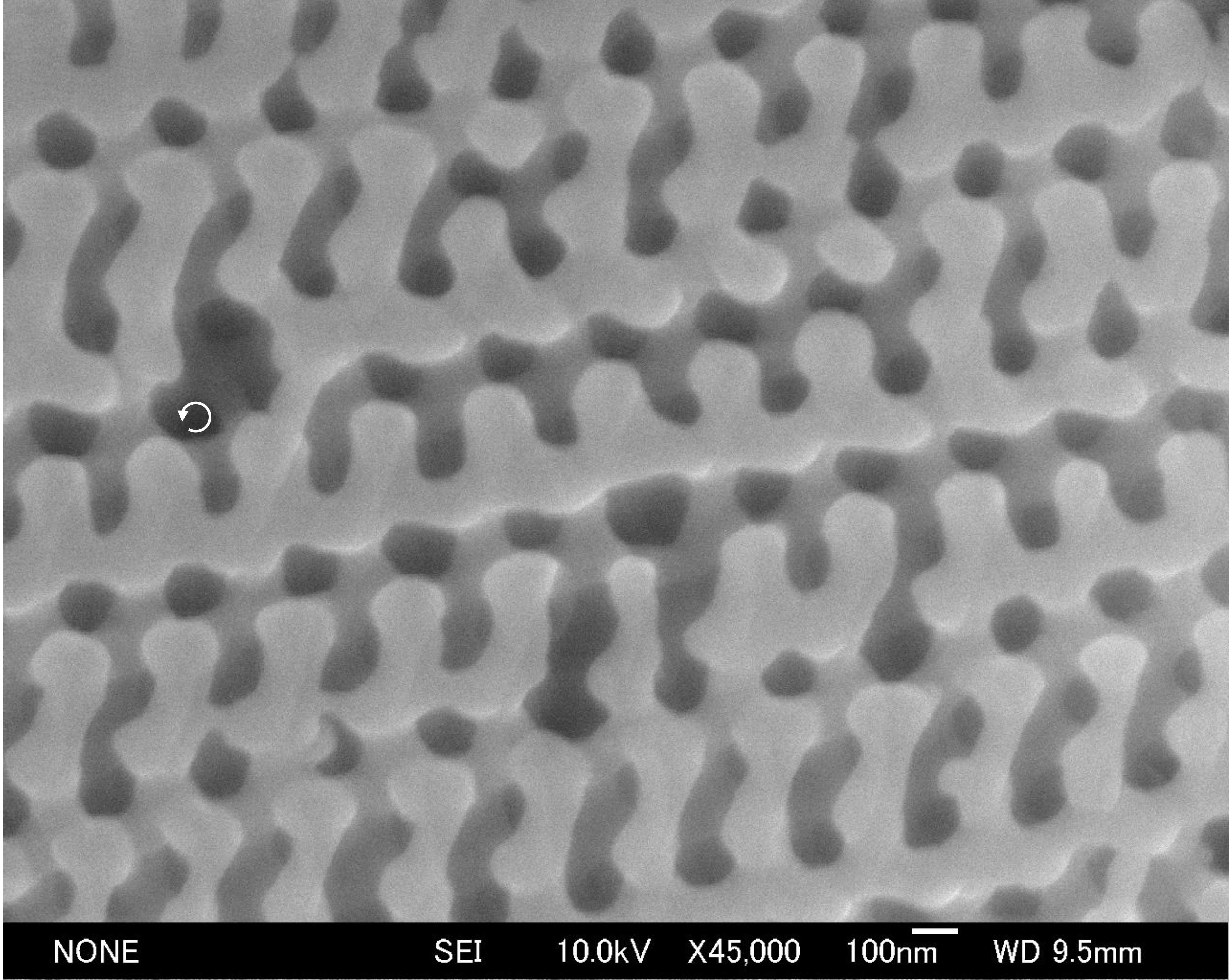

specimen No. 2
scale No. 16
domain No. 2
[100] rh spiral
LH gyroid

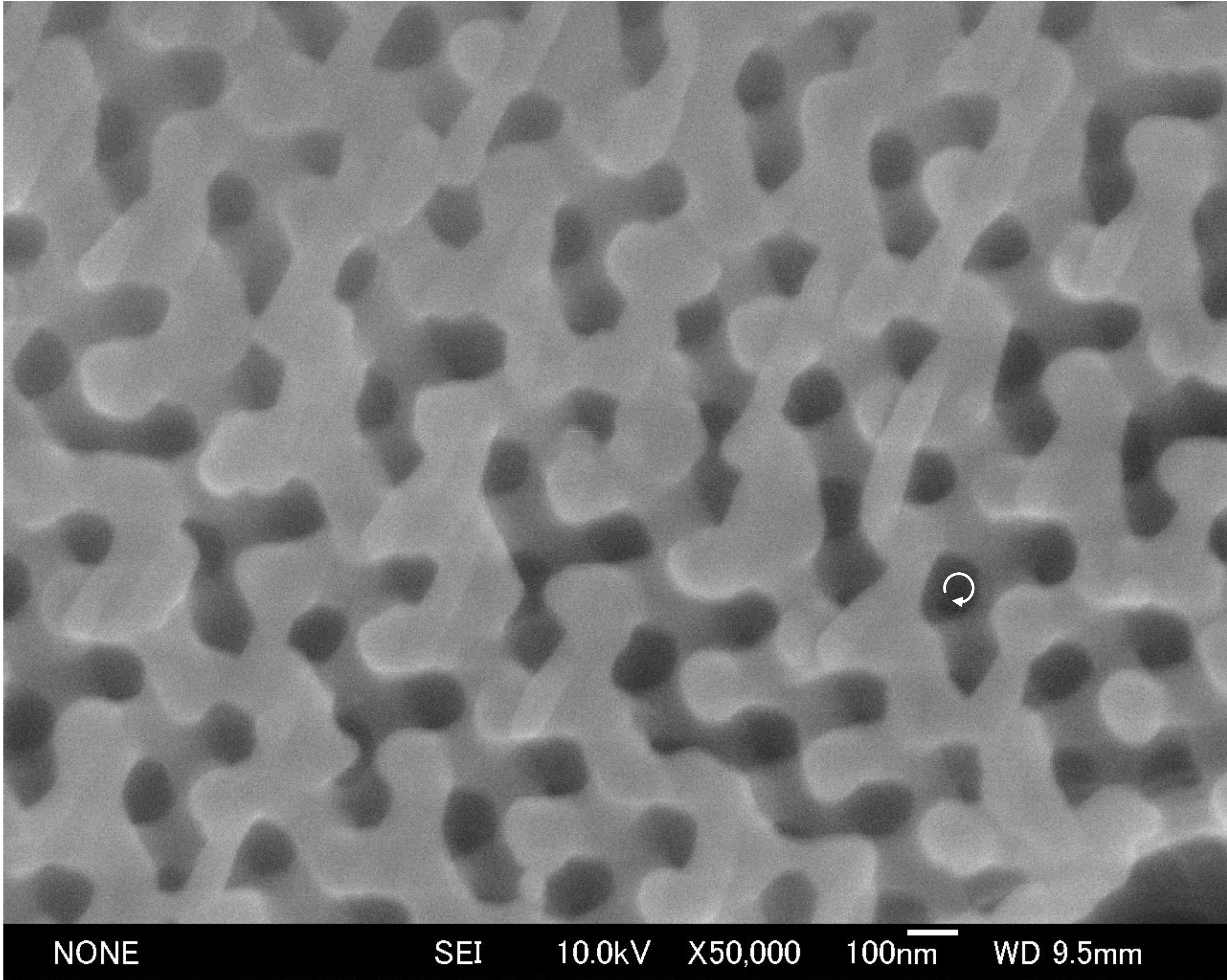

specimen No. 2
scale No. 17

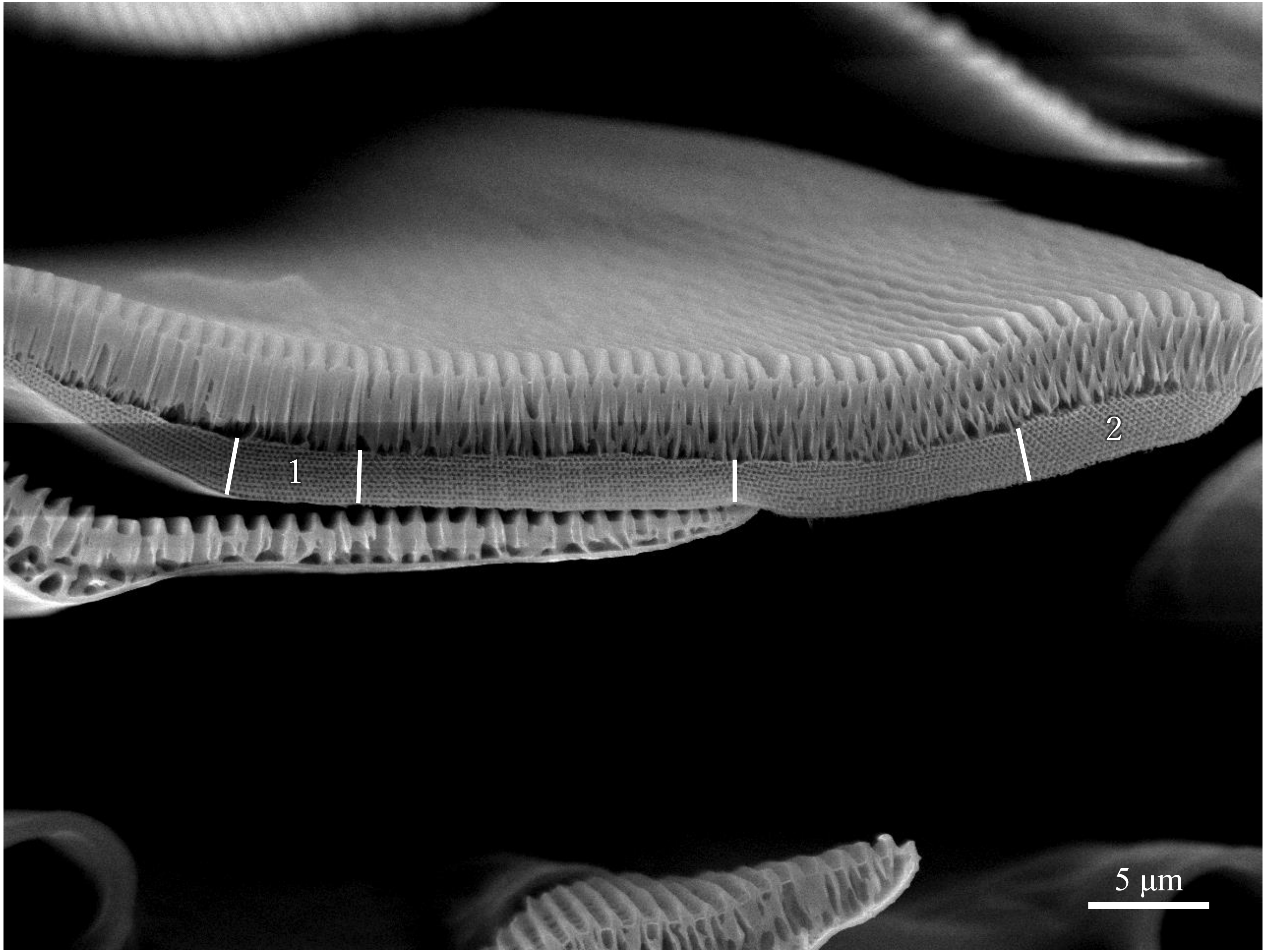

specimen No. 2
scale No. 17
domain No. 1
[111] rh spiral
RH gyroid

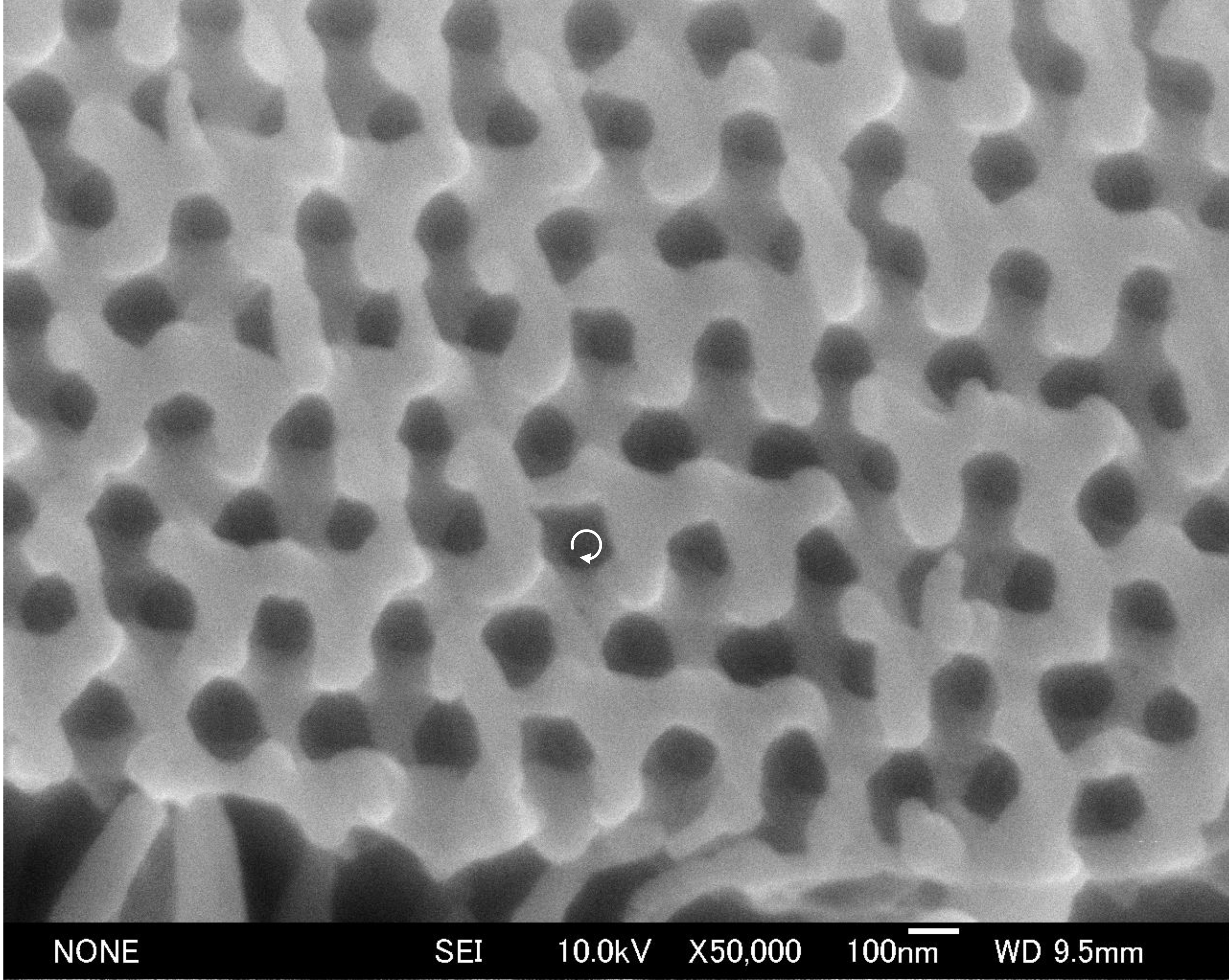

specimen No. 2
scale No. 17
domain No. 2
[111] rh spiral
RH gyroid

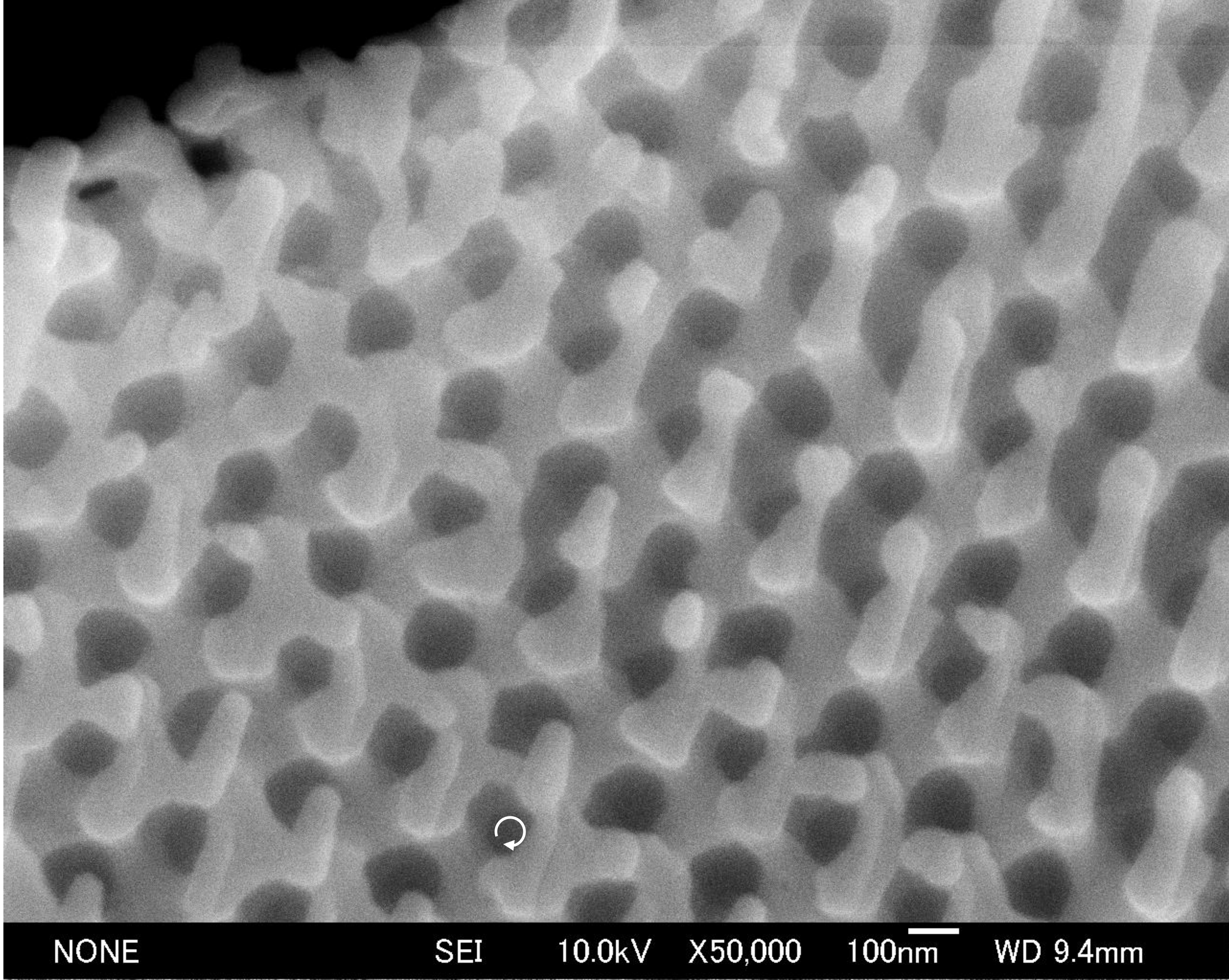

specimen No. 2
scale No. 18

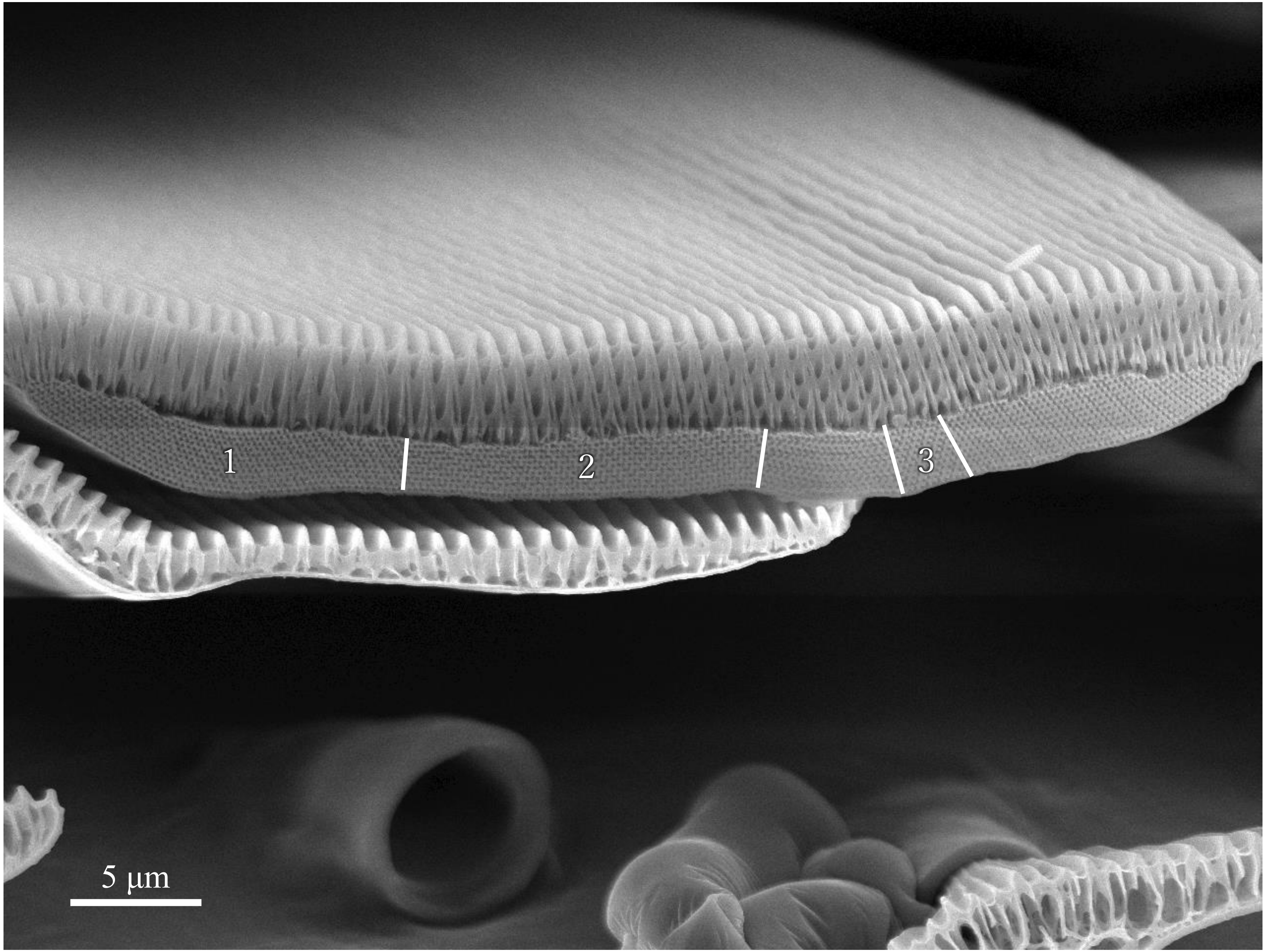

specimen No. 2
scale No. 18
domain No. 1
[111] lh spiral
LH gyroid

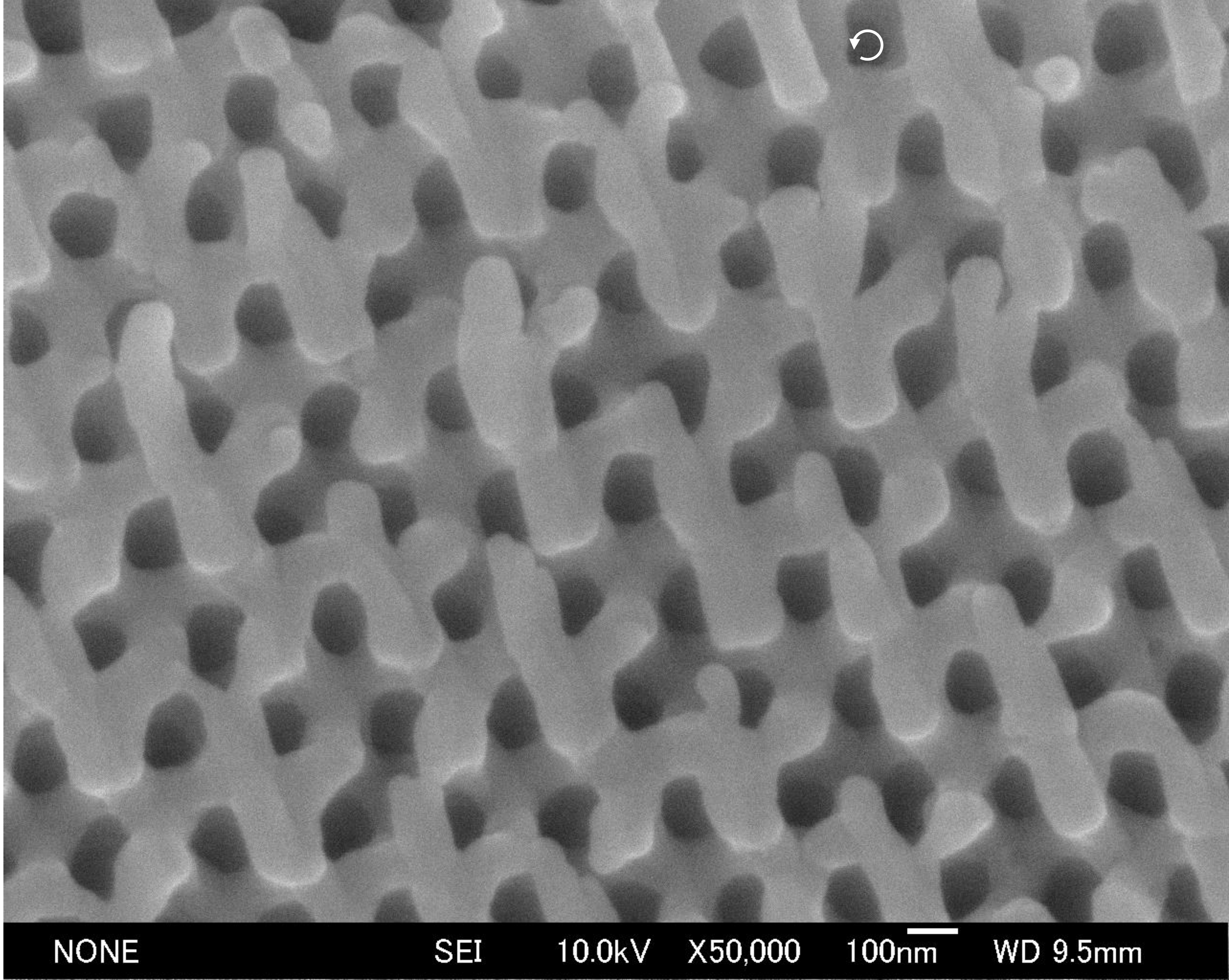

specimen No. 2
scale No. 18
domain No. 2
[100] rh spiral
LH gyroid

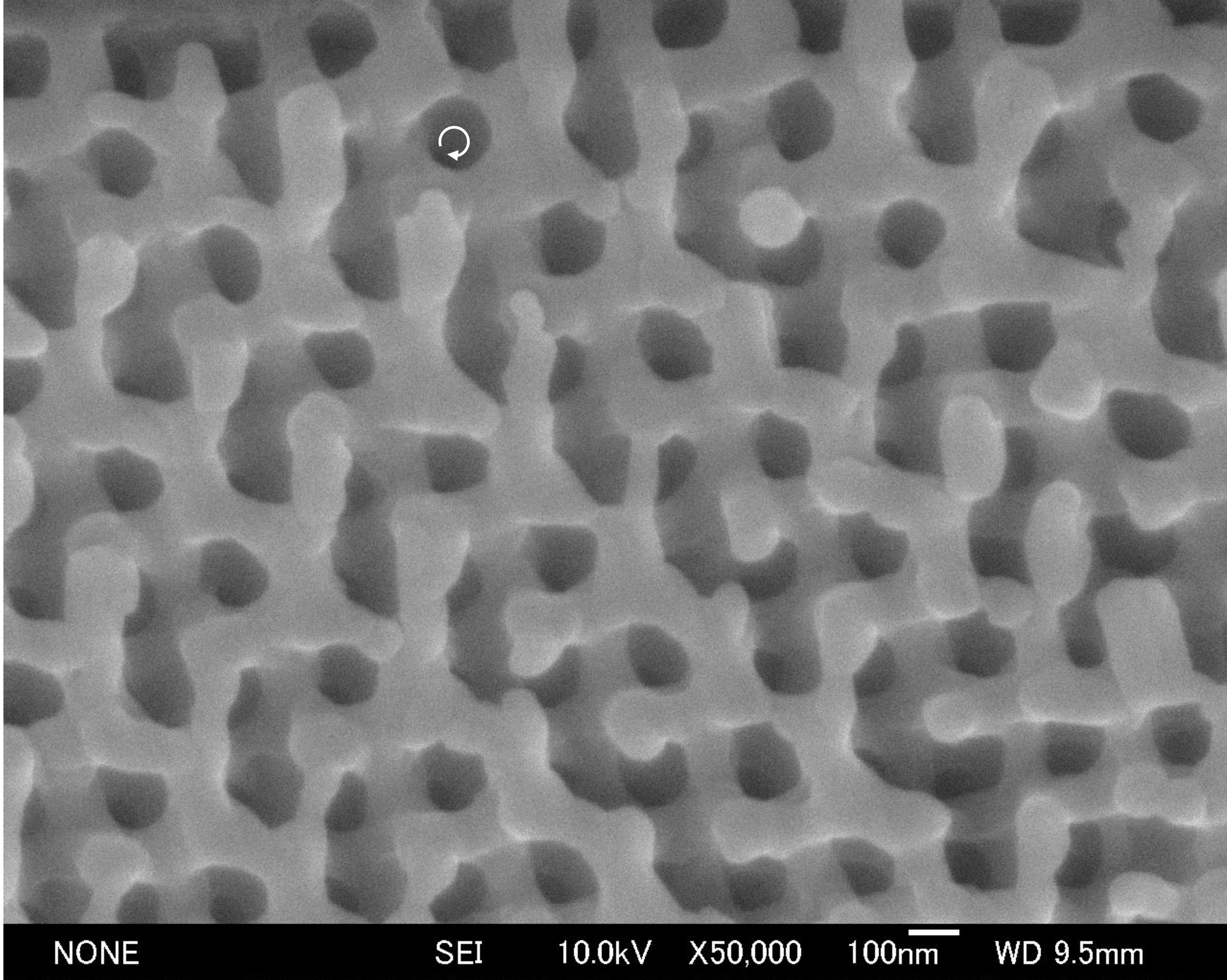

specimen No. 2
scale No. 18
domain No. 3
[111] rh spiral
RH gyroid

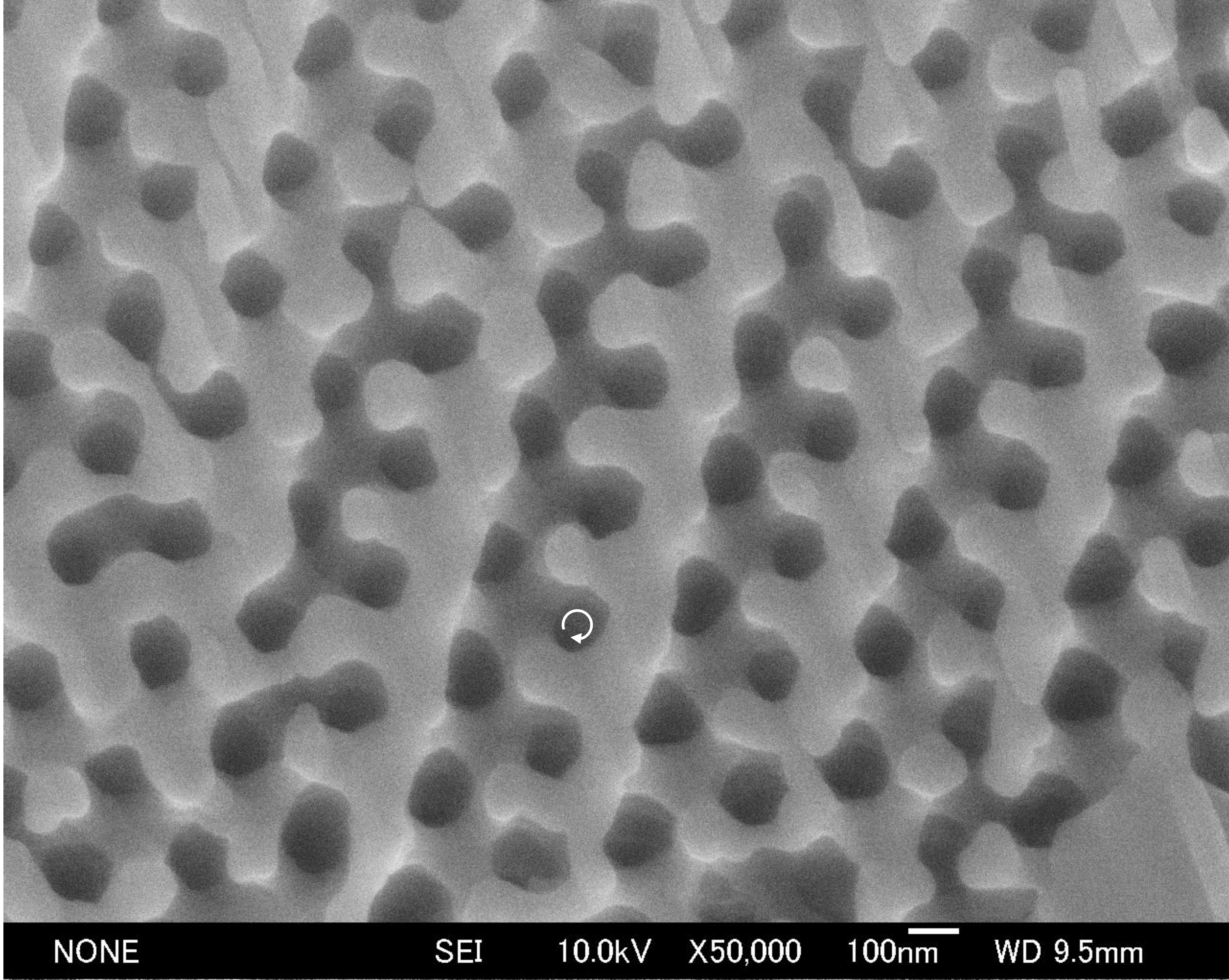

specimen No. 2
scale No. 19

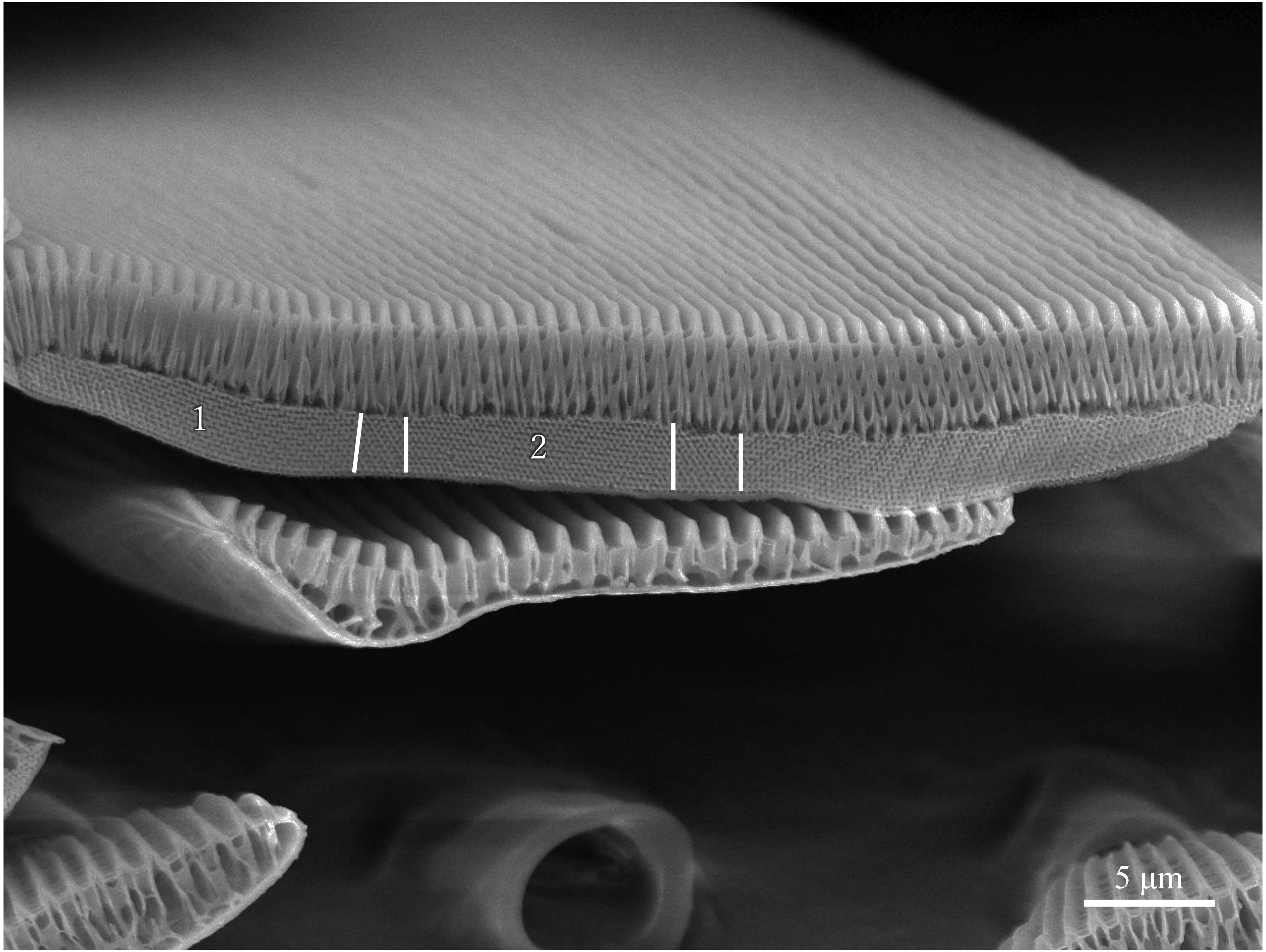

specimen No. 2
scale No. 19
domain No. 1
[100] rh spiral
LH gyroid

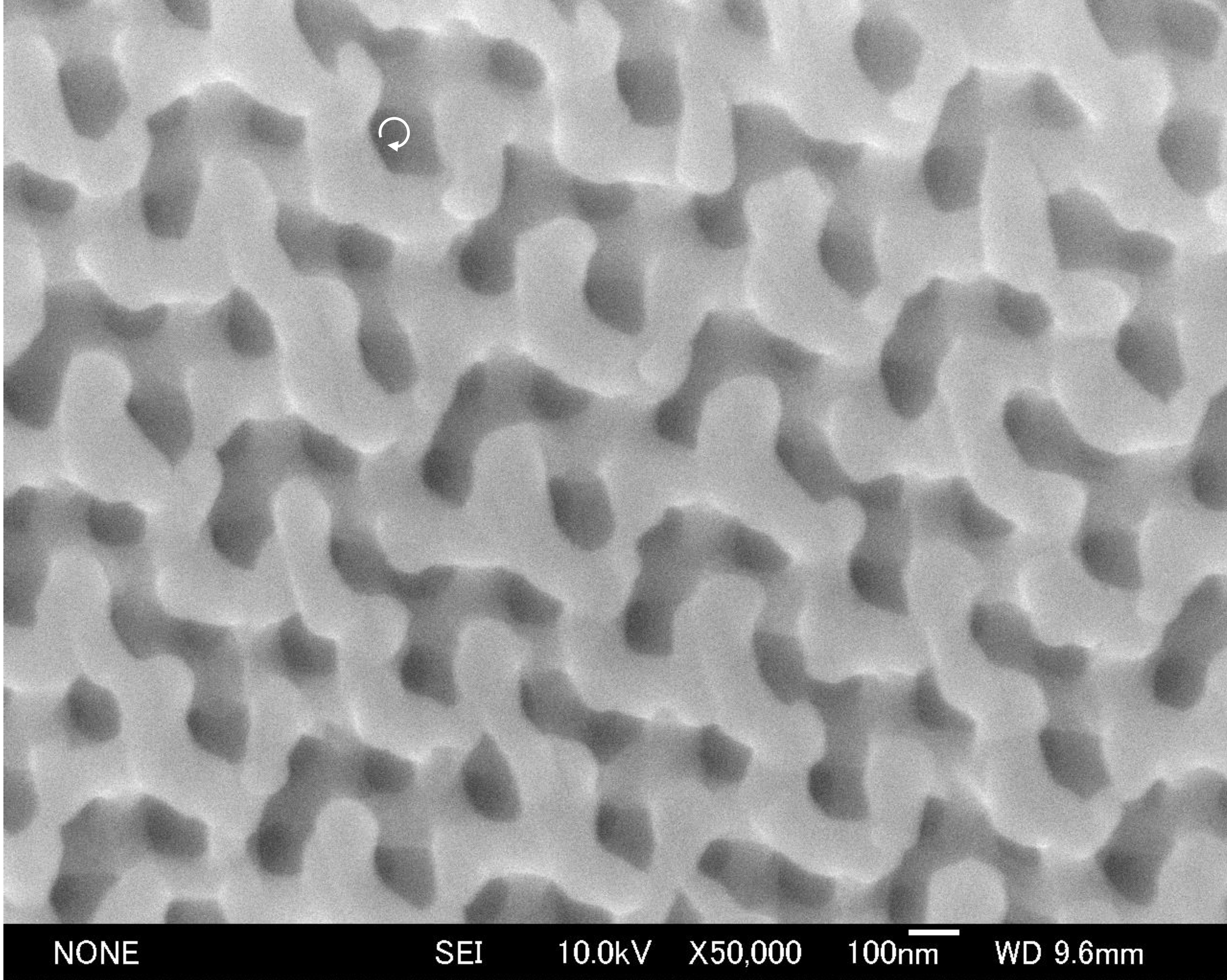

specimen No. 2
scale No. 19
domain No. 2
[111] lh spiral
LH gyroid

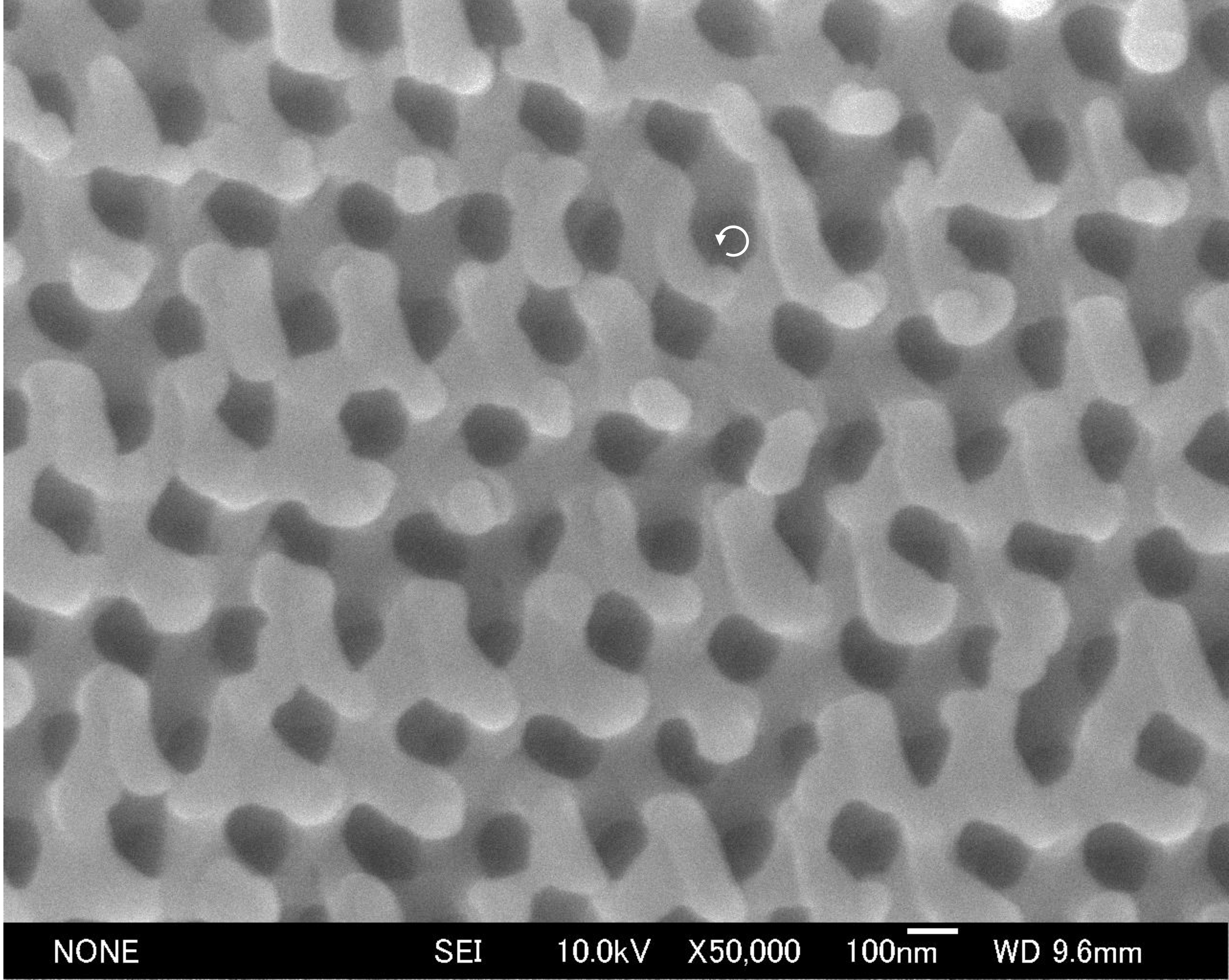

specimen No. 2
scale No. 20

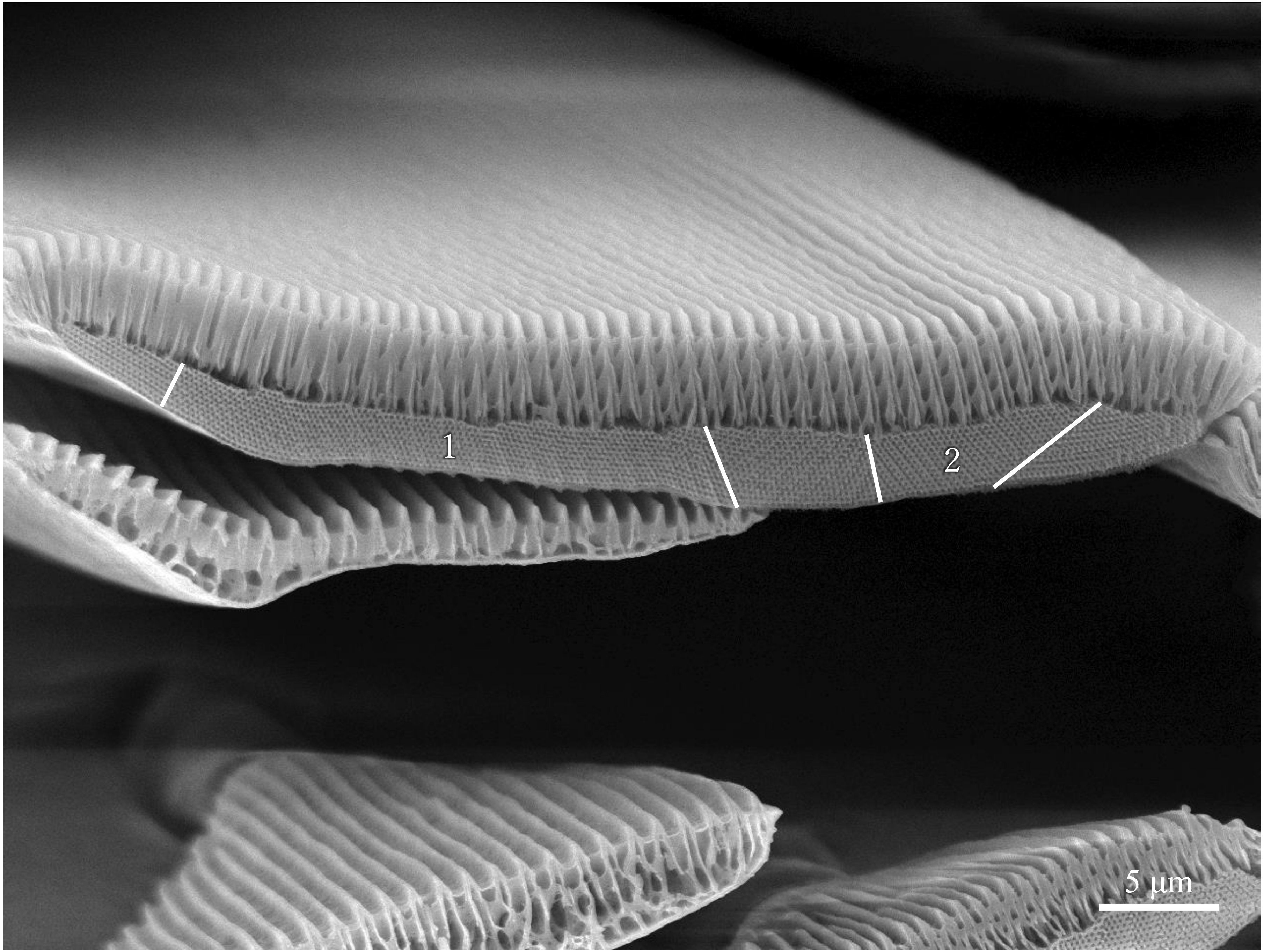

specimen No. 2
scale No. 20
domain No. 1
[111] lh spiral
LH gyroid

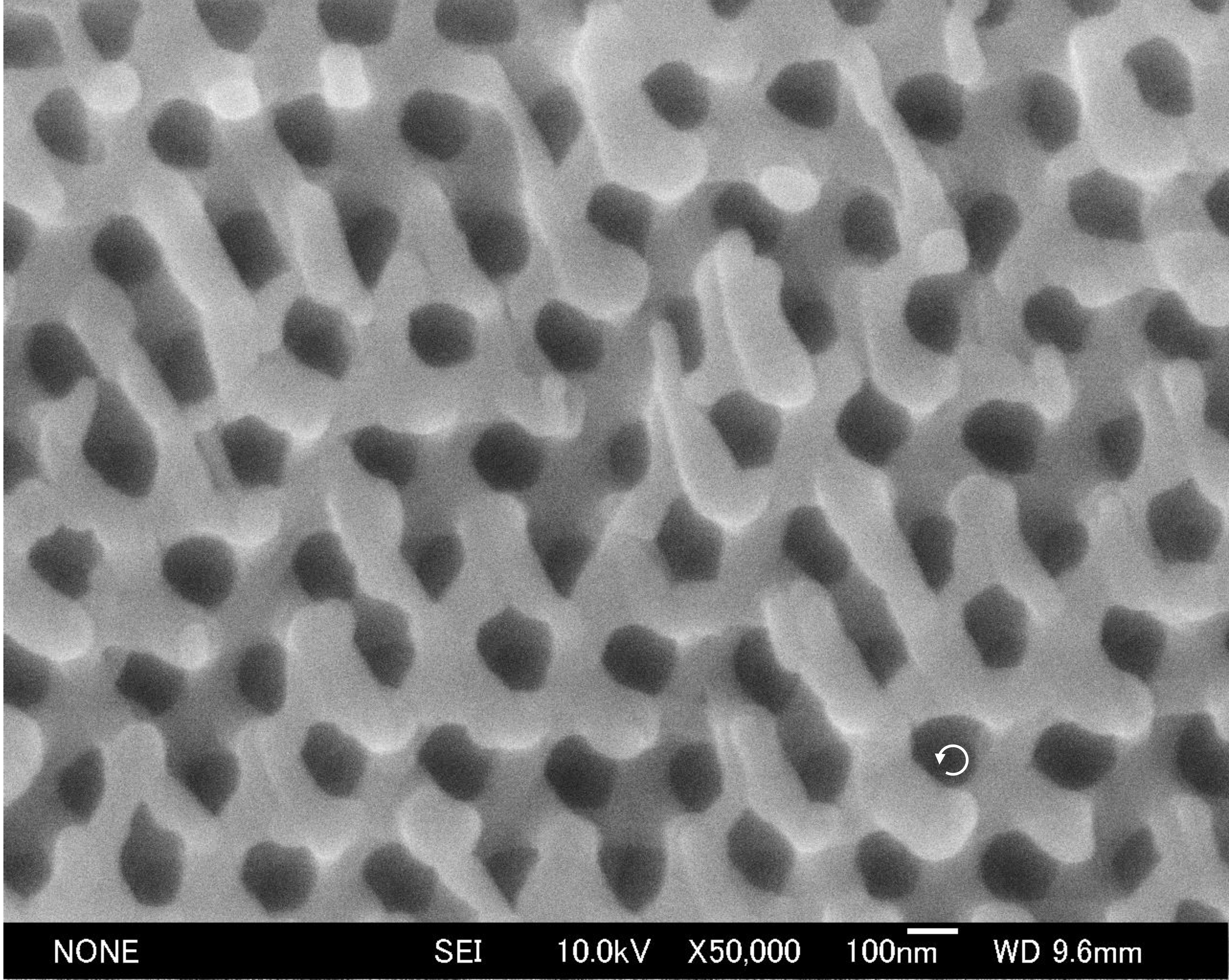

specimen No. 2
scale No. 20
domain No. 2
[111] lh spiral
LH gyroid

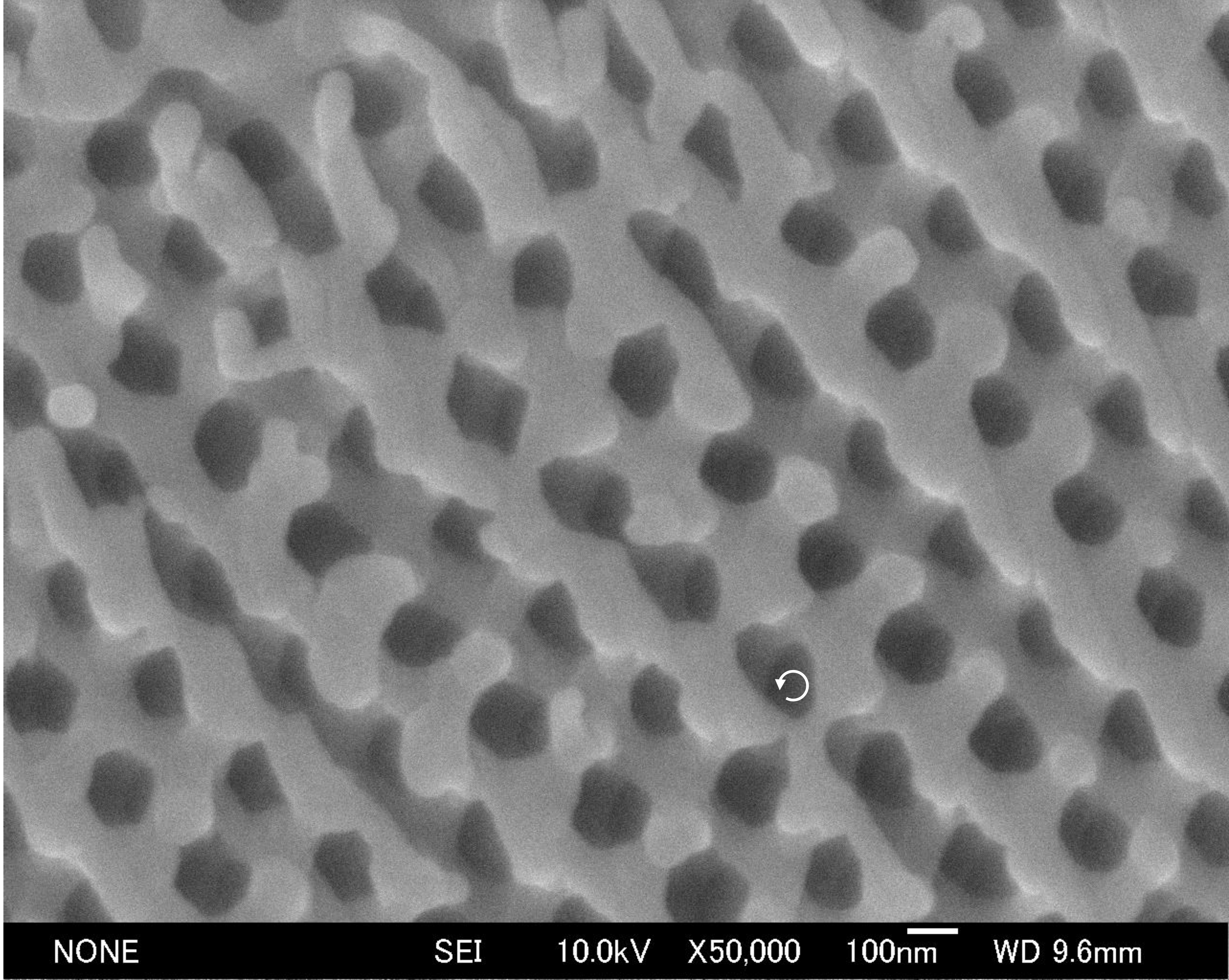

specimen No. 2
scale No. 21

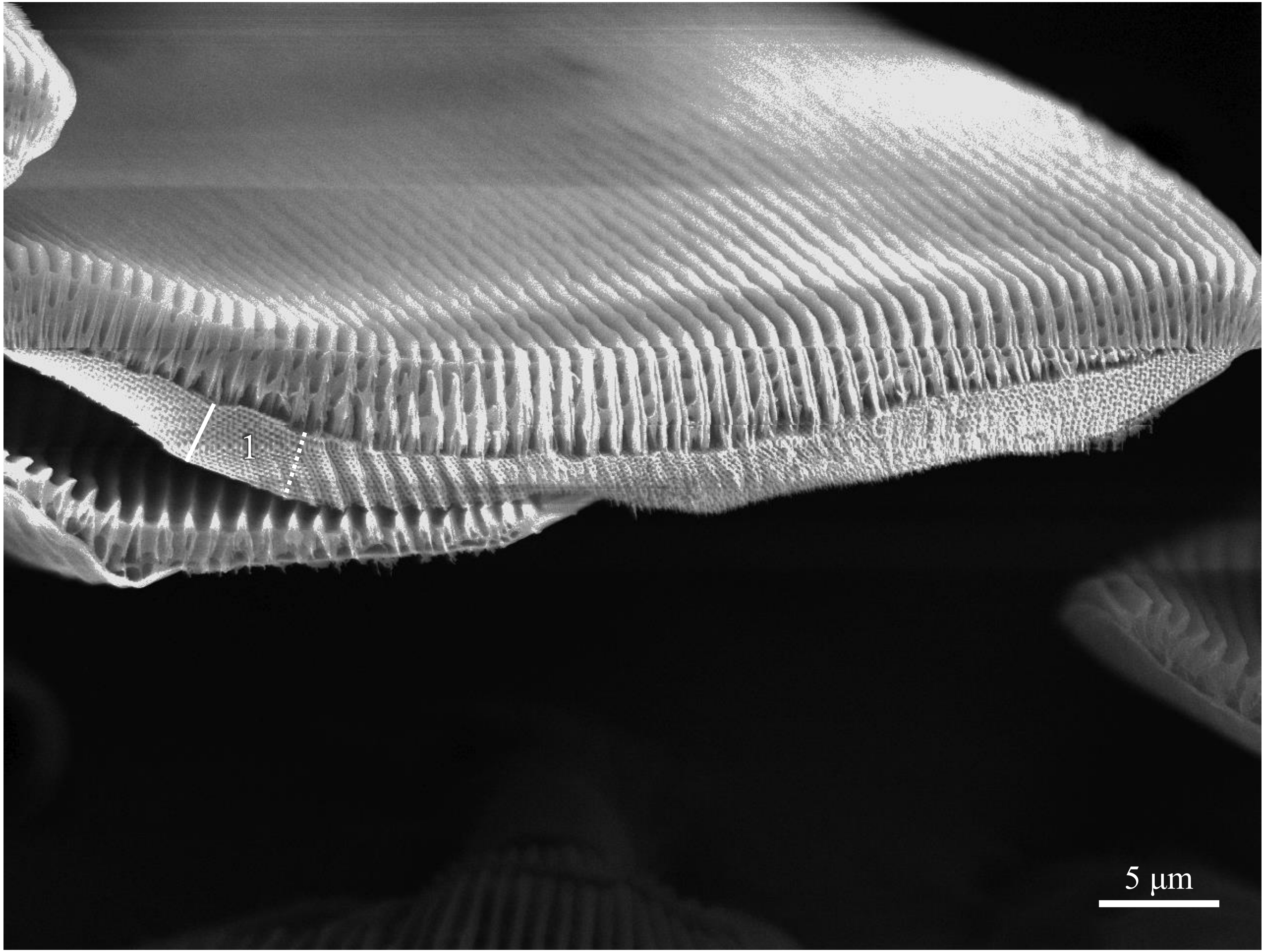

specimen No. 2
scale No. 21
domain No. 1
[111] lh spiral
LH gyroid

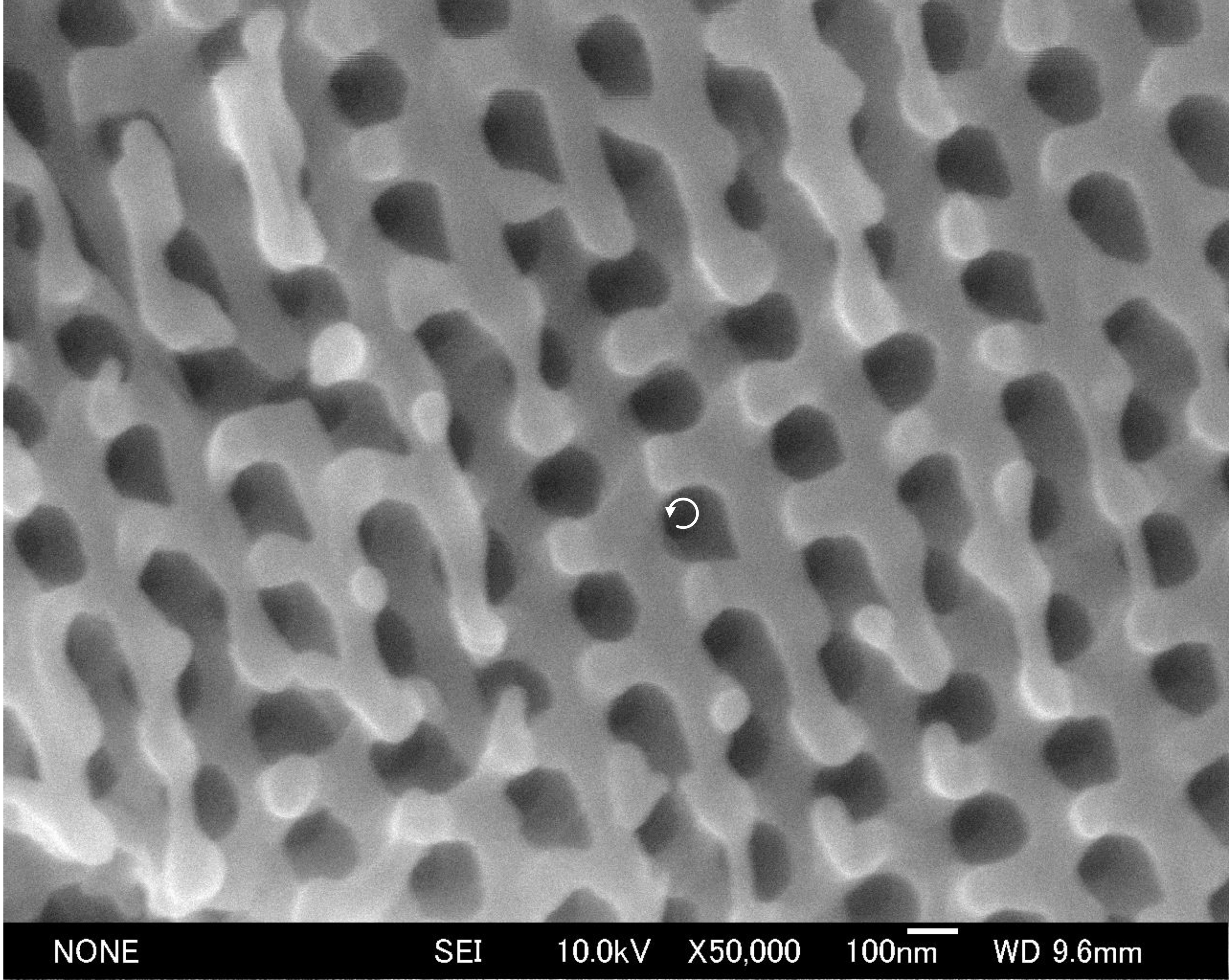

specimen No. 2
scale No. 22

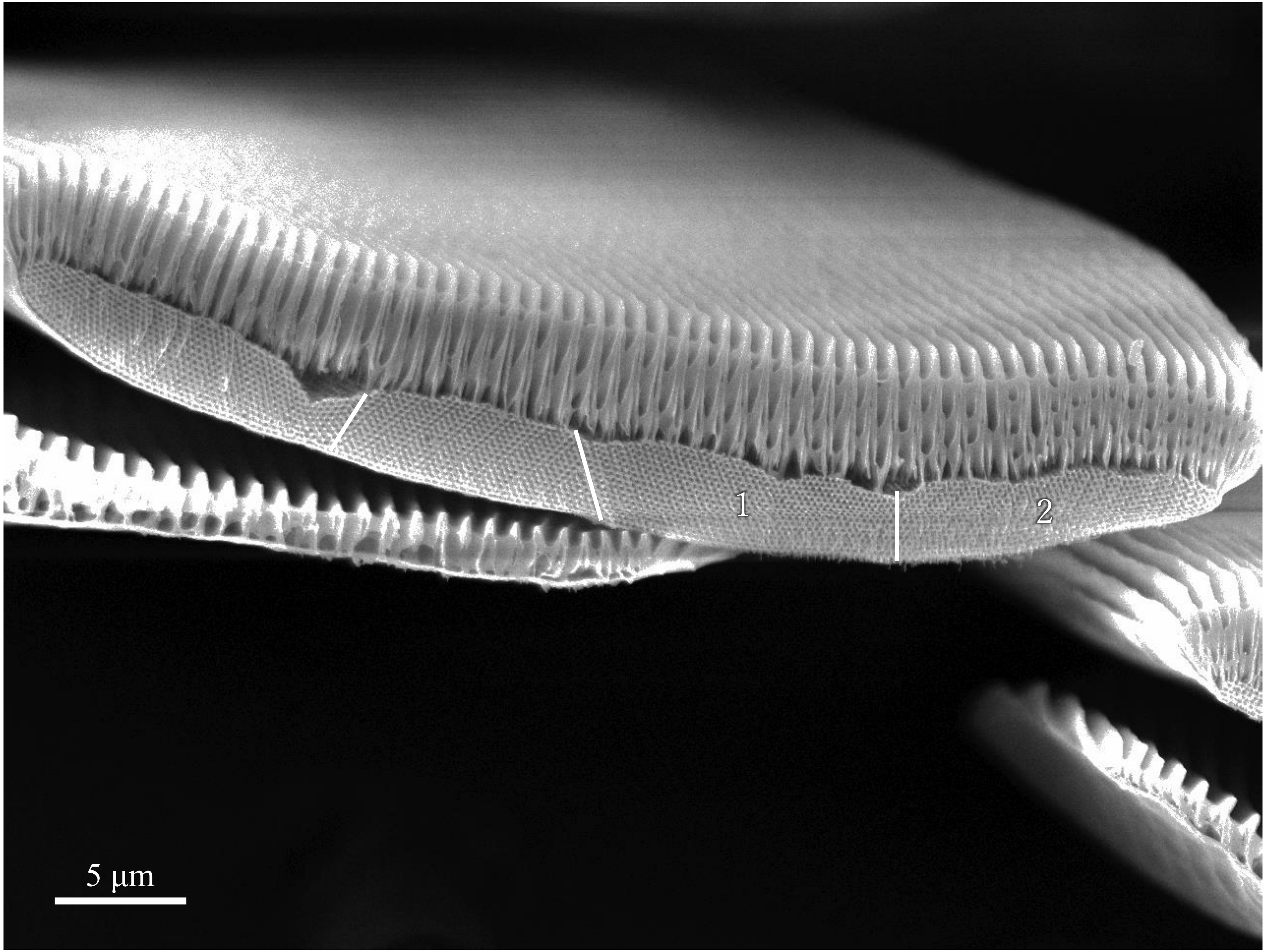

specimen No. 2
scale No. 22
domain No. 1
[111] lh spiral
LH gyroid

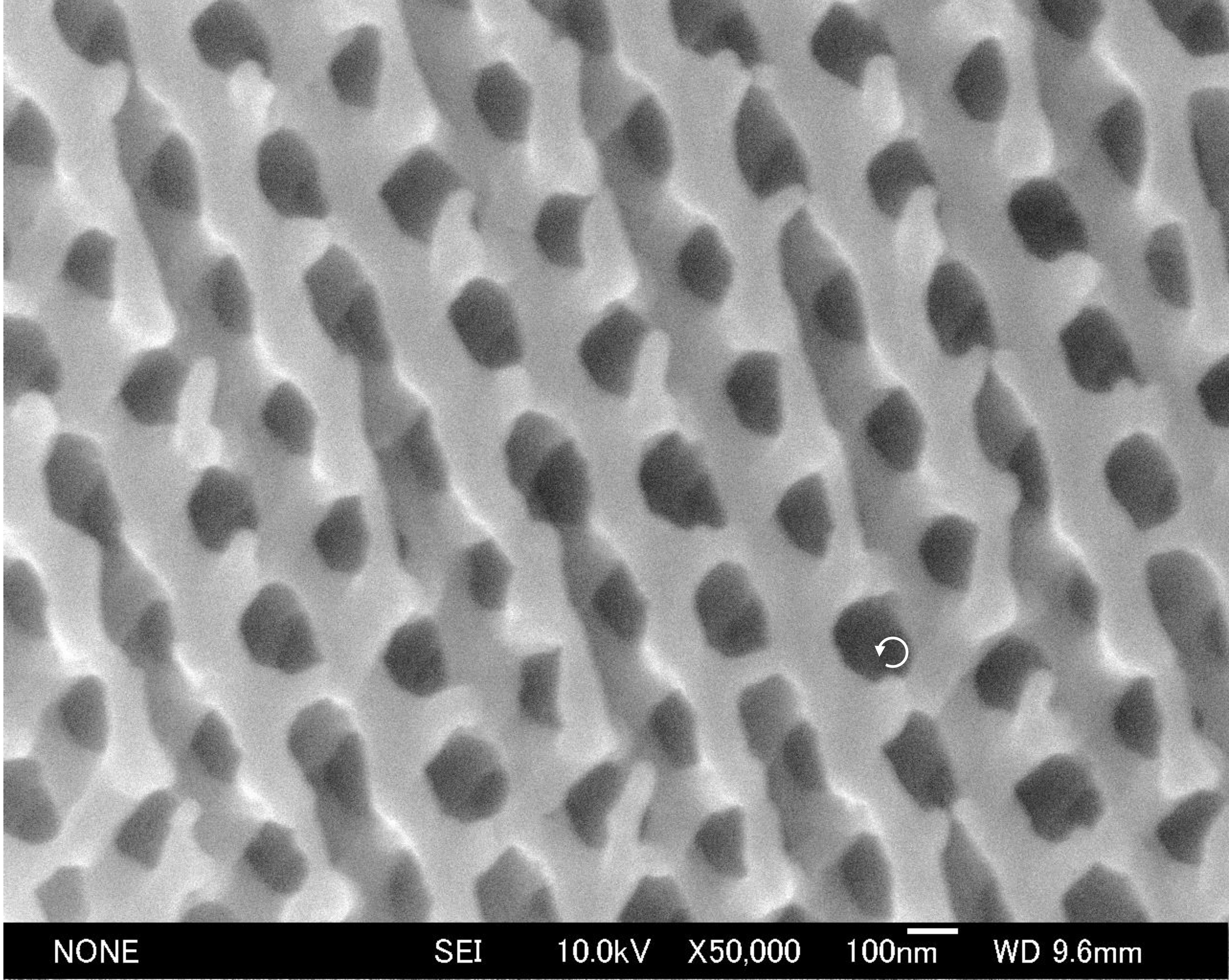

specimen No. 2
scale No. 22
domain No. 2
[111] lh spiral
LH gyroid

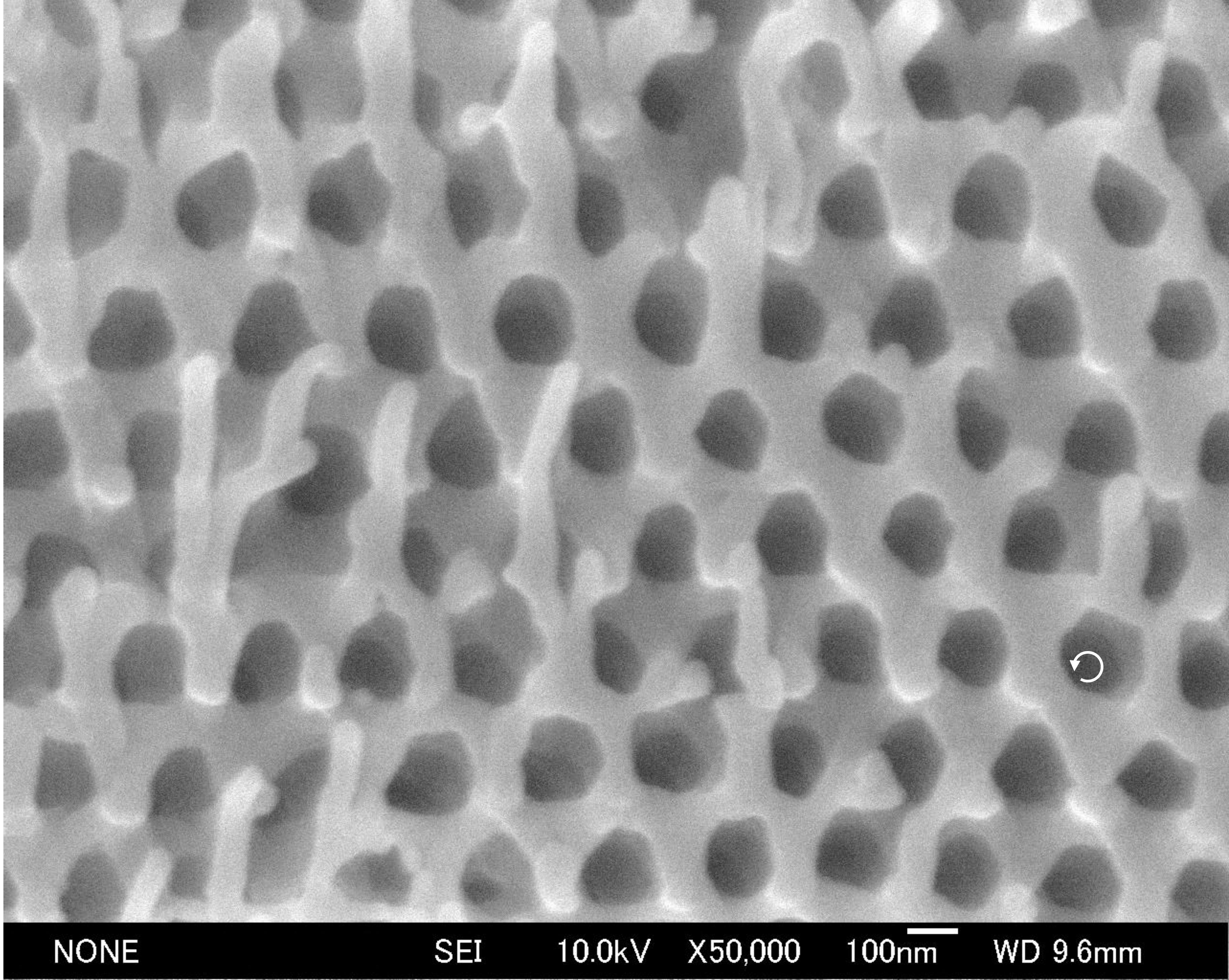

specimen No. 2
scale No. 23

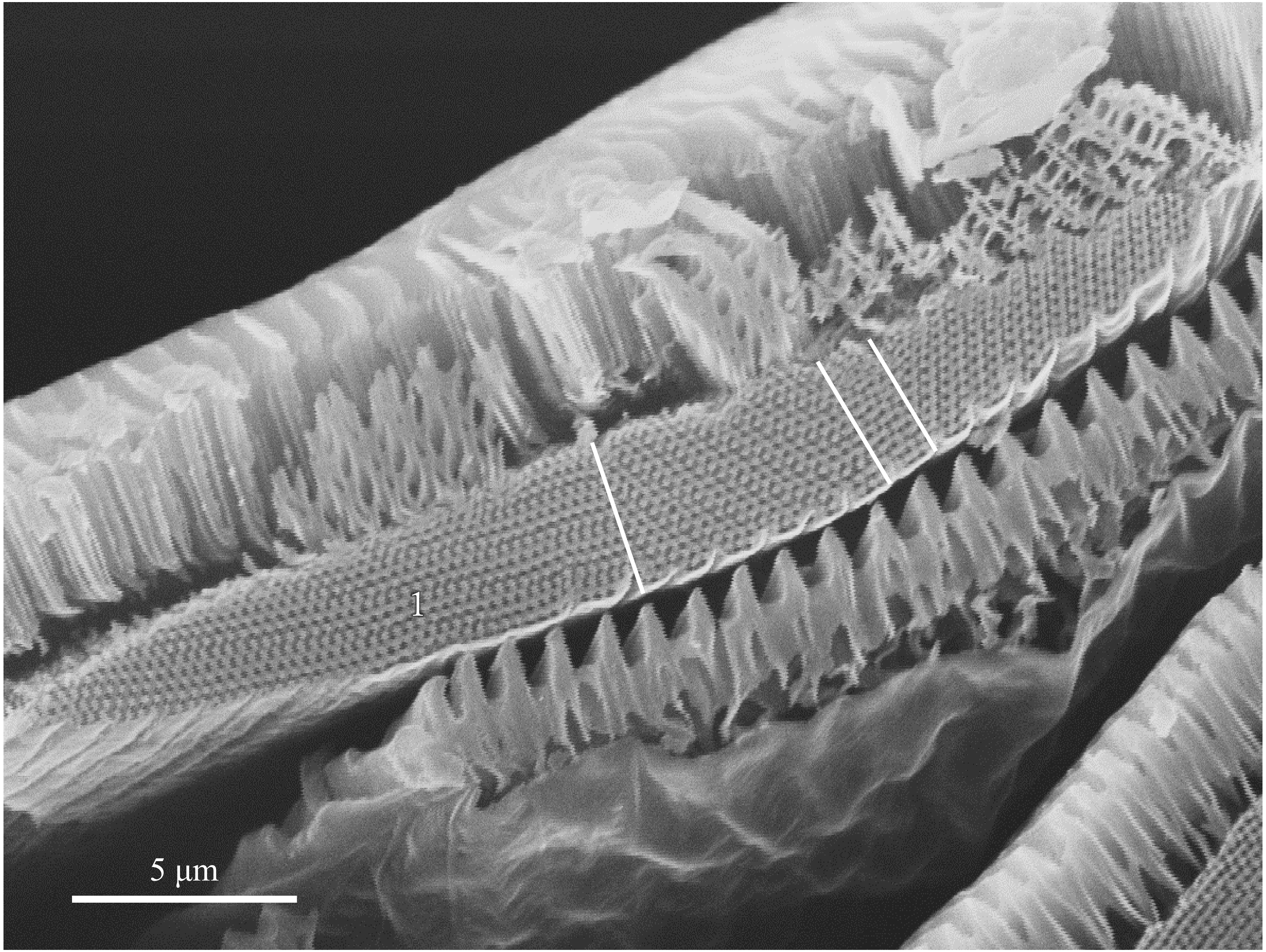

specimen No. 2
scale No. 23
domain No. 1
[111] lh spiral
LH gyroid

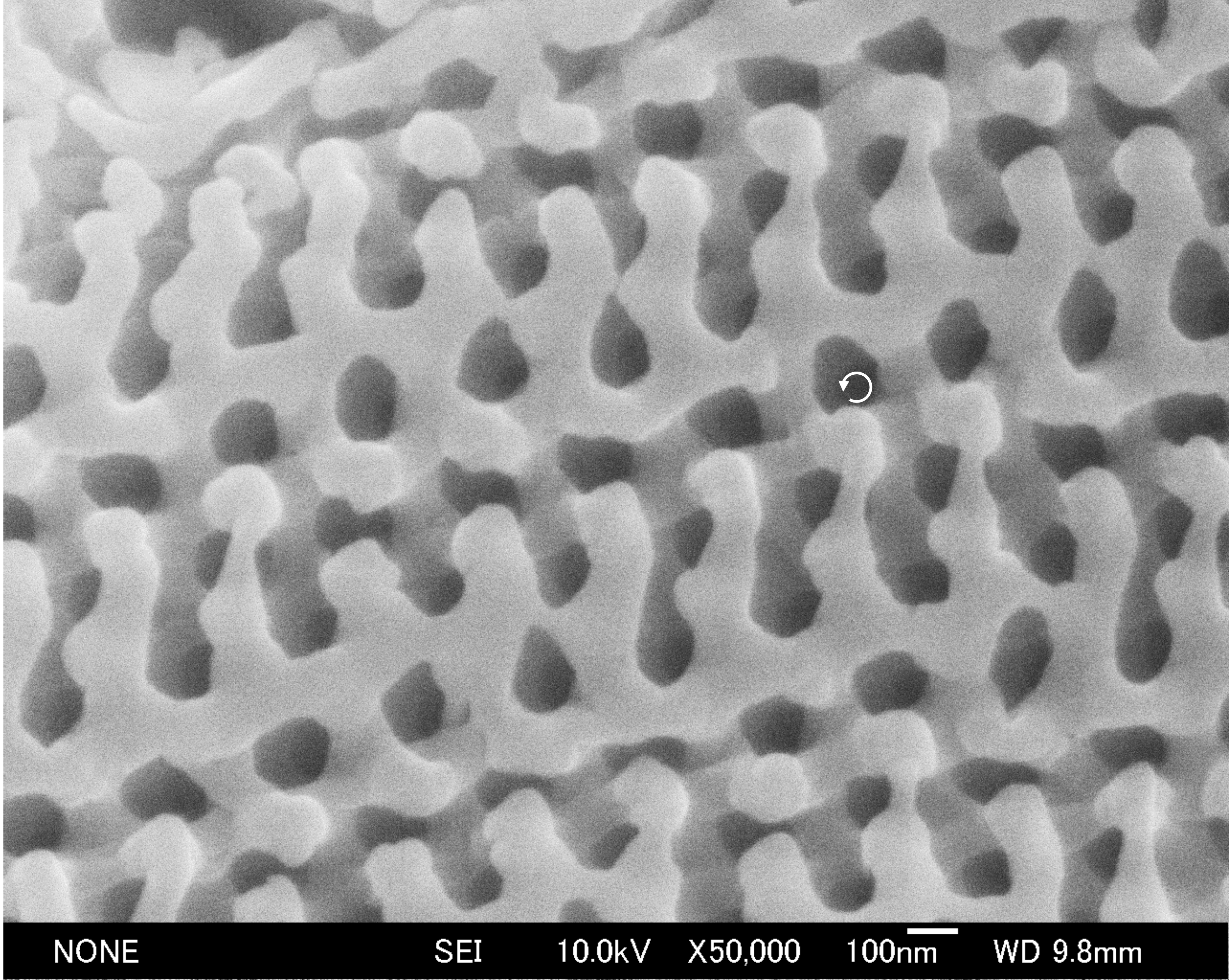

specimen No. 2
scale No. 24

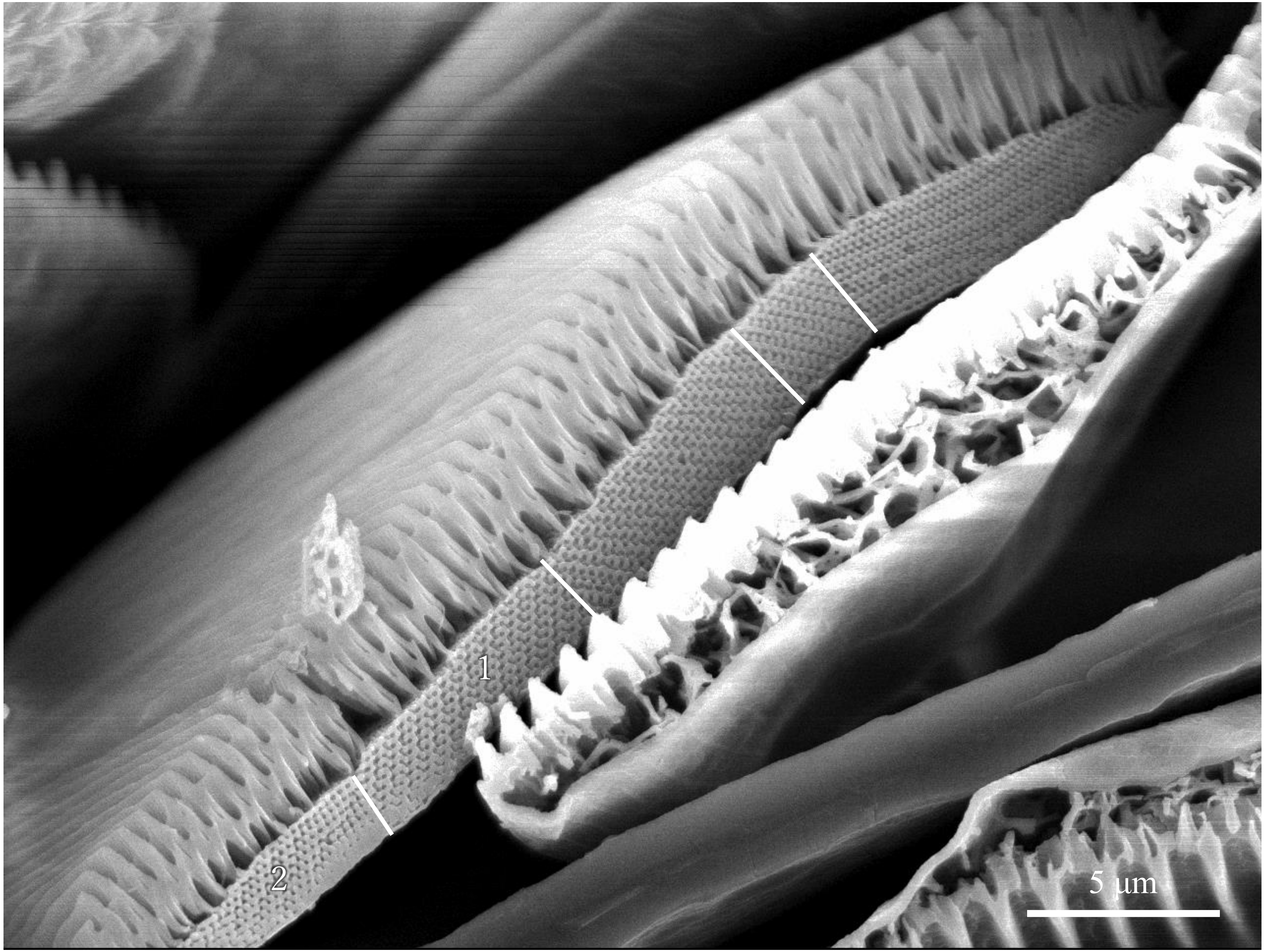

specimen No. 2
scale No. 24
domain No. 1
[100] rh spiral
LH gyroid

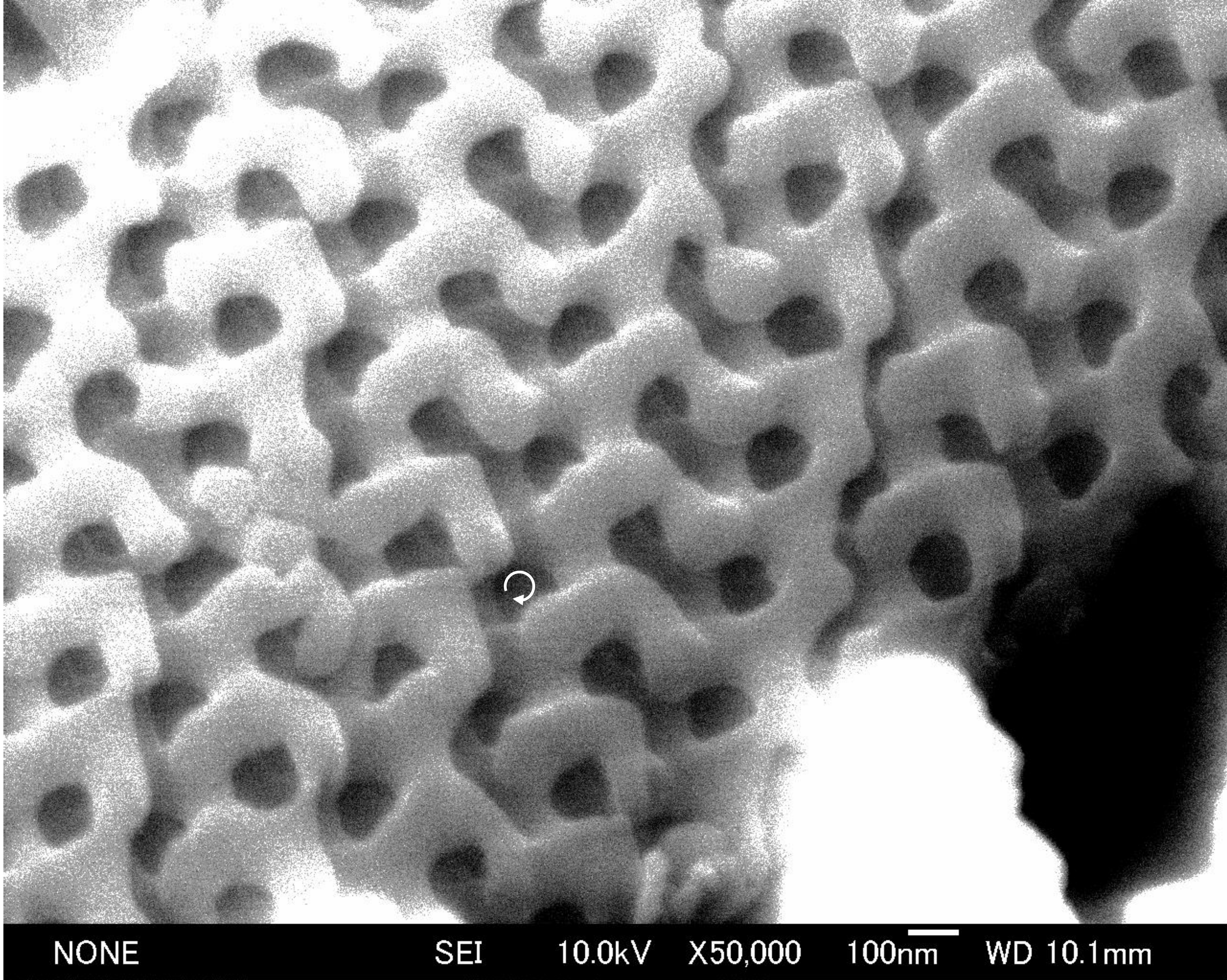

specimen No. 2
scale No. 24
domain No. 2
[111] lh spiral
LH gyroid

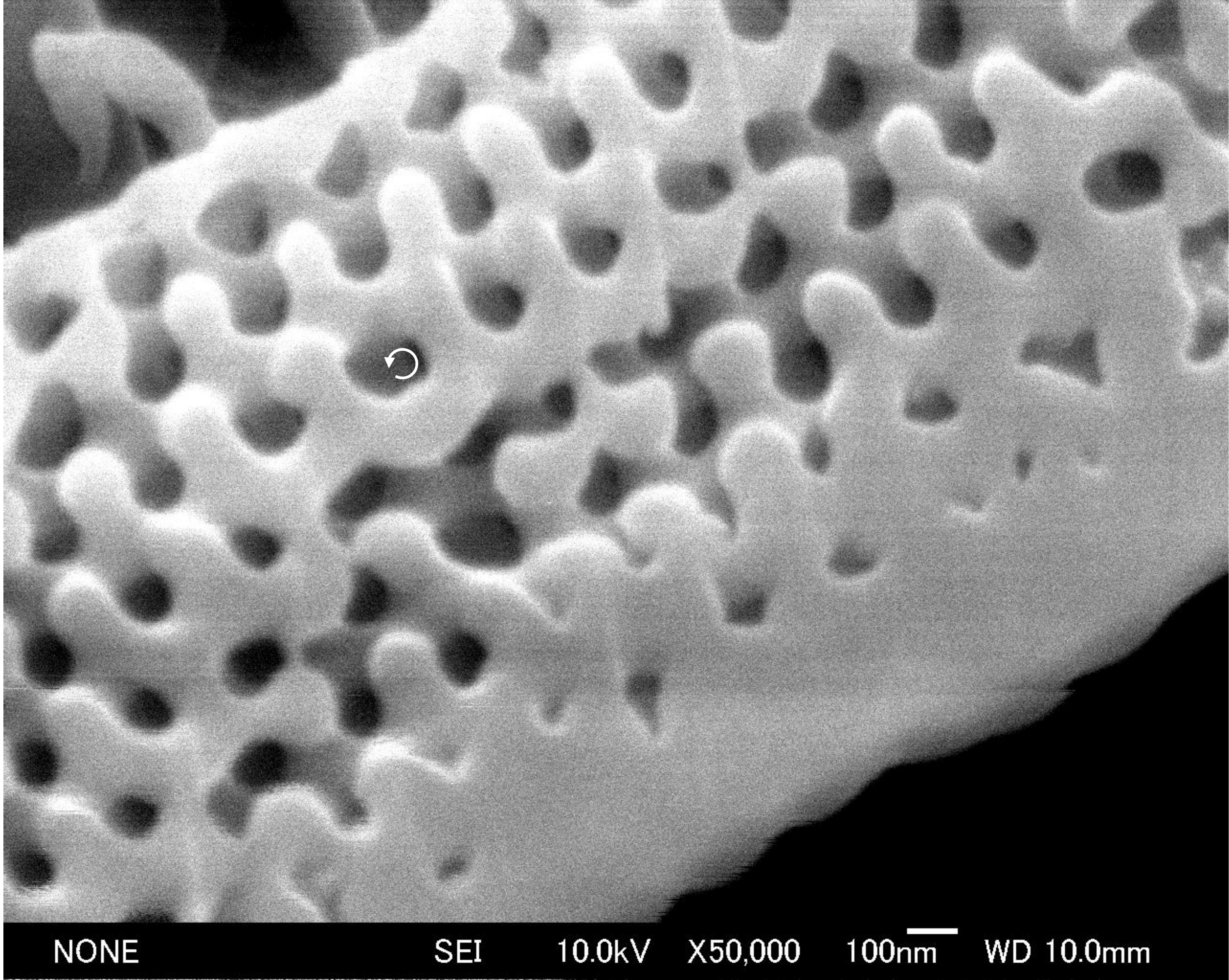

specimen No. 2
scale No. 25

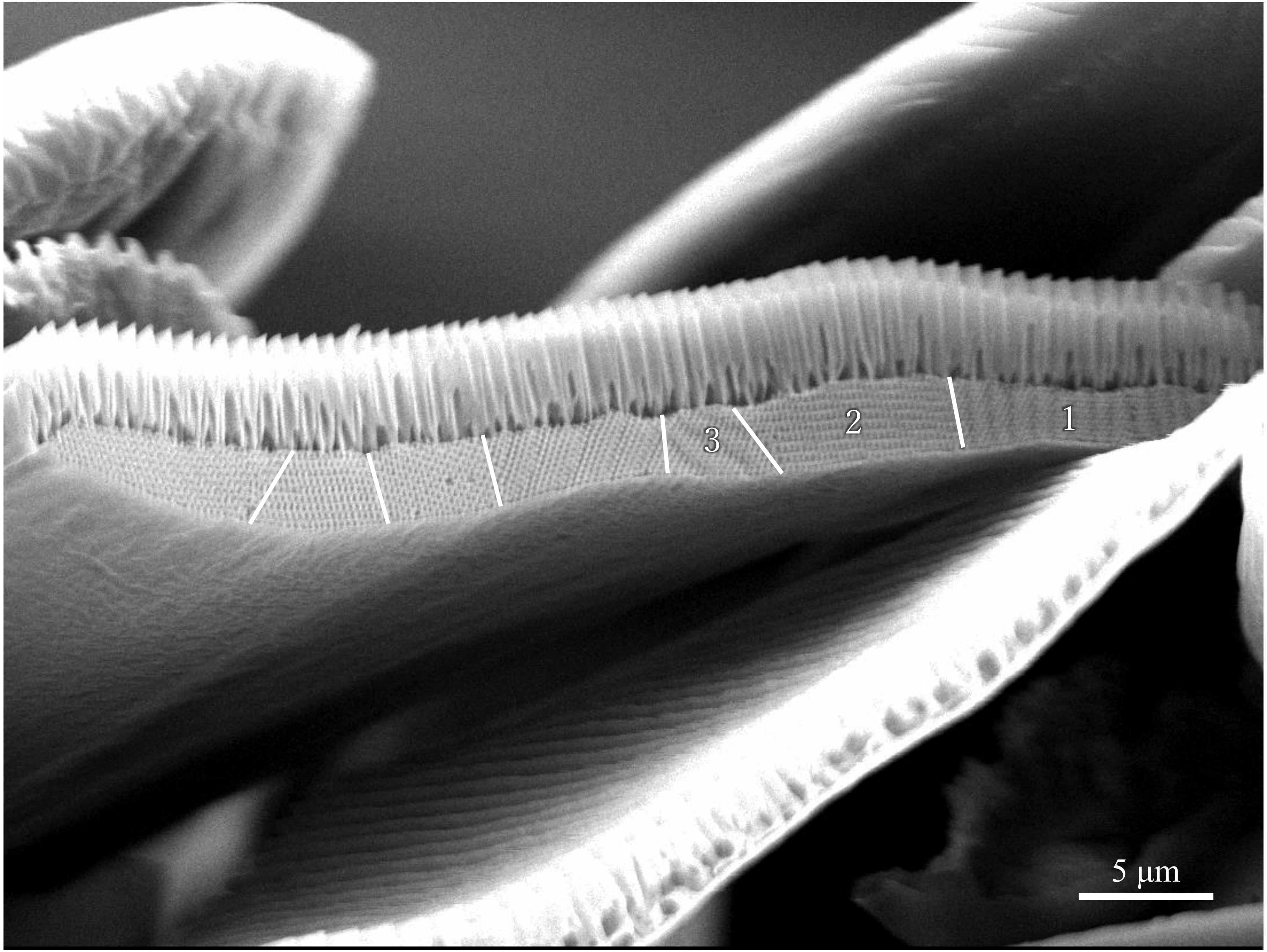

specimen No. 2
scale No. 25
domain No. 1
[111] lh spiral
LH gyroid

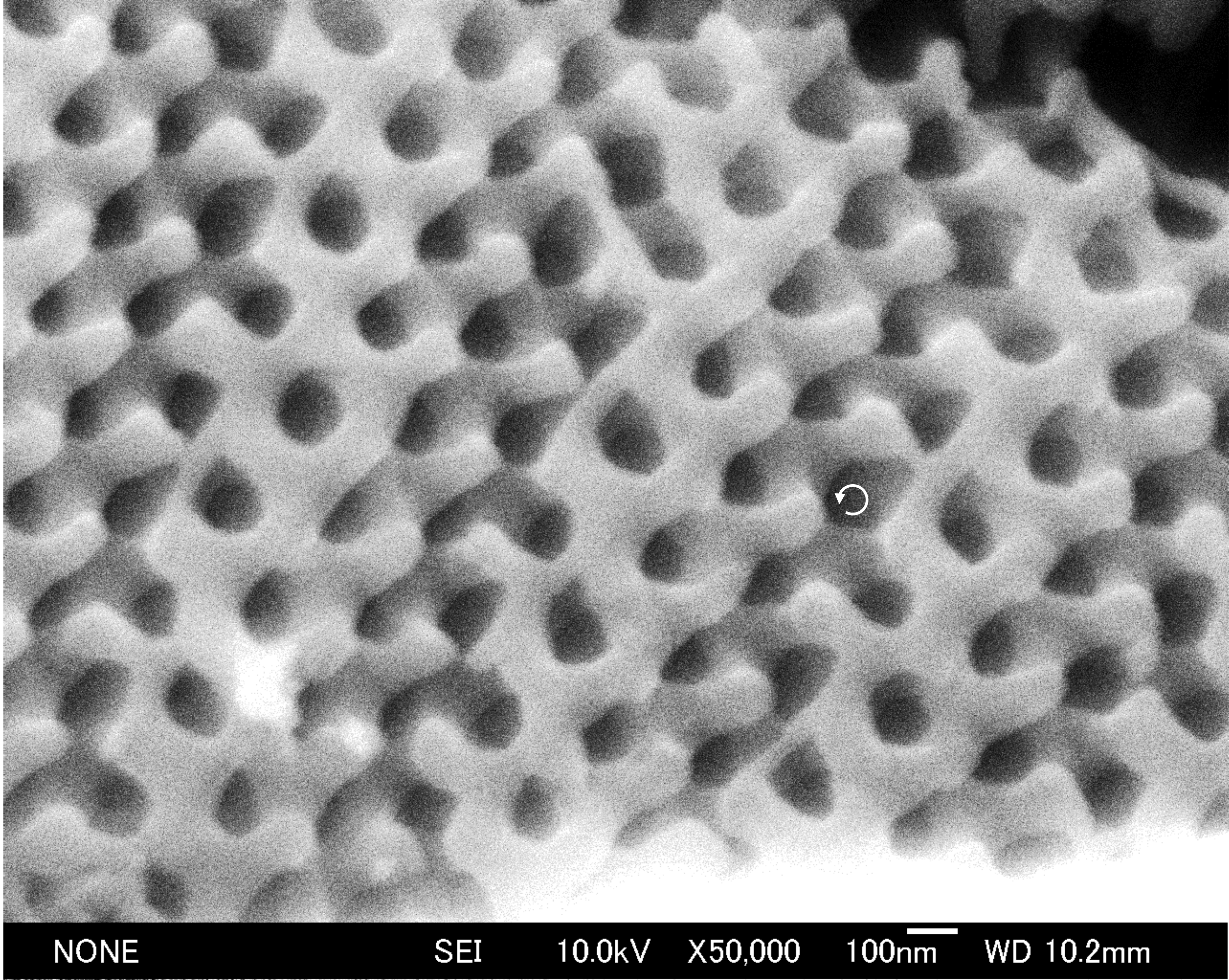

specimen No. 2
scale No. 25
domain No. 2
[100] rh spiral
LH gyroid

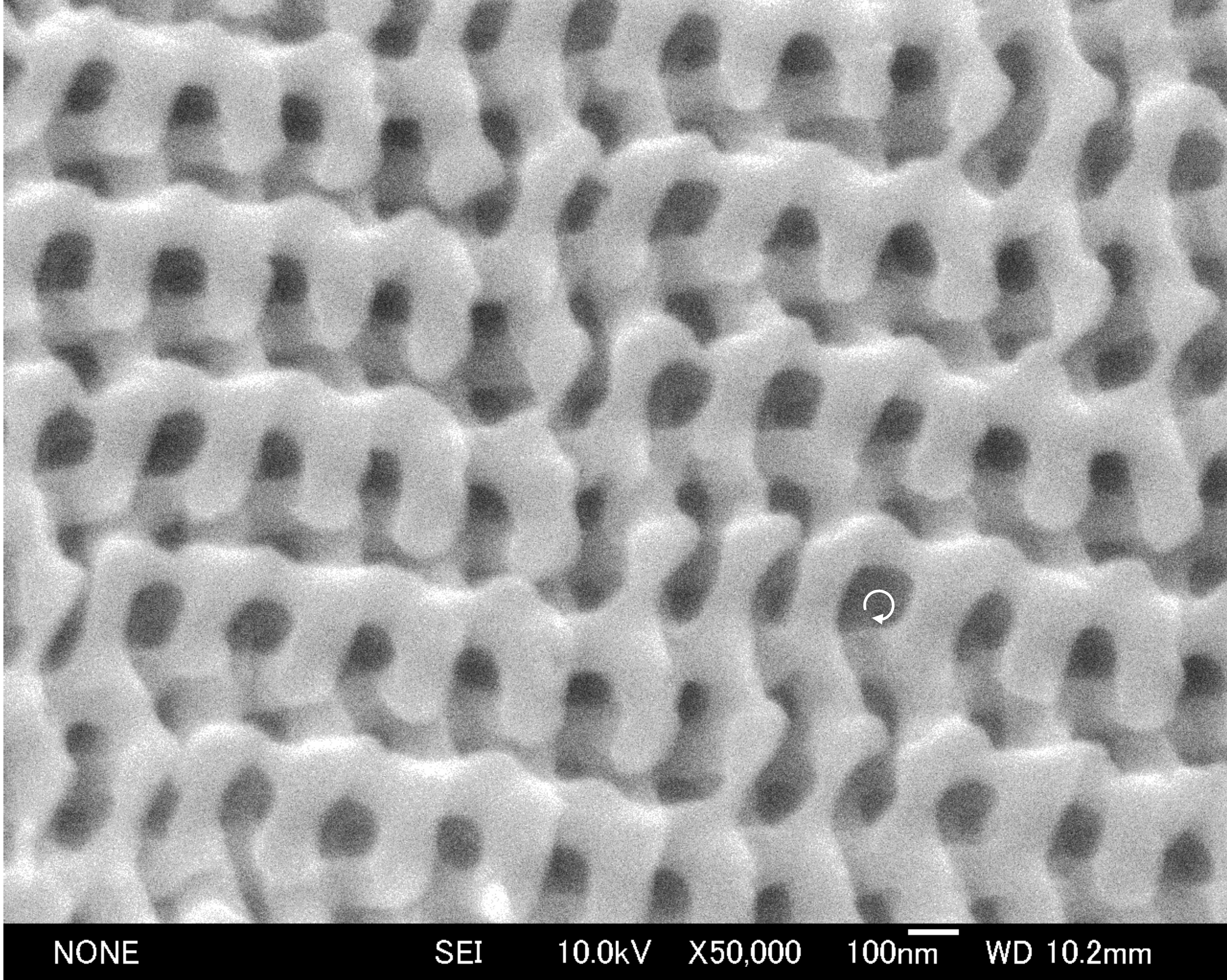

specimen No. 2
scale No. 25
domain No. 3
[111] lh spiral
LH gyroid

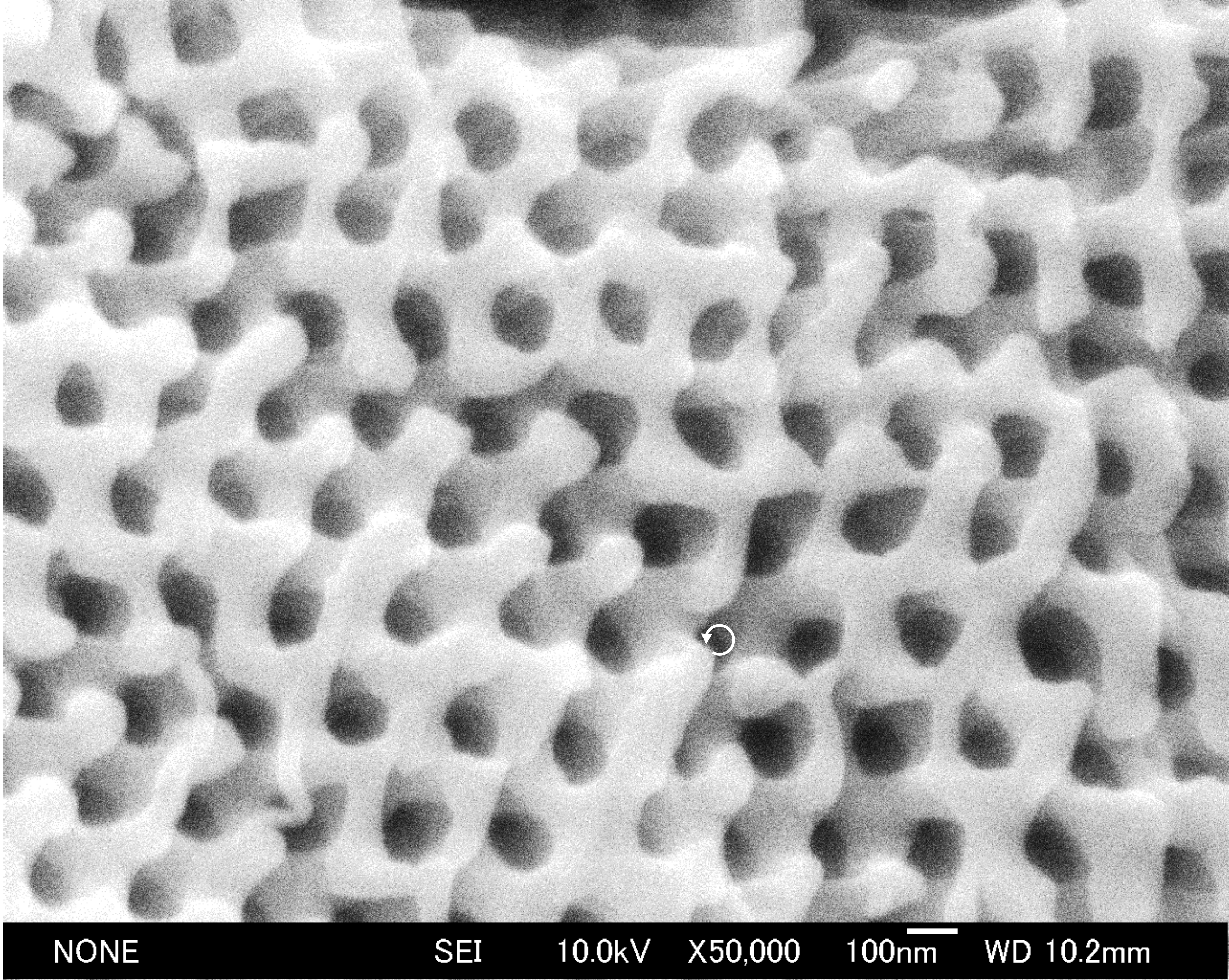

specimen No. 2
scale No. 26

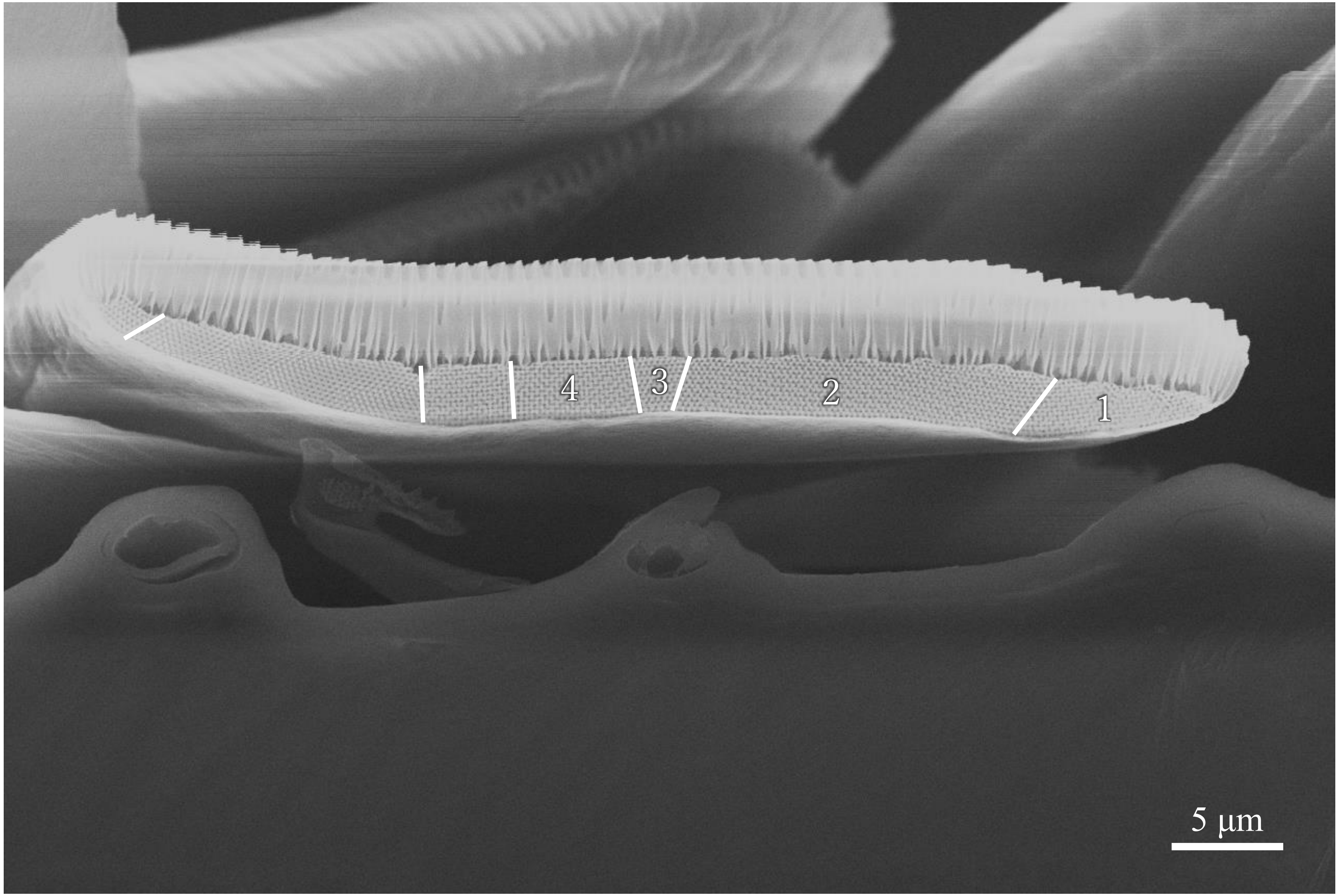

specimen No. 2
scale No. 26
domain No. 1
[100] rh spiral
LH gyroid

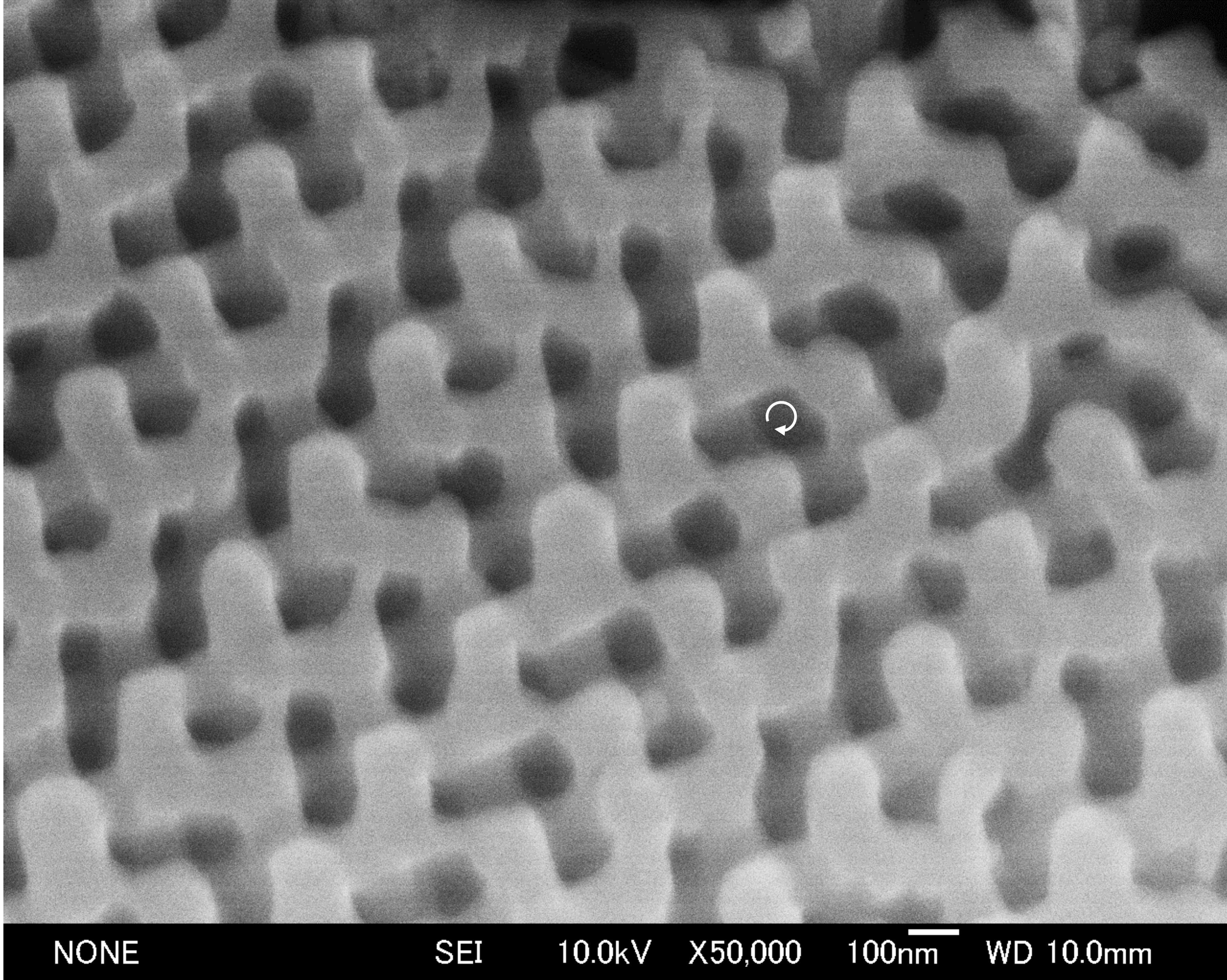

specimen No. 2
scale No. 26
domain No. 2
[111] rh spiral
RH gyroid

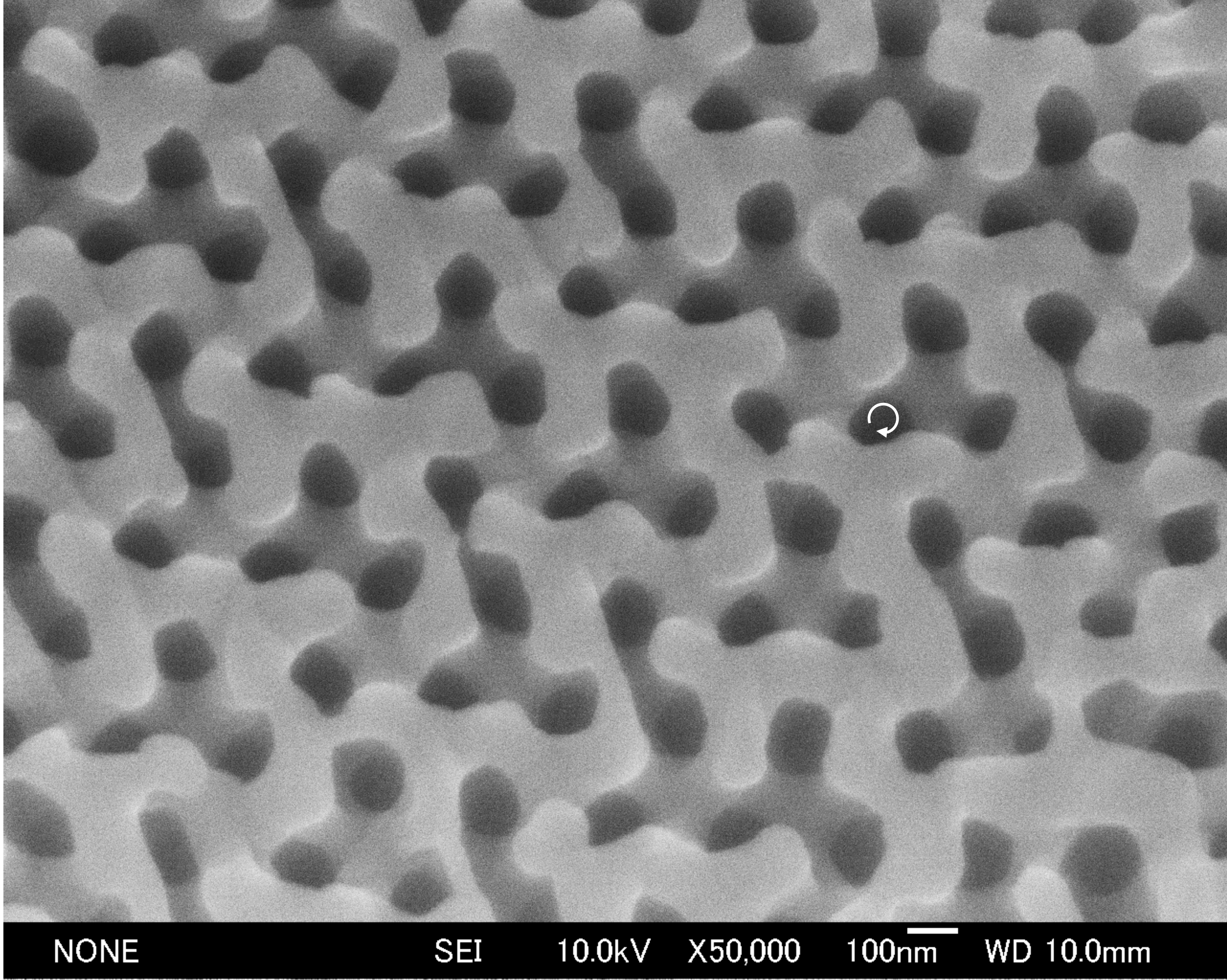

specimen No. 2
scale No. 26
domain No. 3
[111] lh spiral
LH gyroid

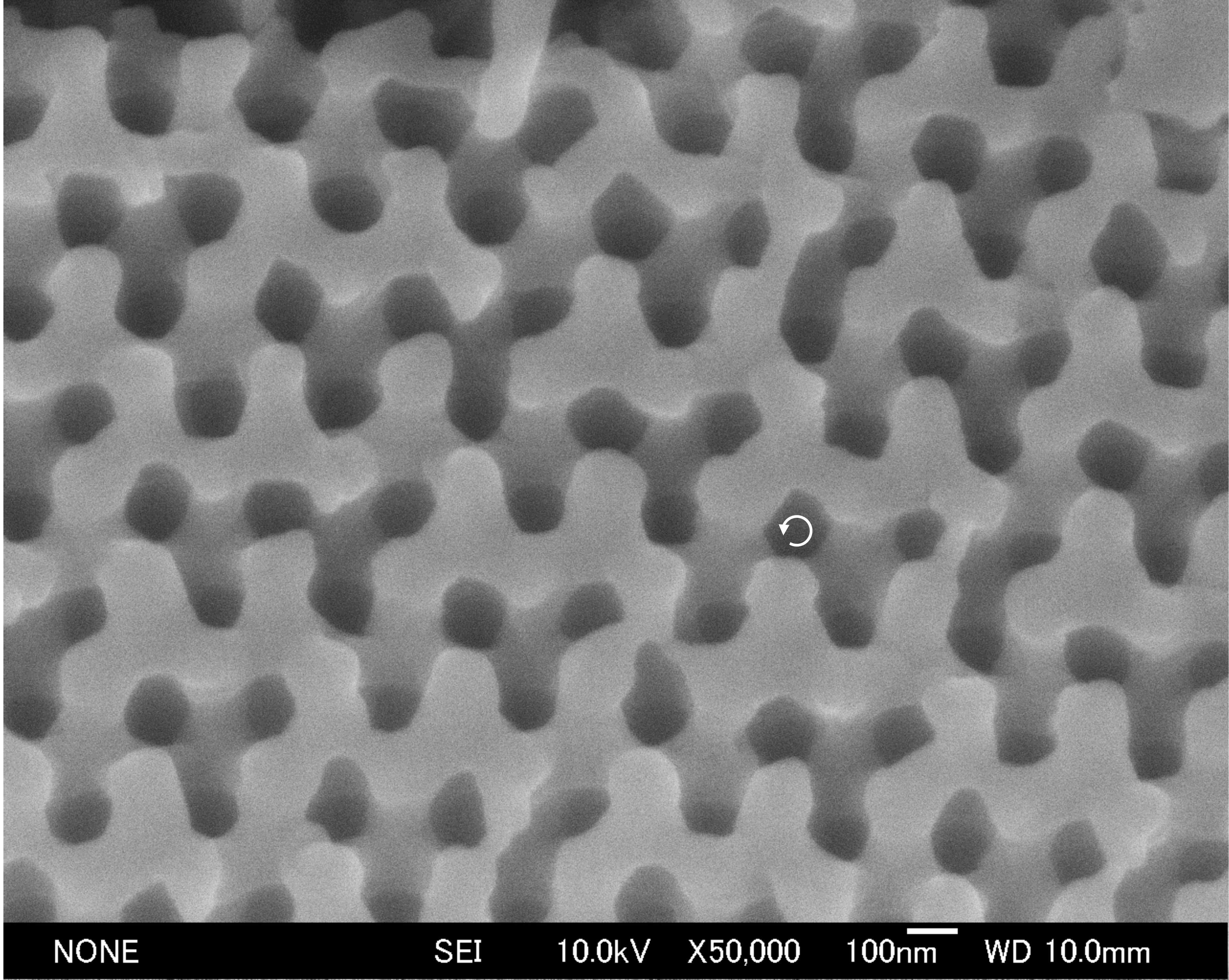

specimen No. 2
scale No. 26
domain No. 4
[111] lh spiral
LH gyroid

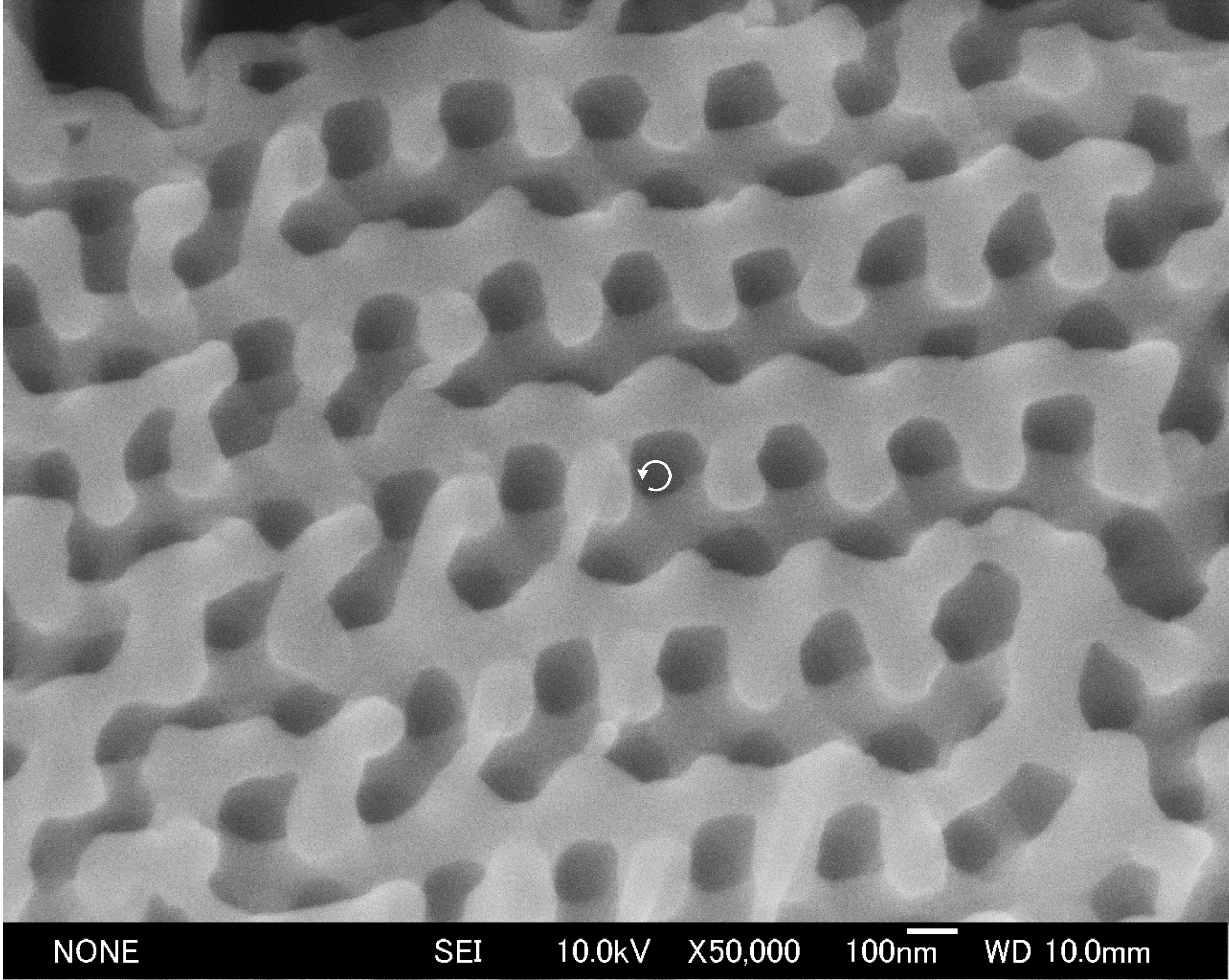

Supplement: Supplementary file 4 — Supplementary Information 4. [file 41598_2025_5750_MOESM4_ESM.pdf]
