# Supplementary material for: Study on the chirality of gyroid photonic crystals in butterfly wing scales
Source: Sci Rep. 2025 Jul 1;15:20968. doi: 10.1038/s41598-025-05750-2 (PMC12215952; doi:10.1038/s41598-025-05750-2)

specimen No. 1
scale No. 1

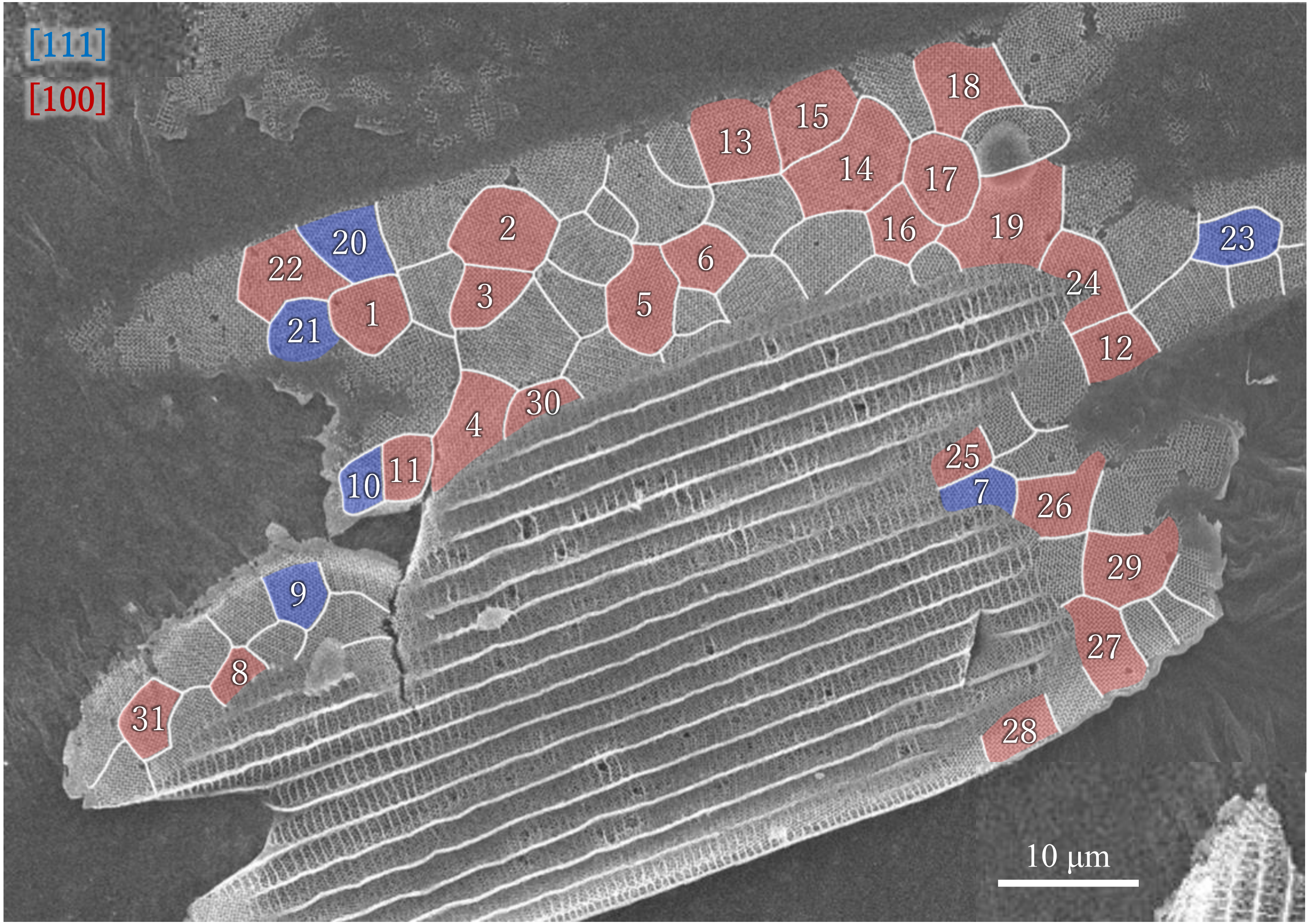

specimen No. 1
scale No. 1
domain No. 1
[100] rh spiral
LH gyroid

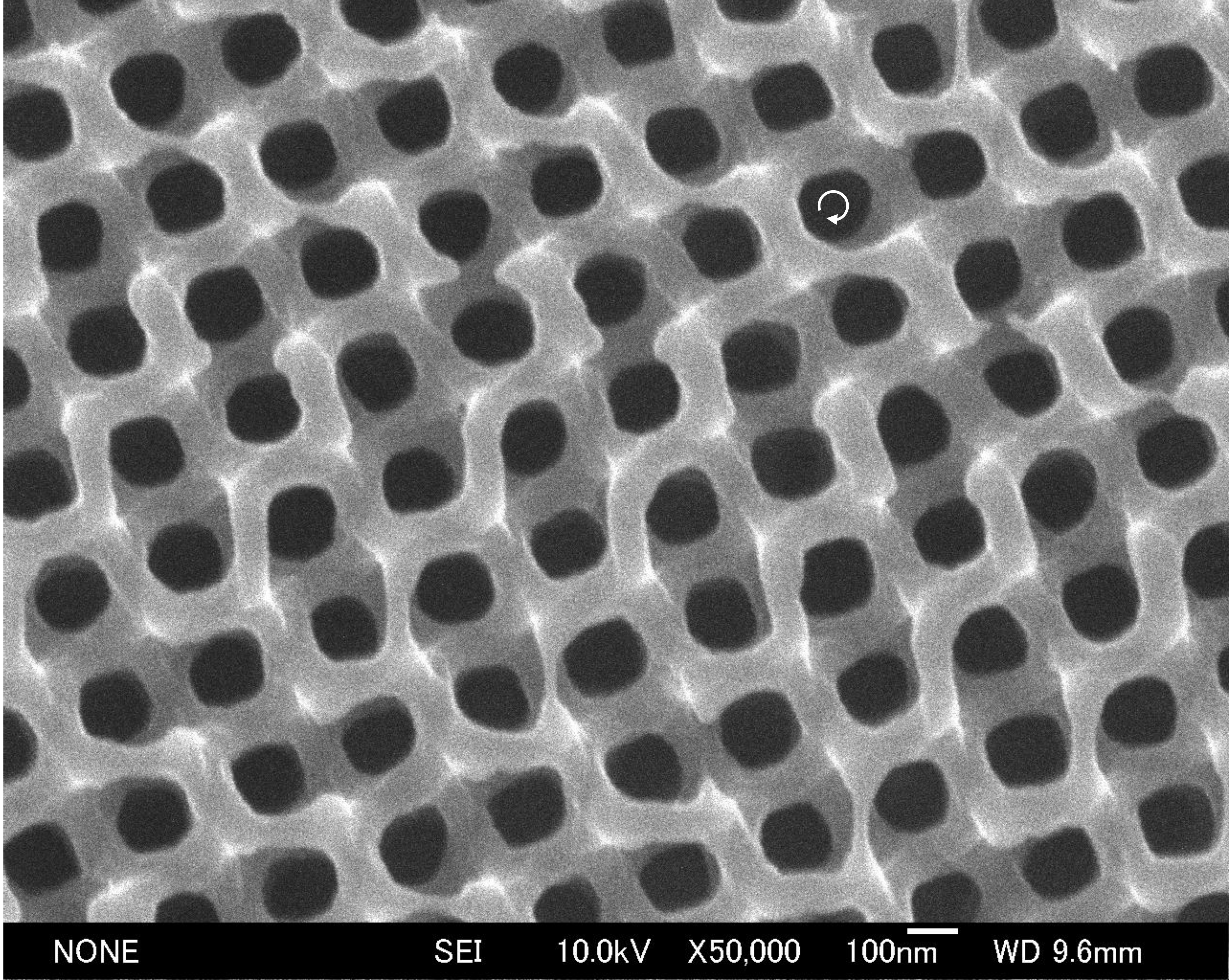

specimen No. 1
scale No. 1
domain No. 2
[100] lh spiral
RH gyroid

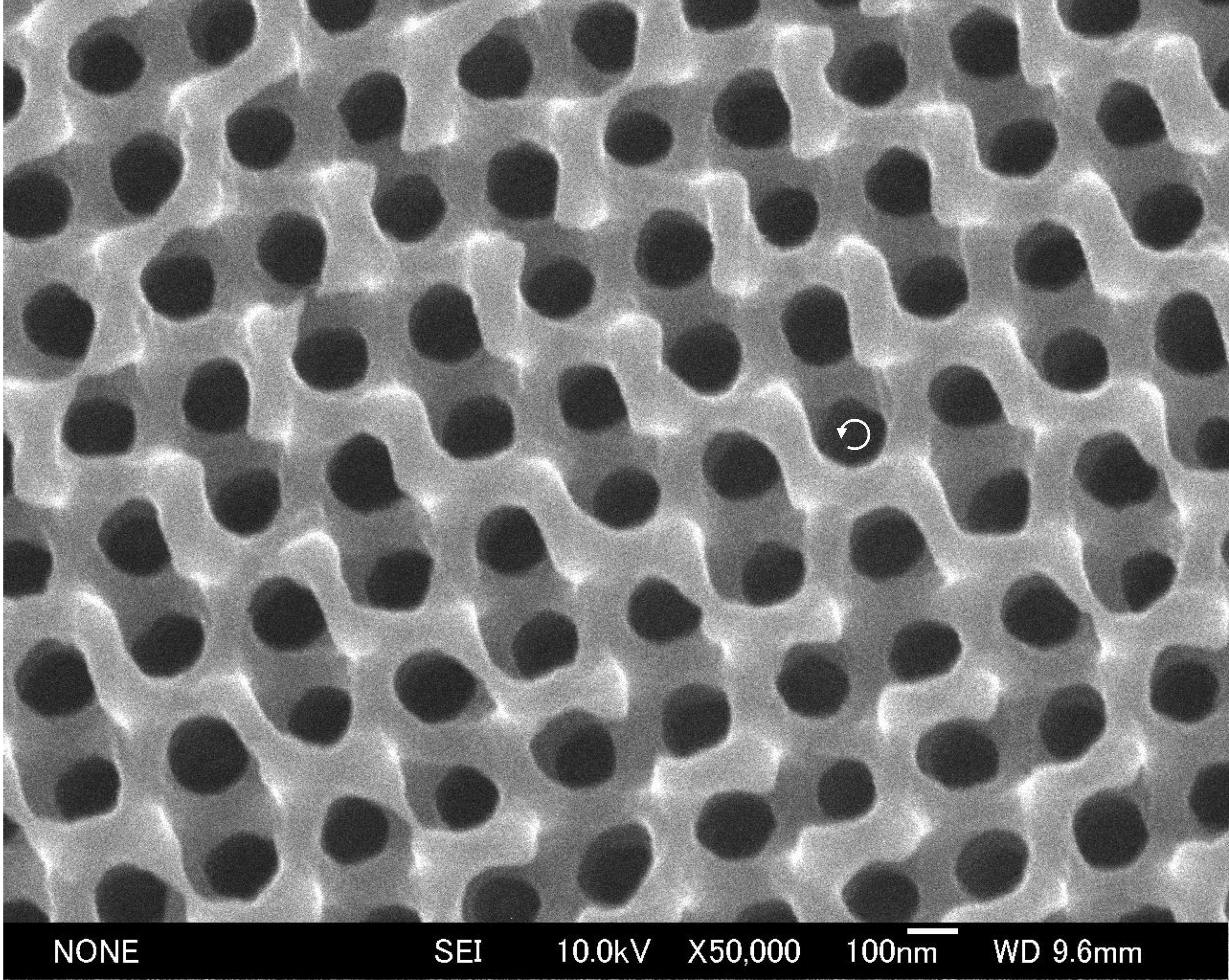

specimen No. 1
scale No. 1
domain No. 3
[100] rh spiral
LH gyroid

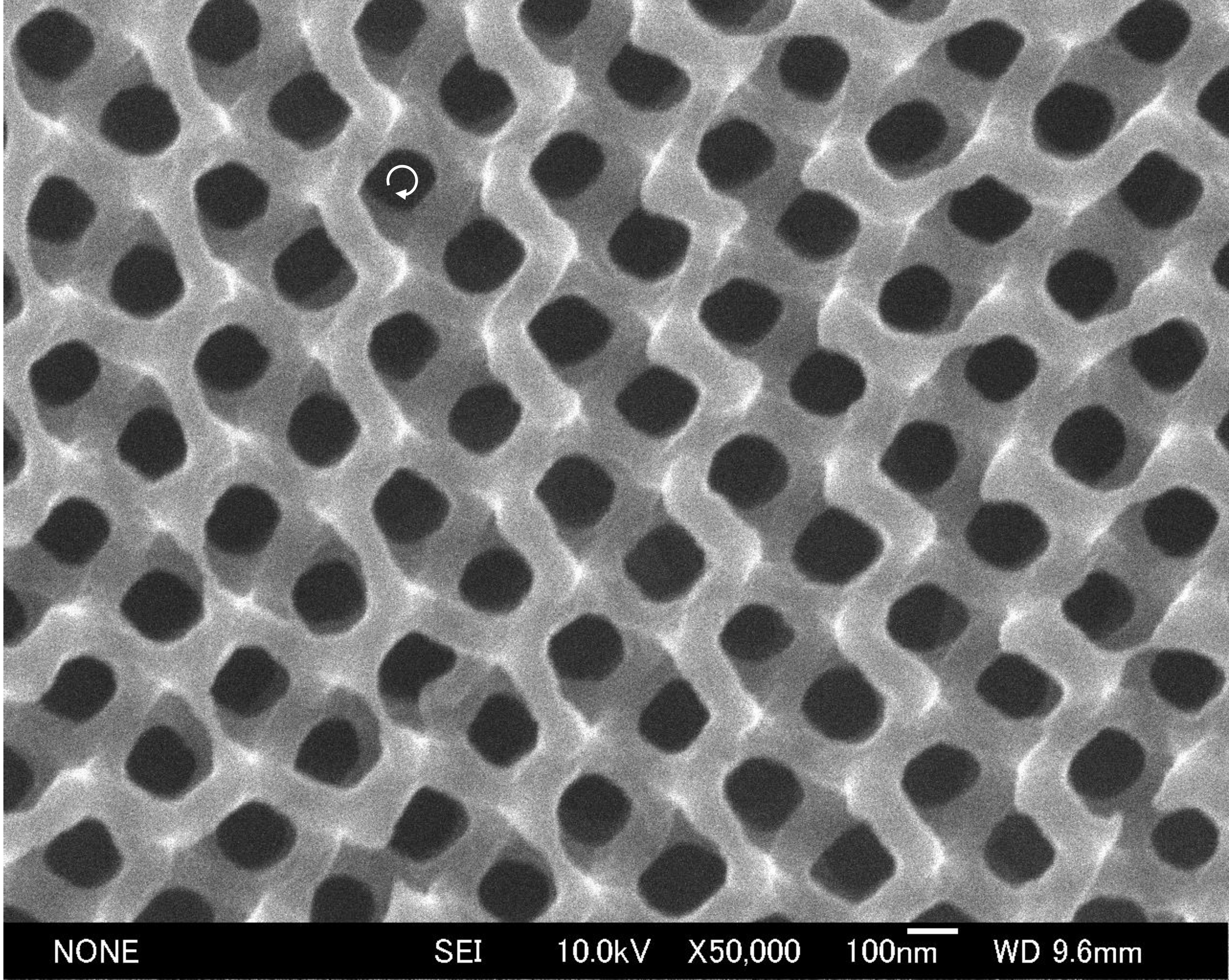

specimen No. 1
scale No. 1
domain No. 4
[100] rh spiral
LH gyroid

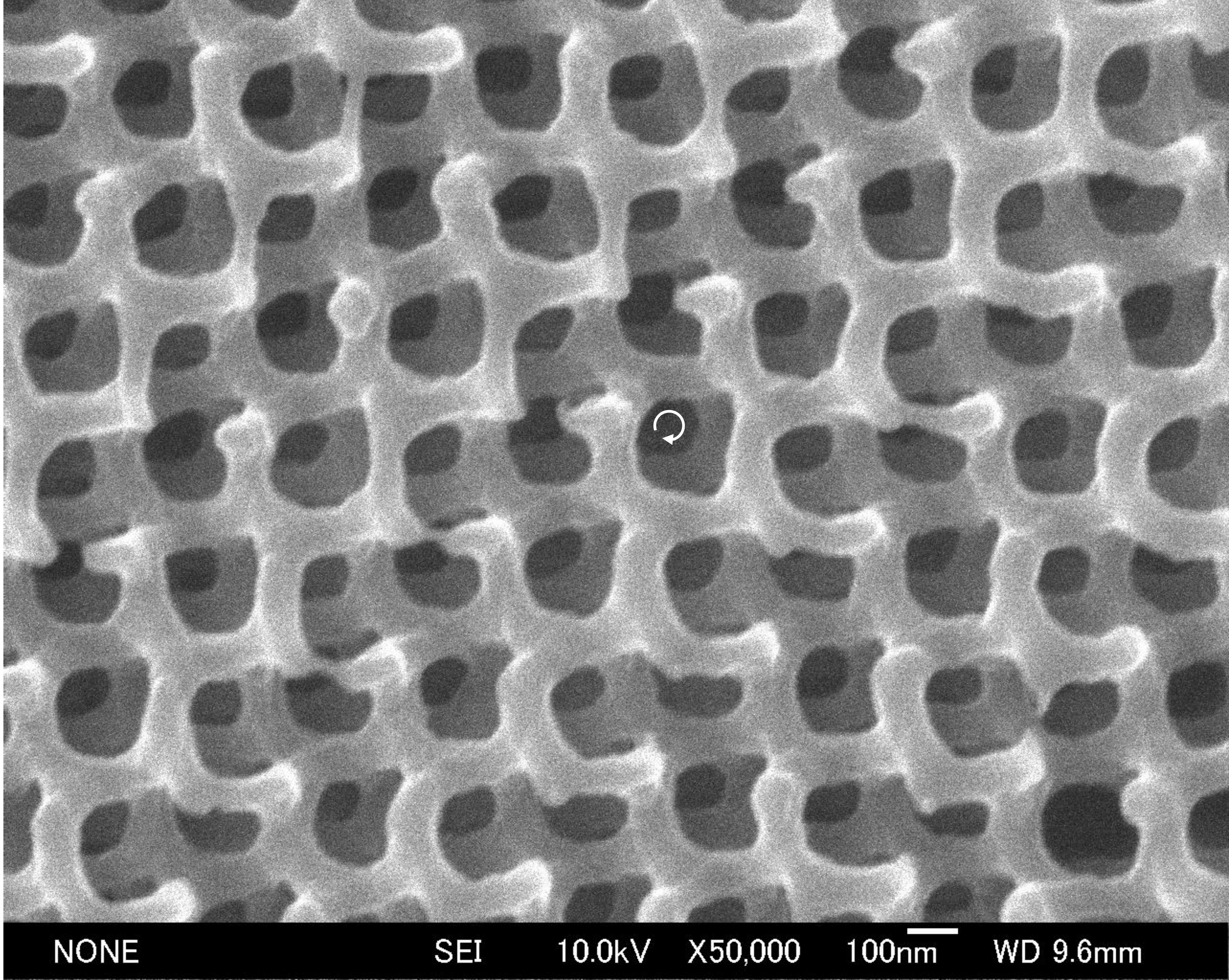

specimen No. 1
scale No. 1
domain No. 5
[100] rh spiral
LH gyroid

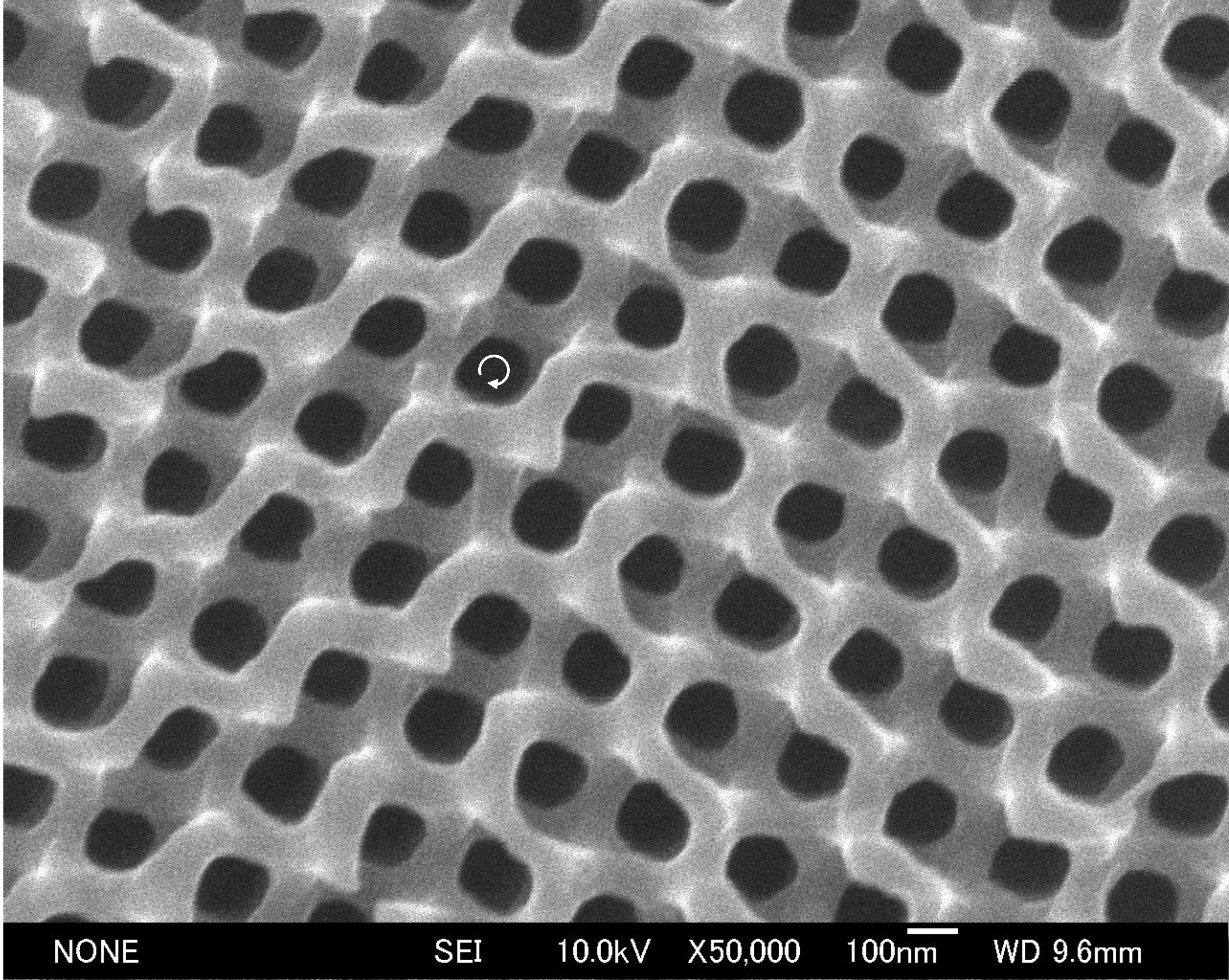

specimen No. 1
scale No. 1
domain No. 6
[100] lh spiral
RH gyroid

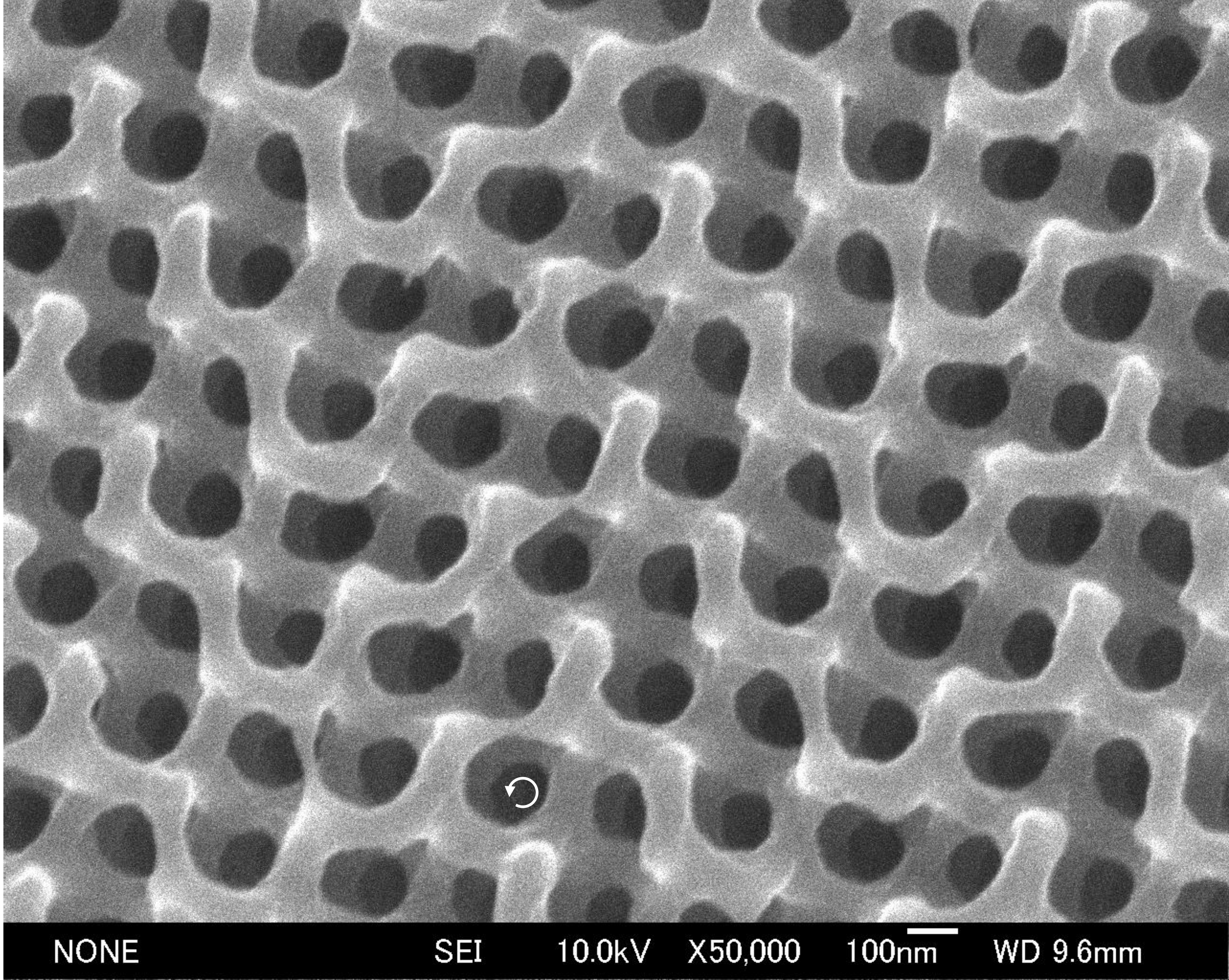

specimen No. 1
scale No. 1
domain No. 7
[111] lh spiral
LH gyroid

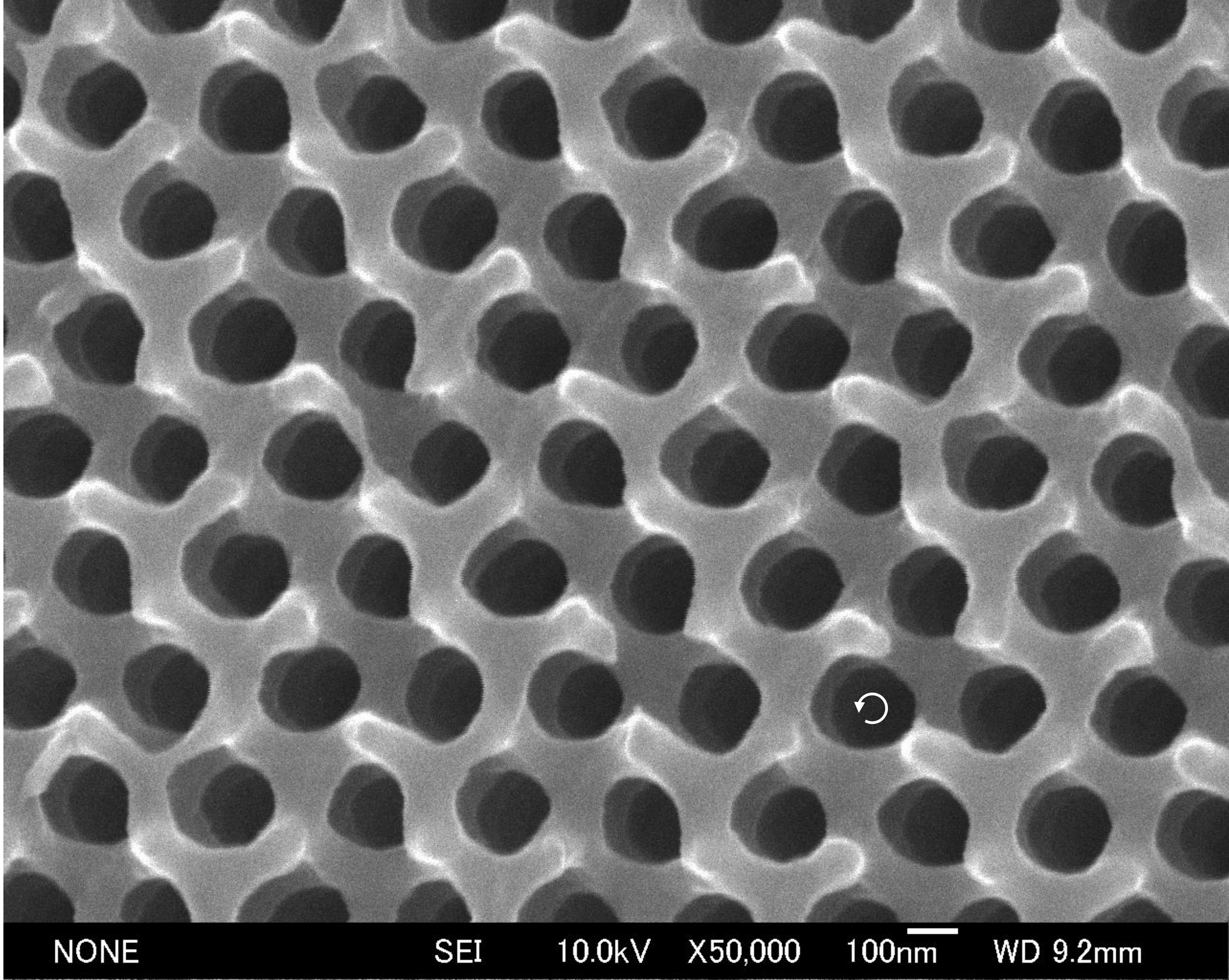

specimen No. 1
scale No. 1
domain No. 8
[100] rh spiral
LH gyroid

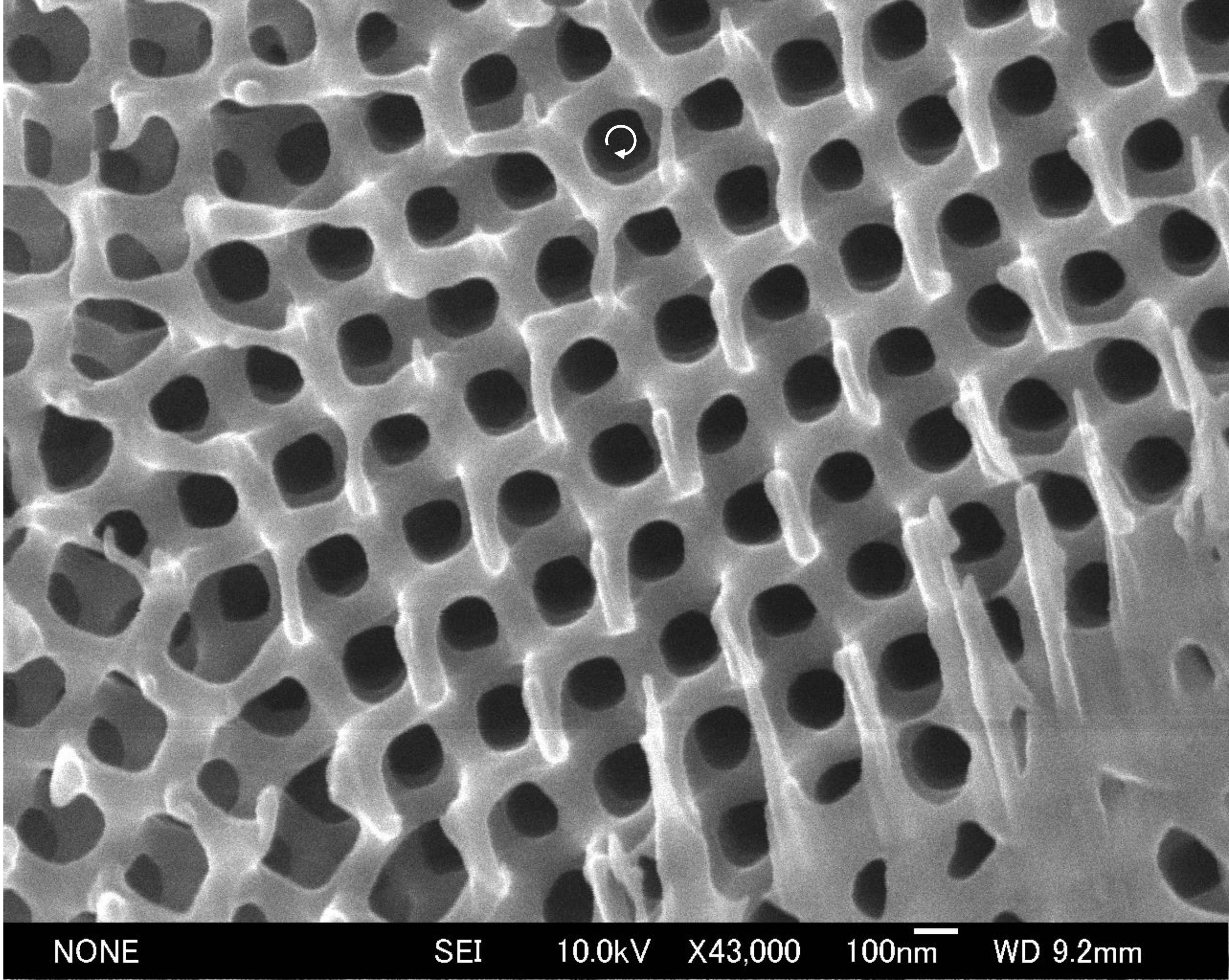

specimen No. 1
scale No. 1
domain No. 9
[111] rh spiral
RH gyroid

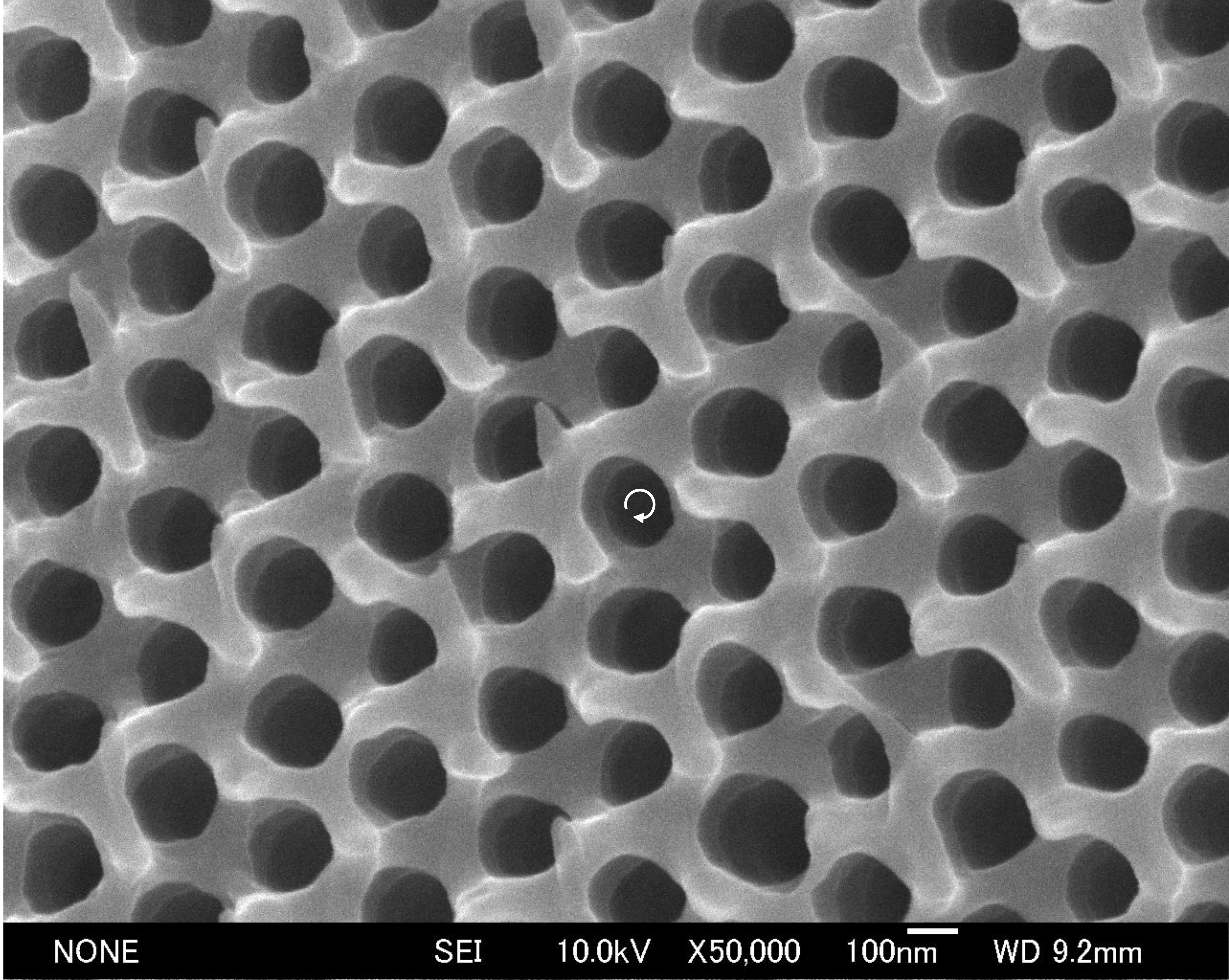

specimen No. 1
scale No. 1
domain No. 10
[111] lh spiral
LH gyroid

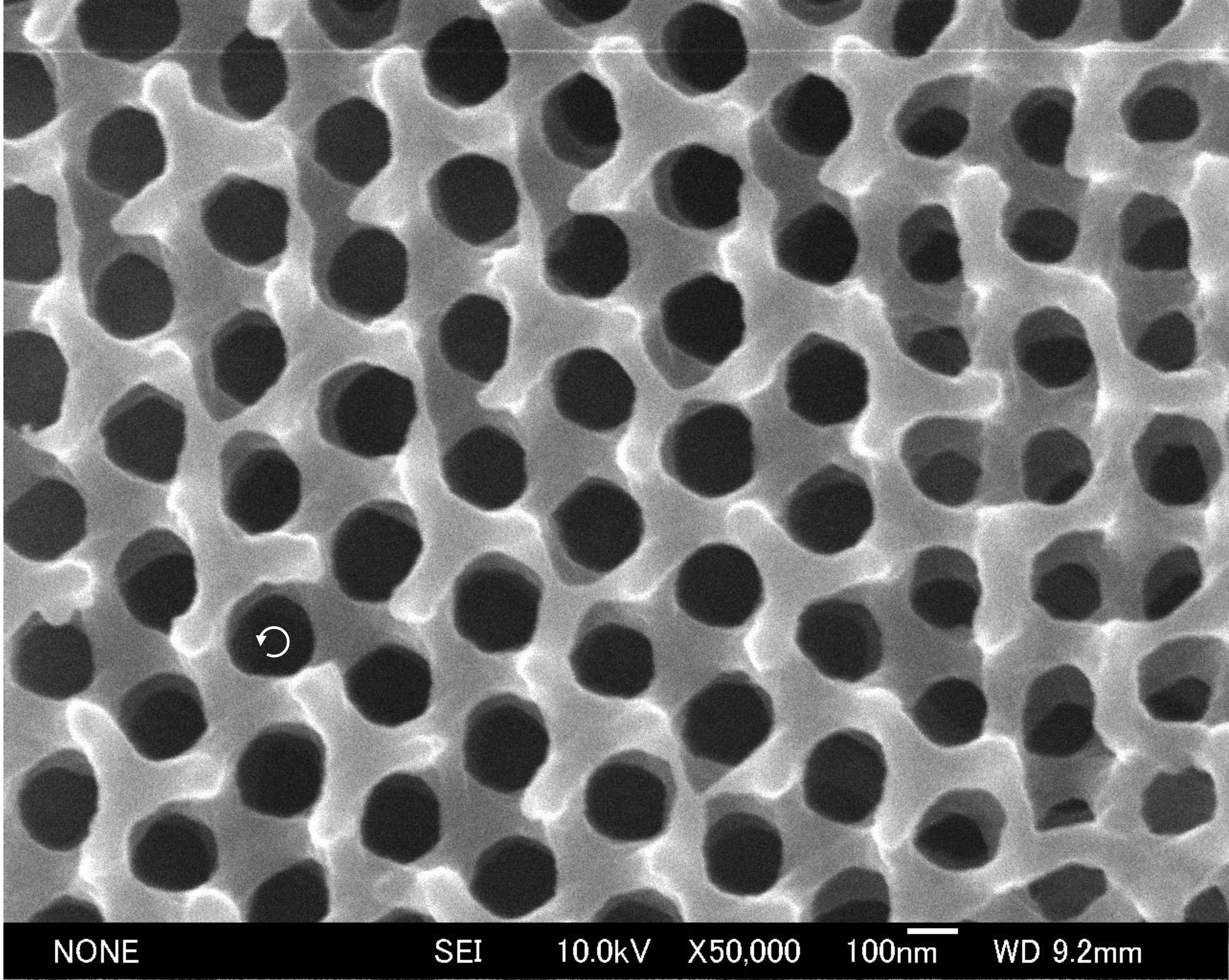

specimen No. 1
scale No. 1
domain No. 11
[100] lh spiral
RH gyroid

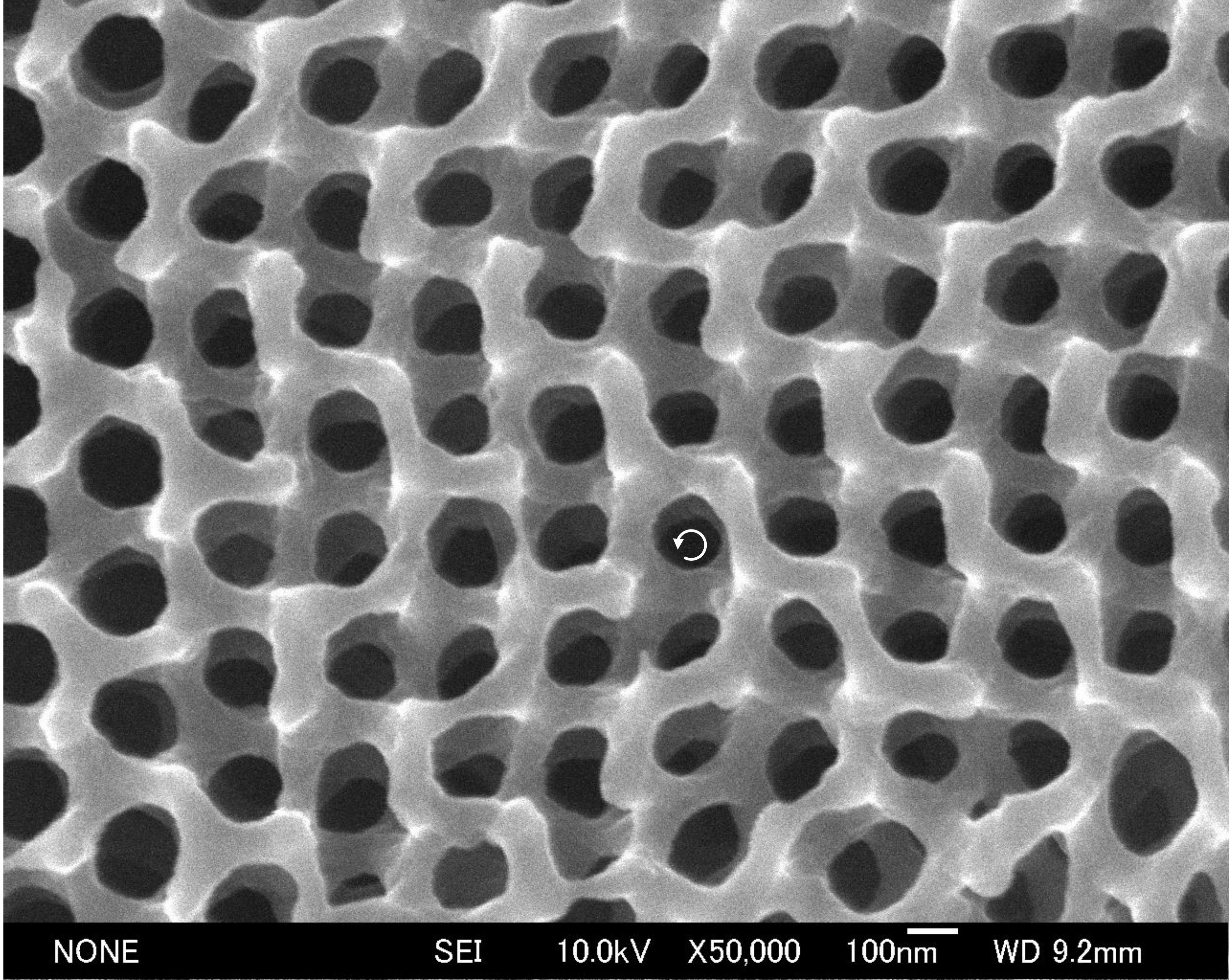

specimen No. 1
scale No. 1
domain No. 12
[100] rh spiral
LH gyroid

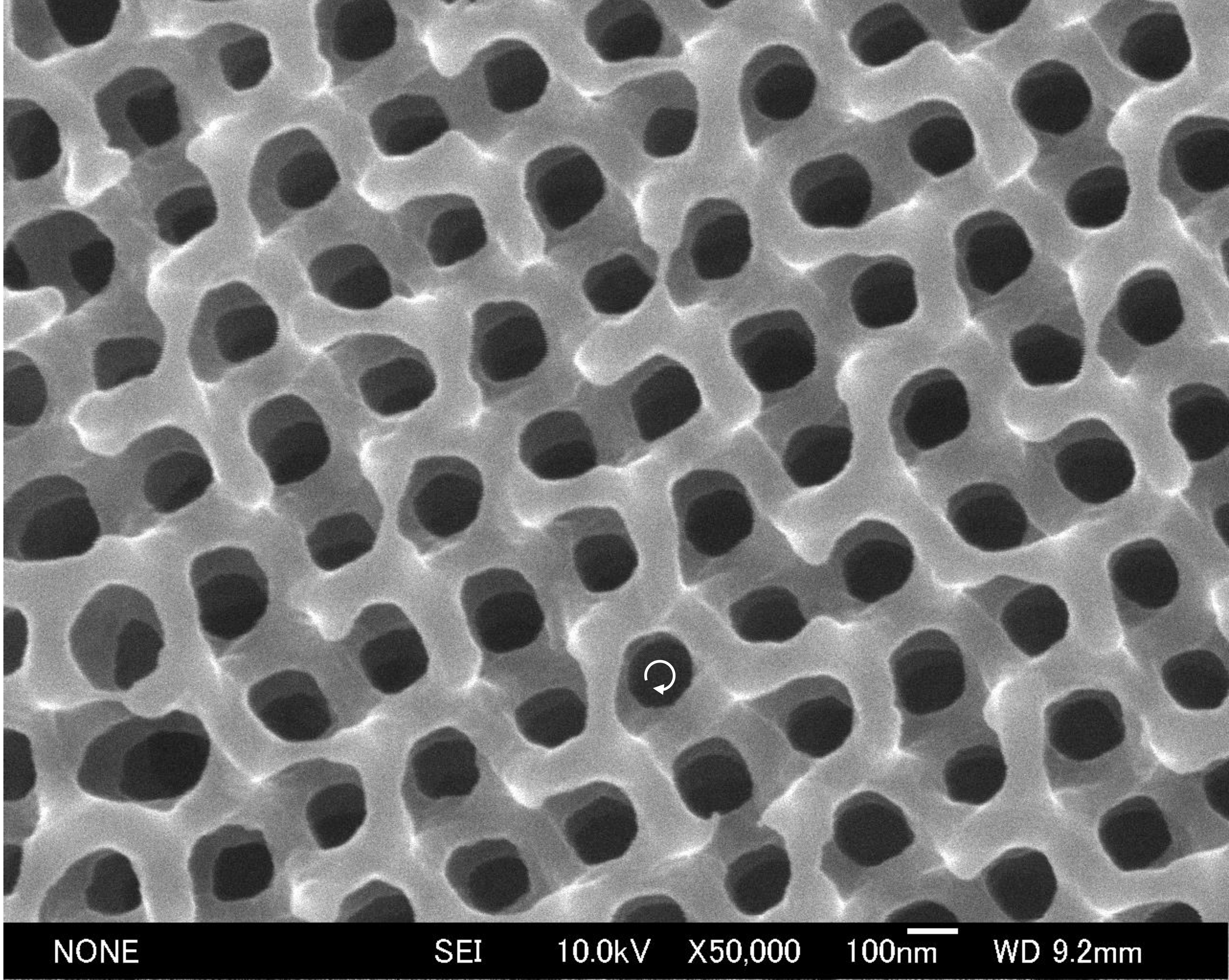

specimen No. 1
scale No. 1
domain No. 13
[100] rh spiral
LH gyroid

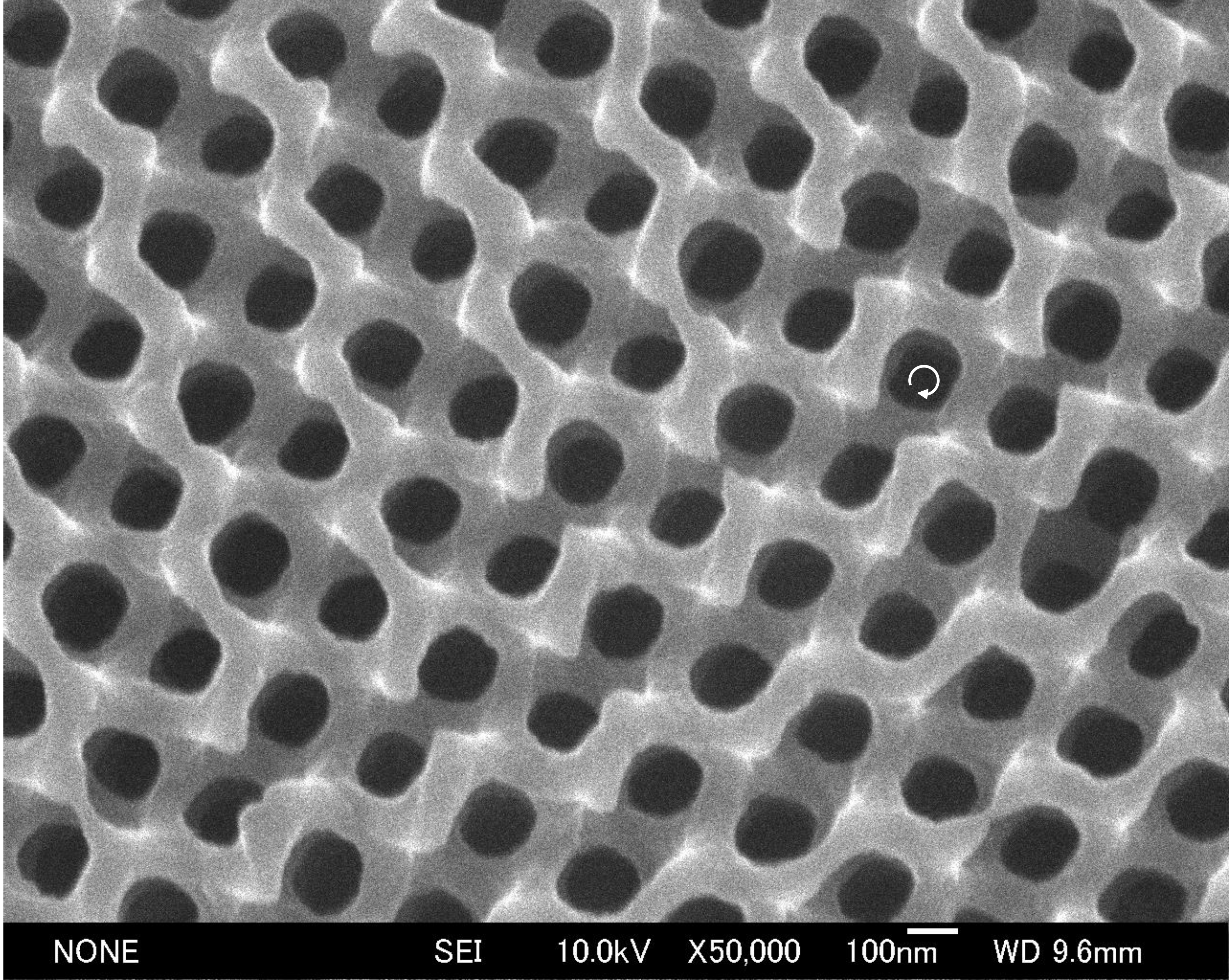

specimen No. 1
scale No. 1
domain No. 14
[100] rh spiral
LH gyroid

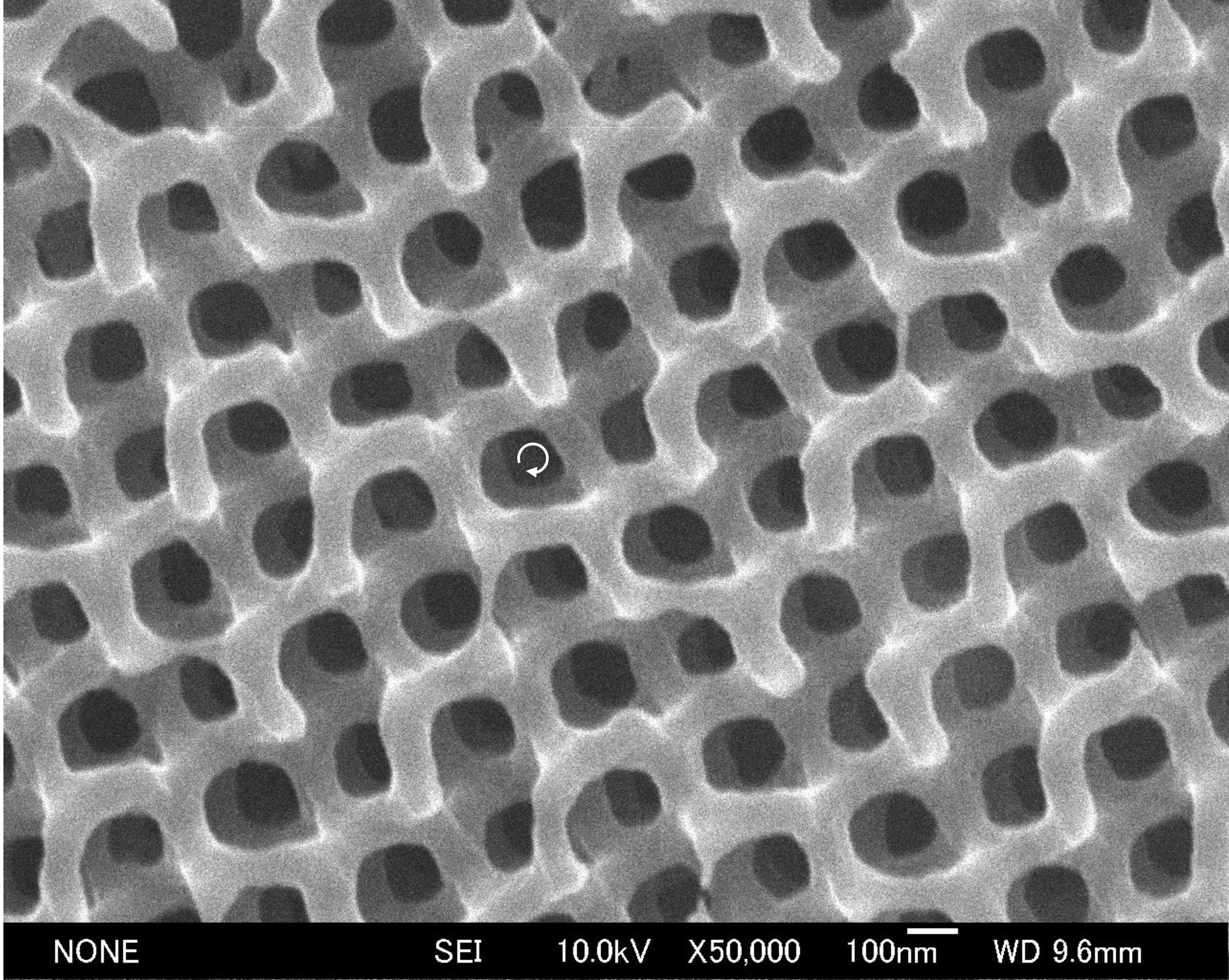

specimen No. 1
scale No. 1
domain No. 15
[100] rh spiral
LH gyroid

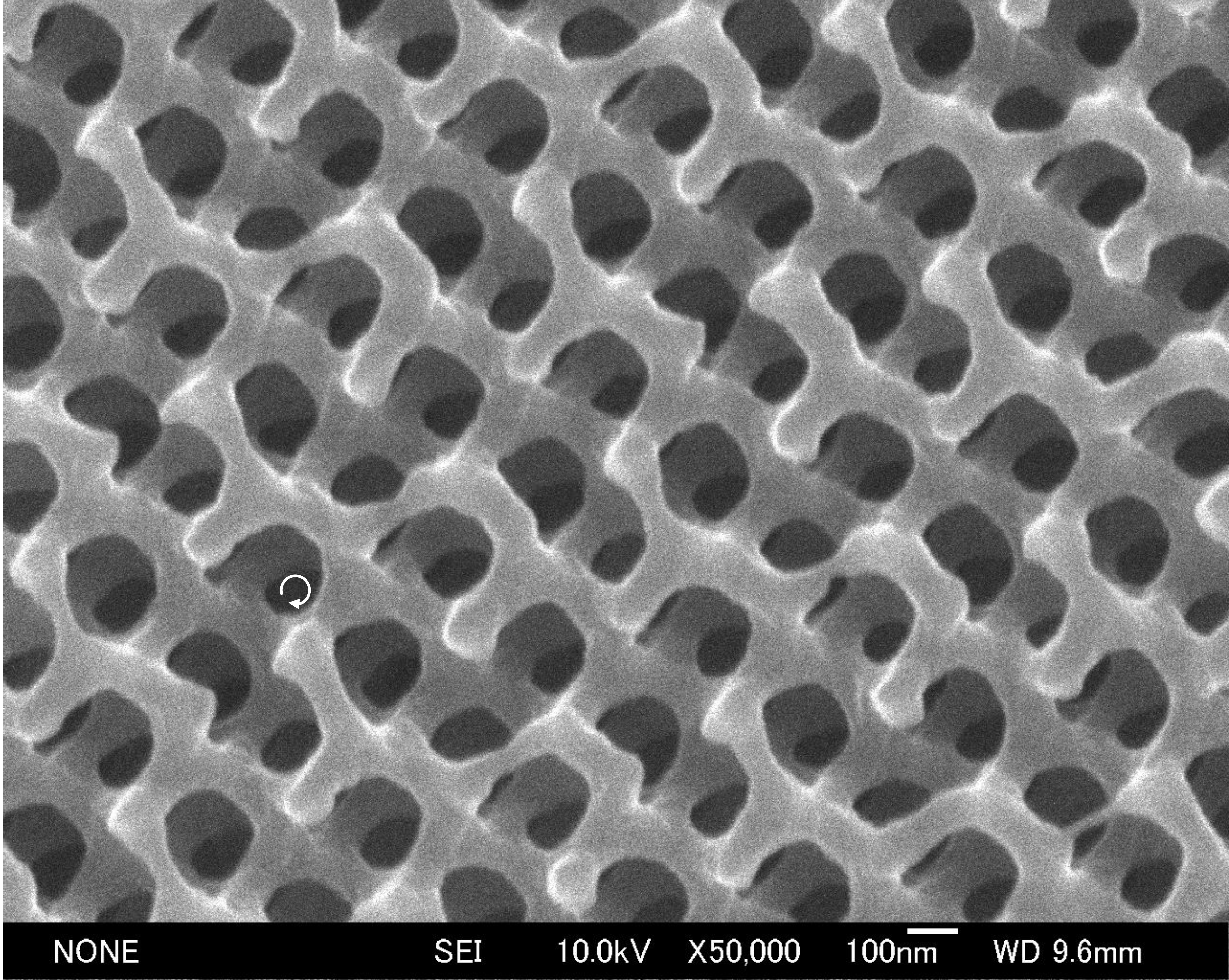

specimen No. 1
scale No. 1
domain No. 16
[100] lh spiral
RH gyroid

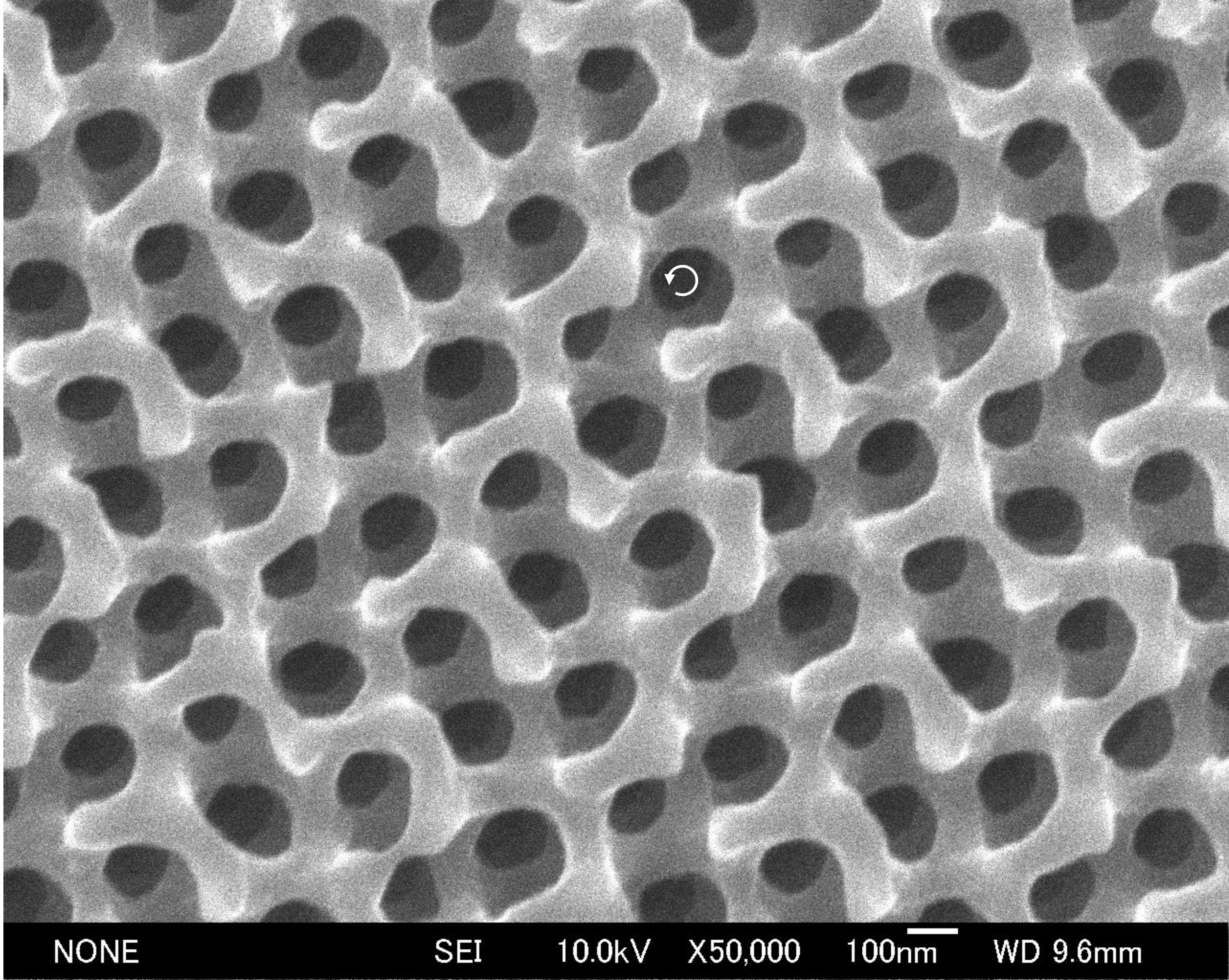

specimen No. 1
scale No. 1
domain No. 17
[100] rh spiral
LH gyroid

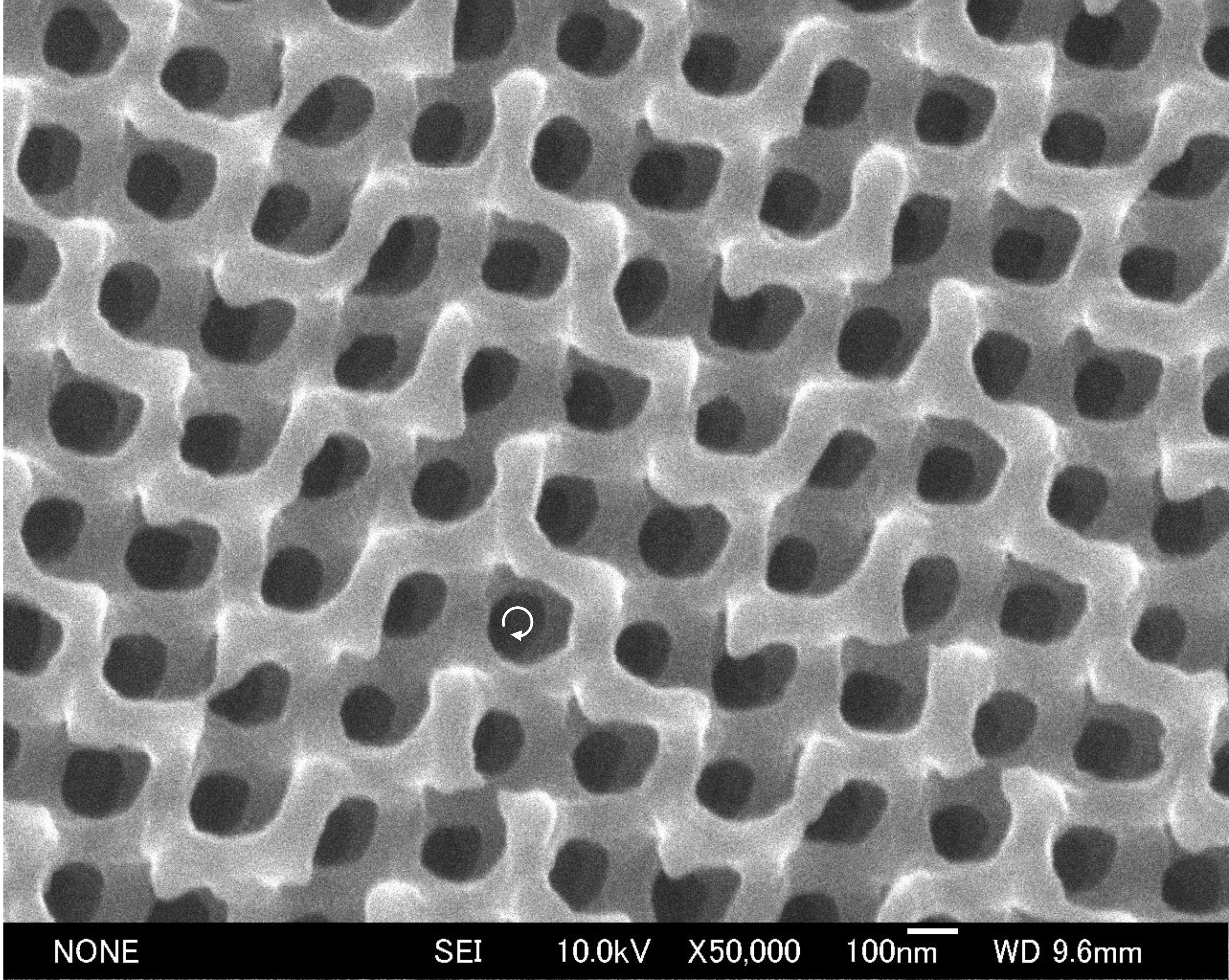

specimen No. 1
scale No. 1
domain No. 18
[100] lh spiral
RH gyroid

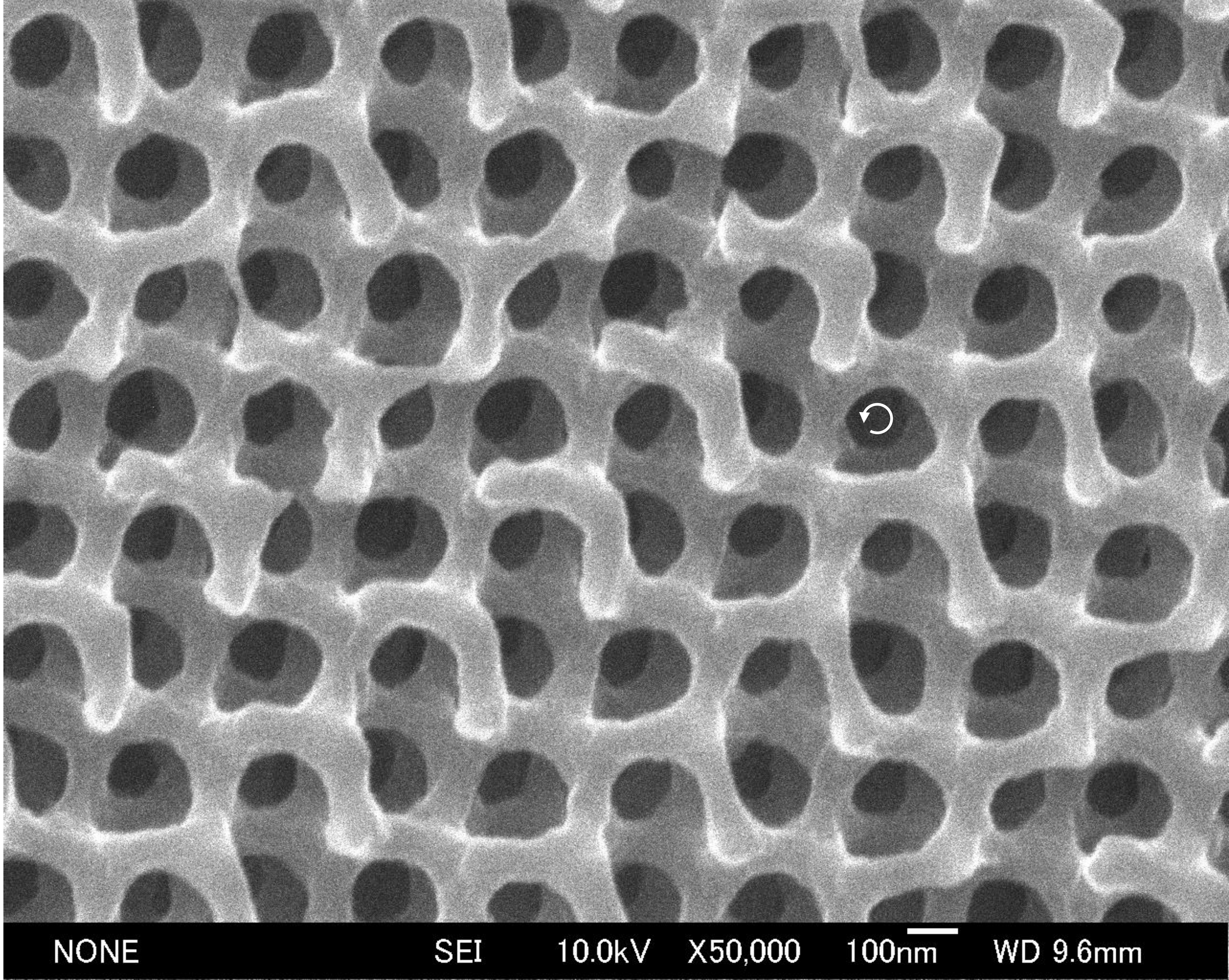

specimen No. 1
scale No. 1
domain No. 19
[100] lh spiral
RH gyroid

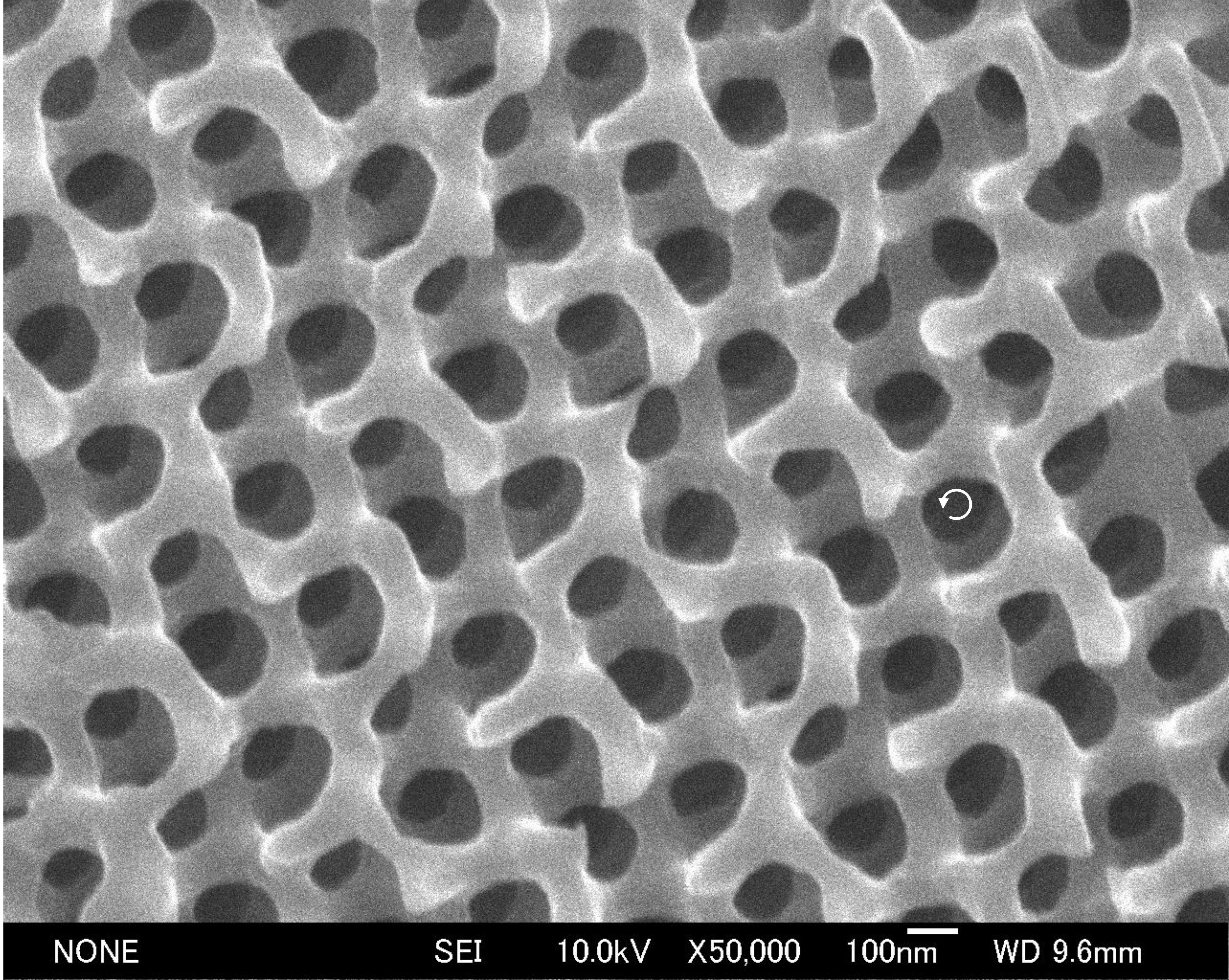

specimen No. 1
scale No. 1
domain No. 20
[111] lh spiral
LH gyroid

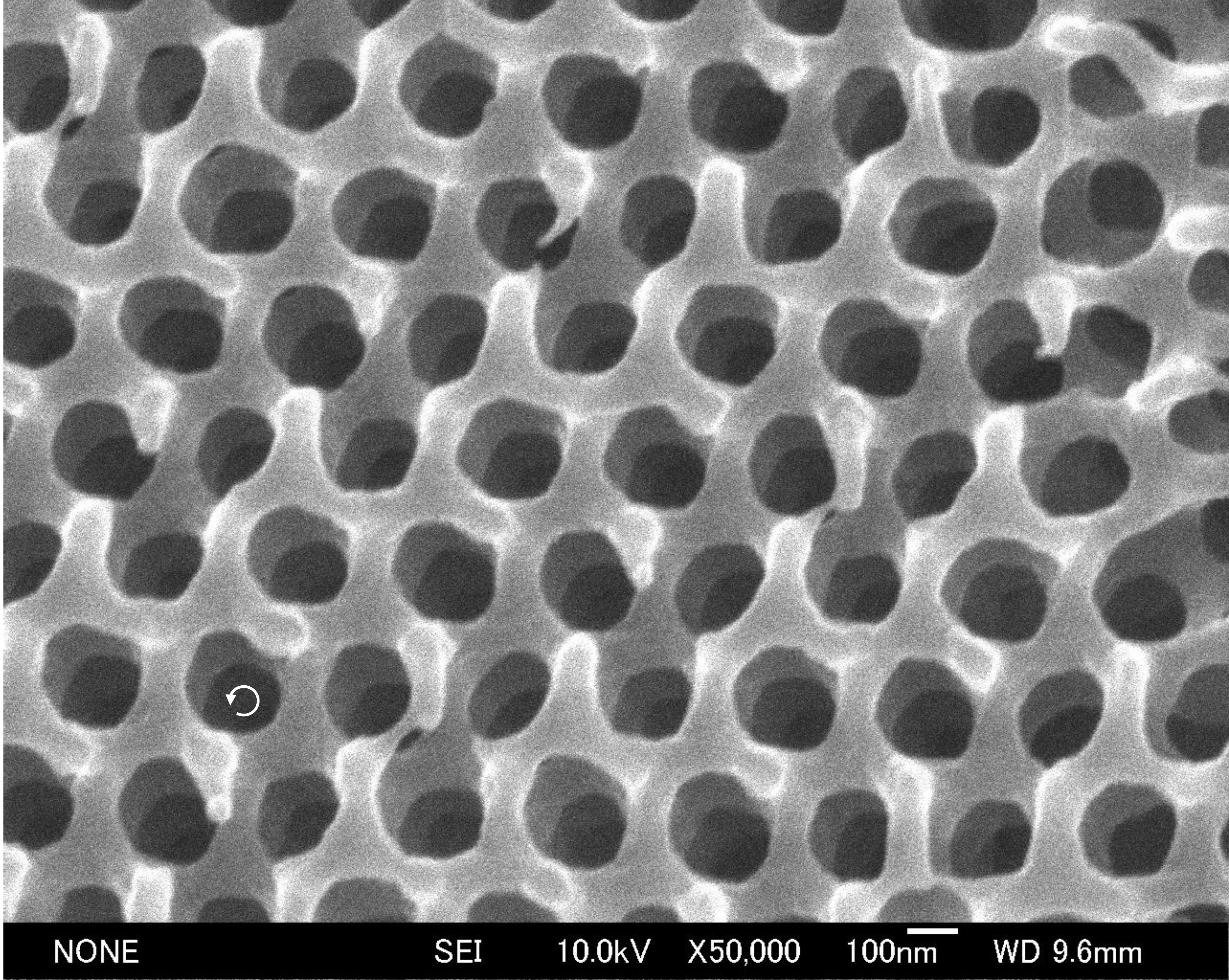

specimen No. 1
scale No. 1
domain No. 21
[111] lh spiral
LH gyroid

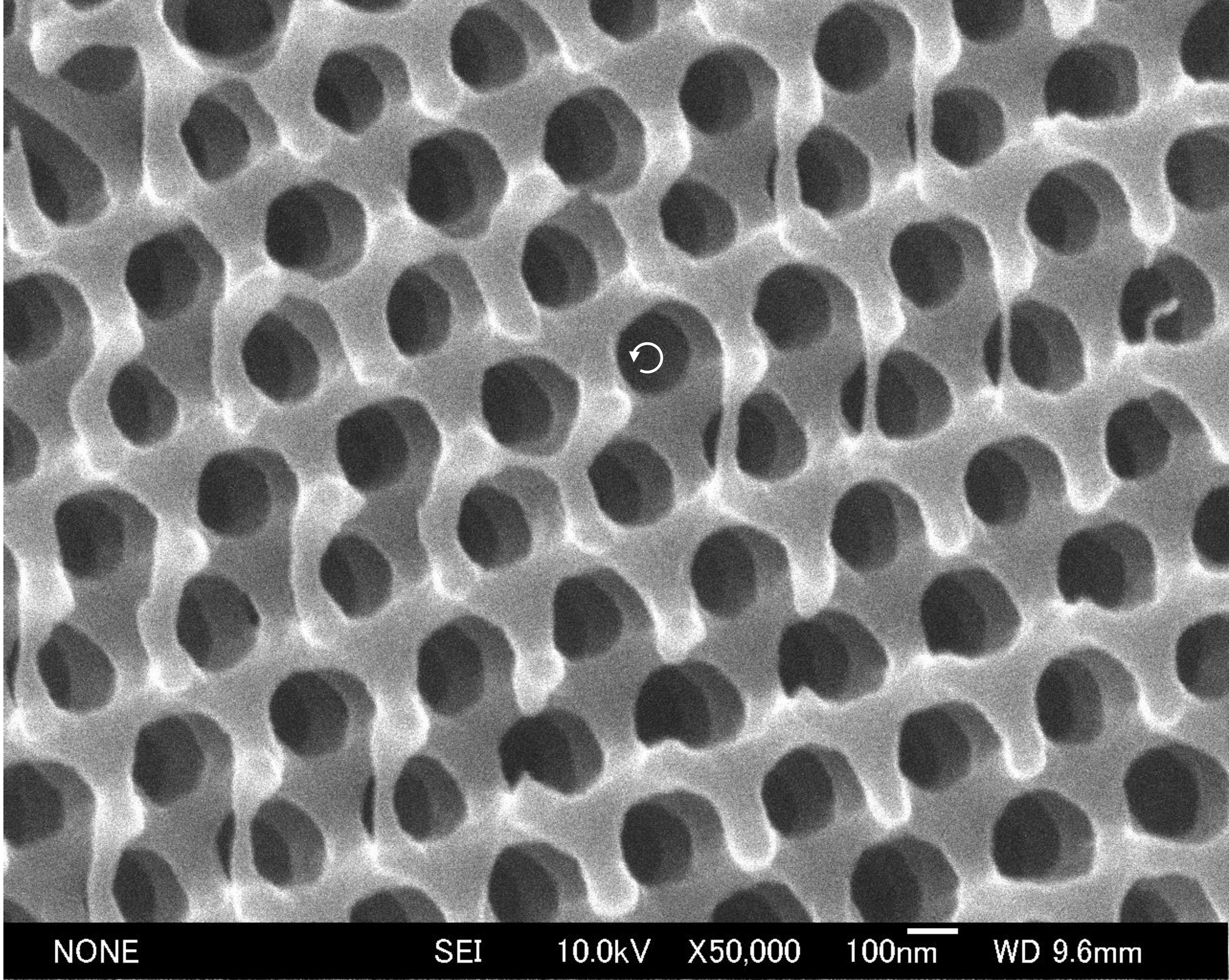

specimen No. 1
scale No. 1
domain No. 22
[100] rh spiral
LH gyroid

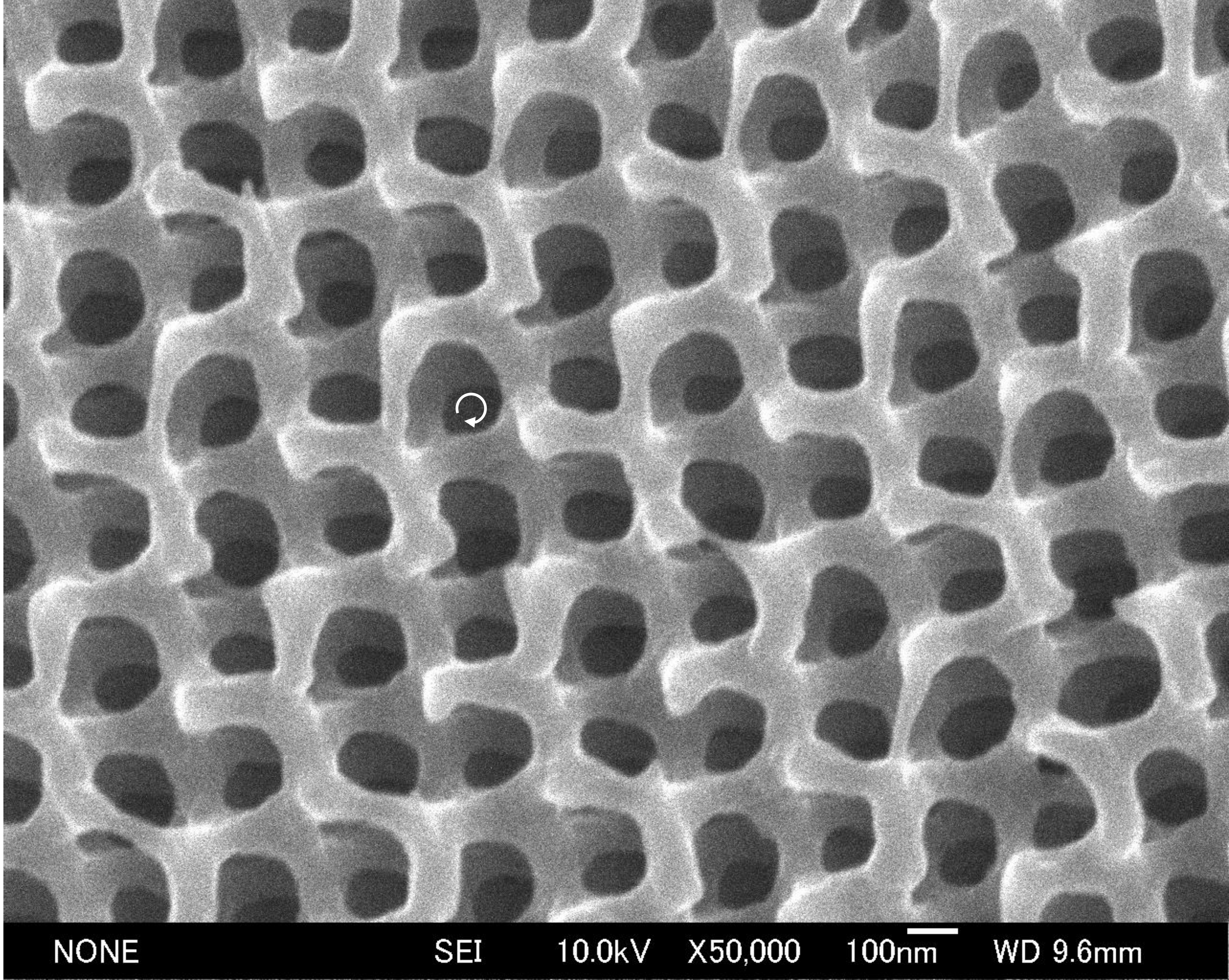

specimen No. 1
scale No. 1
domain No. 23
[111] lh spiral
LH gyroid

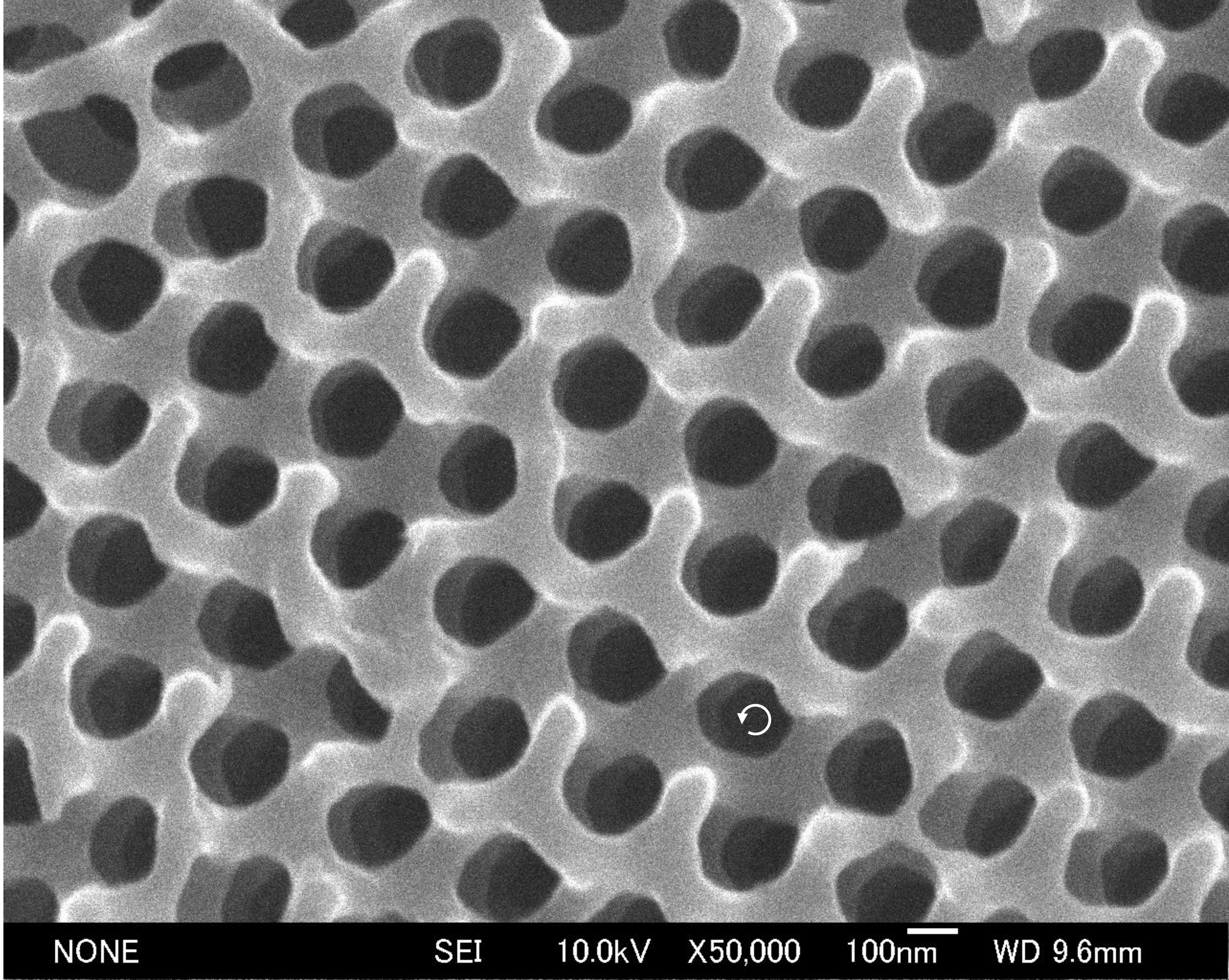

specimen No. 1
scale No. 1
domain No. 24
[100] lh spiral
RH gyroid

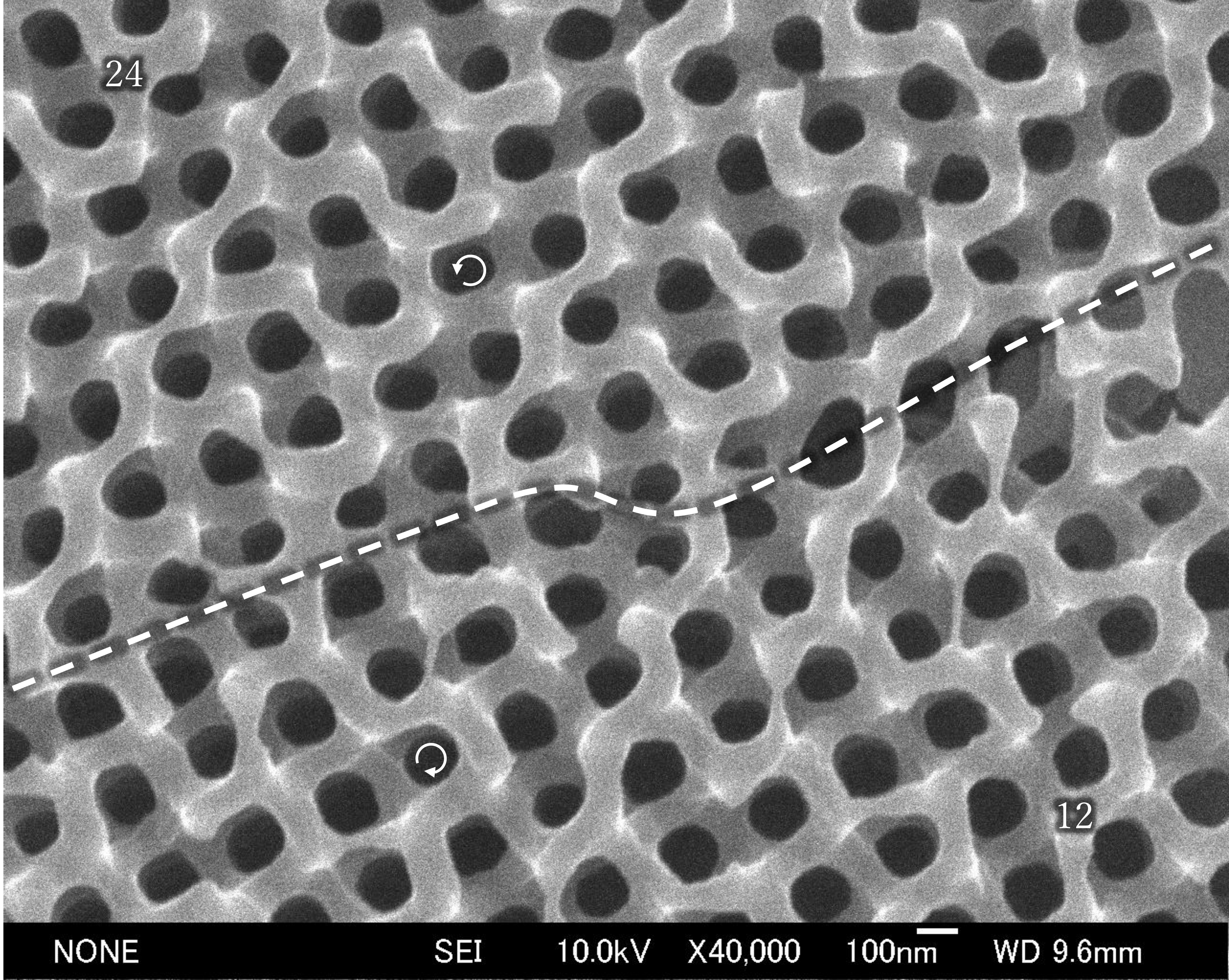

specimen No. 1
scale No. 1
domain No. 25
[100] rh spiral
LH gyroid

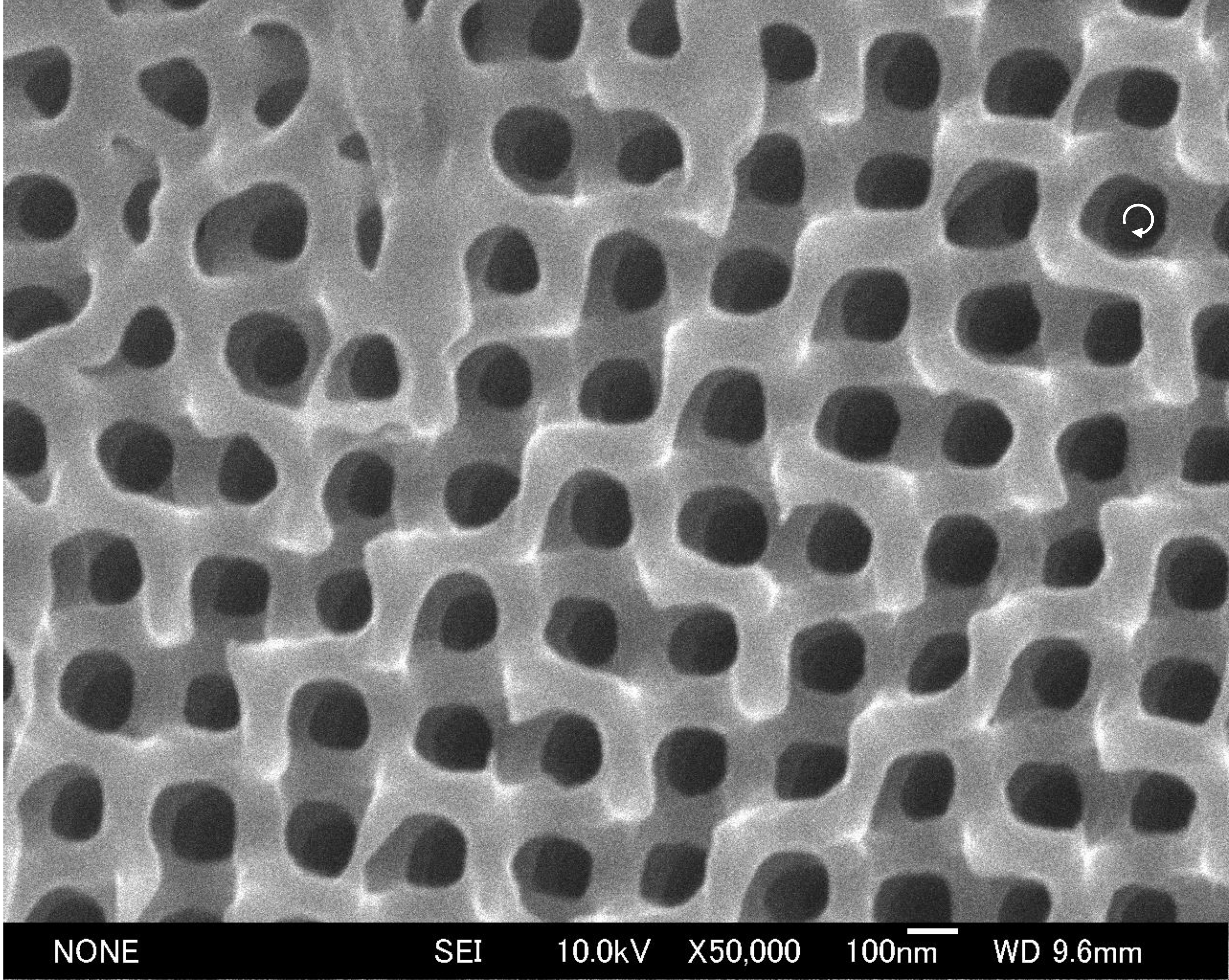

specimen No. 1
scale No. 1
domain No. 26
[100] rh spiral
LH gyroid

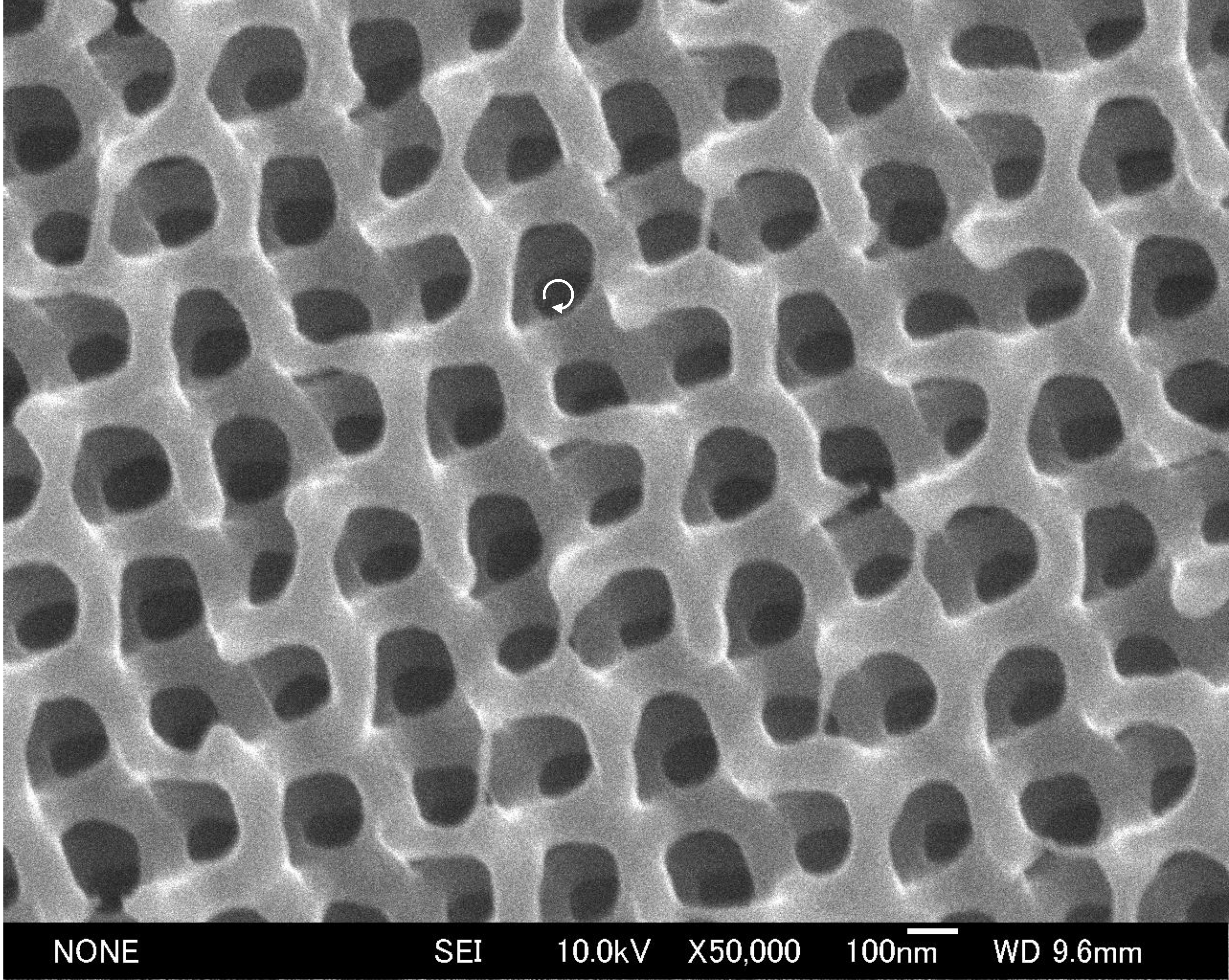

specimen No. 1
scale No. 1
domain No. 27
[100] rh spiral
LH gyroid

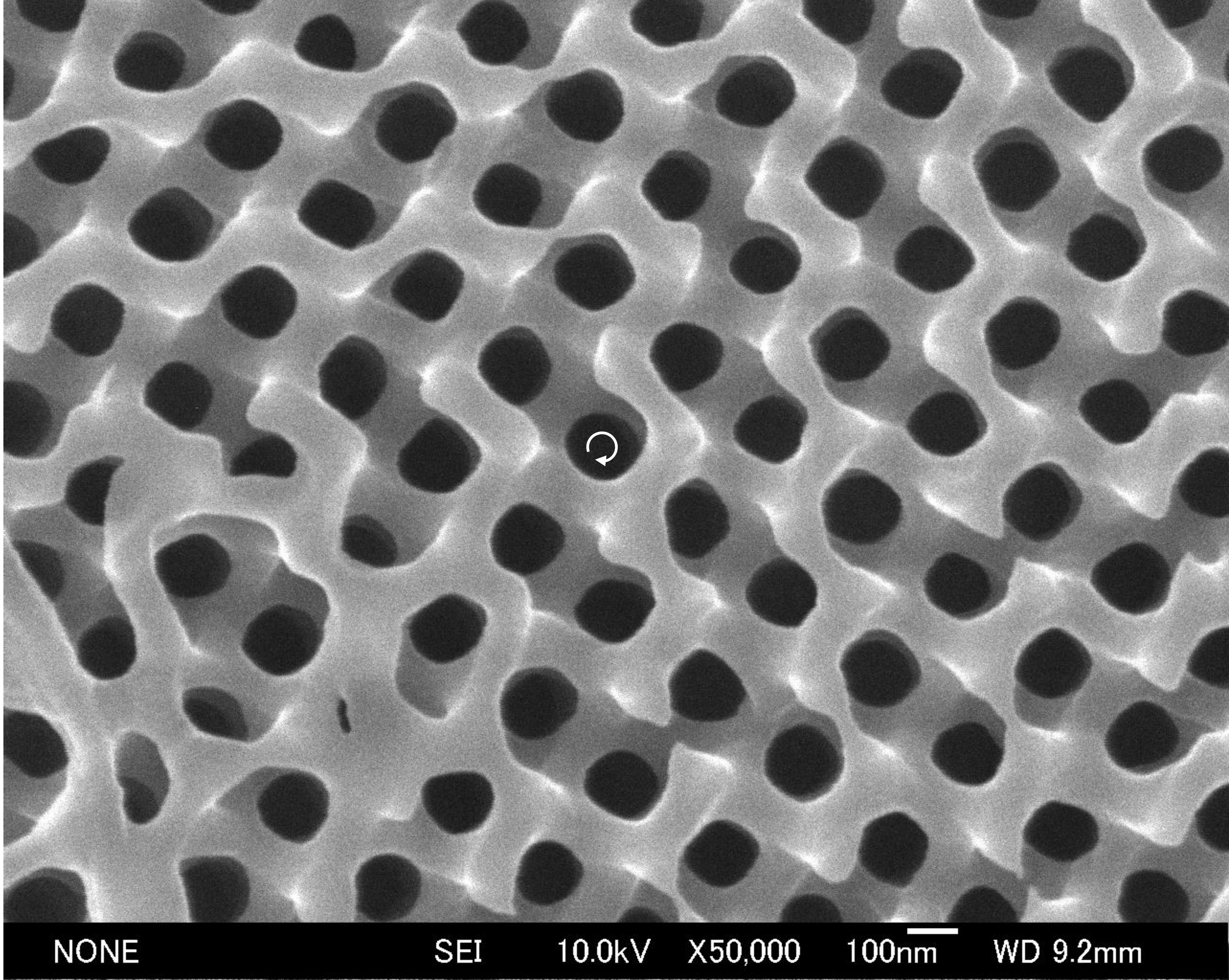

specimen No. 1
scale No. 1
domain No. 28
[100] rh spiral
LH gyroid

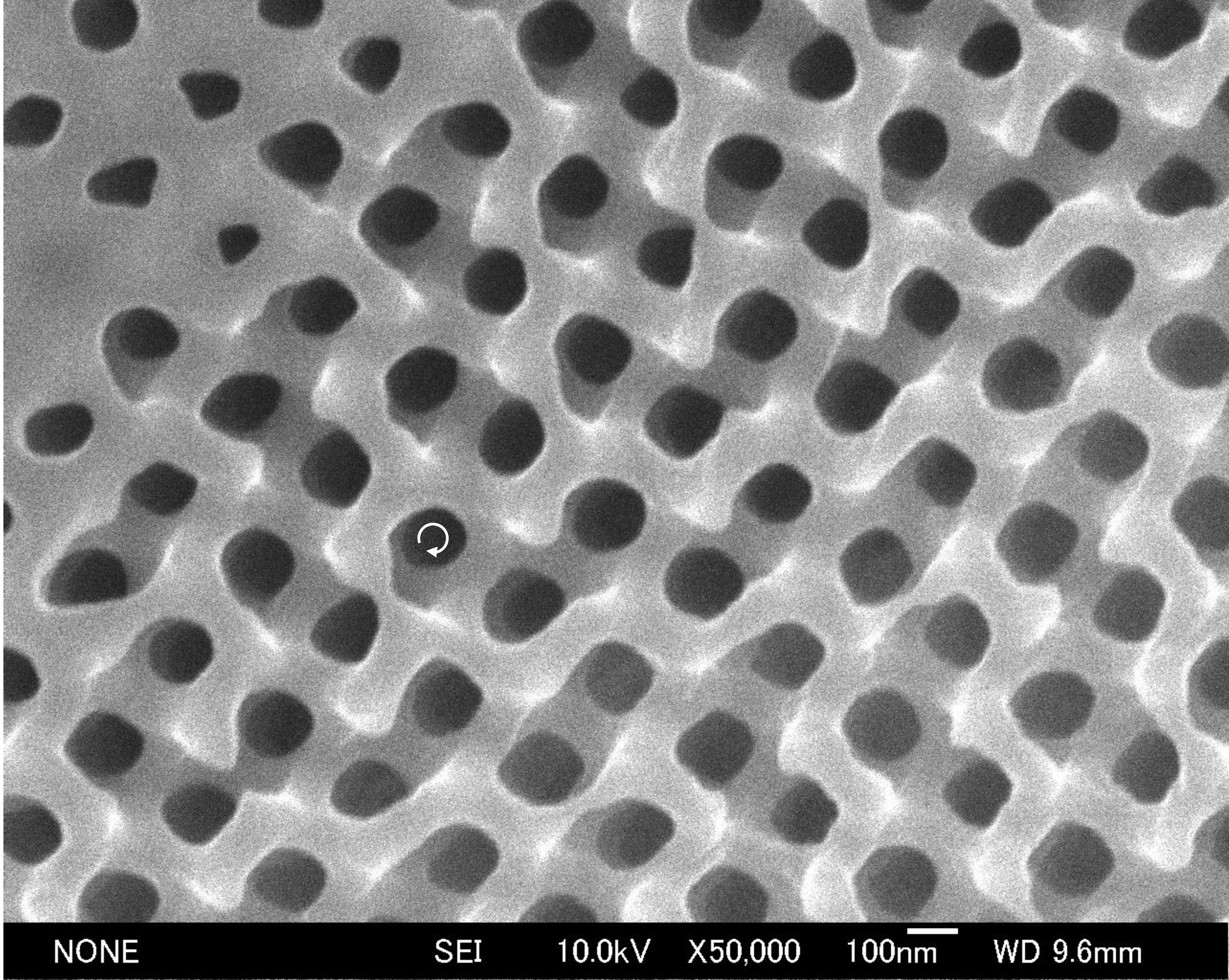

specimen No. 1
scale No. 1
domain No. 29
[100] lh spiral
RH gyroid

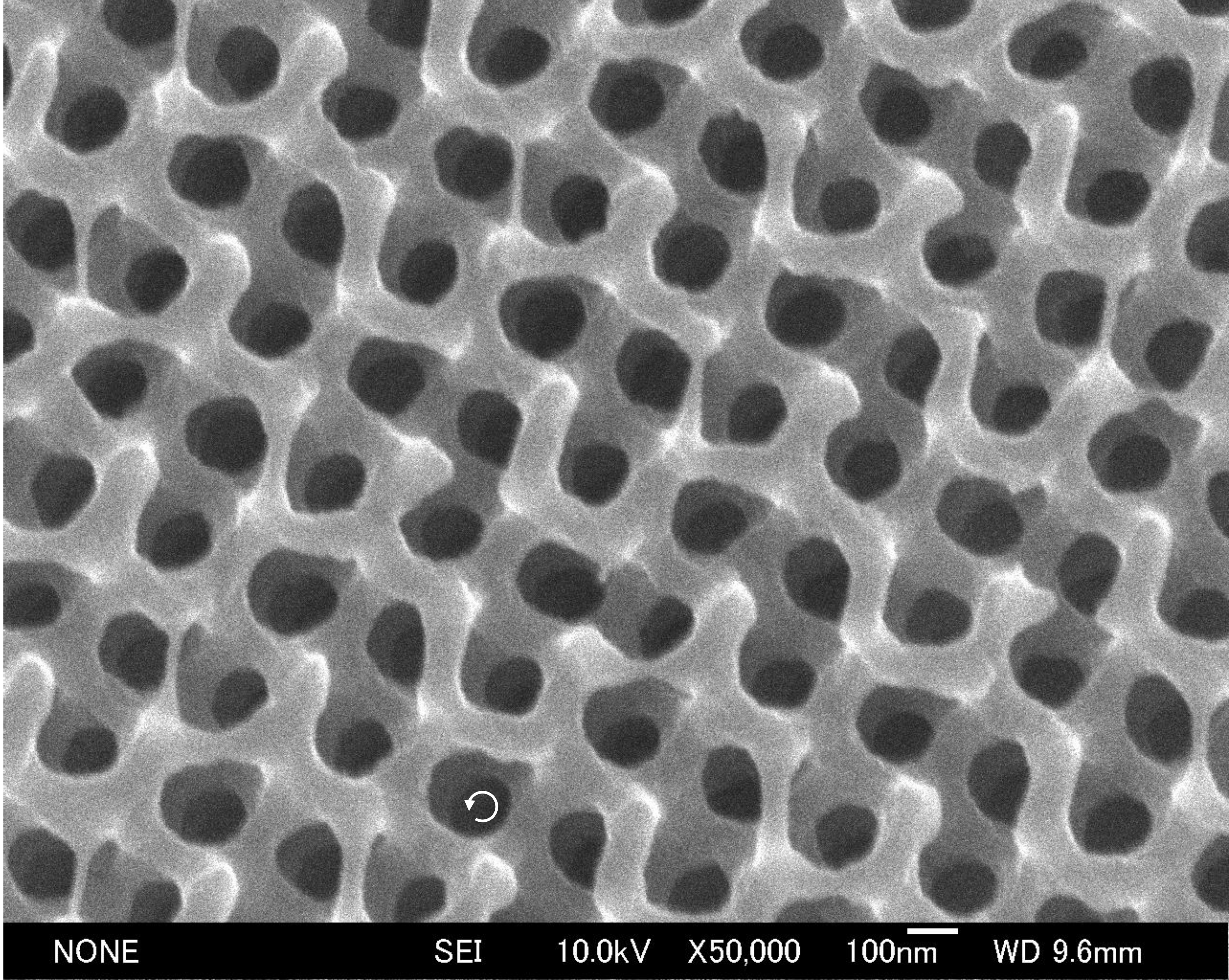

specimen No. 1
scale No. 1
domain No. 30
[100] rh spiral
LH gyroid

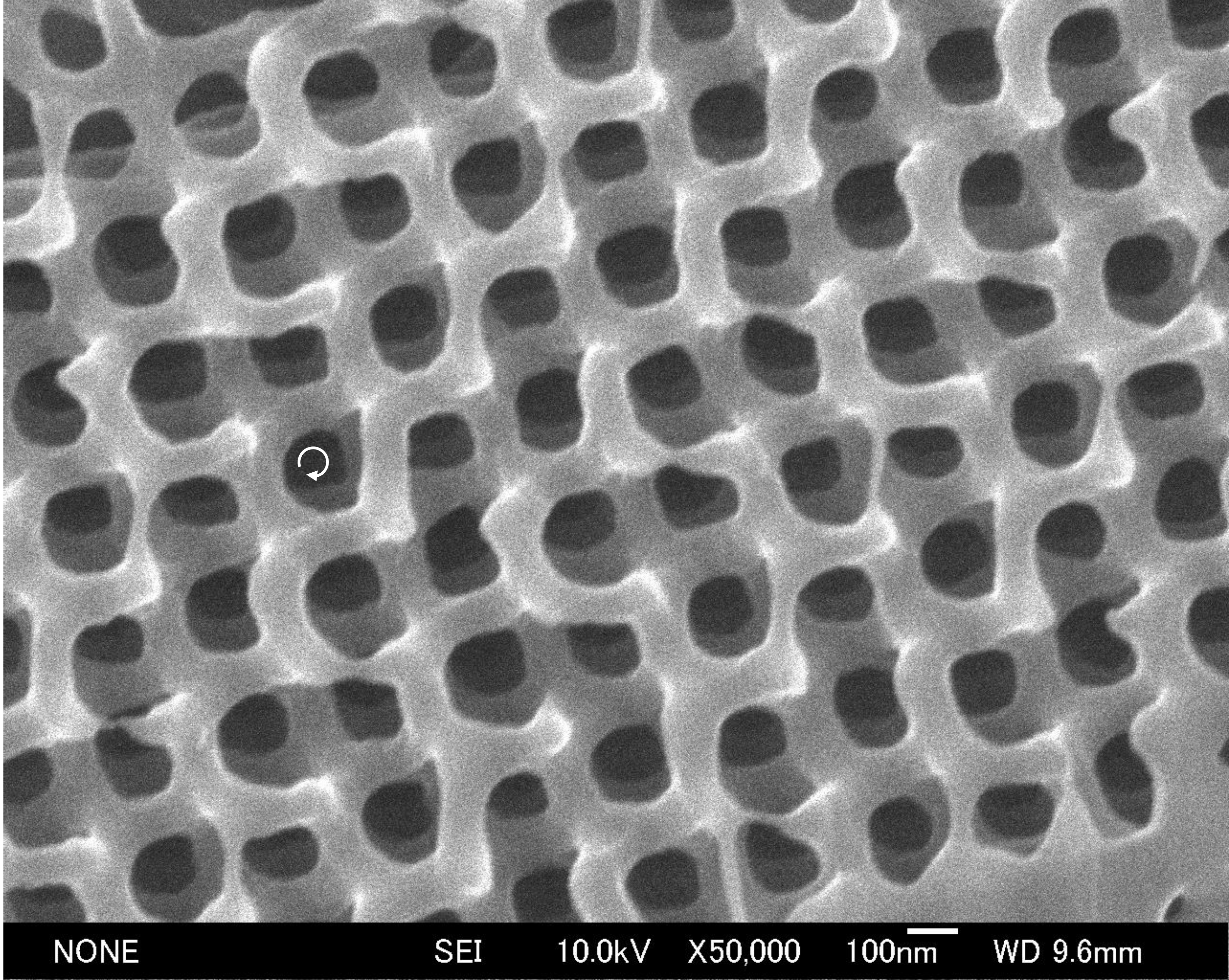

specimen No. 1
scale No. 1
domain No. 31
[100] lh spiral
RH gyroid

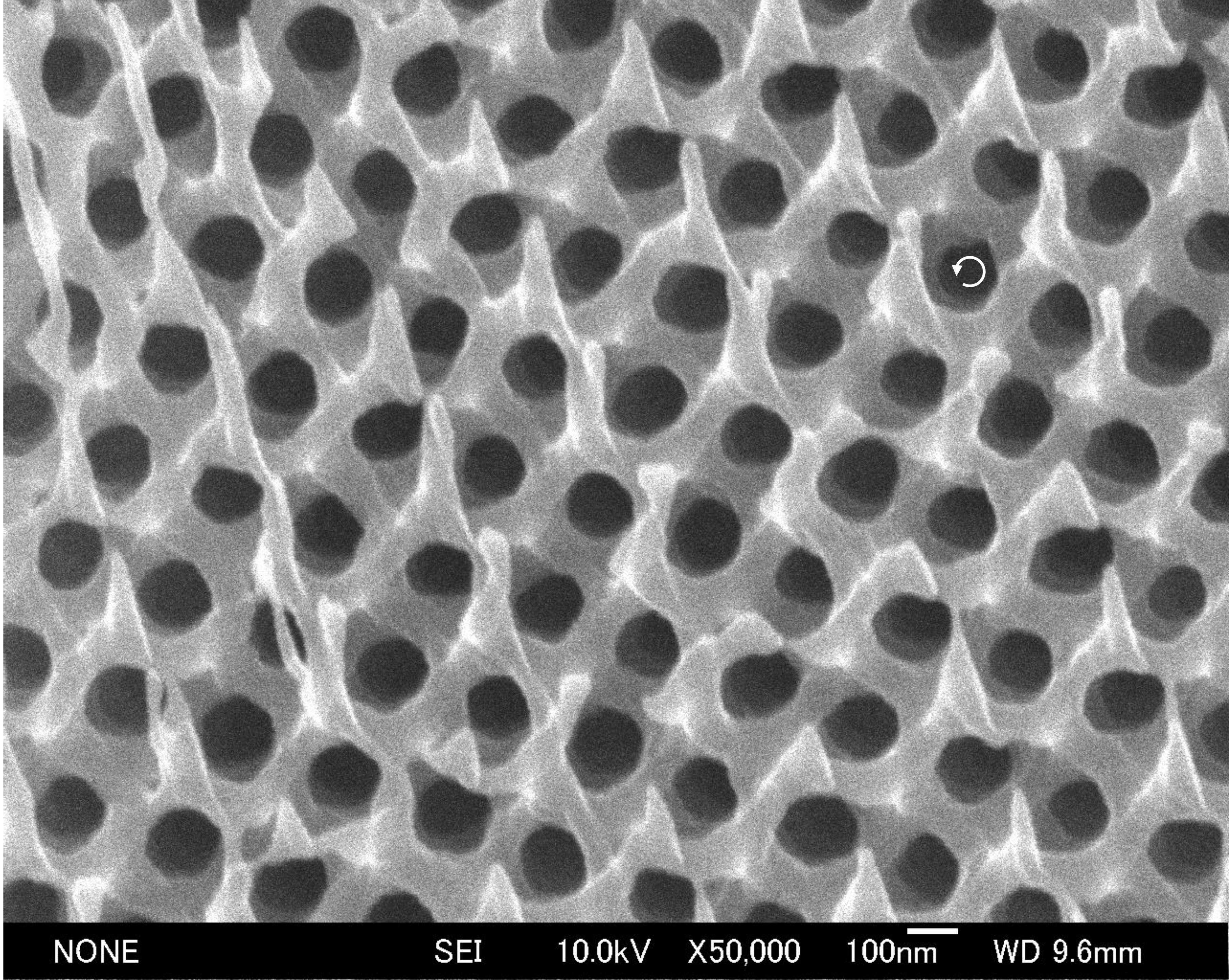

Supplement: Supplementary file 5 — Supplementary Information 5. [file 41598_2025_5750_MOESM5_ESM.pdf]
