# Supplementary material for: Study on the chirality of gyroid photonic crystals in butterfly wing scales
Source: Sci Rep. 2025 Jul 1;15:20968. doi: 10.1038/s41598-025-05750-2 (PMC12215952; doi:10.1038/s41598-025-05750-2)

specimen No. 1
scale No. 1

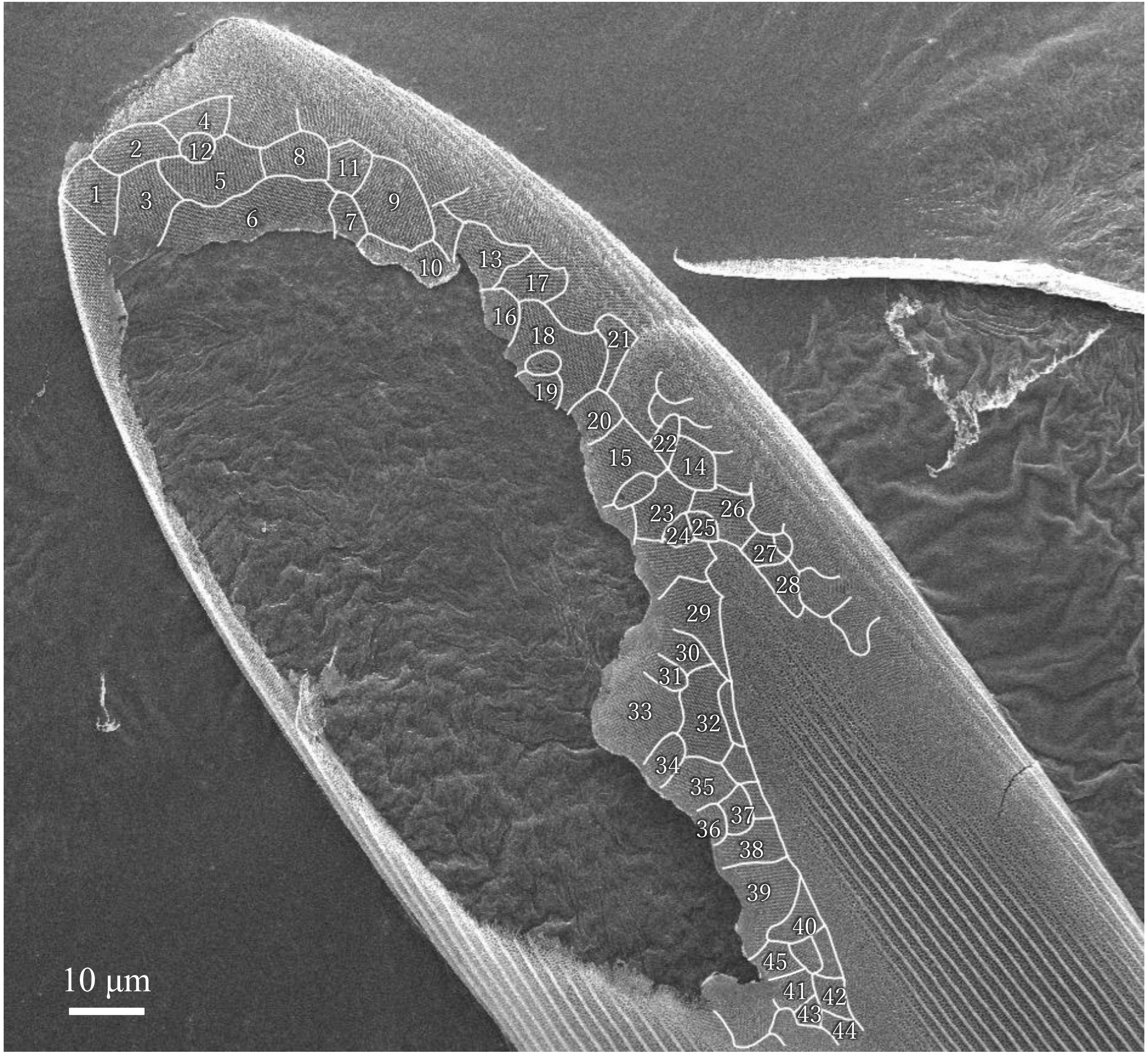

specimen No. 1
scale No. 1
domain No. 1
LH

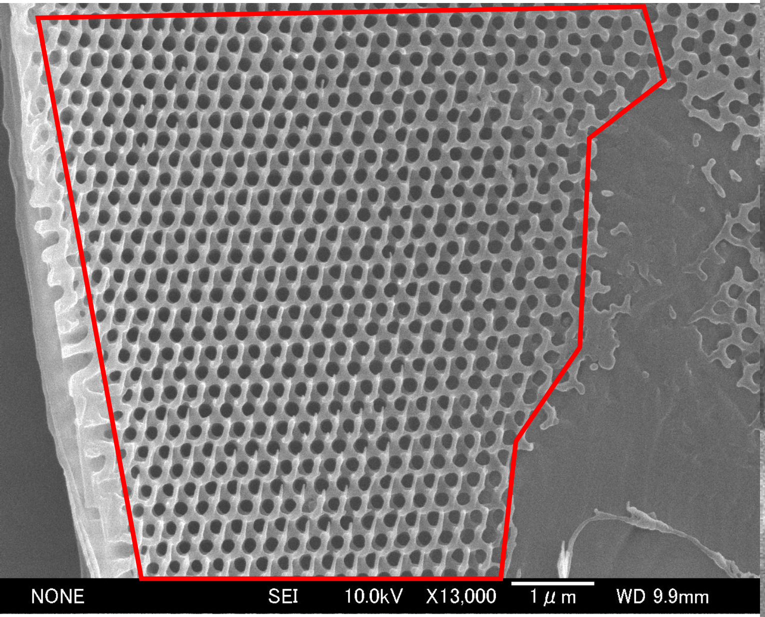

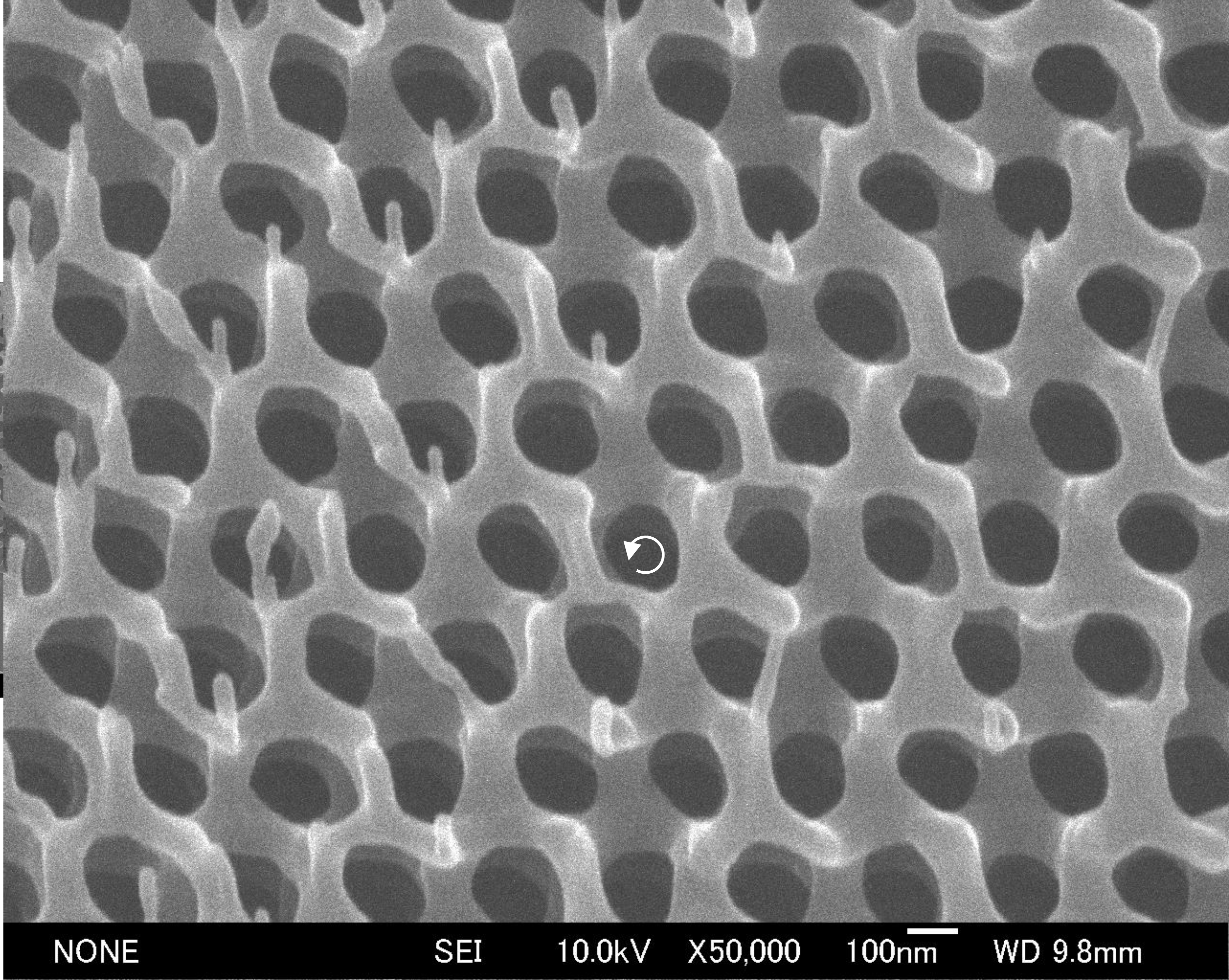

specimen No. 1
scale No. 1
domain No. 2
LH

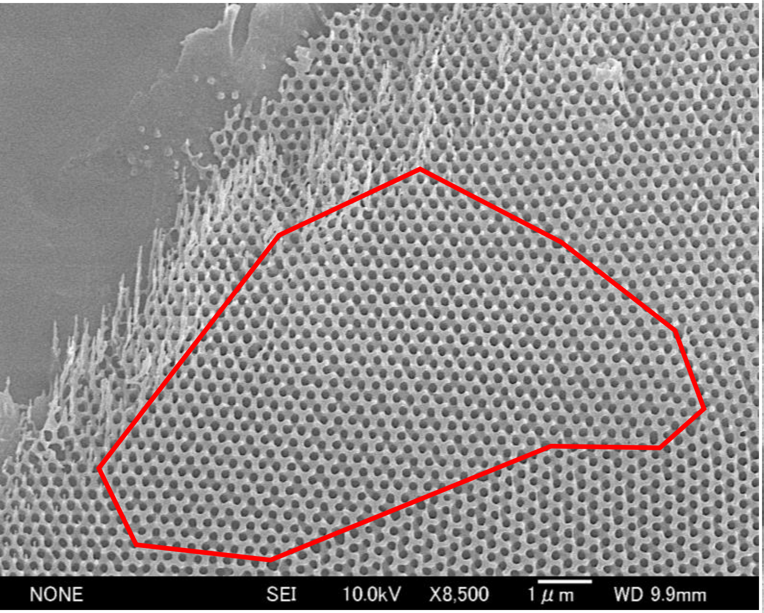

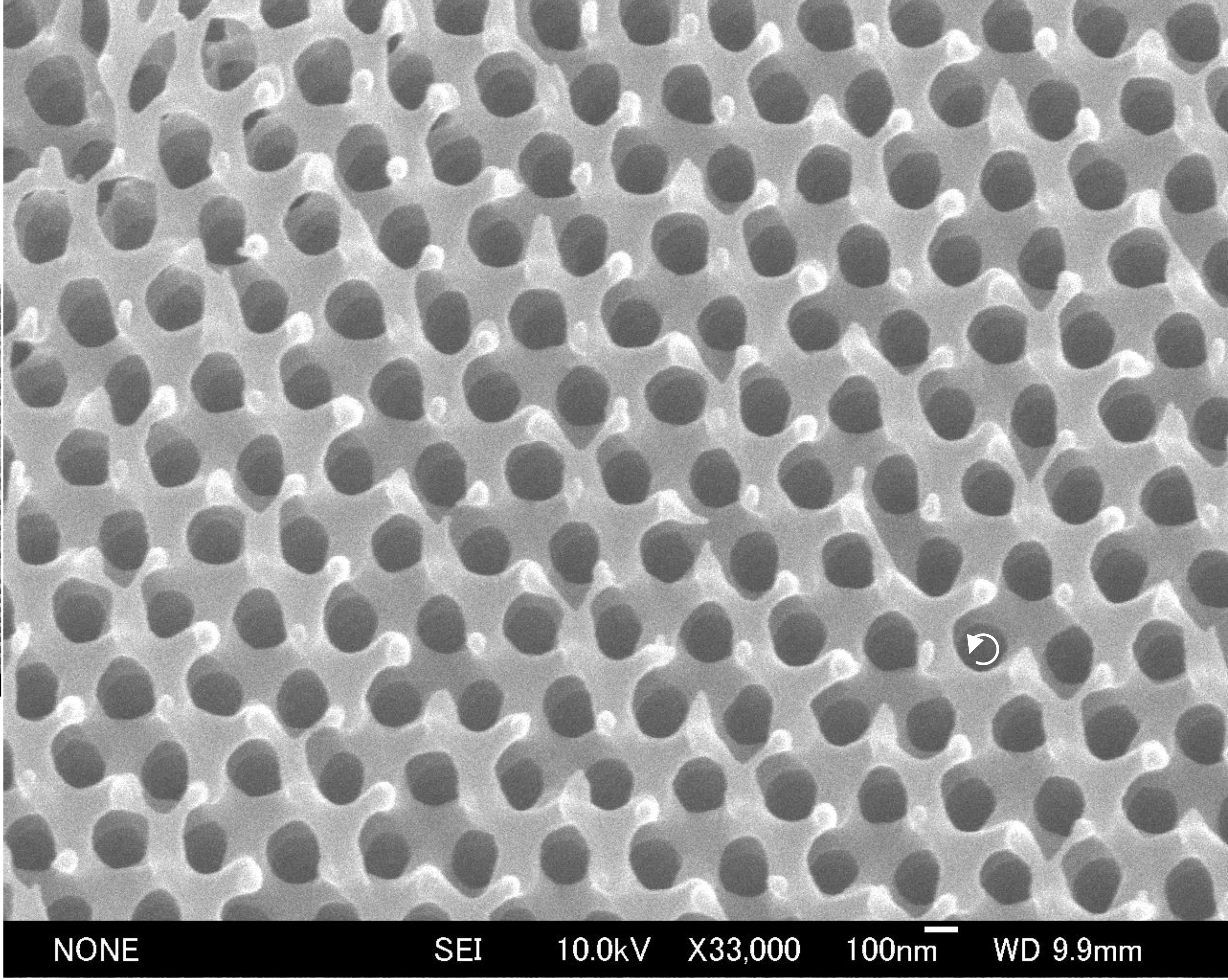

specimen No. 1
scale No. 1
domain No. 3
LH

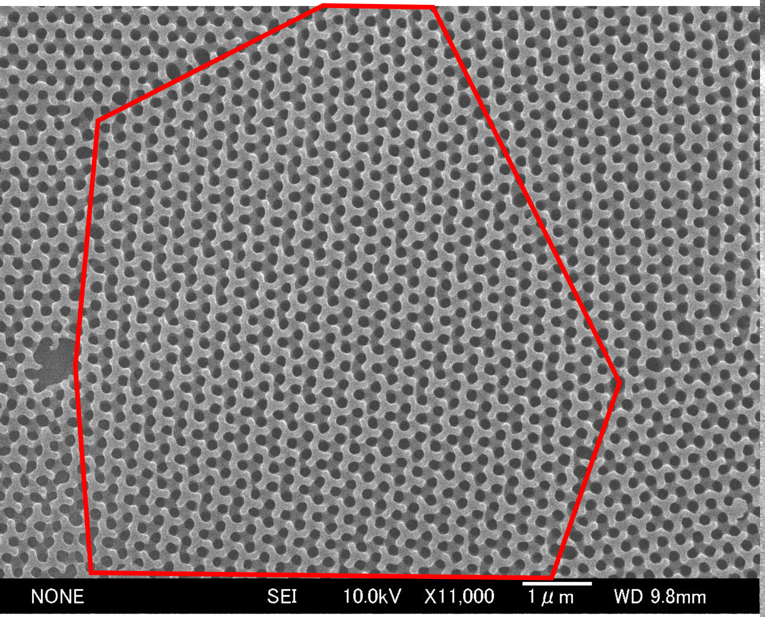

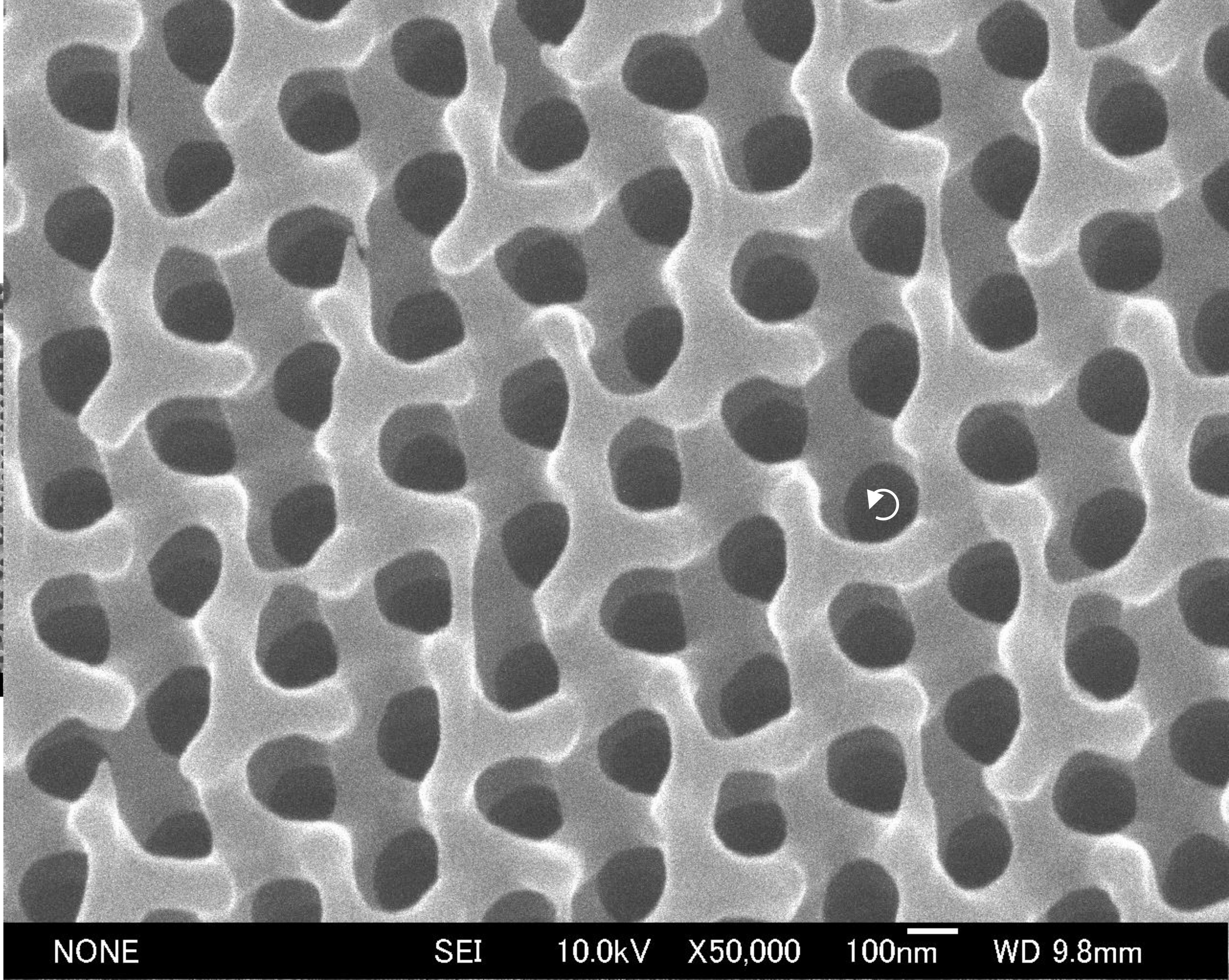

specimen No. 1
scale No. 1
domain No. 4
LH

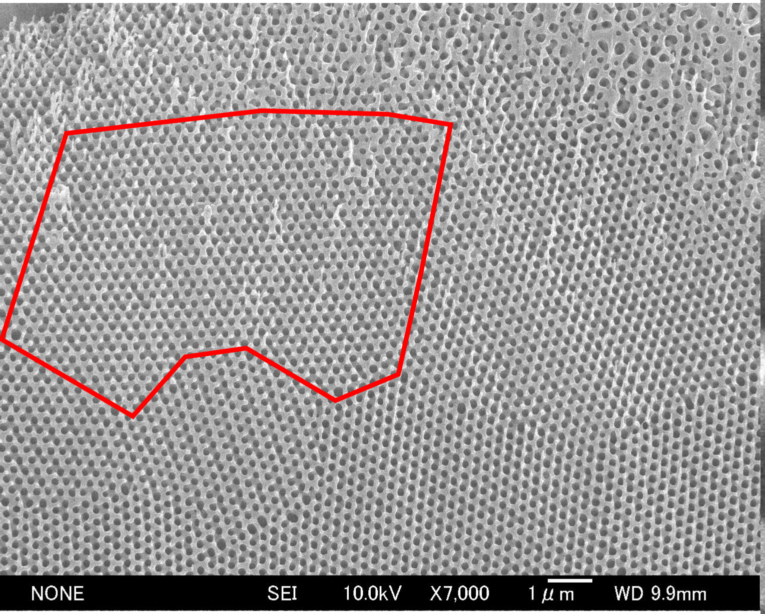

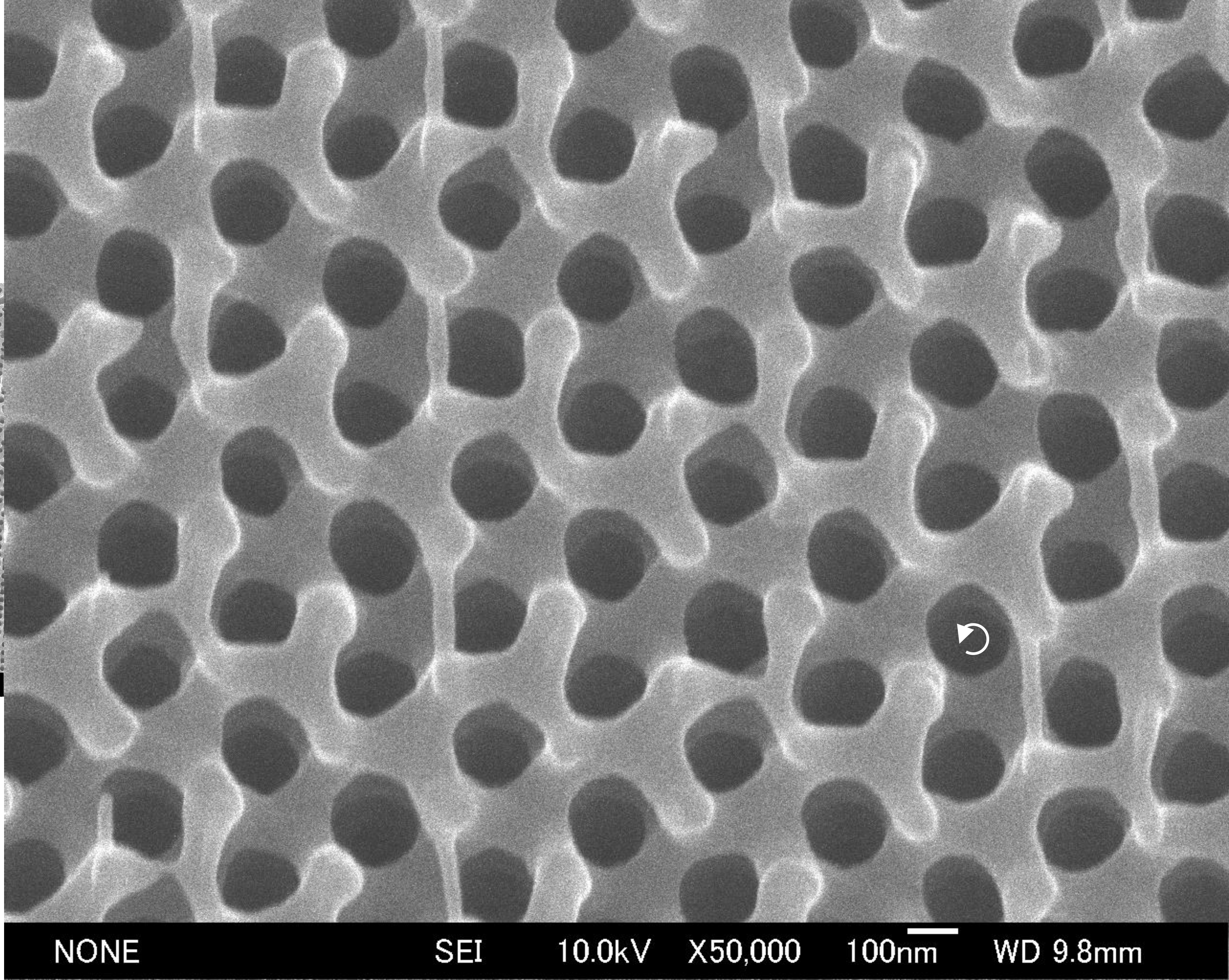

specimen No. 1
scale No. 1
domain No. 5
LH

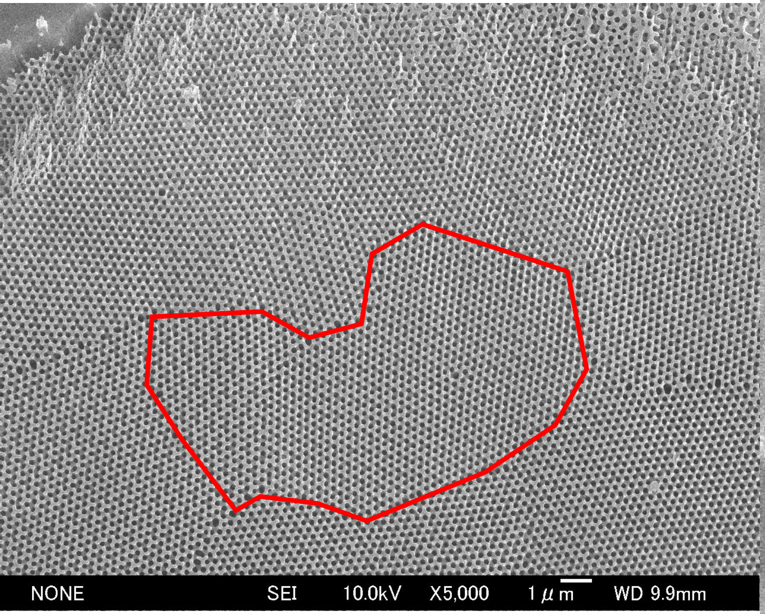

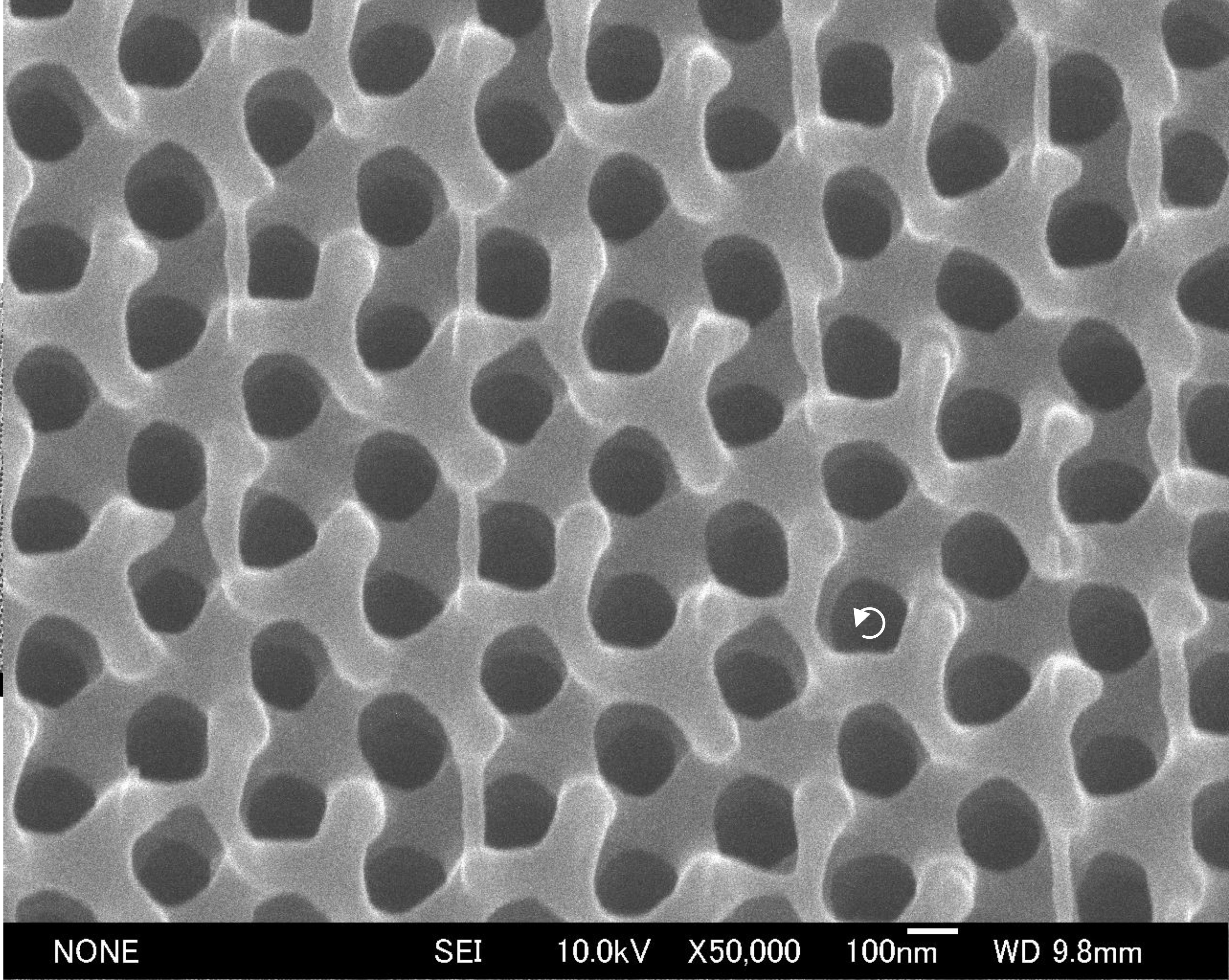

specimen No. 1
scale No. 1
domain No. 6
LH

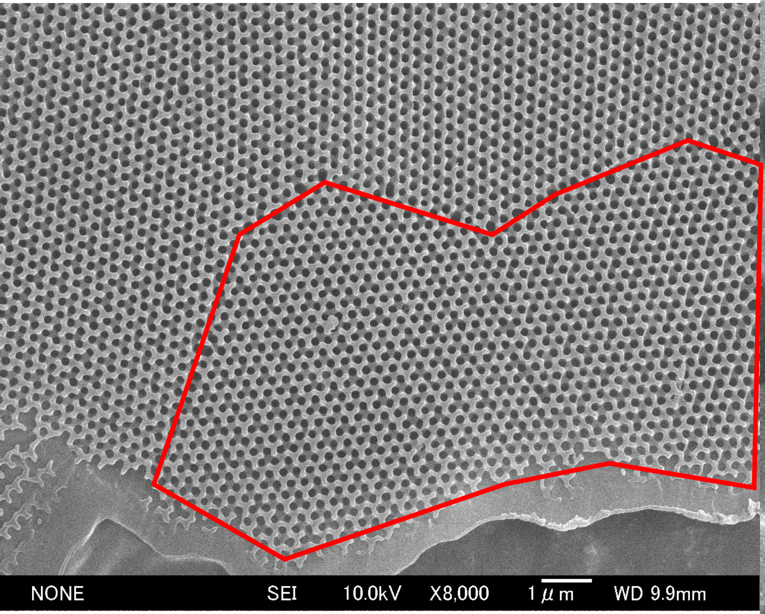

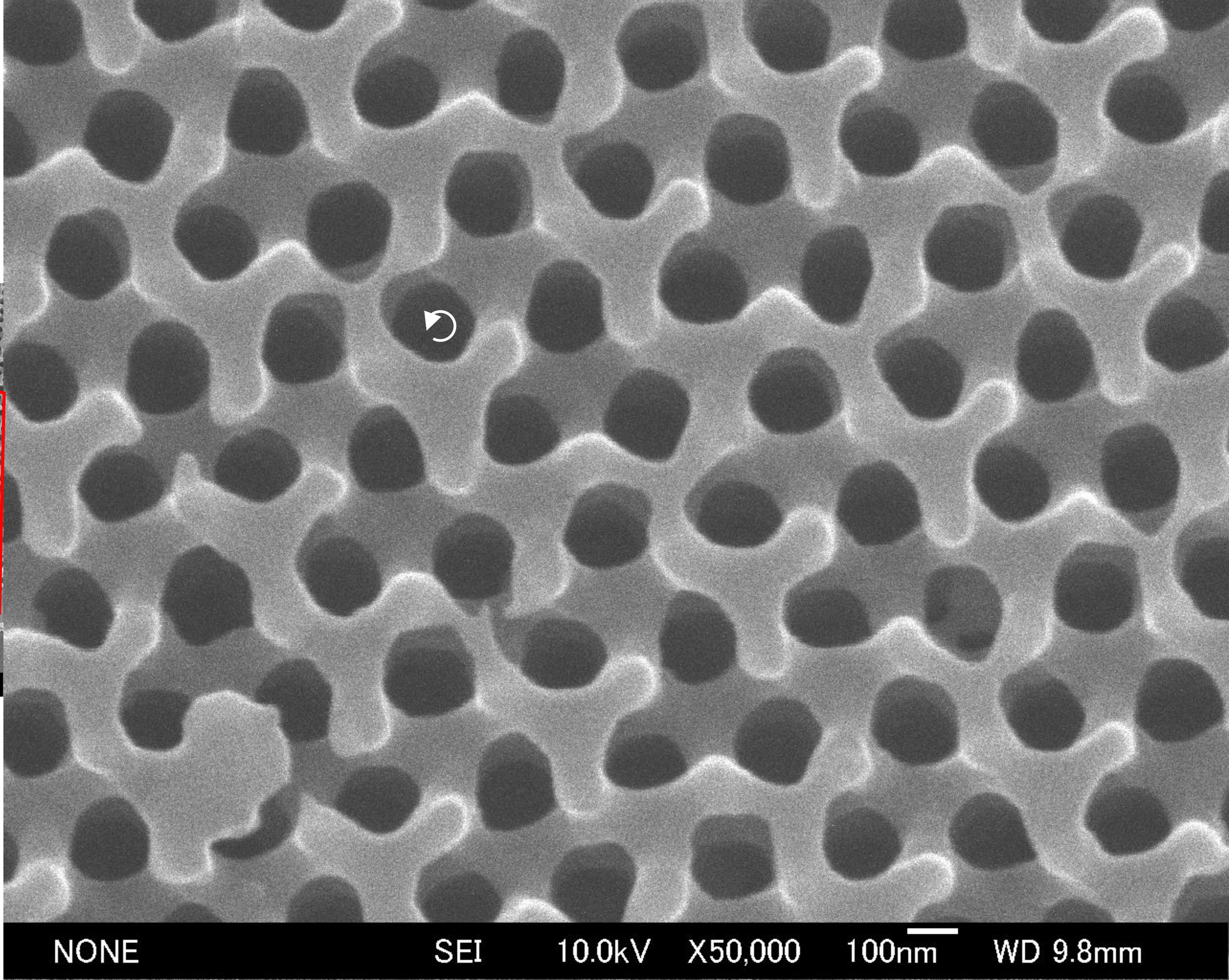

specimen No. 1
scale No. 1
domain No. 7
LH

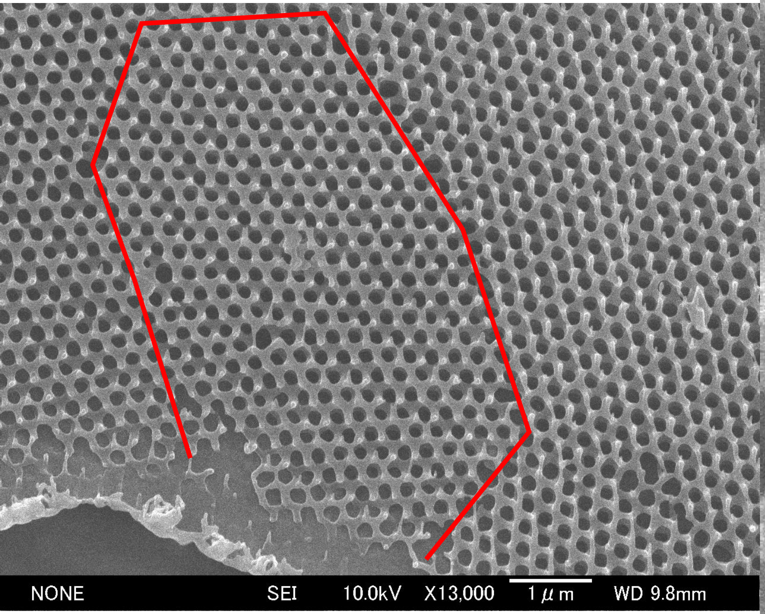

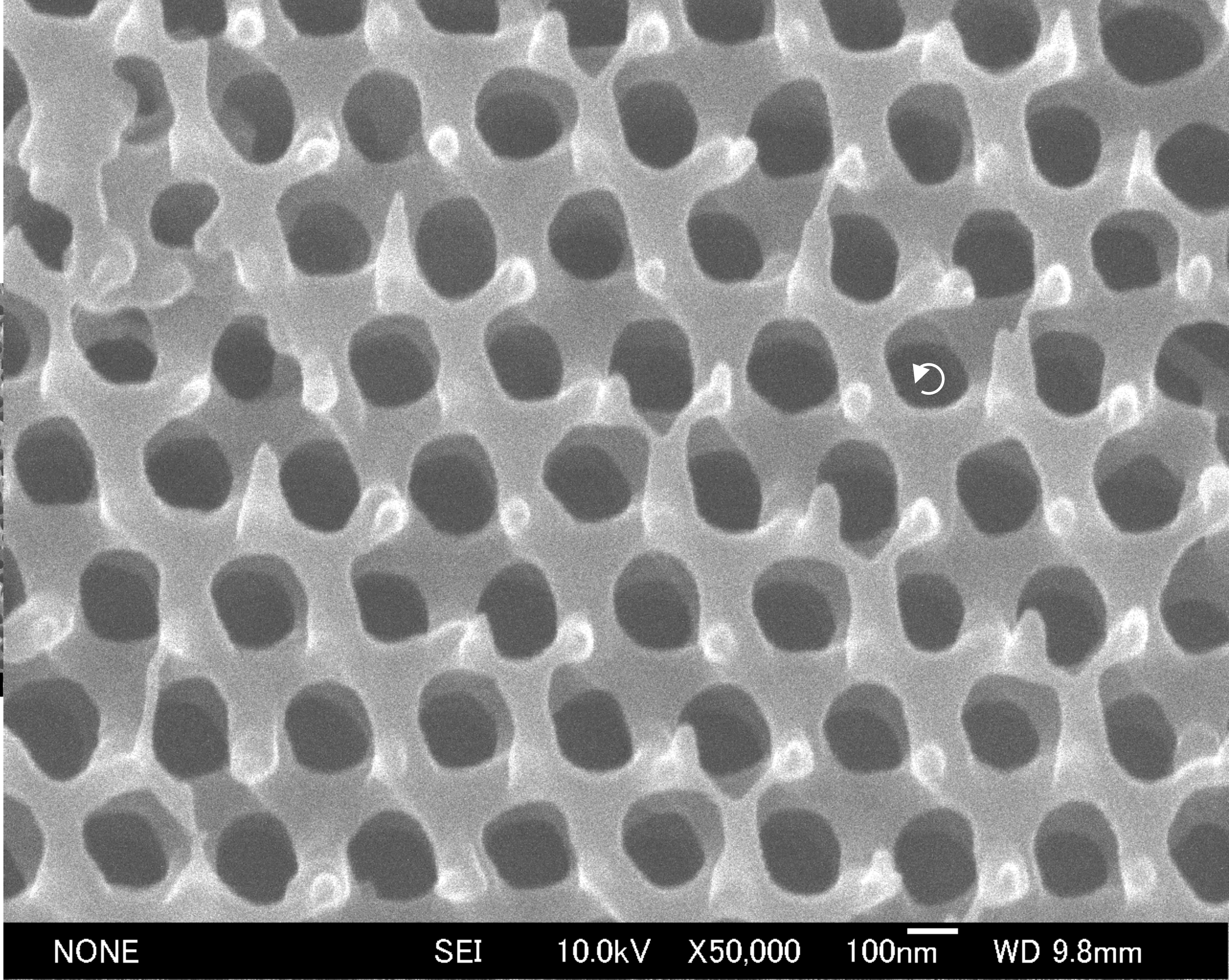

specimen No. 1
scale No. 1
domain No. 8
LH

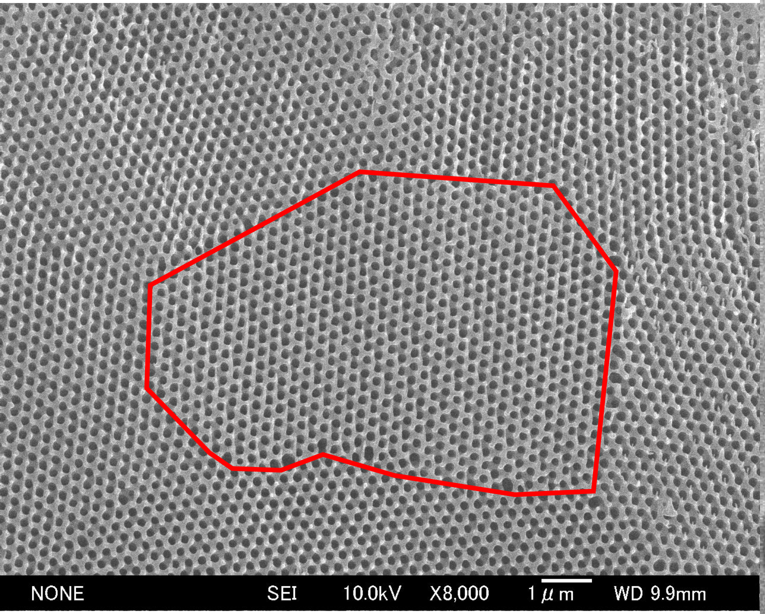

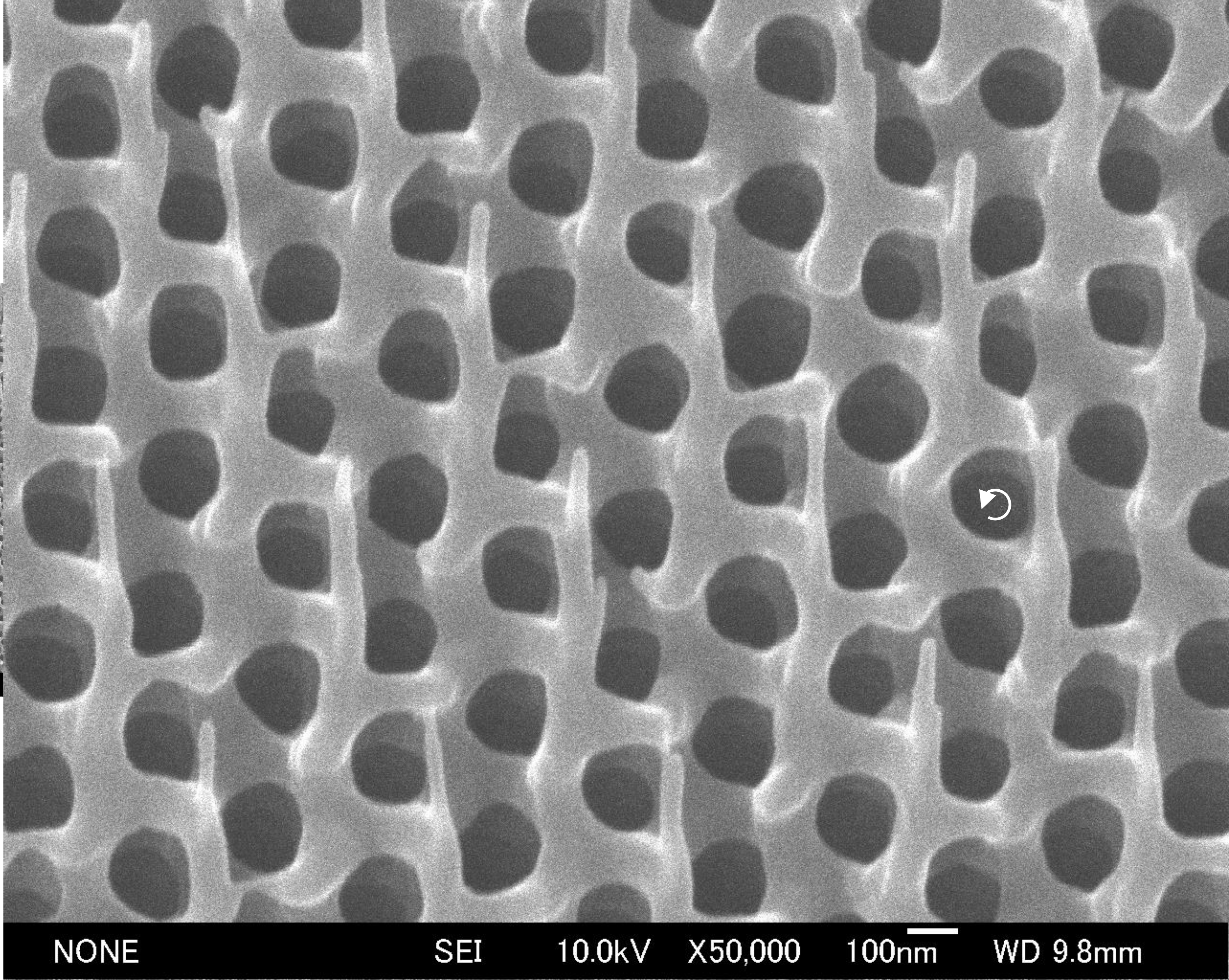

specimen No. 1
scale No. 1
domain No. 9
LH

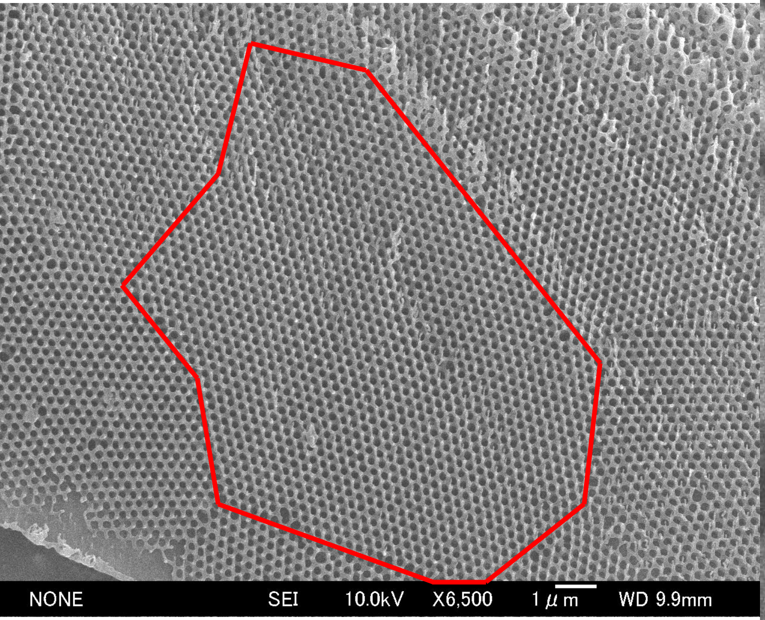

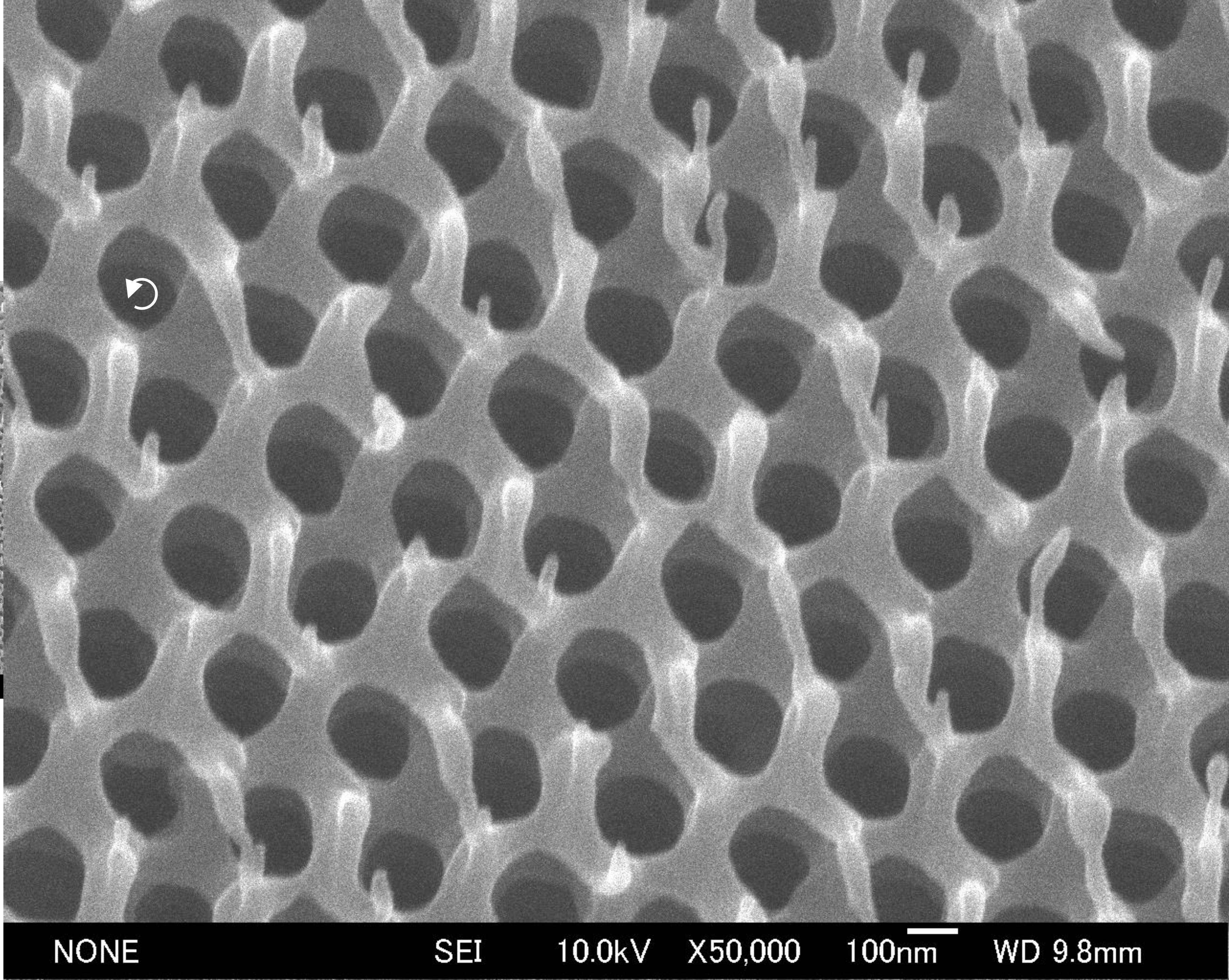

specimen No. 1
scale No. 1
domain No. 10
LH

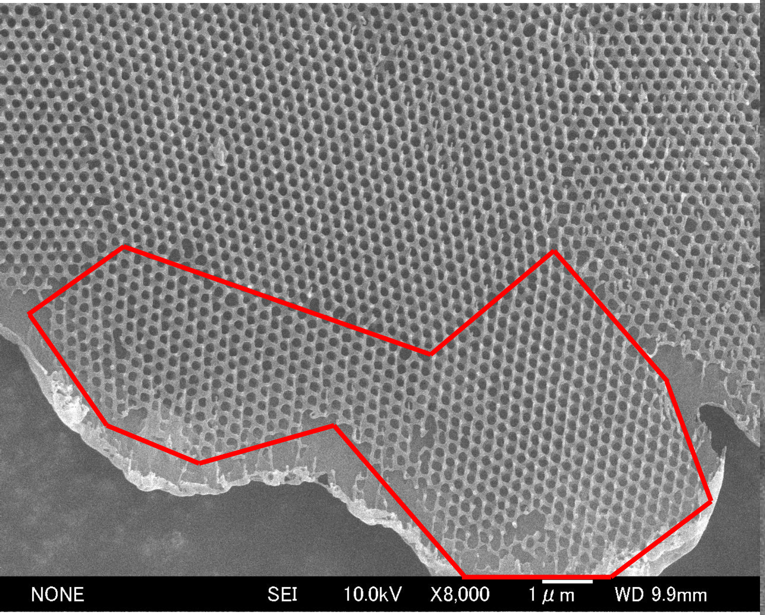

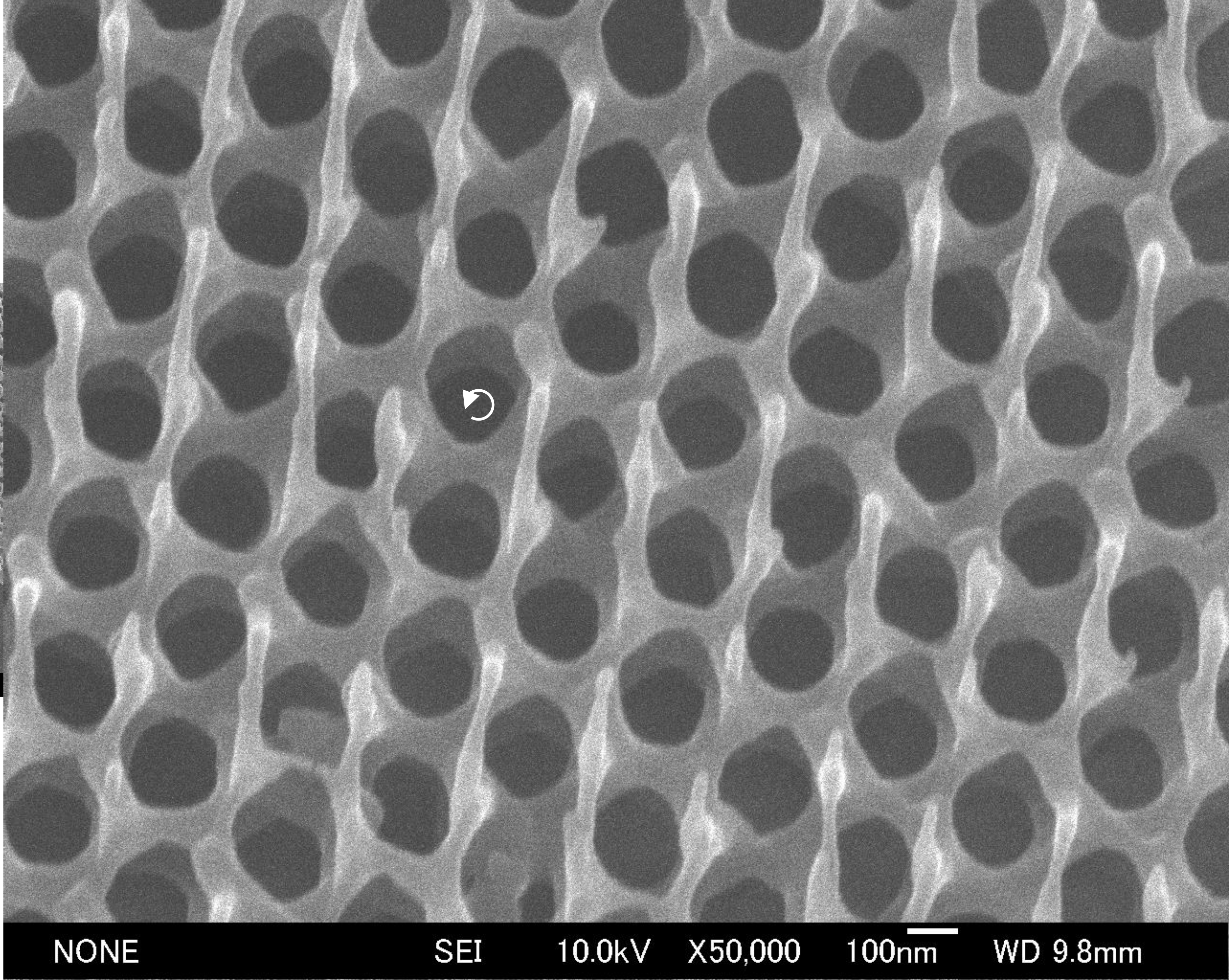

specimen No. 1
scale No. 1
domain No. 11
LH

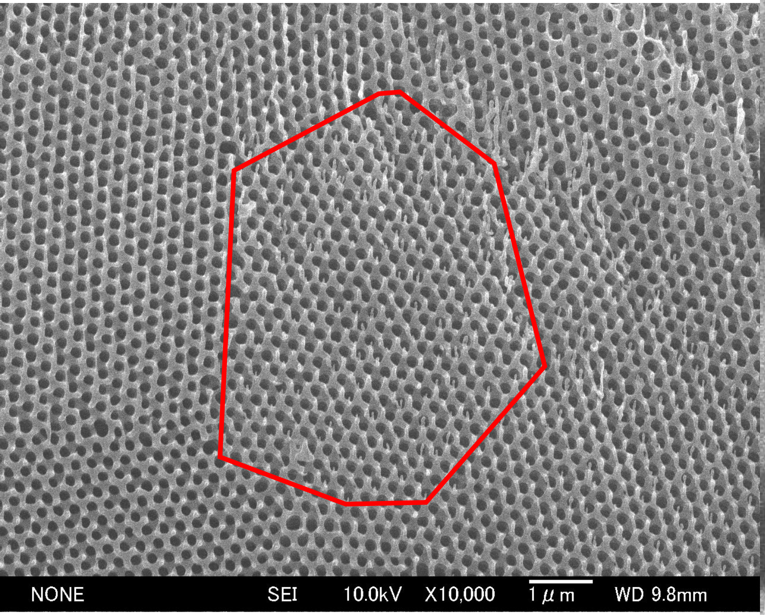

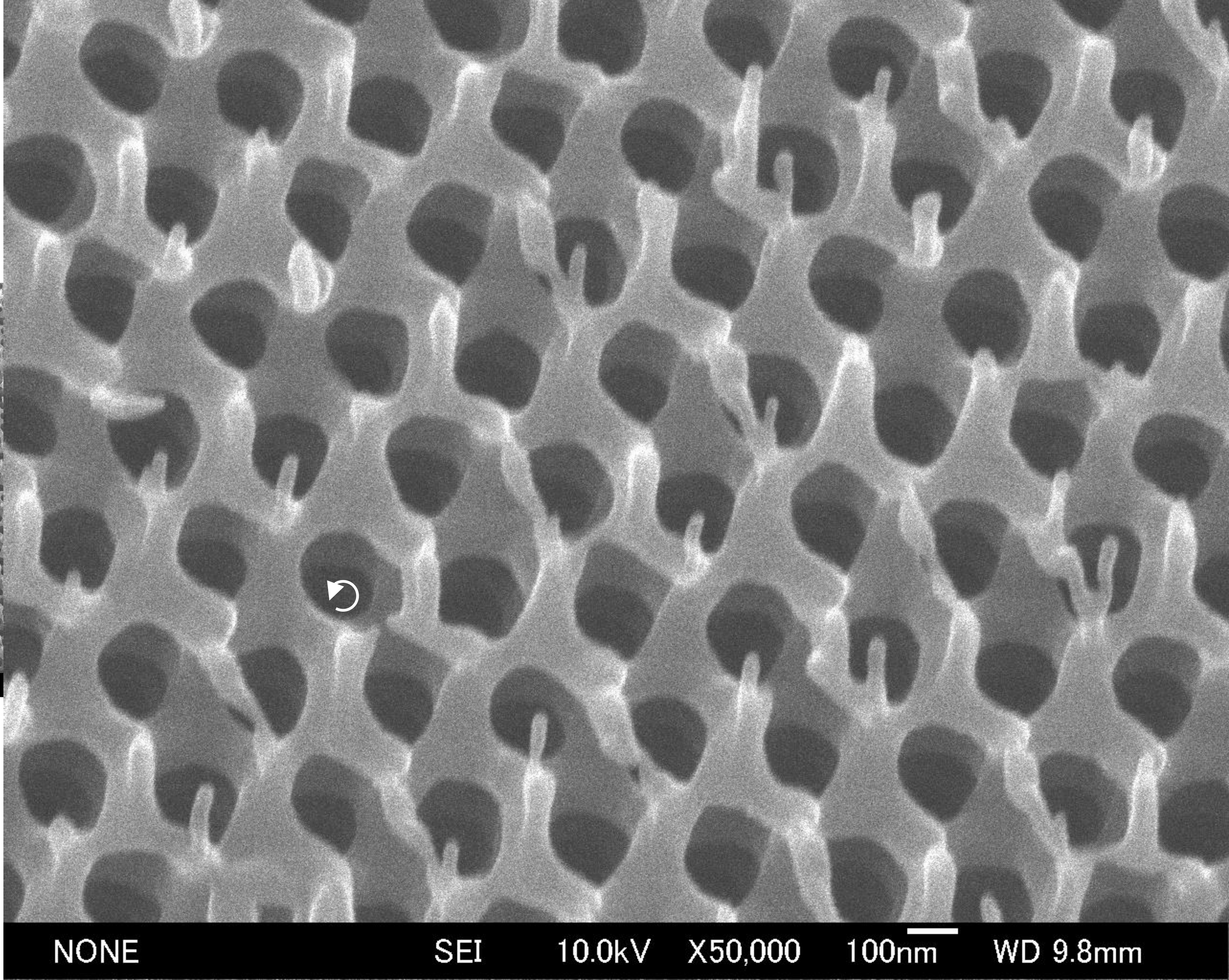

specimen No. 1
scale No. 1
domain No. 12
LH

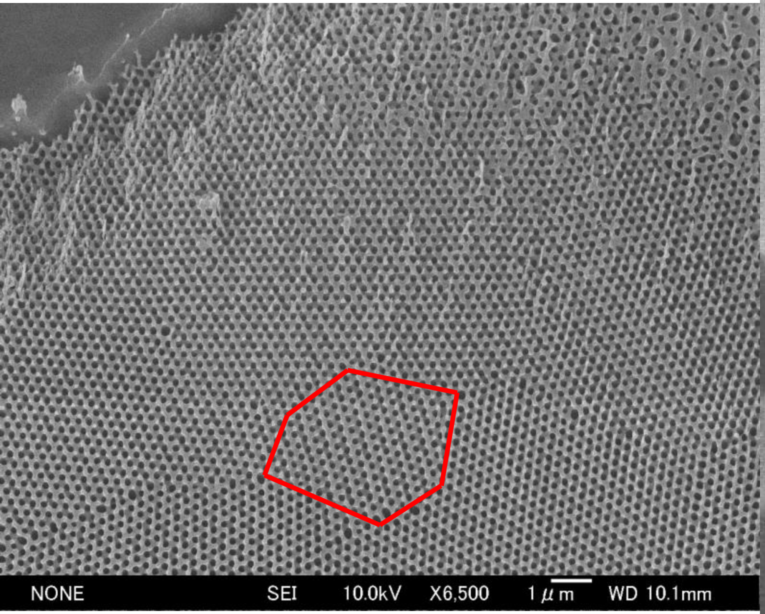

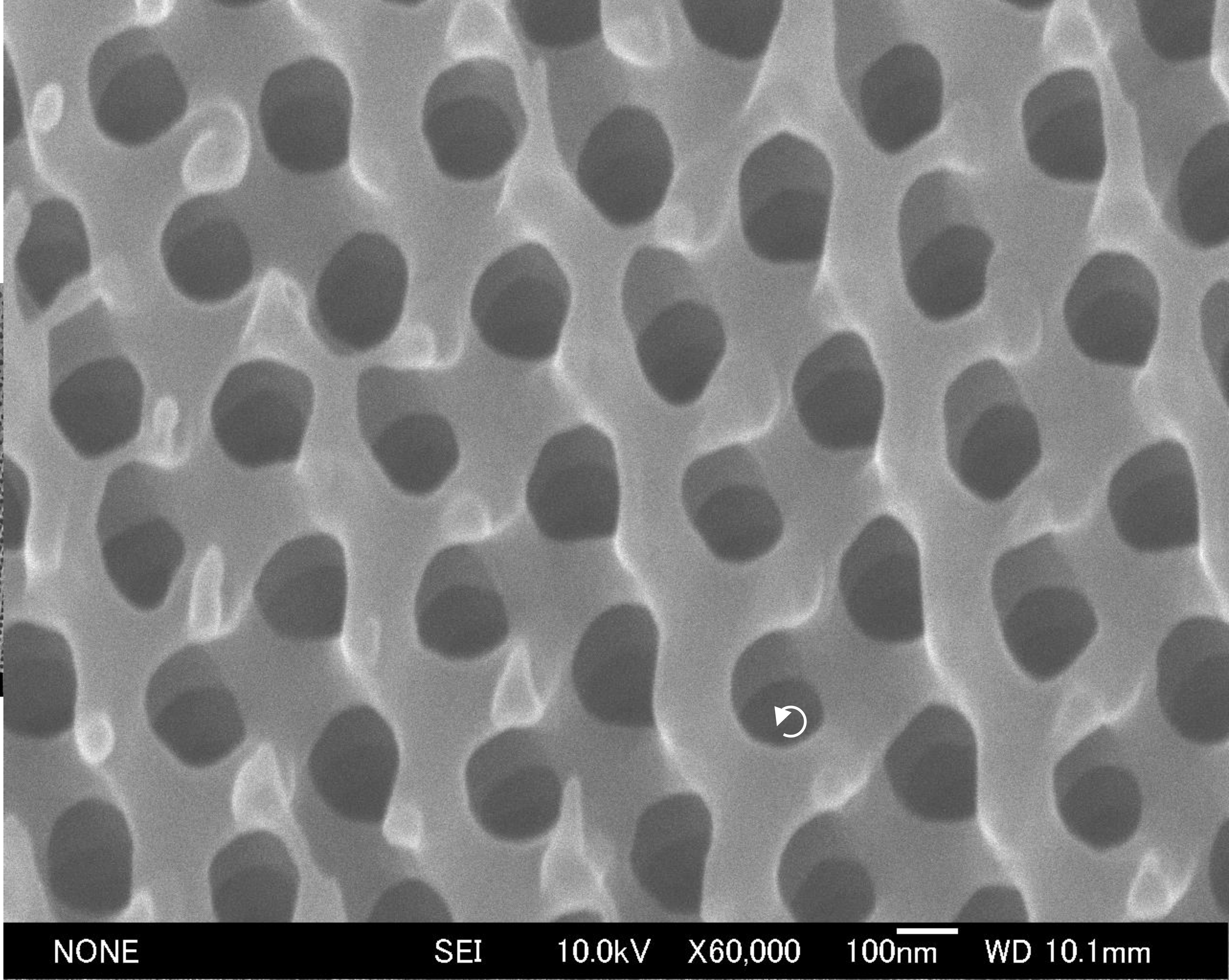

specimen No. 1
scale No. 1
domain No. 13
LH

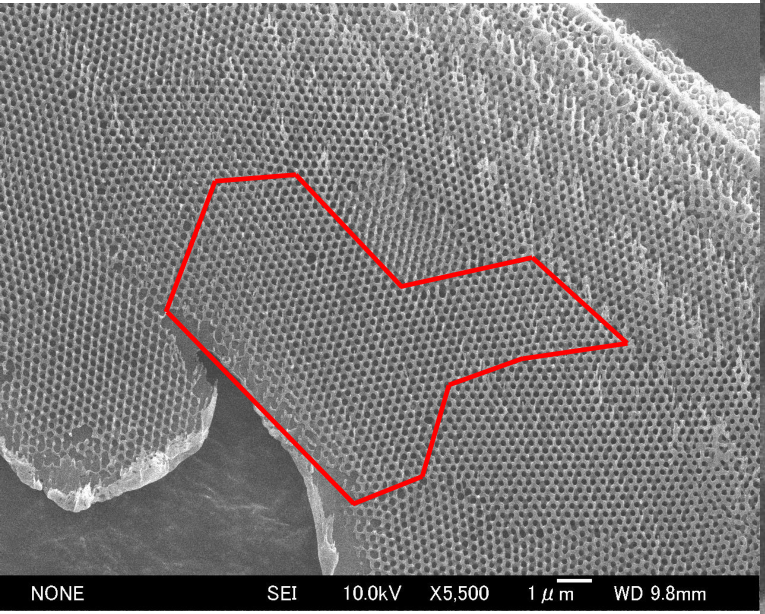

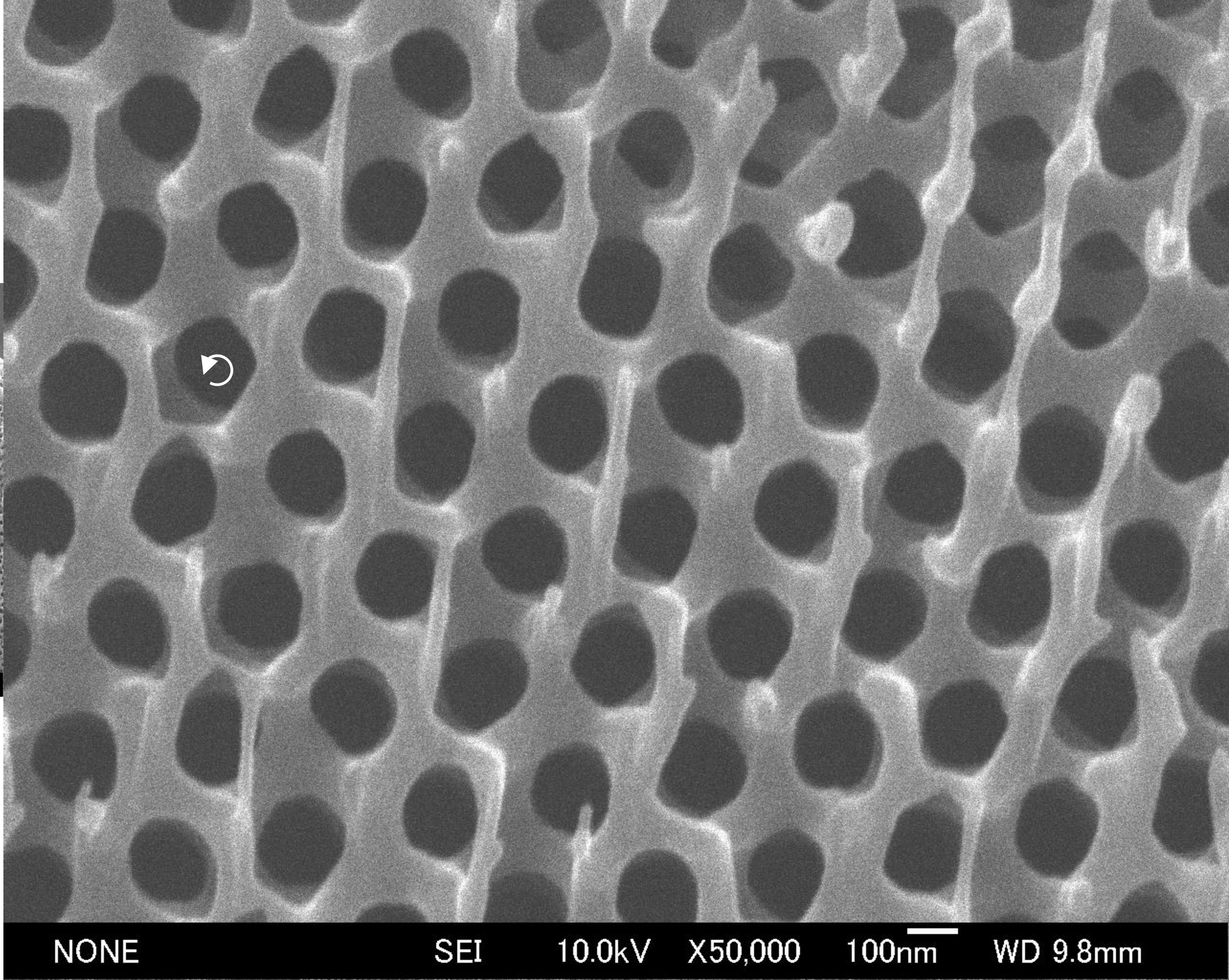

specimen No. 1
scale No. 1
domain No. 14
LH

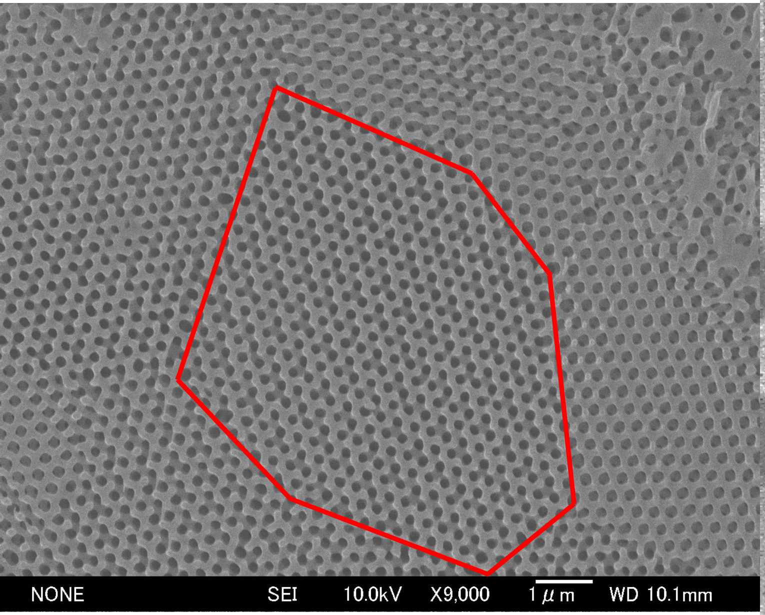

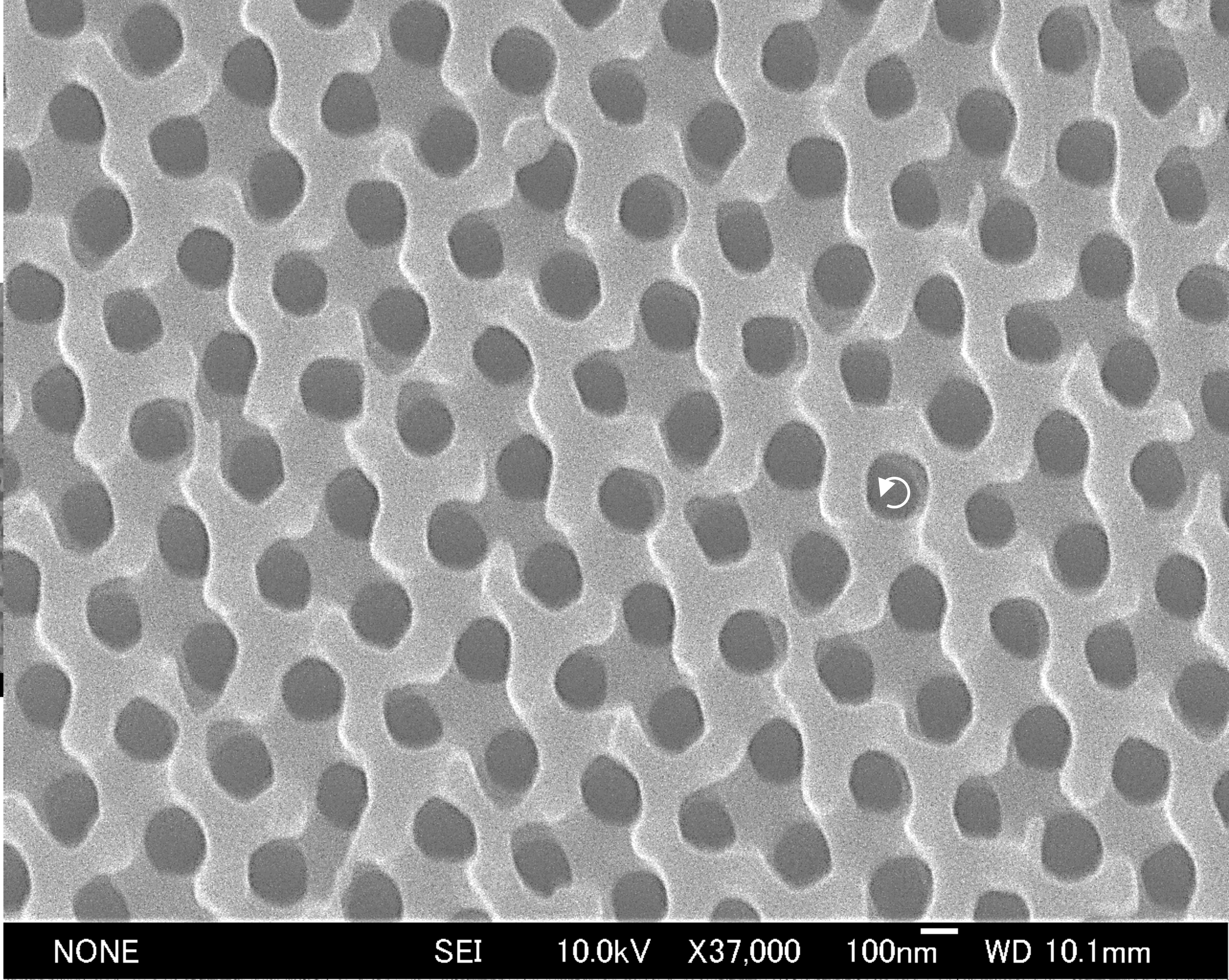

specimen No. 1
scale No. 1
domain No. 15
LH

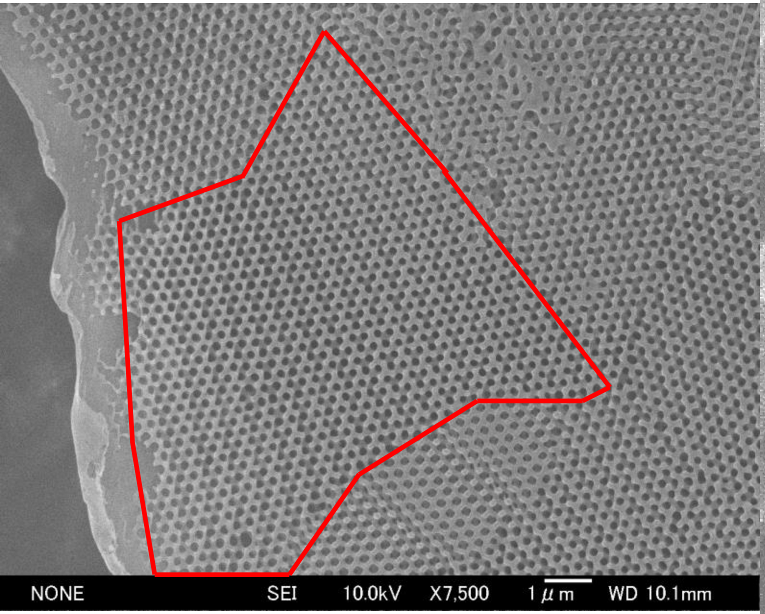

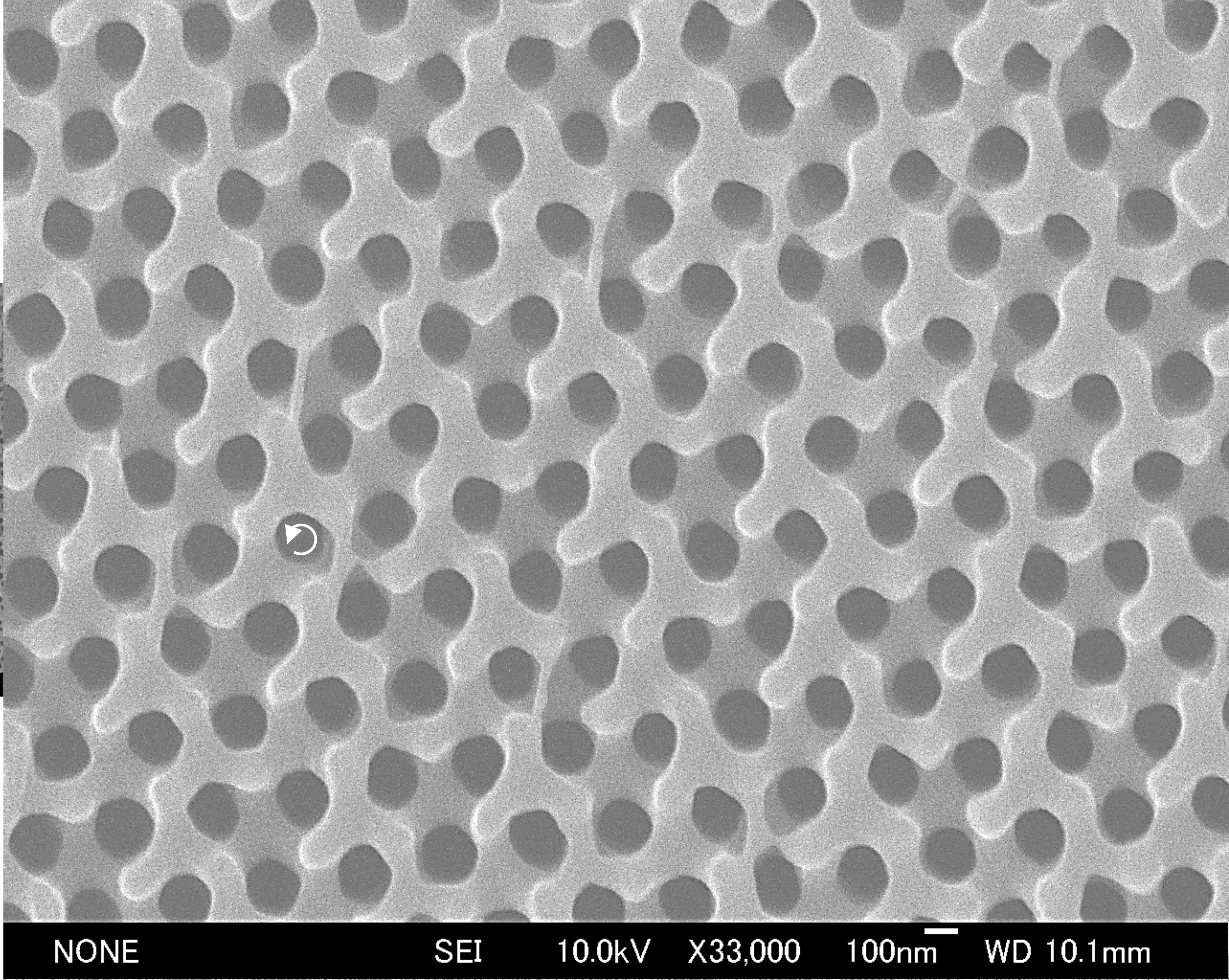

specimen No. 1
scale No. 1
domain No. 16
LH

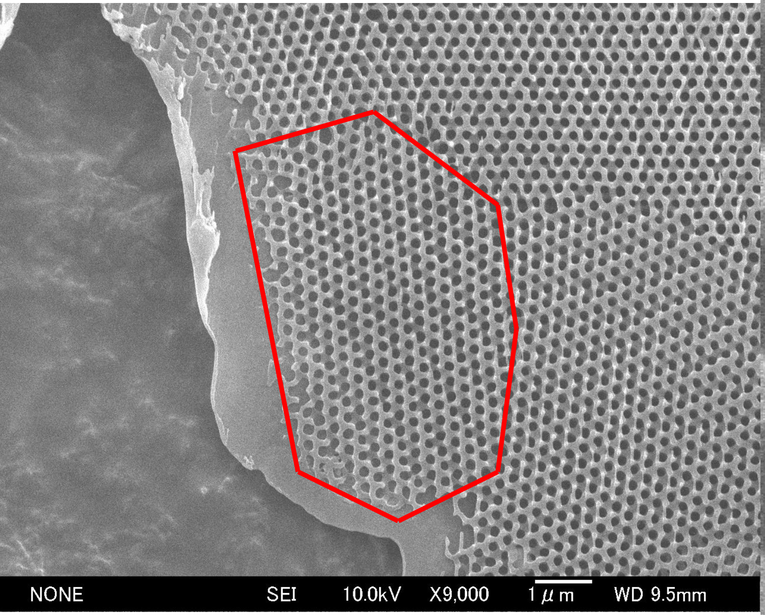

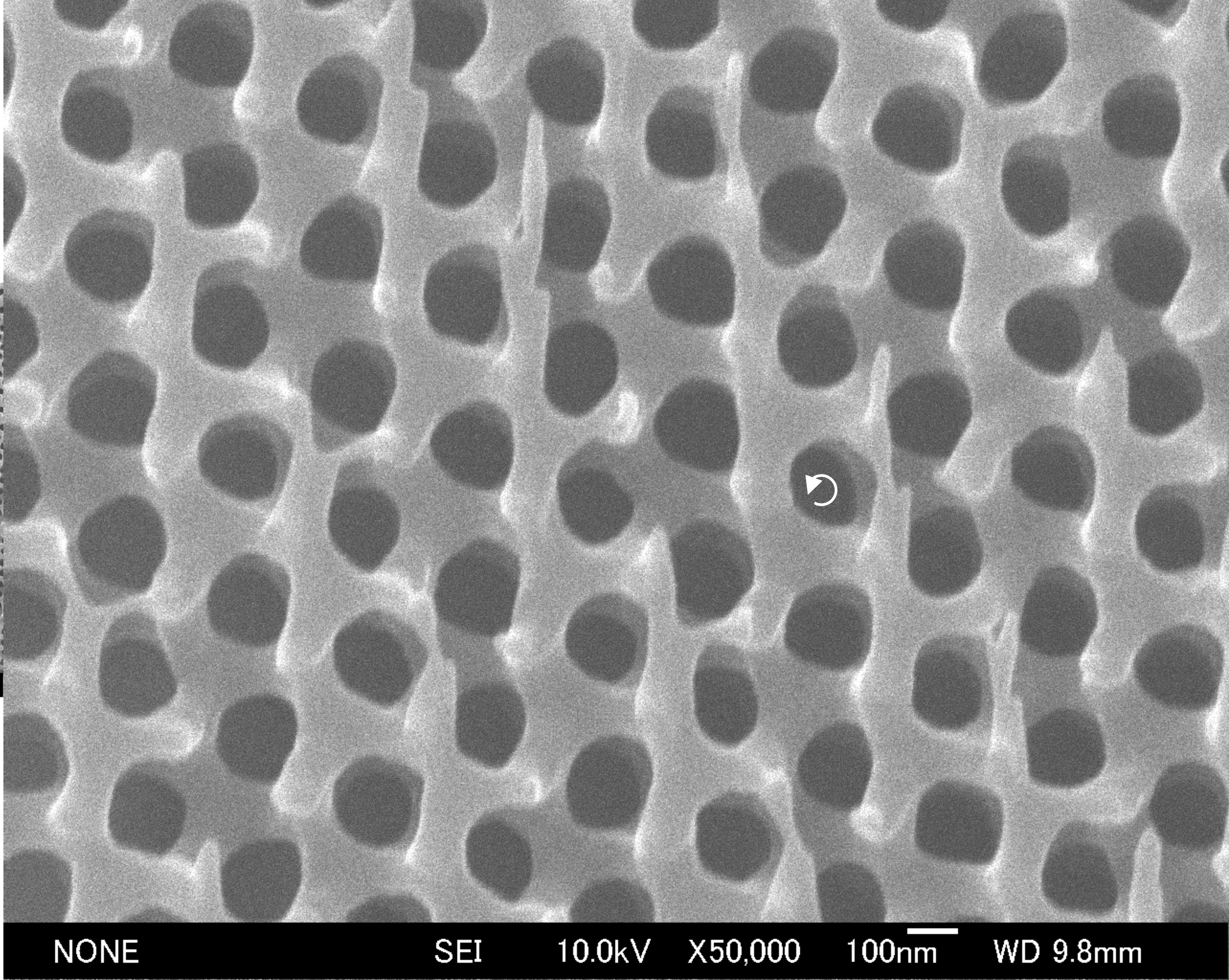

specimen No. 1
scale No. 1
domain No. 17
LH

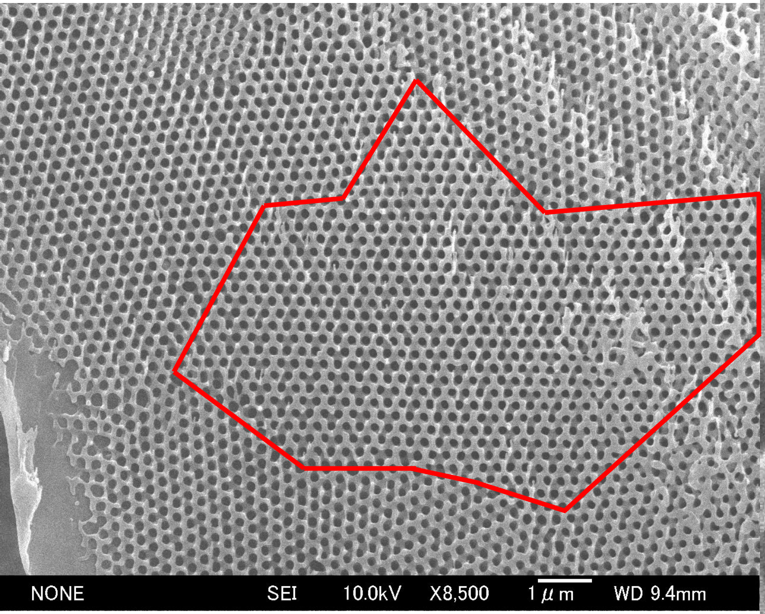

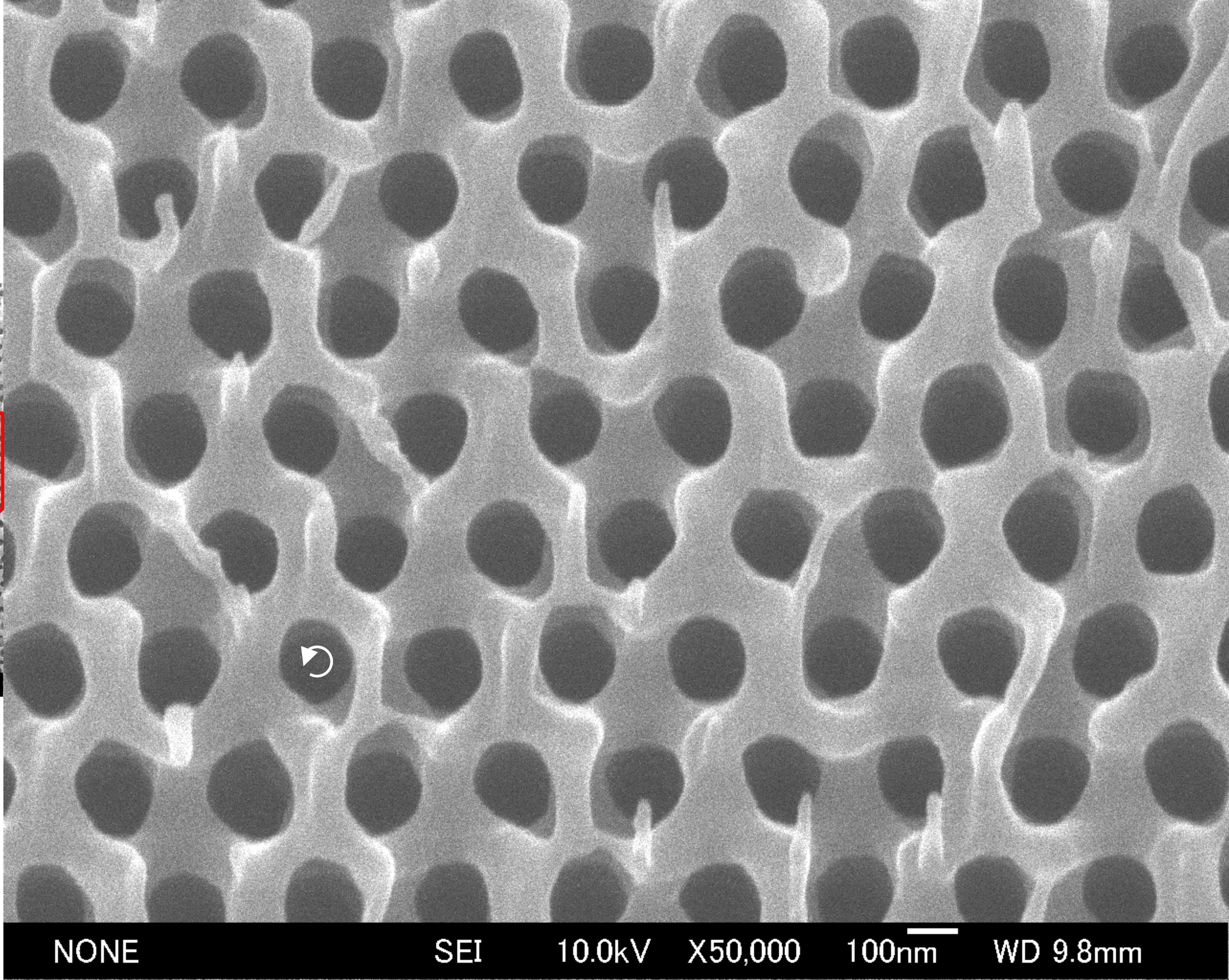

specimen No. 1
scale No. 1
domain No. 18
LH

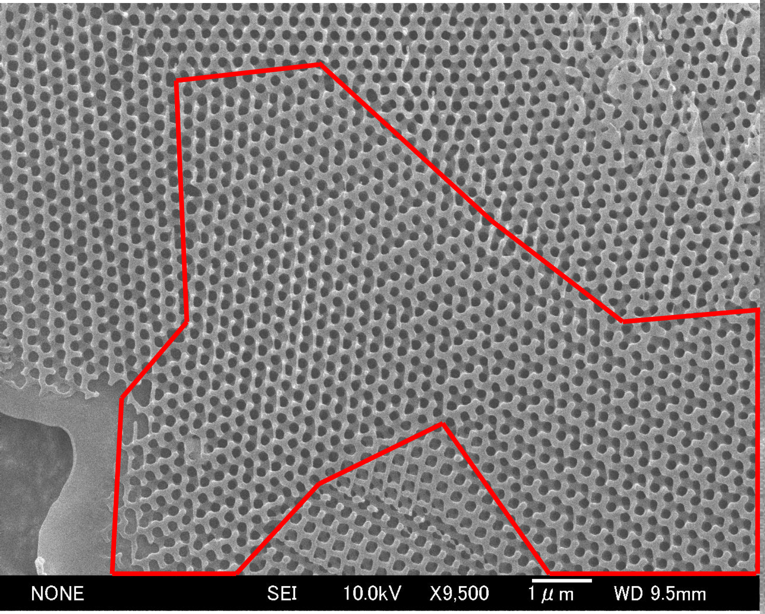

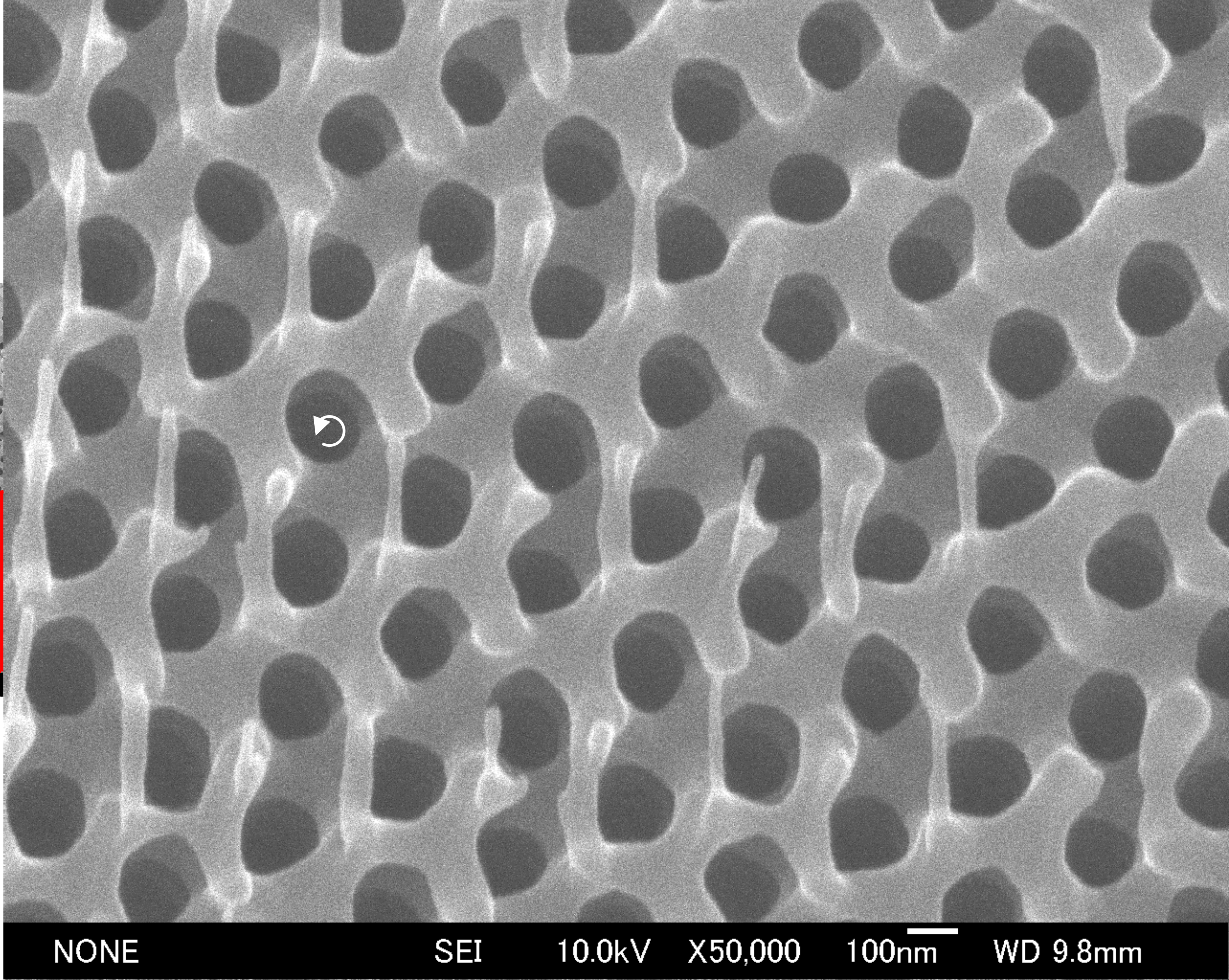

specimen No. 1
scale No. 1
domain No. 19
LH

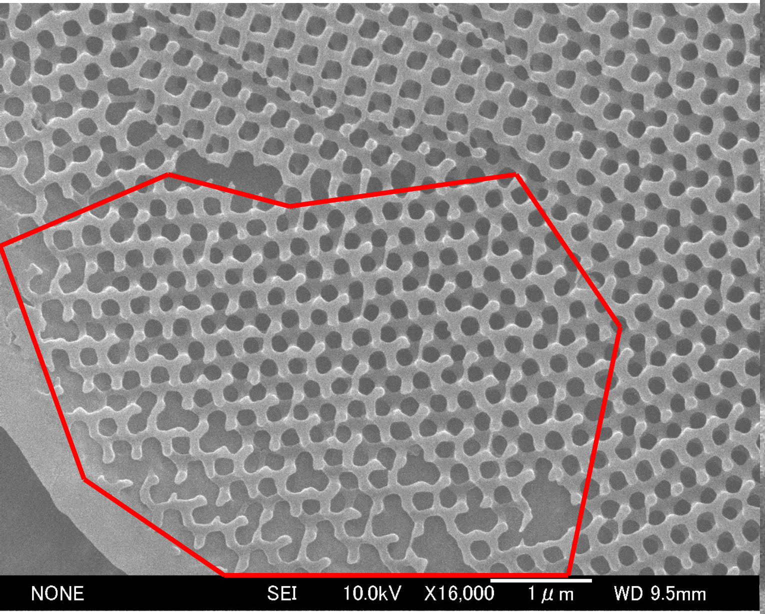

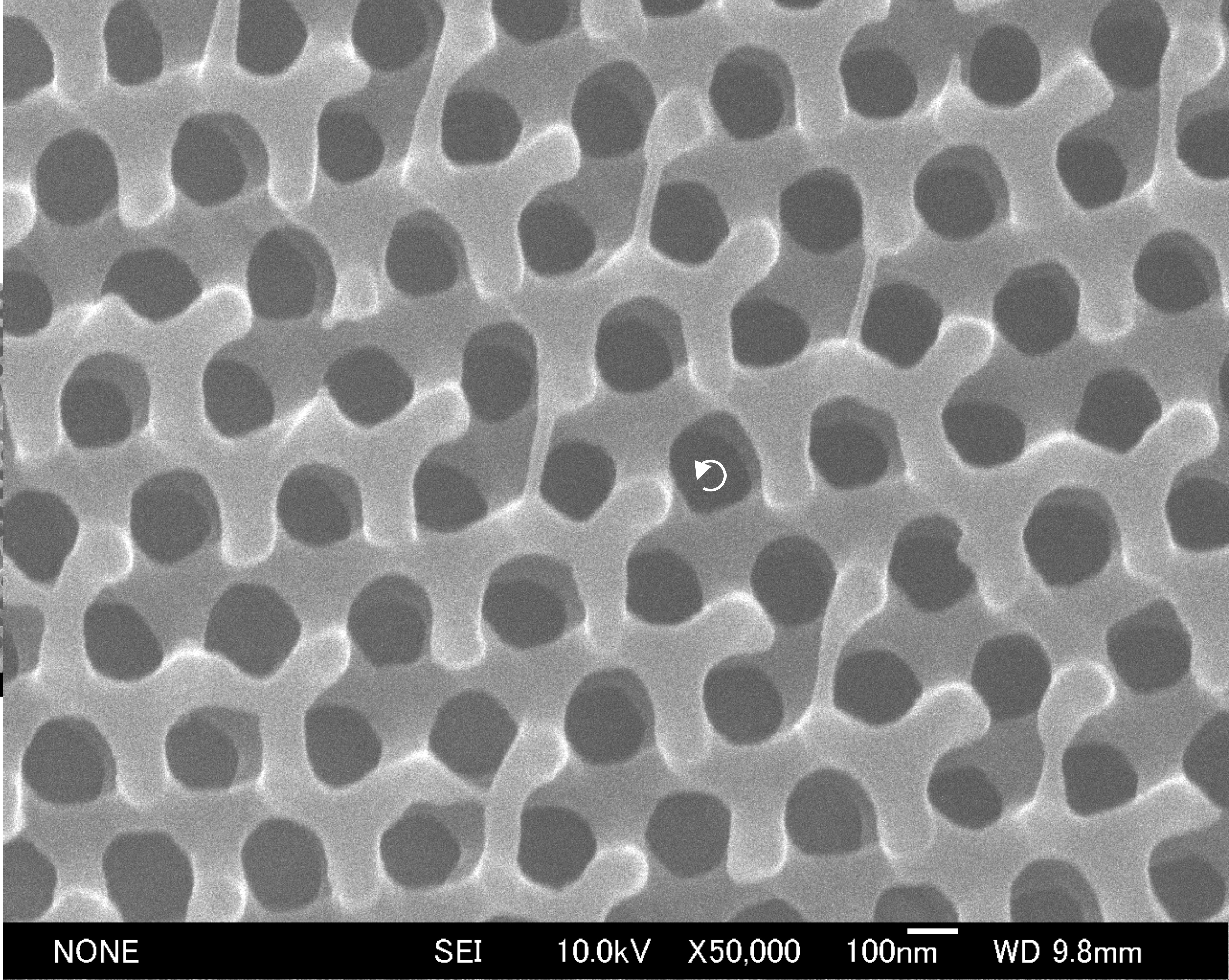

specimen No. 1
scale No. 1
domain No. 20
LH

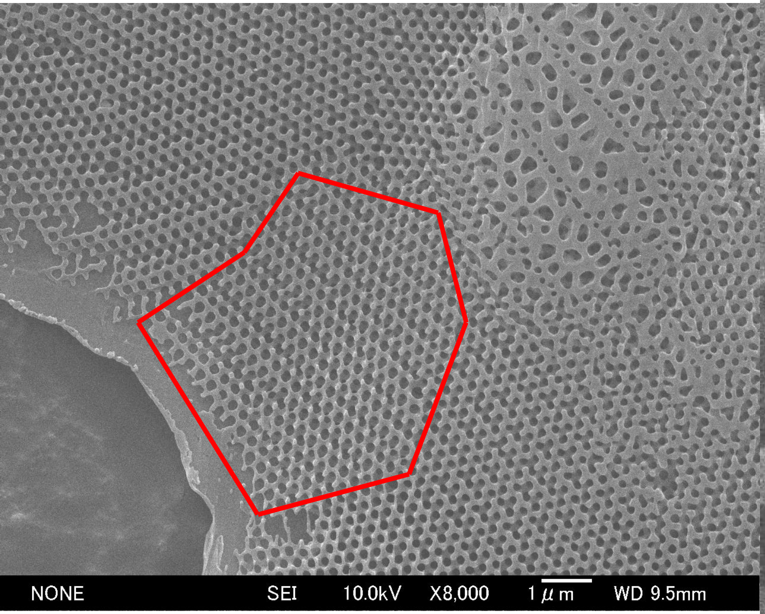

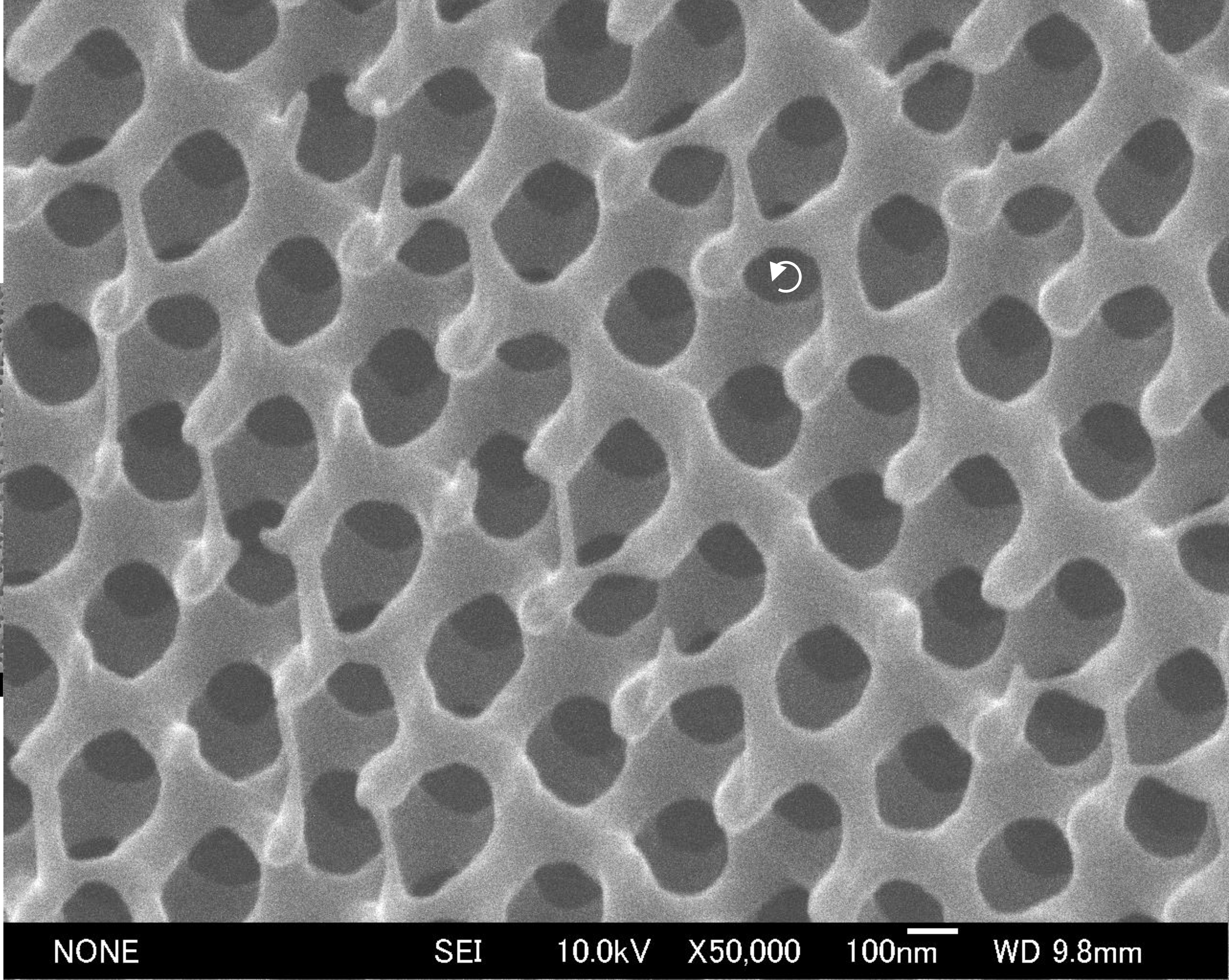

specimen No. 1
scale No. 1
domain No. 21
LH

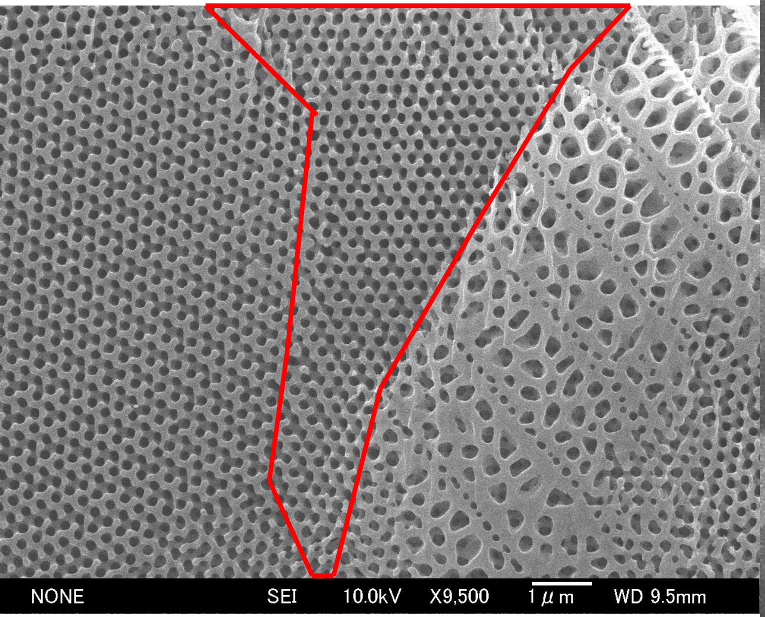

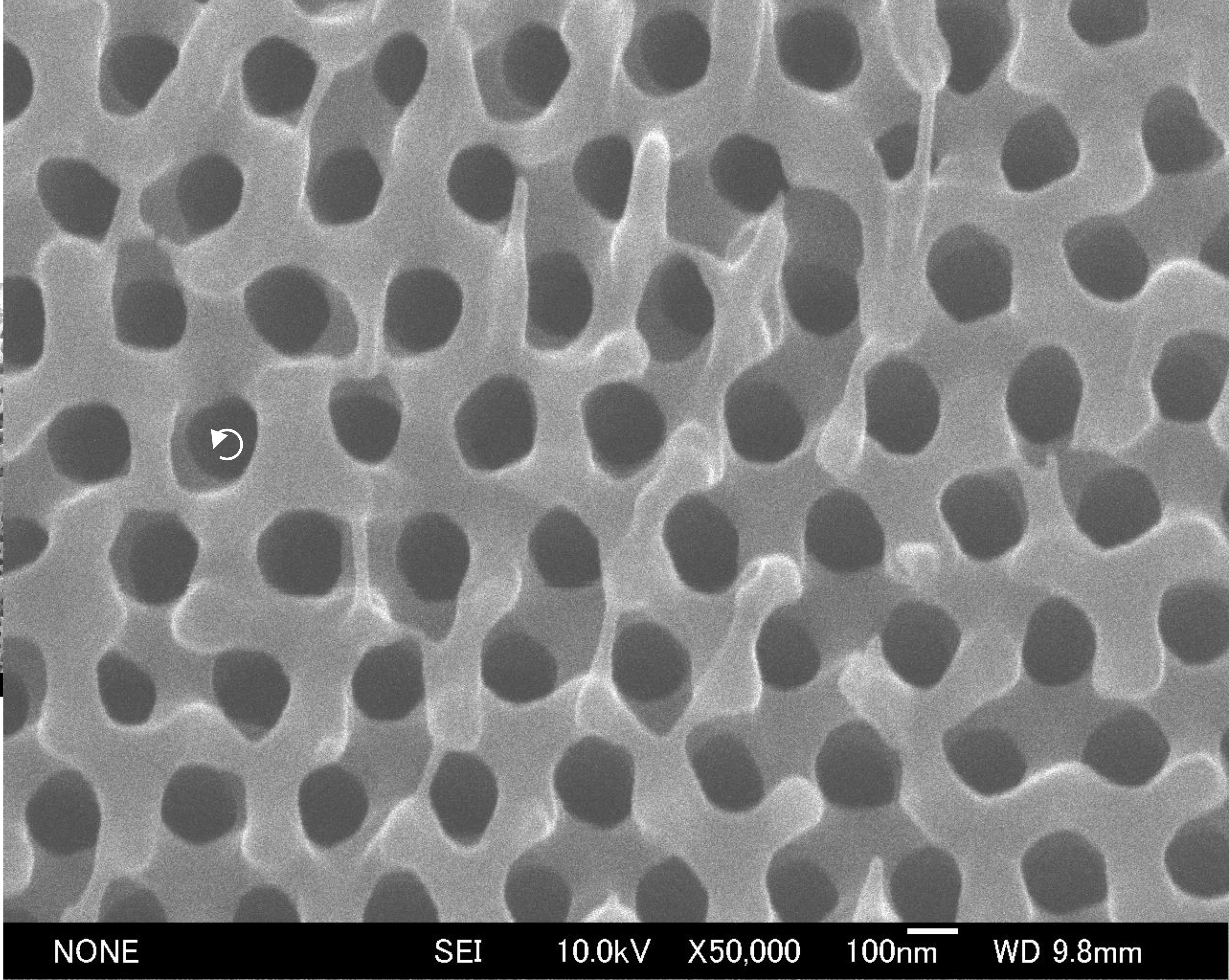

specimen No. 1
scale No. 1
domain No. 22
LH

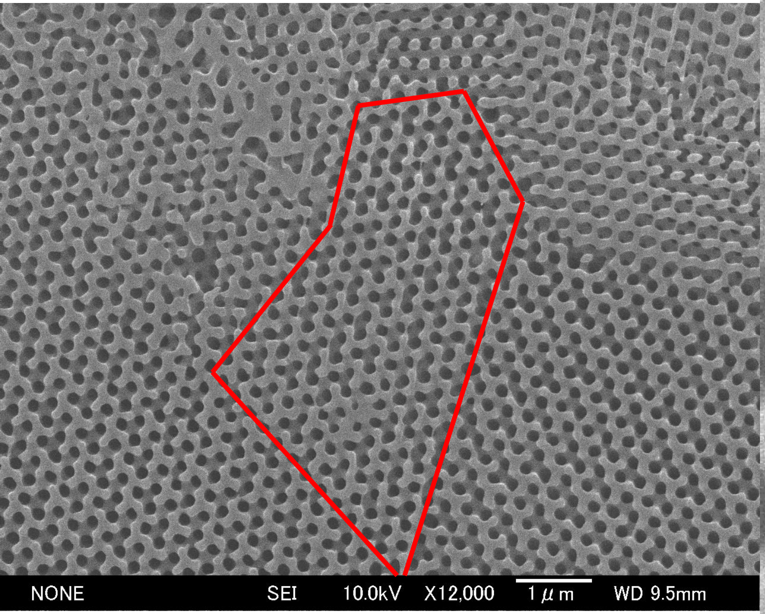

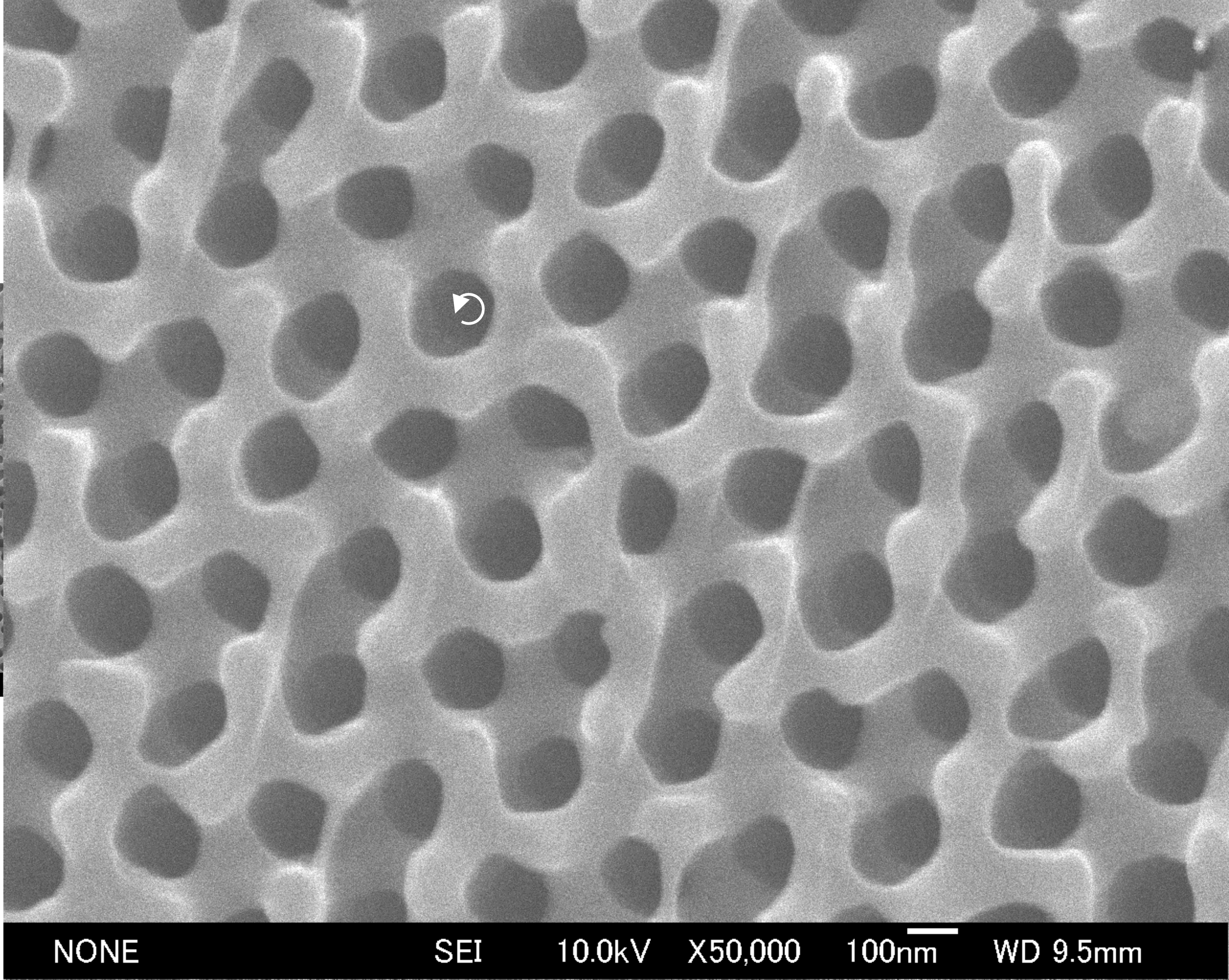

specimen No. 1
scale No. 1
domain No. 23
LH

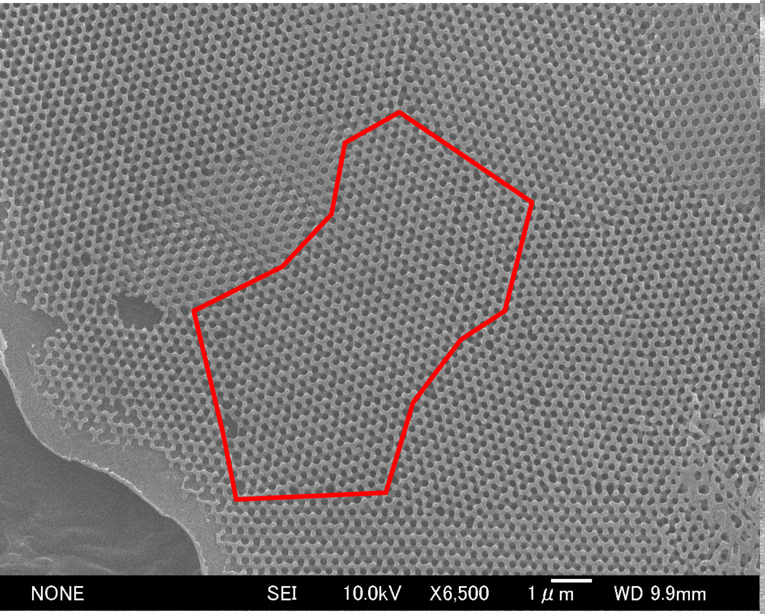

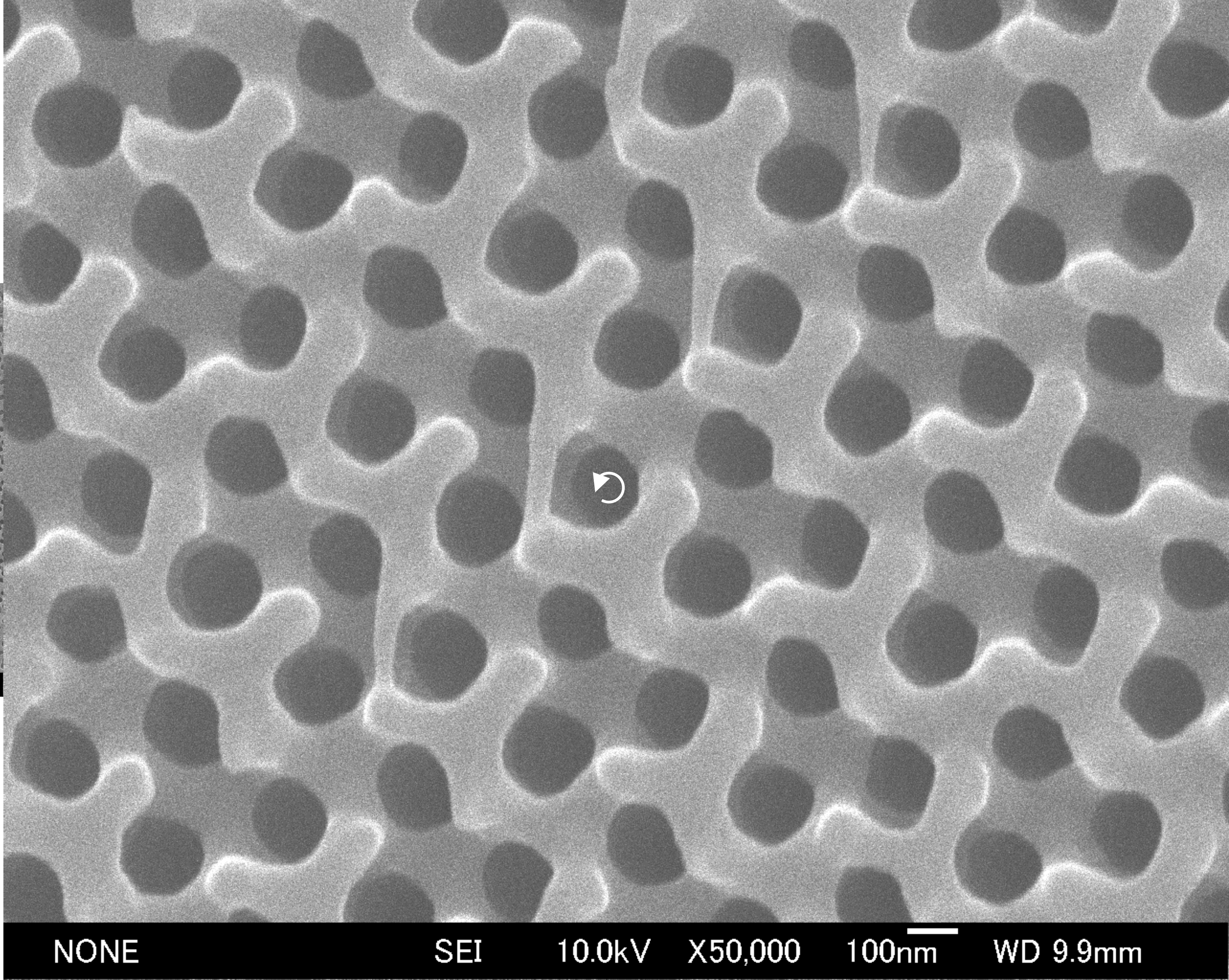

specimen No. 1
scale No. 1
domain No. 24
LH

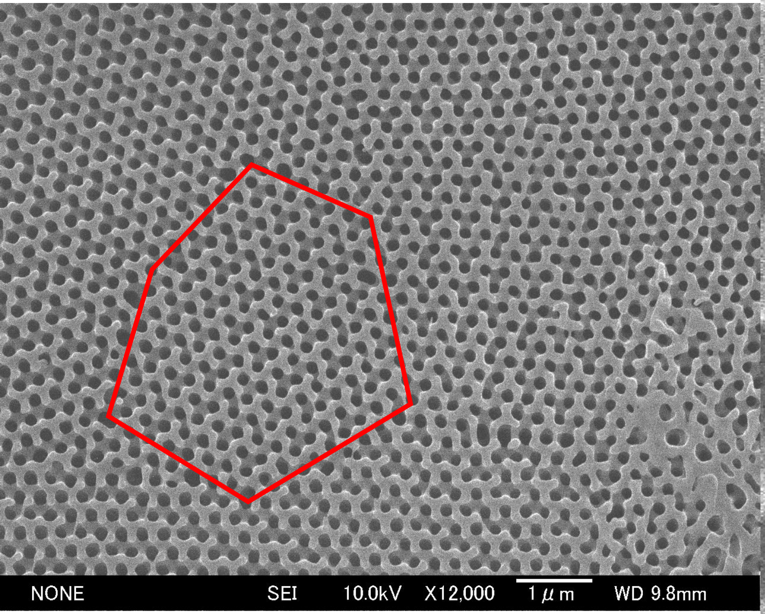

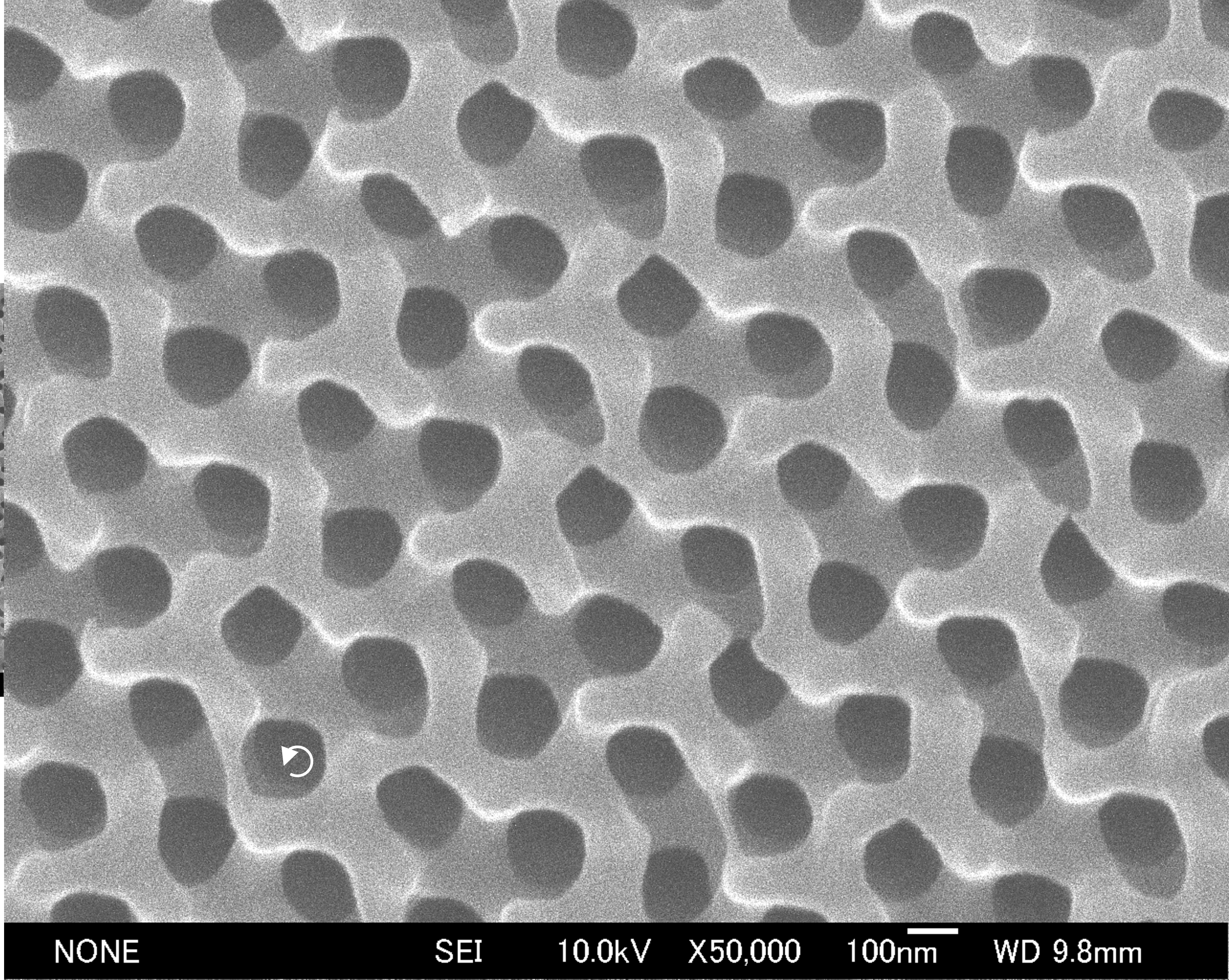

specimen No. 1
scale No. 1
domain No. 25
LH

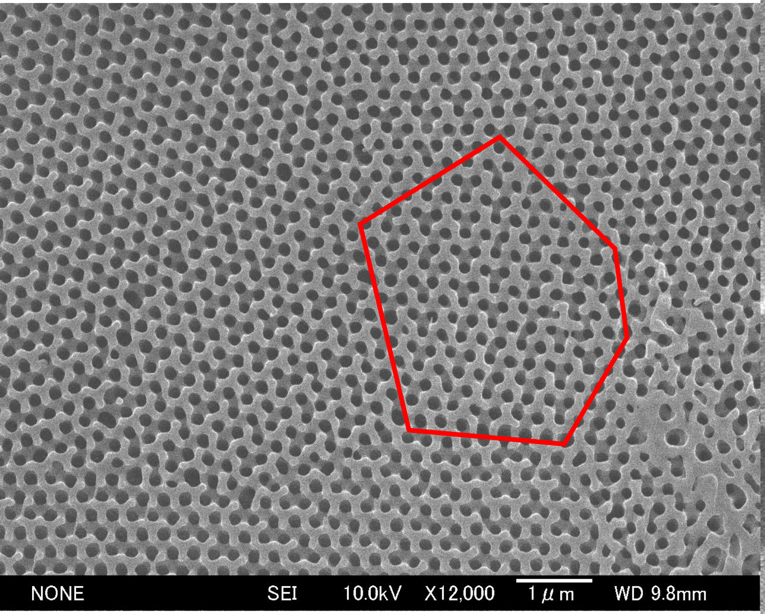

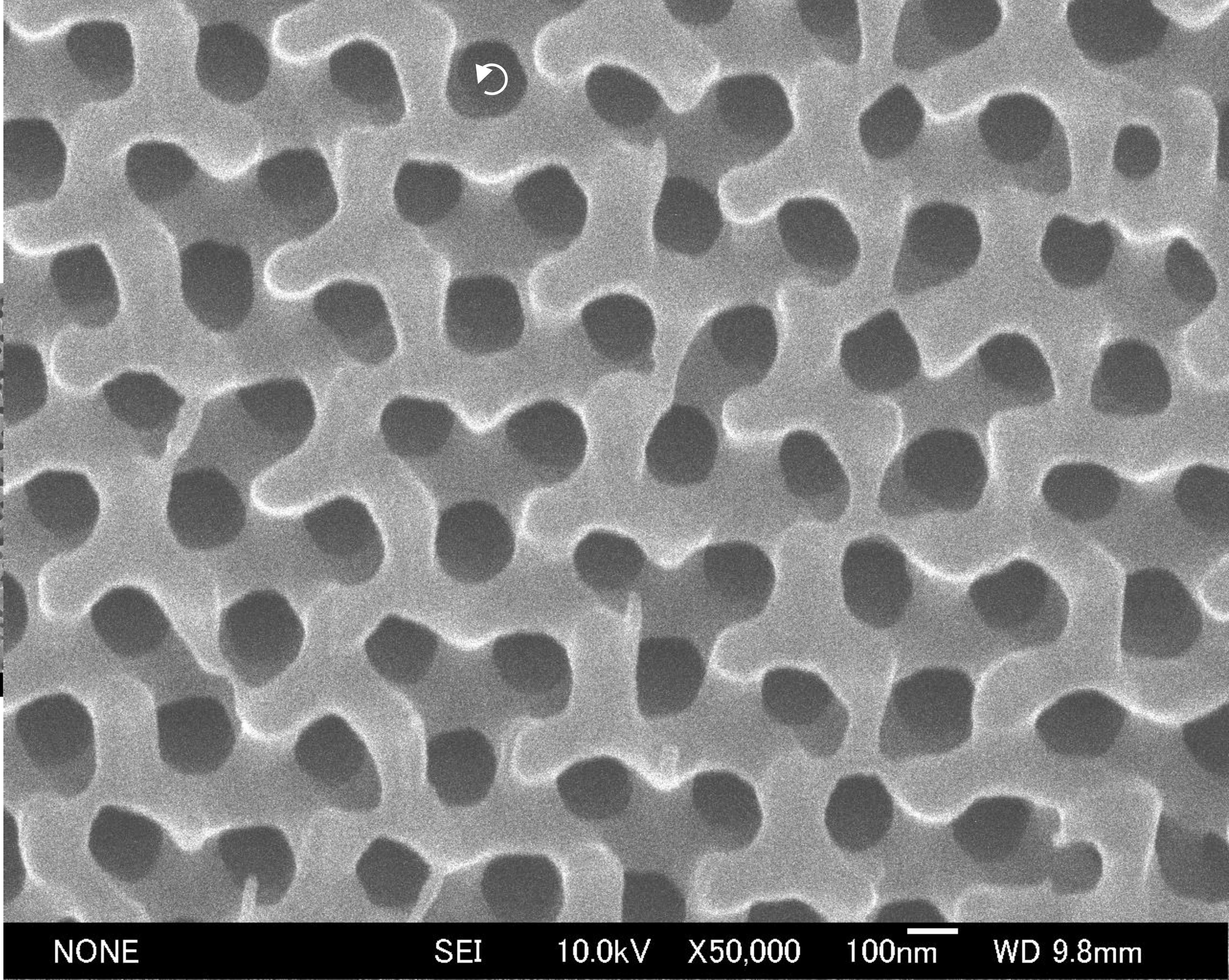

specimen No. 1
scale No. 1
domain No. 26
LH

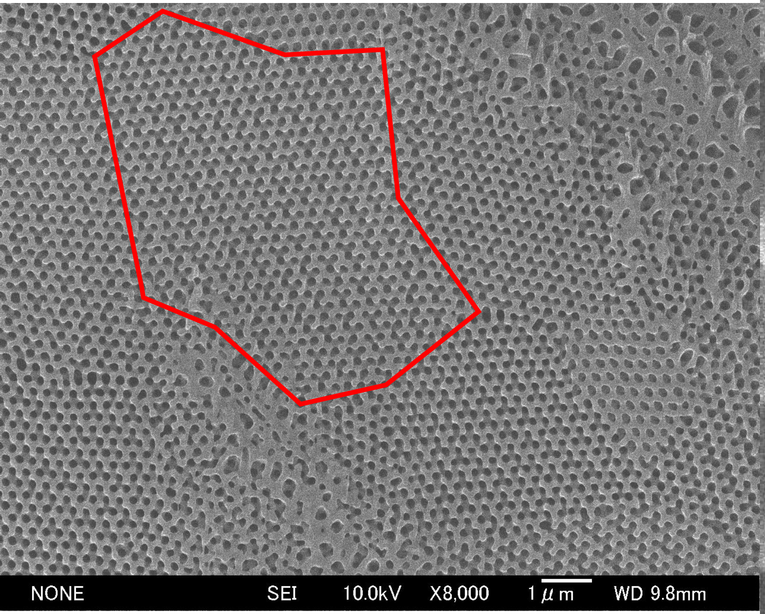

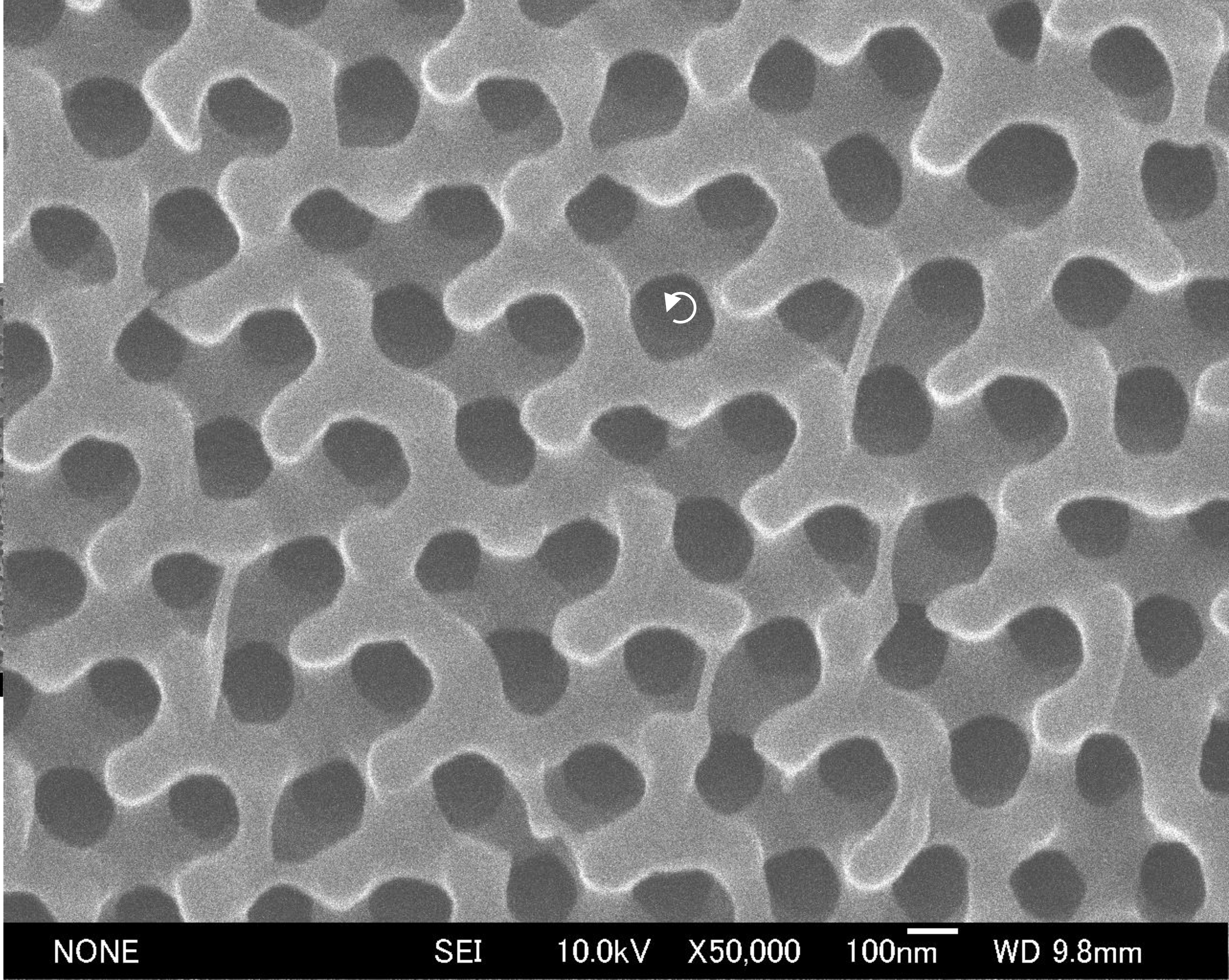

specimen No. 1
scale No. 1
domain No. 27
LH

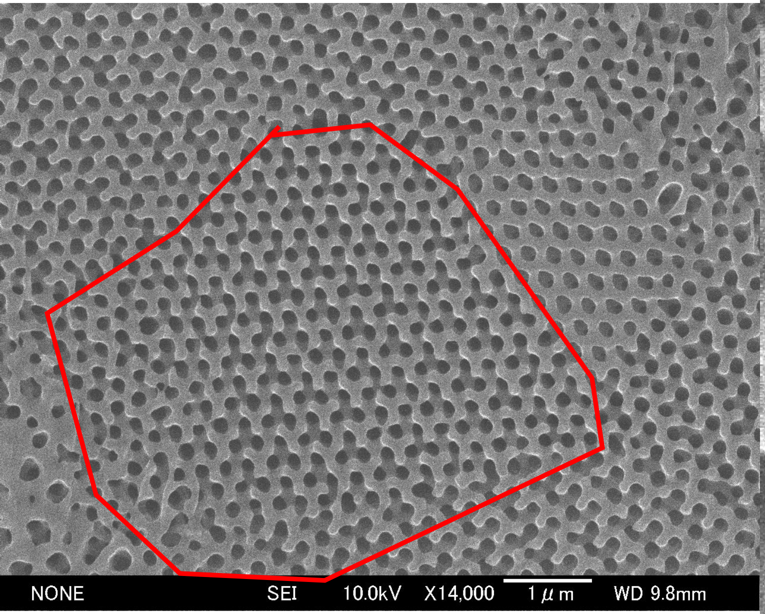

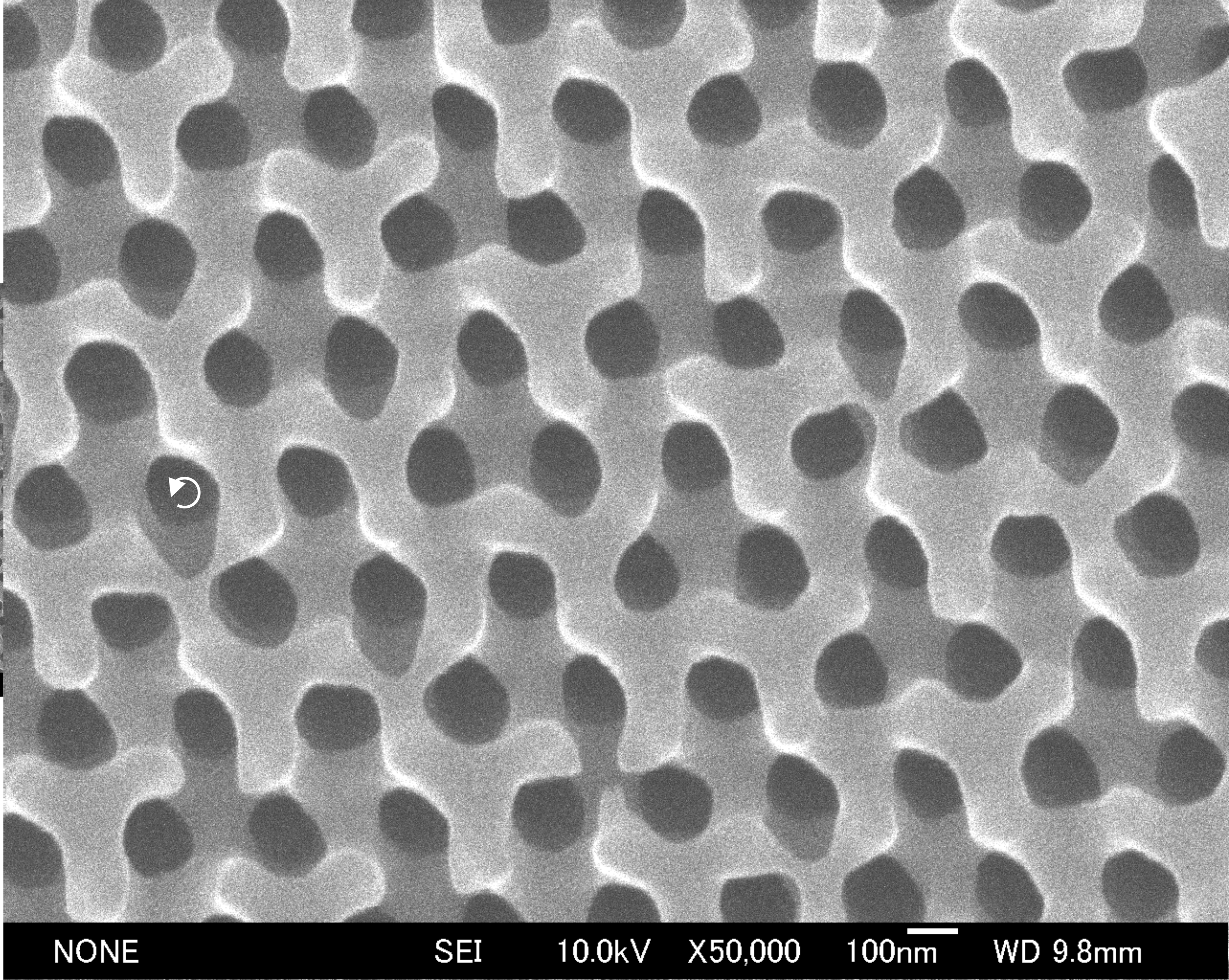

specimen No. 1
scale No. 1
domain No. 28
LH

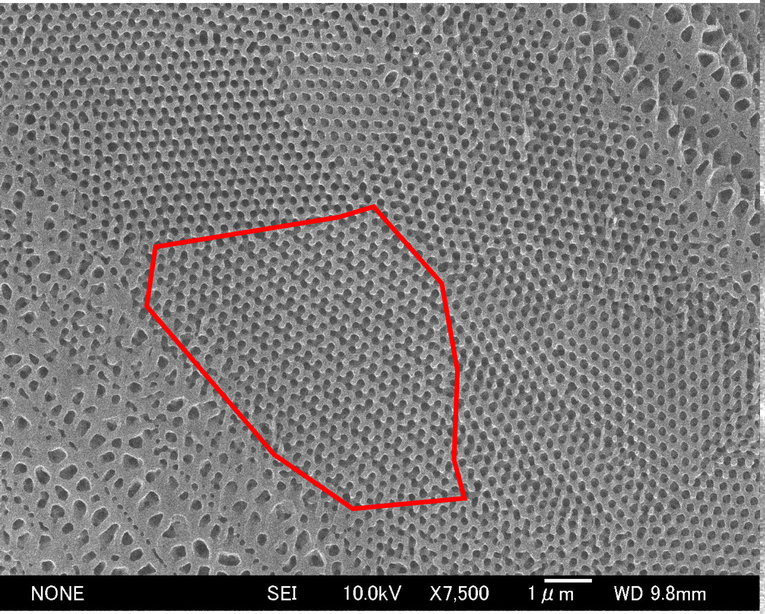

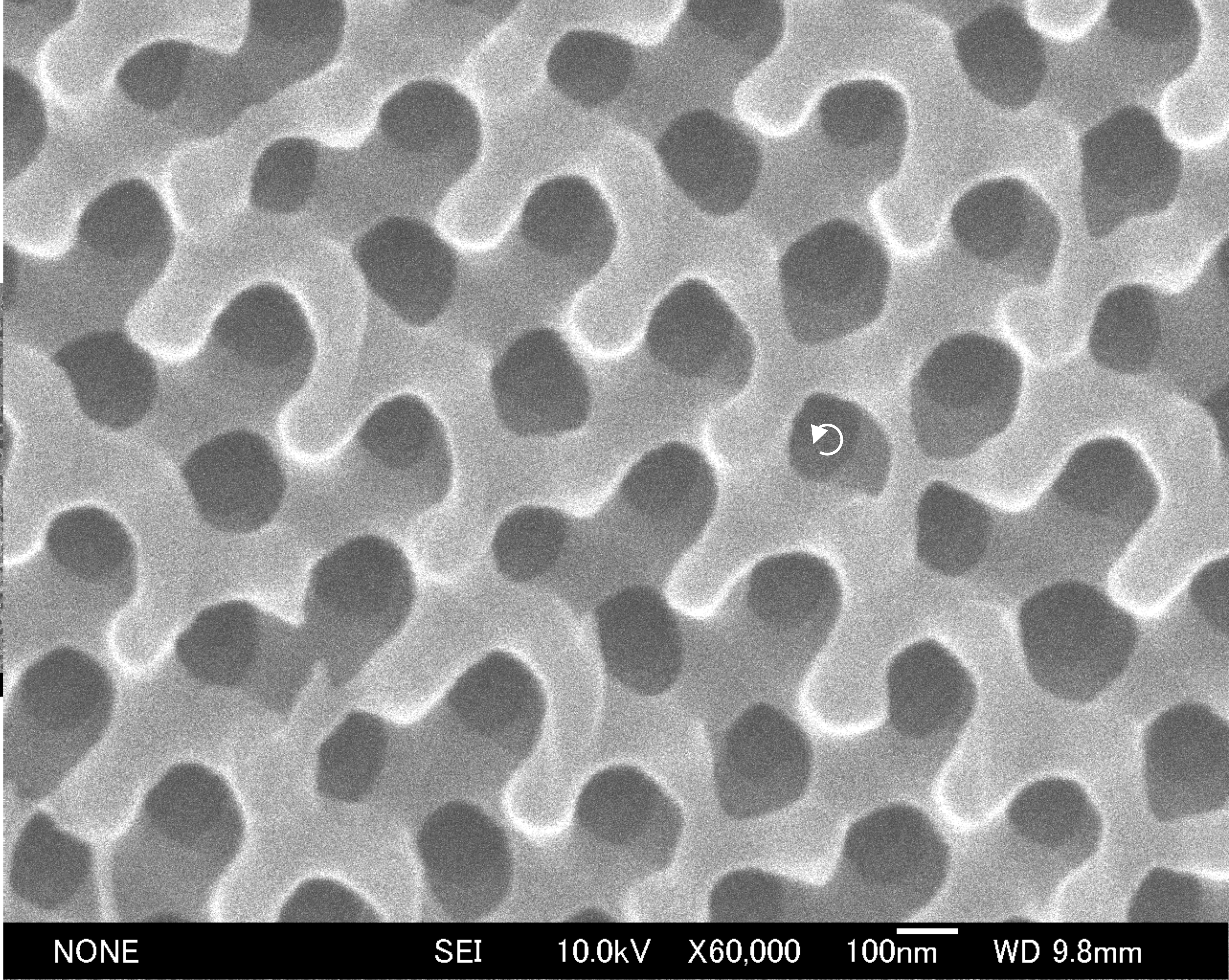

specimen No. 1
scale No. 1
domain No. 29
LH

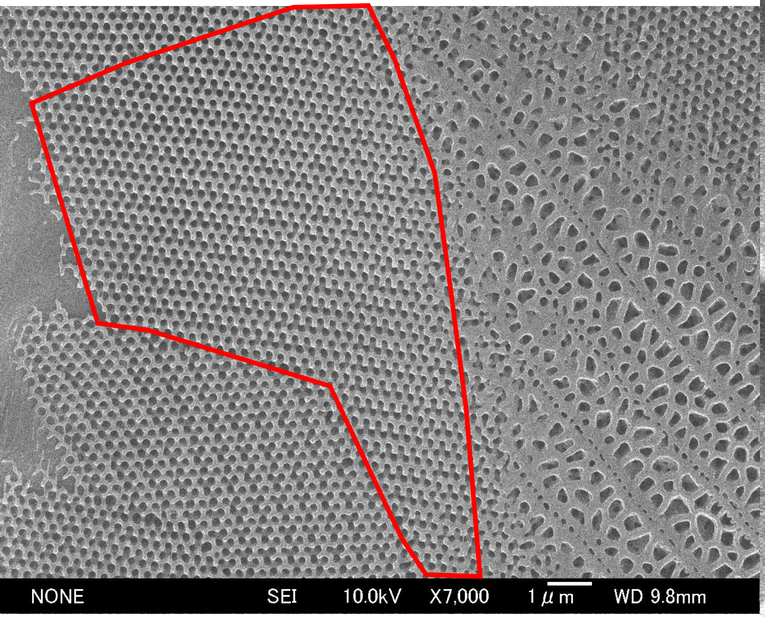

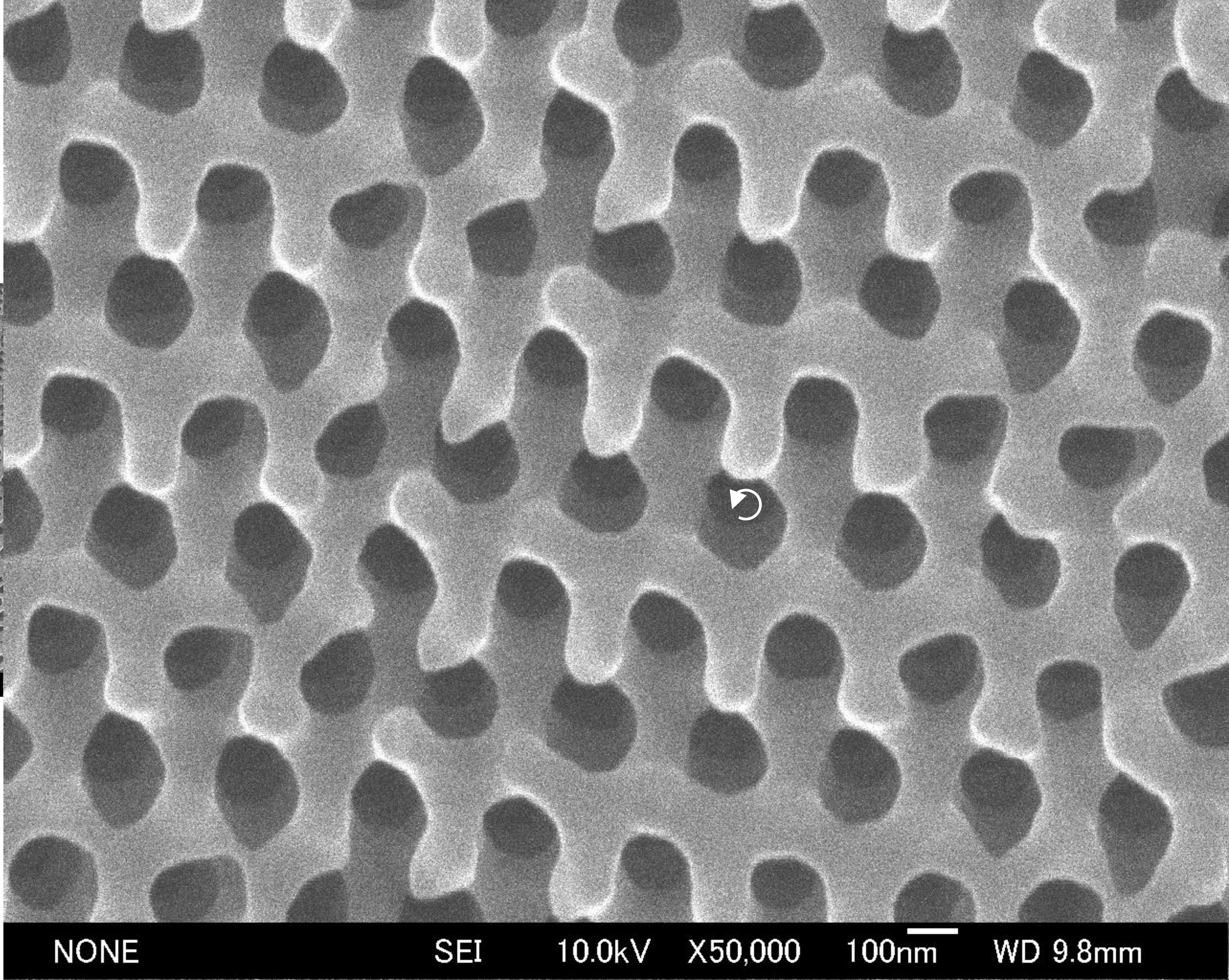

specimen No. 1
scale No. 1
domain No. 30
LH

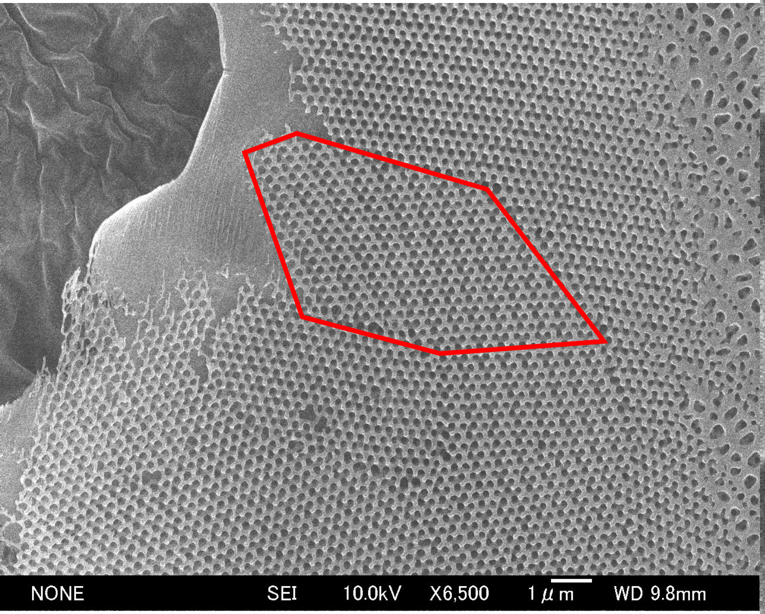

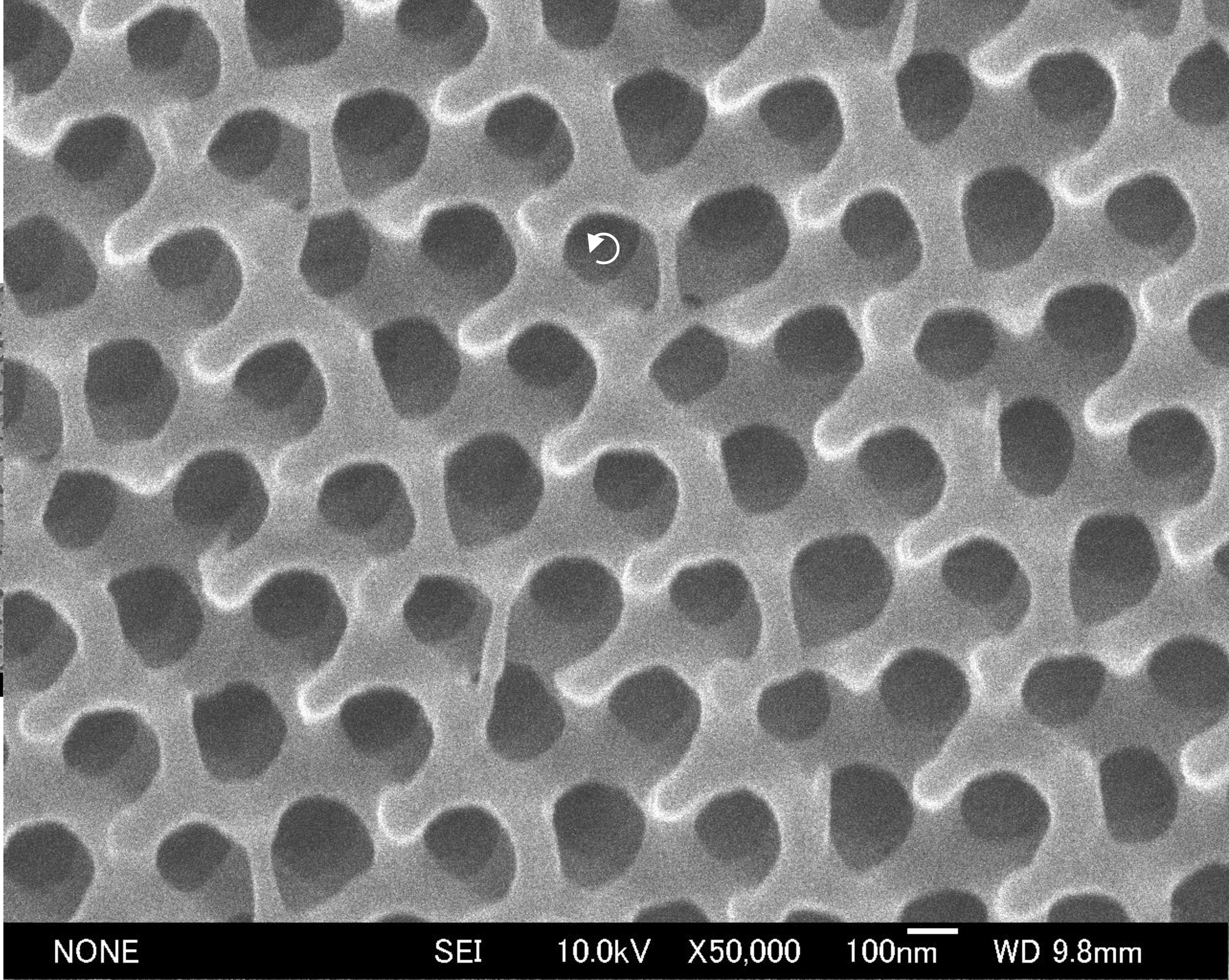

specimen No. 1
scale No. 1
domain No. 31
LH

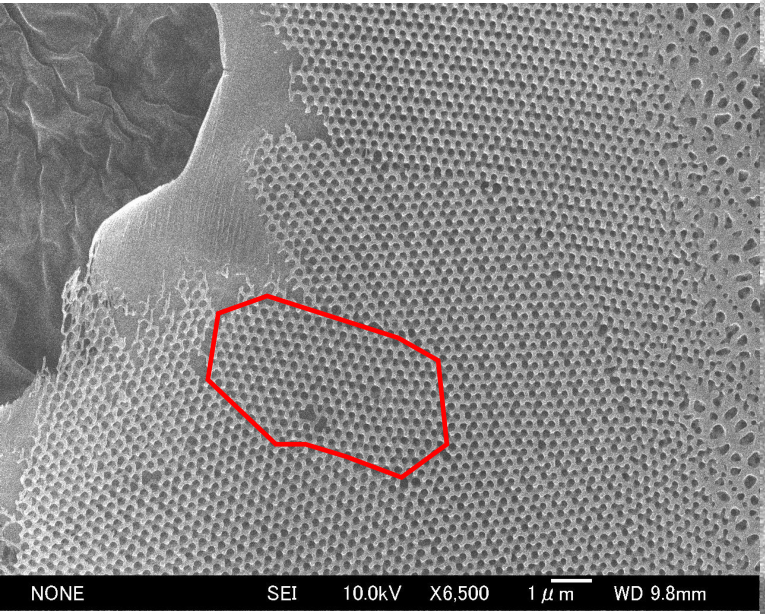

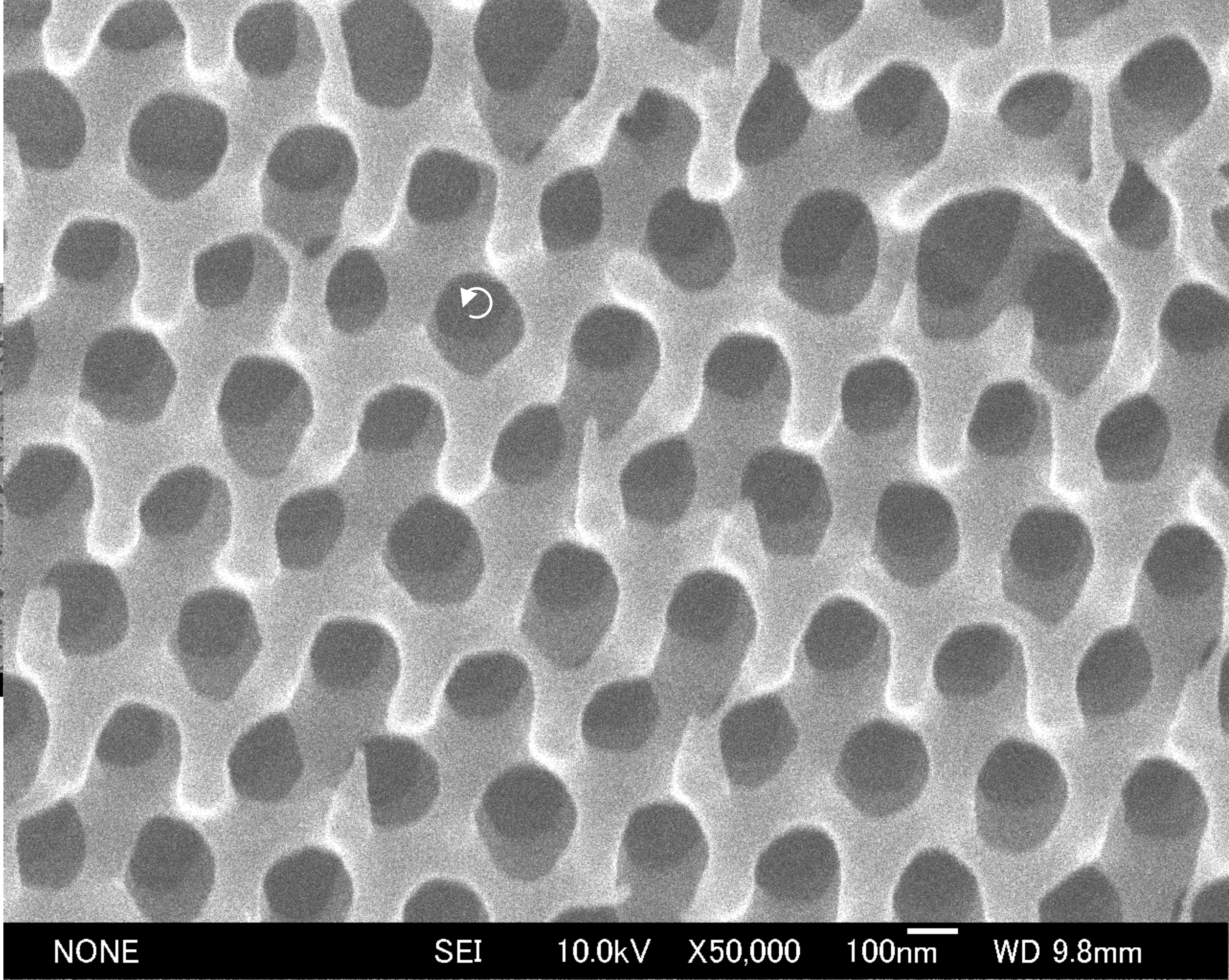

specimen No. 1
scale No. 1
domain No. 32
LH

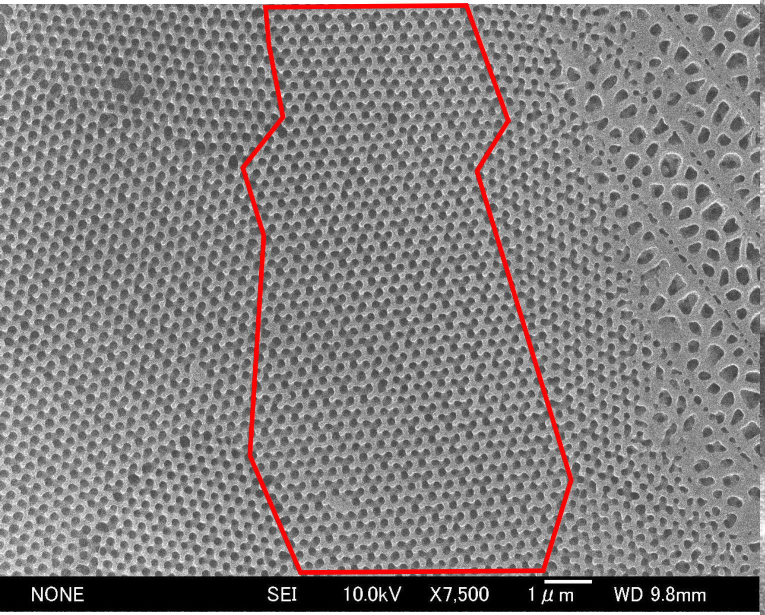

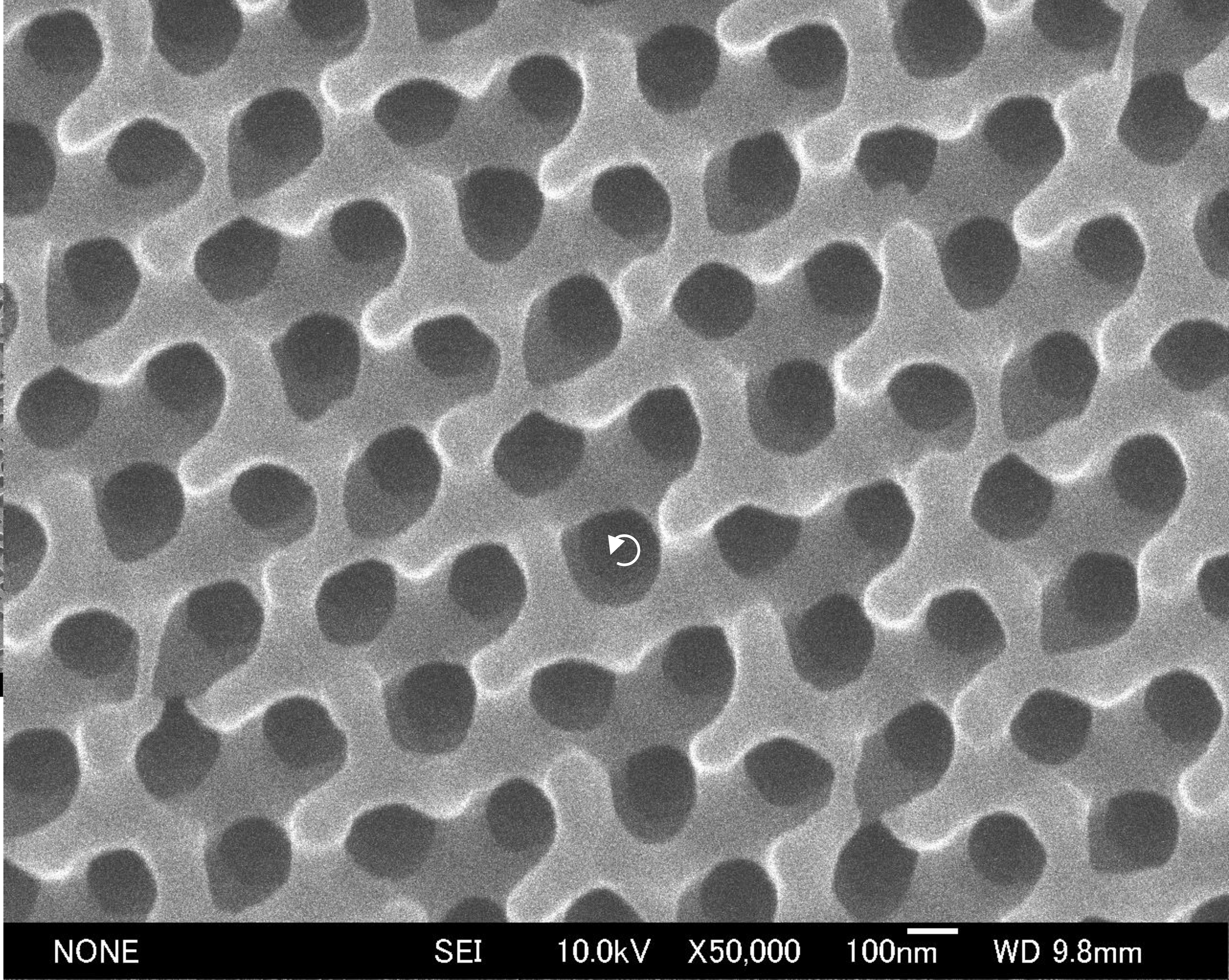

specimen No. 1
scale No. 1
domain No. 33
LH

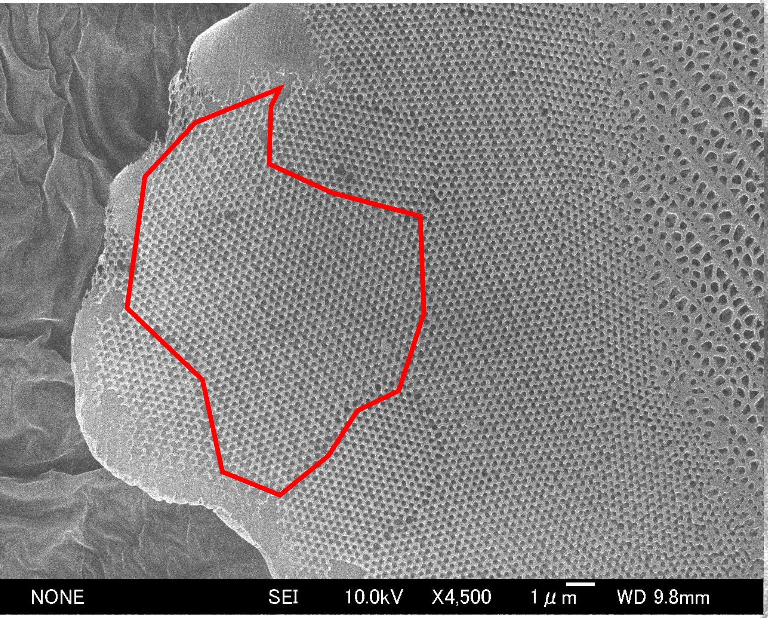

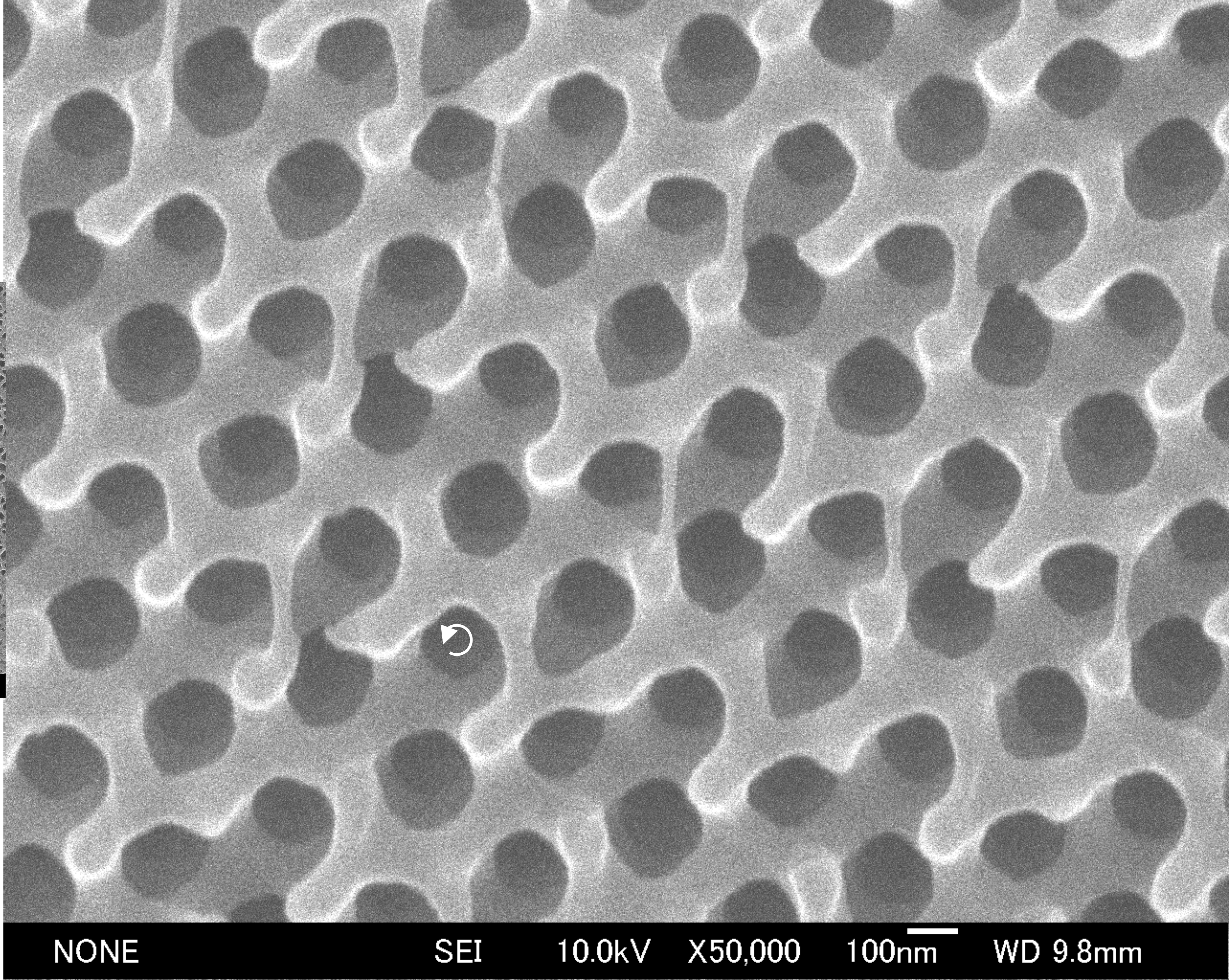

specimen No. 1
scale No. 1
domain No. 34
LH

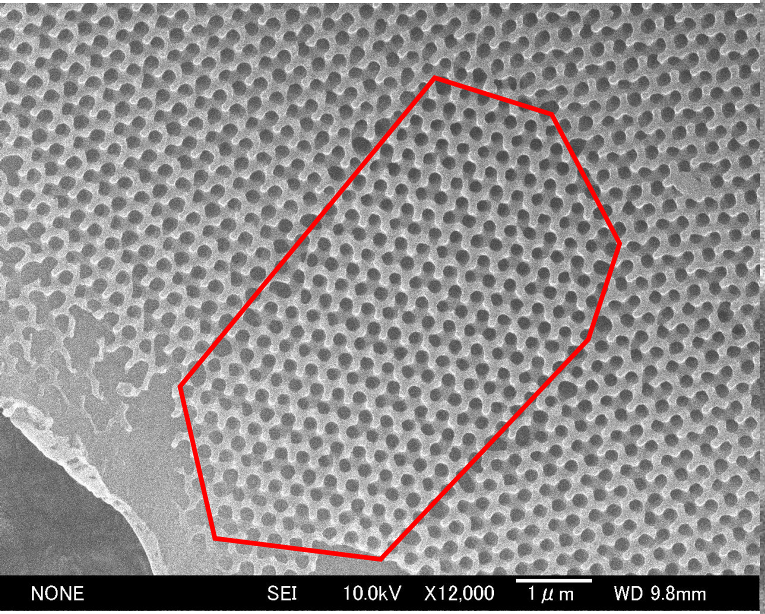

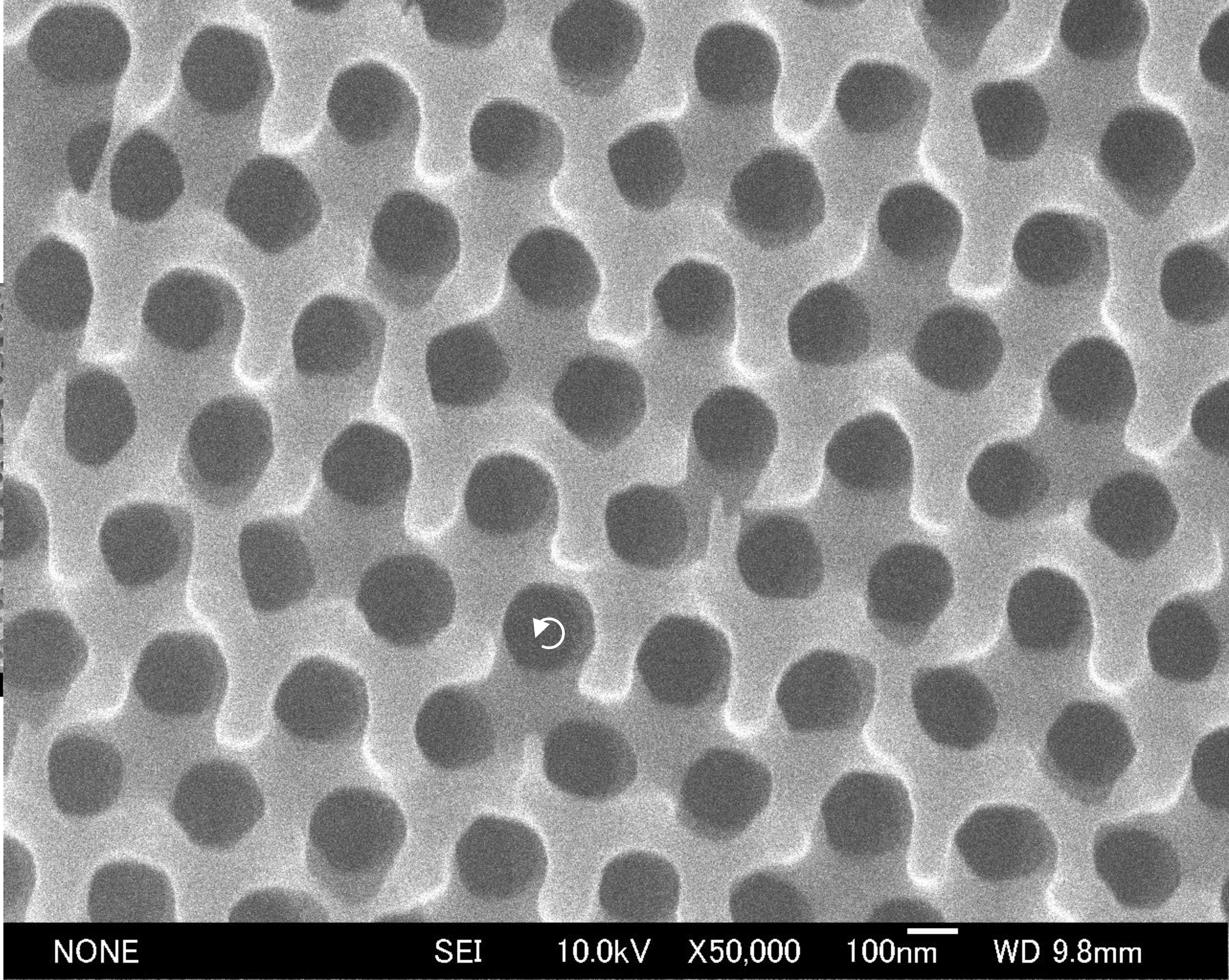

specimen No. 1
scale No. 1
domain No. 35
LH

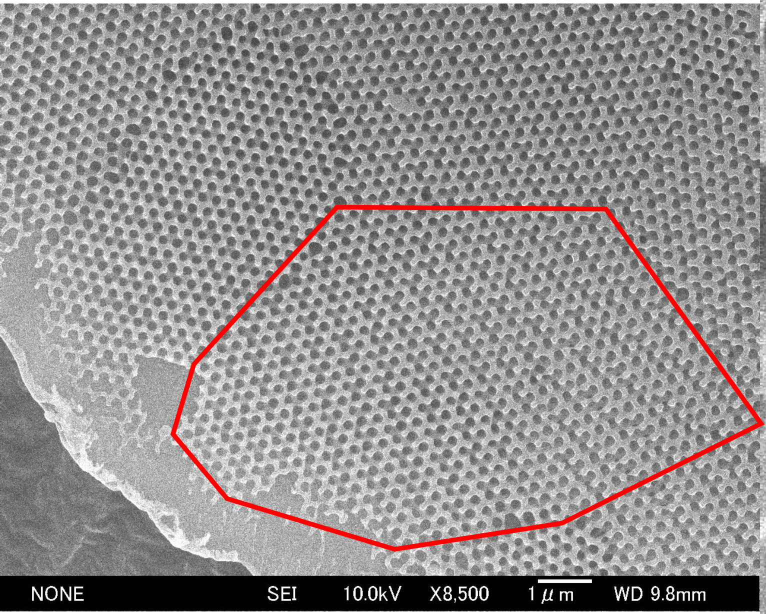

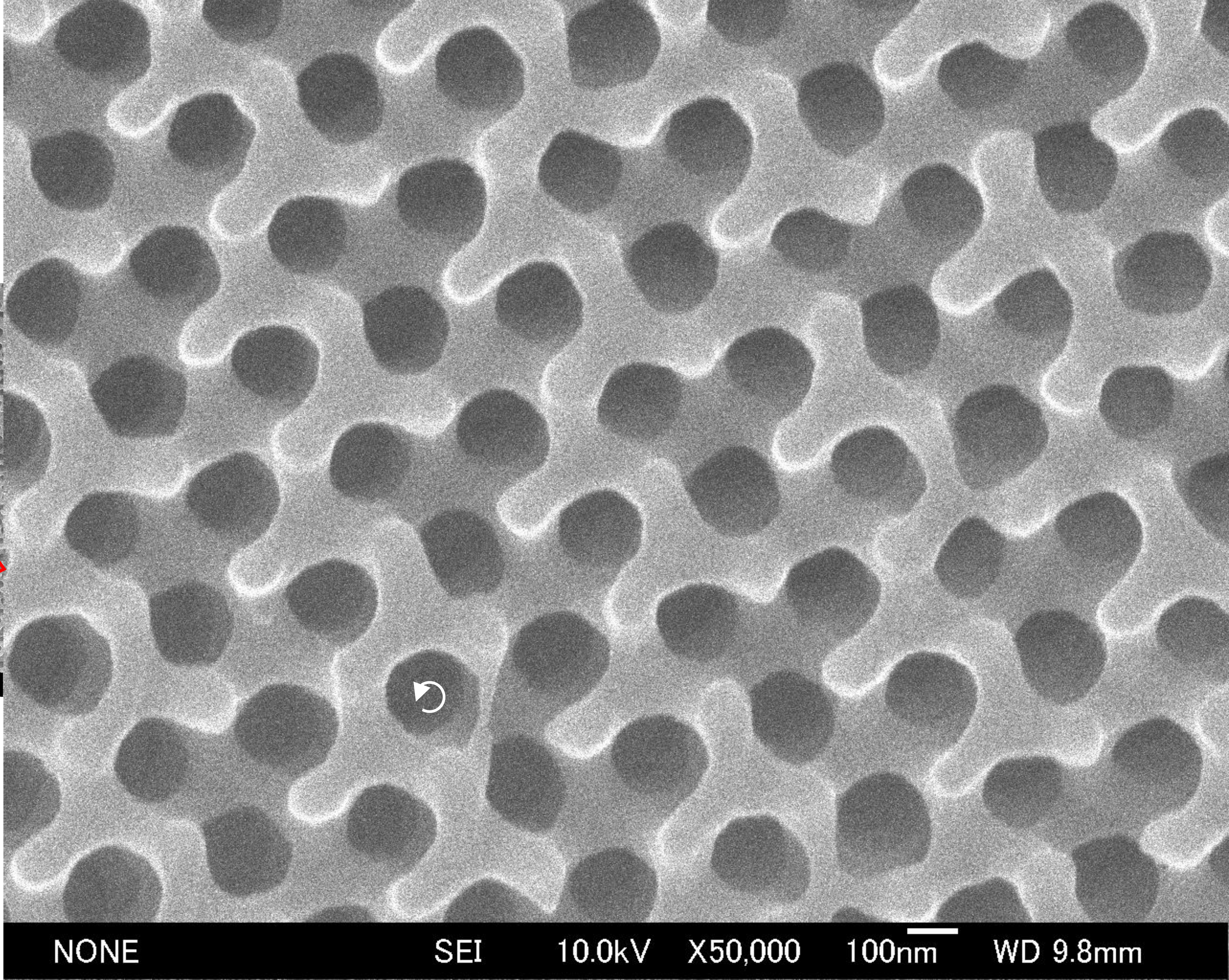

specimen No. 1
scale No. 1
domain No. 36
LH

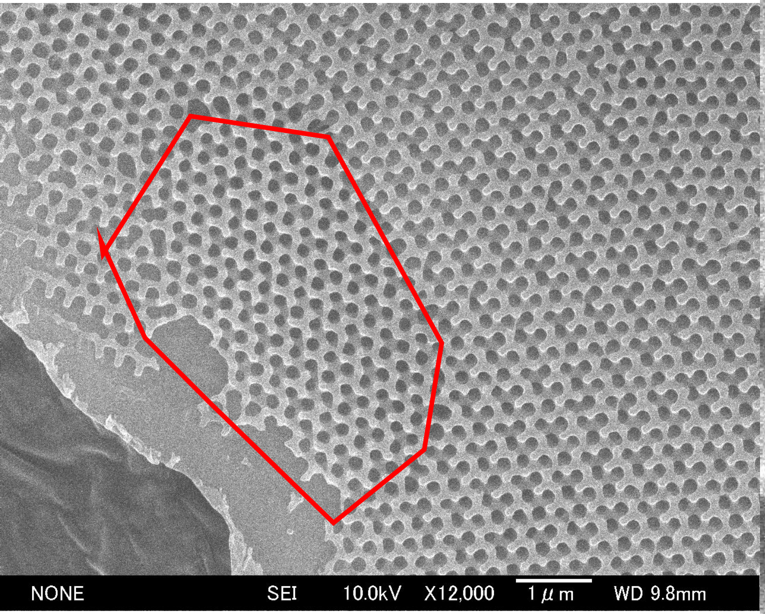

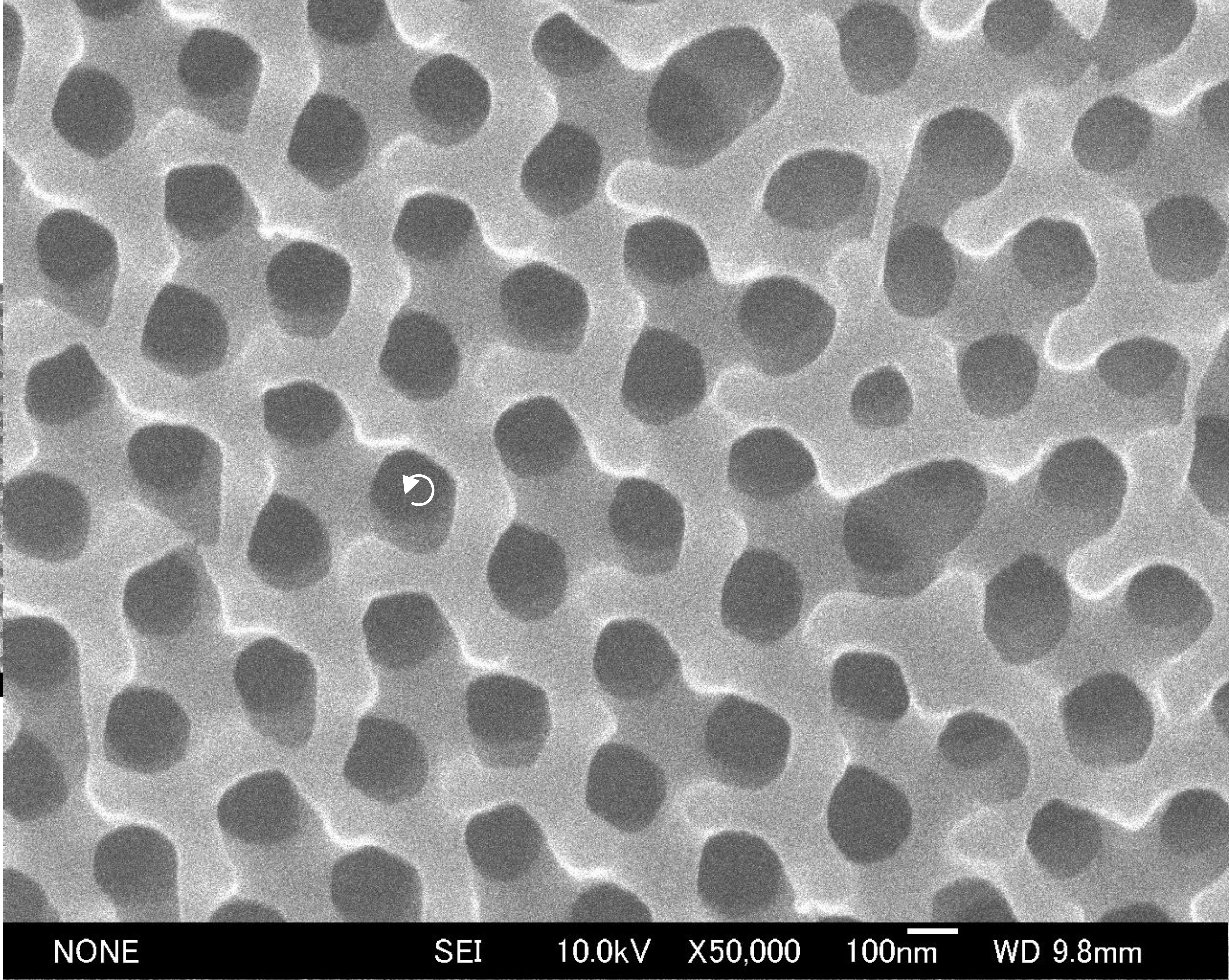

specimen No. 1
scale No. 1
domain No. 37
LH

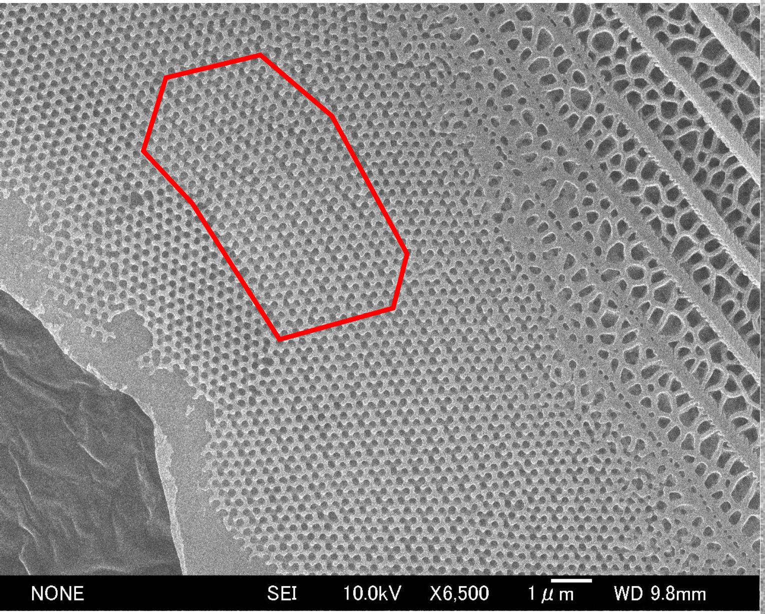

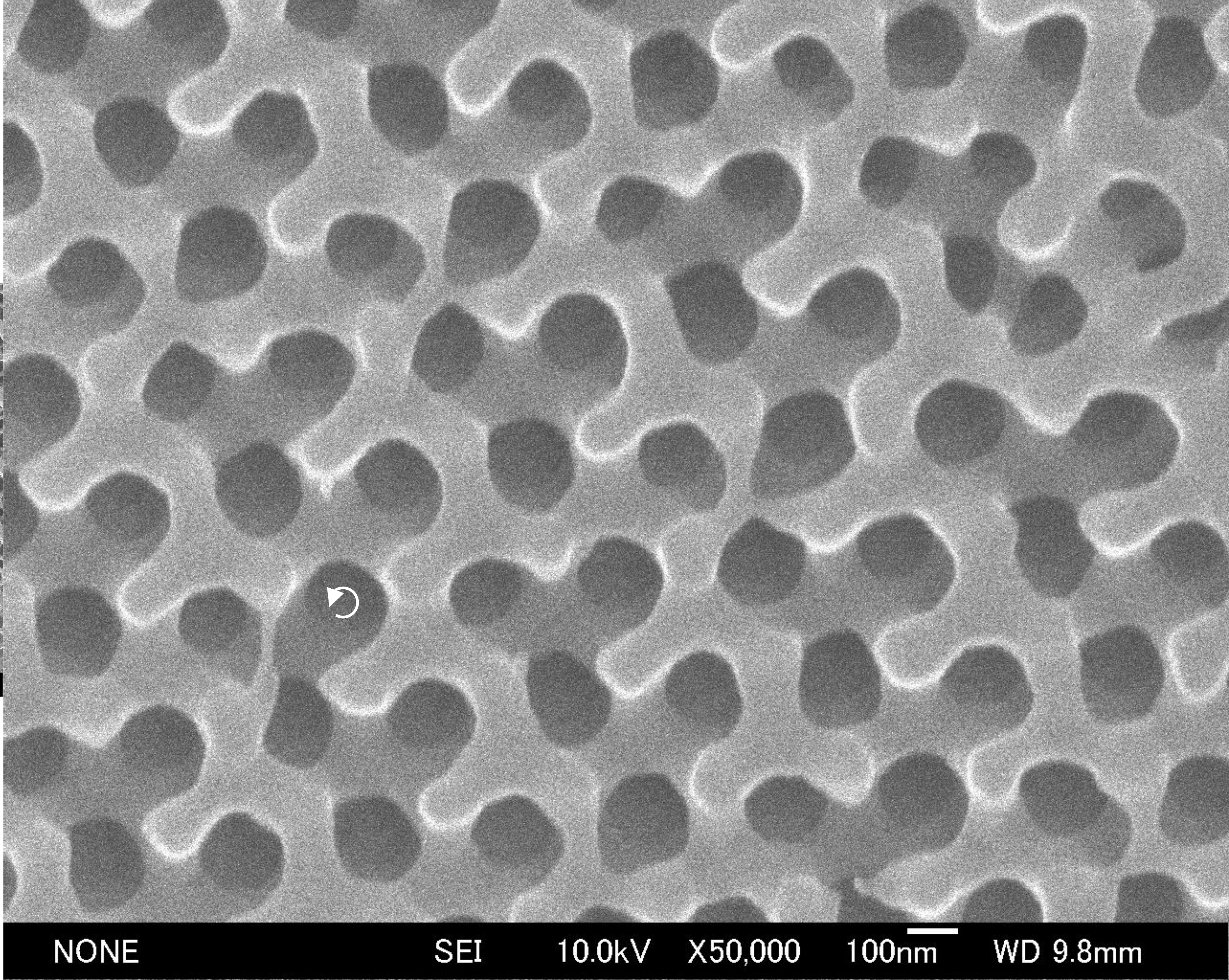

specimen No. 1
scale No. 1
domain No. 38
LH

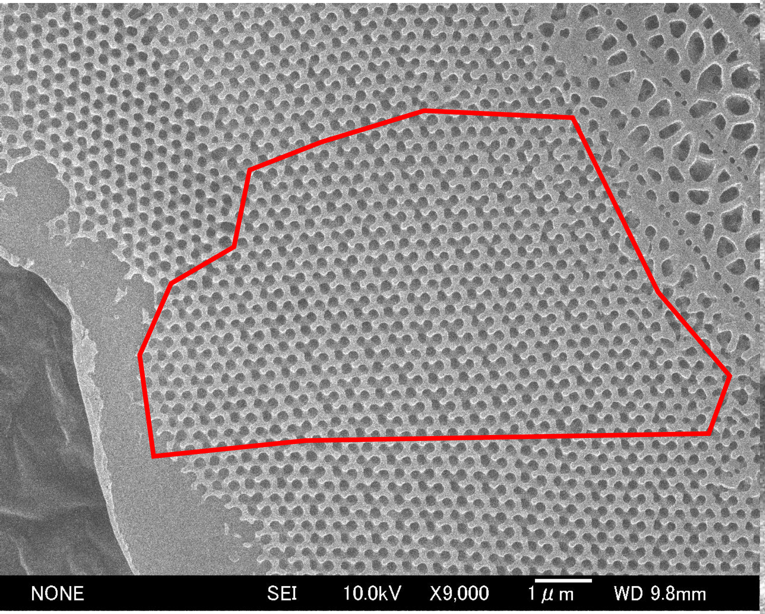

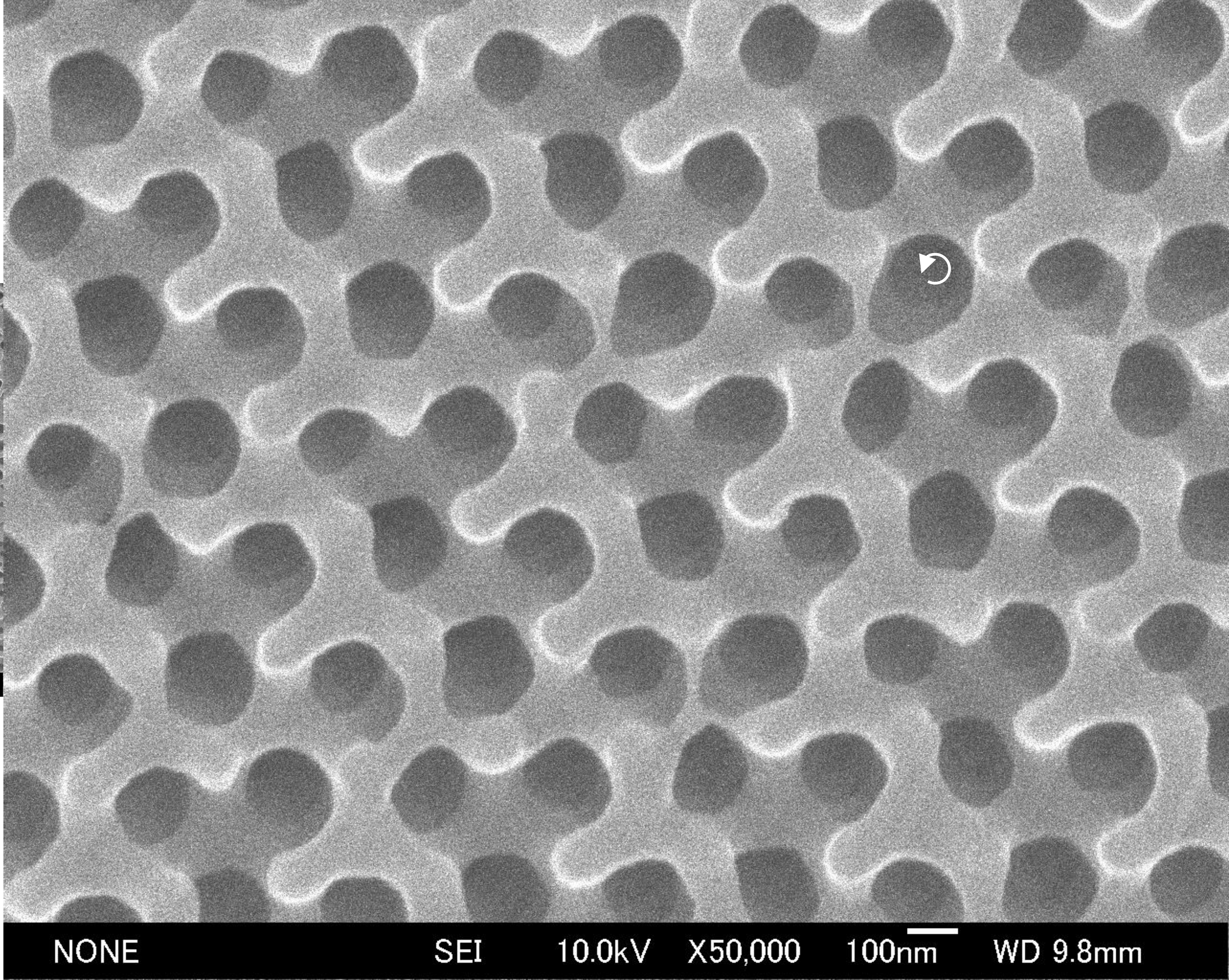

specimen No. 1
scale No. 1
domain No. 39
LH

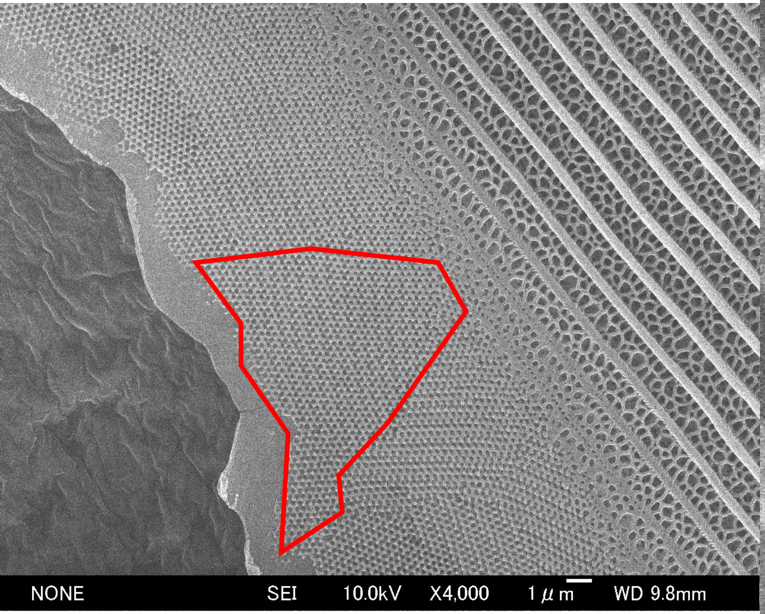

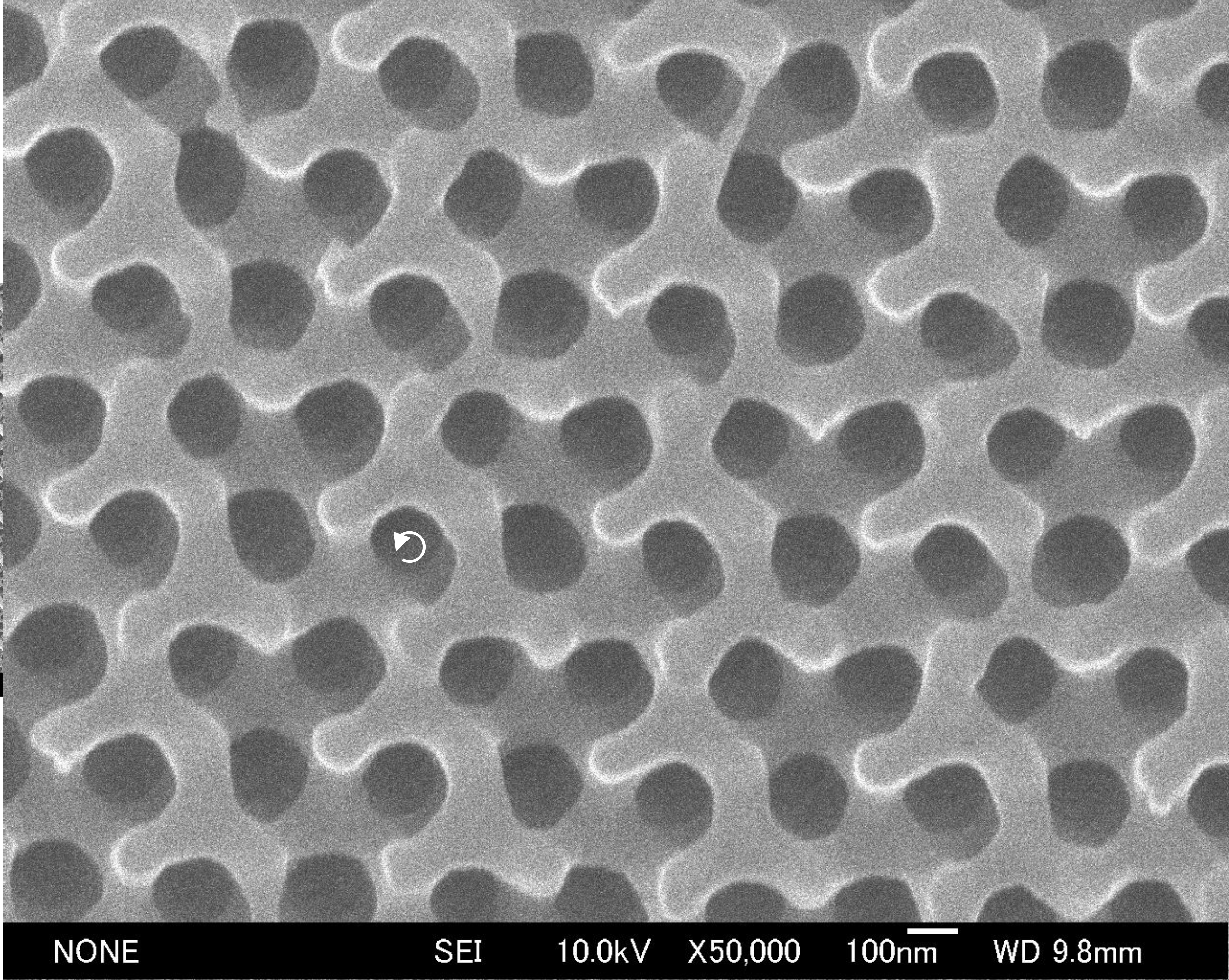

specimen No. 1
scale No. 1
domain No. 40
LH

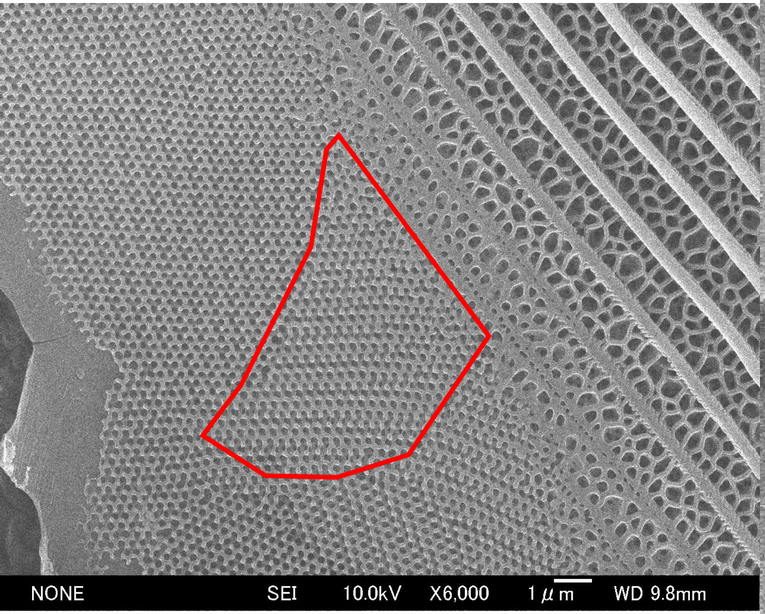

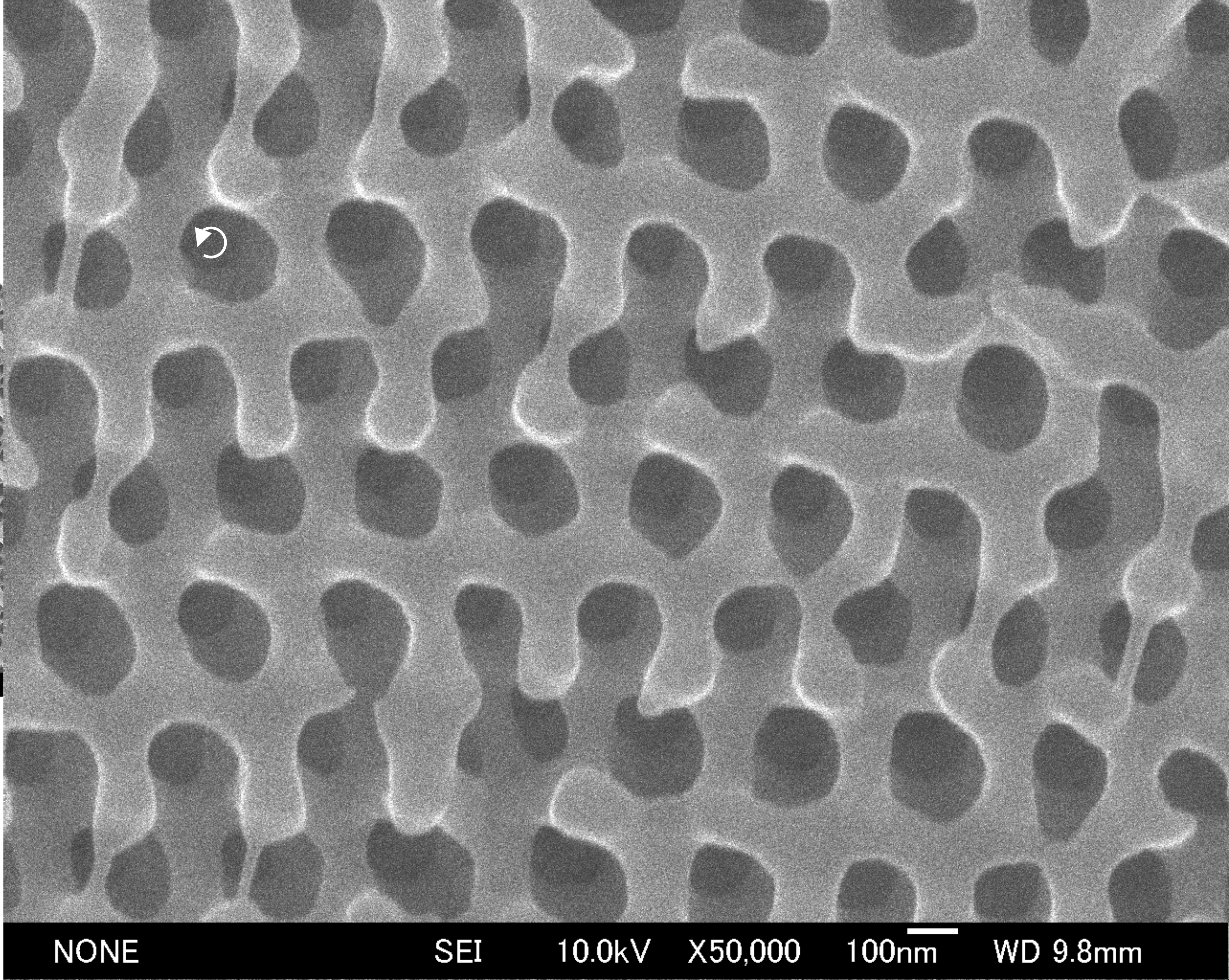

specimen No. 1
scale No. 1
domain No. 41
LH

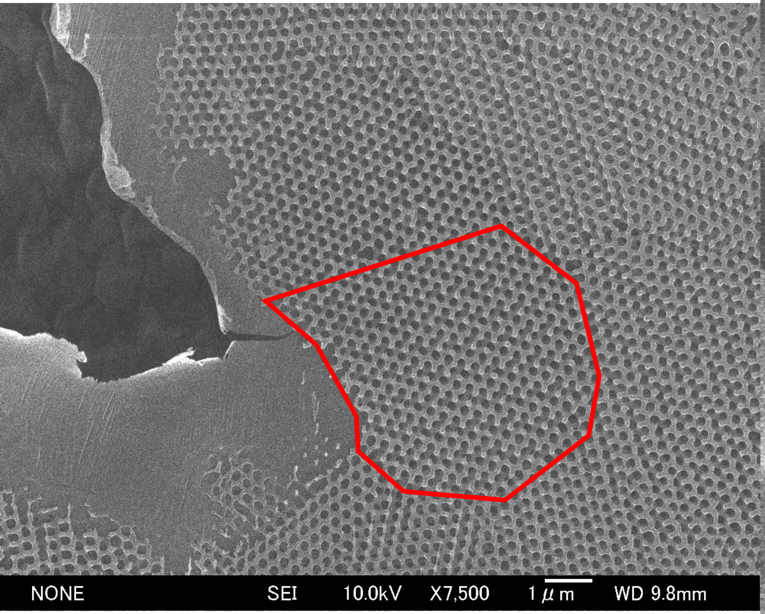

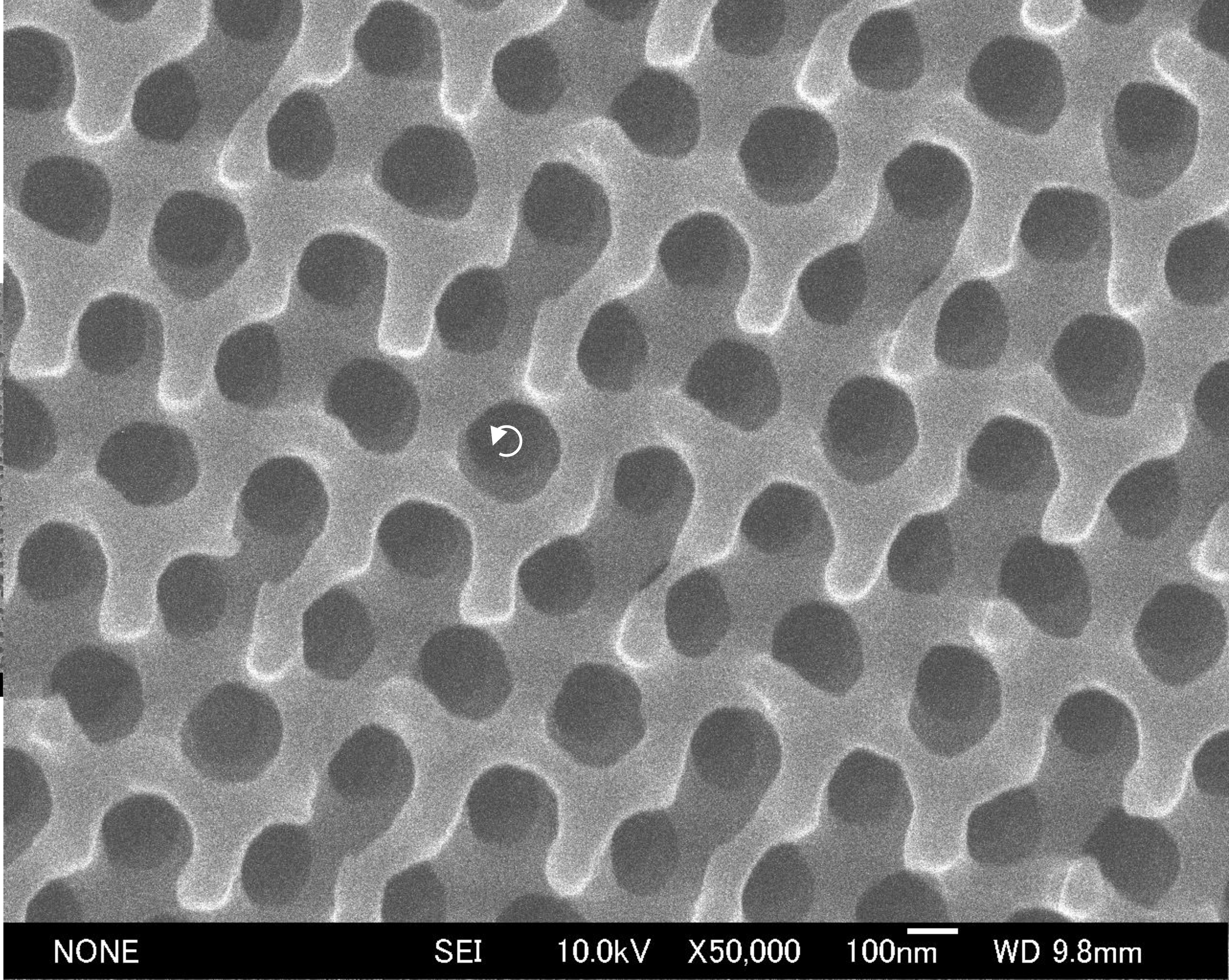

specimen No. 1
scale No. 1
domain No. 42
LH

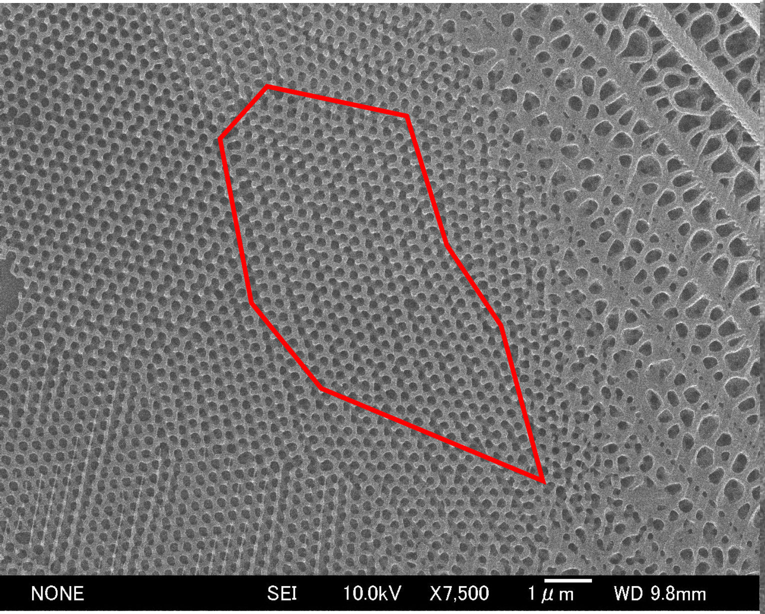

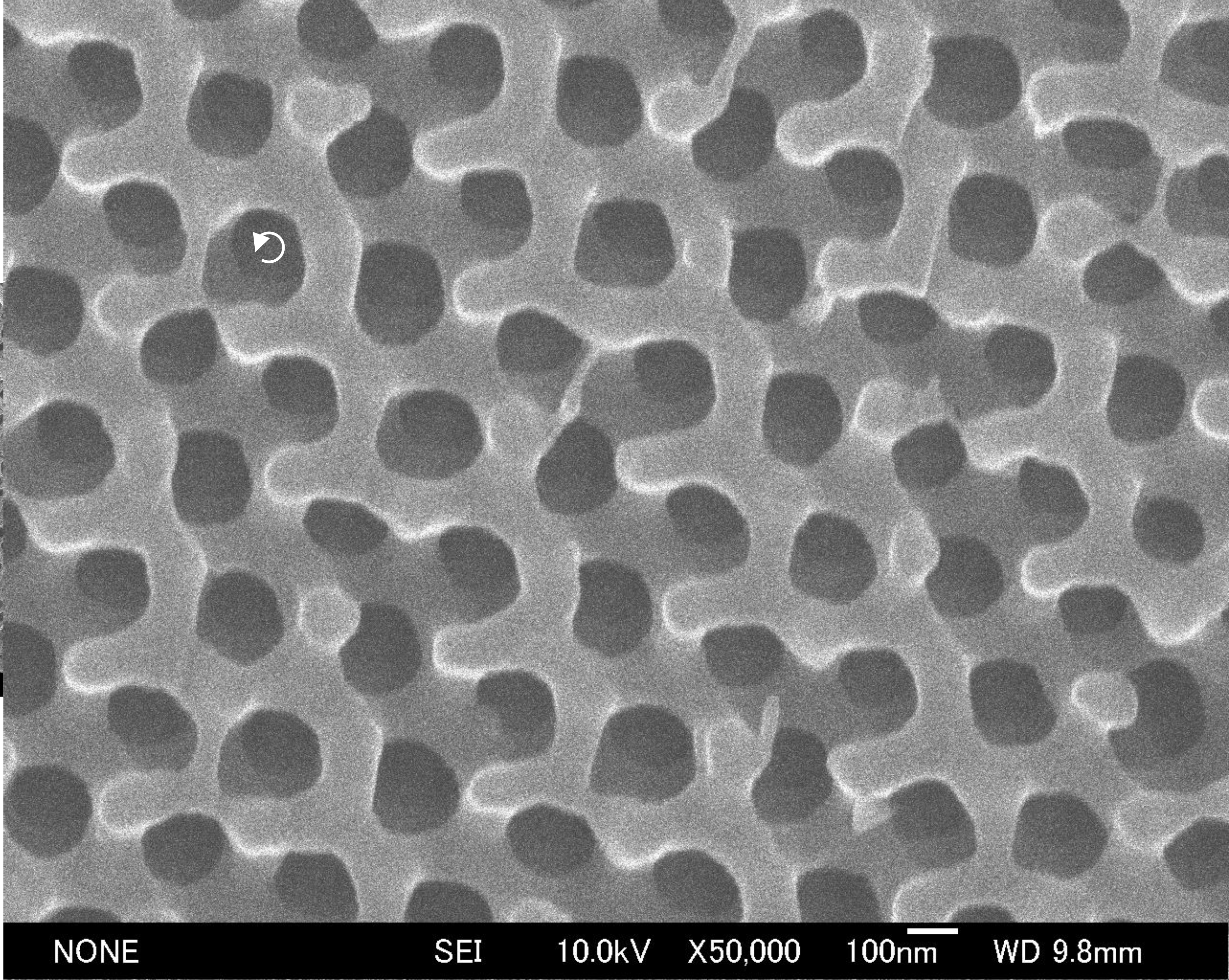

specimen No. 1
scale No. 1
domain No. 43
LH

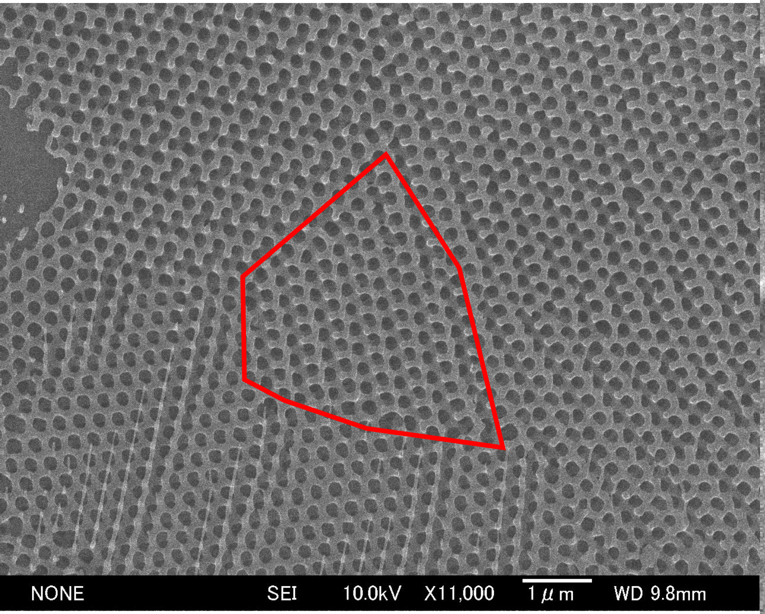

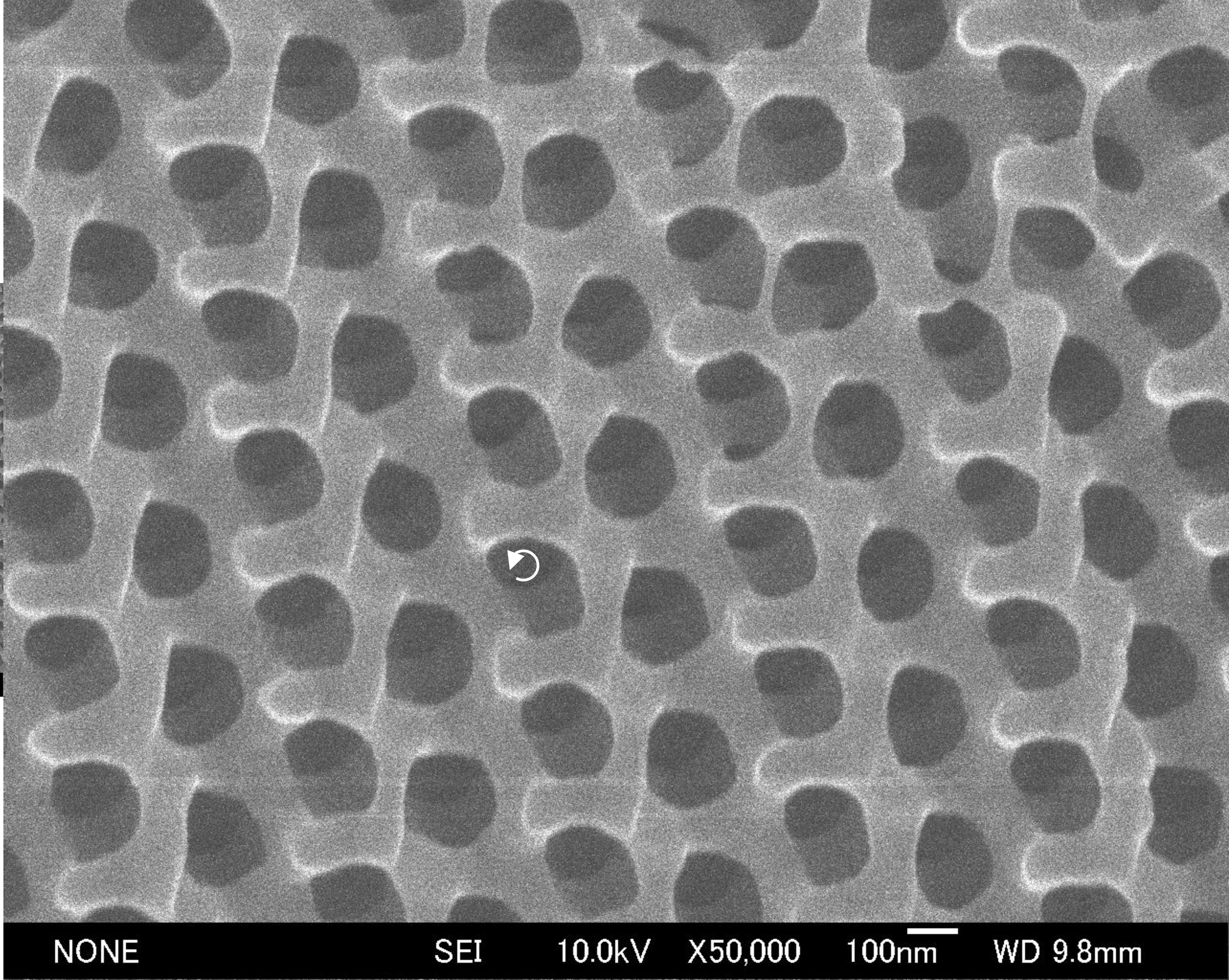

specimen No. 1
scale No. 1
domain No. 44
LH

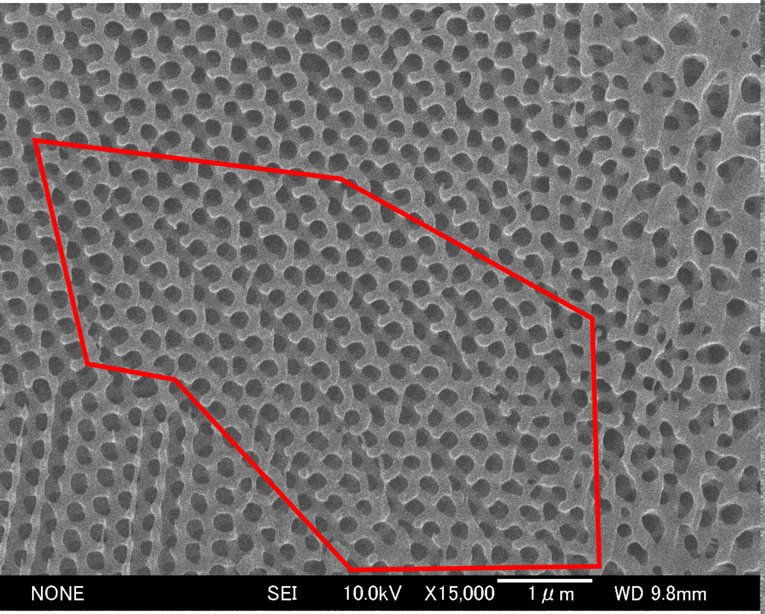

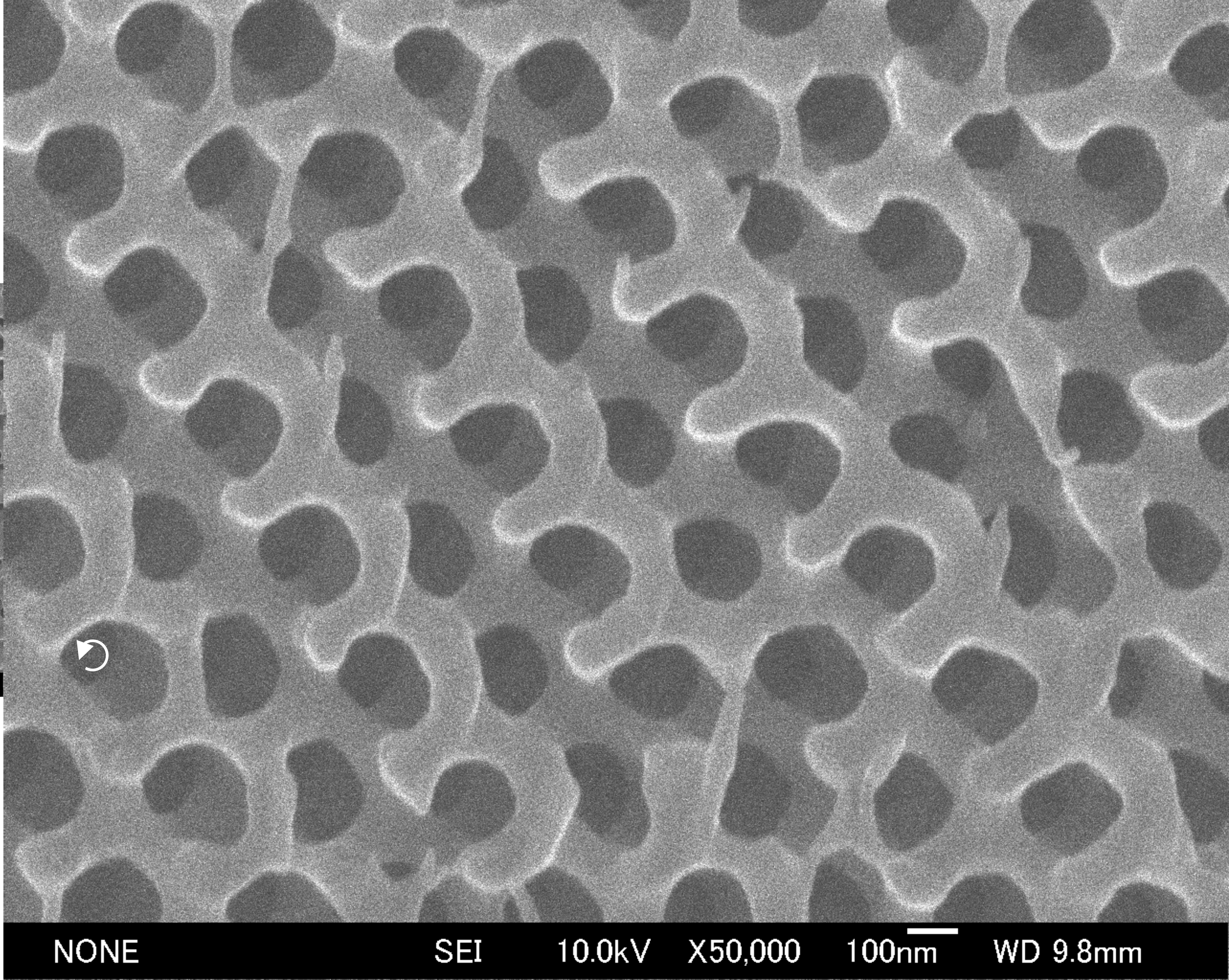

specimen No. 1
scale No. 1
domain No. 45
LH

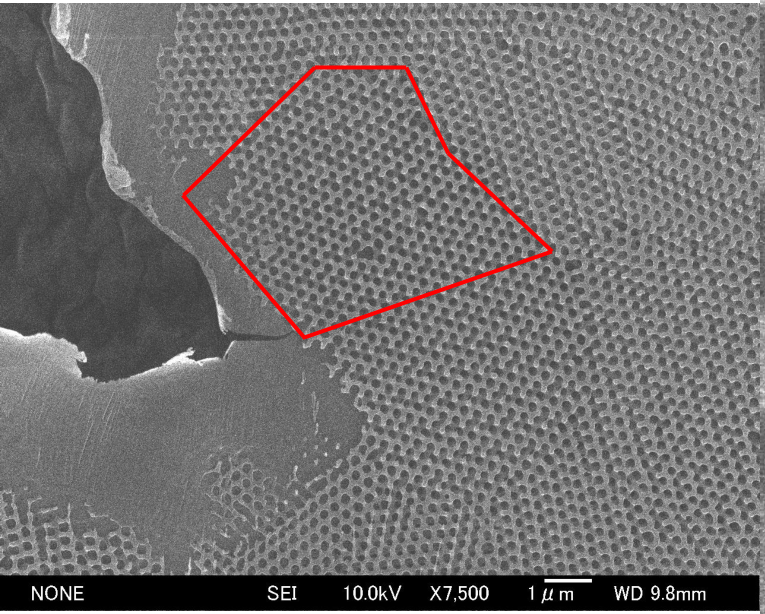

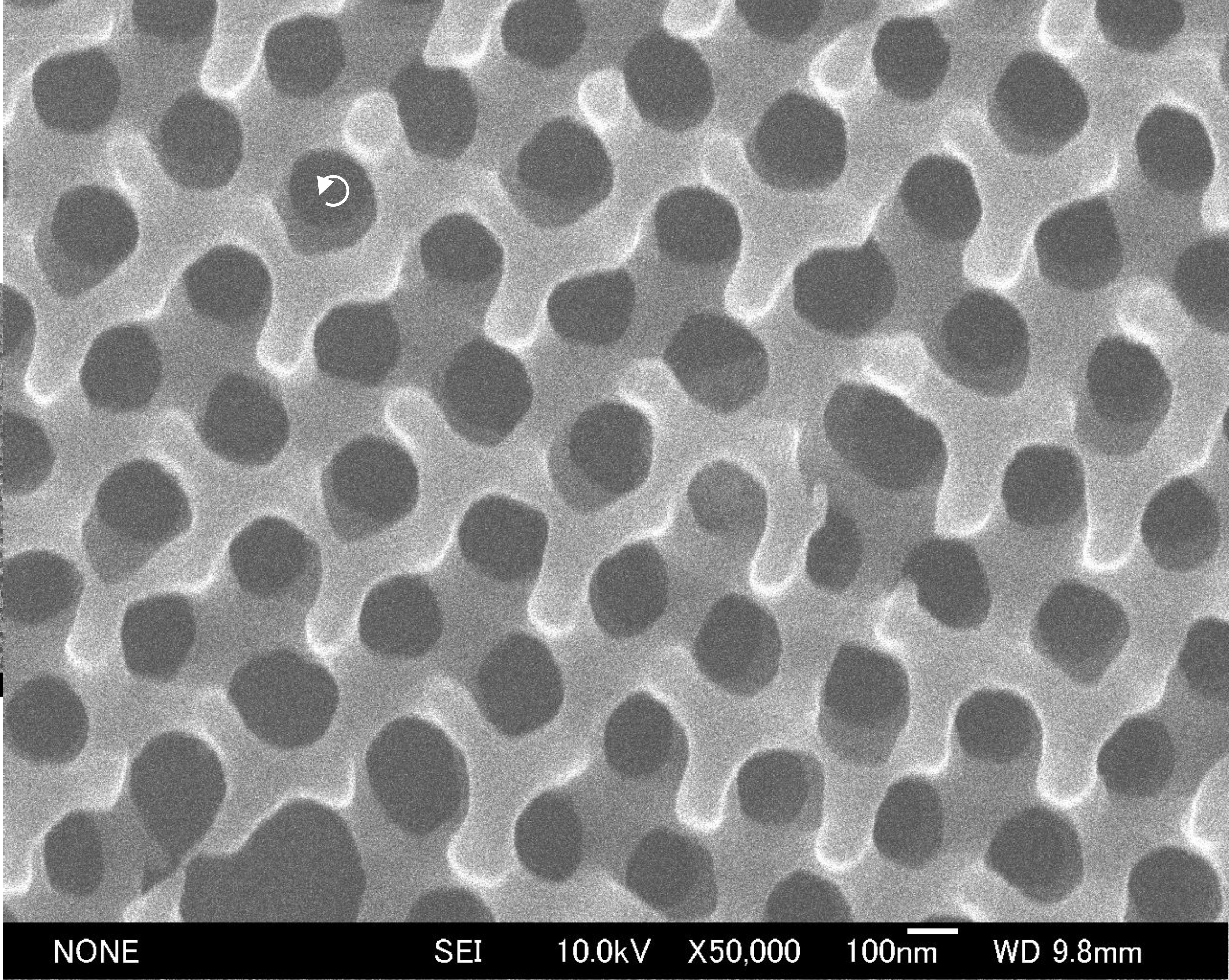

specimen No. 1, scale No. 2

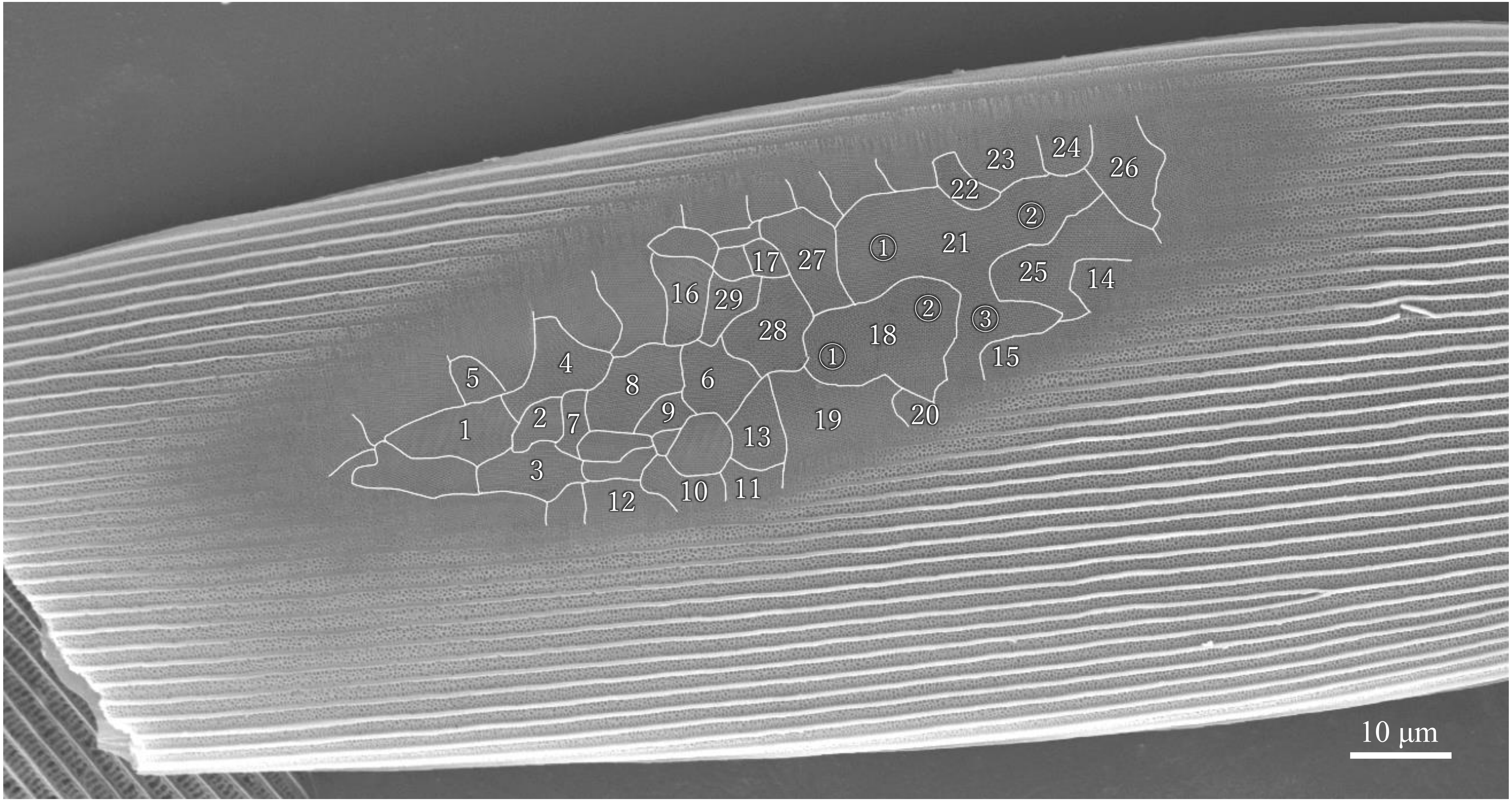

specimen No. 1
scale No. 2
domain No. 1
LH

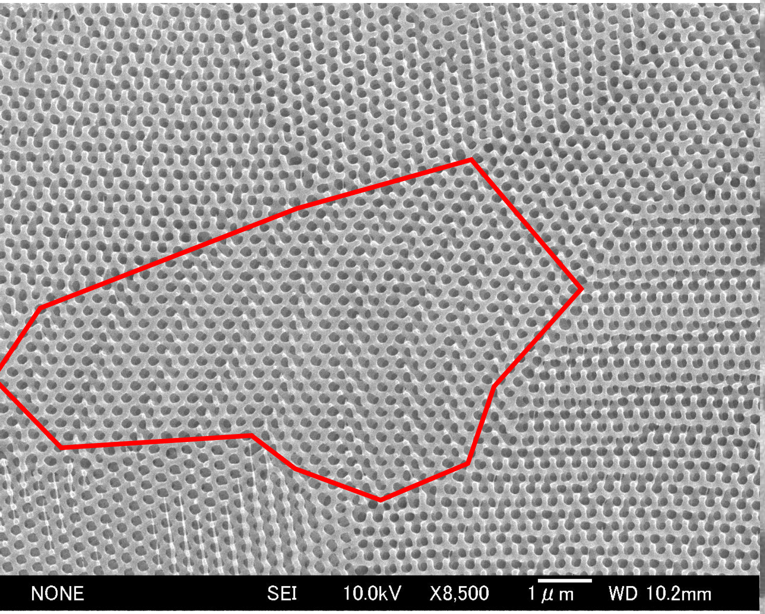

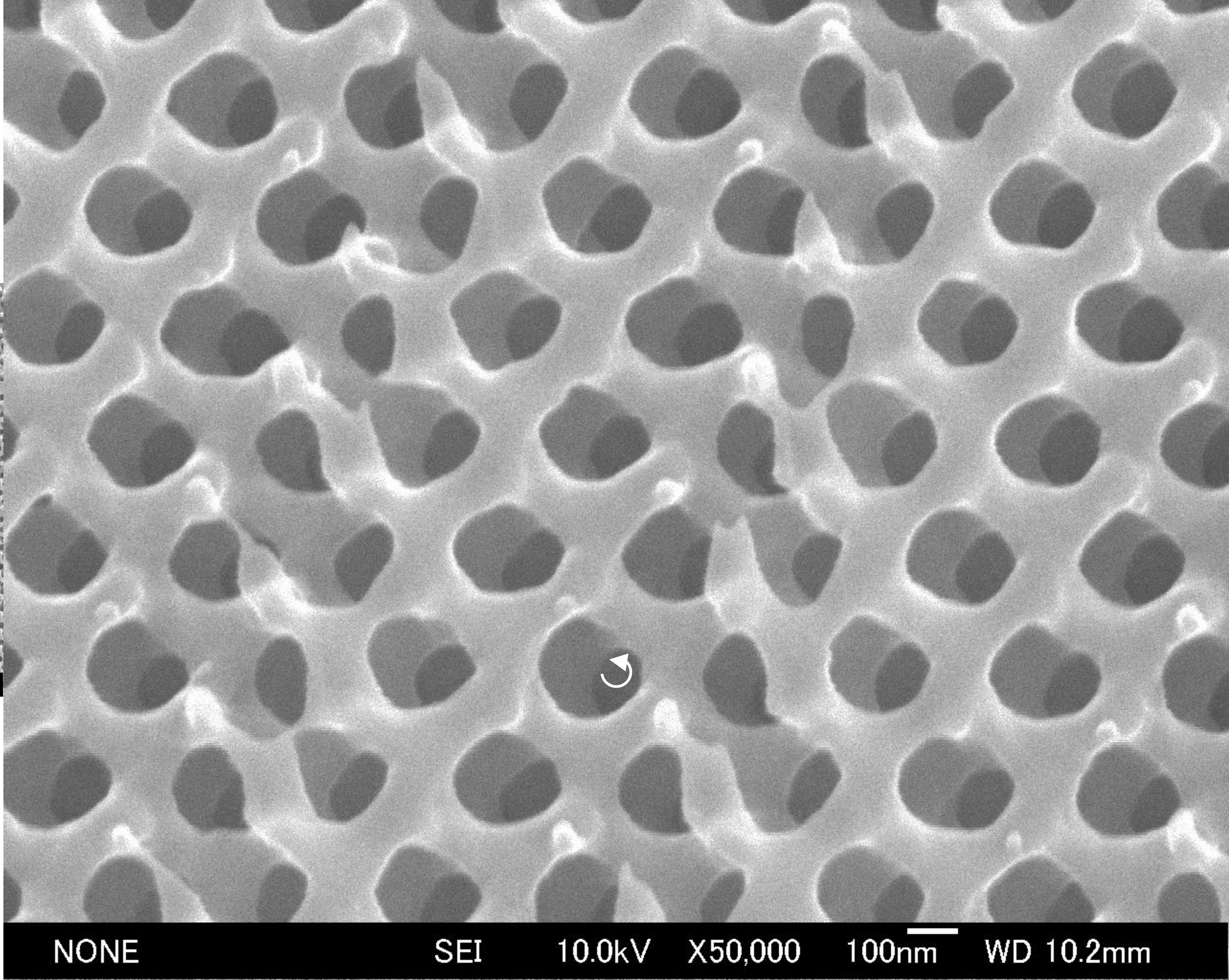

specimen No. 1
scale No. 2
domain No. 2
LH

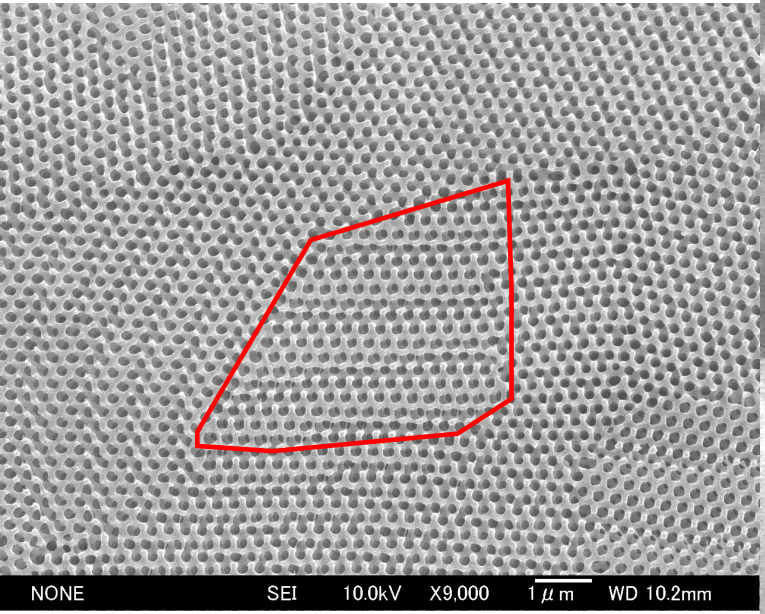

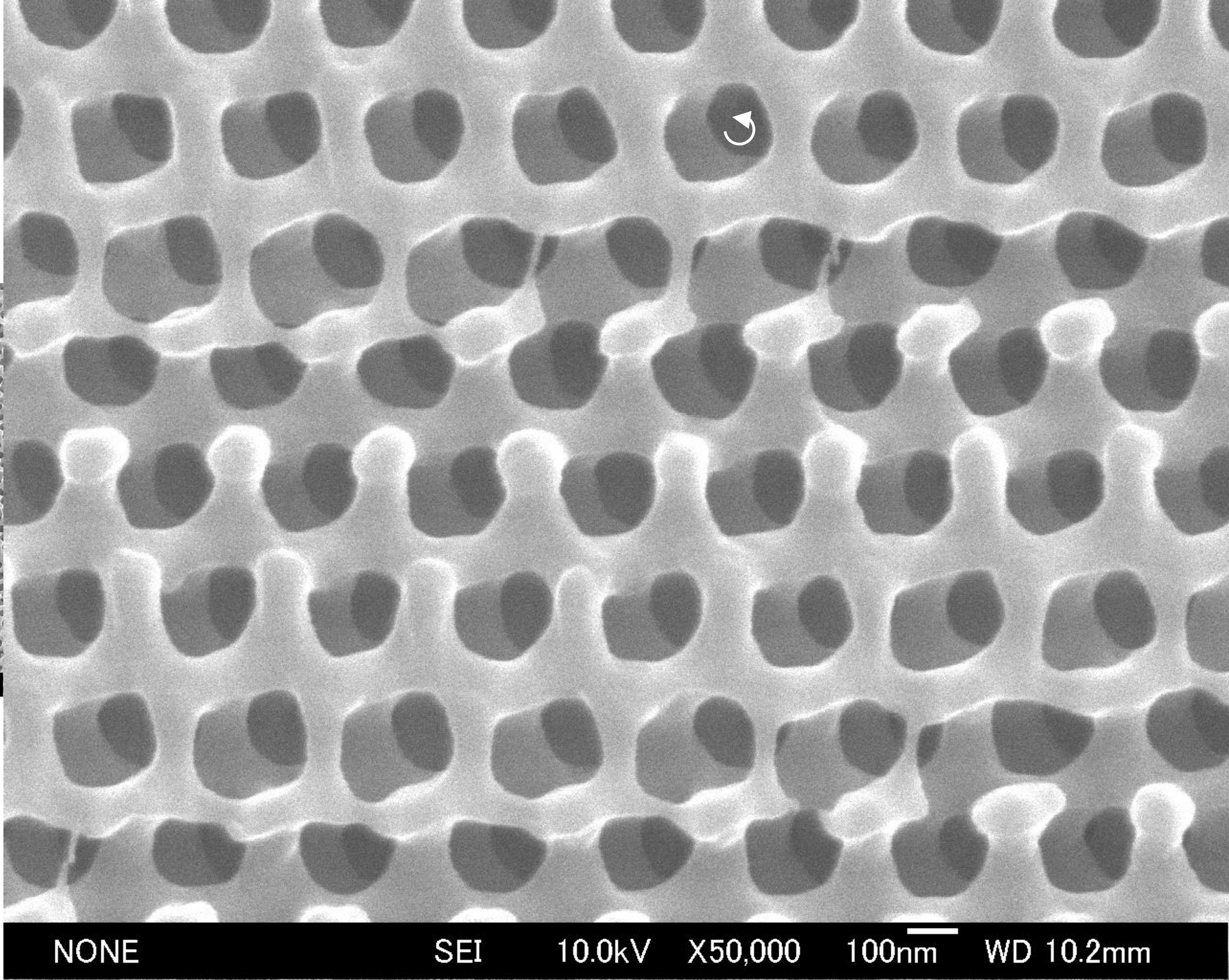

specimen No. 1
scale No. 2
domain No. 3
LH

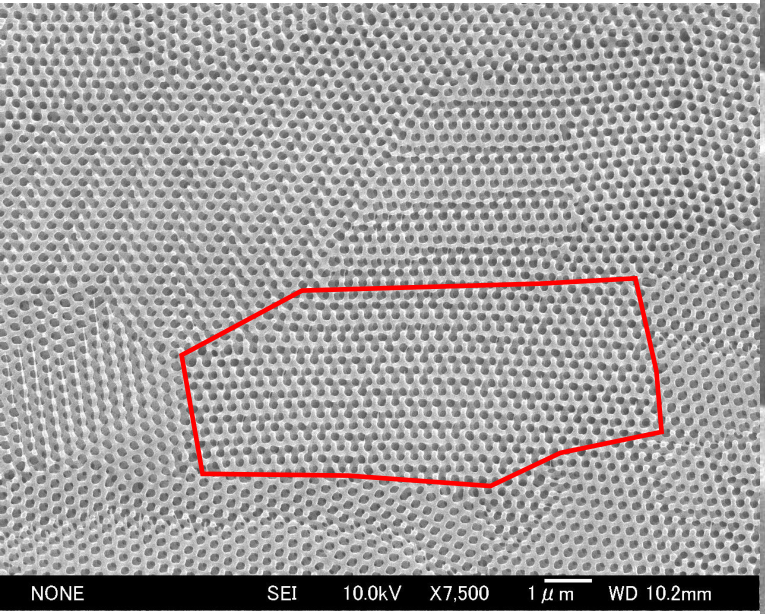

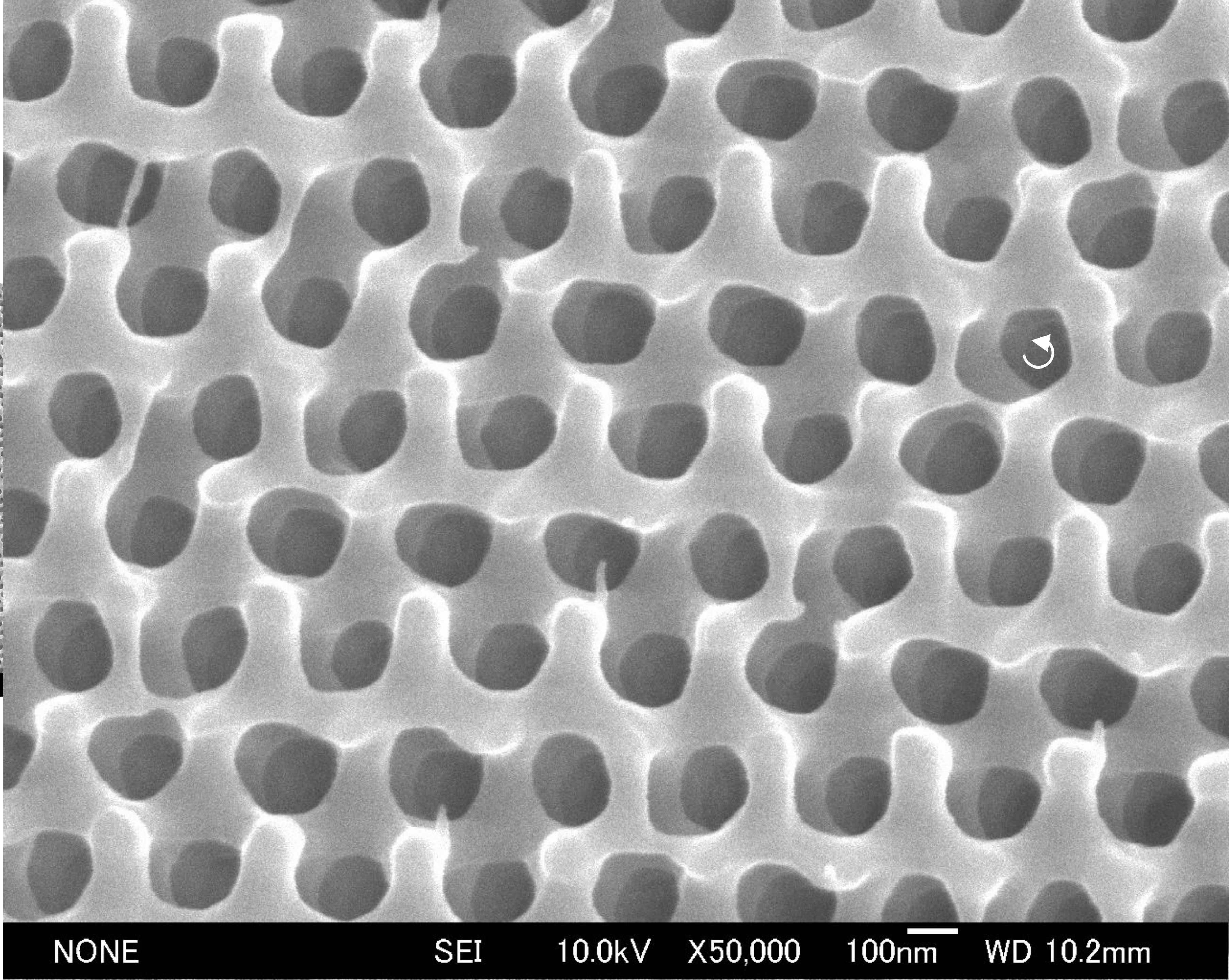

specimen No. 1
scale No. 2
domain No. 4
LH

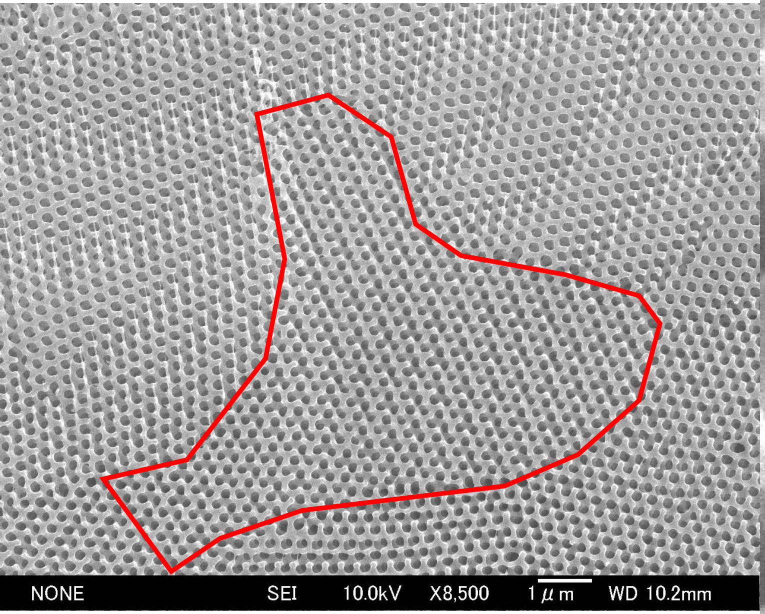

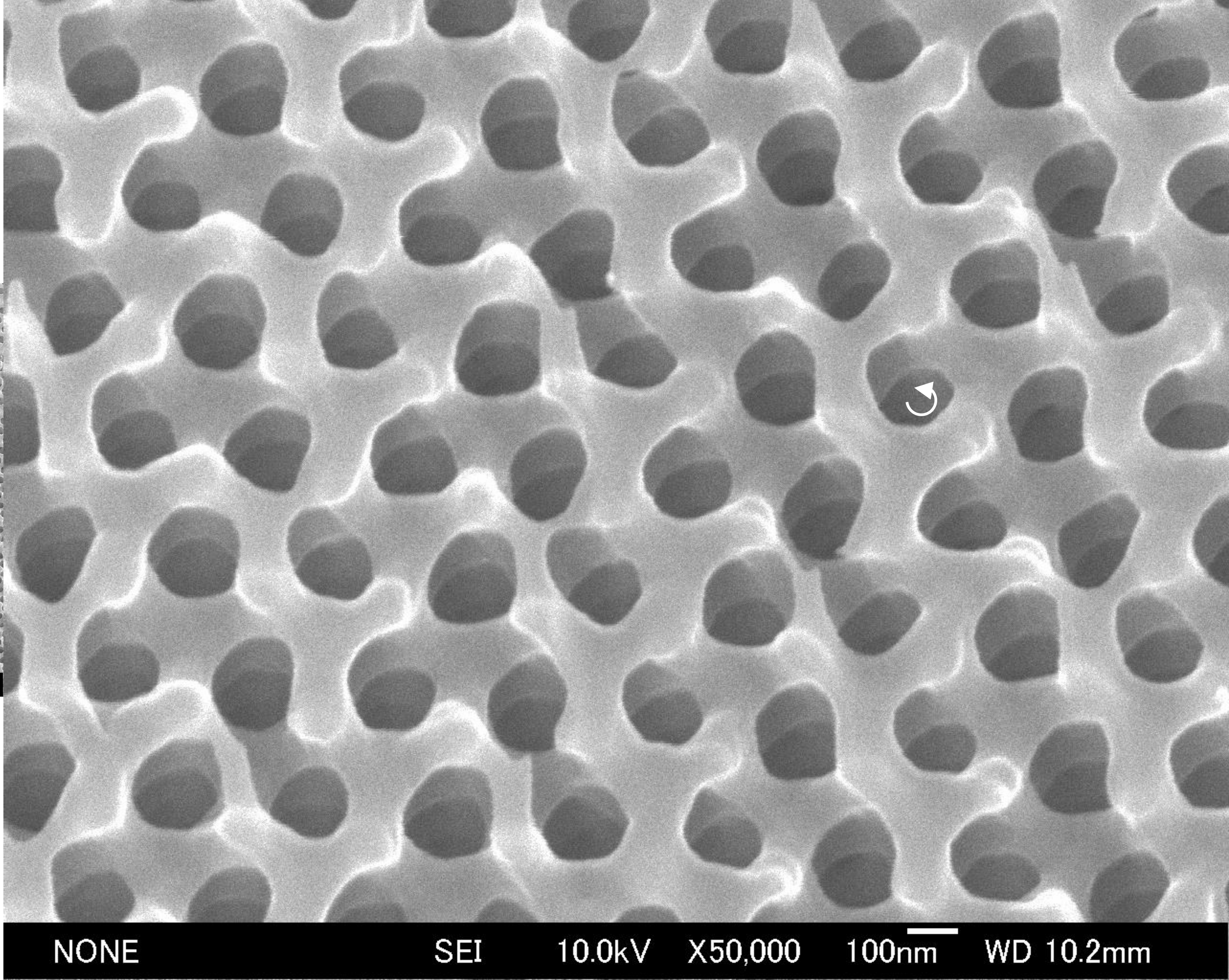

specimen No. 1
scale No. 2
domain No. 5
LH

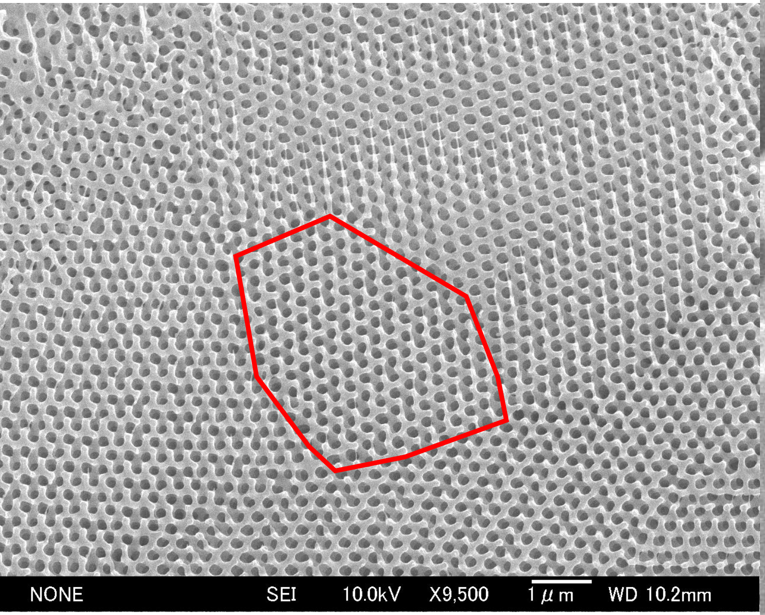

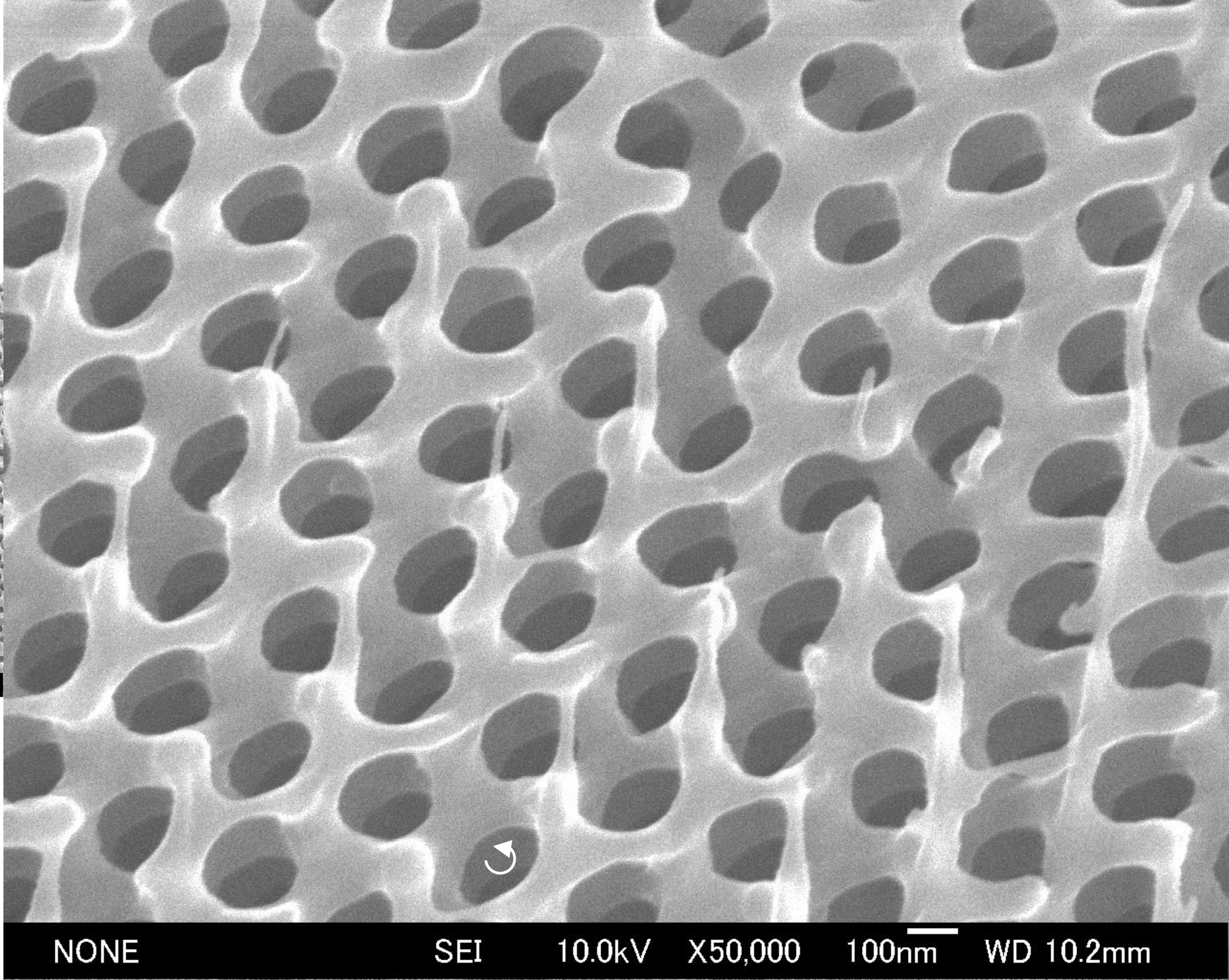

specimen No. 1
scale No. 2
domain No. 6
LH

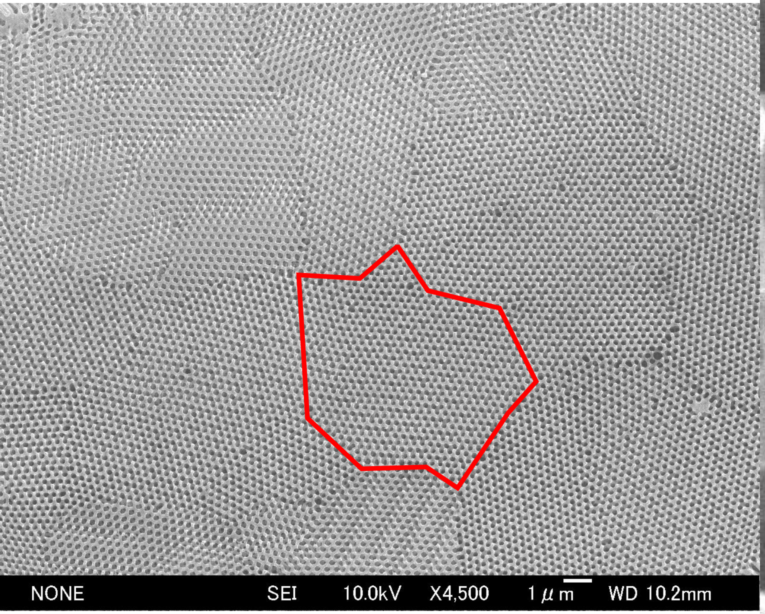

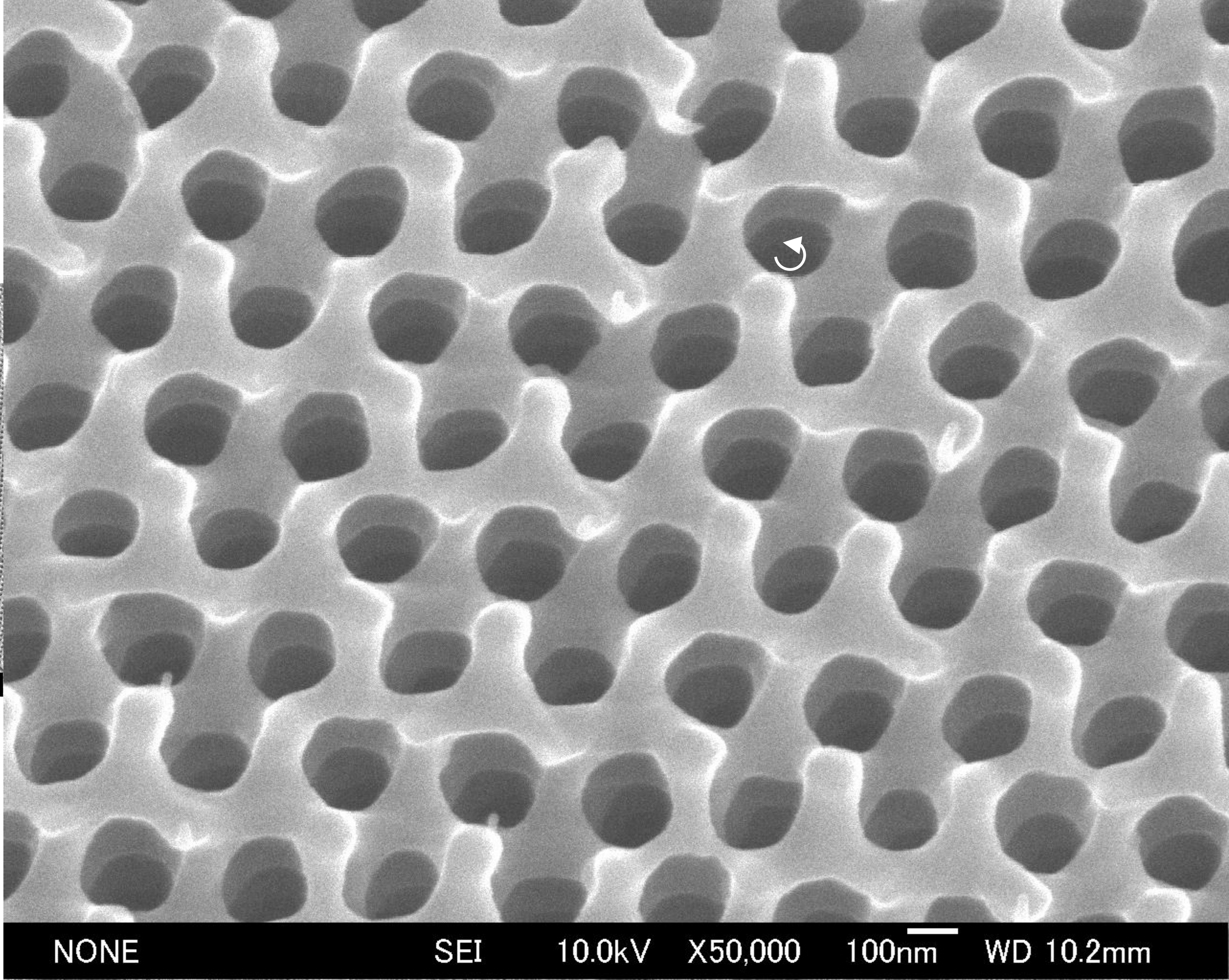

specimen No. 1
scale No. 2
domain No. 7
LH

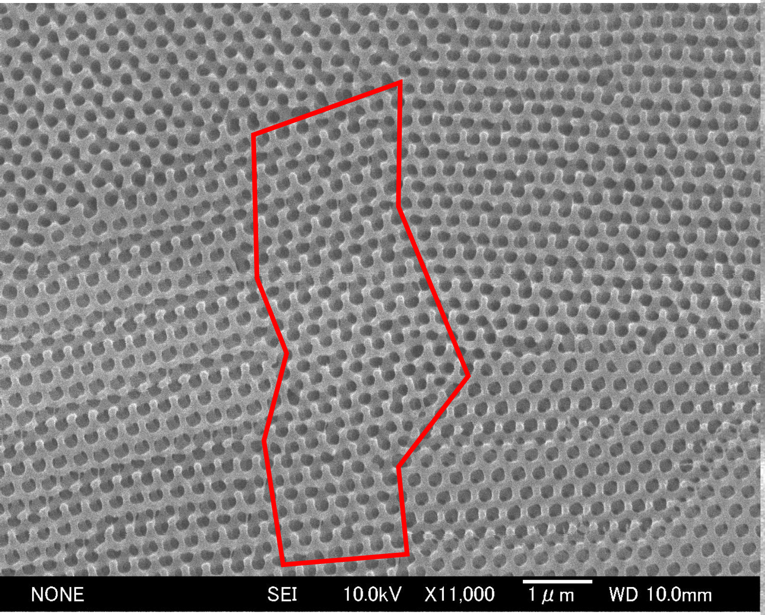

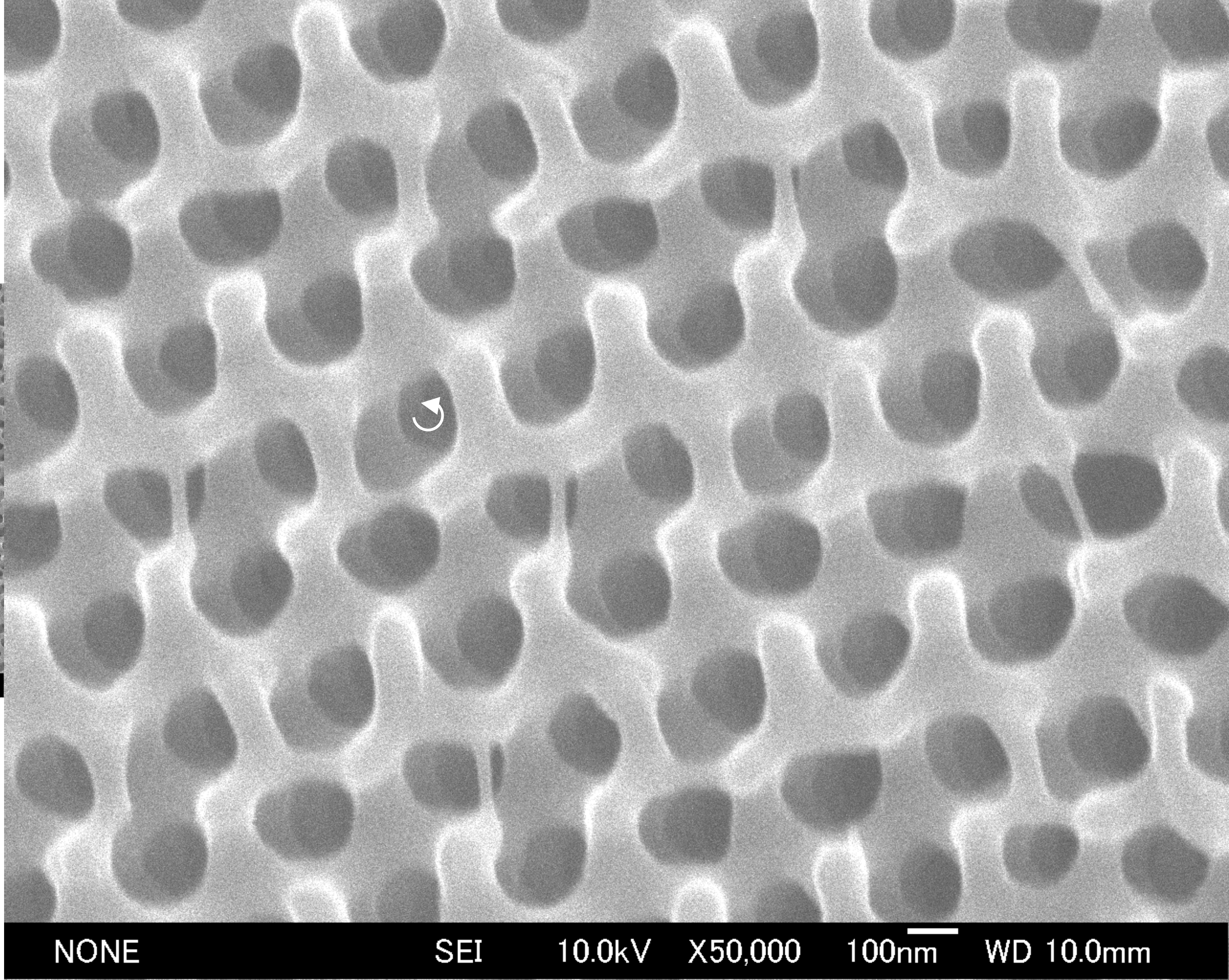

specimen No. 1
scale No. 2
domain No. 8
LH

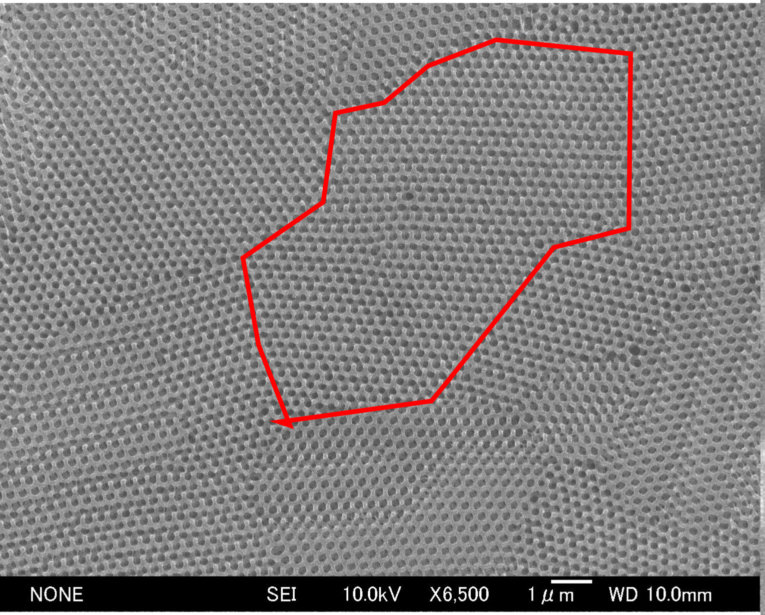

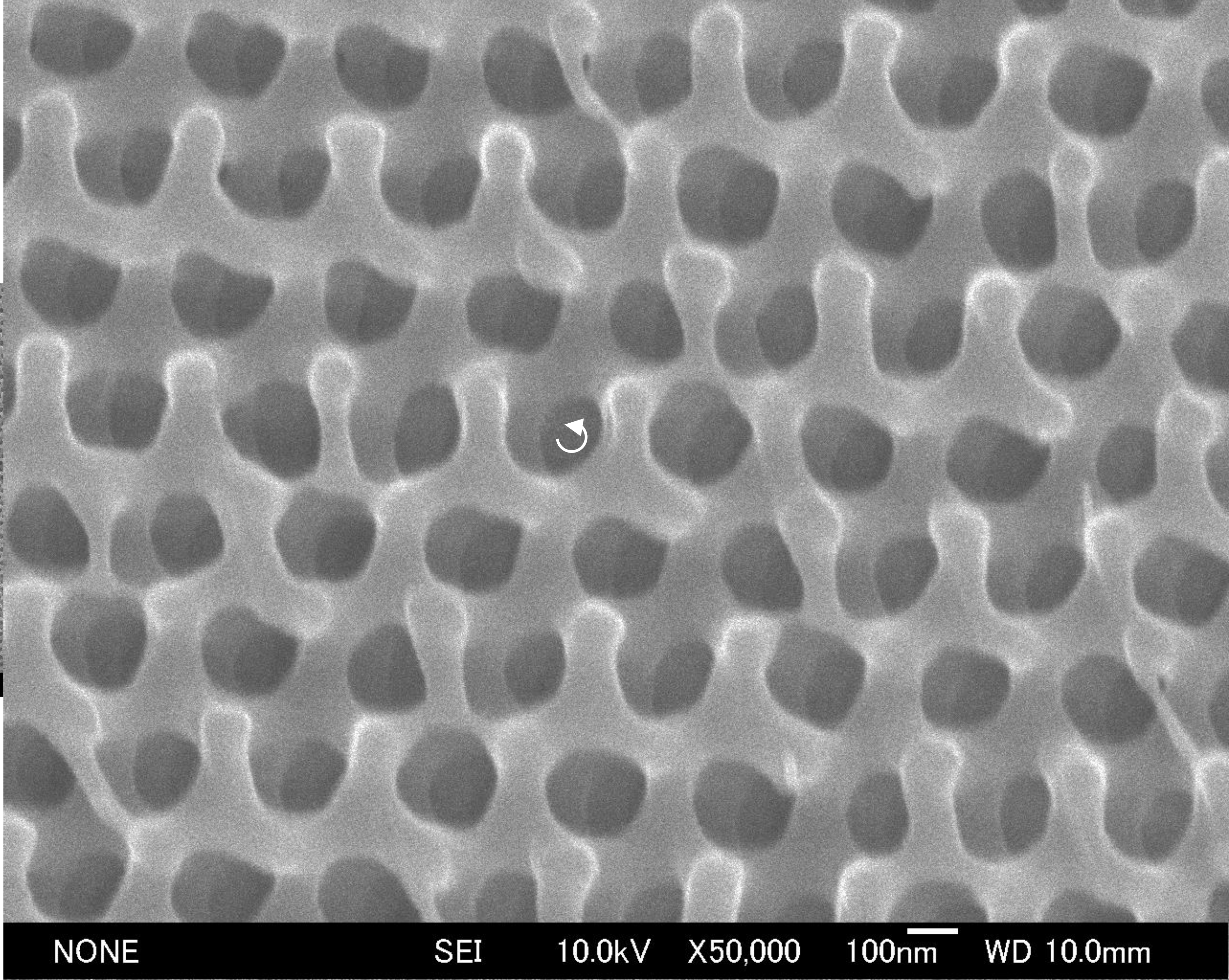

specimen No. 1
scale No. 2
domain No. 9
LH

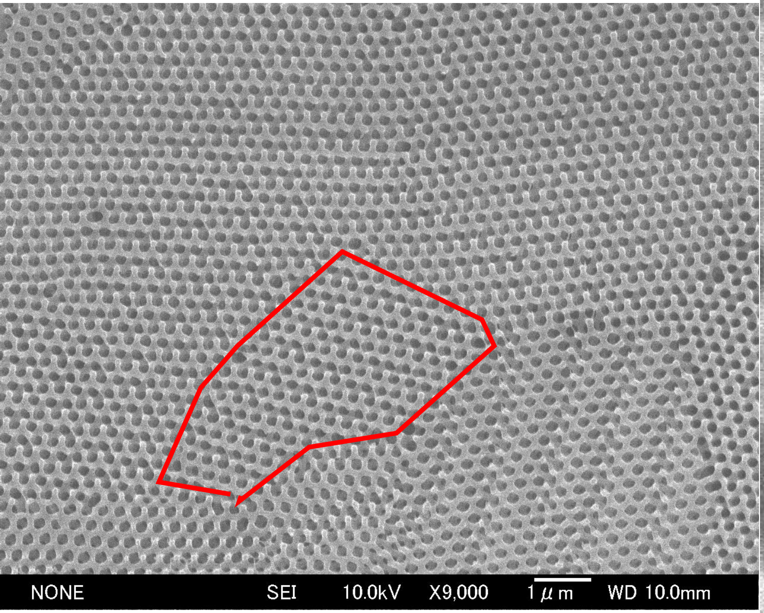

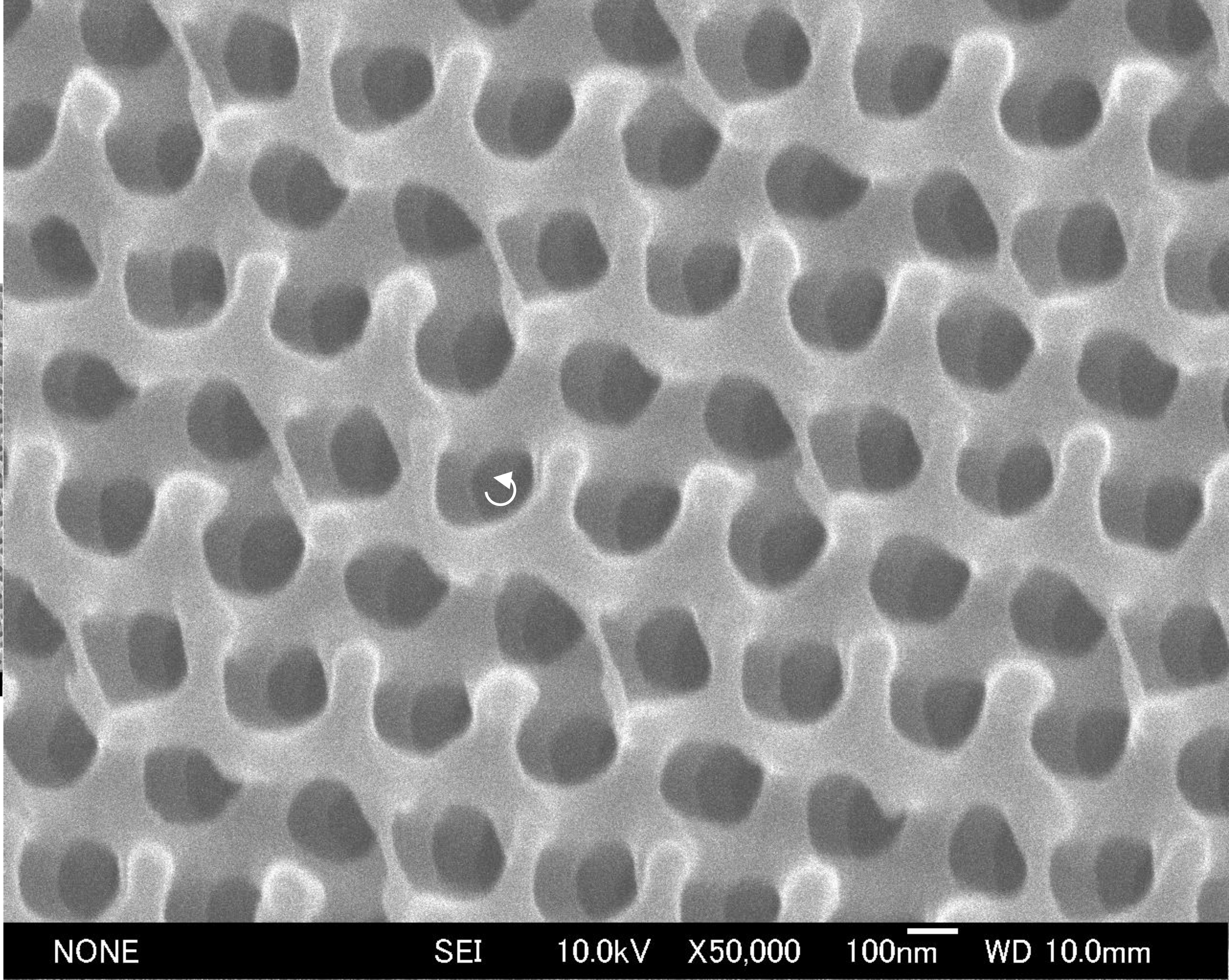

specimen No. 1
scale No. 2
domain No. 10
LH

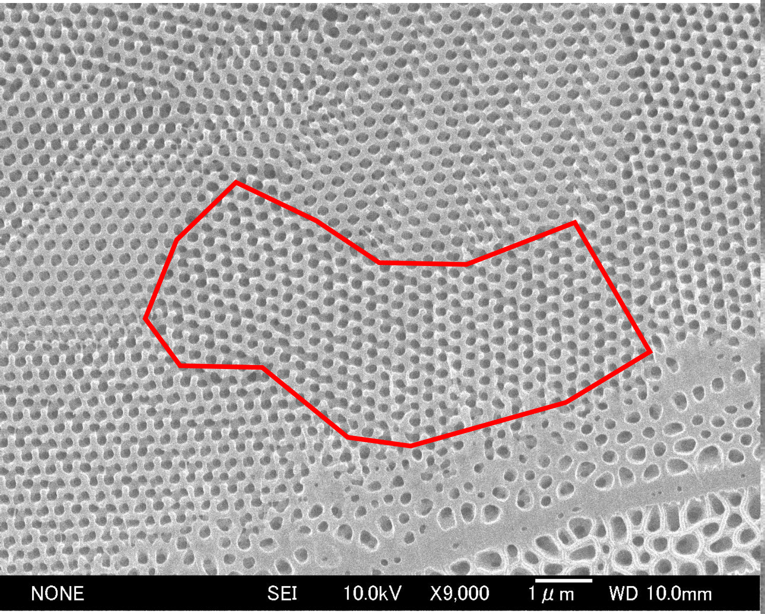

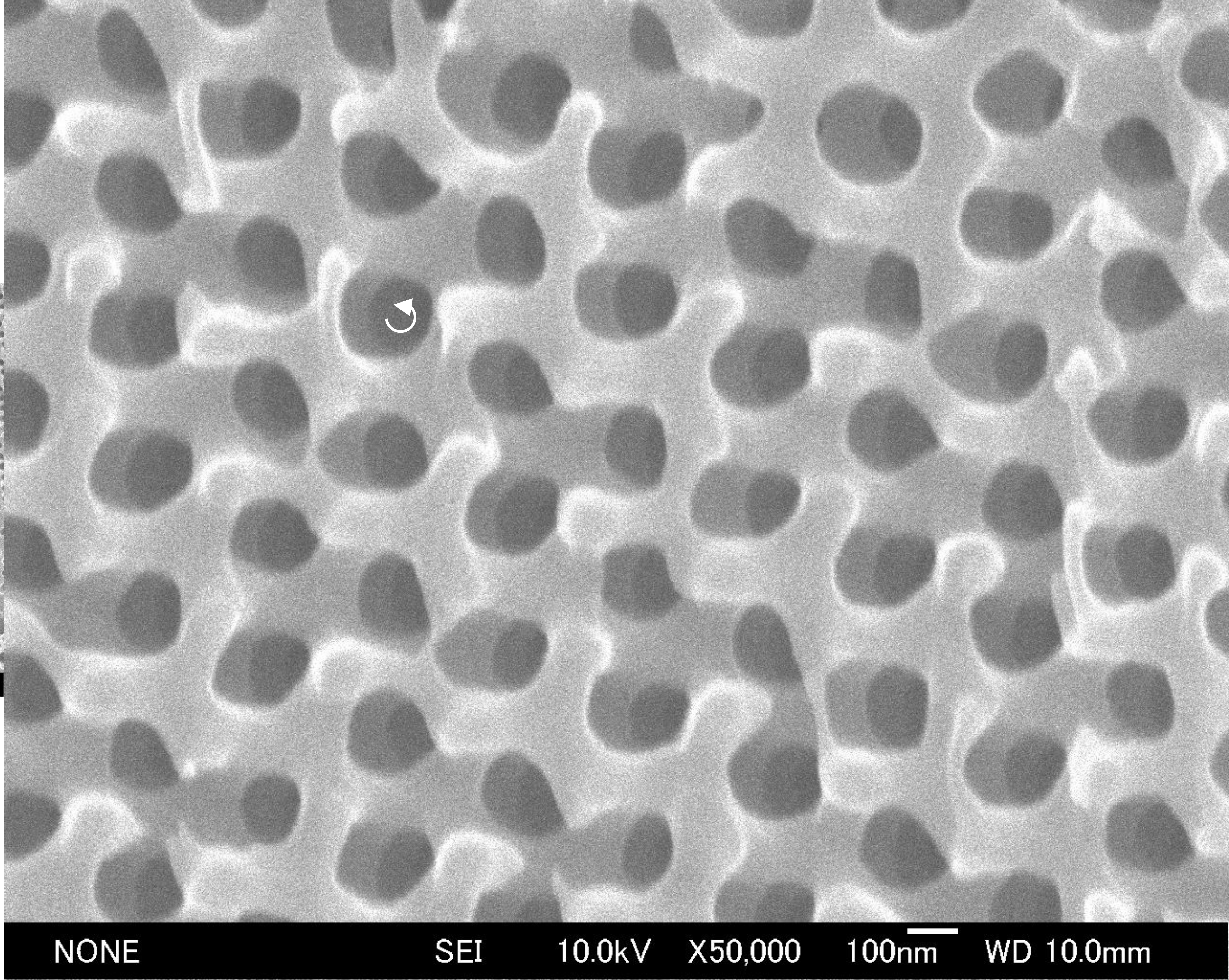

specimen No. 1
scale No. 2
domain No. 11
LH

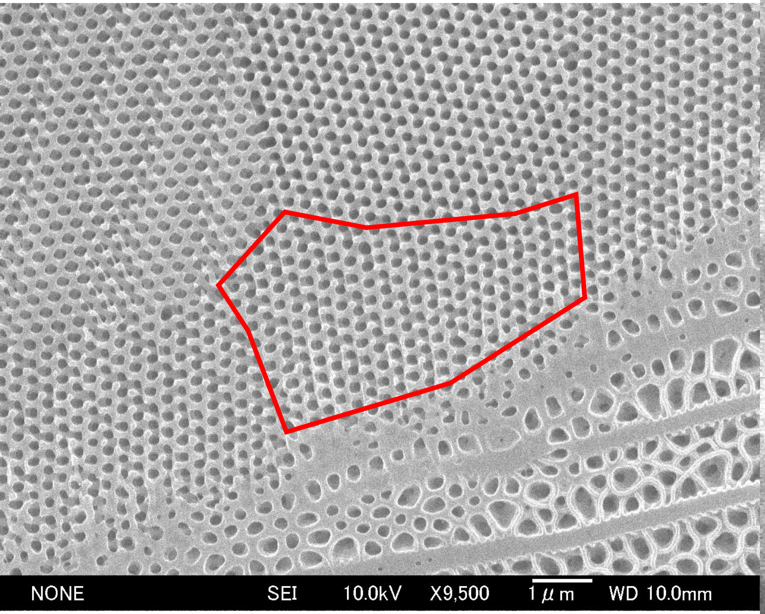

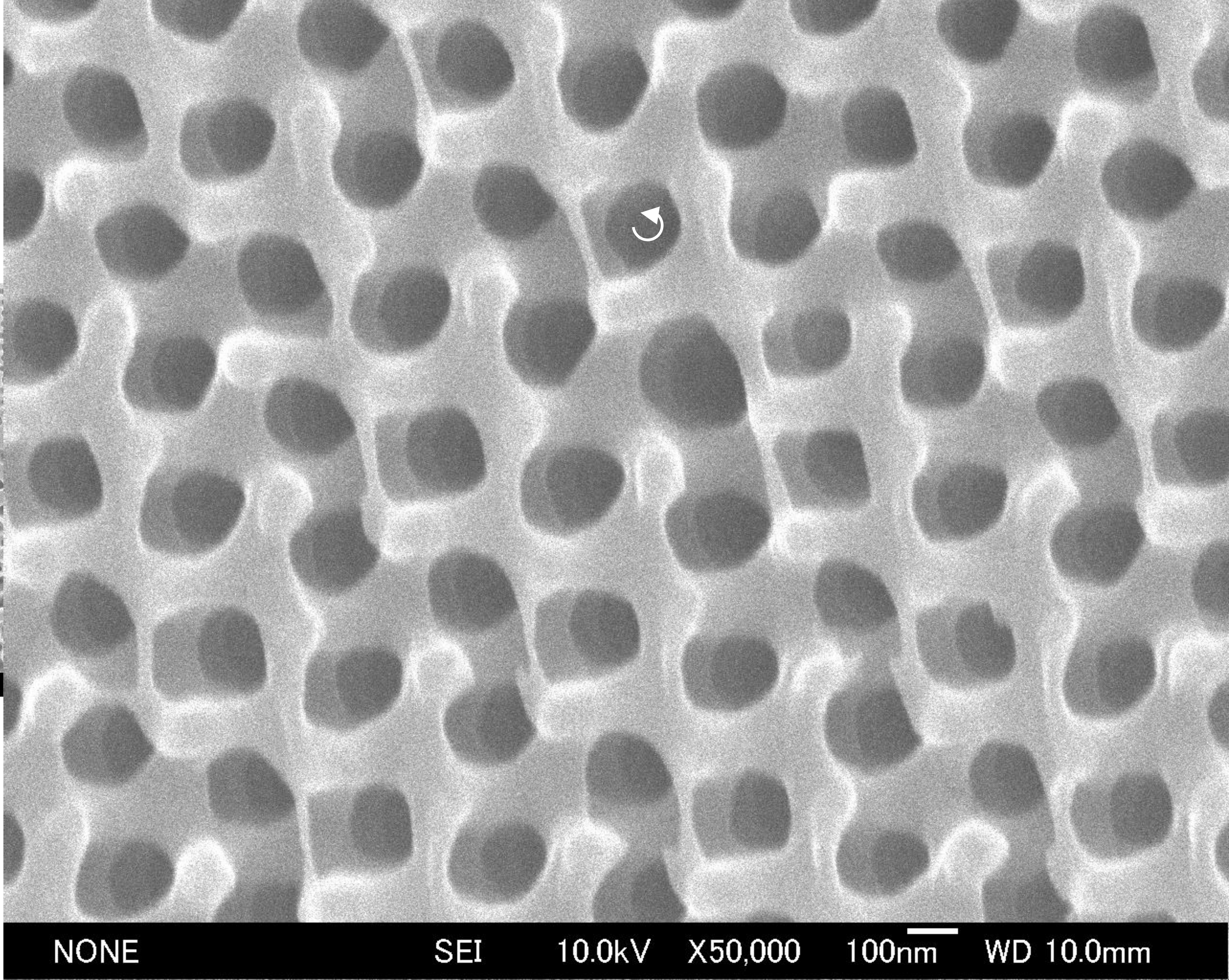

specimen No. 1
scale No. 2
domain No. 12
LH

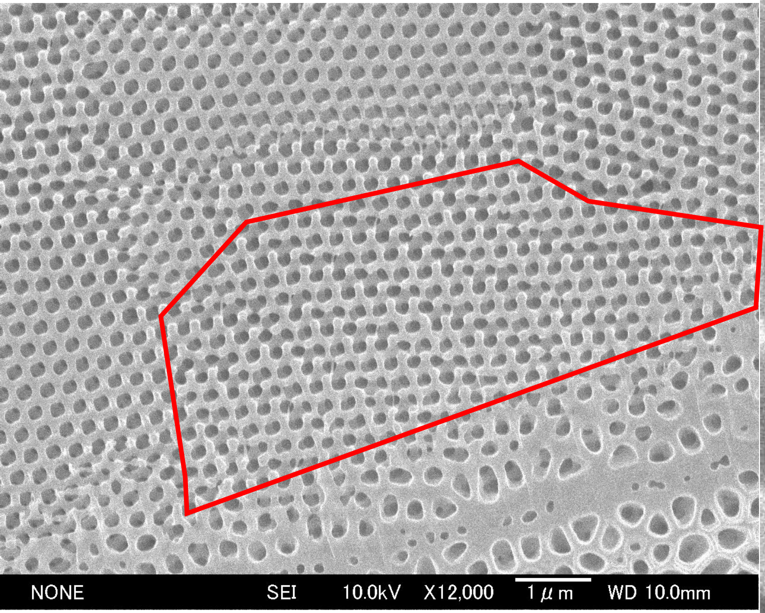

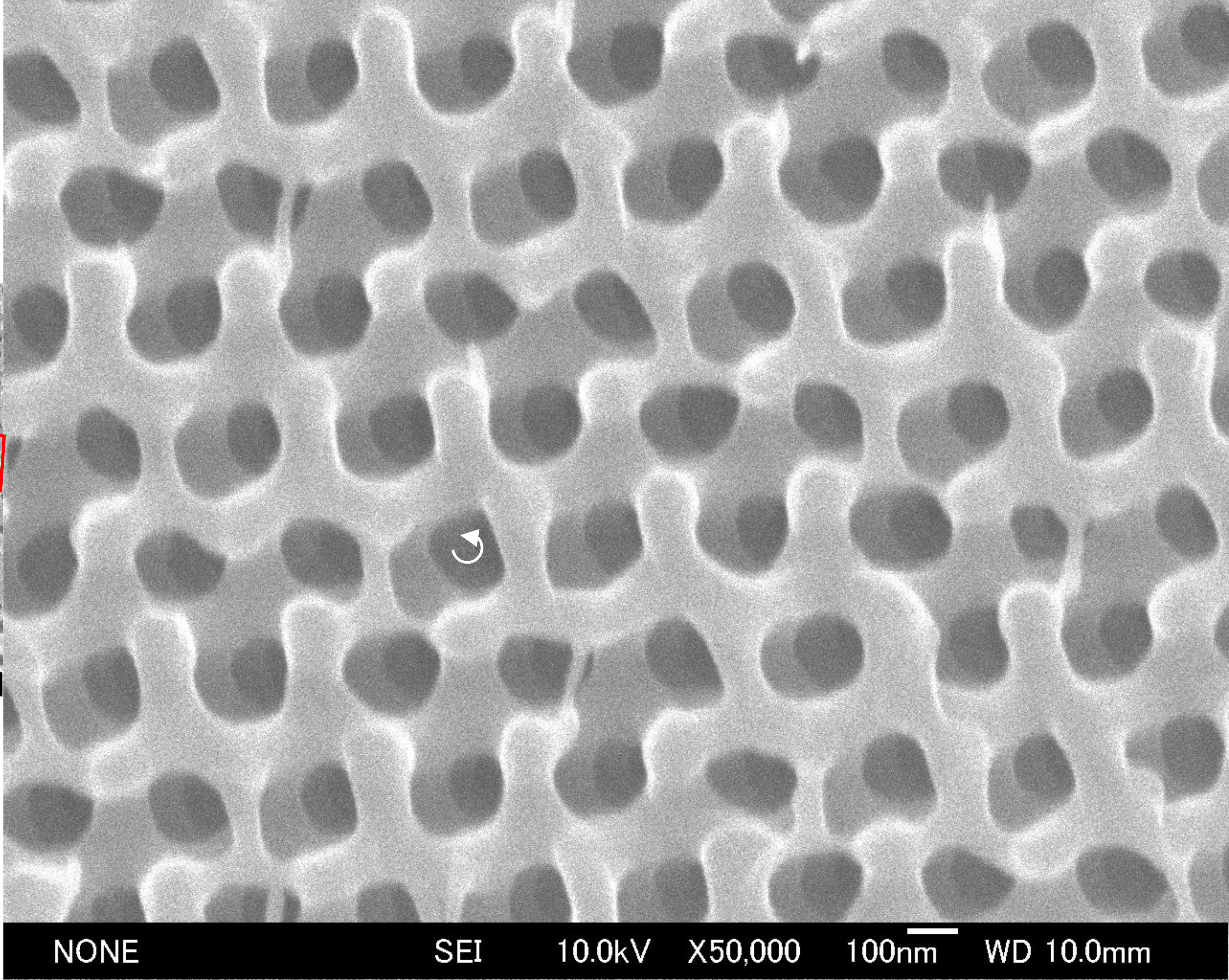

specimen No. 1
scale No. 2
domain No. 13
LH

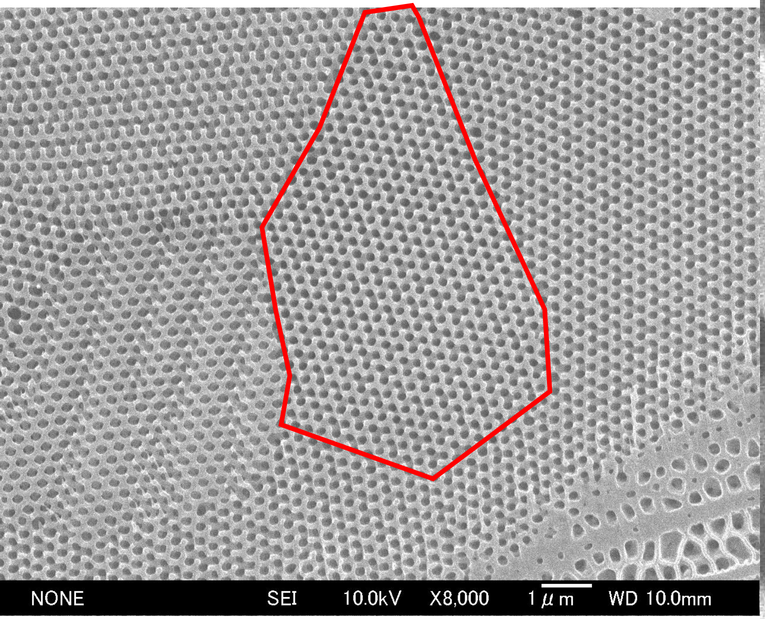

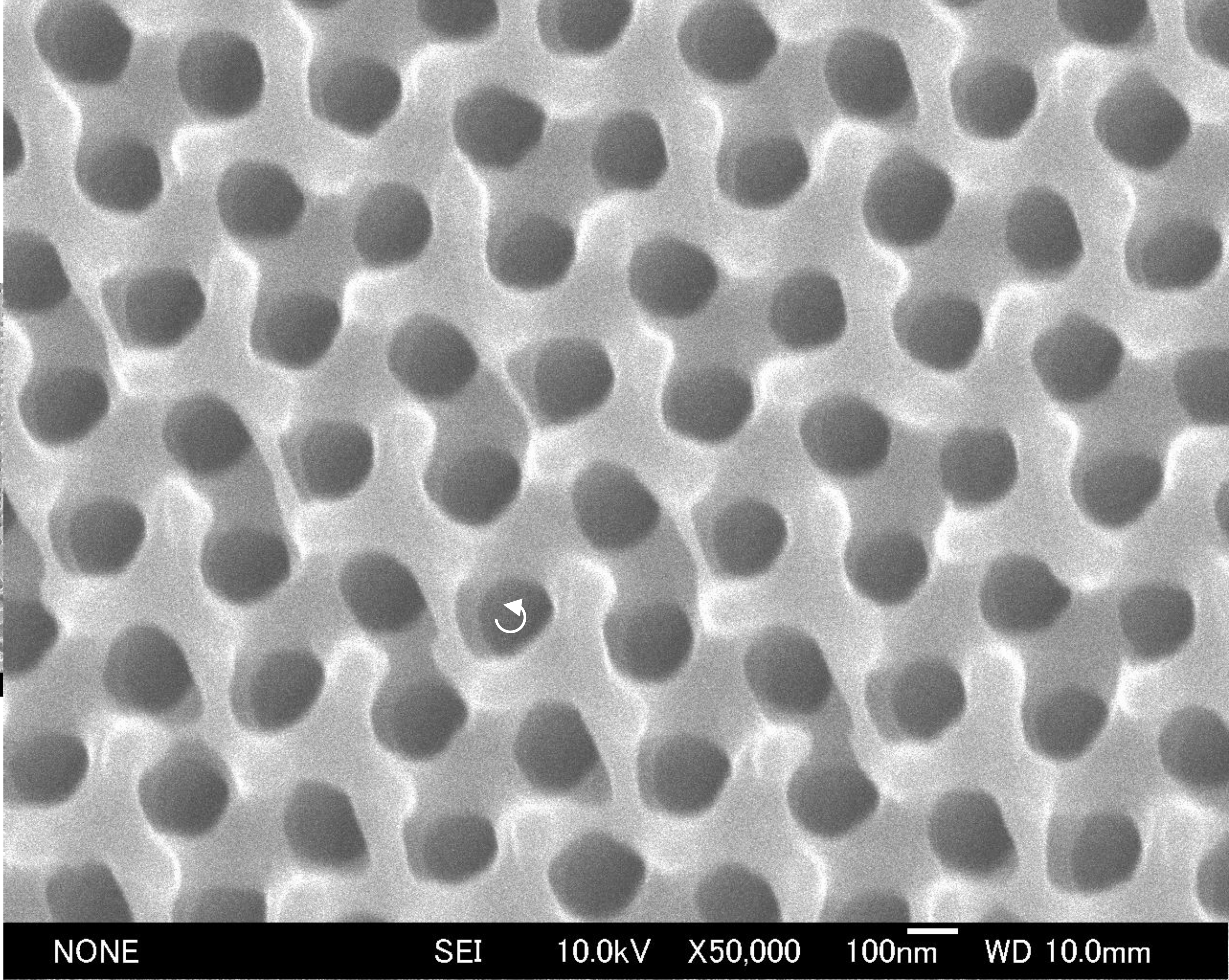

specimen No. 1
scale No. 2
domain No. 14
LH

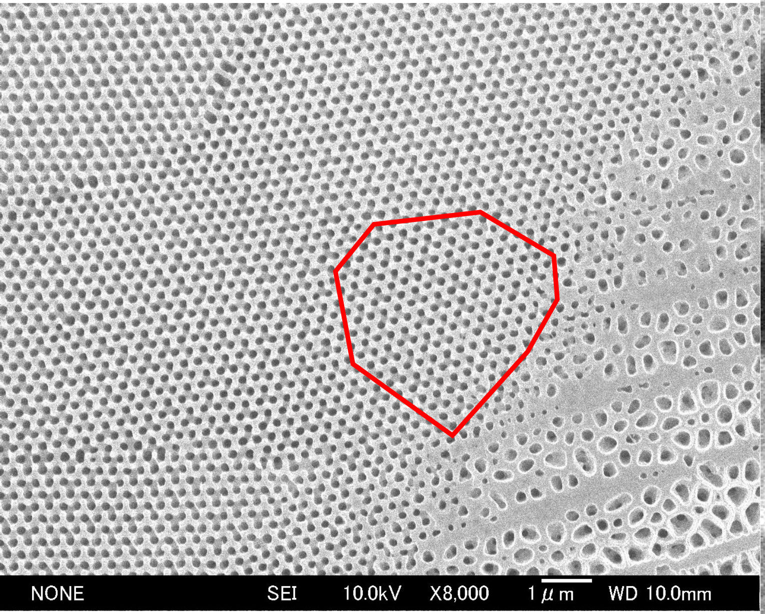

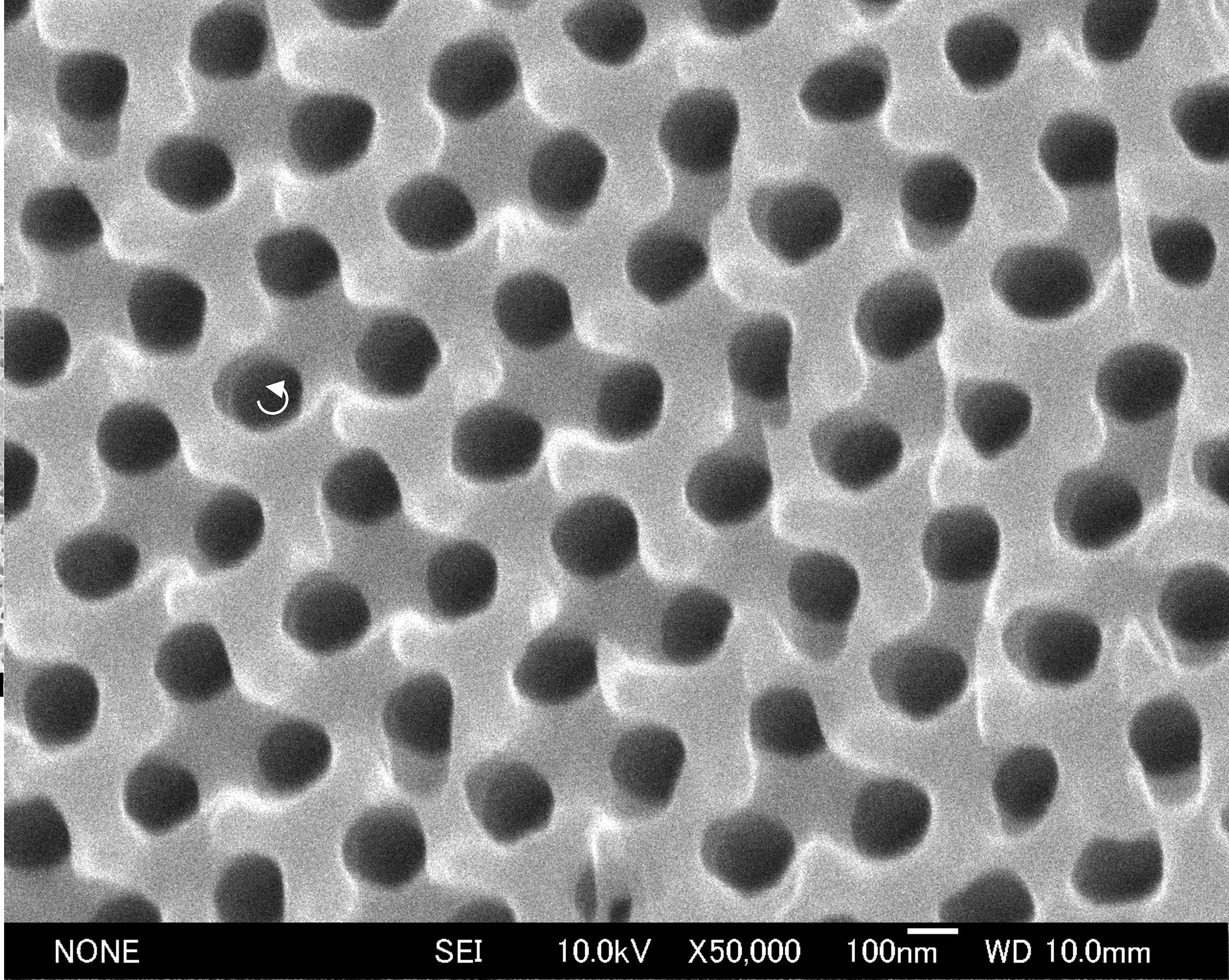

specimen No. 1
scale No. 2
domain No. 15
LH

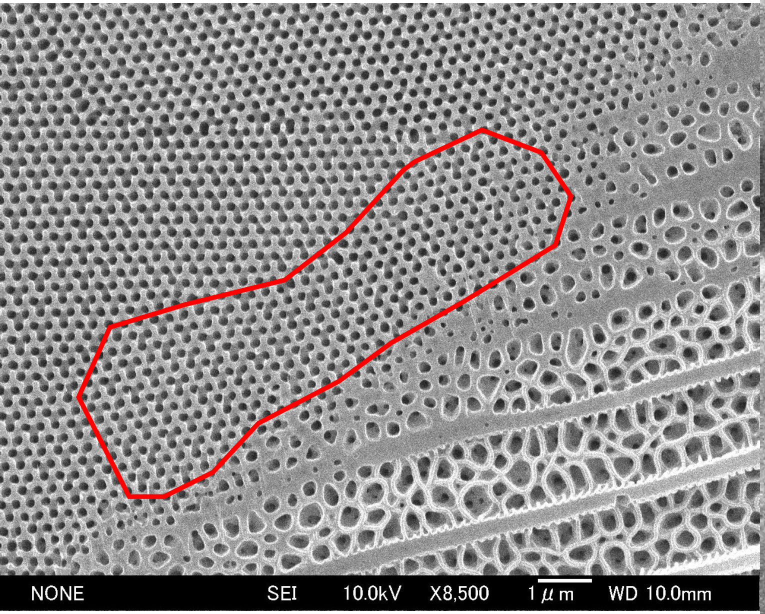

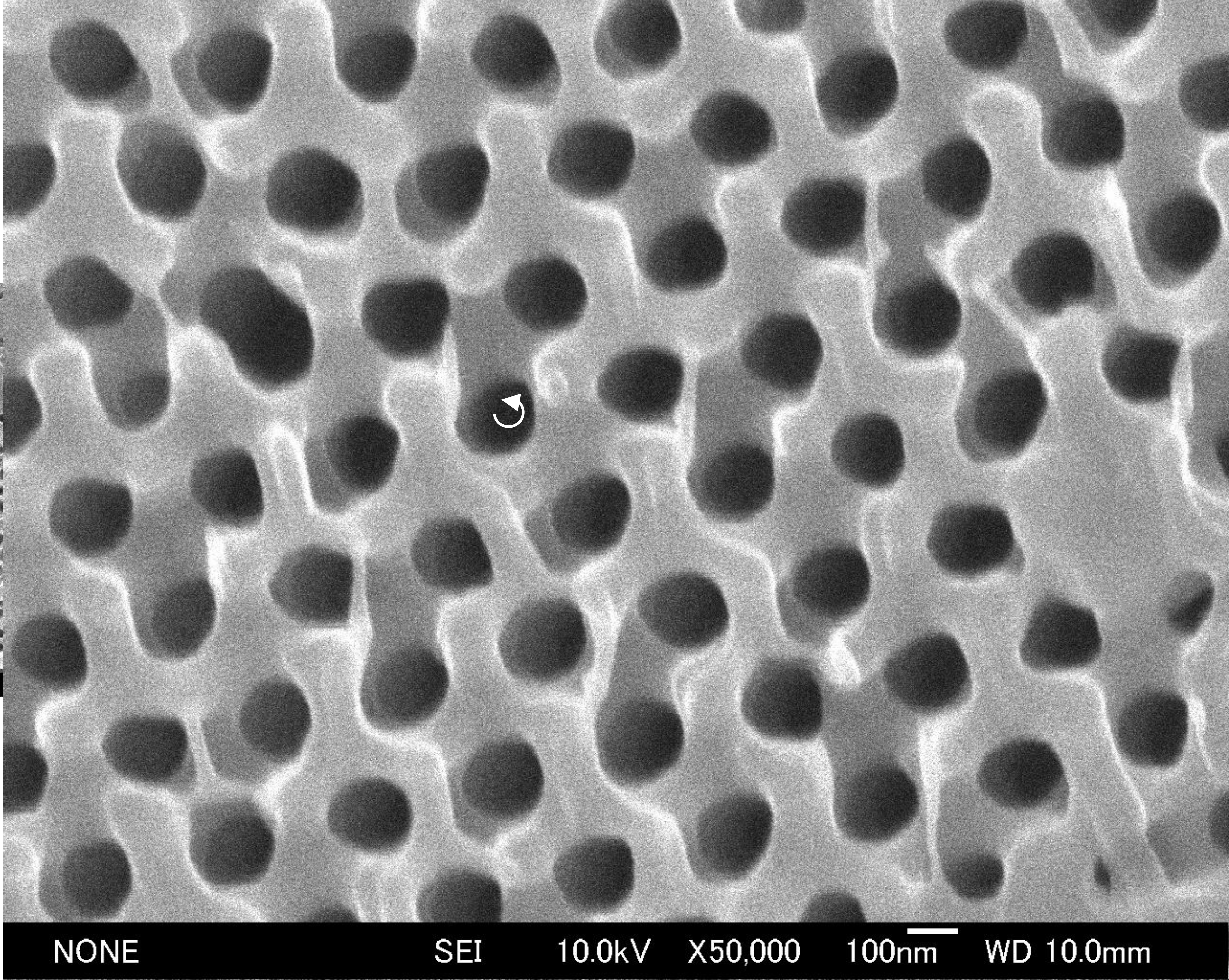

specimen No. 1
scale No. 2
domain No. 16
LH

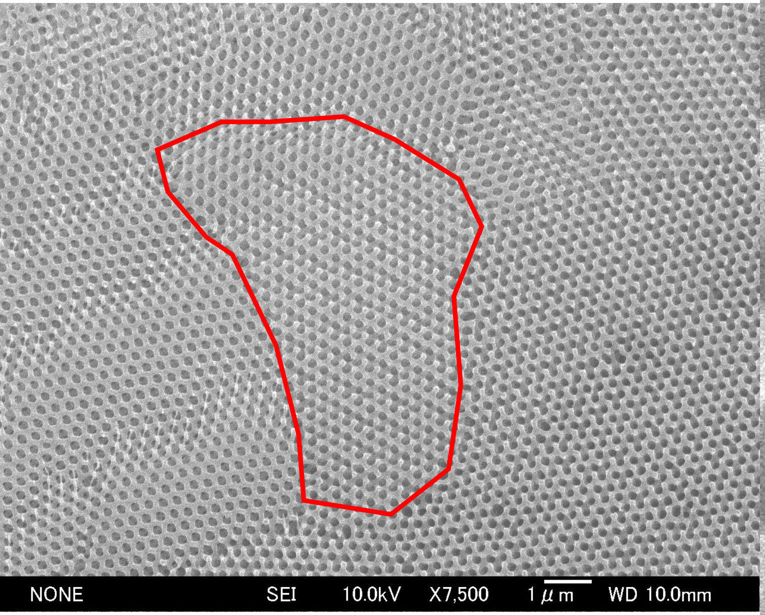

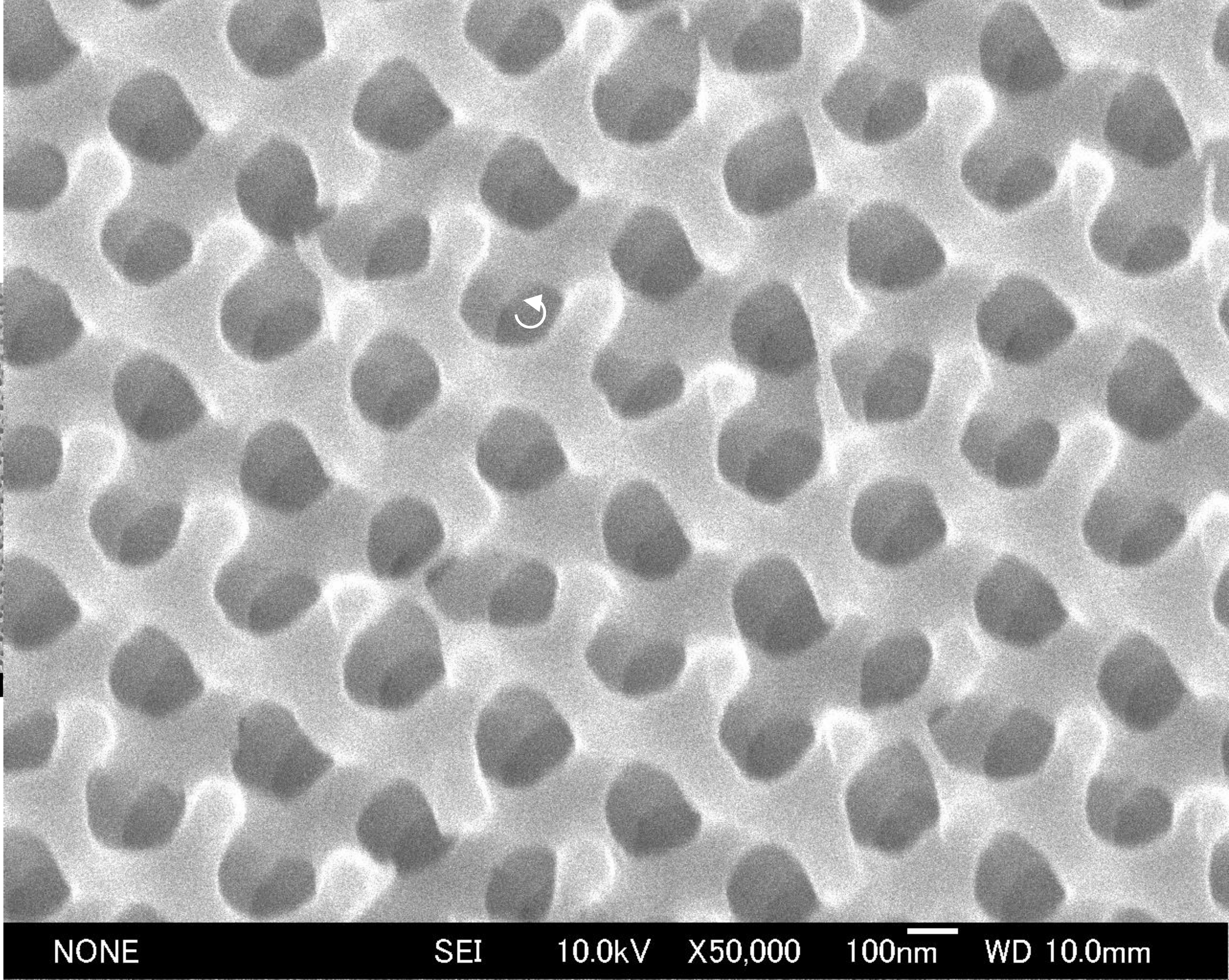

specimen No. 1
scale No. 2
domain No. 17
LH

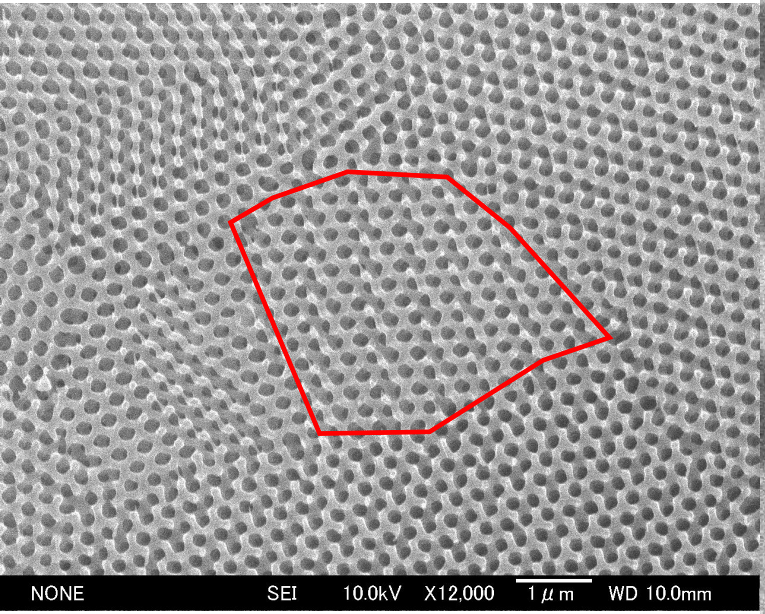

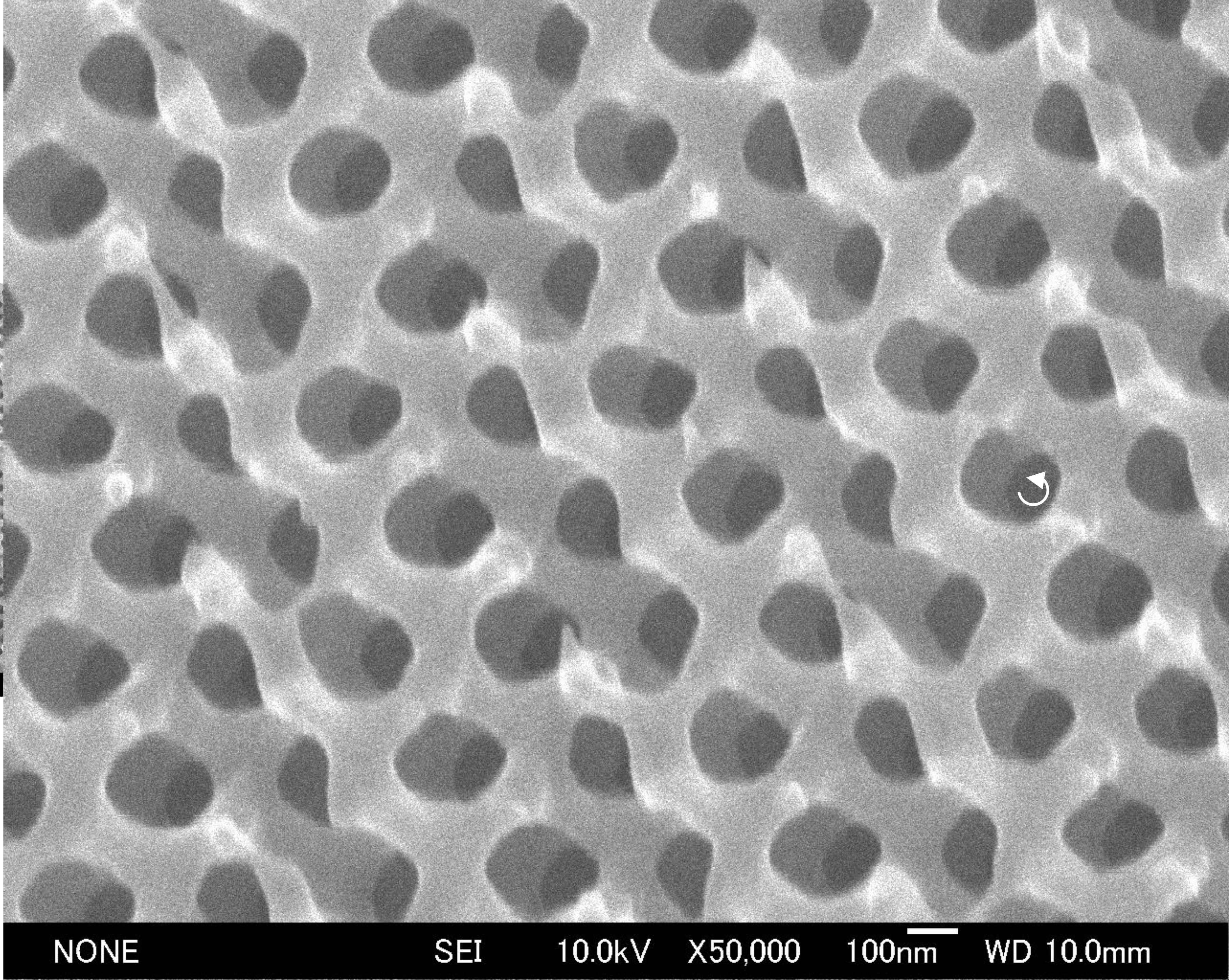

specimen No. 1
scale No. 2
domain No. 18 ①
LH

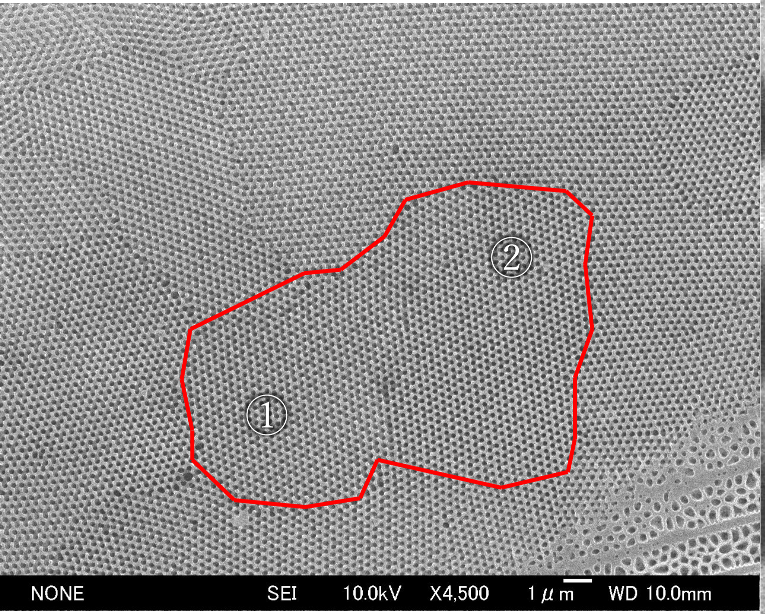

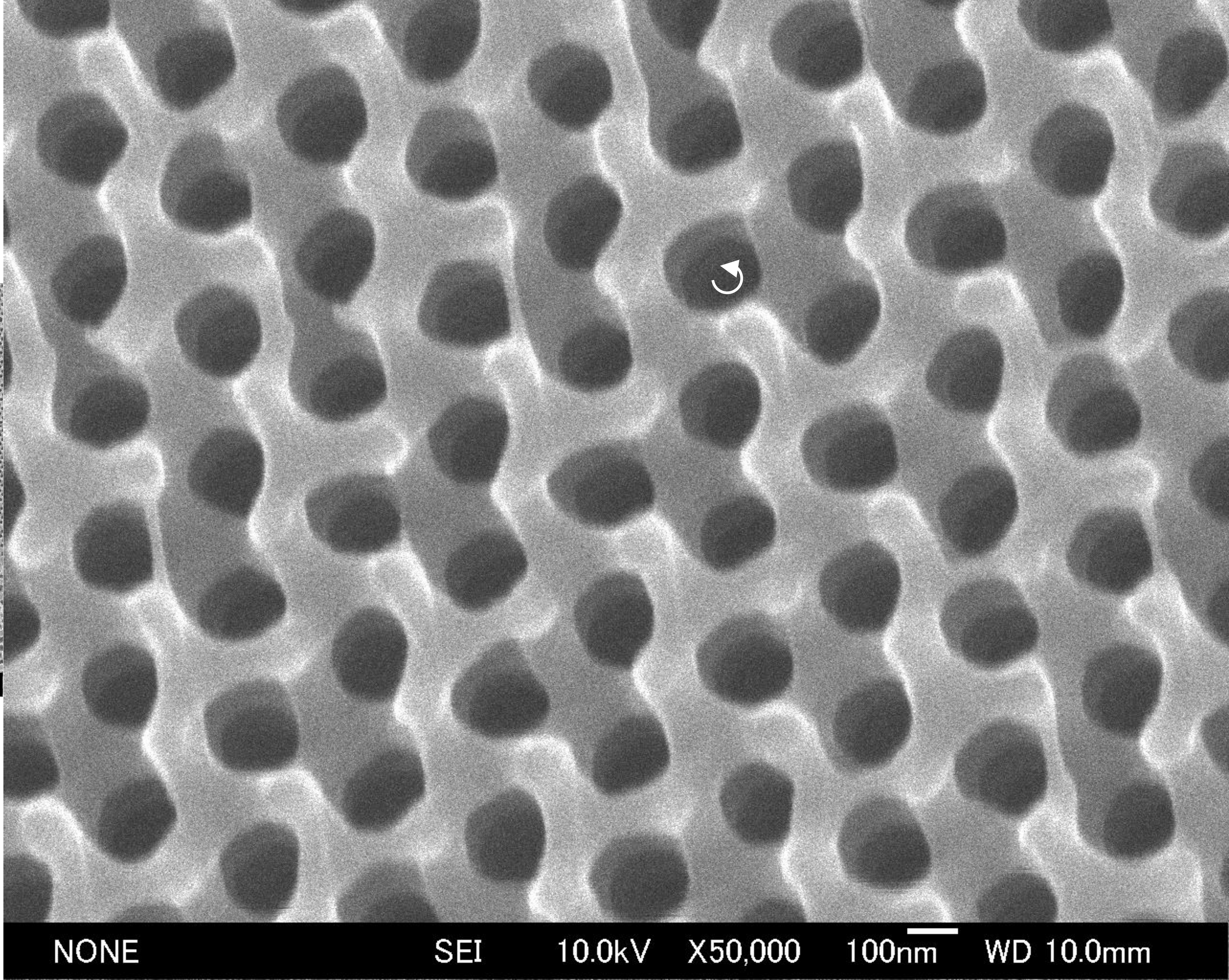

specimen No. 1
scale No. 2
domain No. 18 ②
LH

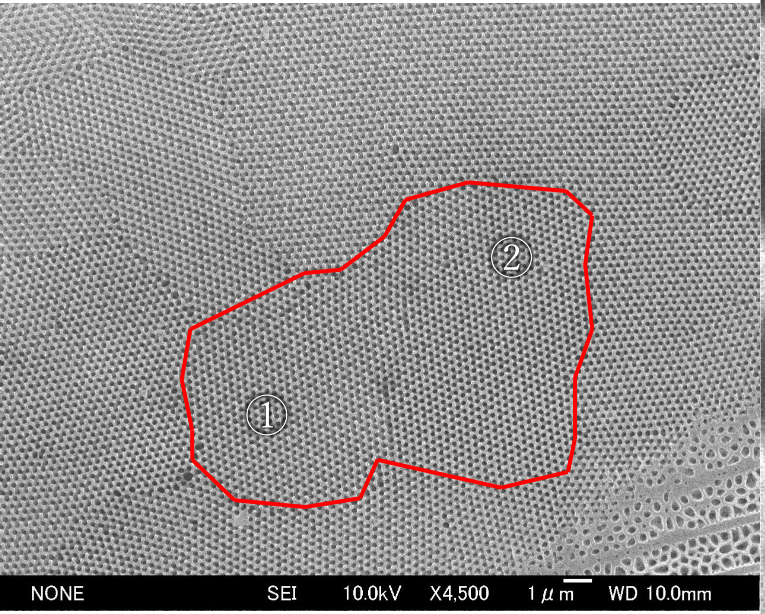

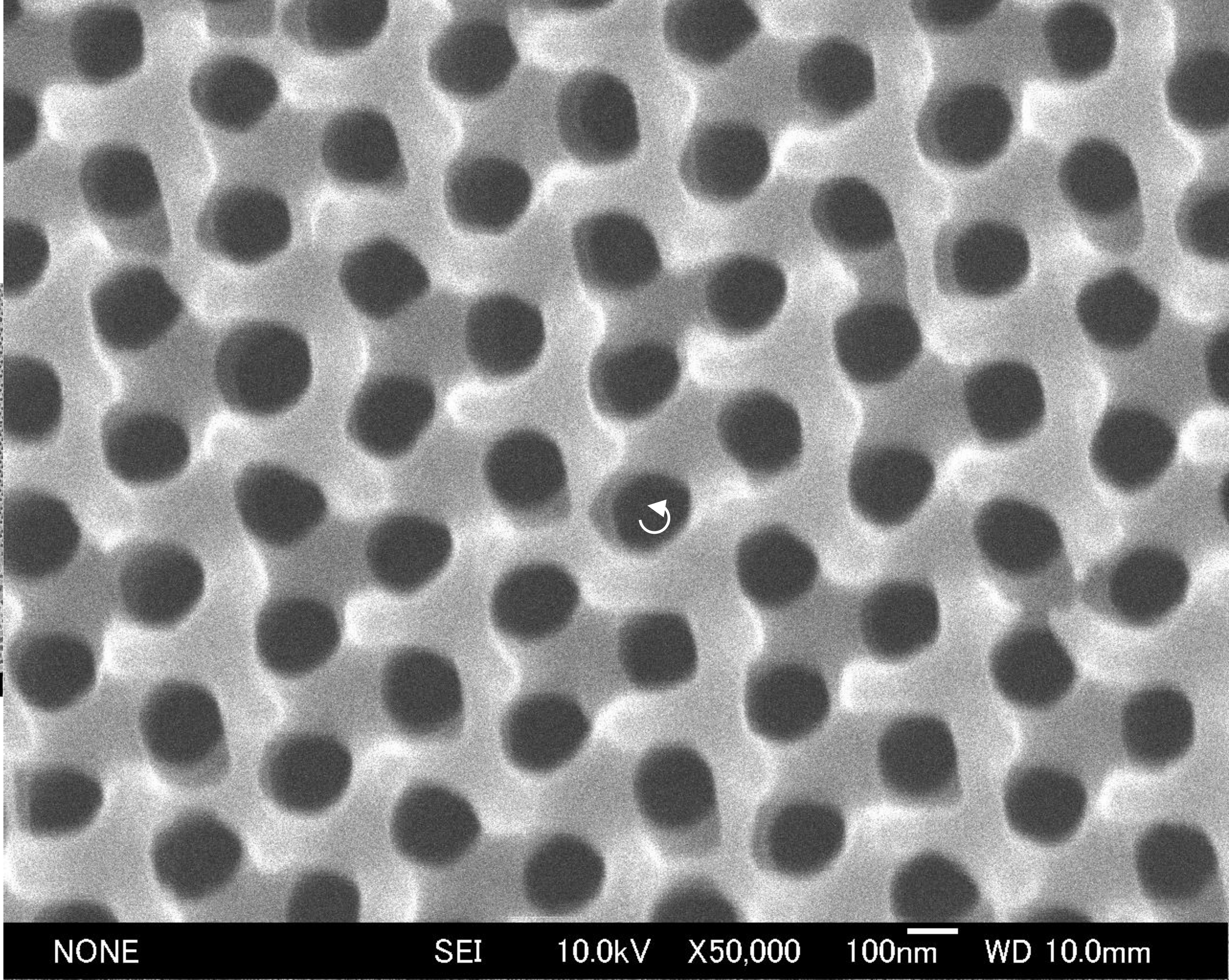

specimen No. 1
scale No. 2
domain No. 19
LH

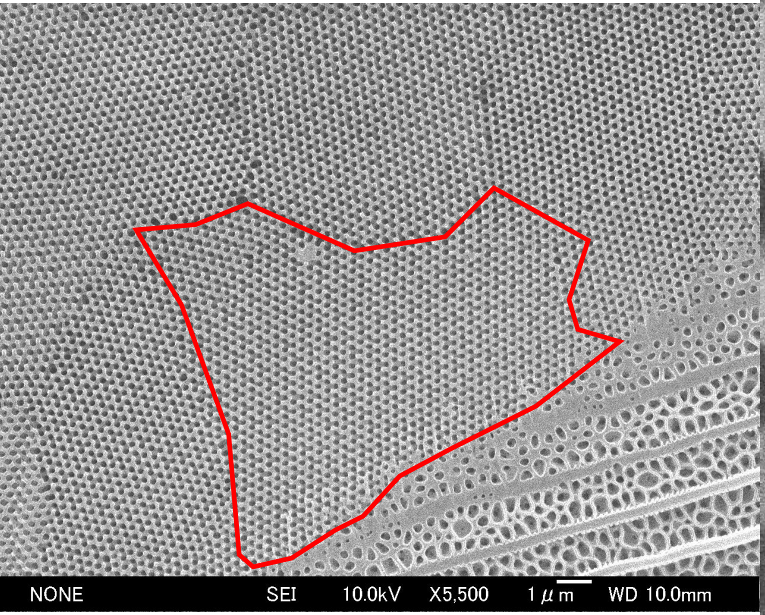

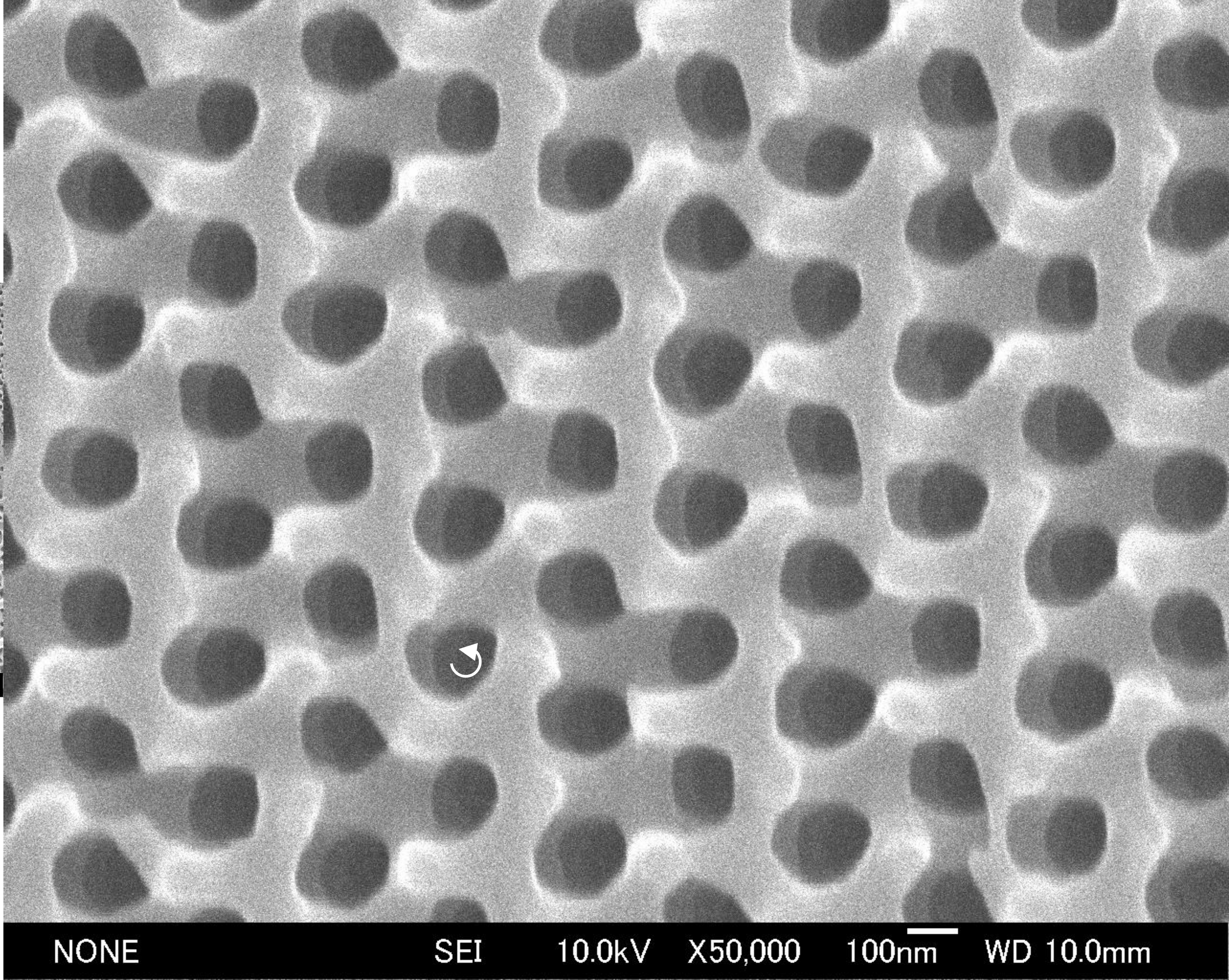

specimen No. 1
scale No. 2
domain No. 20
LH

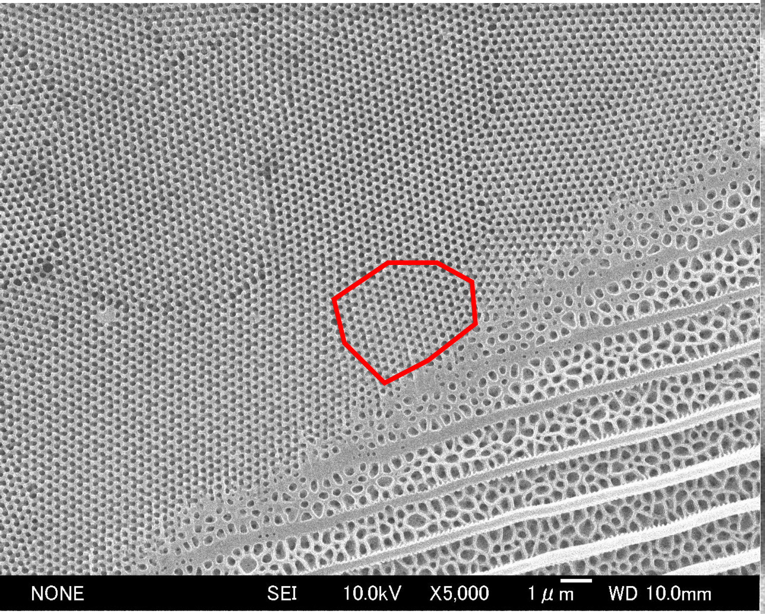

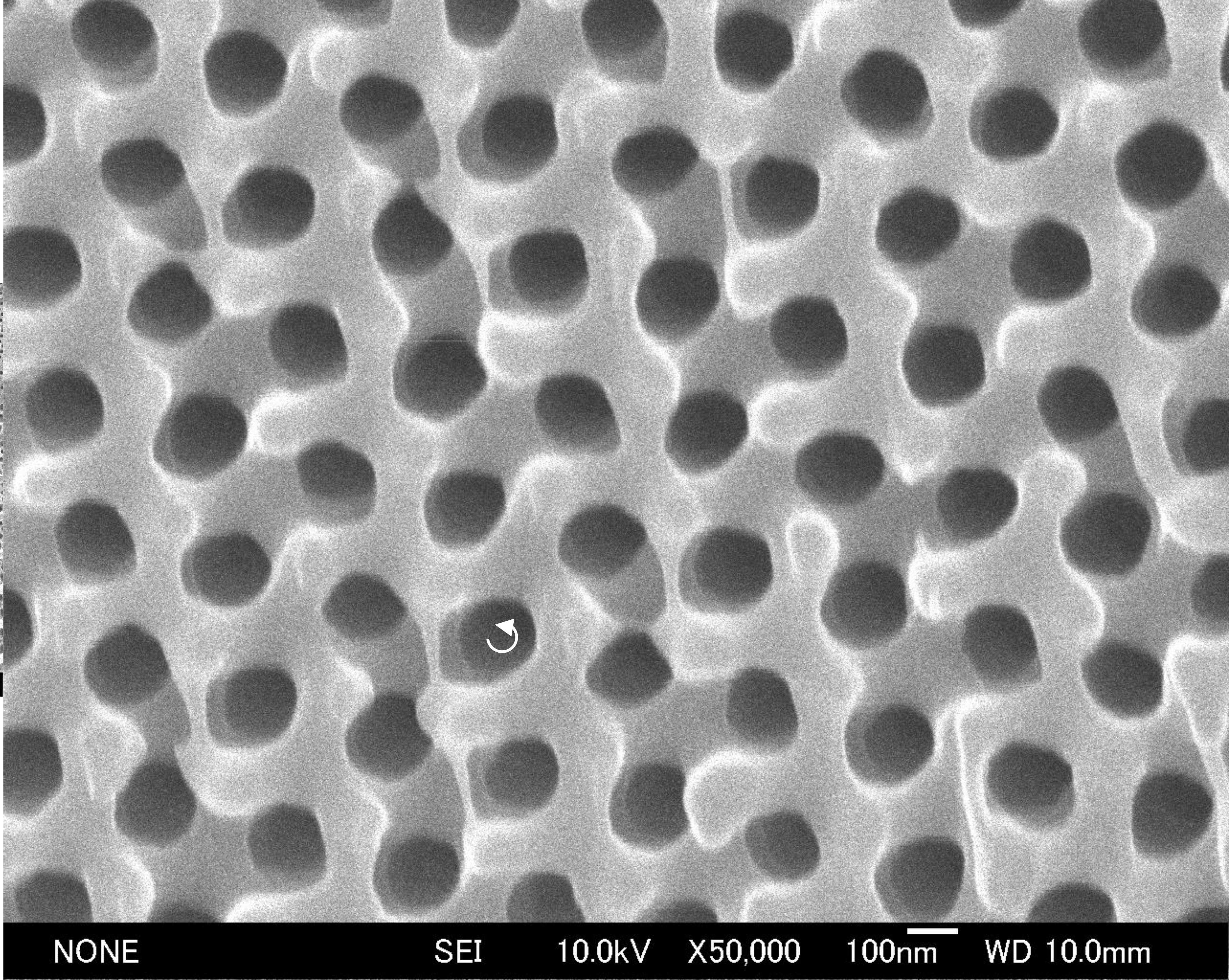

specimen No. 1
scale No. 2
domain No. 21 ①
LH

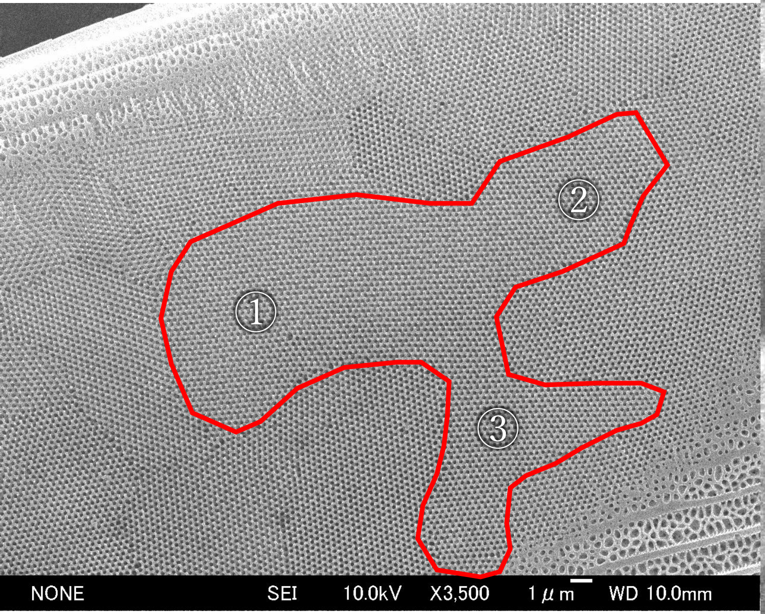

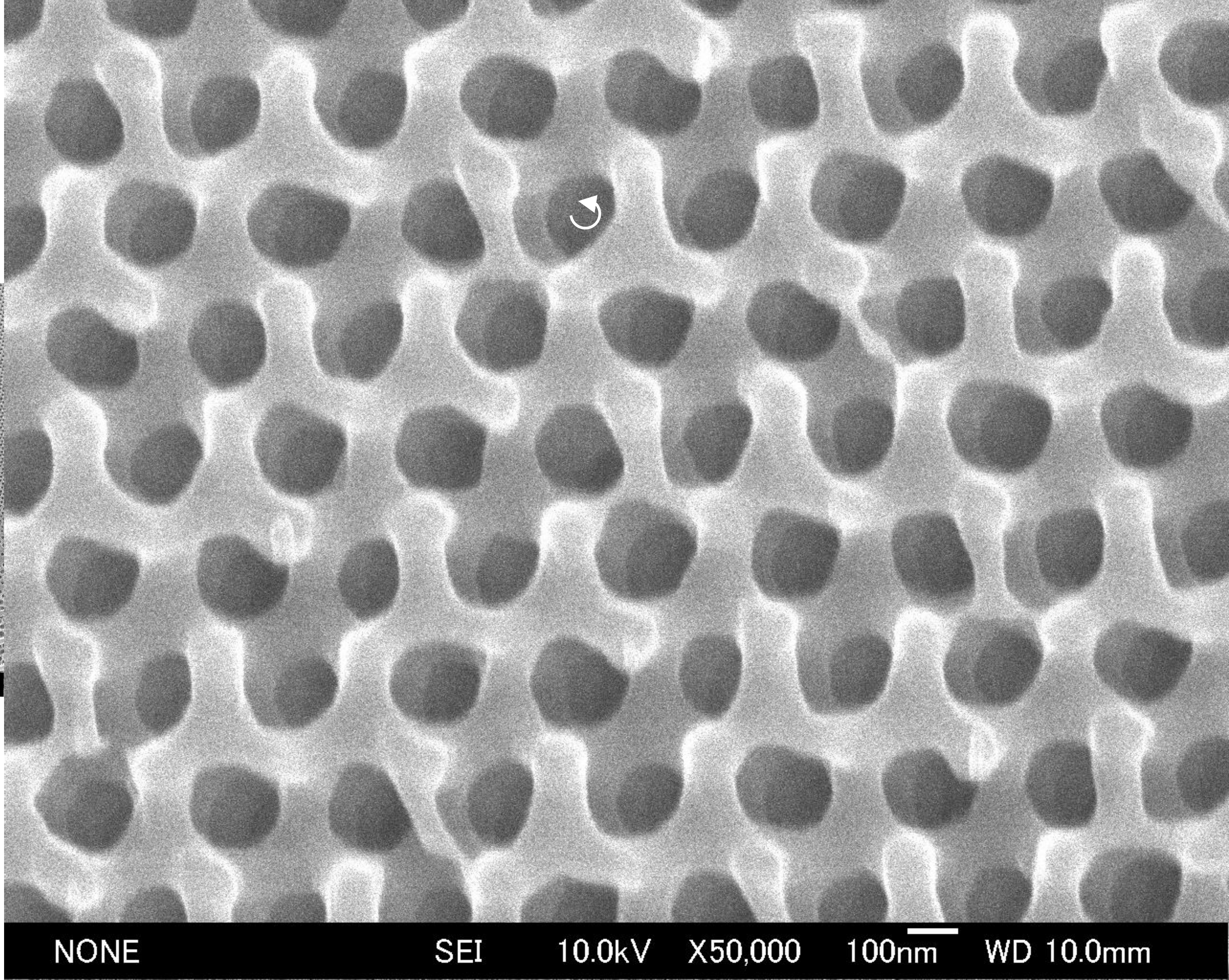

specimen No. 1
scale No. 2
domain No. 21 ②
LH

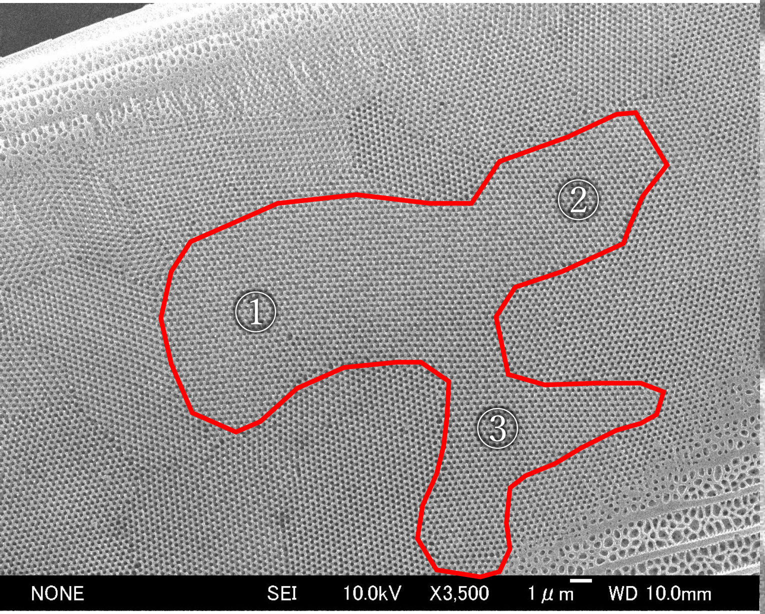

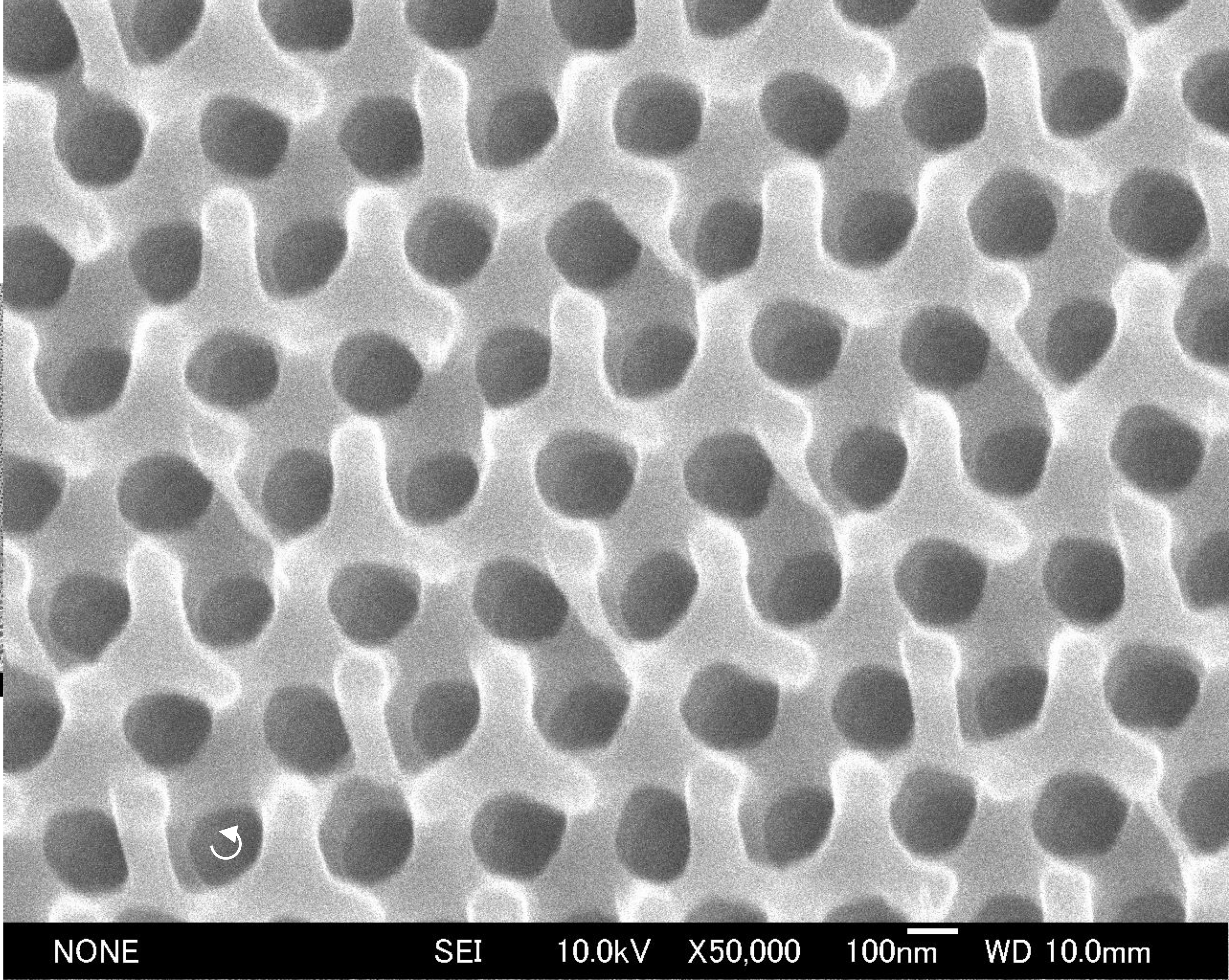

specimen No. 1
scale No. 2
domain No. 21 ③
LH

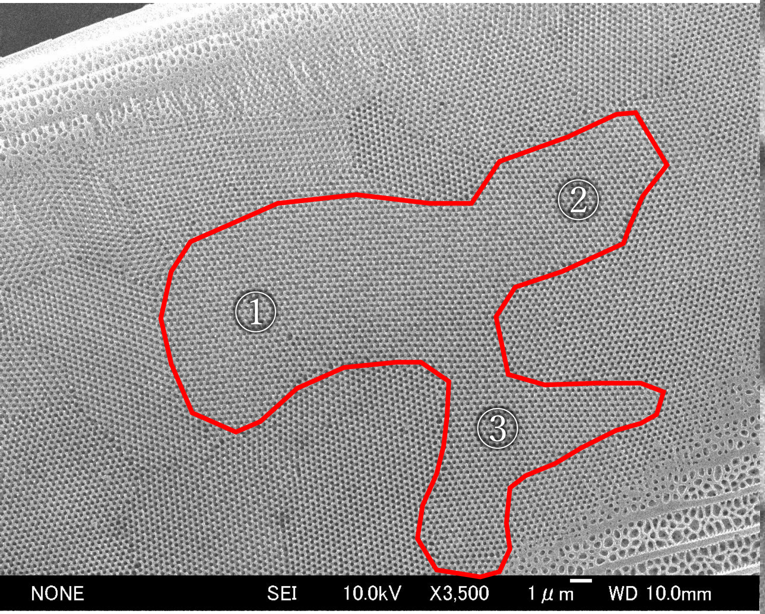

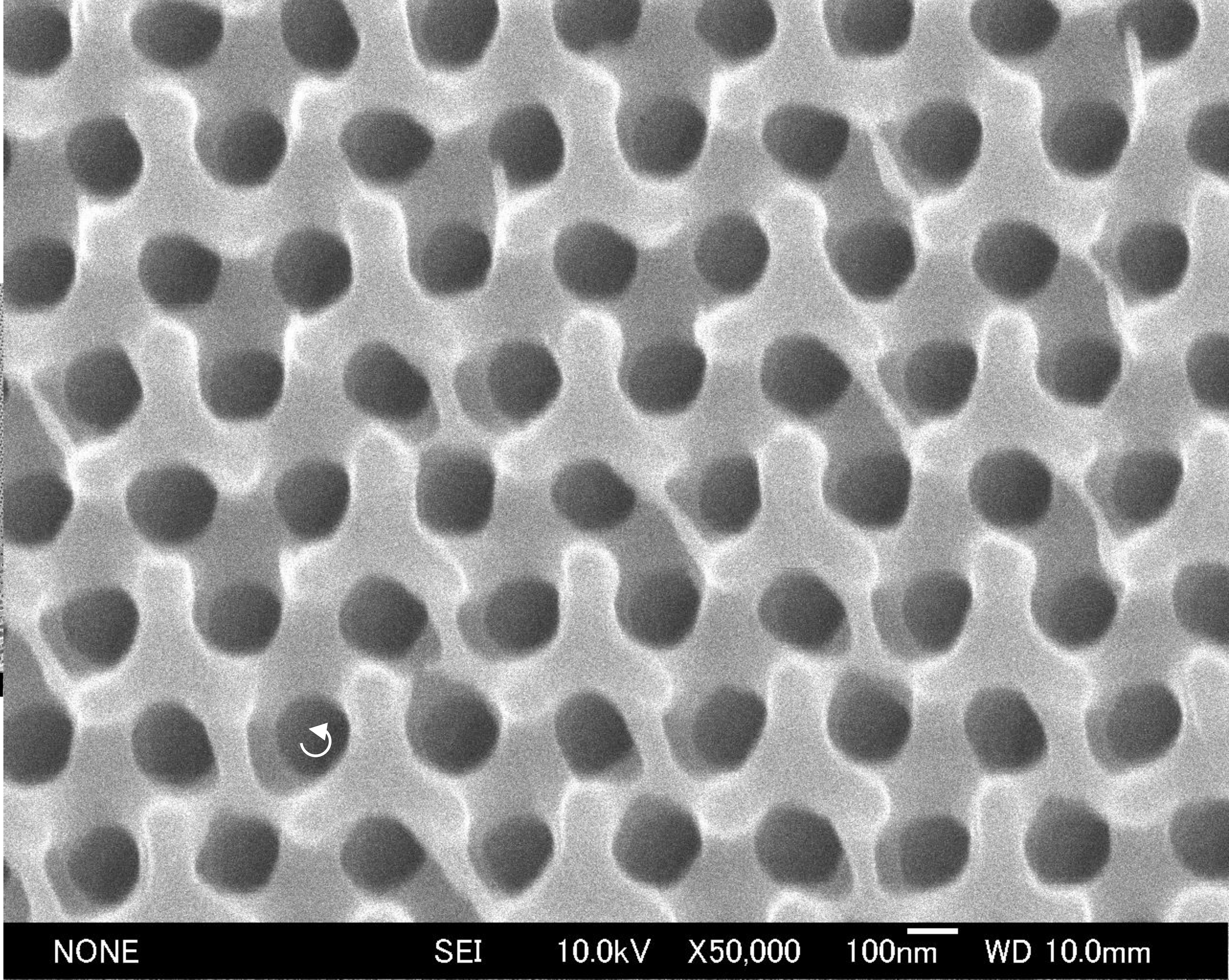

specimen No. 1
scale No. 2
domain No. 22
LH

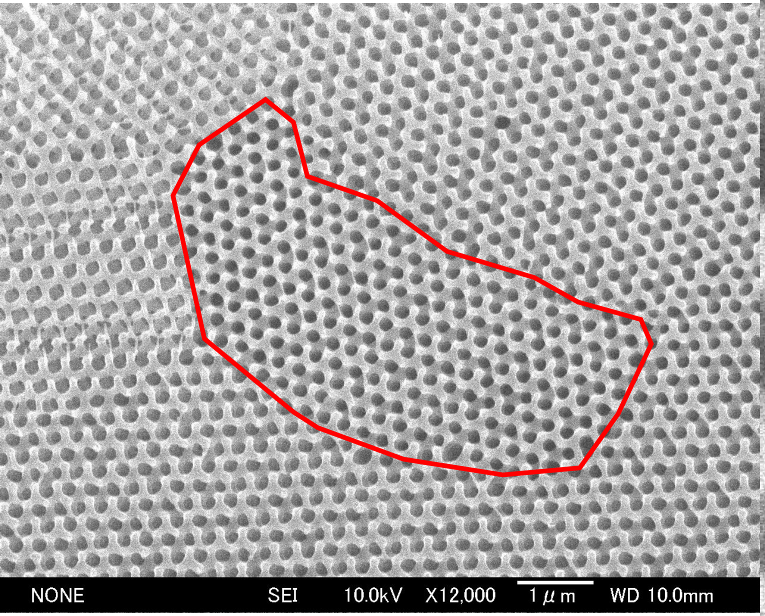

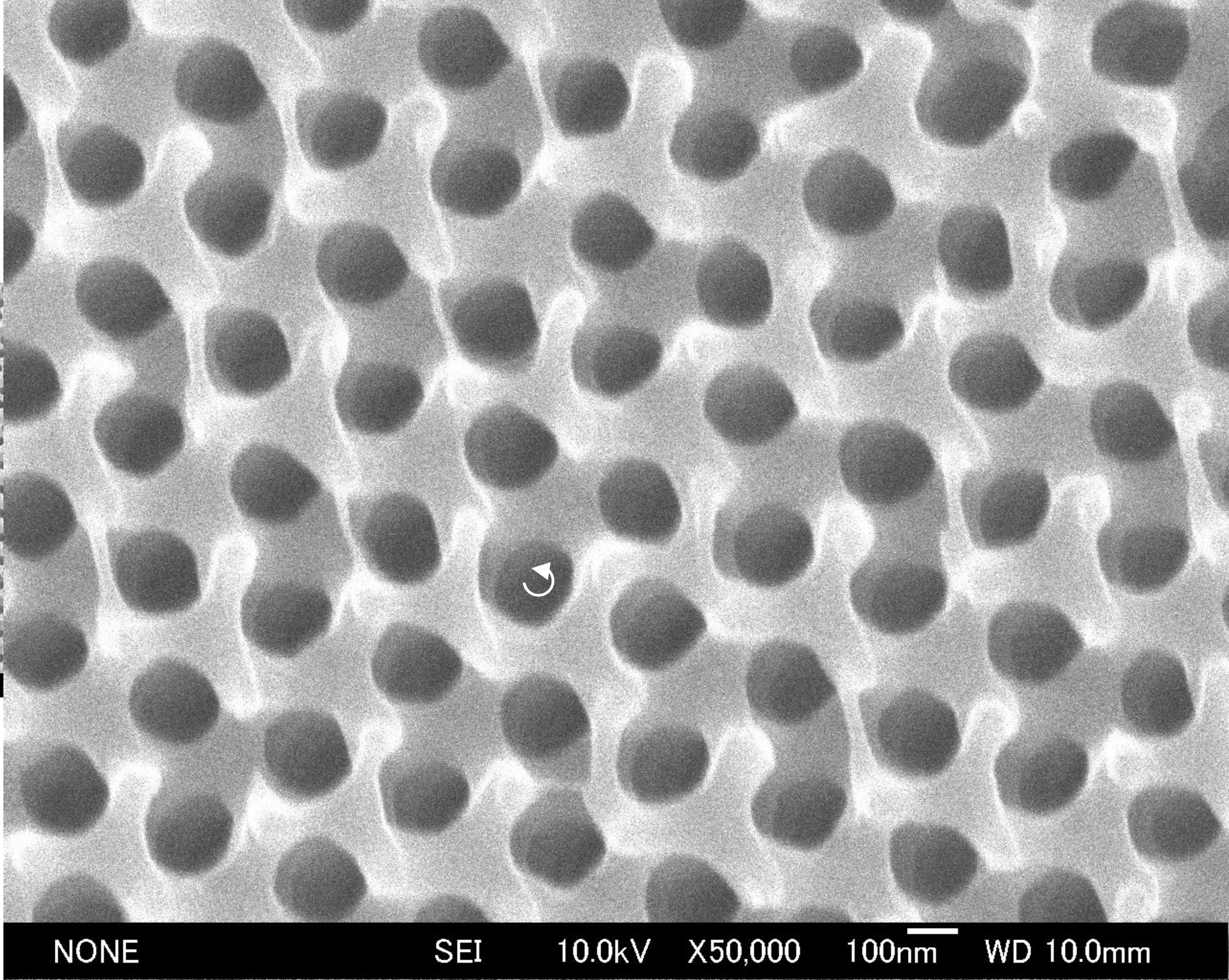

specimen No. 1
scale No. 2
domain No. 23
LH

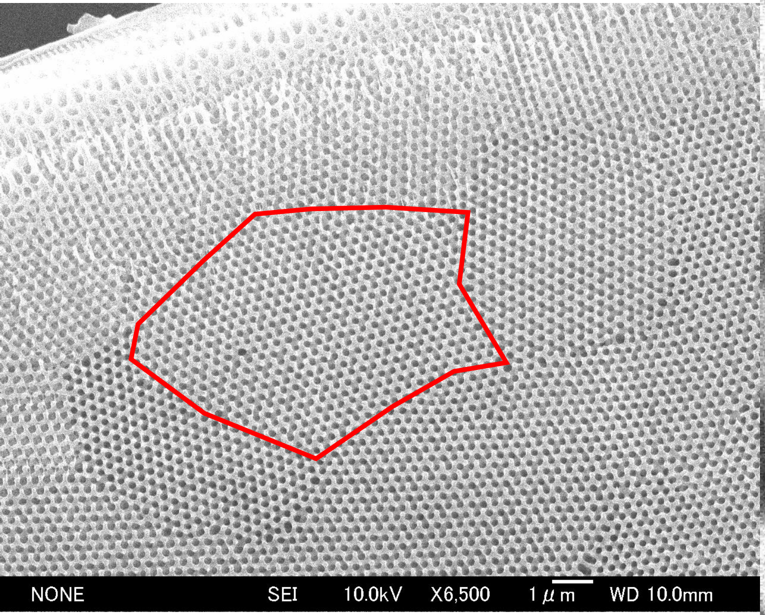

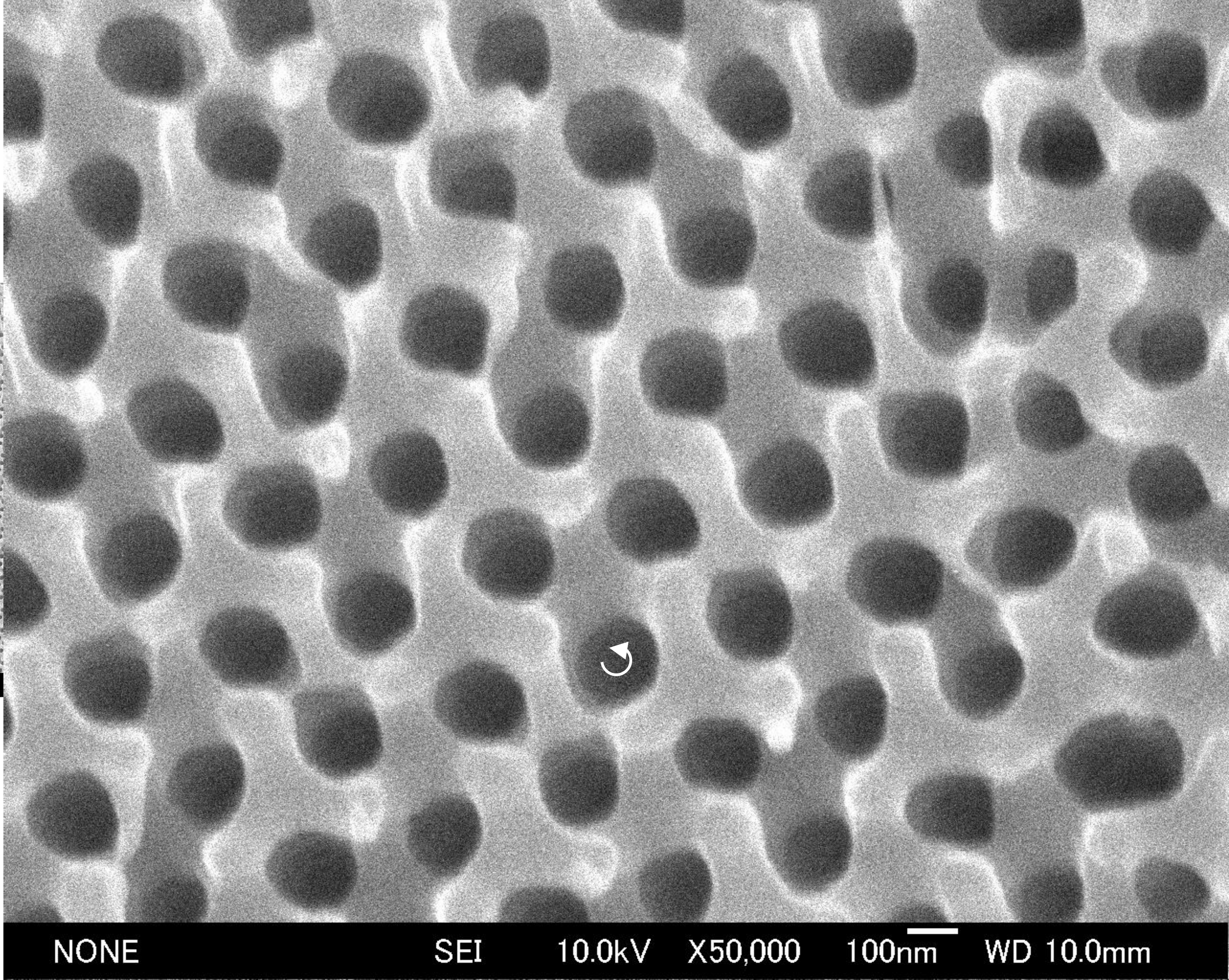

specimen No. 1
scale No. 2
domain No. 24
LH

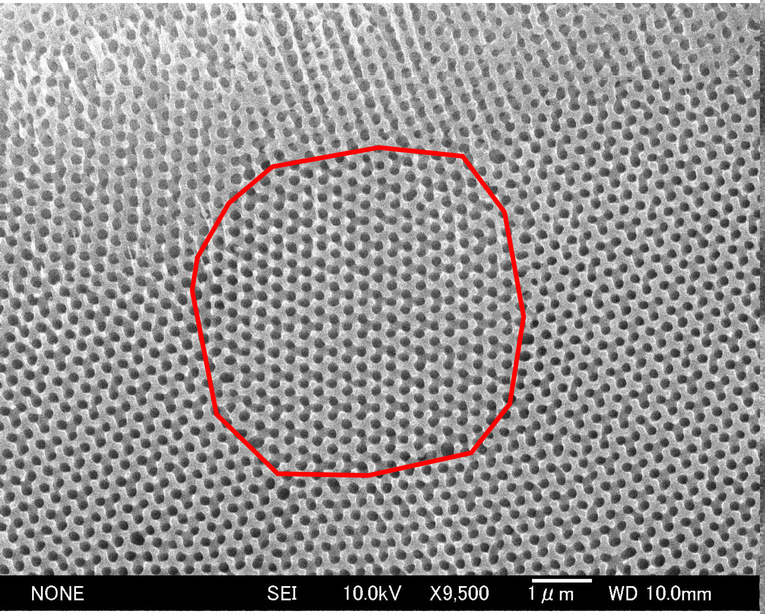

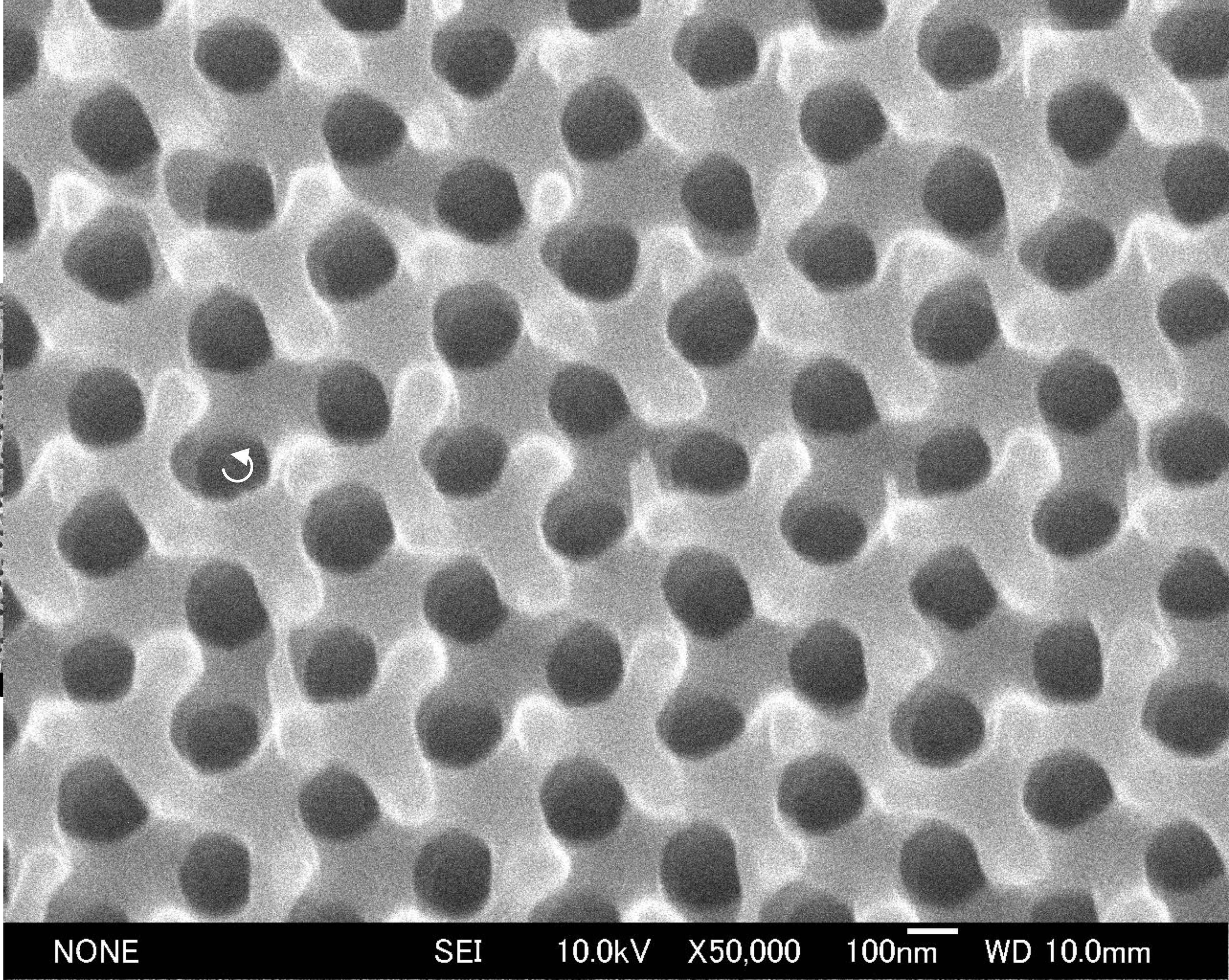

specimen No. 1
scale No. 2
domain No. 25
LH

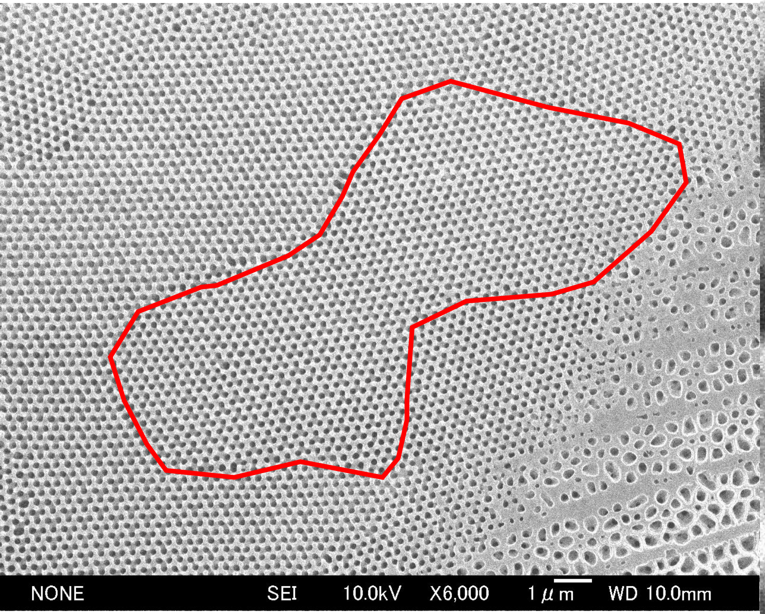

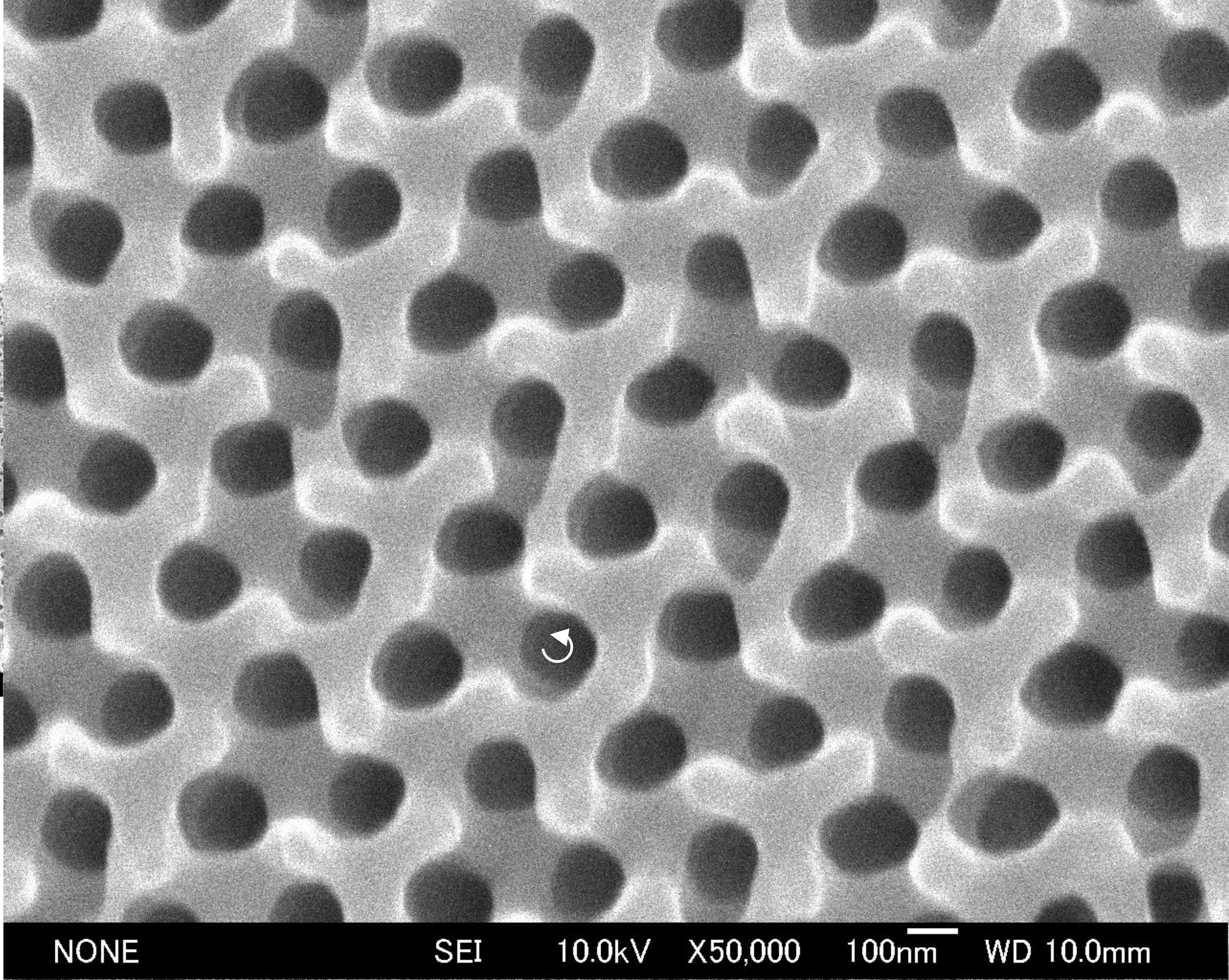

specimen No. 1
scale No. 2
domain No. 26
LH

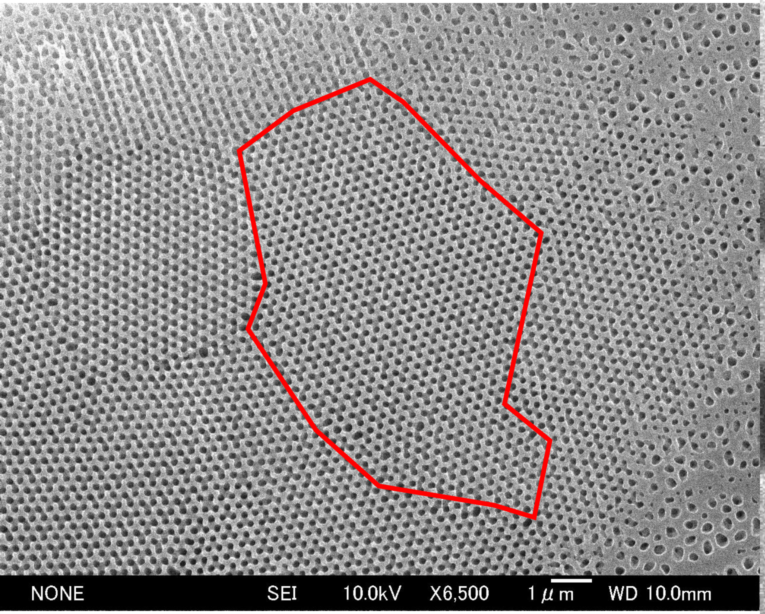

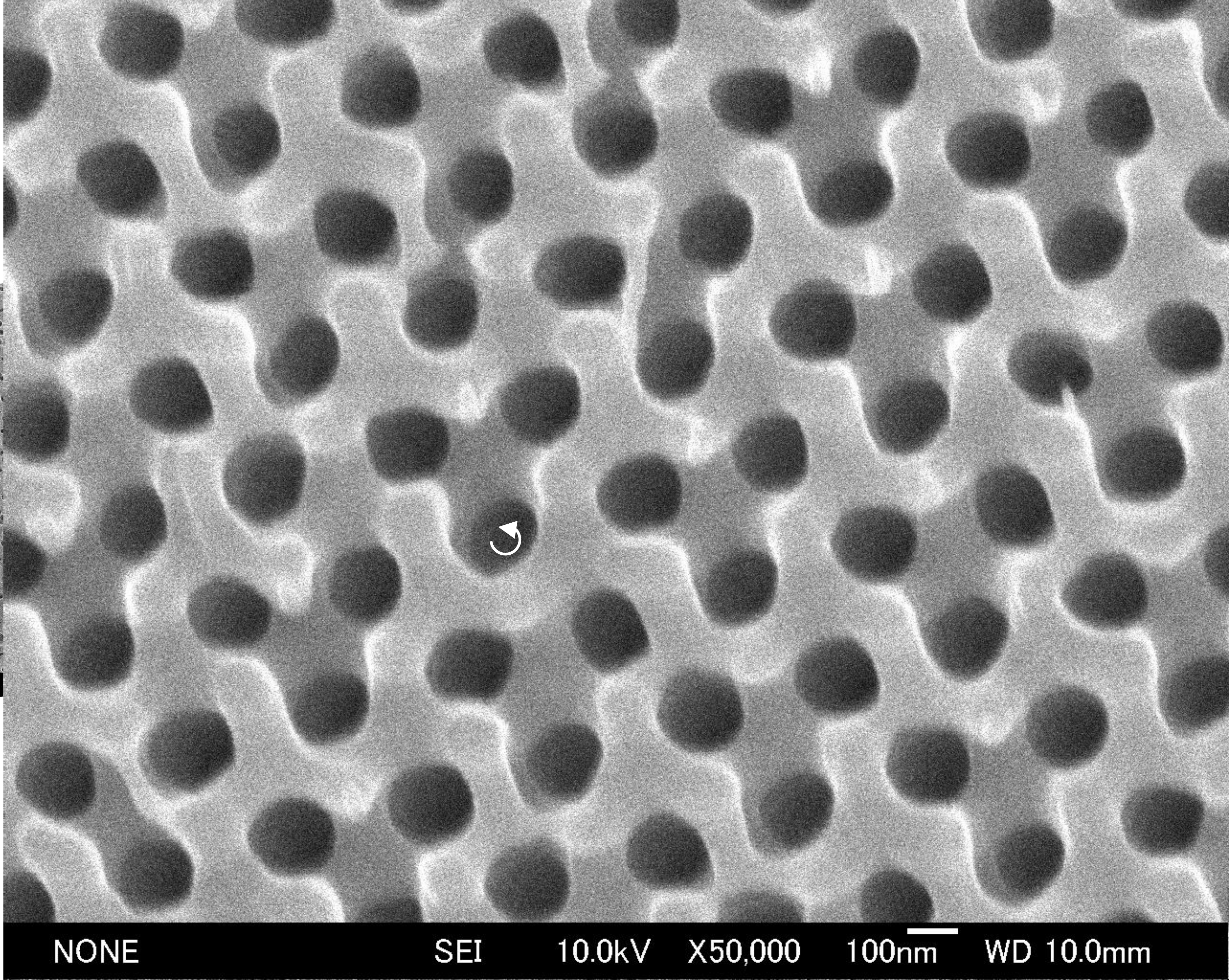

specimen No. 1
scale No. 2
domain No. 27
LH

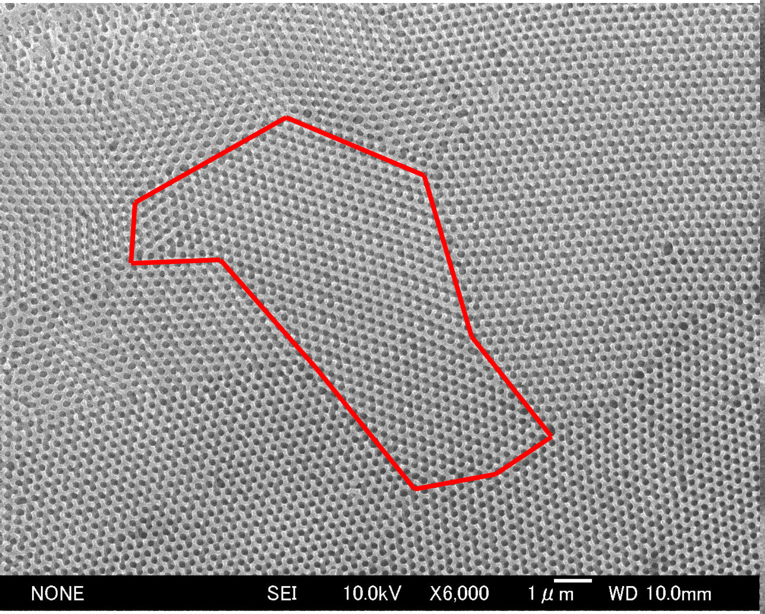

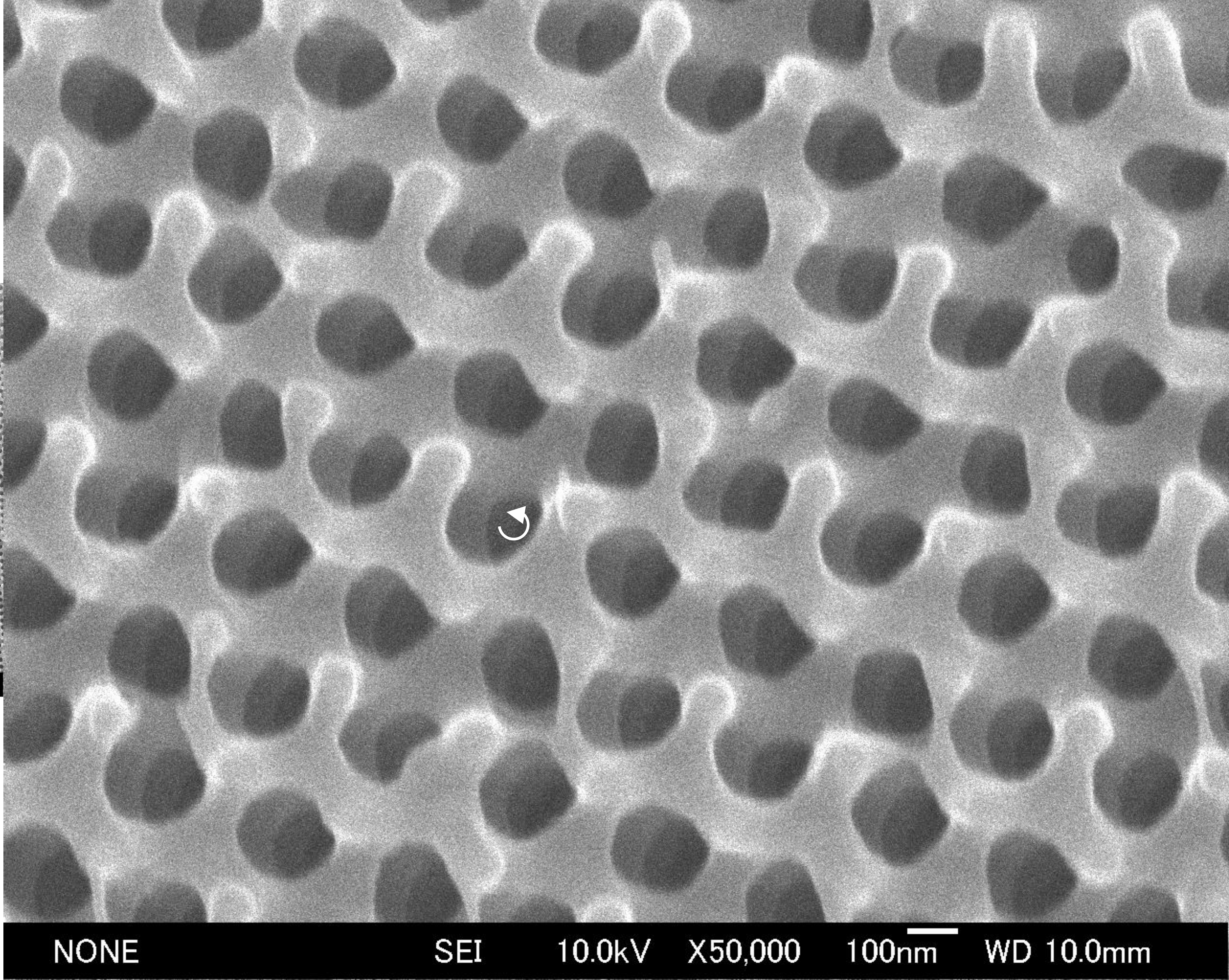

specimen No. 1
scale No. 2
domain No. 28
LH

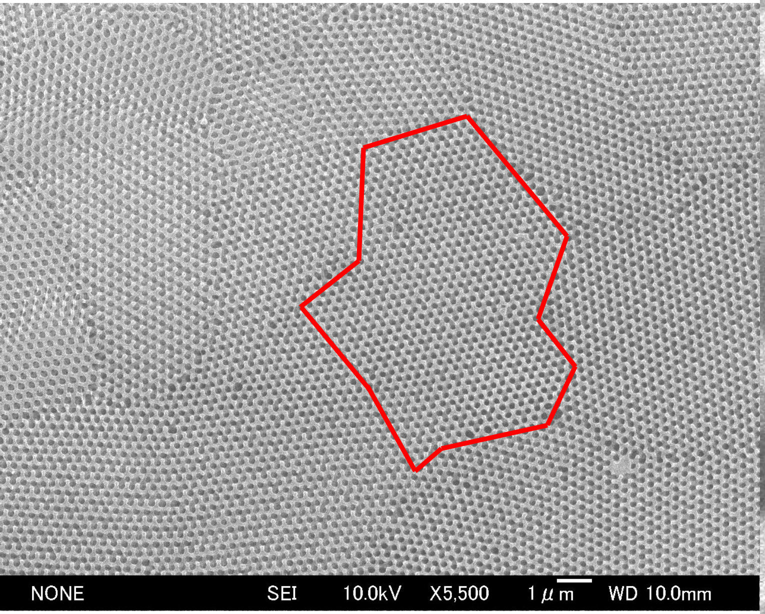

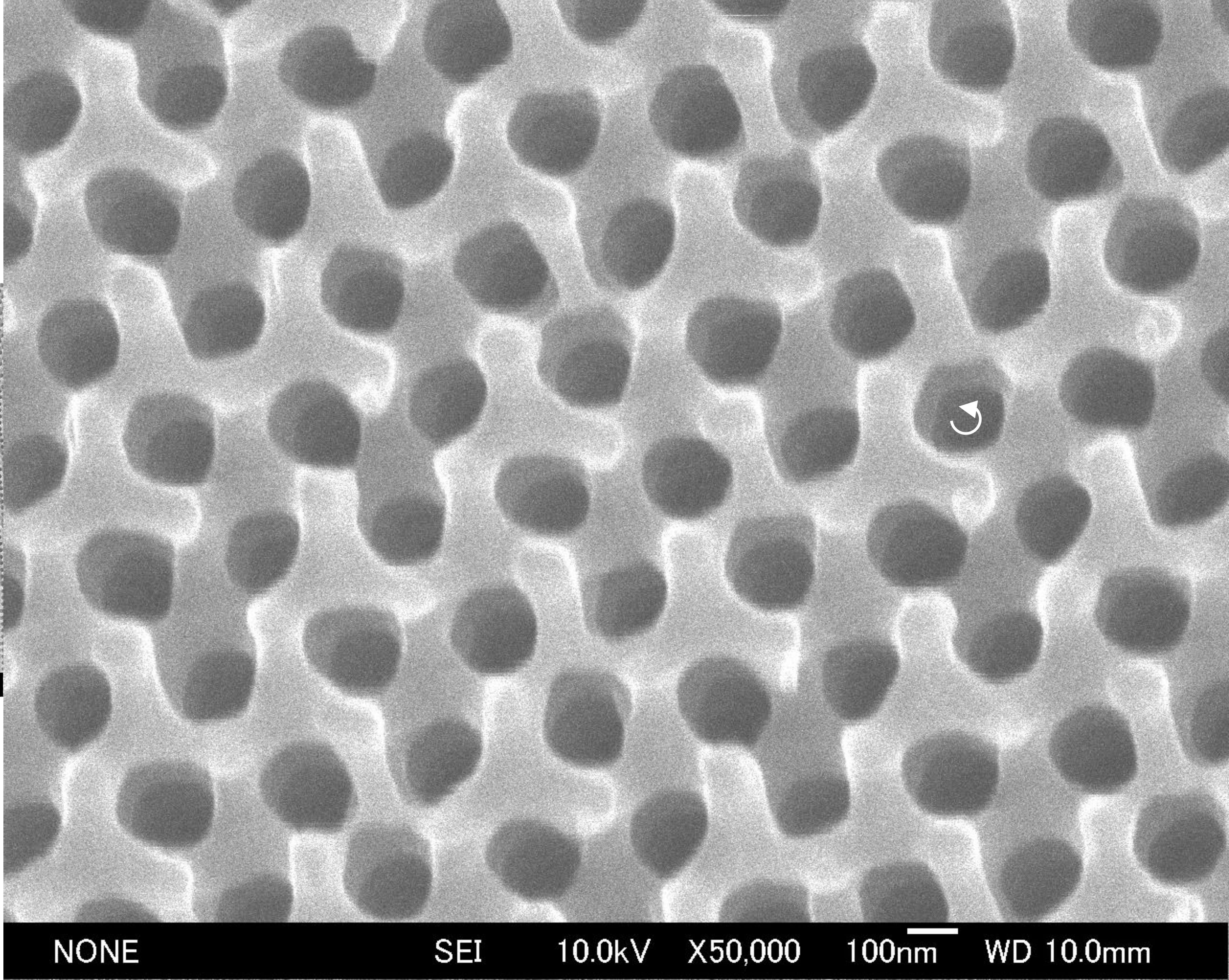

specimen No. 1
scale No. 2
domain No. 29
LH

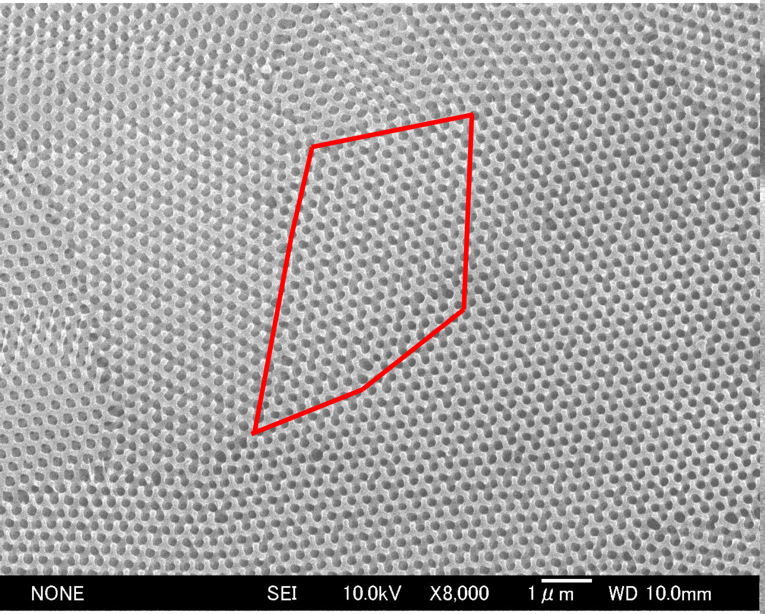

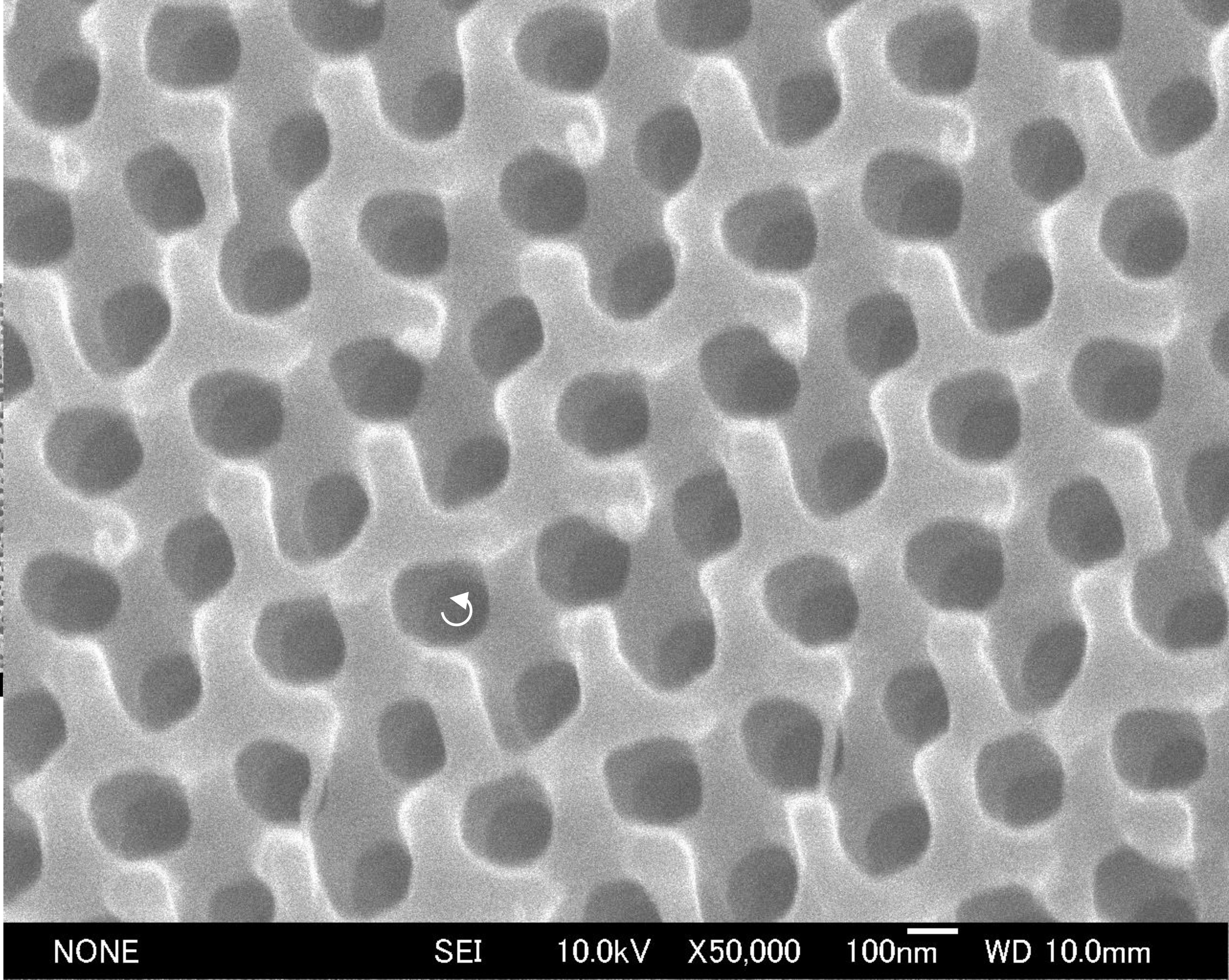

specimen No. 1
scale No. 3

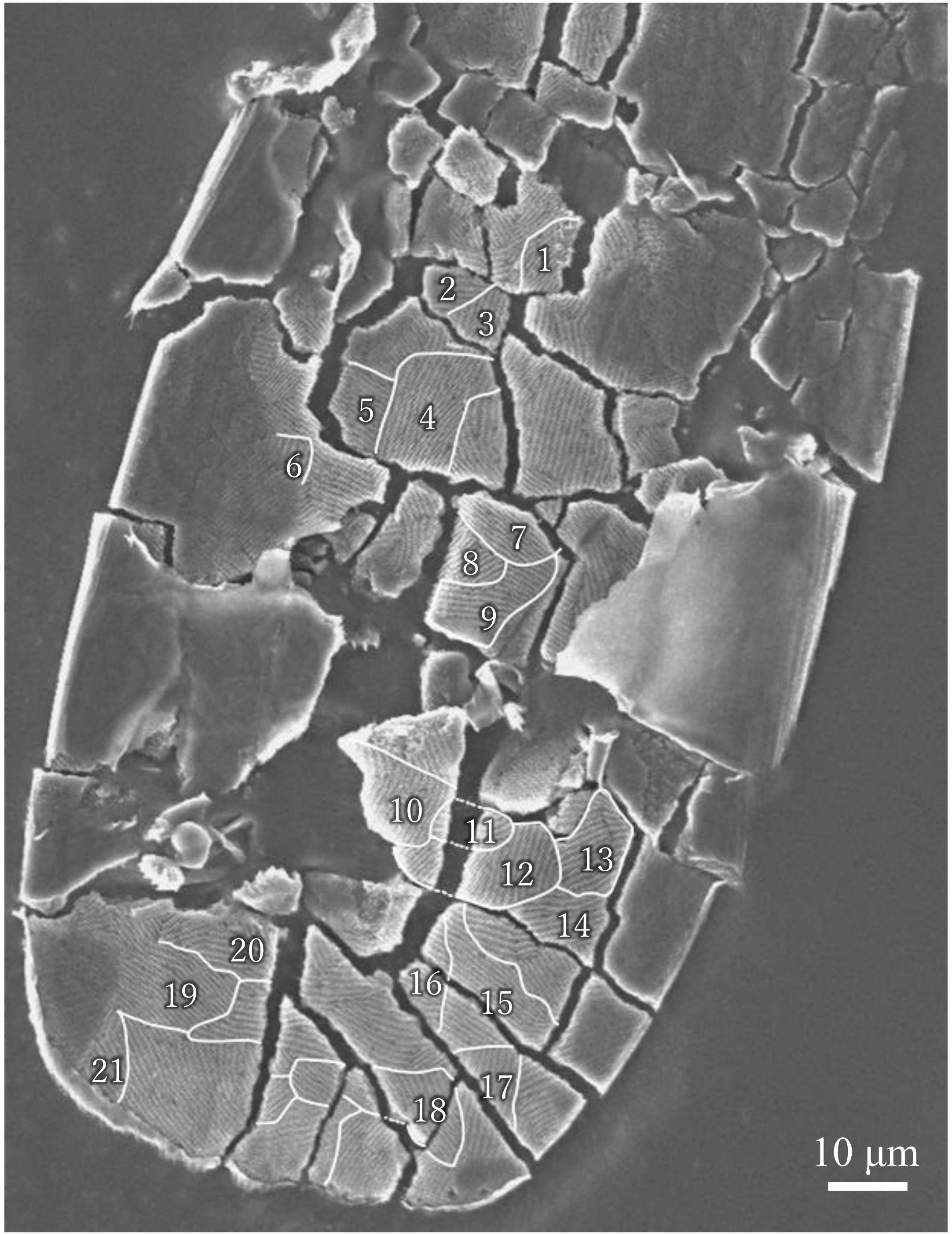

specimen No. 1
scale No. 3
domain No. 1
LH

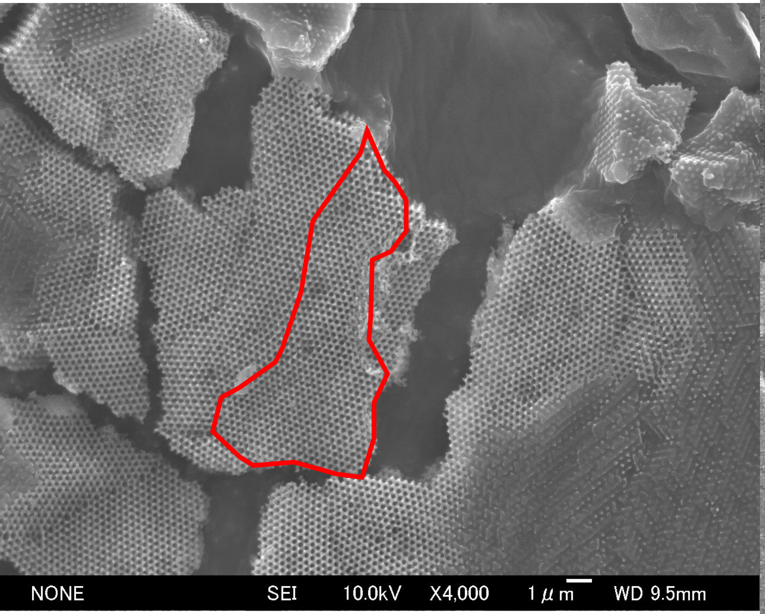

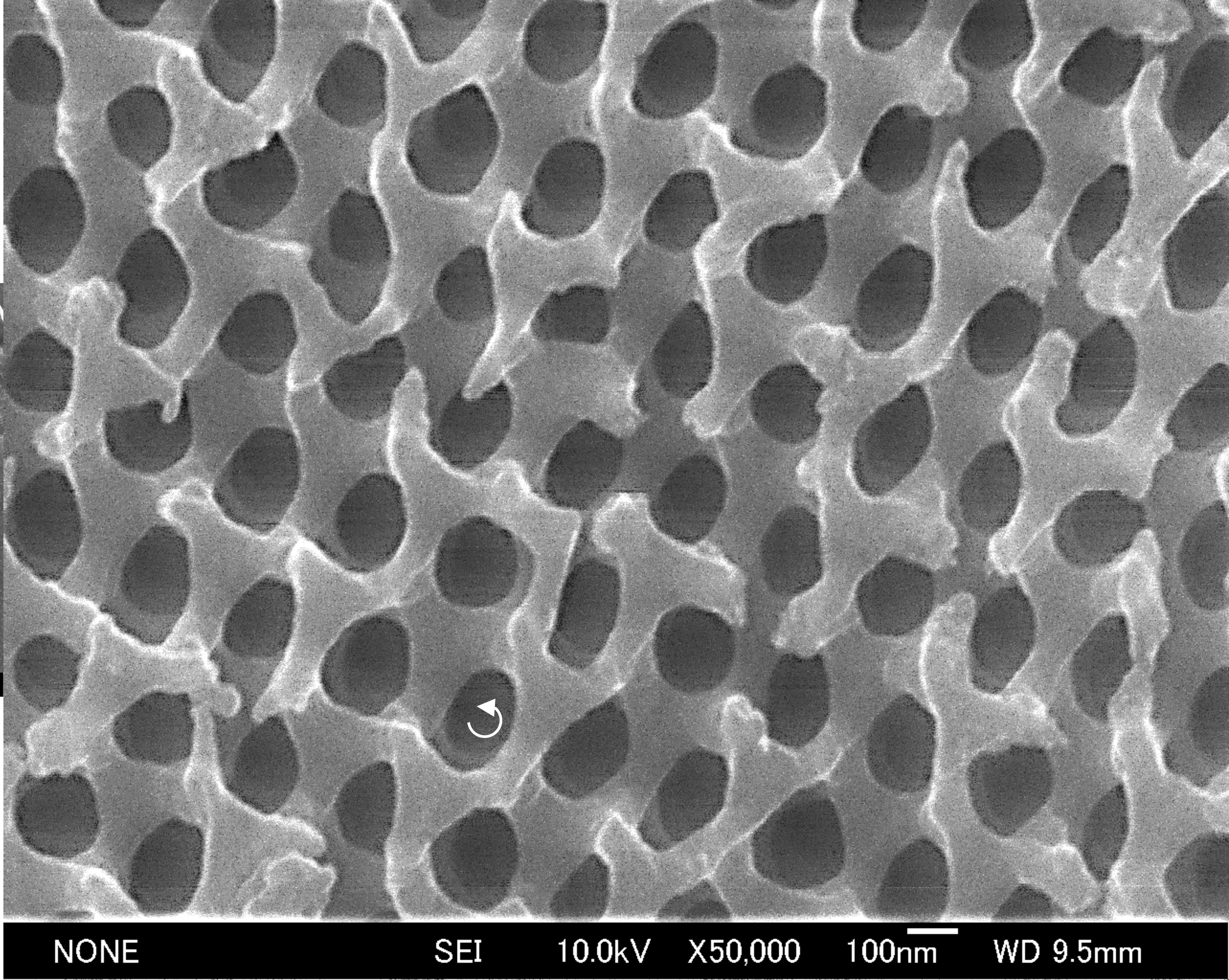

specimen No. 1
scale No. 3
domain No. 2
LH

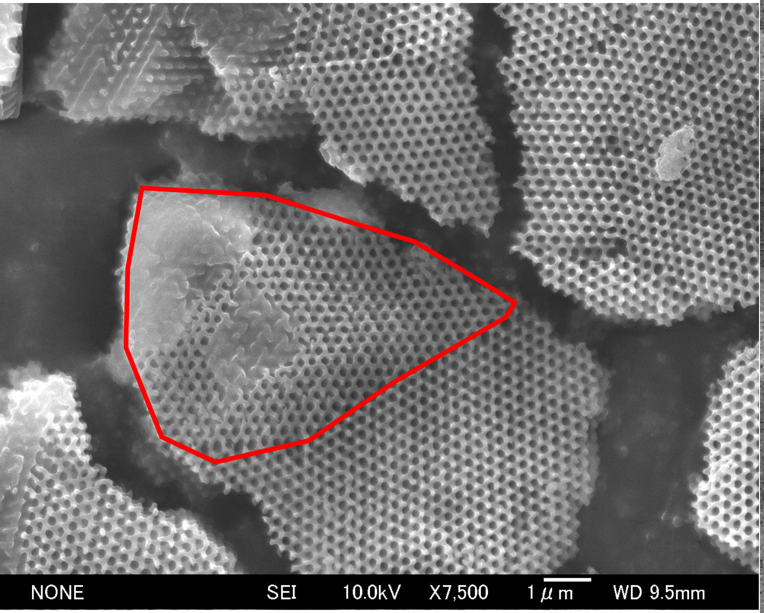

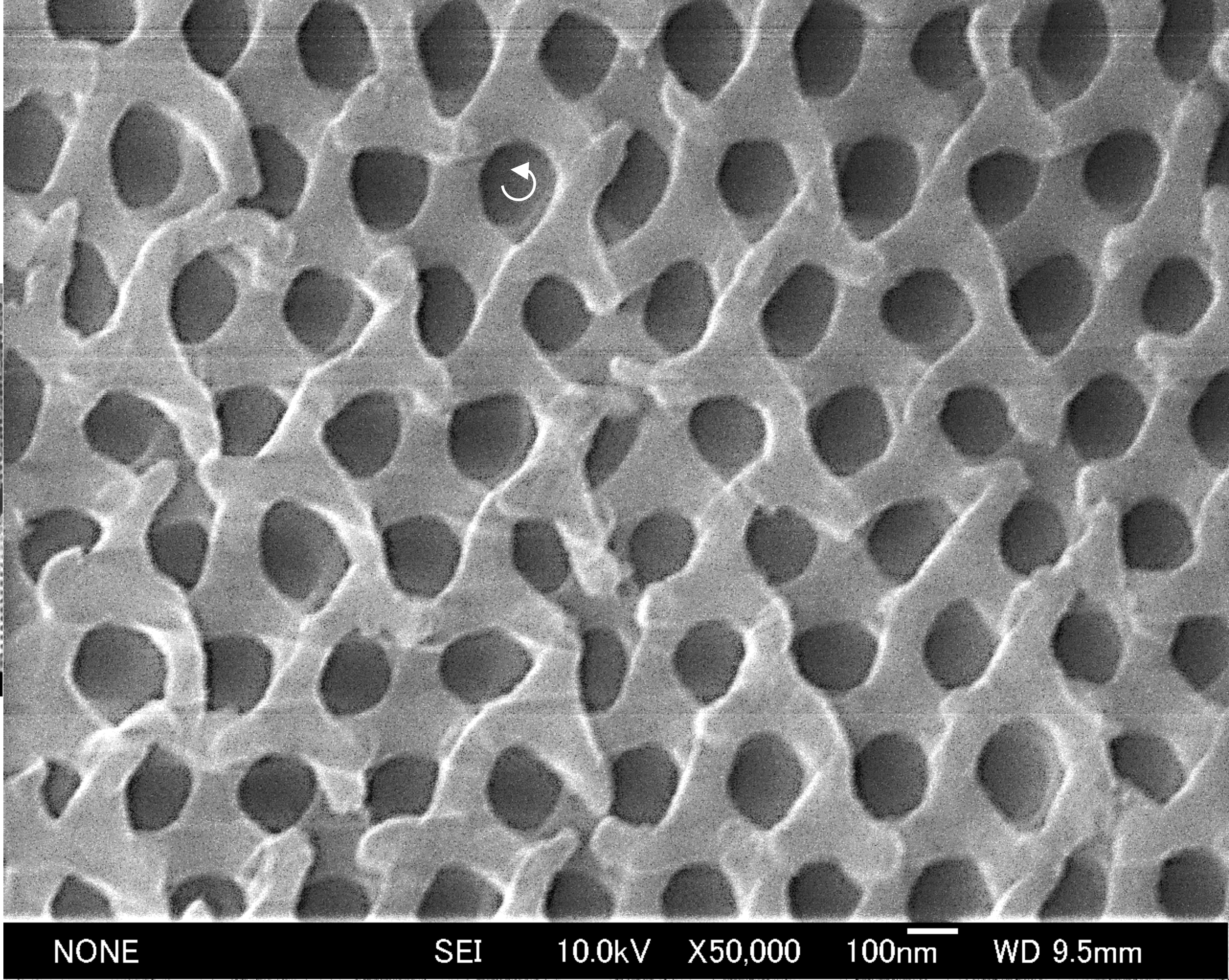

specimen No. 1
scale No. 3
domain No. 3
LH

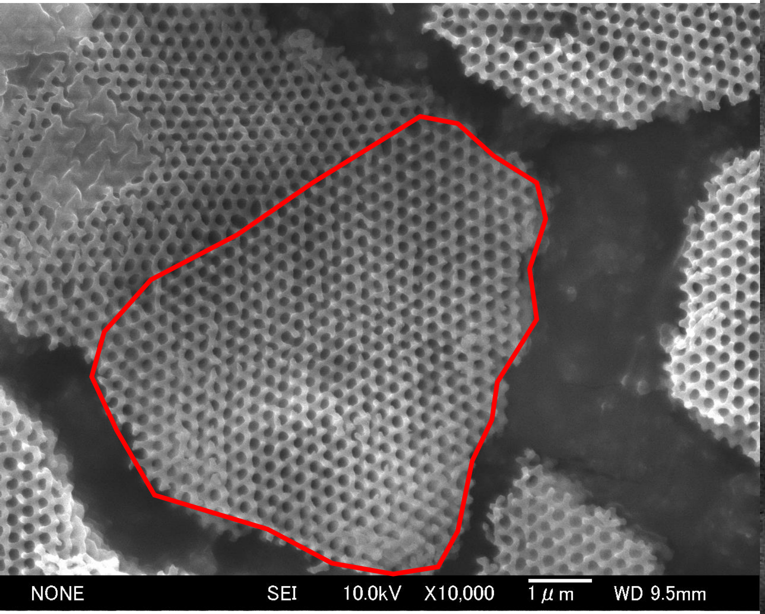

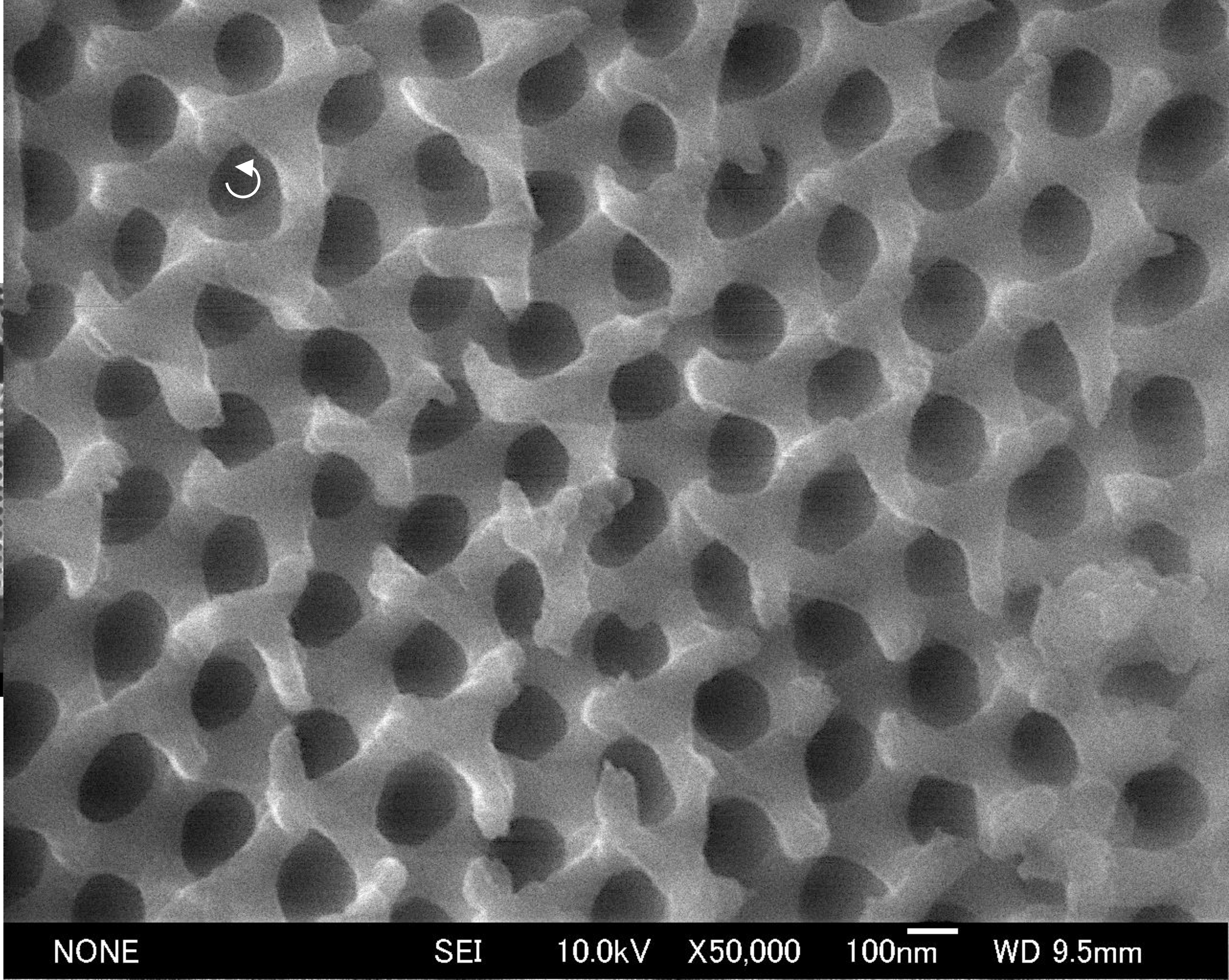

specimen No. 1
scale No. 3
domain No. 4
LH

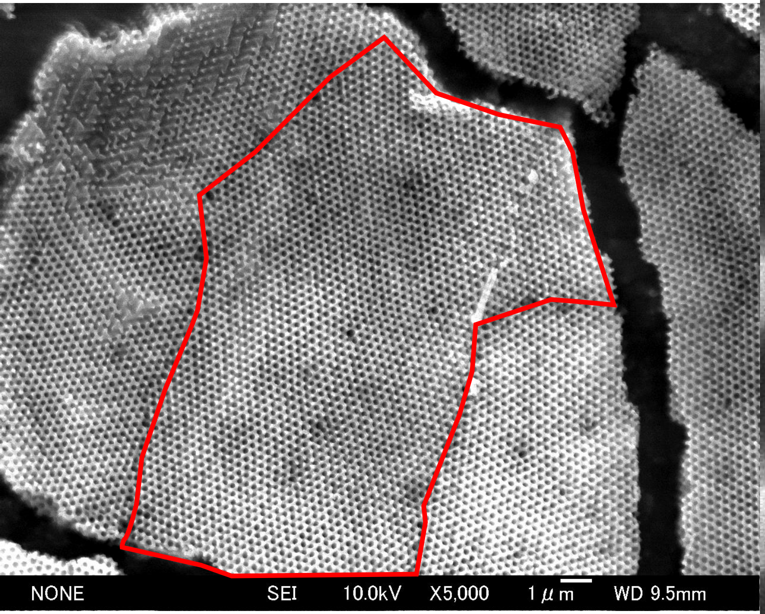

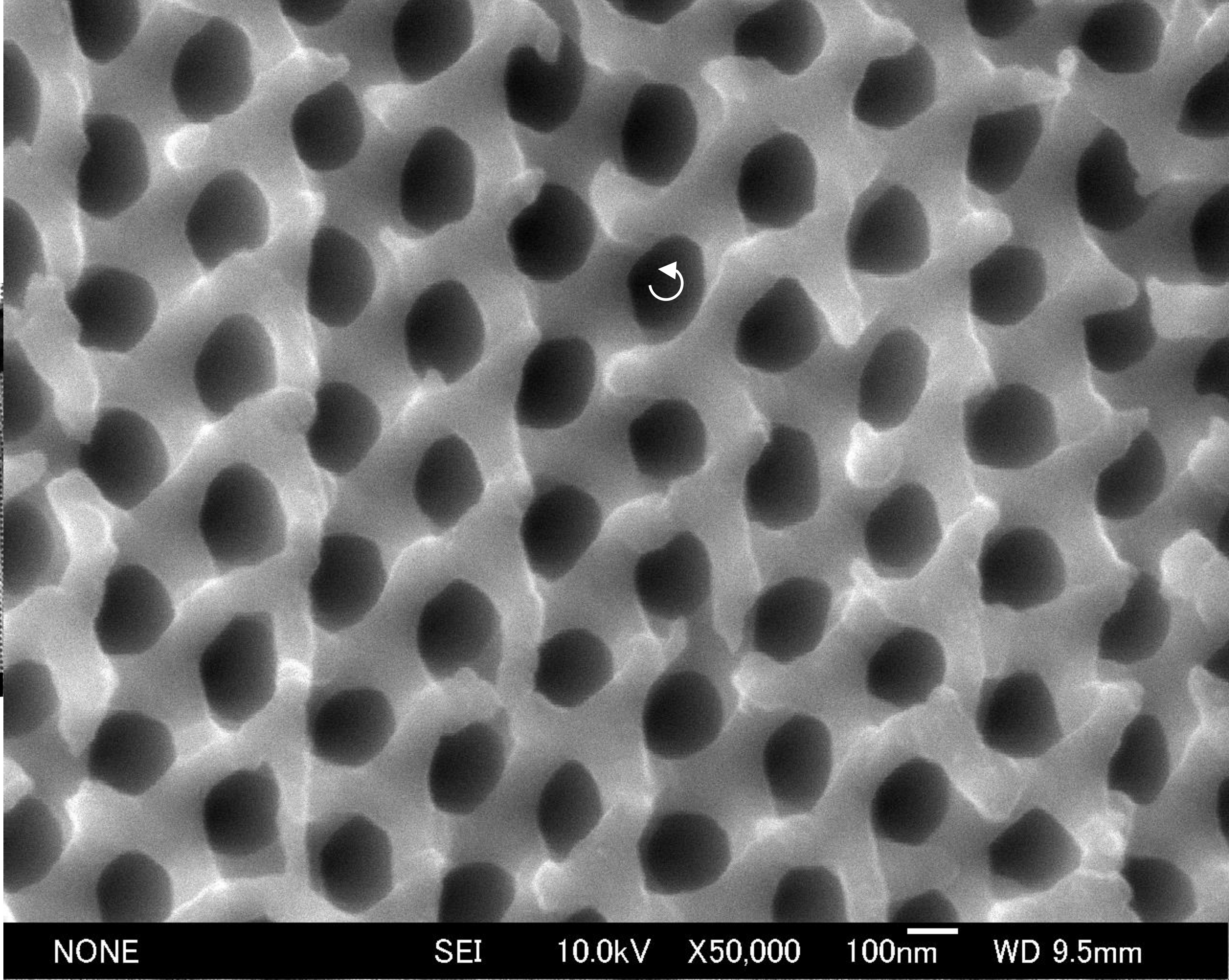

specimen No. 1
scale No. 3
domain No. 5
LH

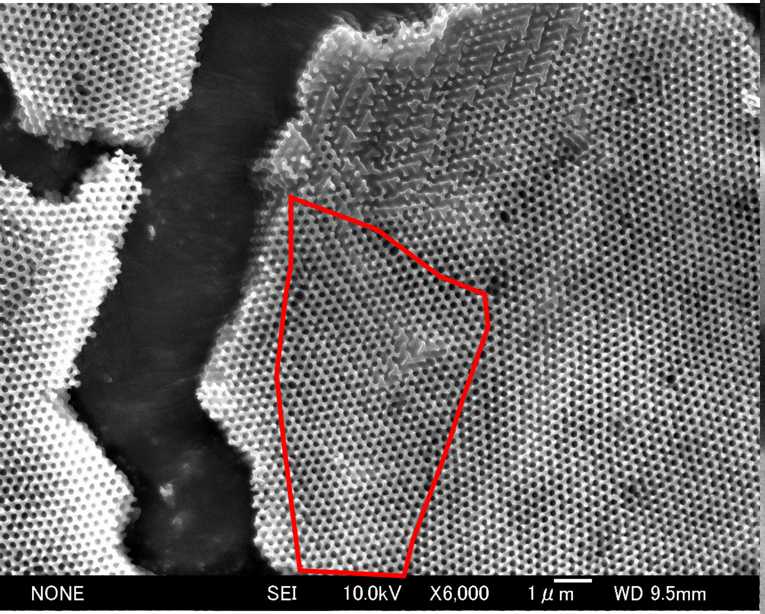

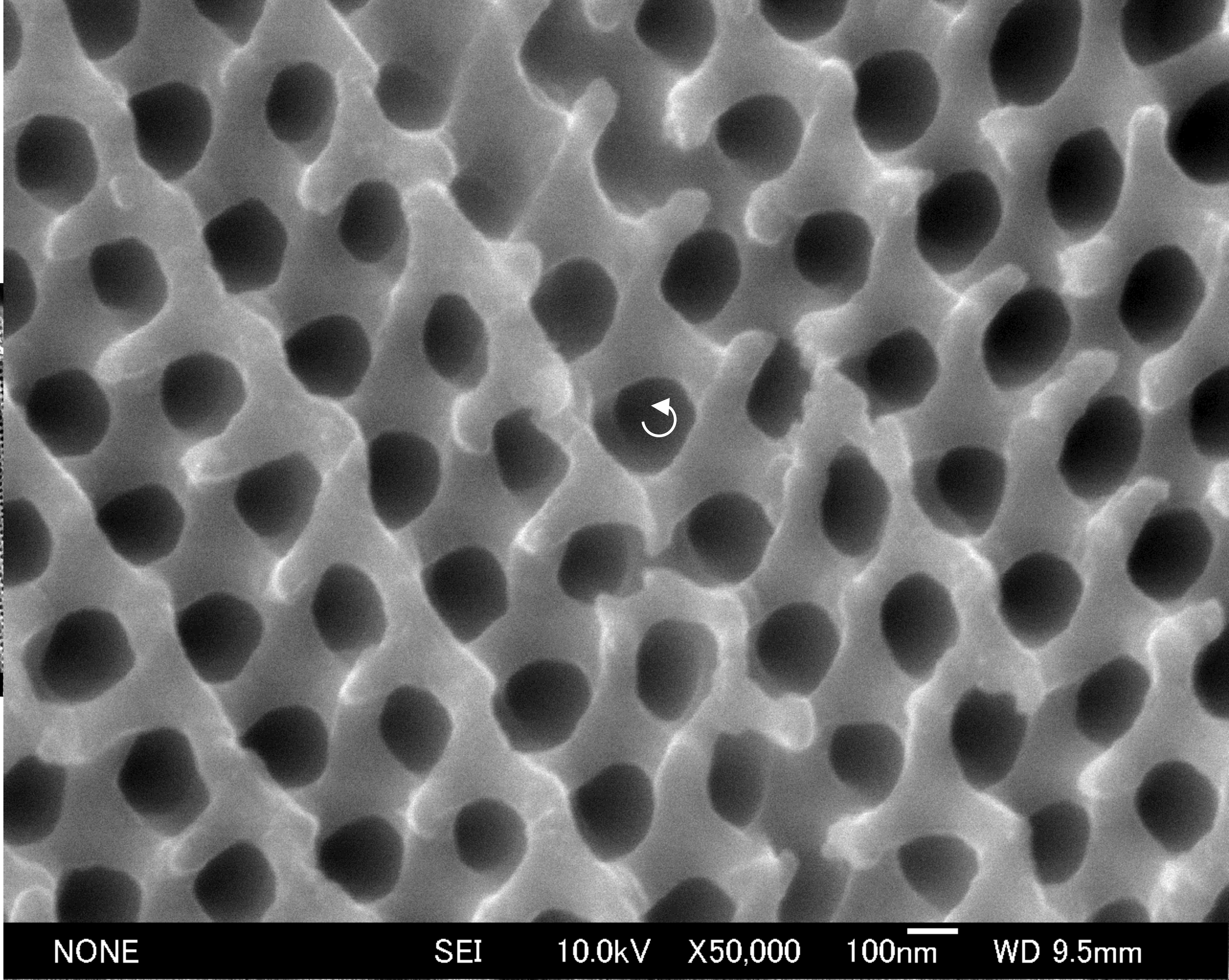

specimen No. 1
scale No. 3
domain No. 6
LH

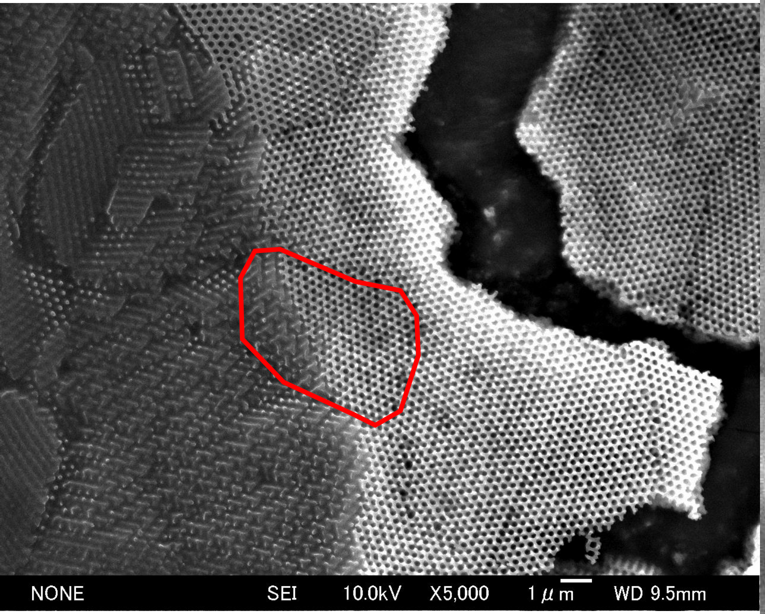

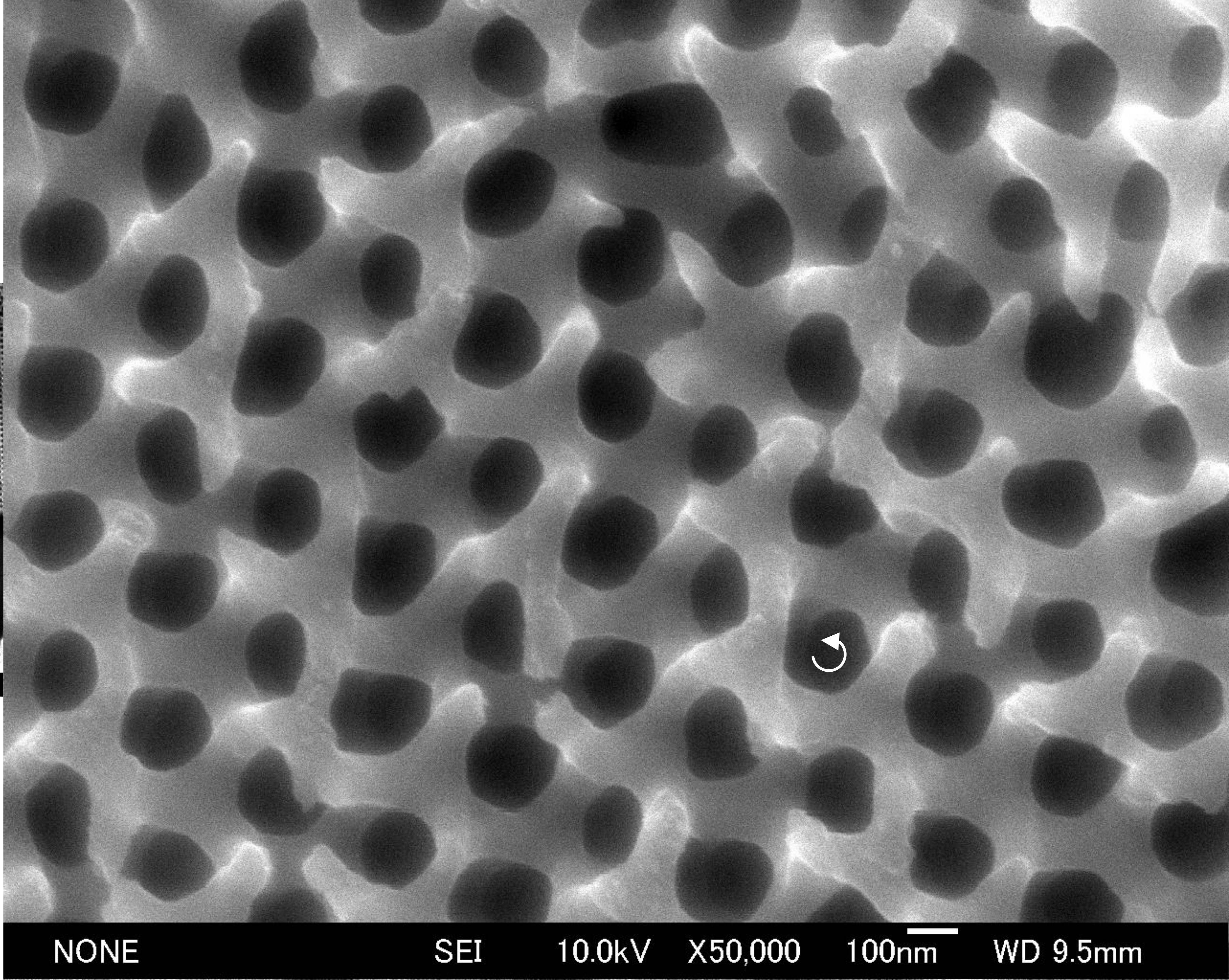

specimen No. 1
scale No. 3
domain No. 7
LH

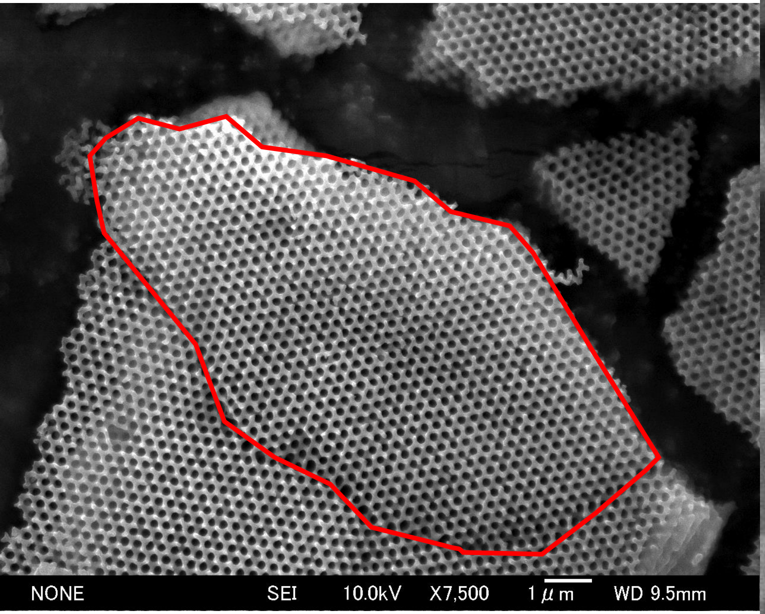

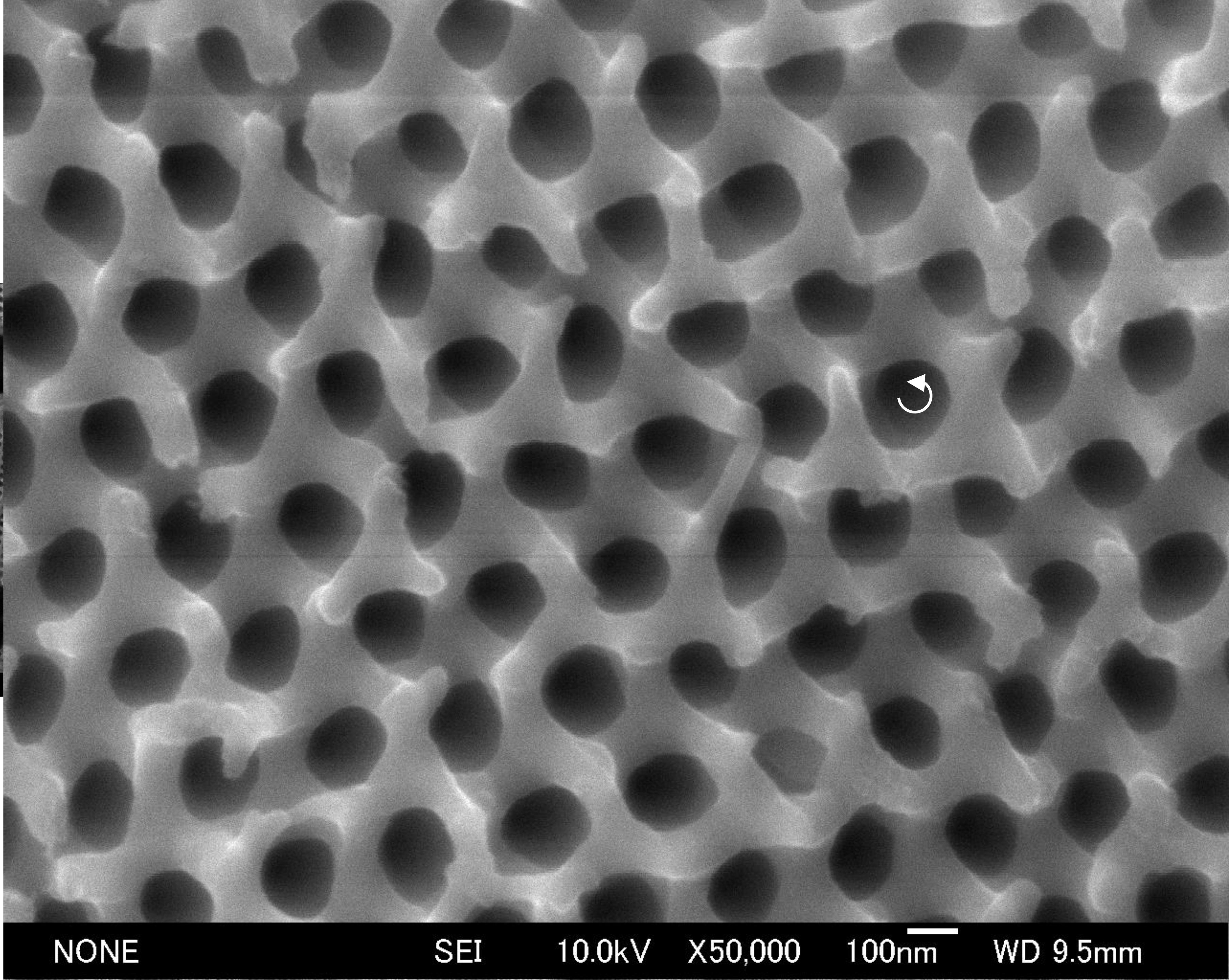

specimen No. 1
scale No. 3
domain No. 8
LH

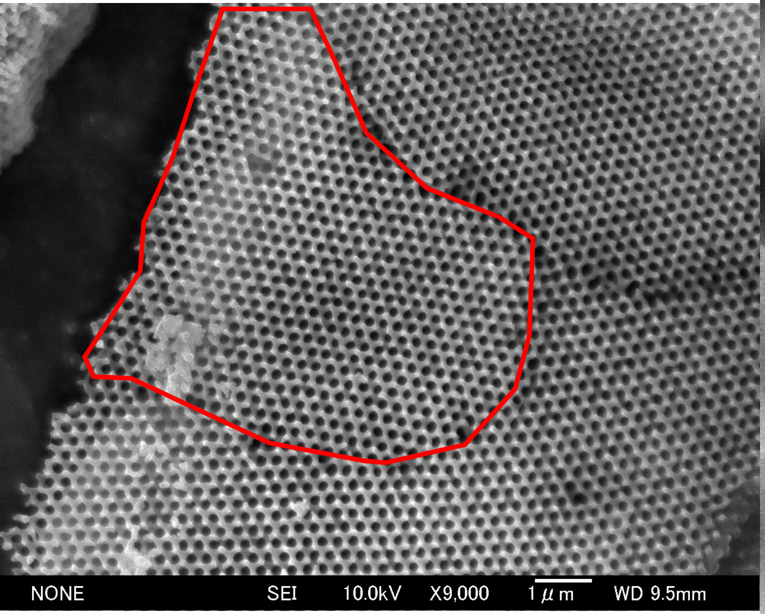

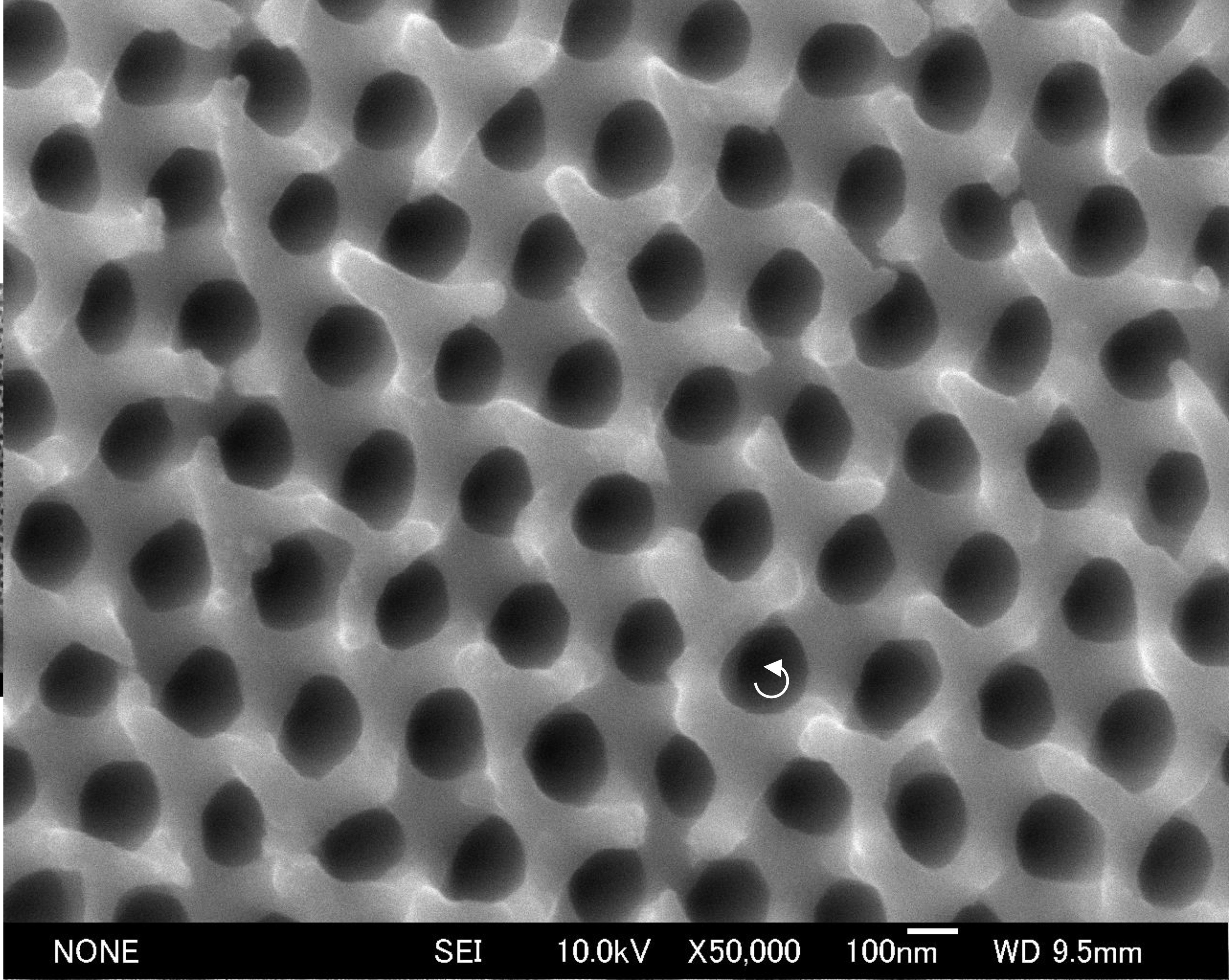

specimen No. 1
scale No. 3
domain No. 9
LH

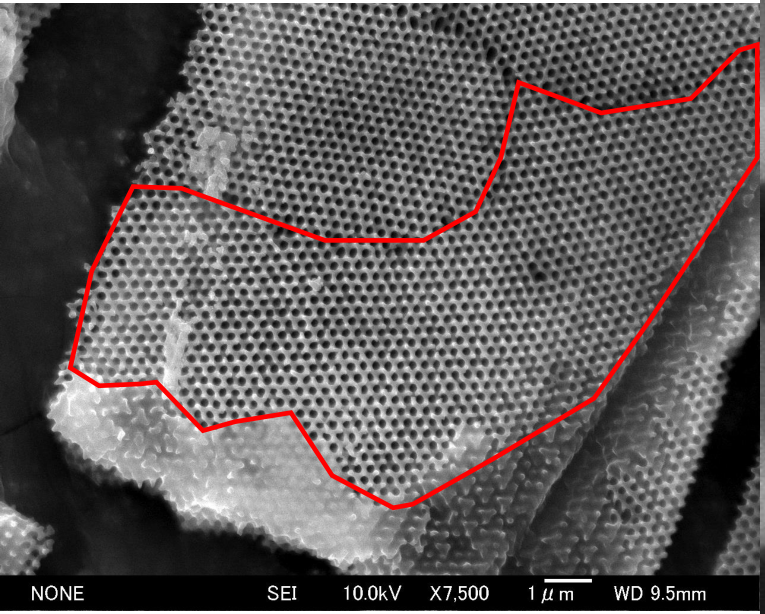

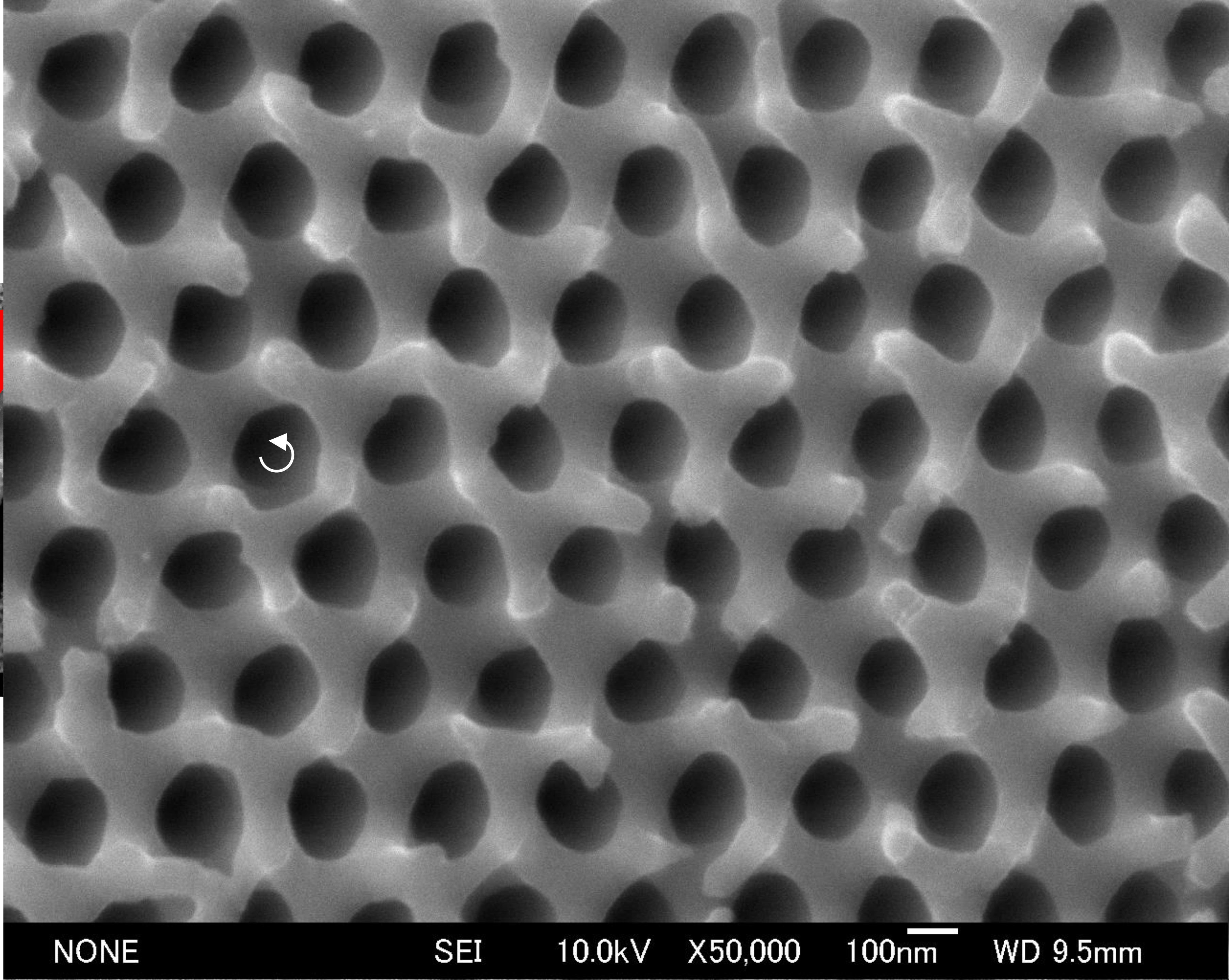

specimen No. 1
scale No. 3
domain No. 10
LH

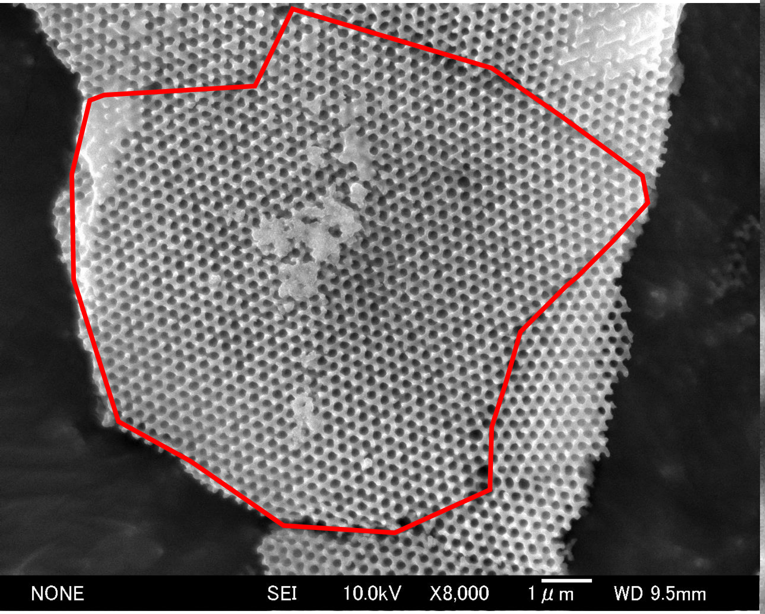

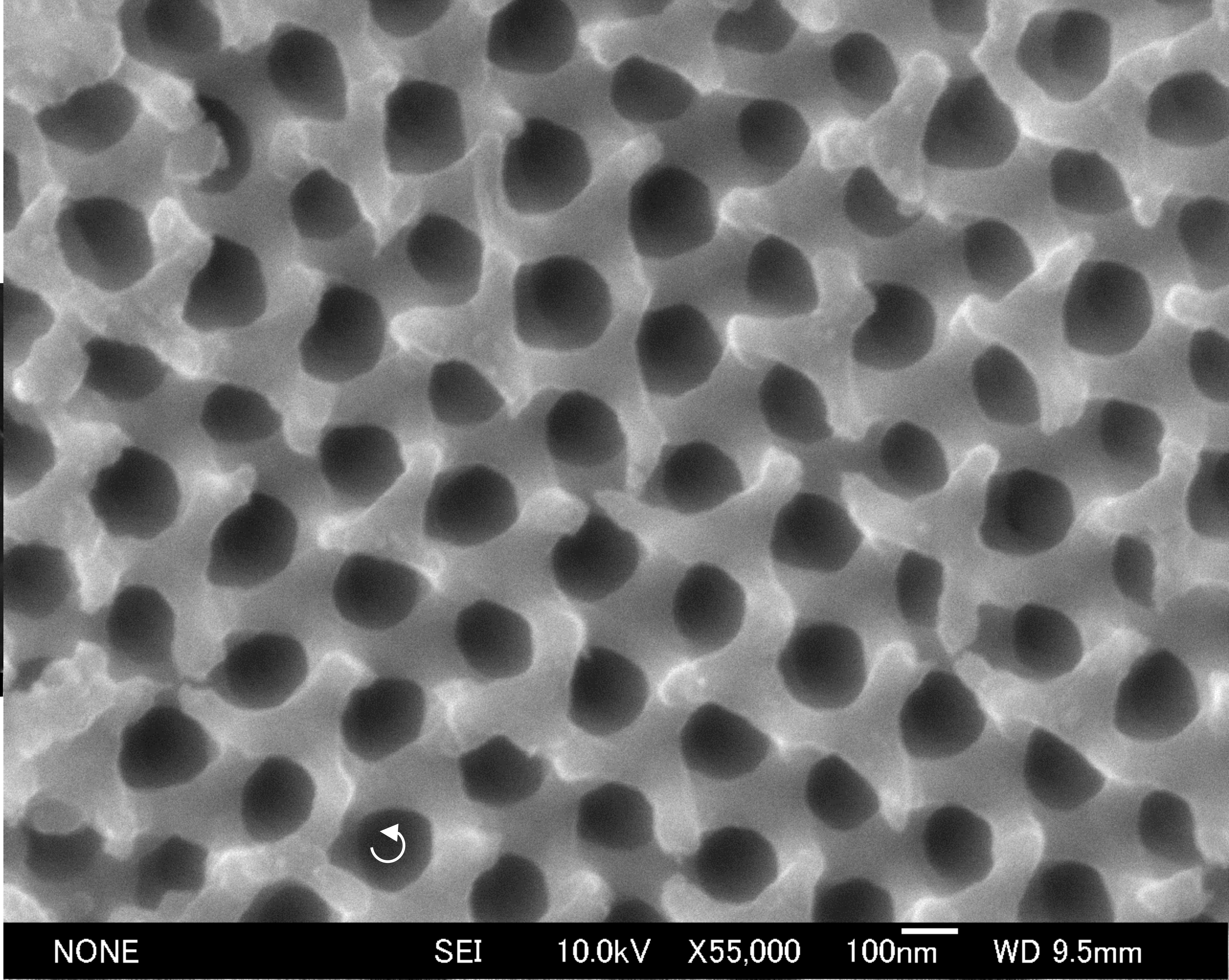

specimen No. 1
scale No. 3
domain No. 11
LH

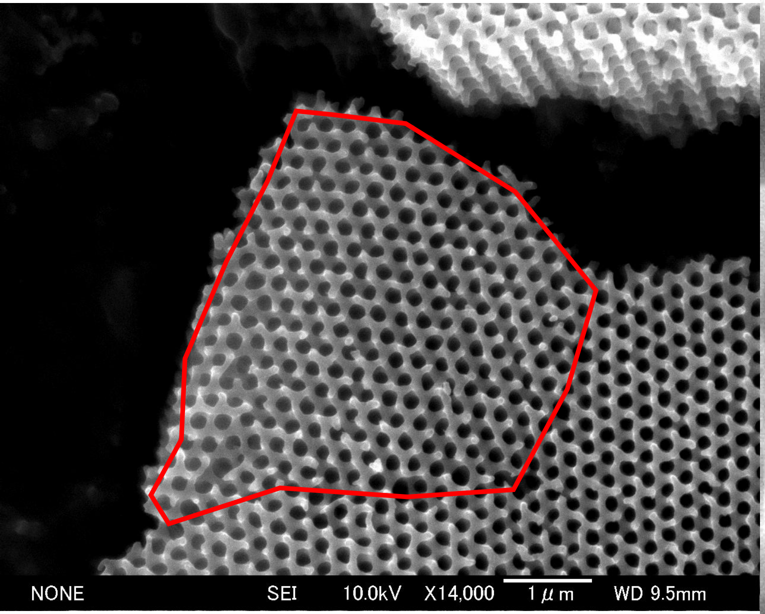

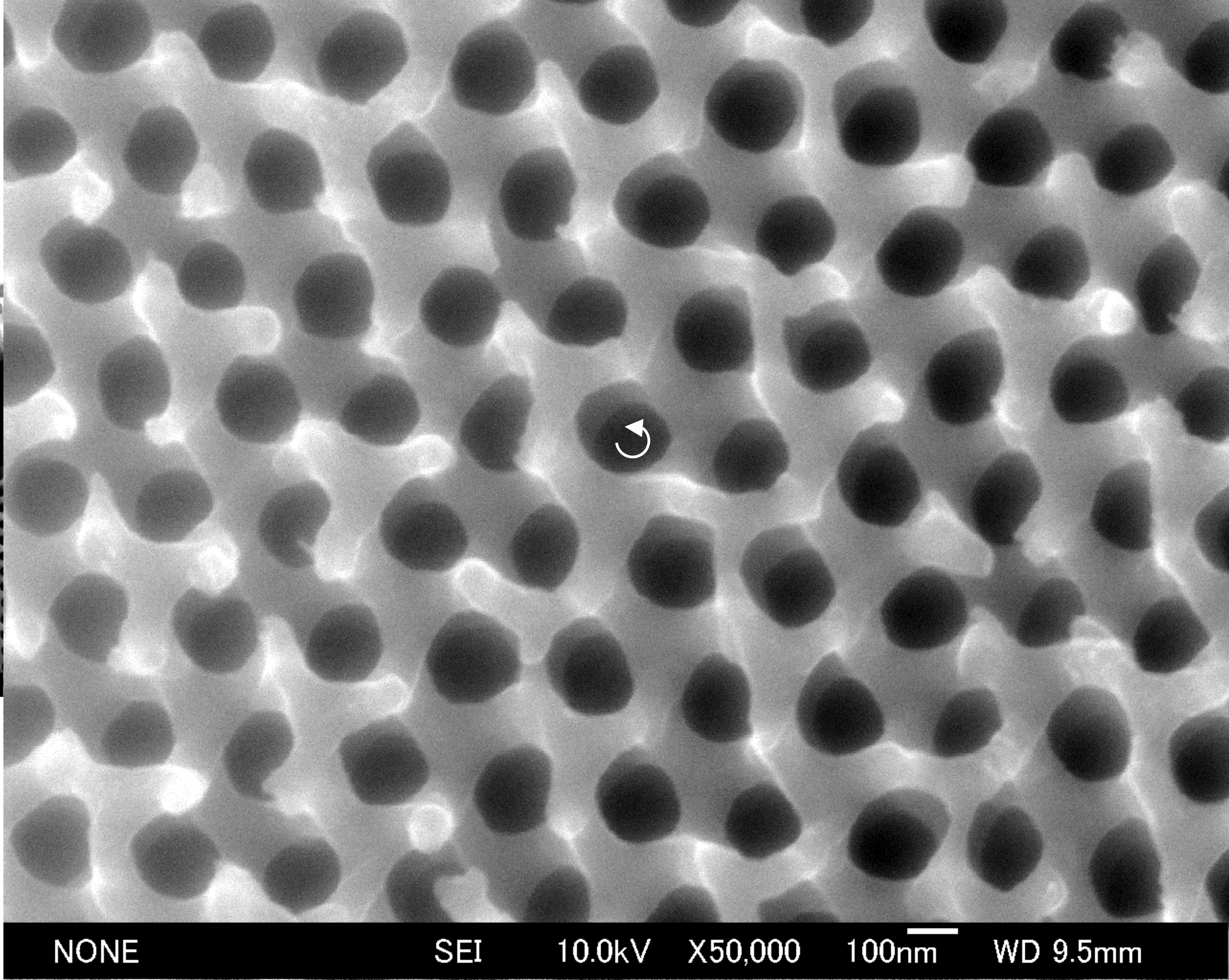

specimen No. 1
scale No. 3
domain No. 12
LH

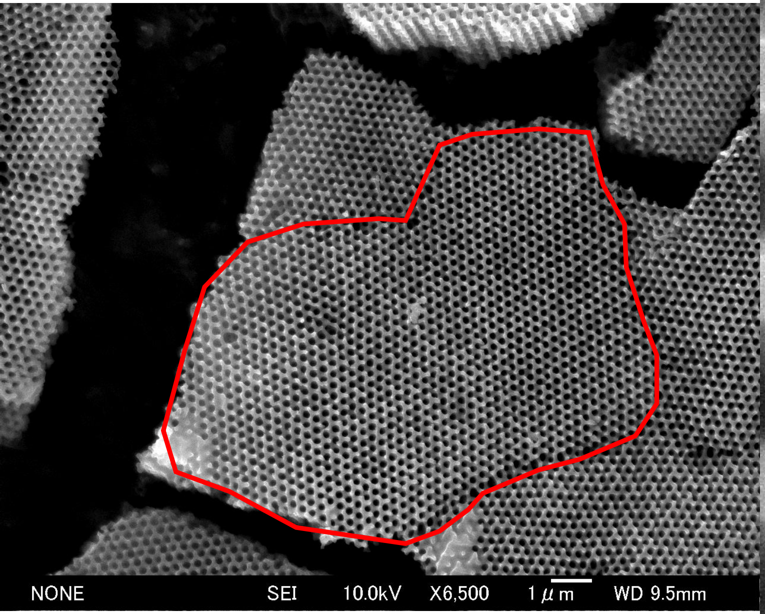

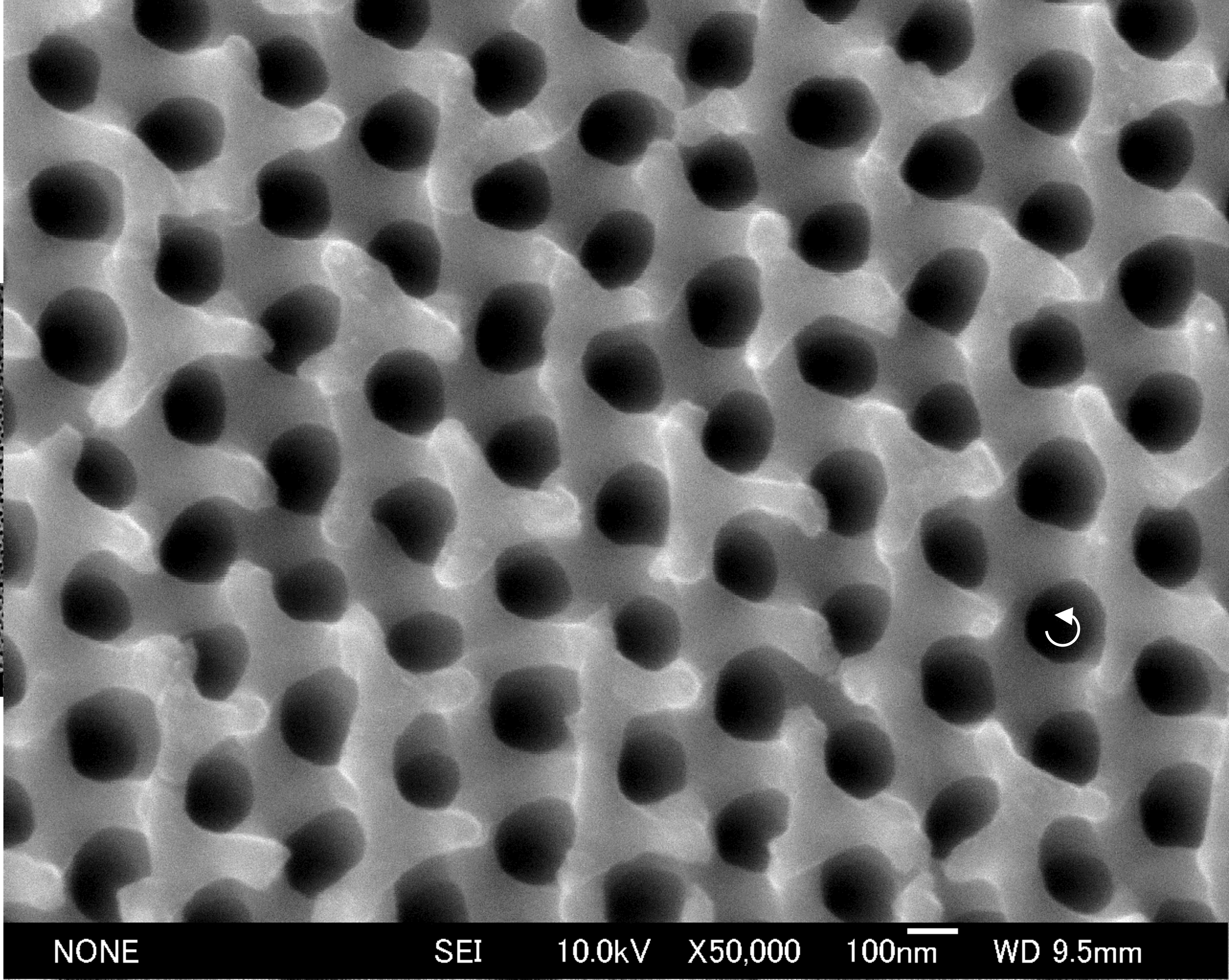

specimen No. 1
scale No. 3
domain No. 13
LH

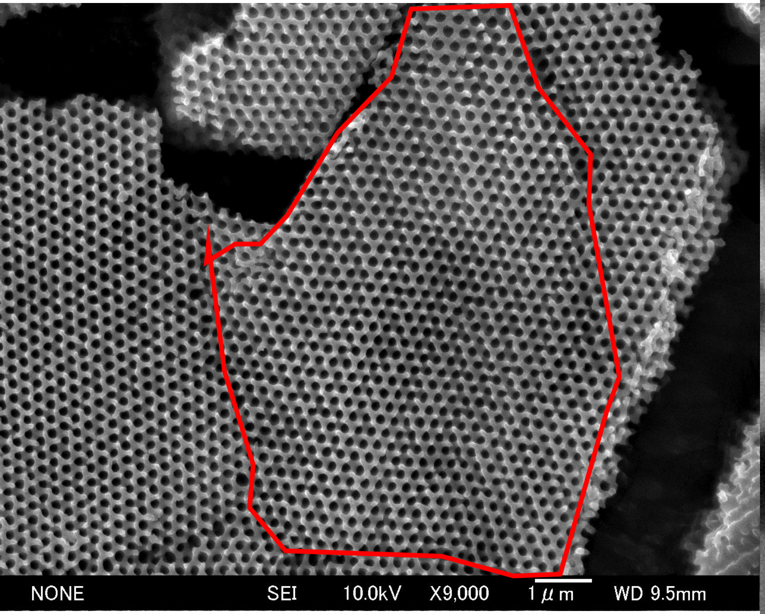

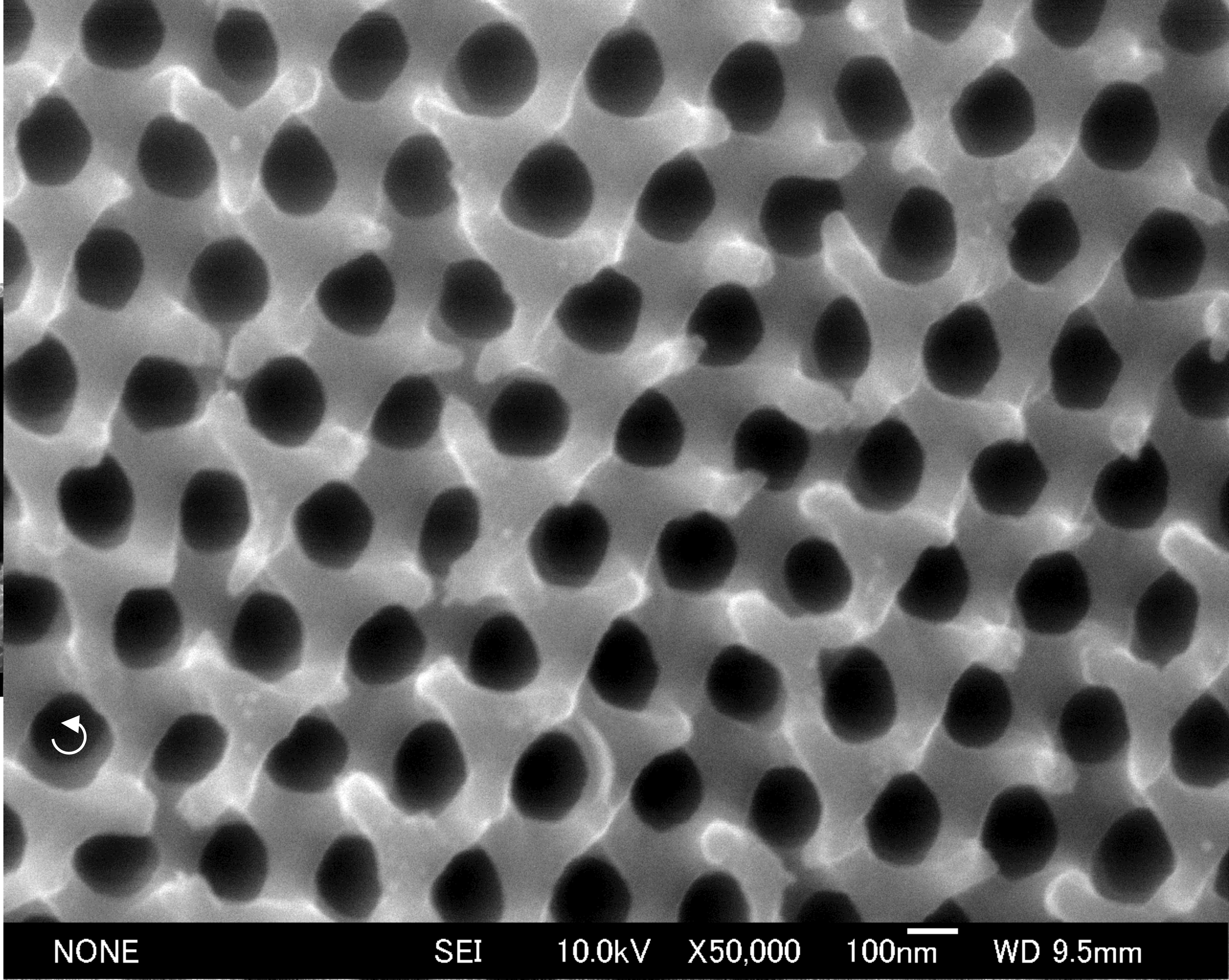

specimen No. 1
scale No. 3
domain No. 14
LH

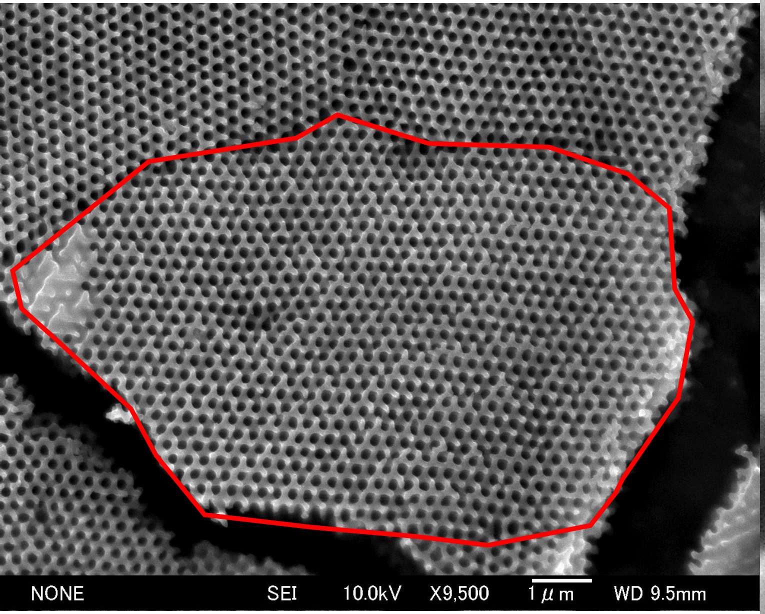

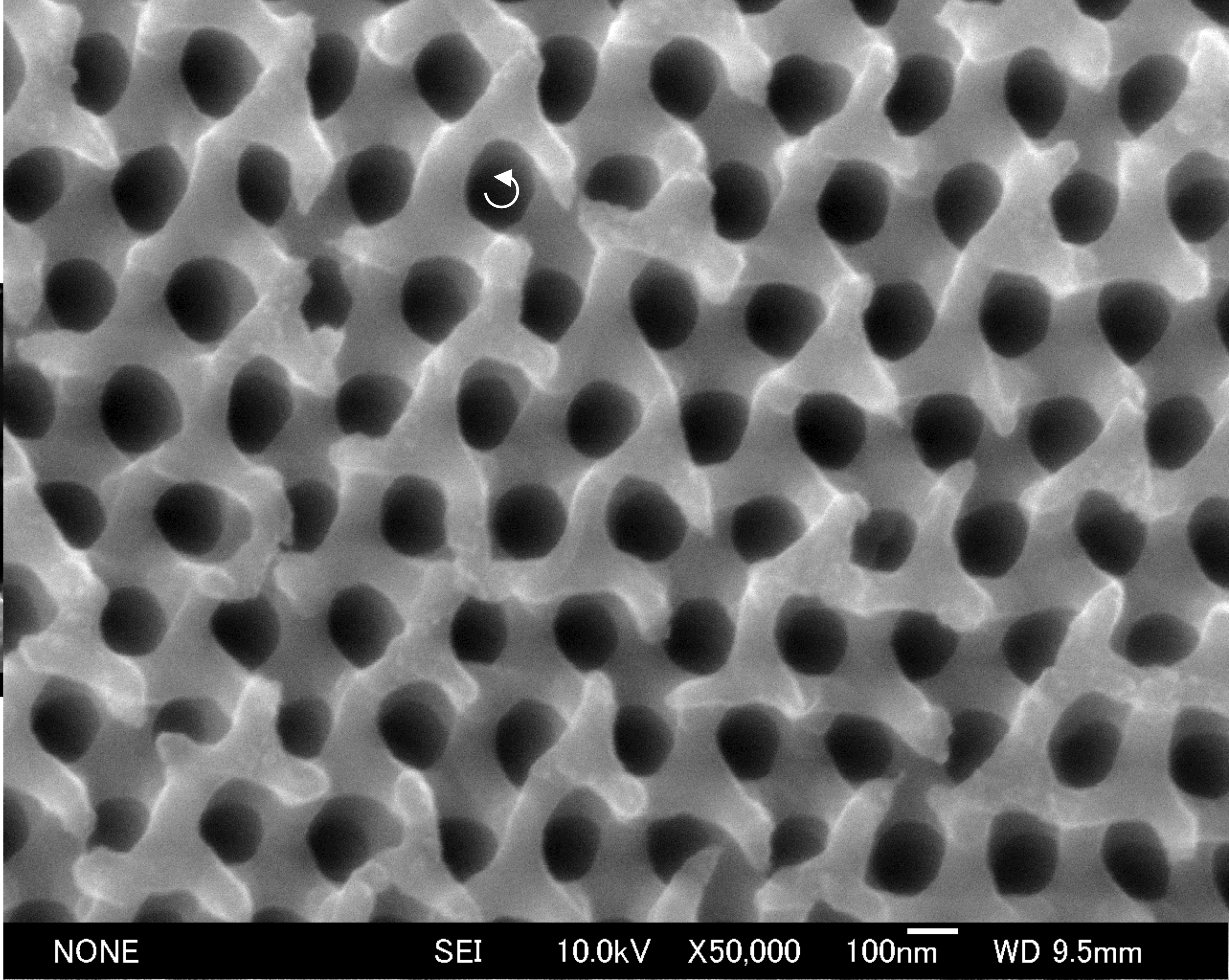

specimen No. 1
scale No. 3
domain No. 15
LH

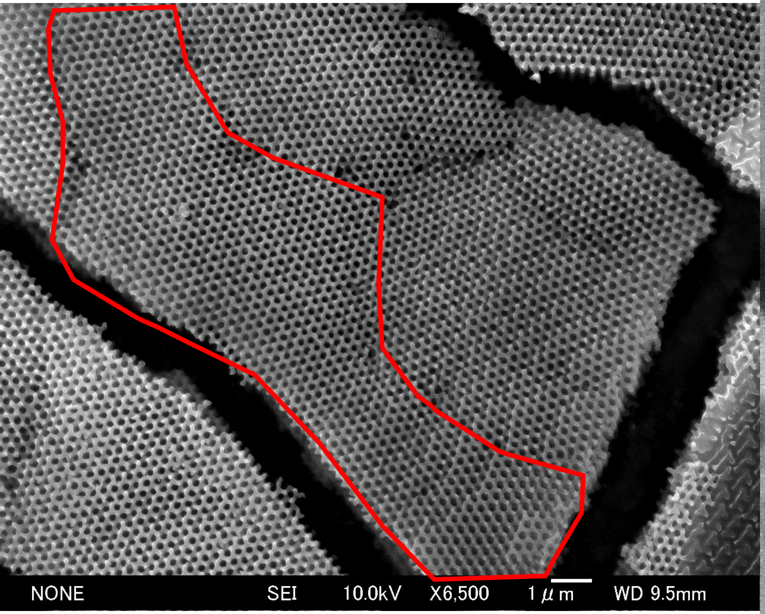

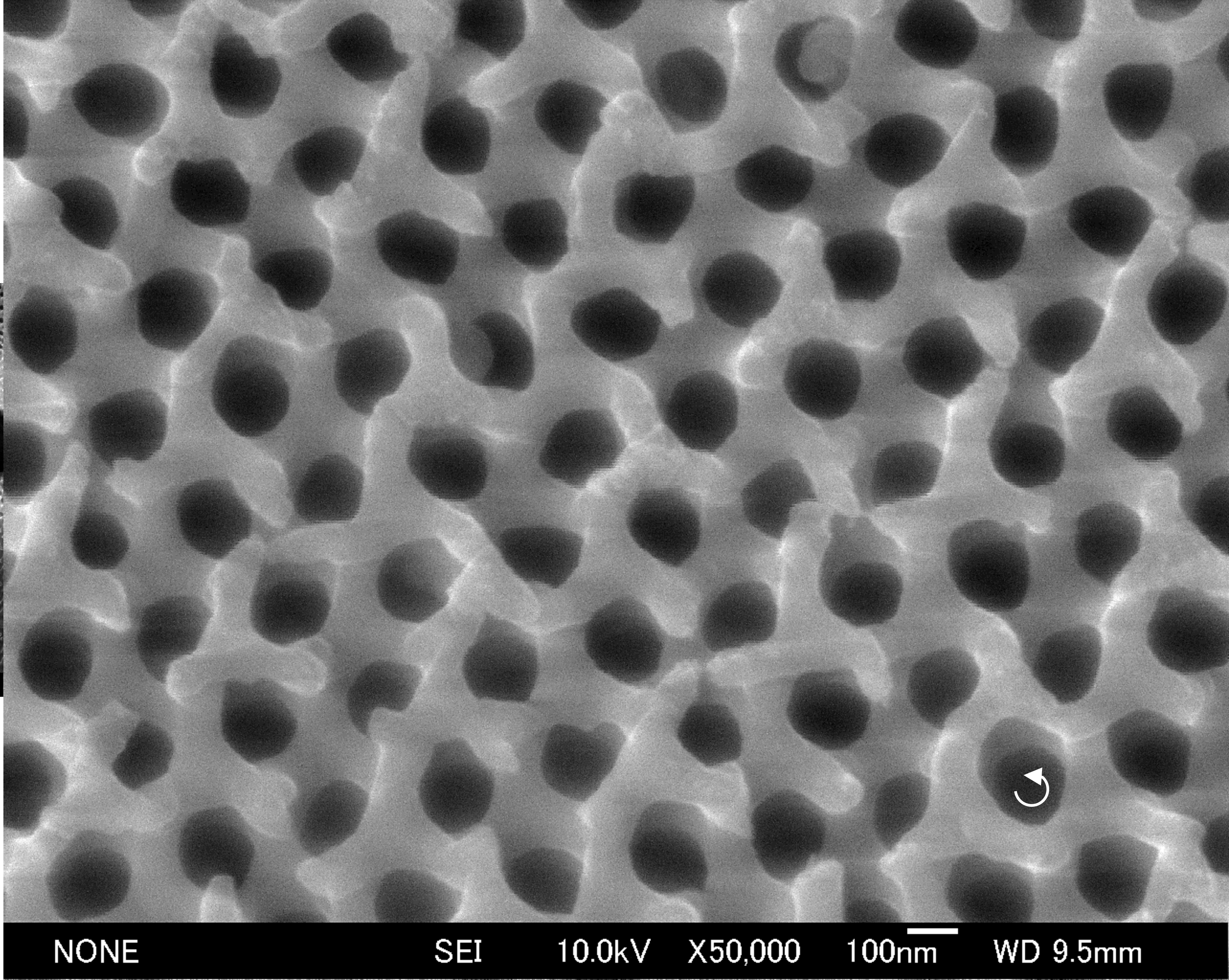

specimen No. 1
scale No. 3
domain No. 16
LH

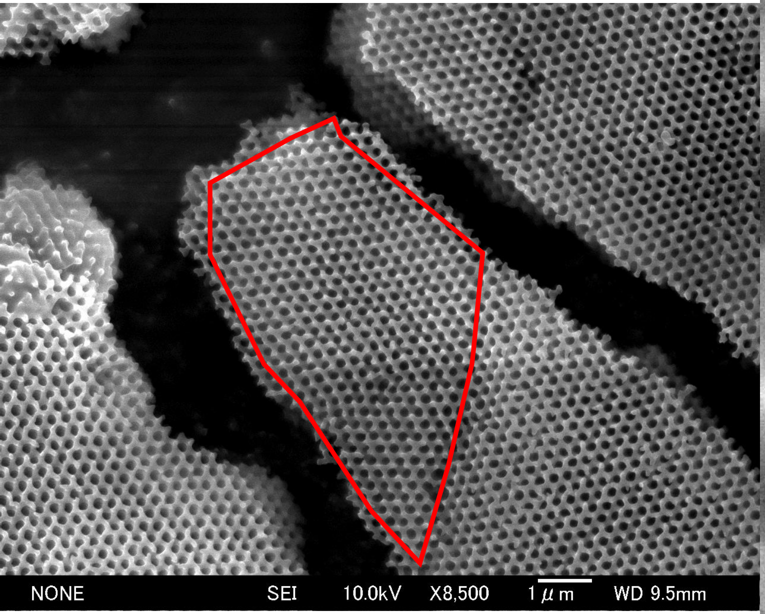

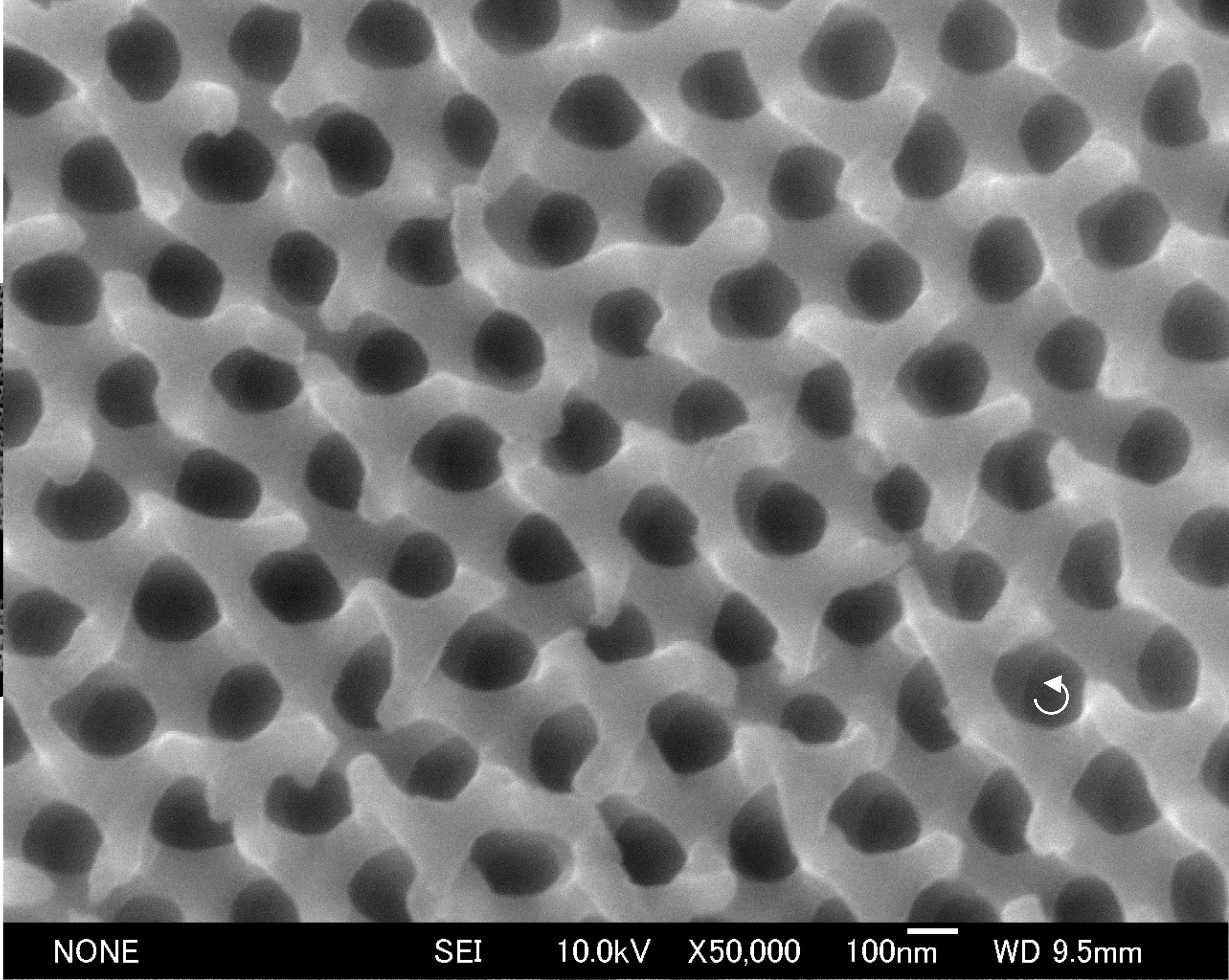

specimen No. 1
scale No. 3
domain No. 17
LH

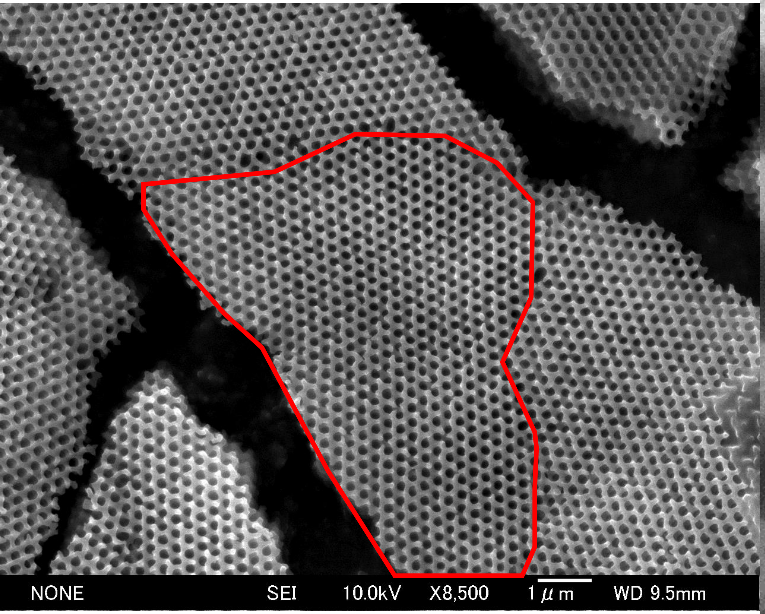

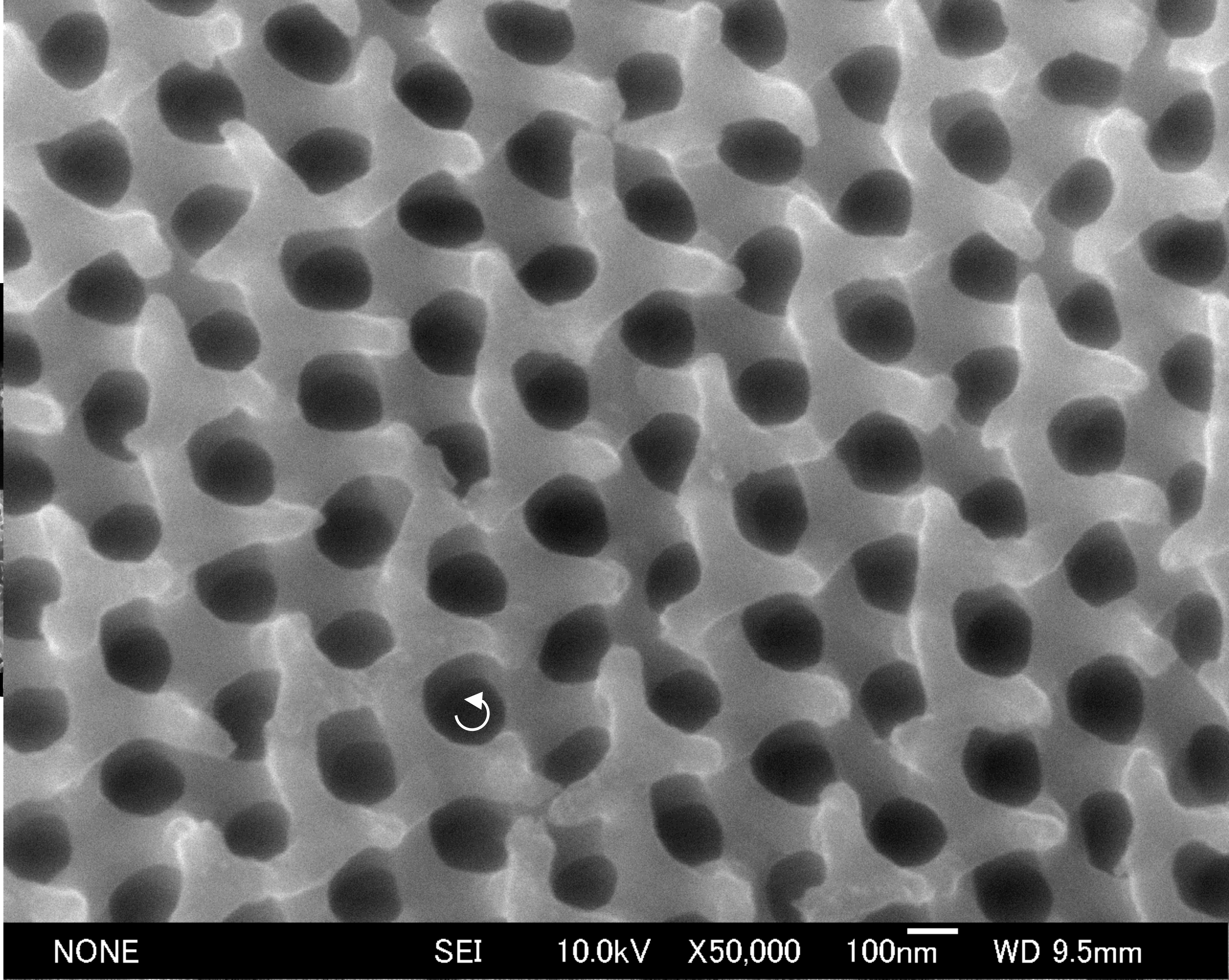

specimen No. 1
scale No. 3
domain No. 18
LH

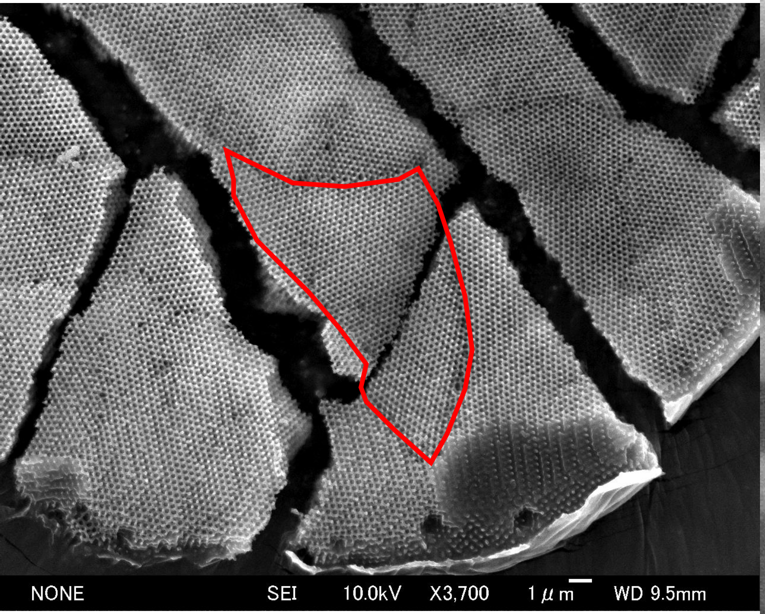

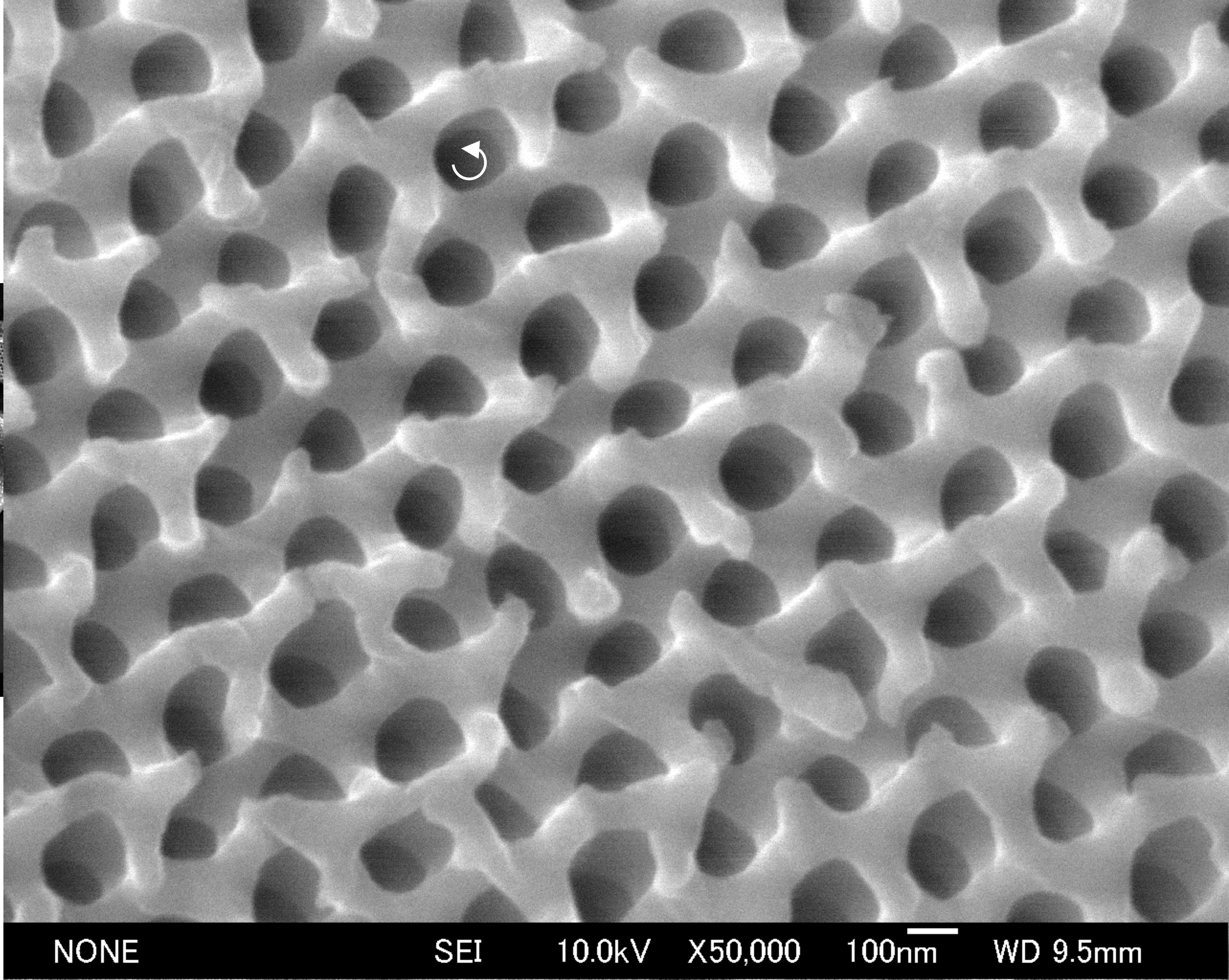

specimen No. 1
scale No. 3
domain No. 19
LH

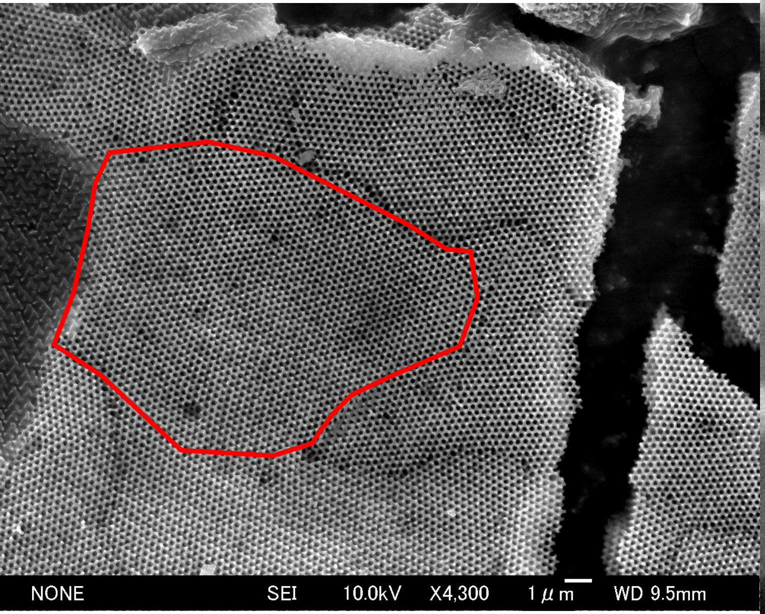

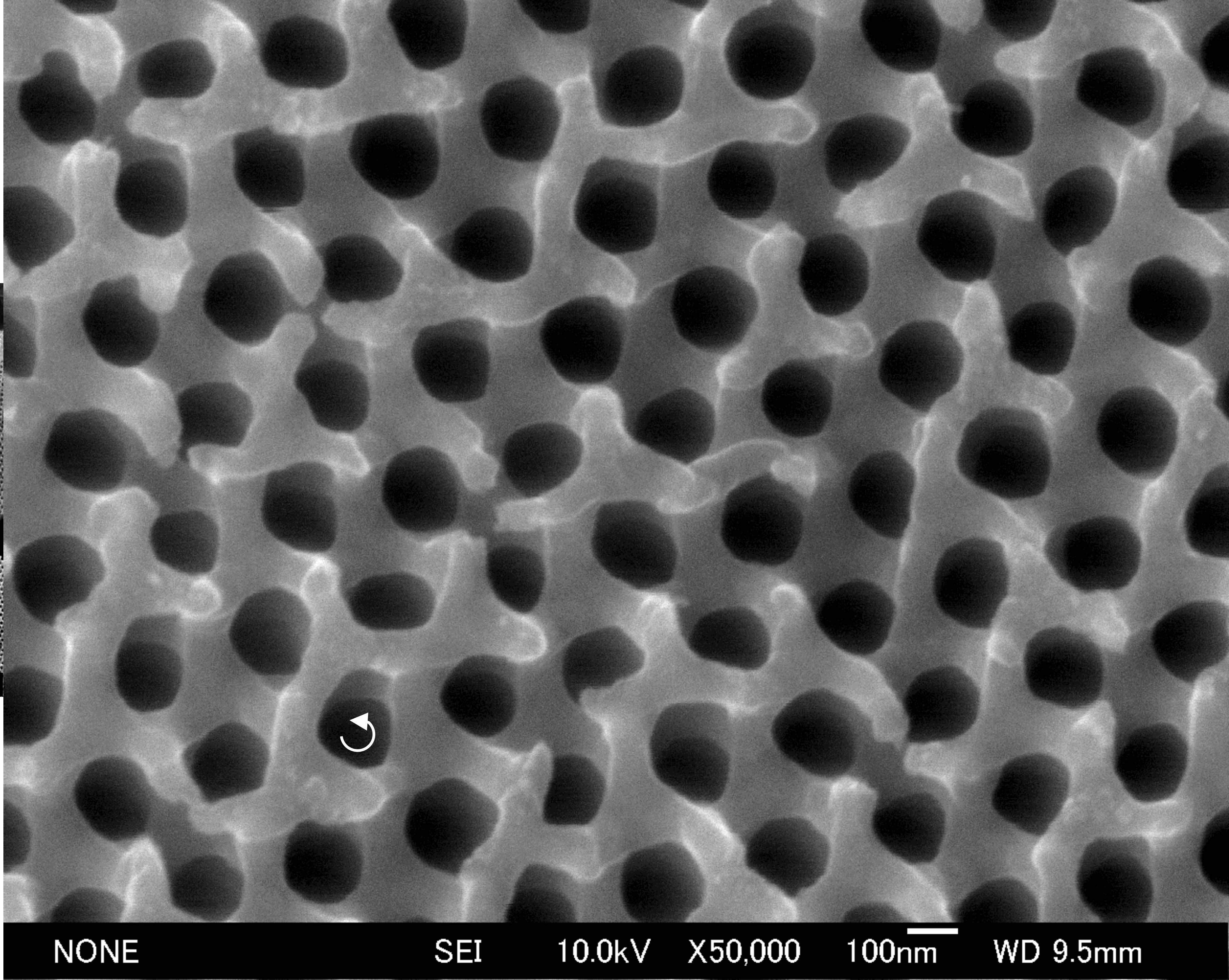

specimen No. 1
scale No. 3
domain No. 20
LH

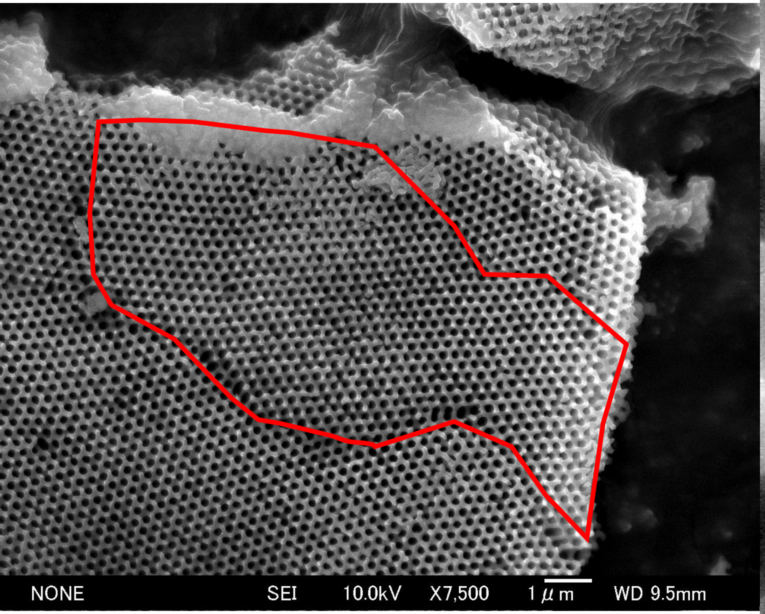

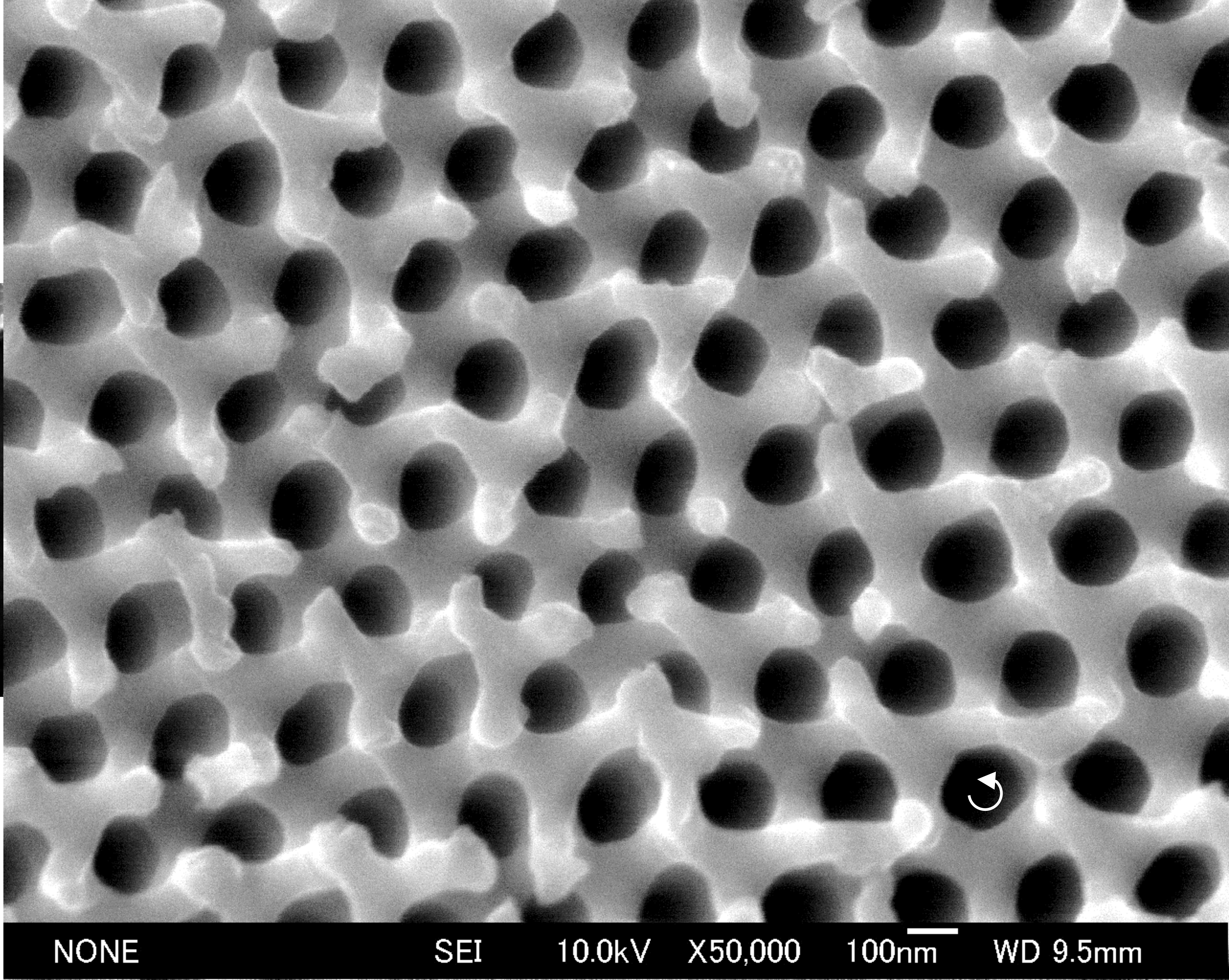

specimen No. 1
scale No. 3
domain No. 21
LH

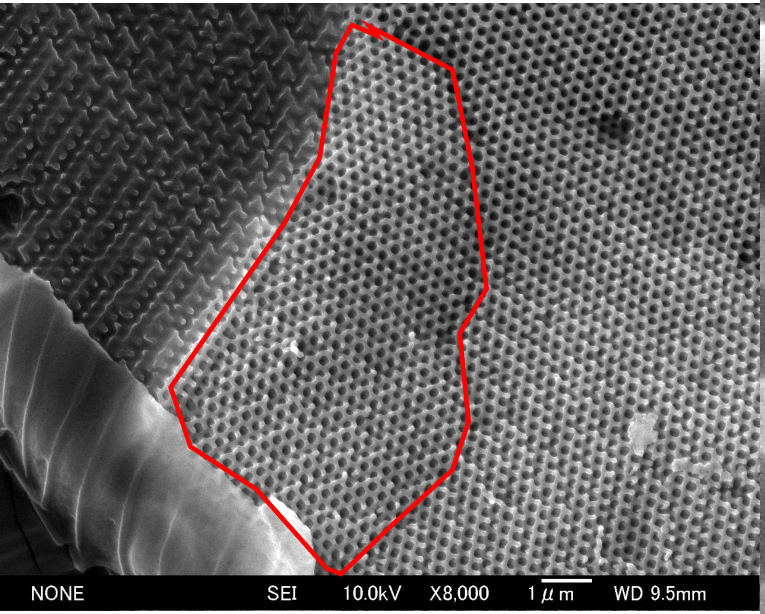

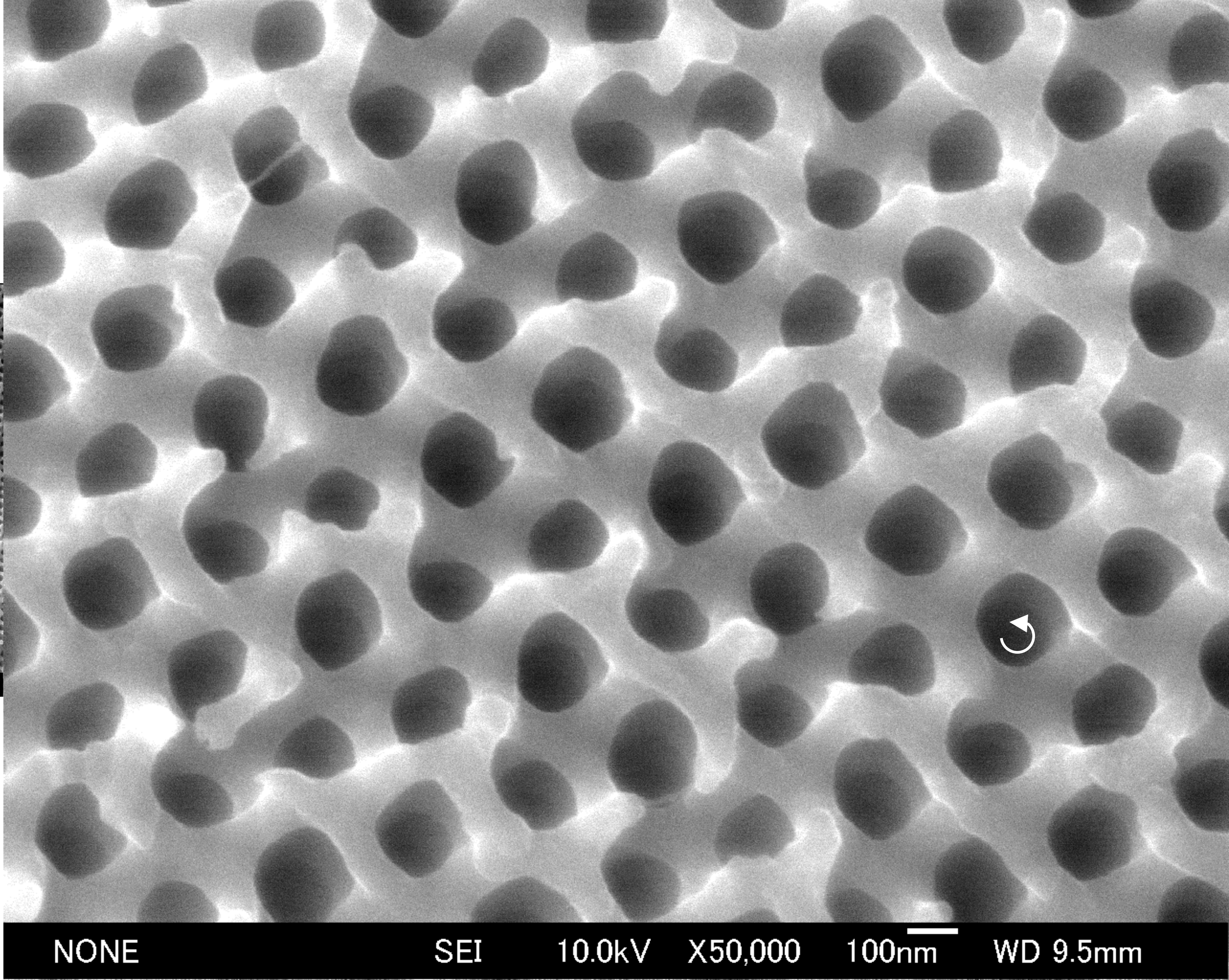

specimen No. 1
scale No. 4

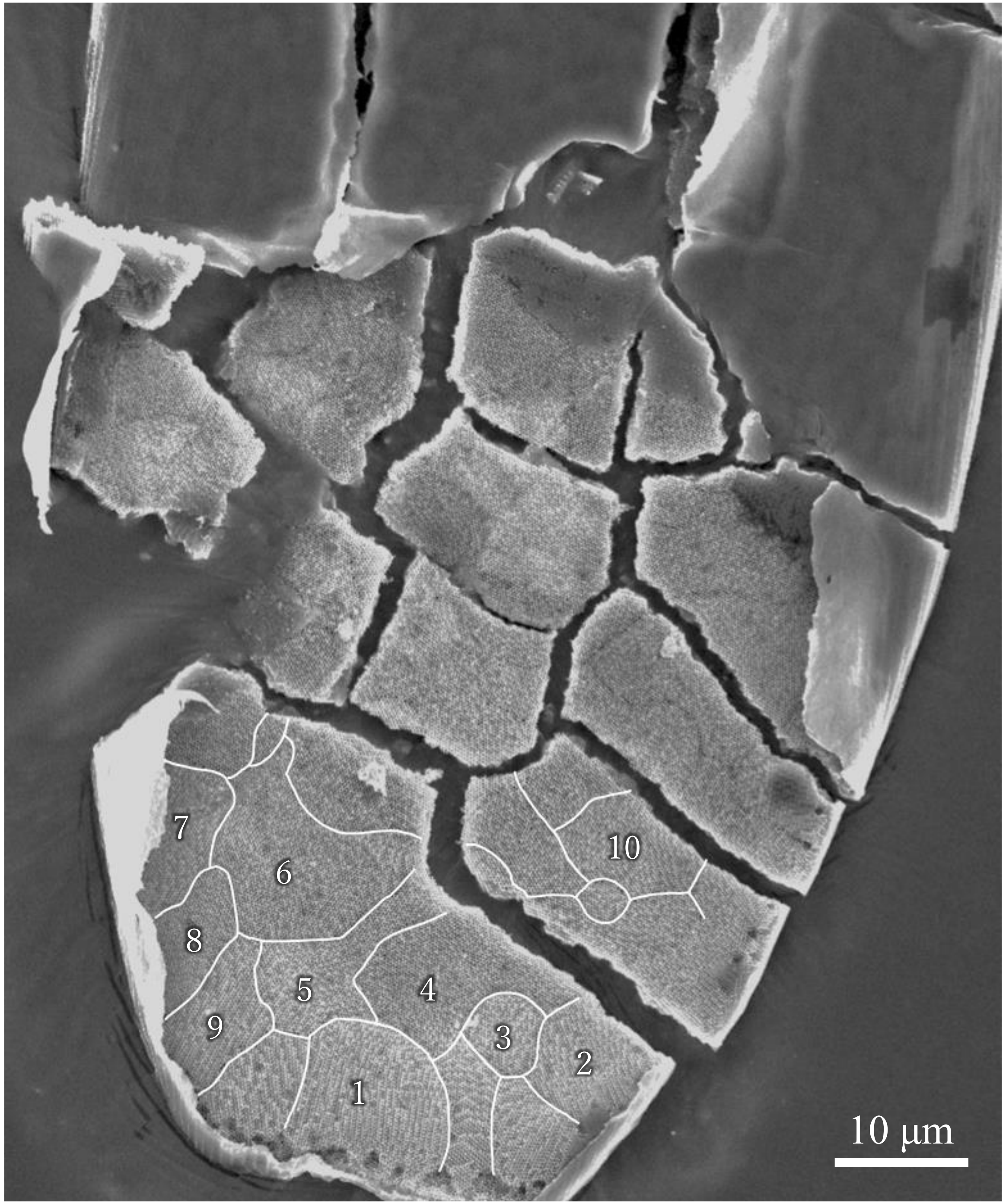

specimen No. 1
scale No. 4
domain No. 1
LH

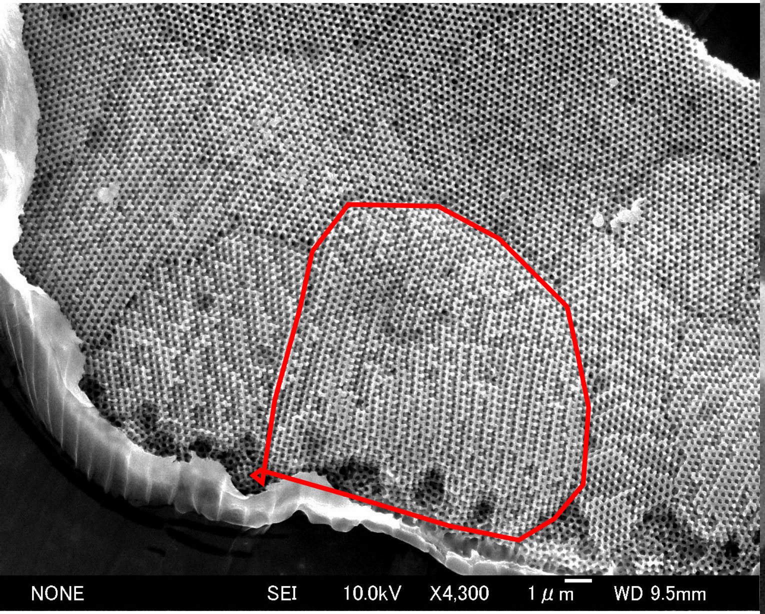

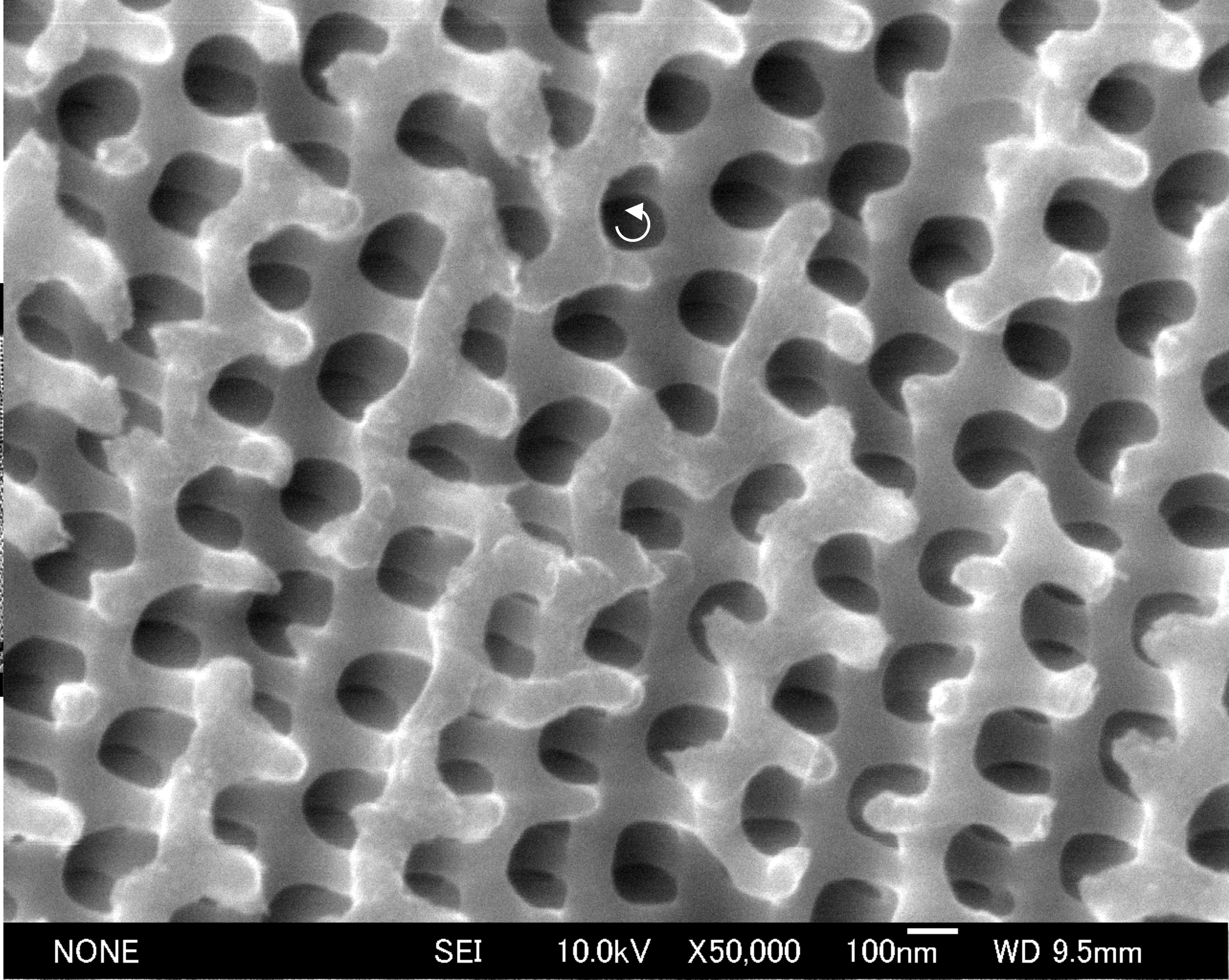

specimen No. 1
scale No. 4
domain No. 2
LH

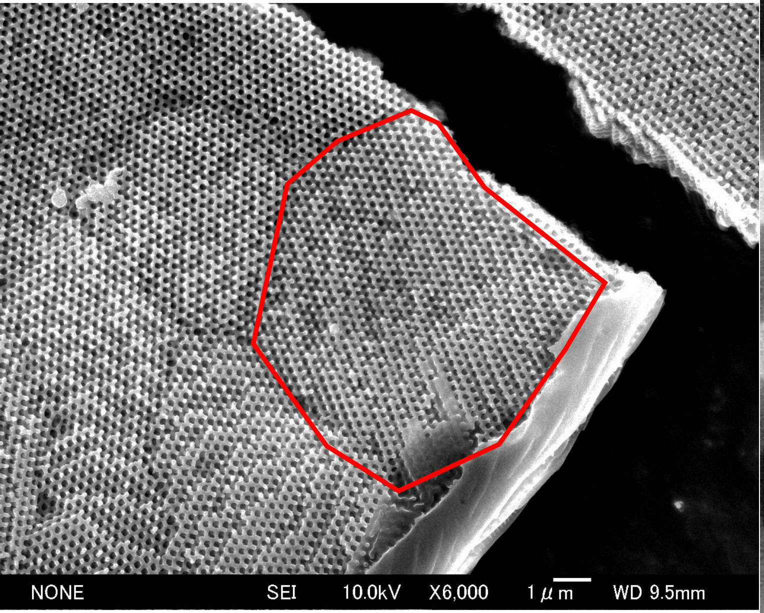

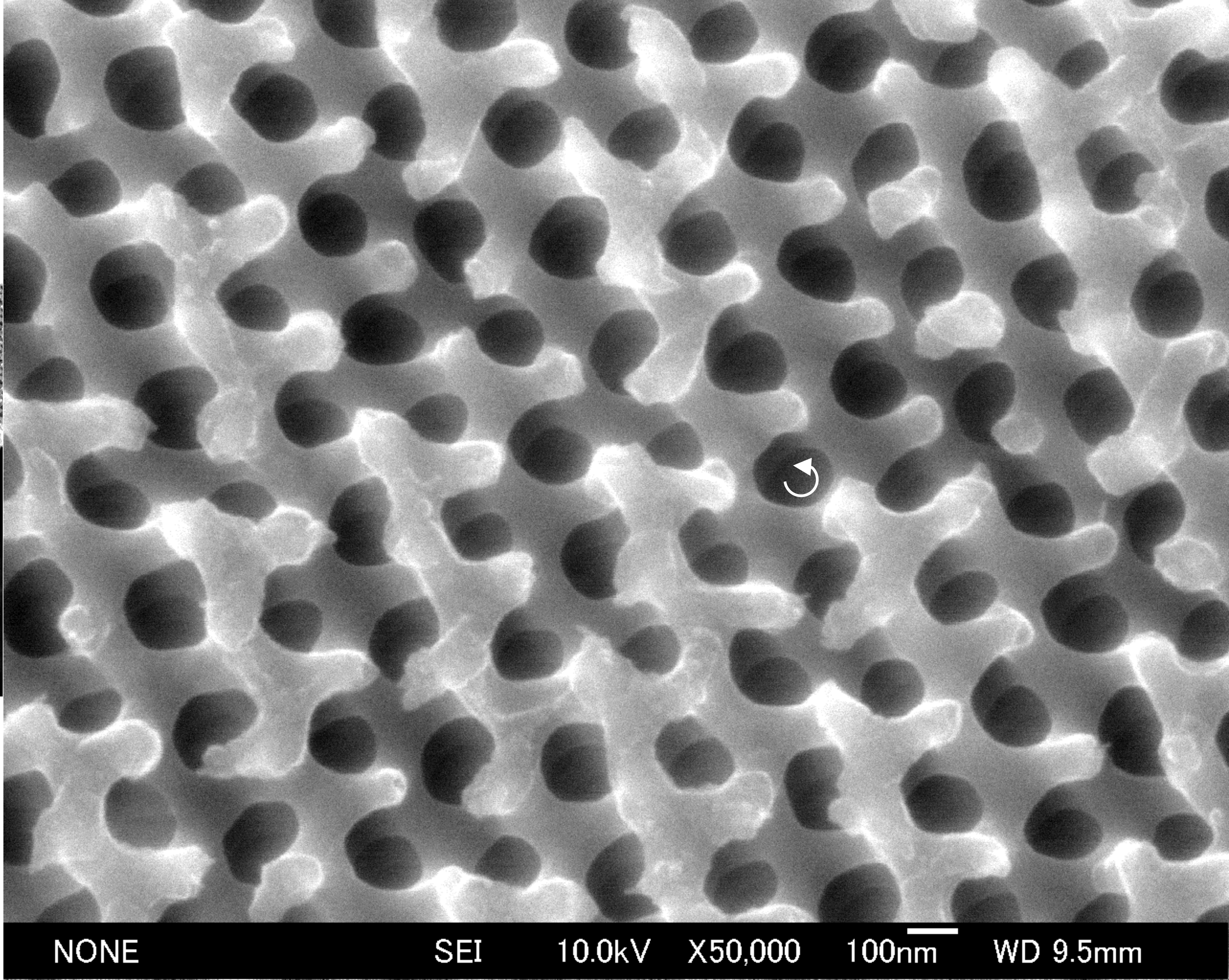

specimen No. 1
scale No. 4
domain No. 3
LH

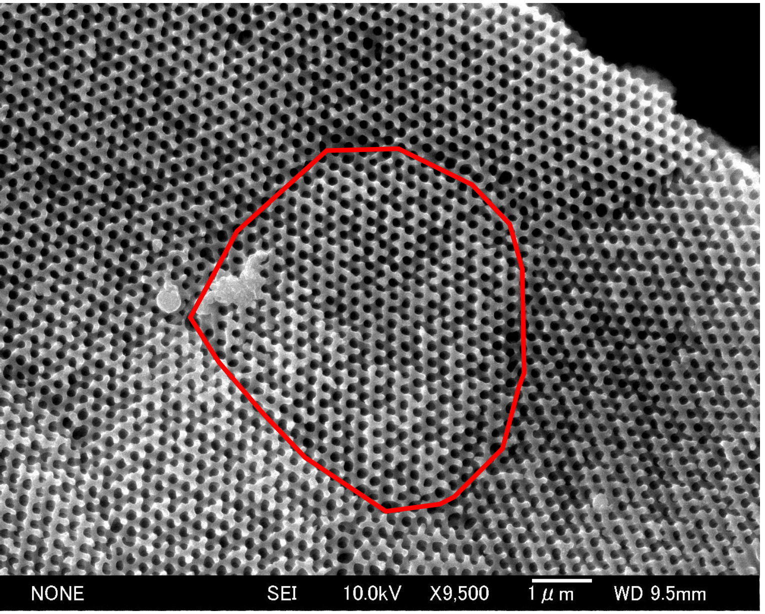

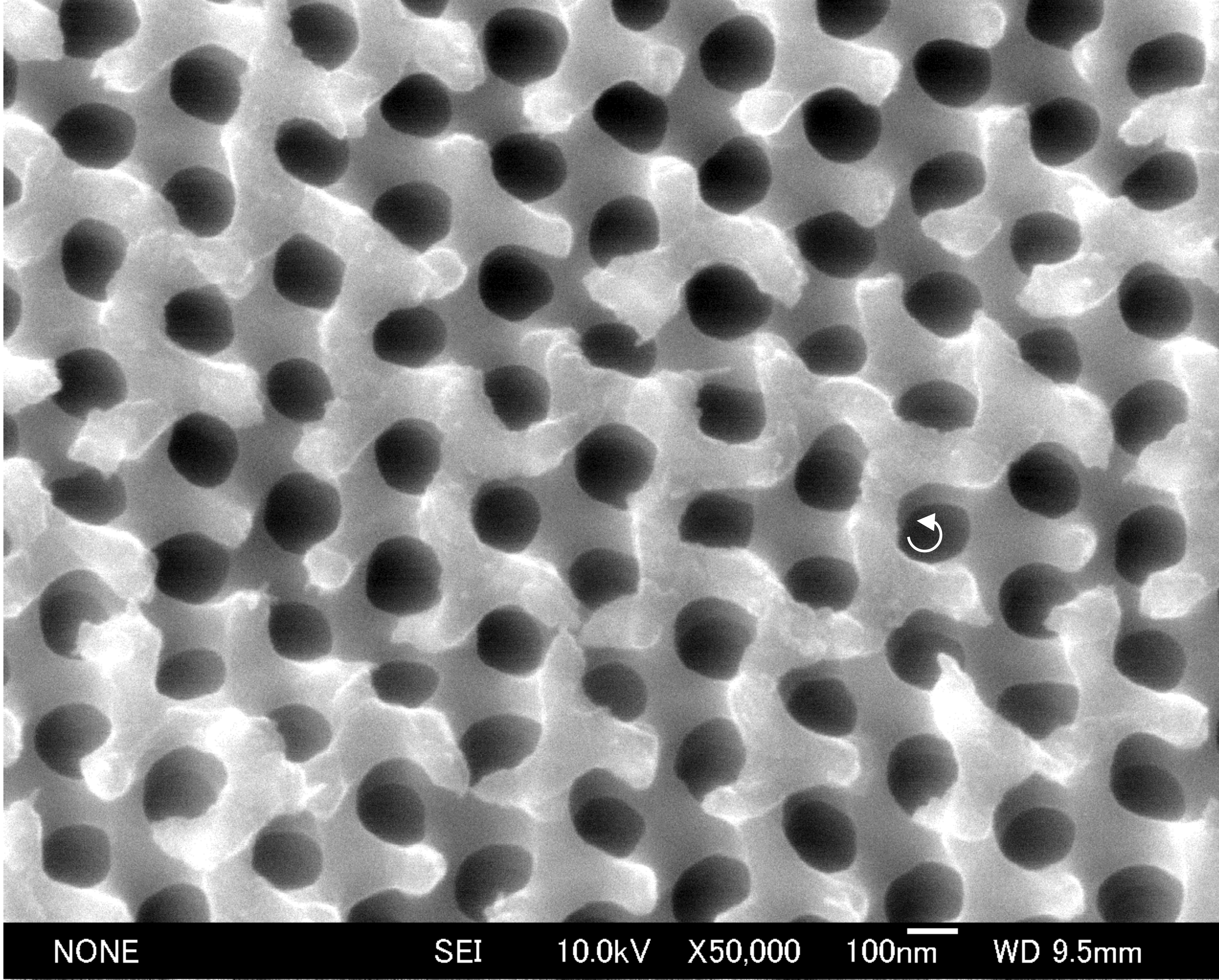

specimen No. 1
scale No. 4
domain No. 4
LH

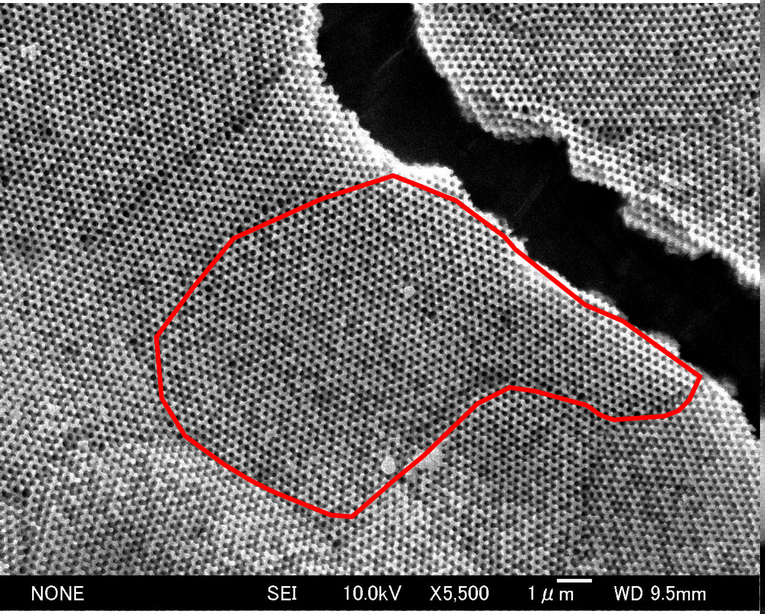

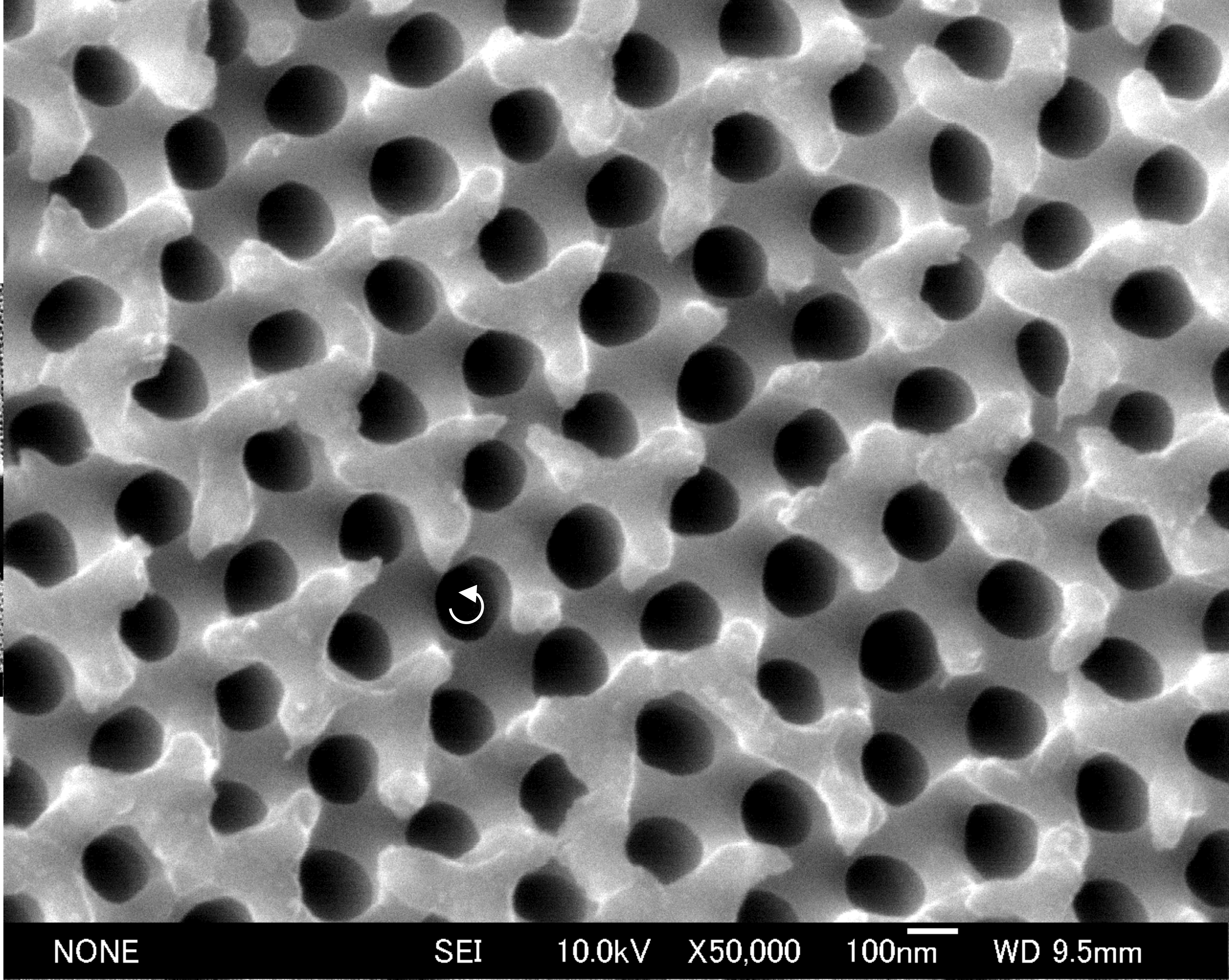

specimen No. 1
scale No. 4
domain No. 5
LH

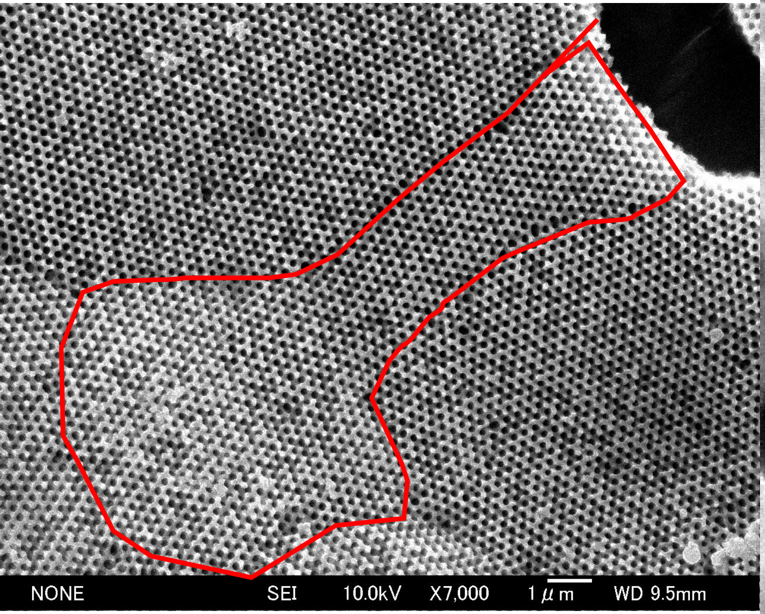

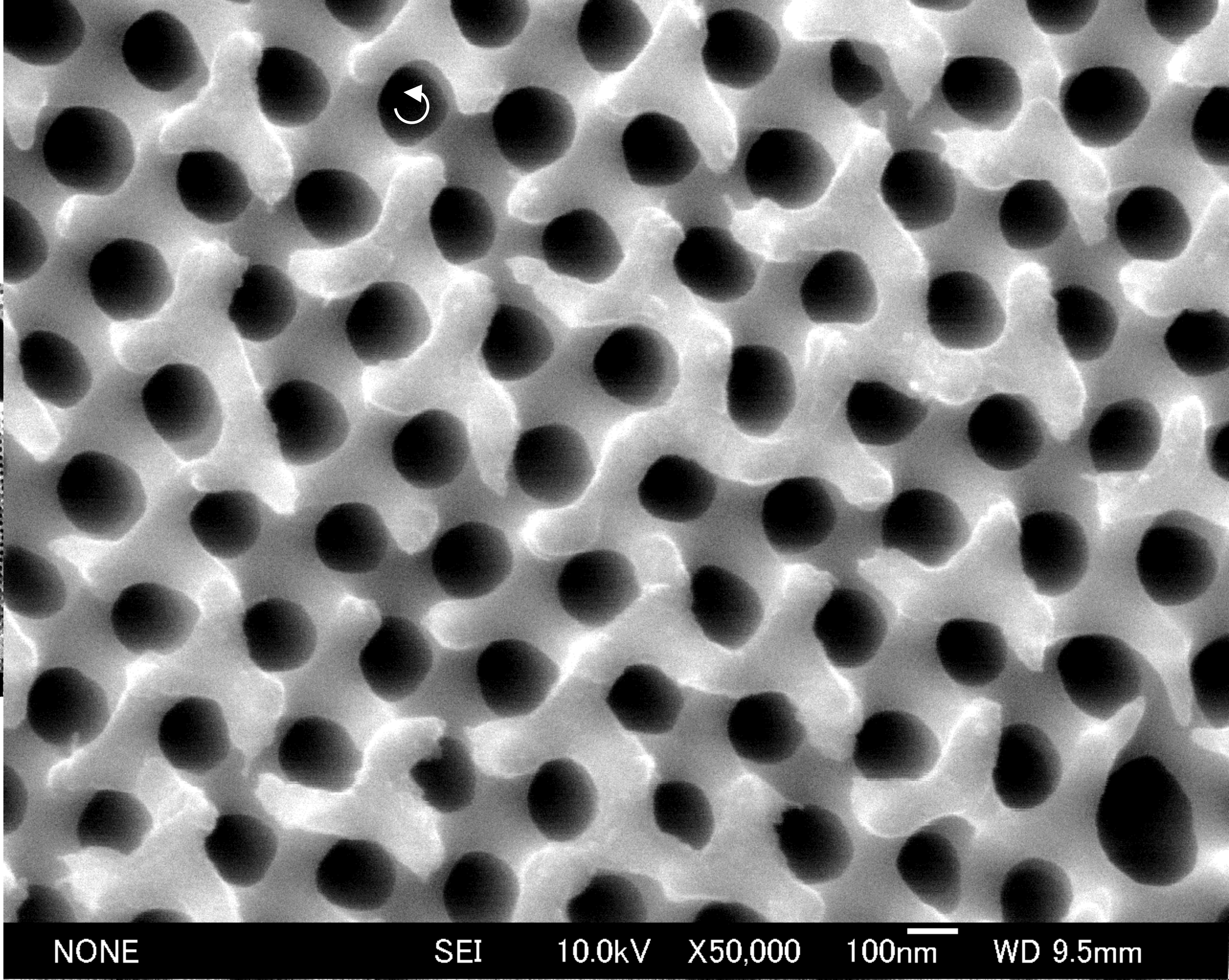

specimen No. 1
scale No. 4
domain No. 6
LH

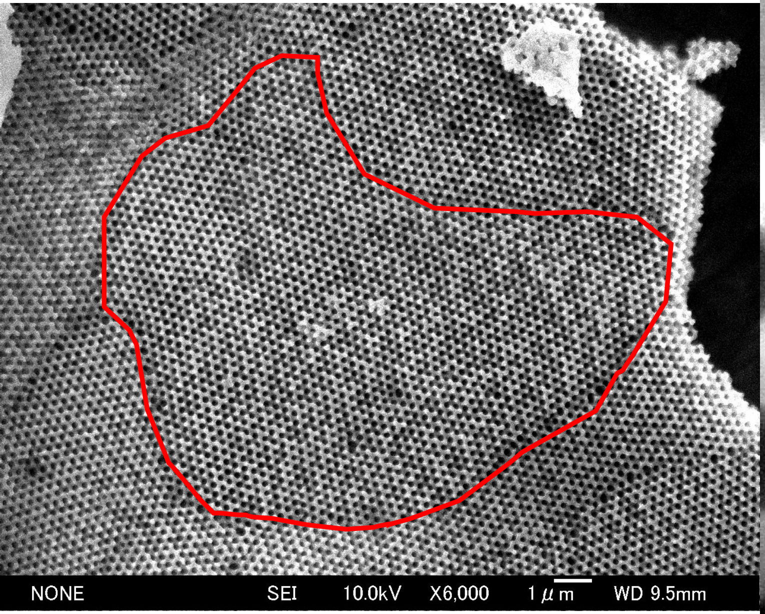

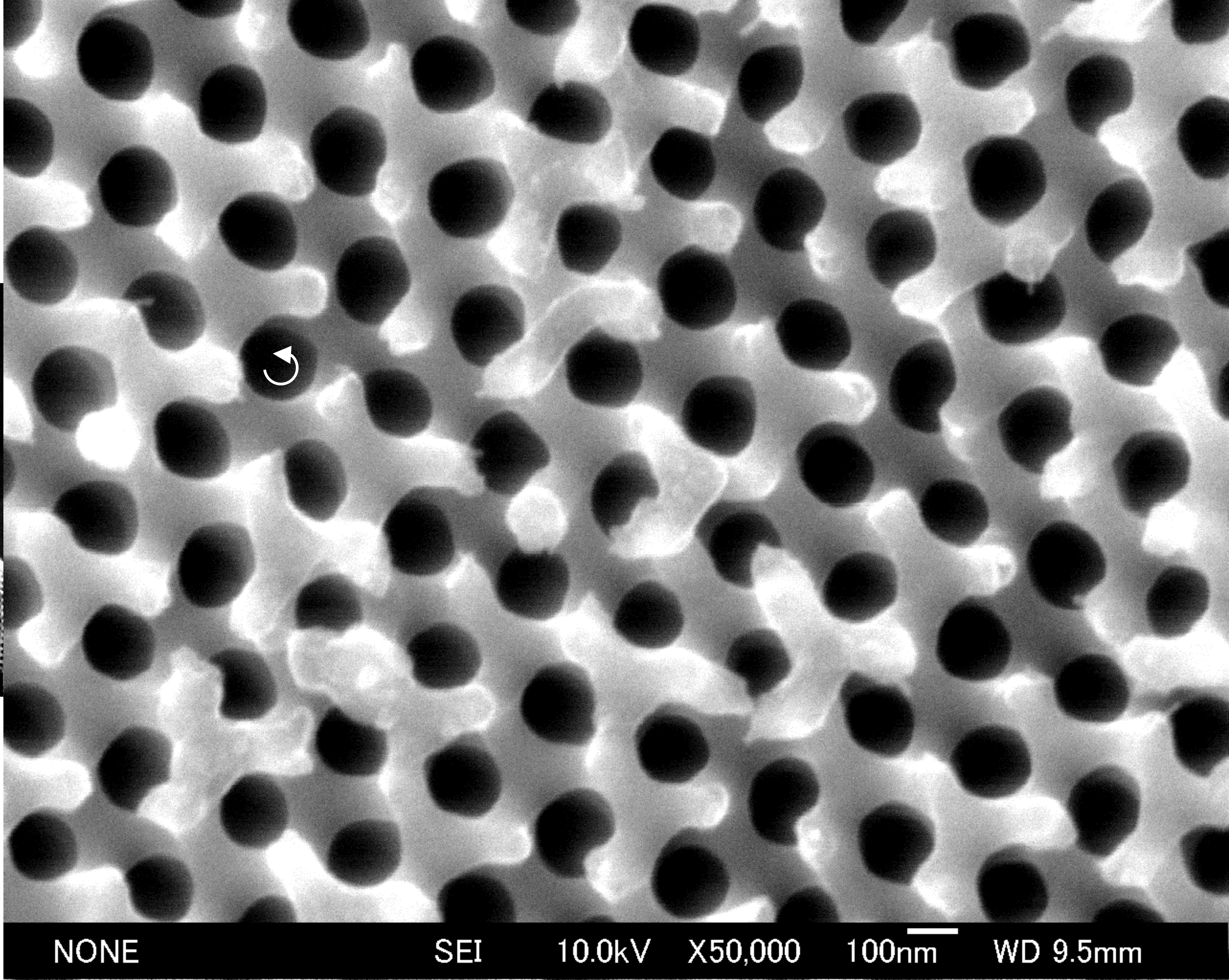

specimen No. 1
scale No. 4
domain No. 7
LH

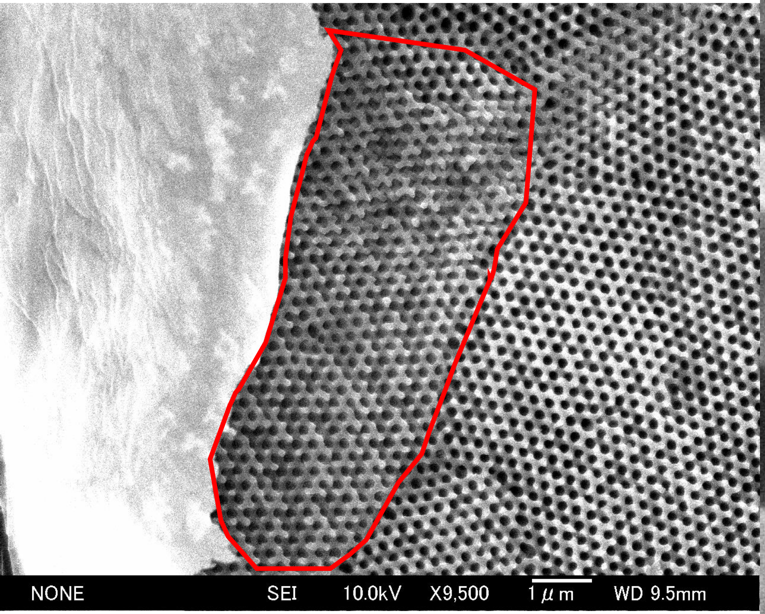

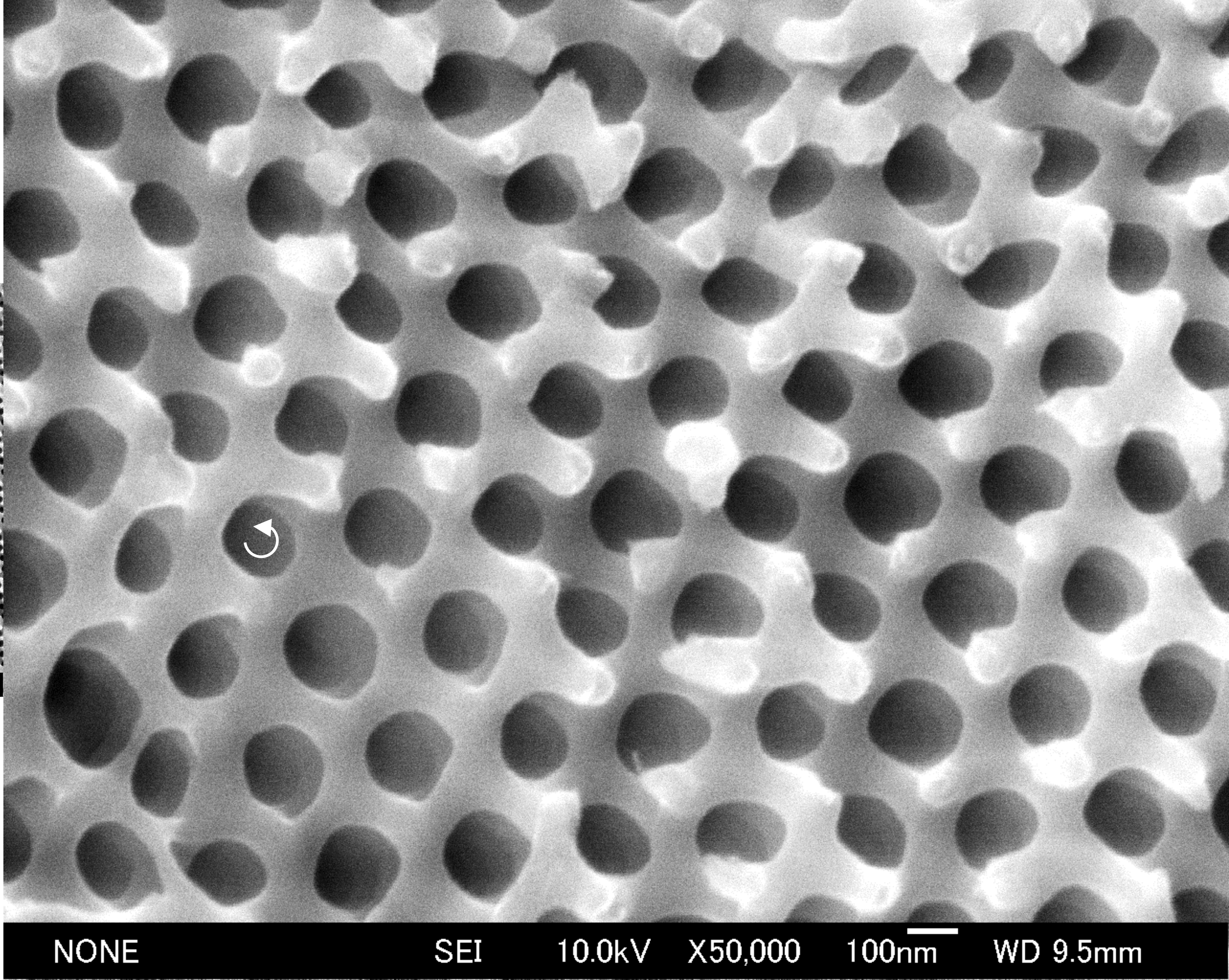

specimen No. 1
scale No. 4
domain No. 8
LH

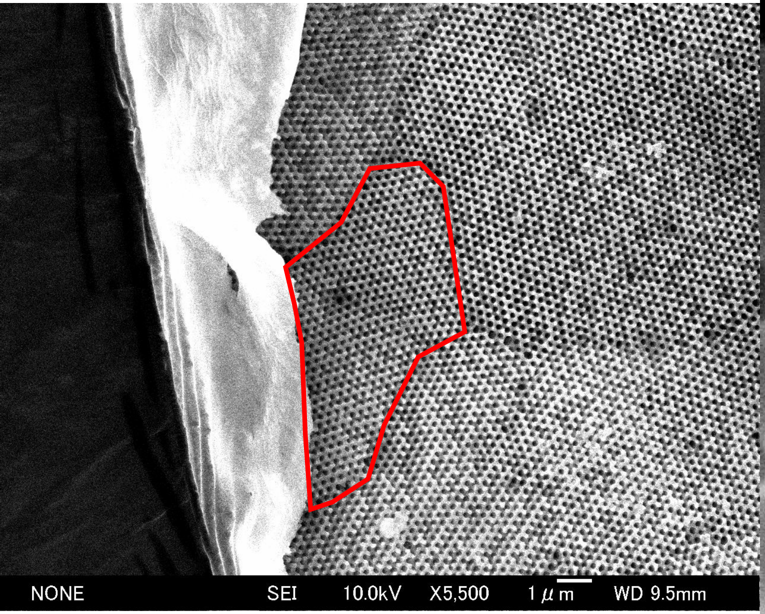

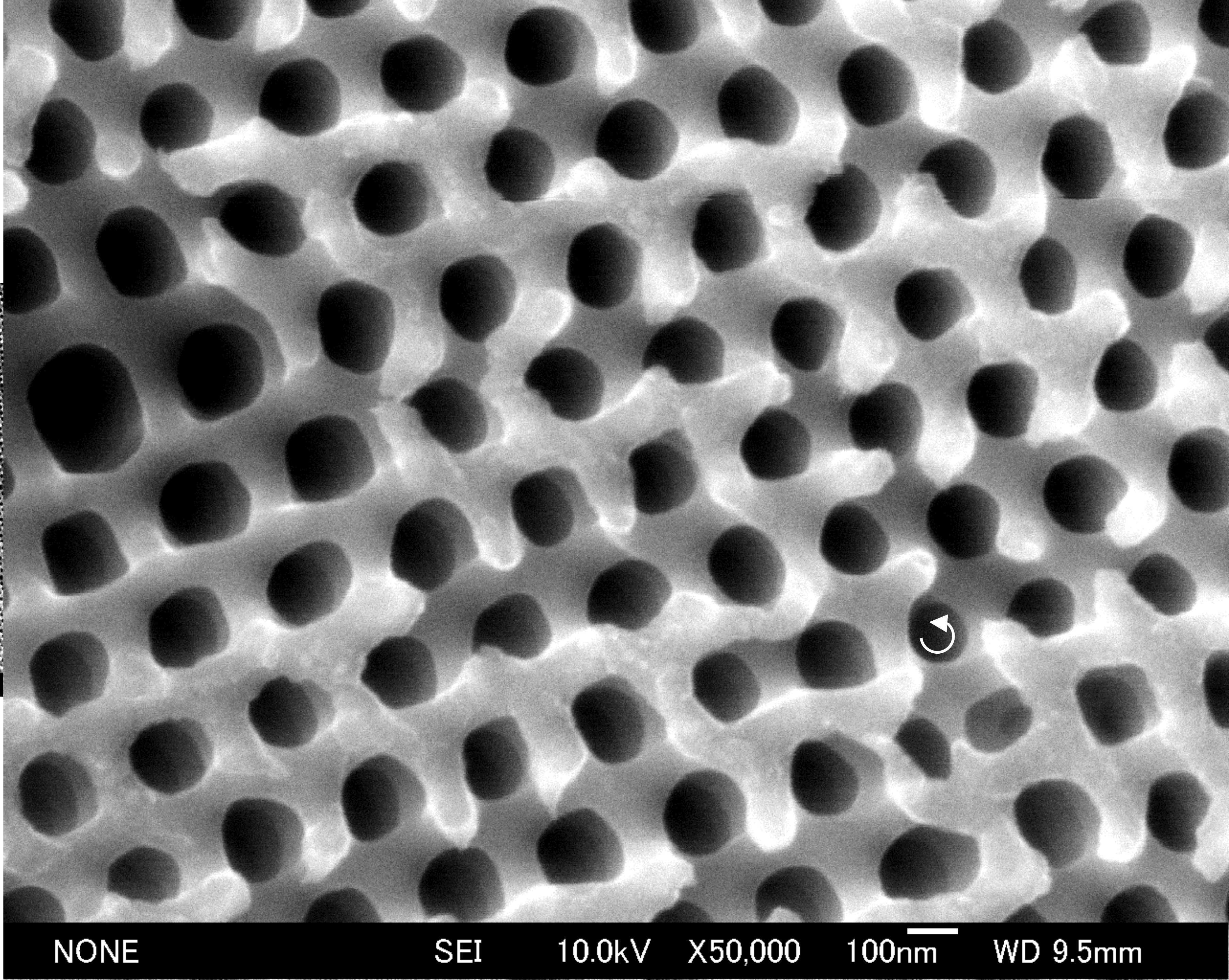

specimen No. 1
scale No. 4
domain No. 9
LH

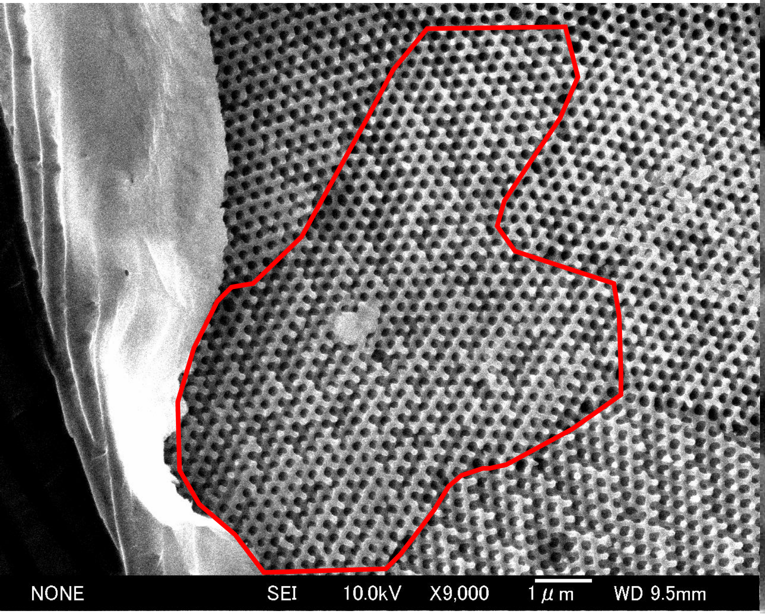

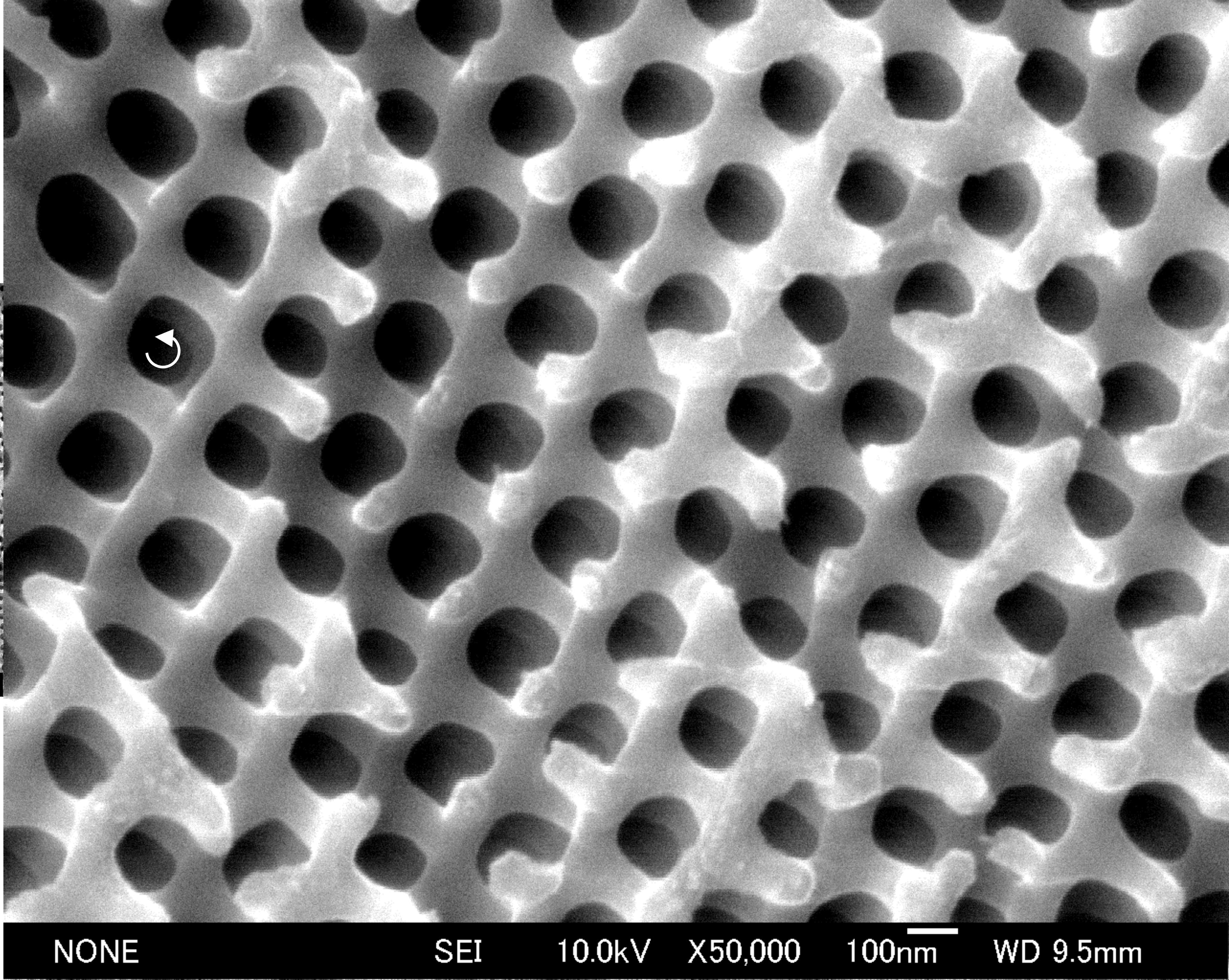

specimen No. 1
scale No. 4
domain No. 10
LH

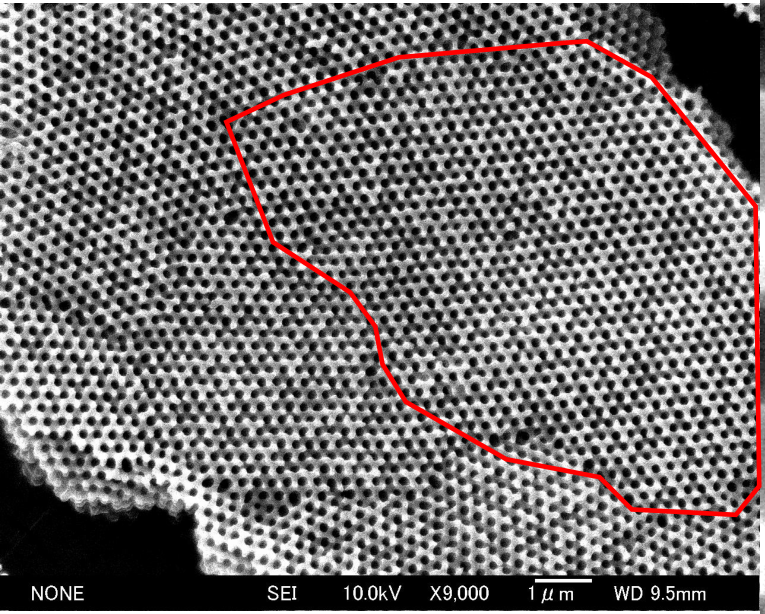

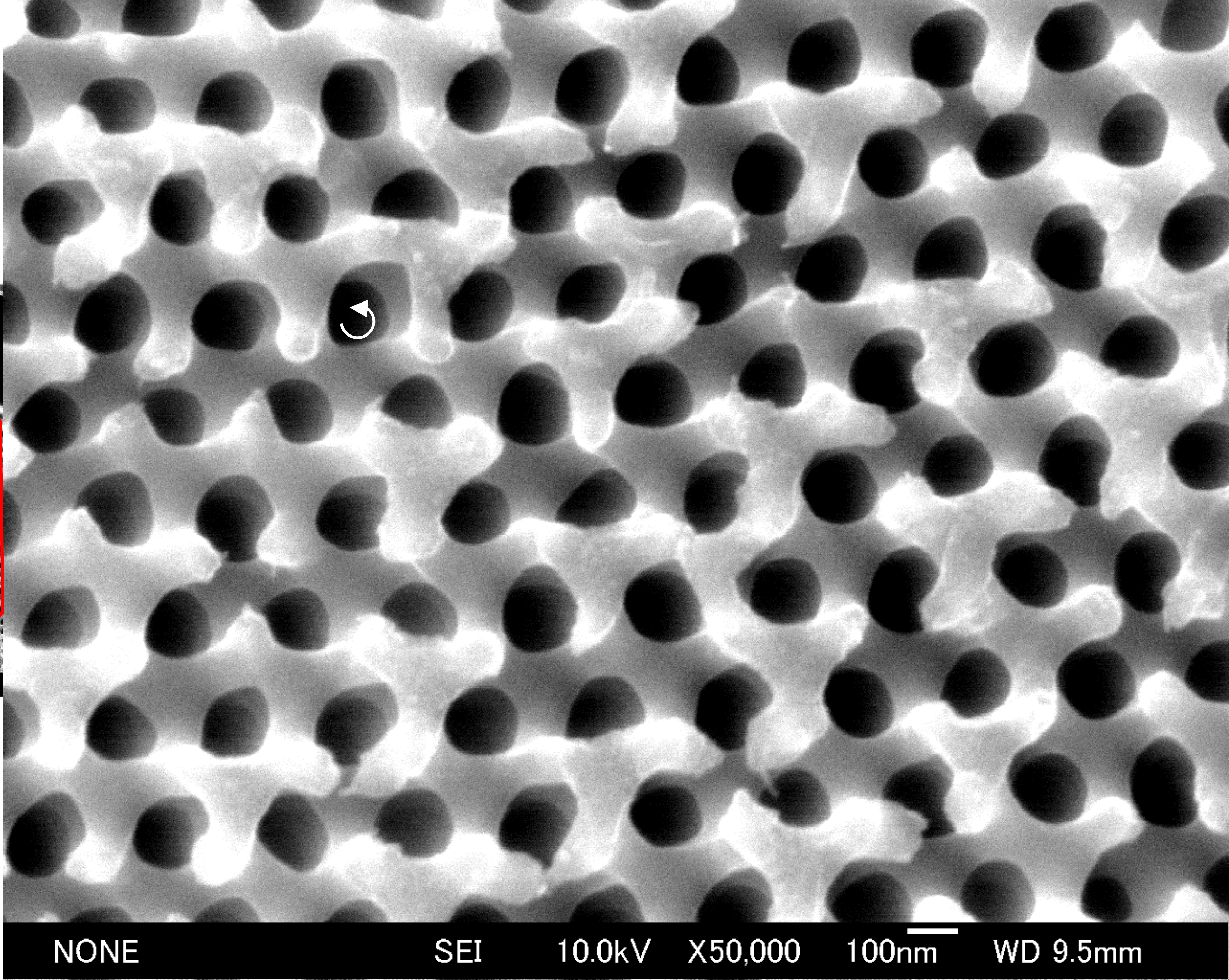

specimen No. 1
scale No. 5

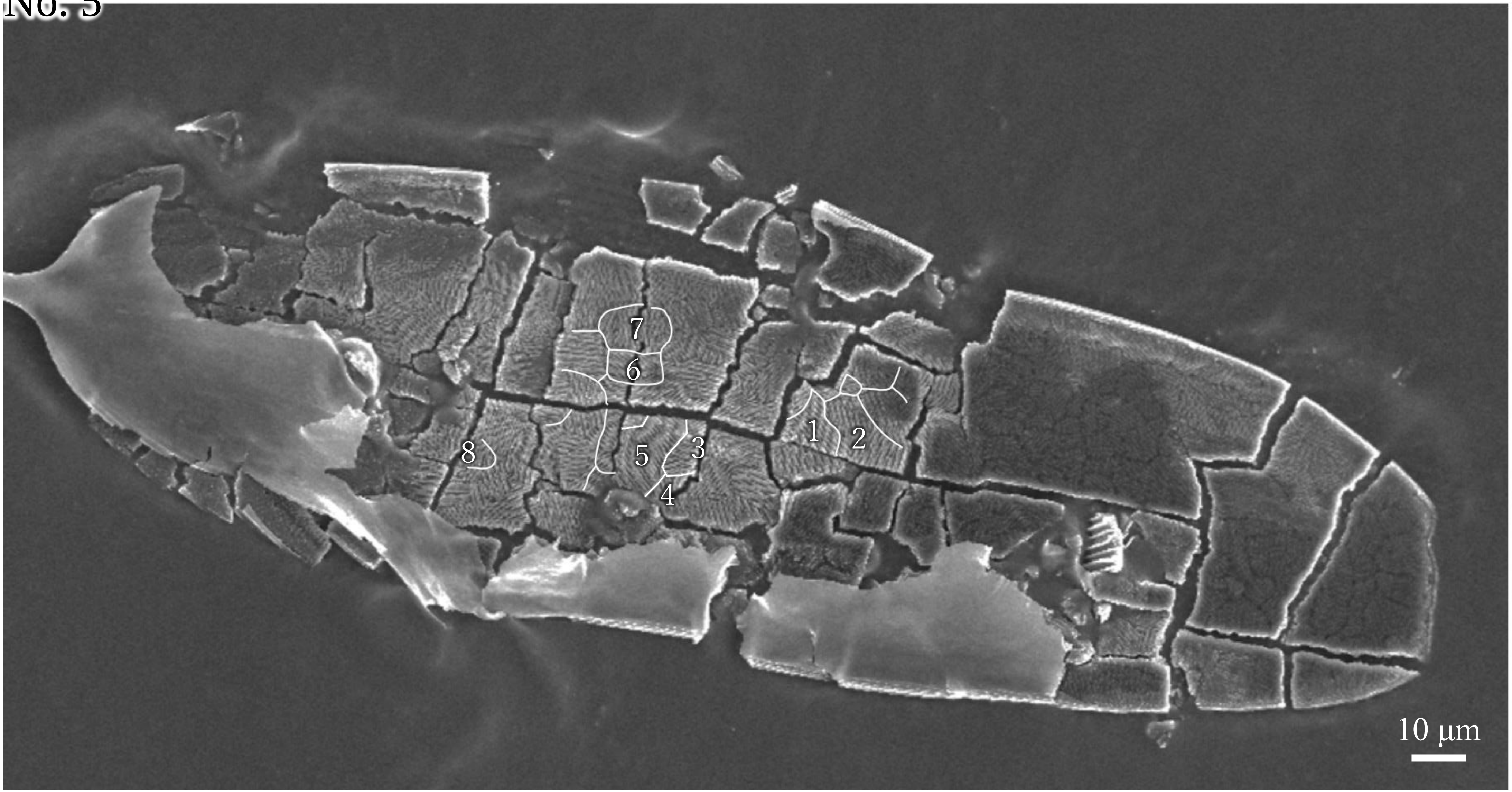

specimen No. 1
scale No. 5
domain No. 1
LH

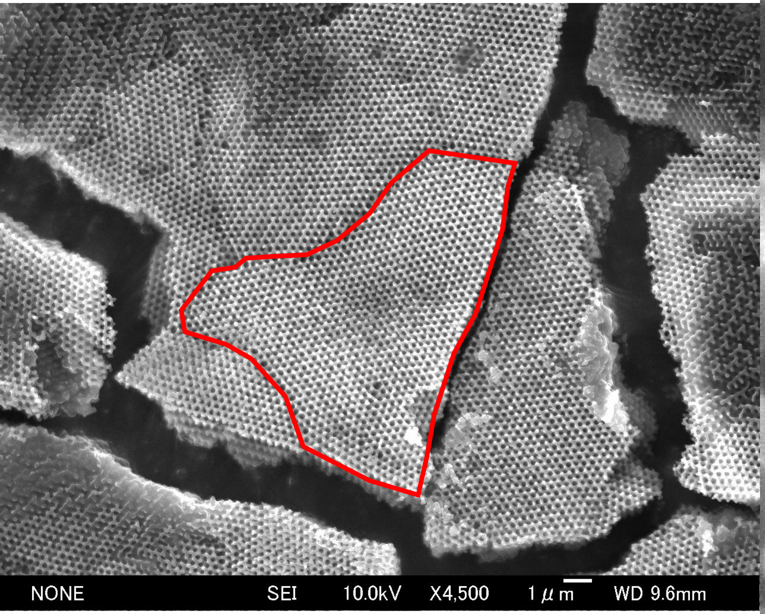

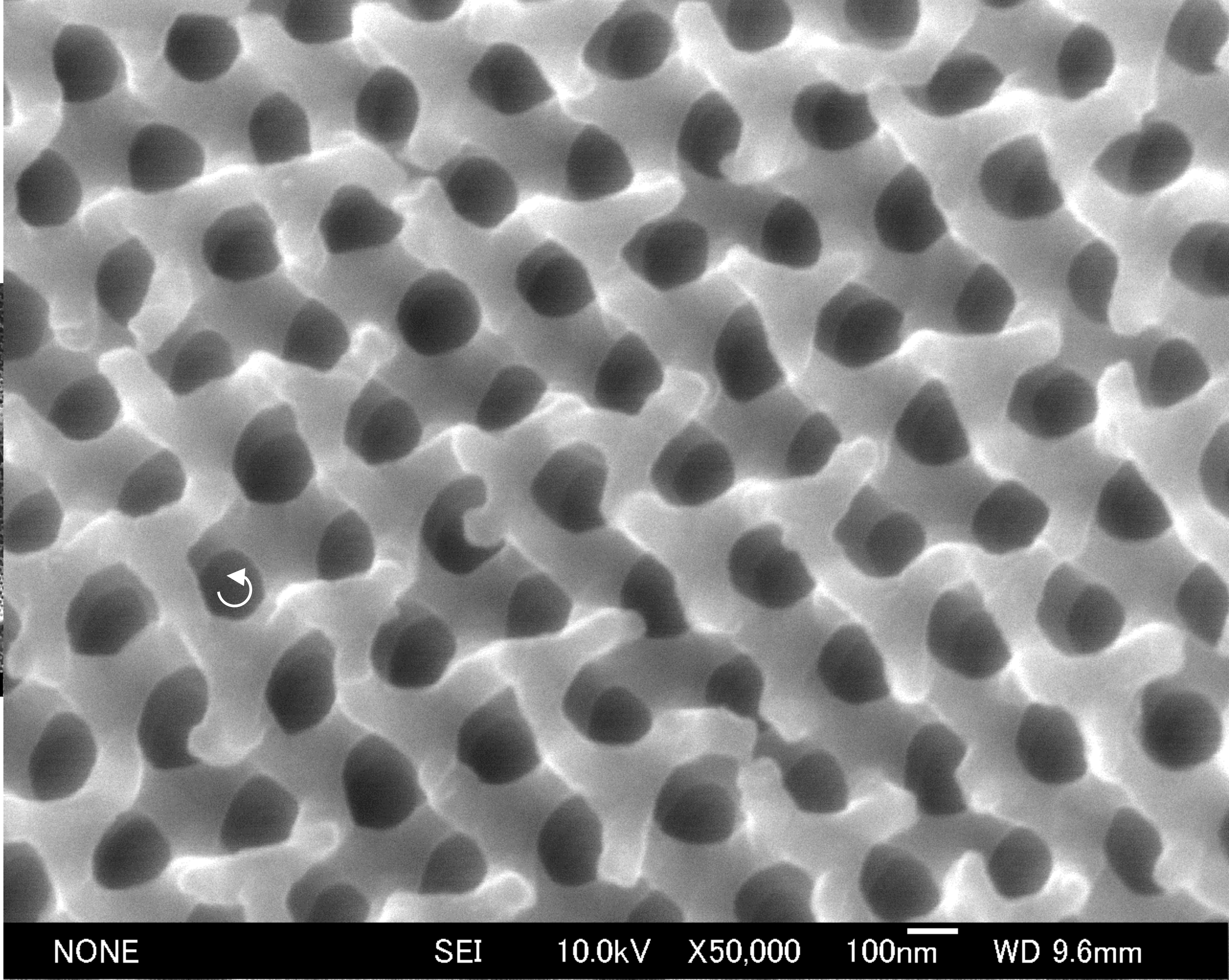

specimen No. 1
scale No. 5
domain No. 2
LH

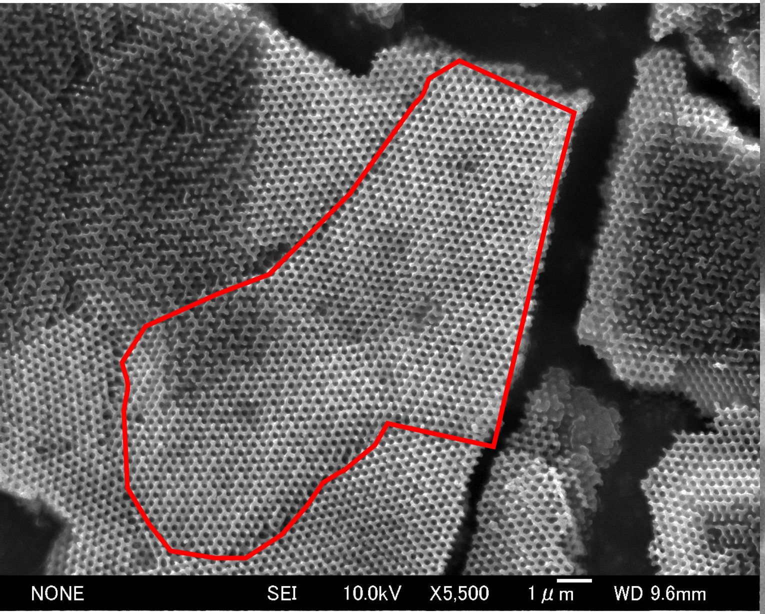

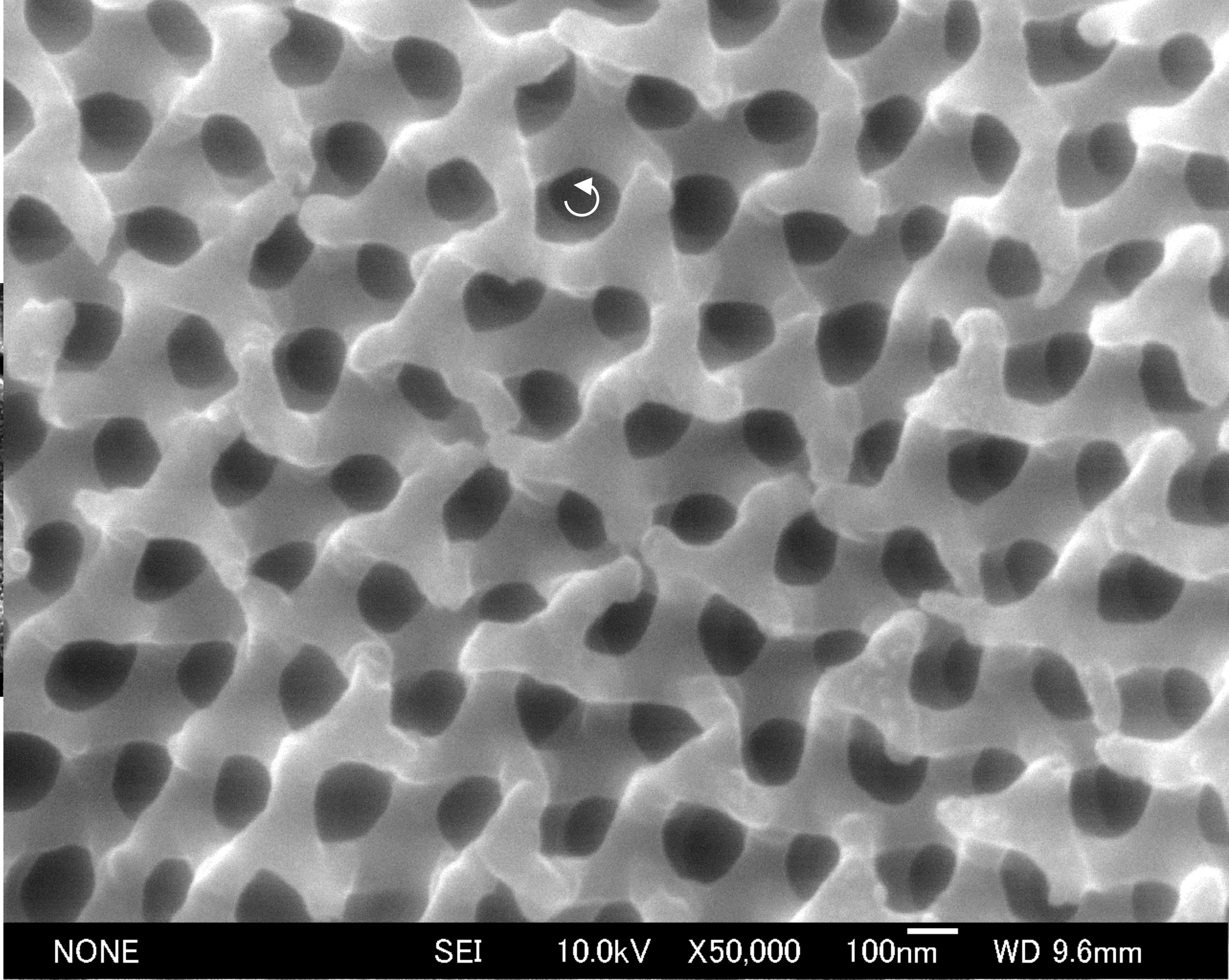

specimen No. 1
scale No. 5
domain No. 3
LH

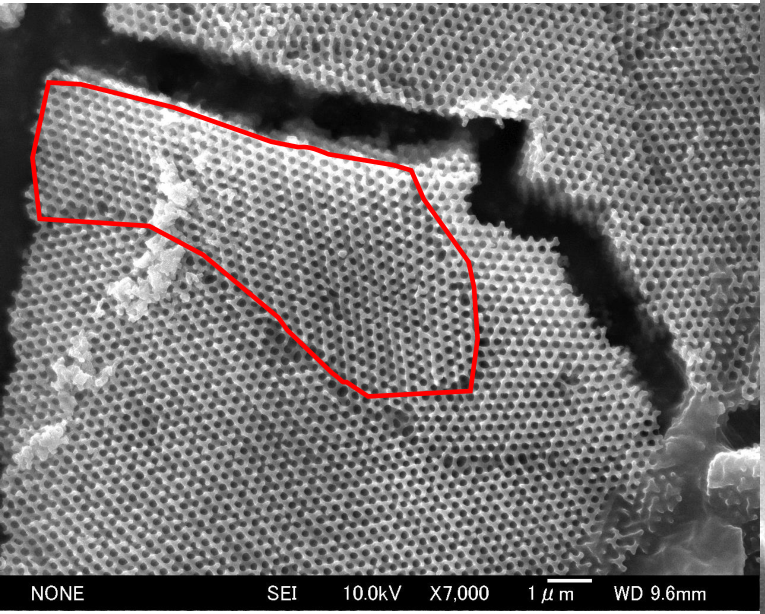

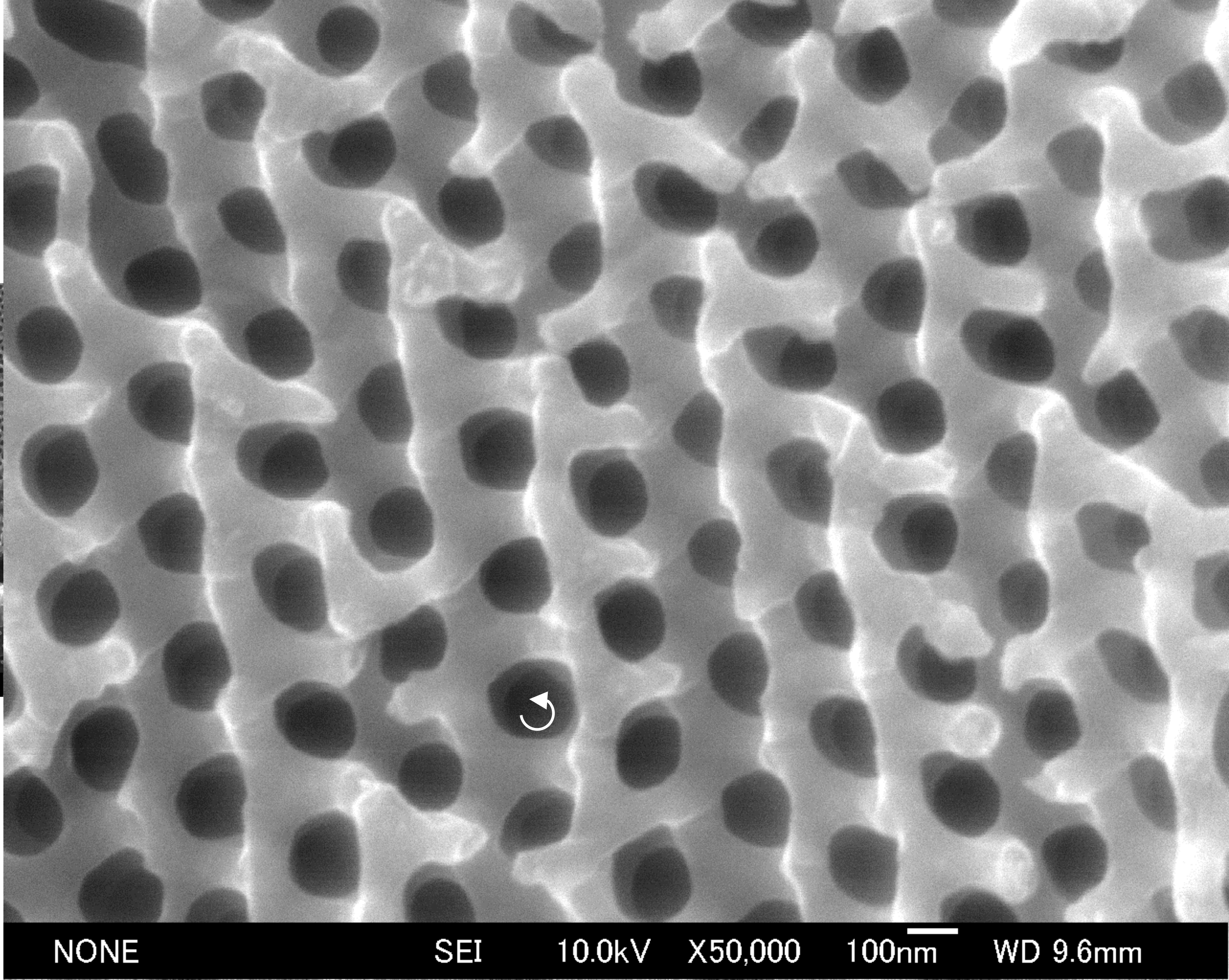

specimen No. 1
scale No. 5
domain No. 4
LH

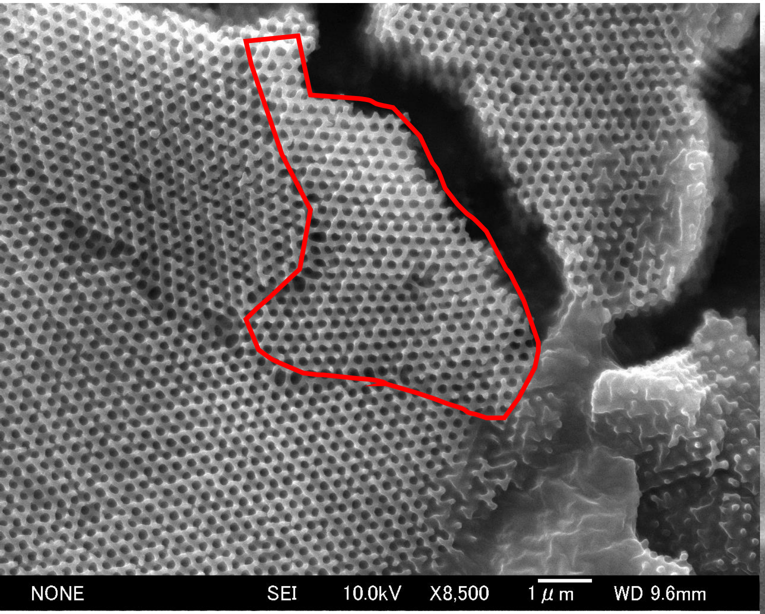

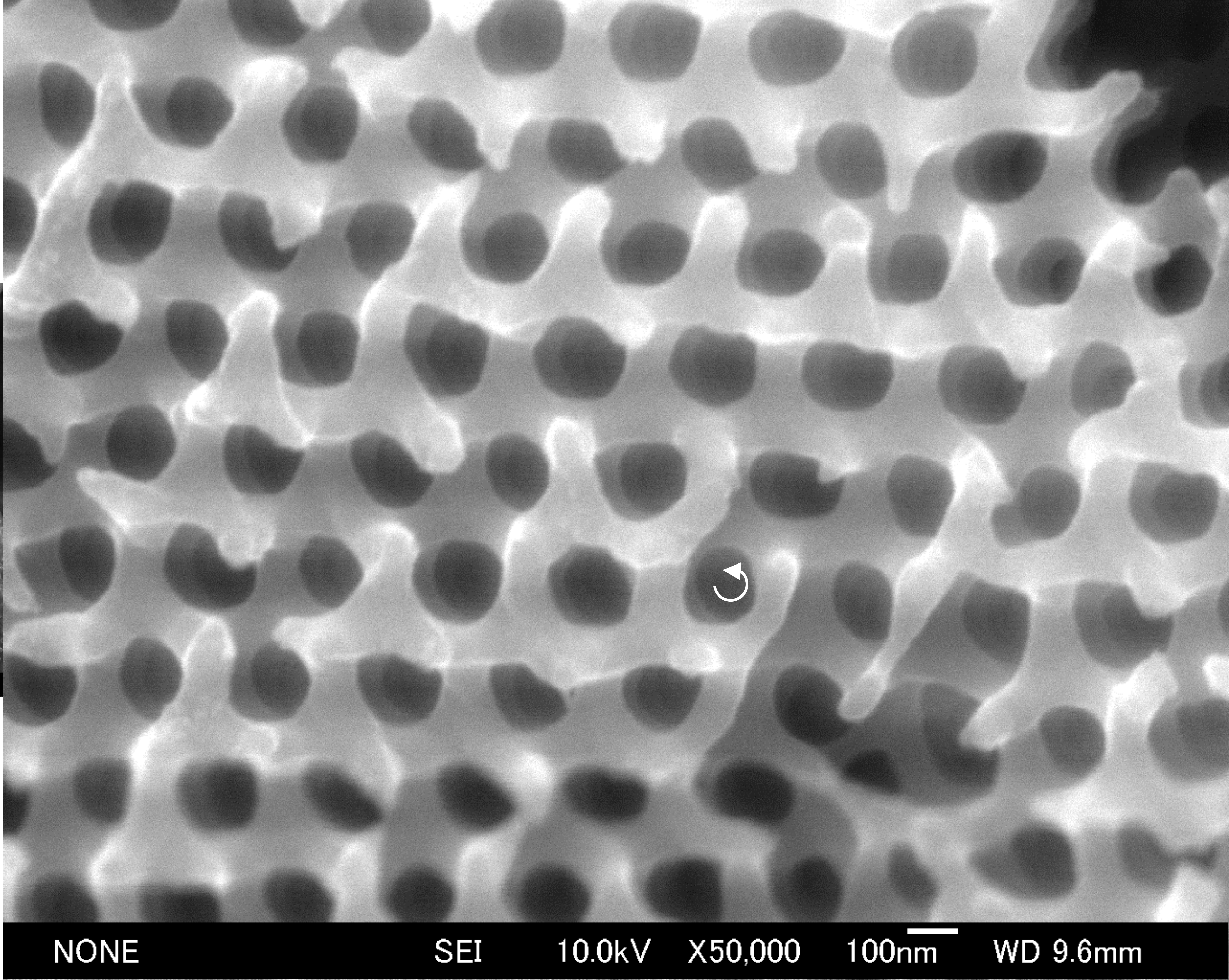

specimen No. 1
scale No. 5
domain No. 5
LH

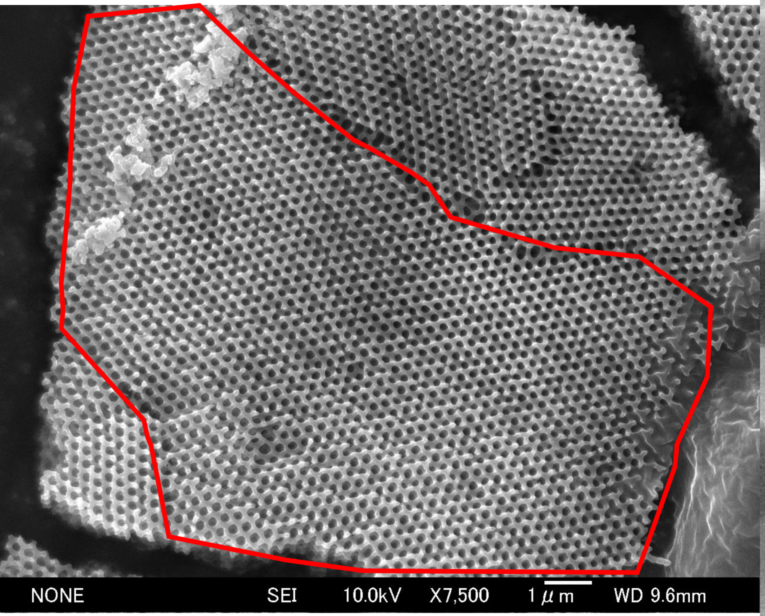

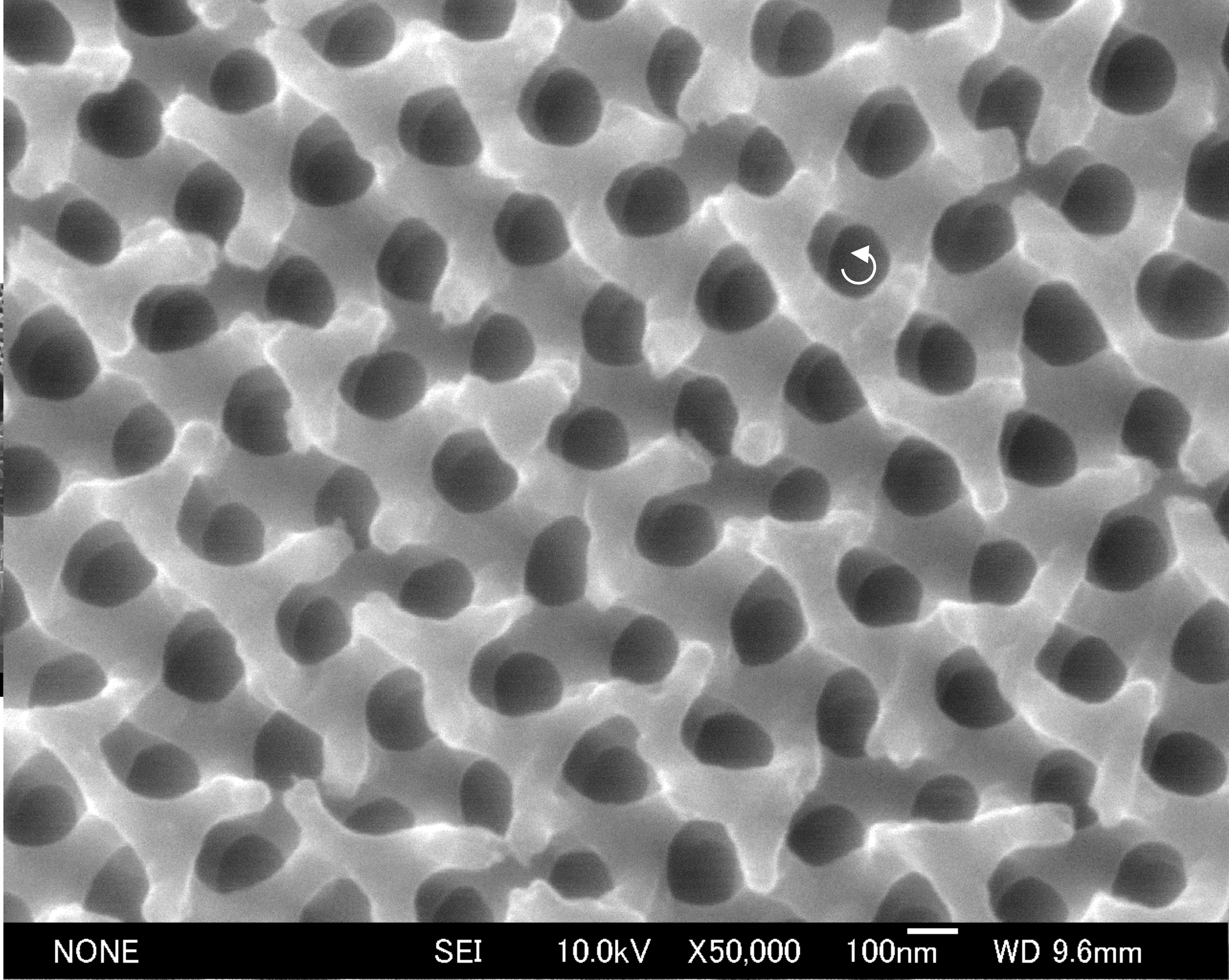

specimen No. 1
scale No. 5
domain No. 6
LH

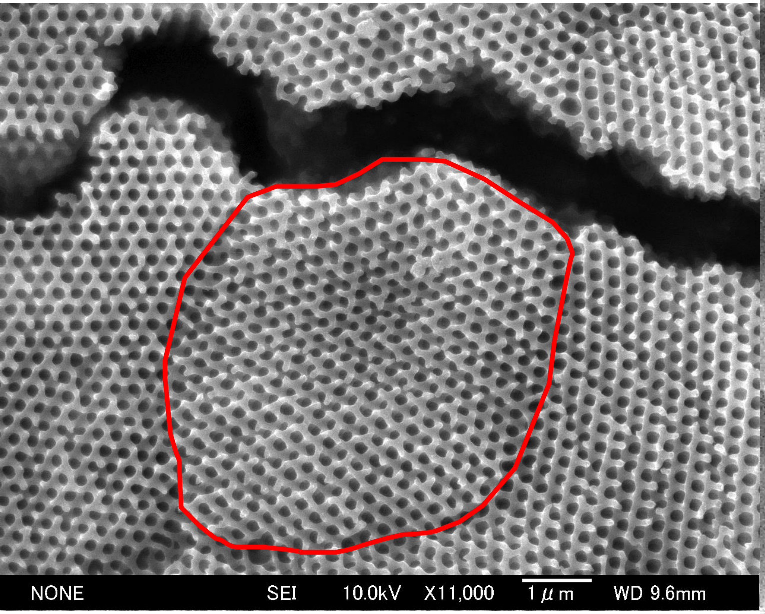

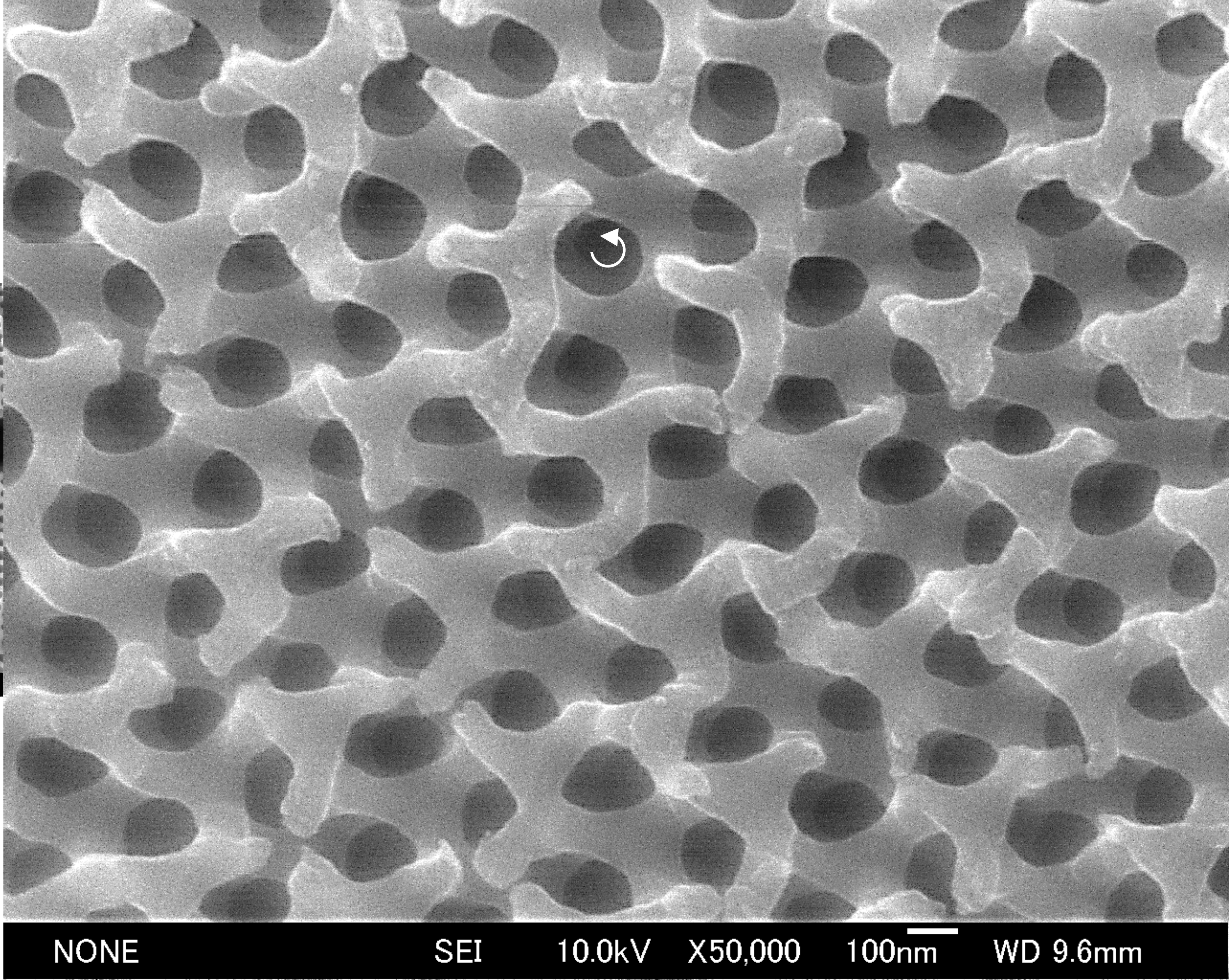

specimen No. 1
scale No. 5
domain No. 7
LH

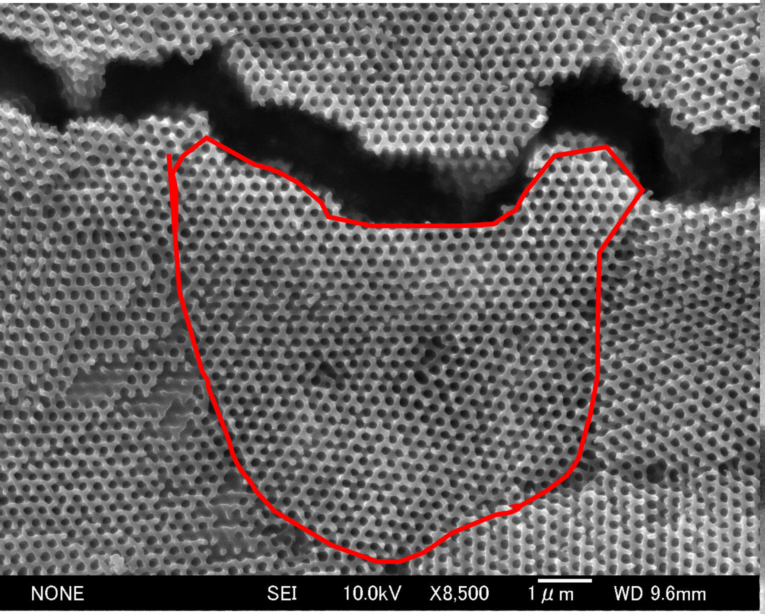

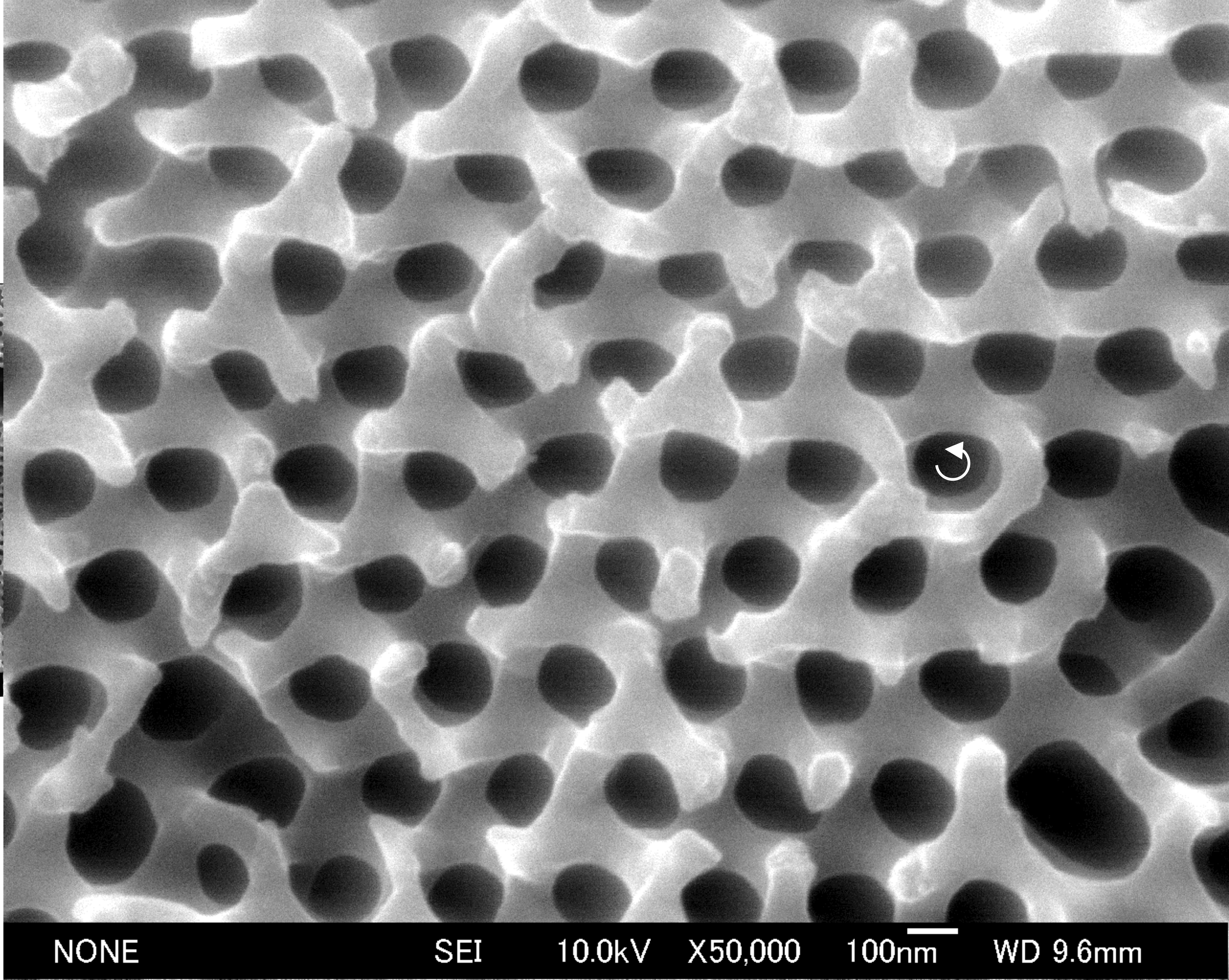

specimen No. 1
scale No. 5
domain No. 8
LH

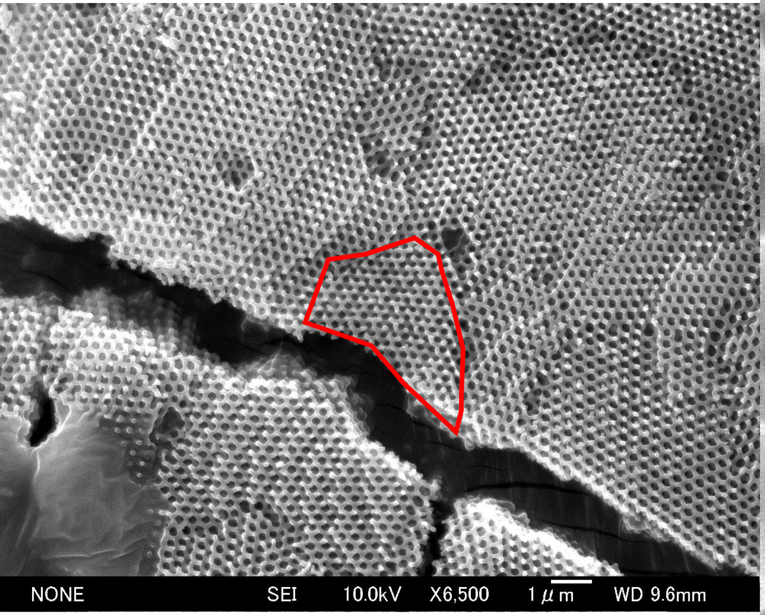

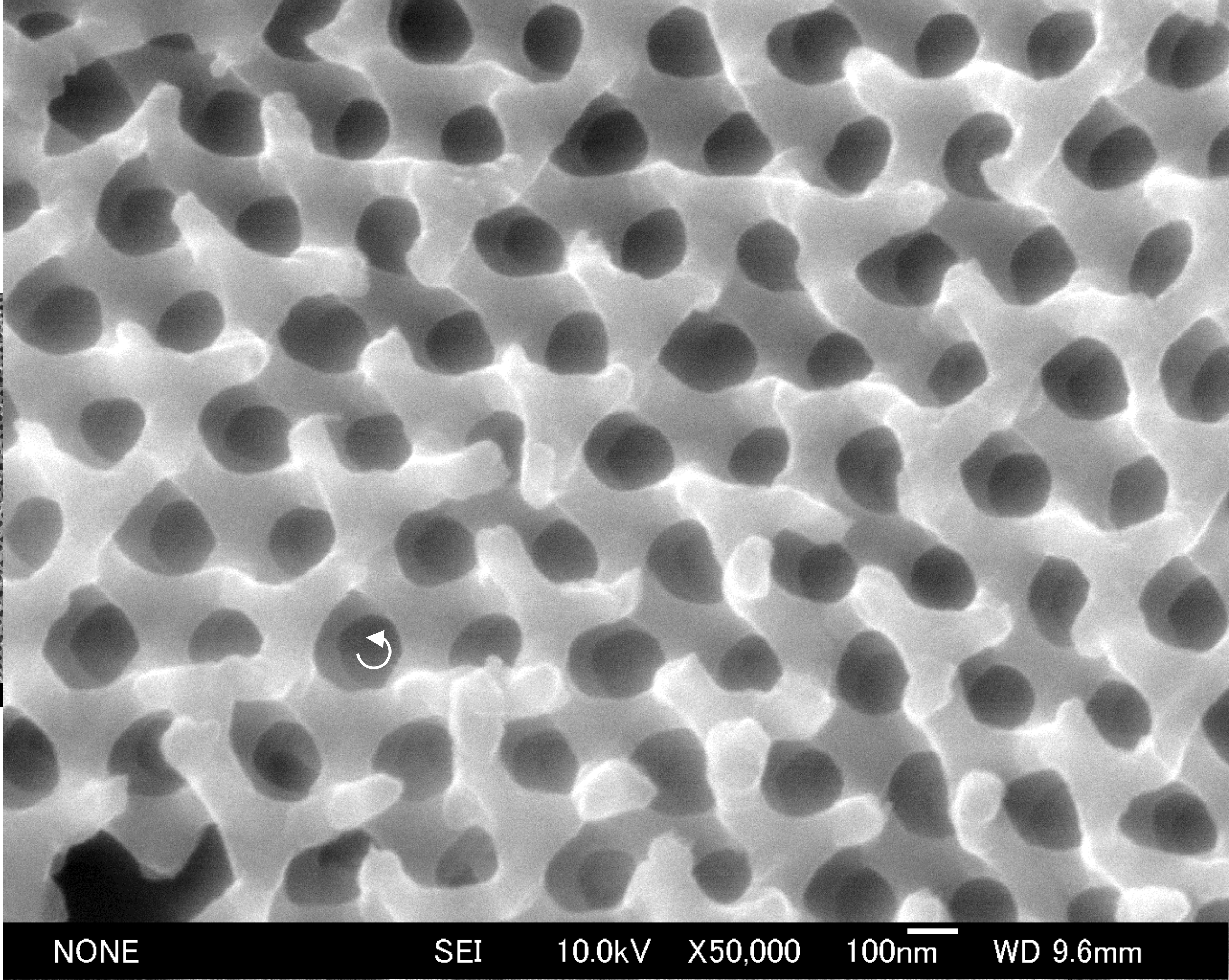

specimen No. 2
scale No. 6

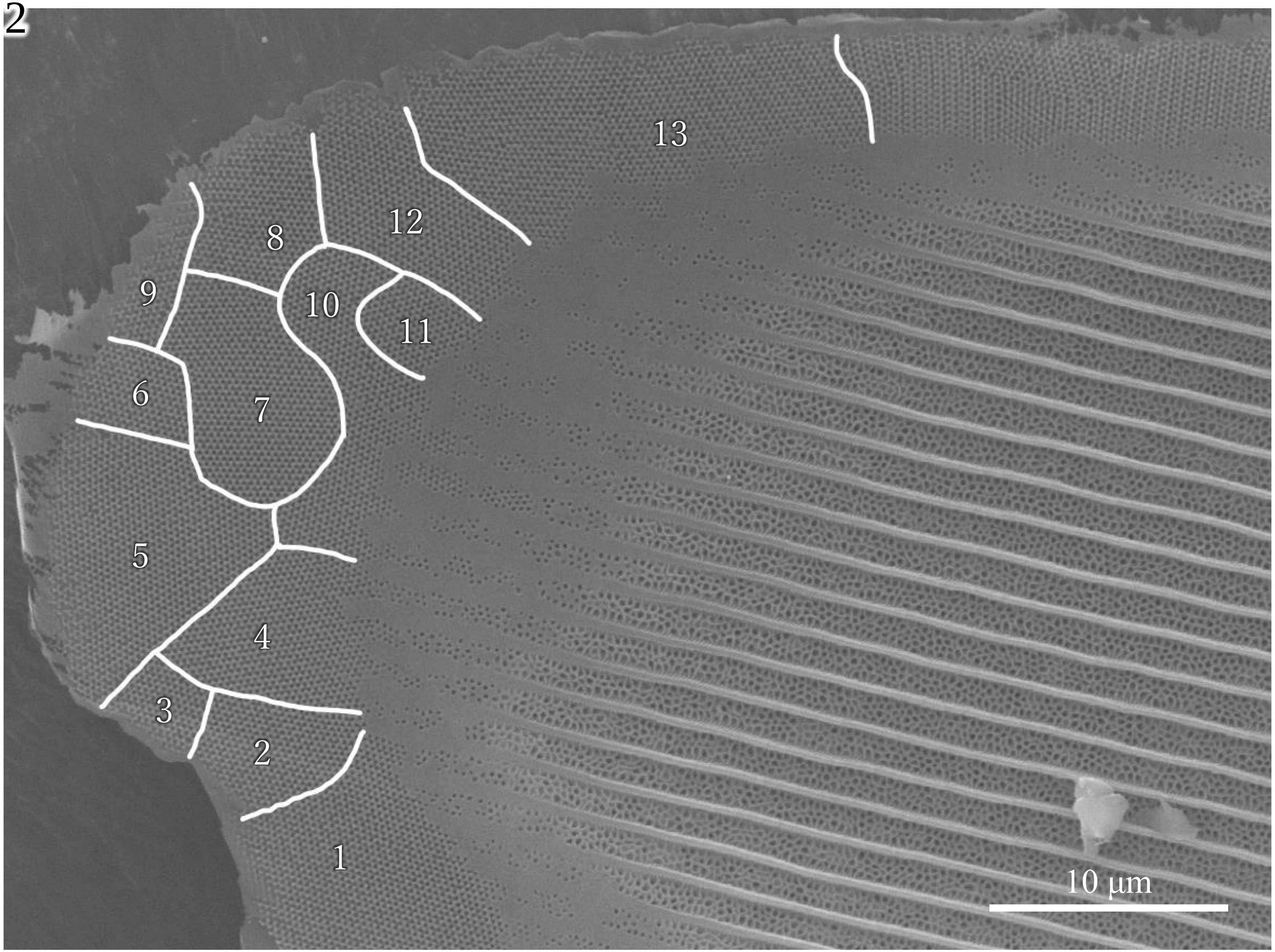

specimen No. 2
scale No. 6
domain No. 1
LH

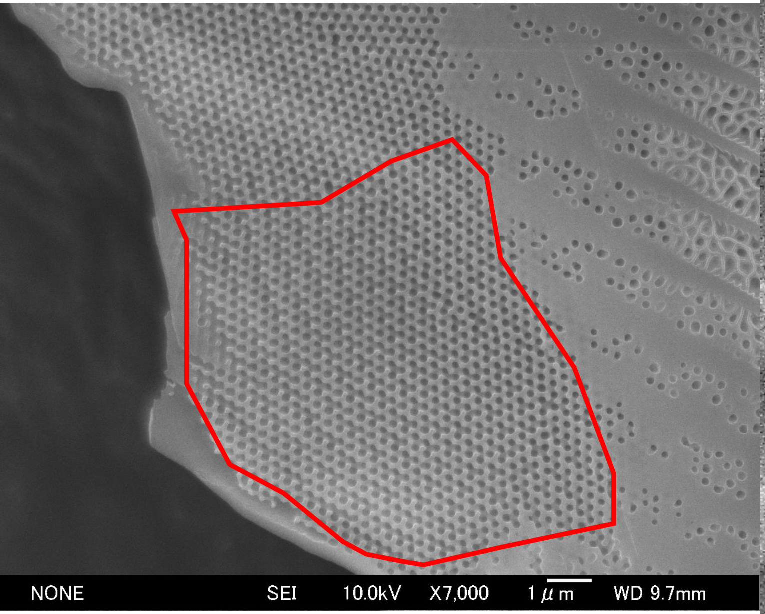

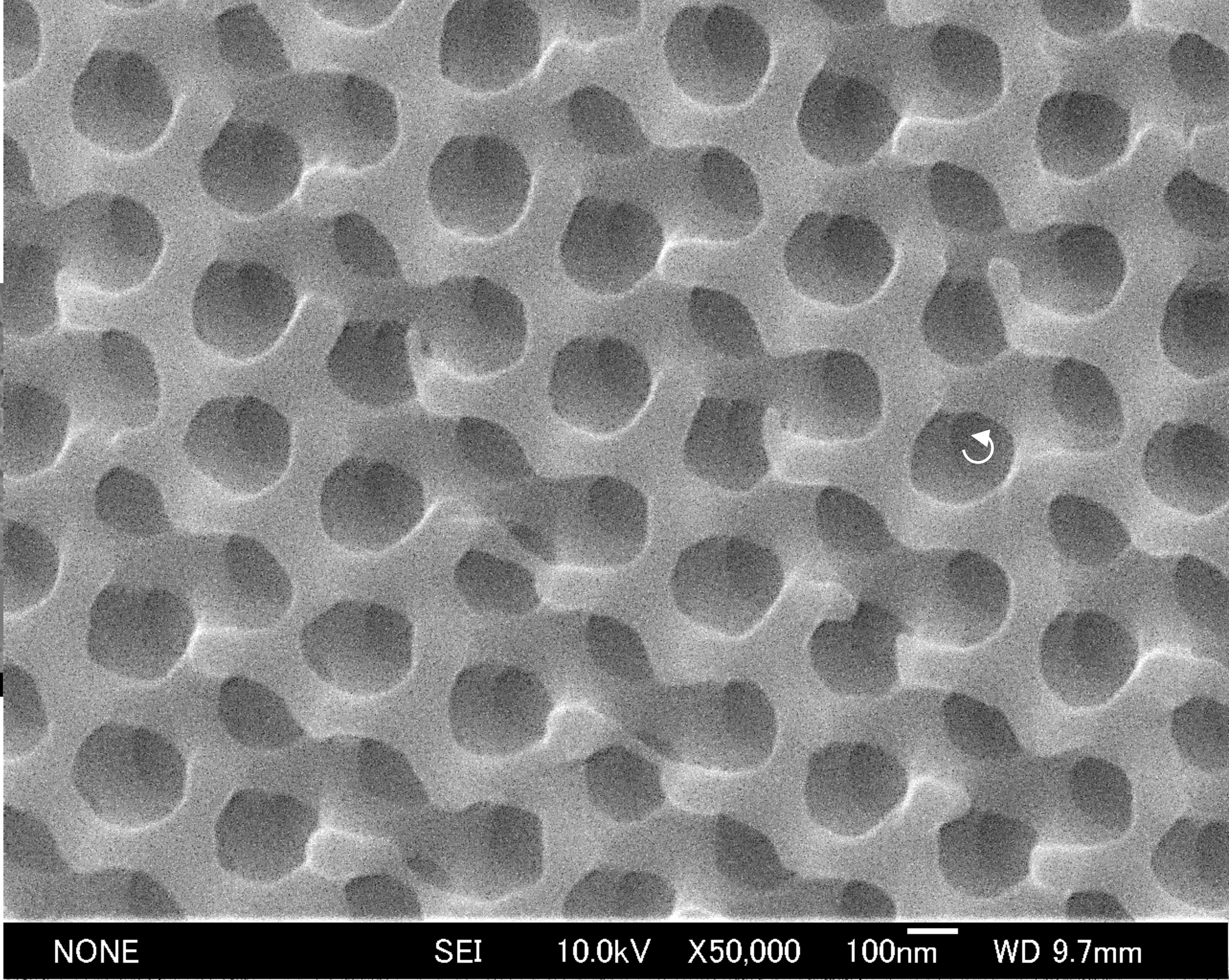

specimen No. 2
scale No. 6
domain No. 2
LH

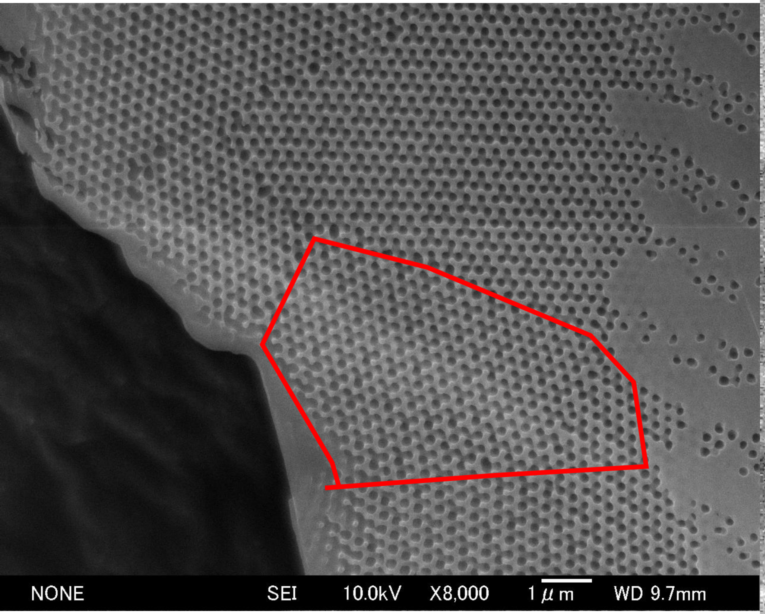

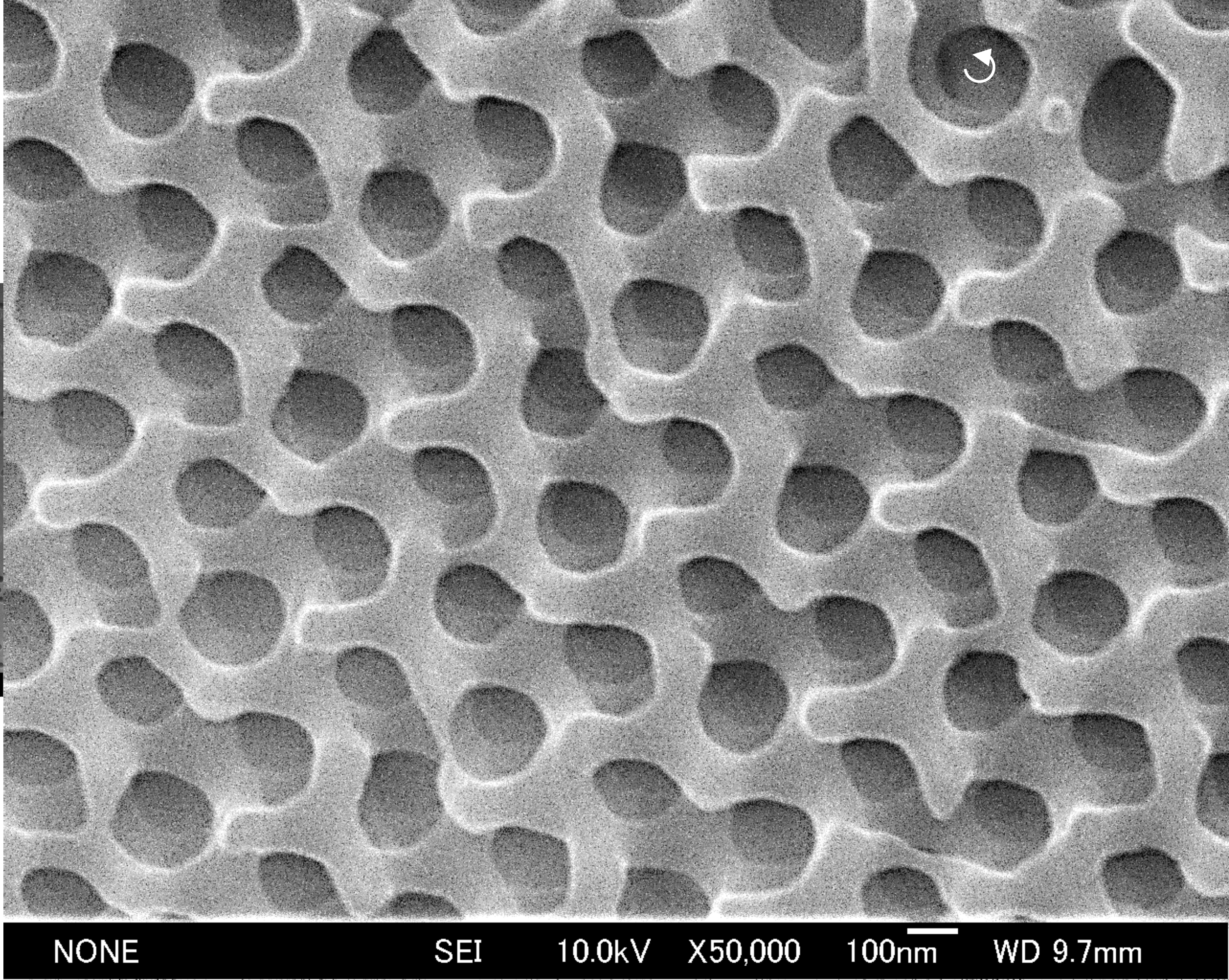

specimen No. 2
scale No. 6
domain No. 3
LH

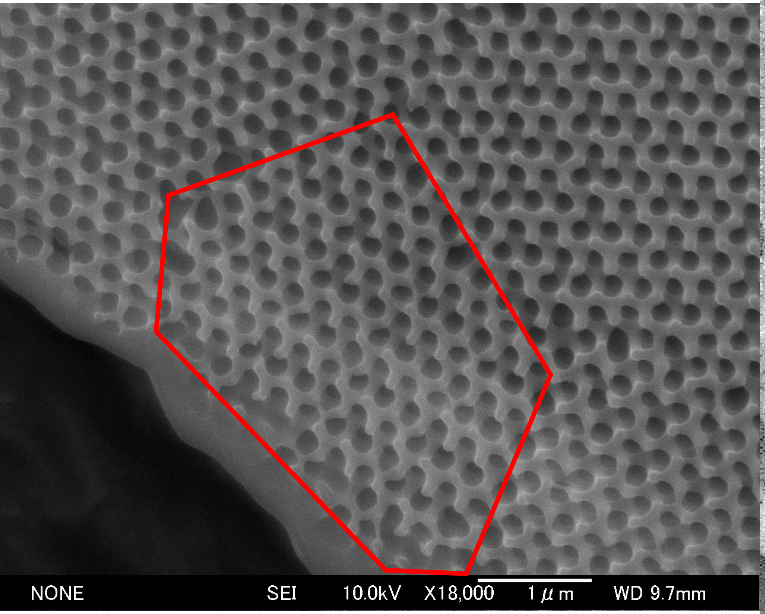

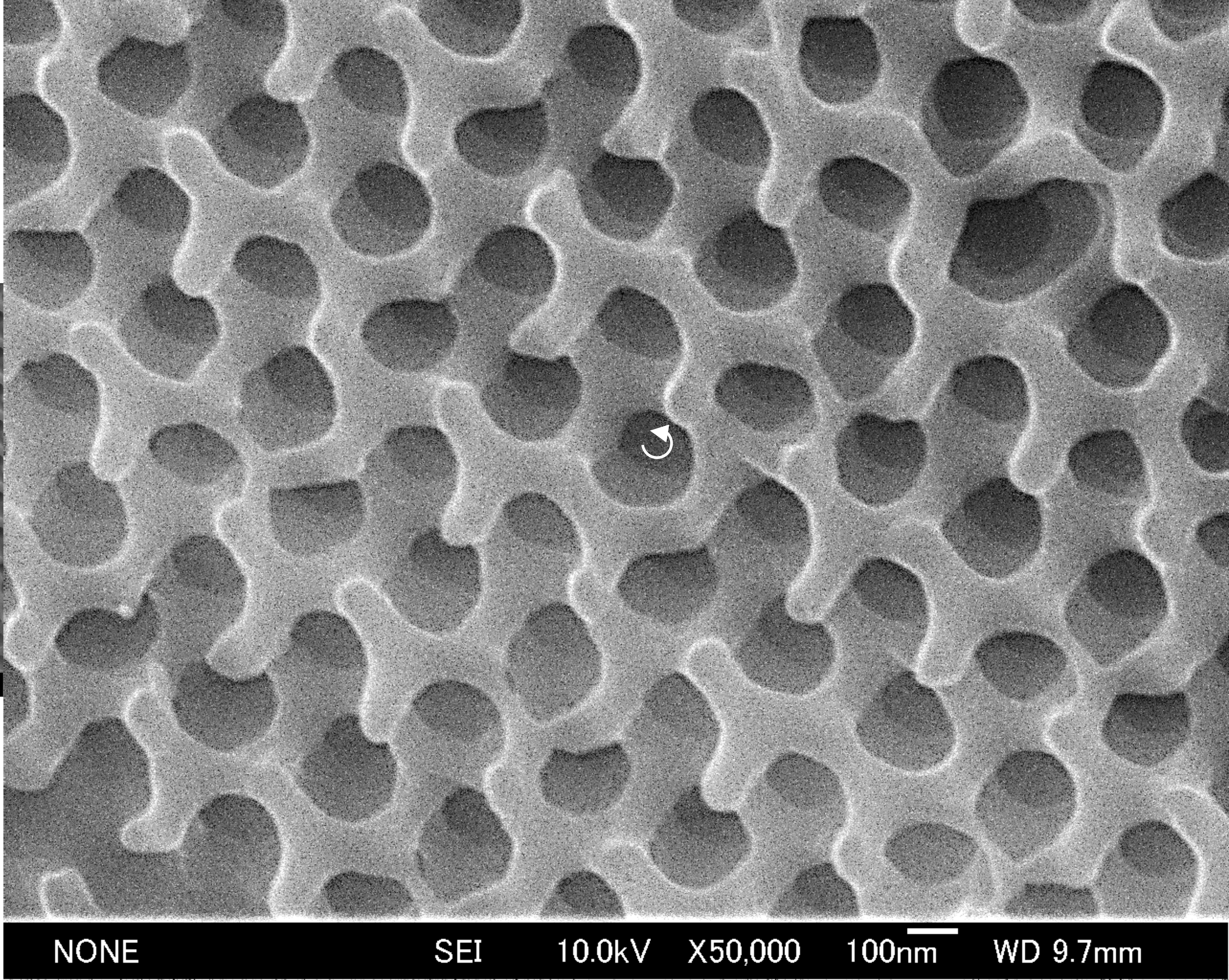

specimen No. 2
scale No. 6
domain No. 4
LH

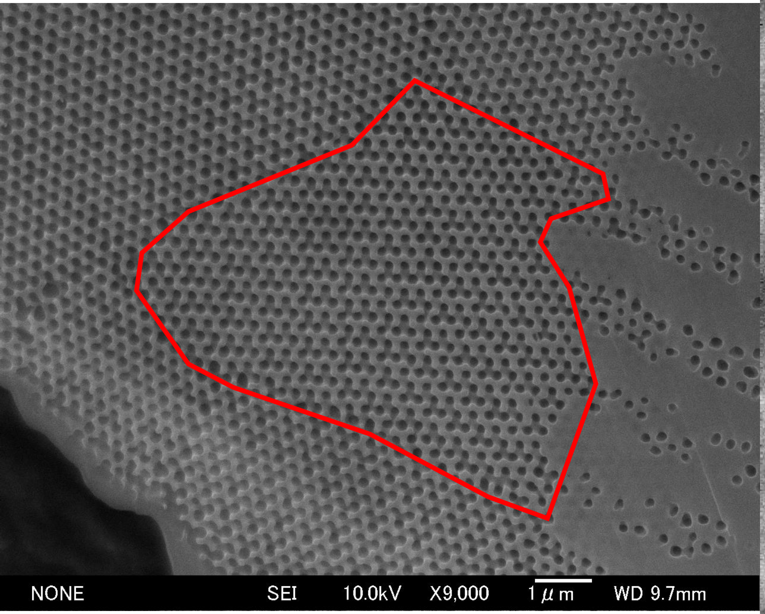

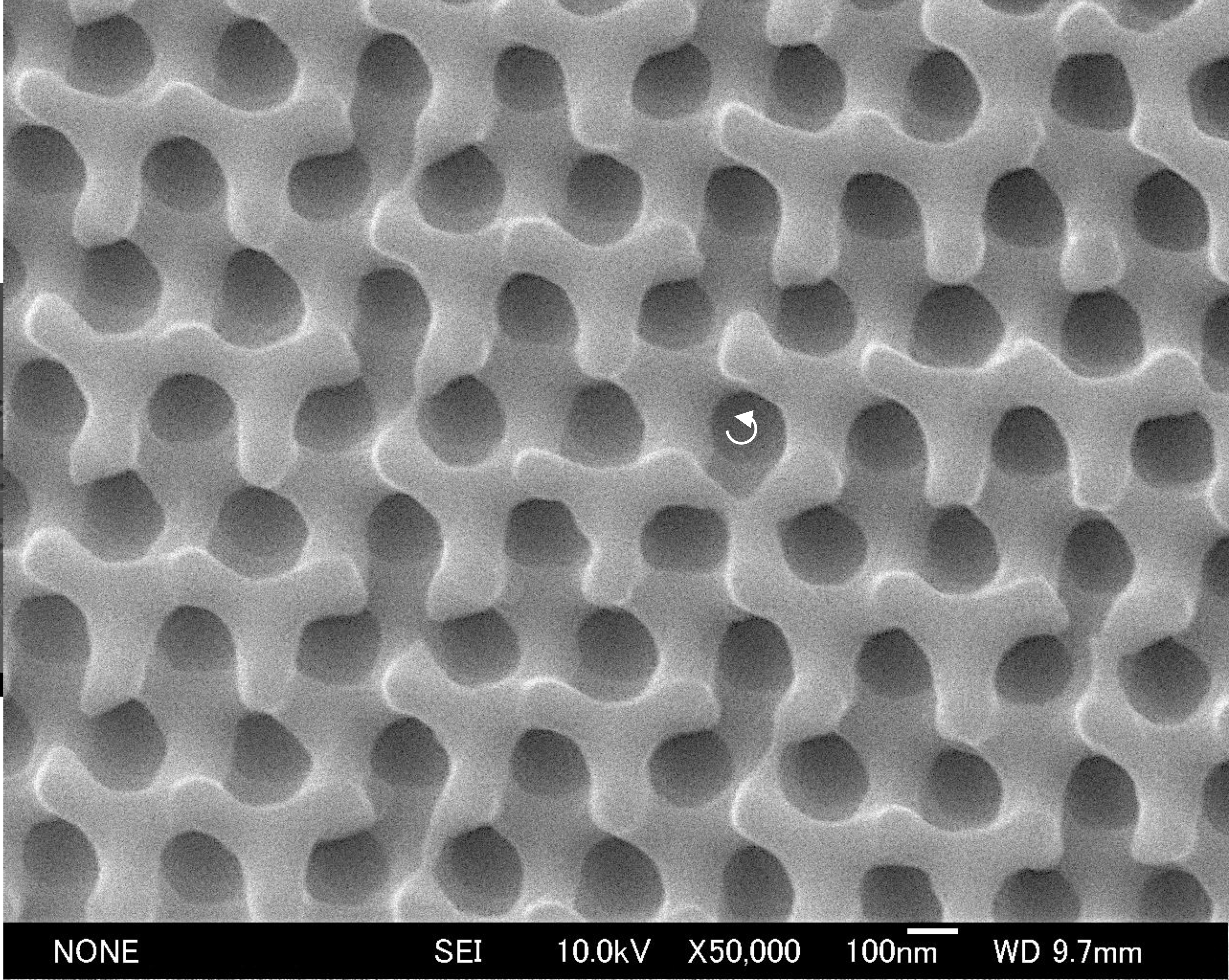

specimen No. 2
scale No. 6
domain No. 5
LH

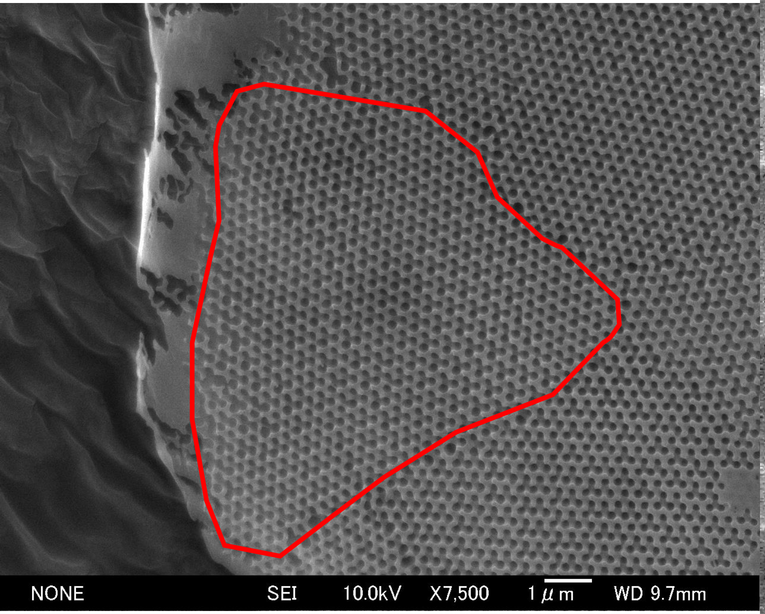

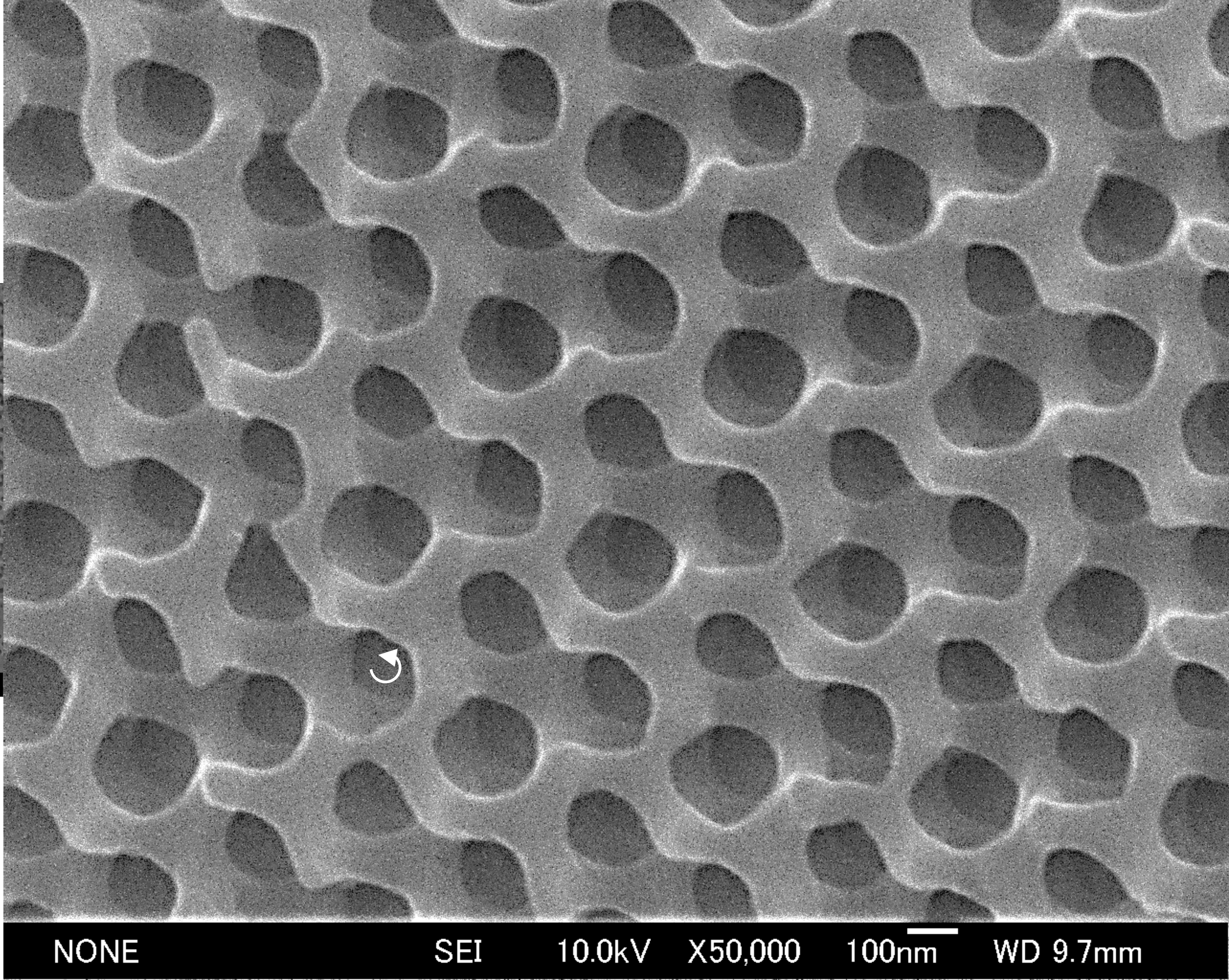

specimen No. 2
scale No. 6
domain No. 6
LH

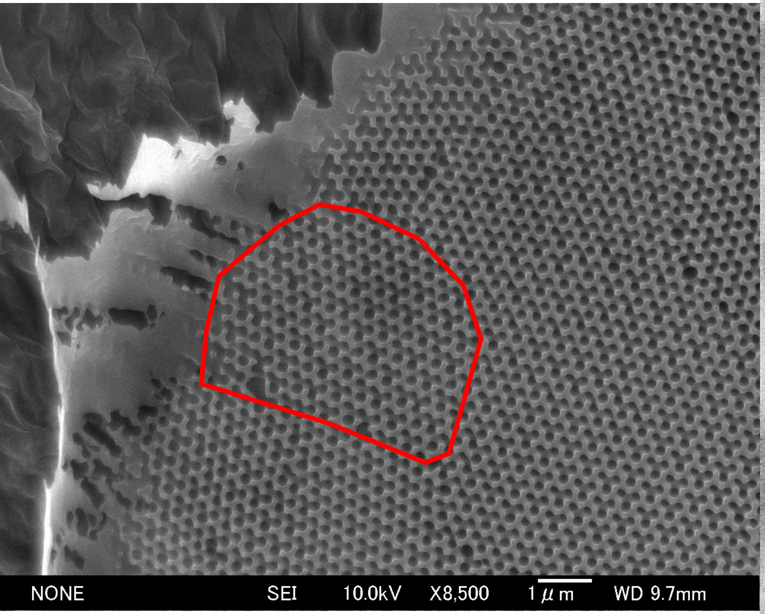

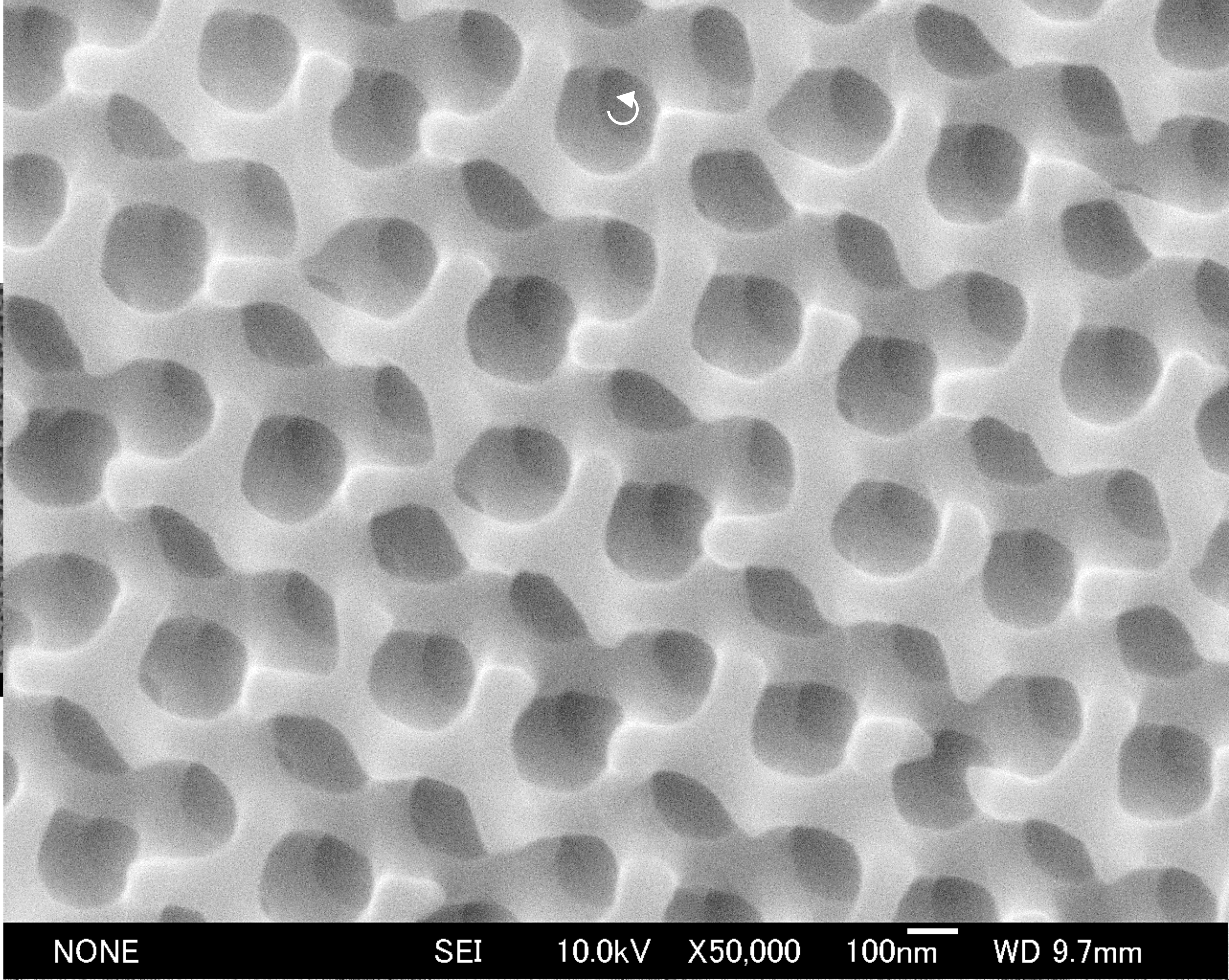

specimen No. 2
scale No. 6
domain No. 7
LH

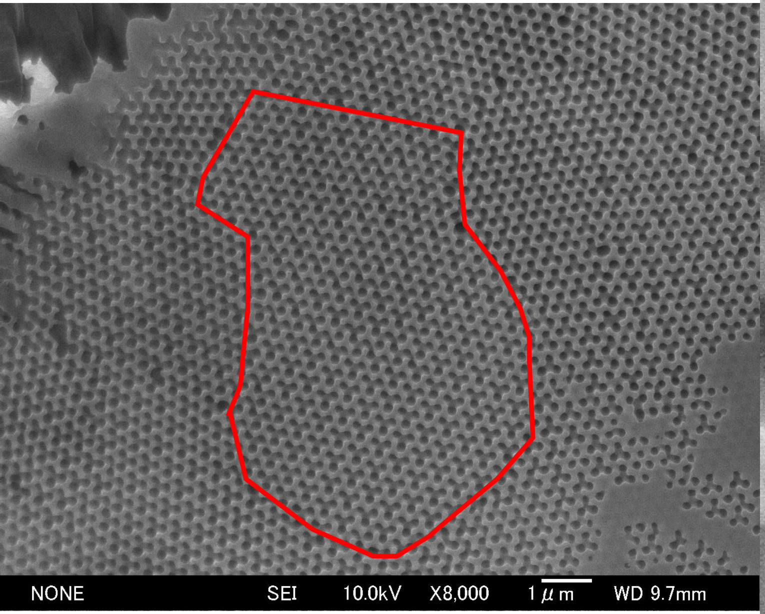

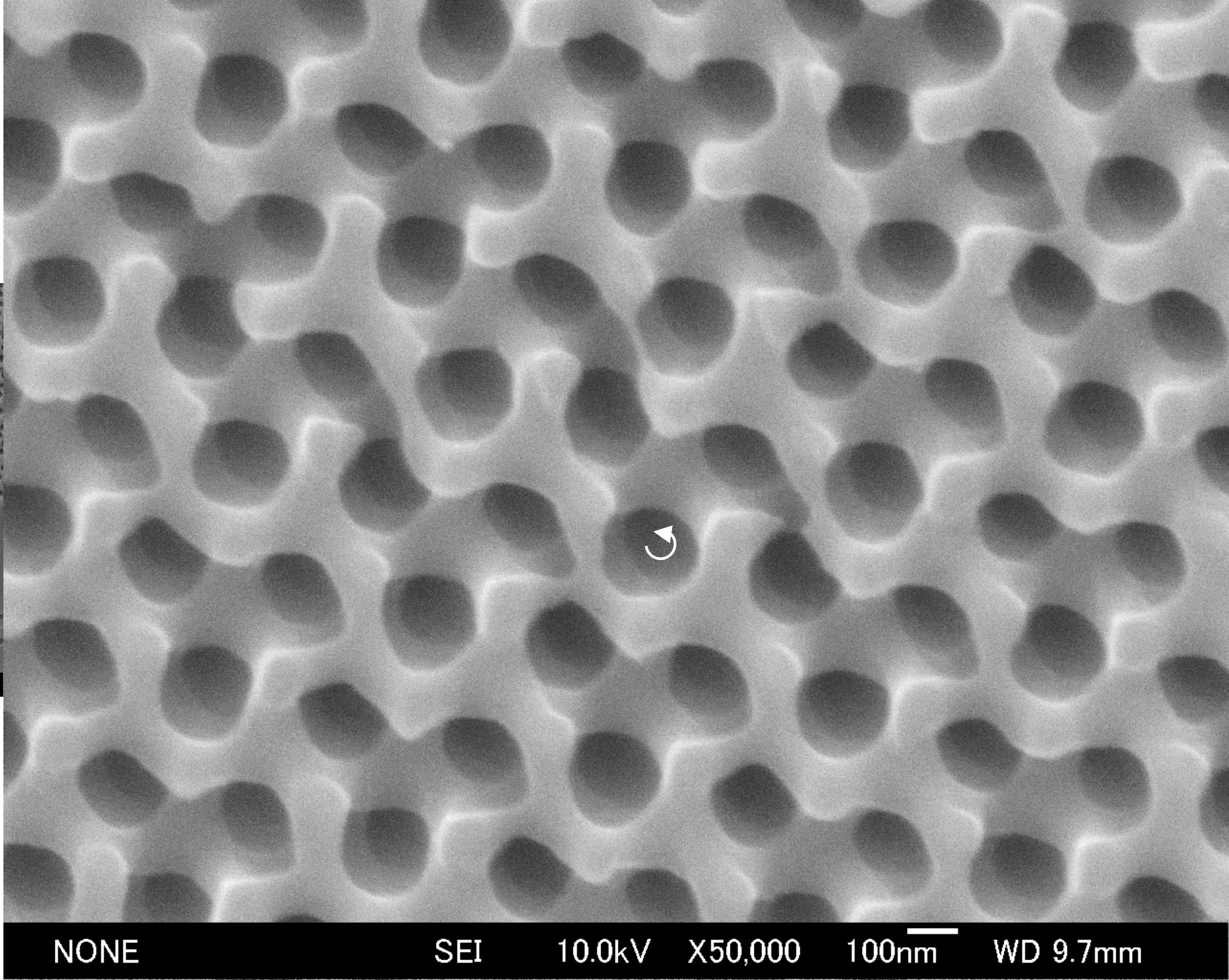

specimen No. 2
scale No. 6
domain No. 8
LH

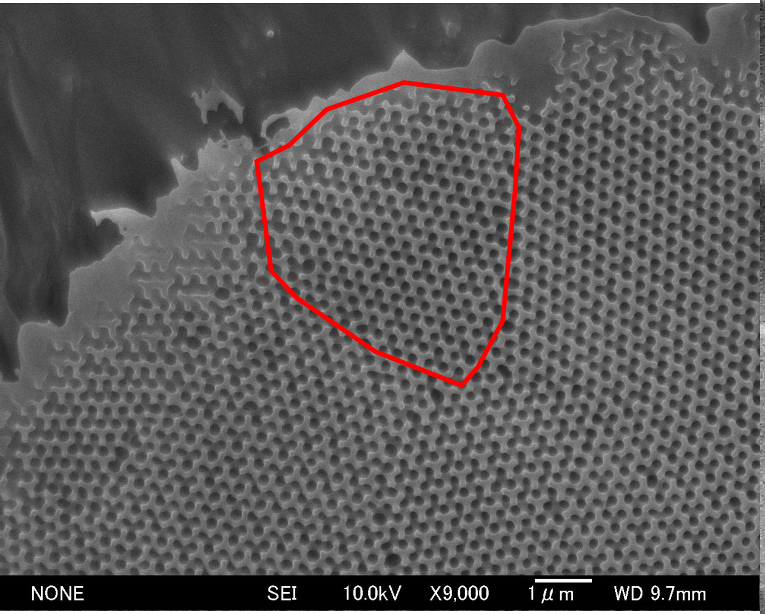

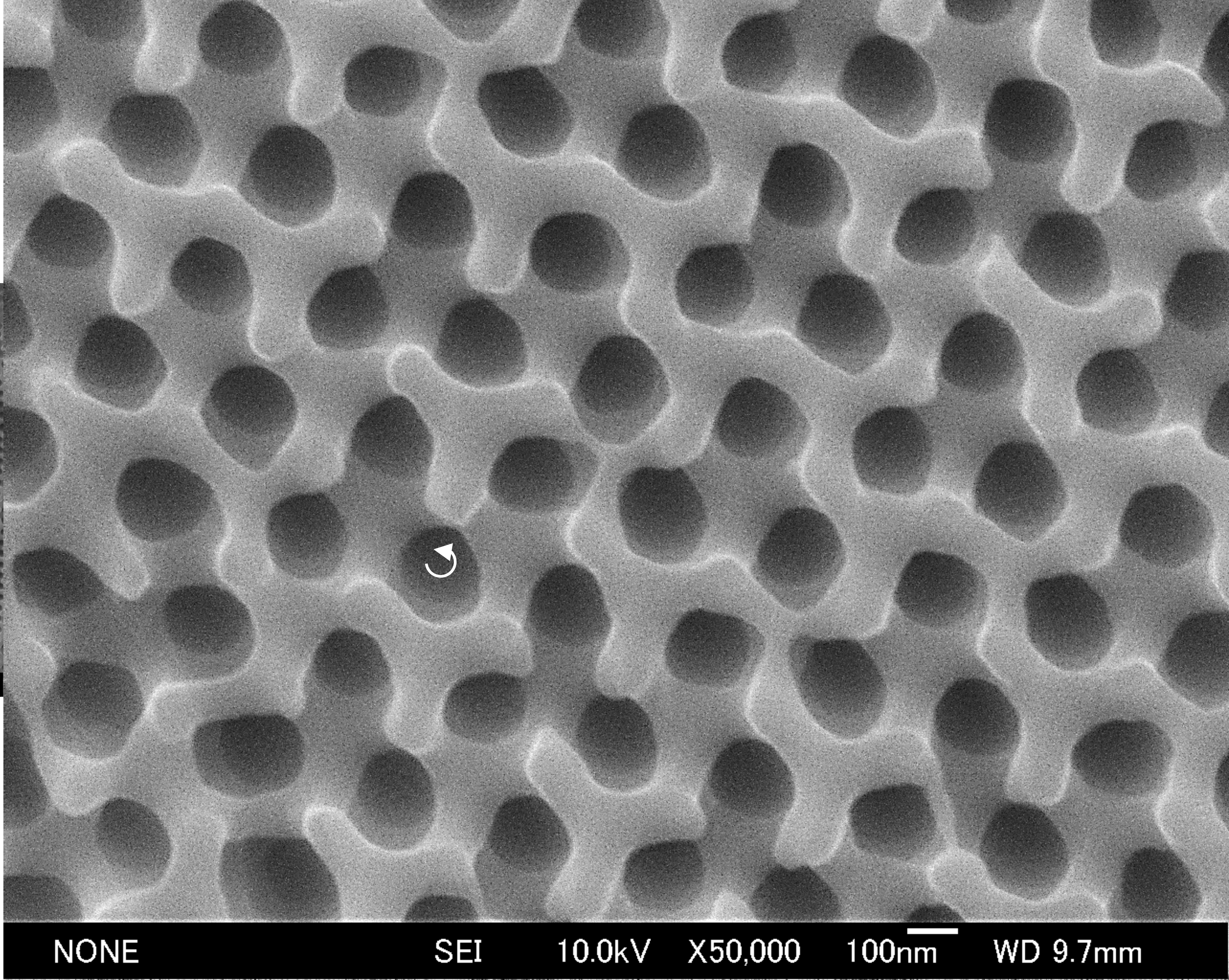

specimen No. 2
scale No. 6
domain No. 9
LH

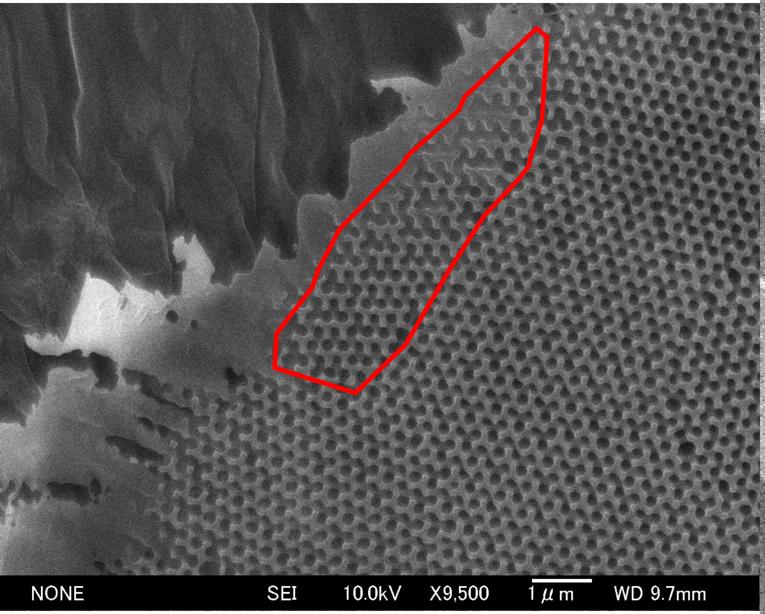

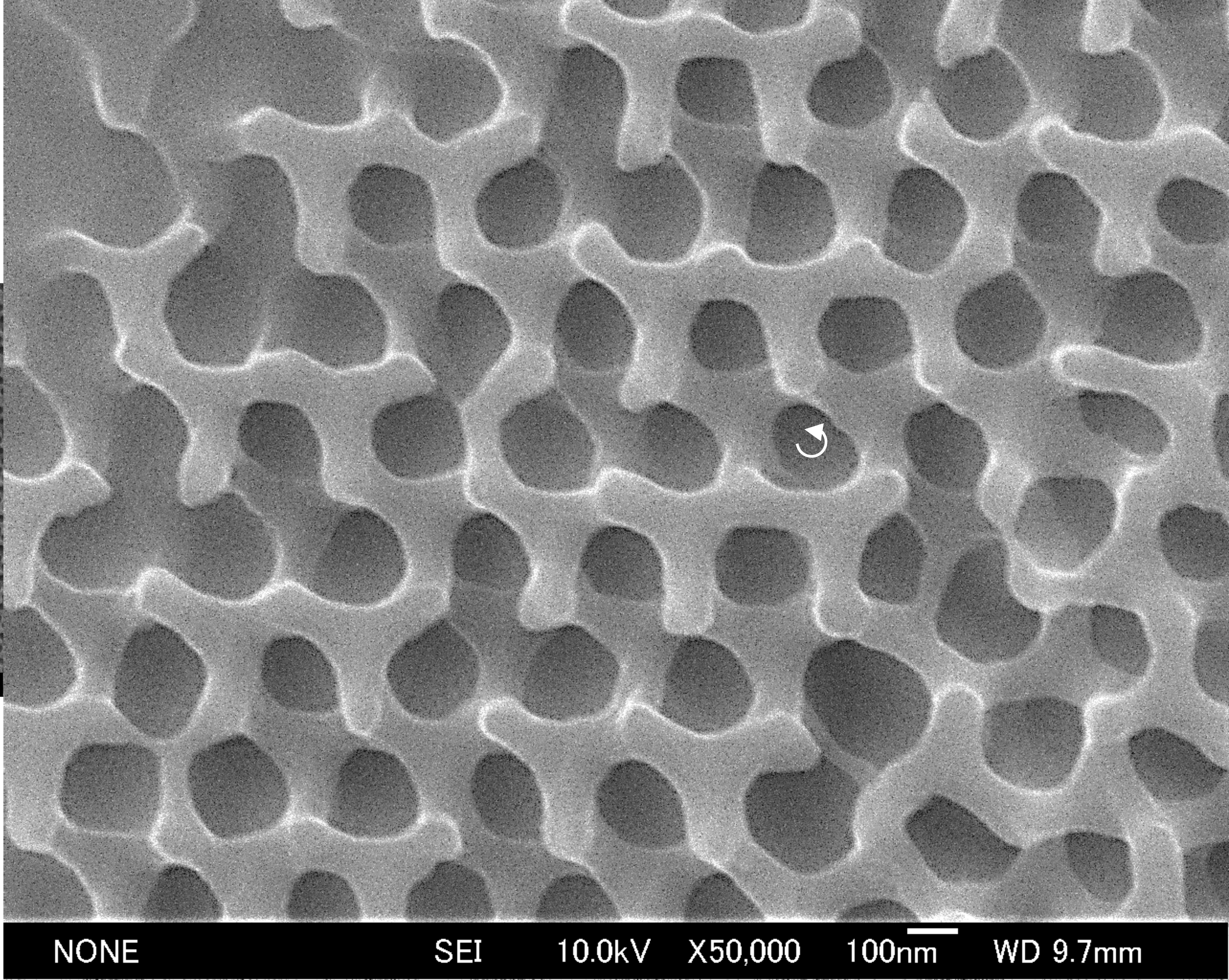

specimen No. 2
scale No. 6
domain No. 10
LH

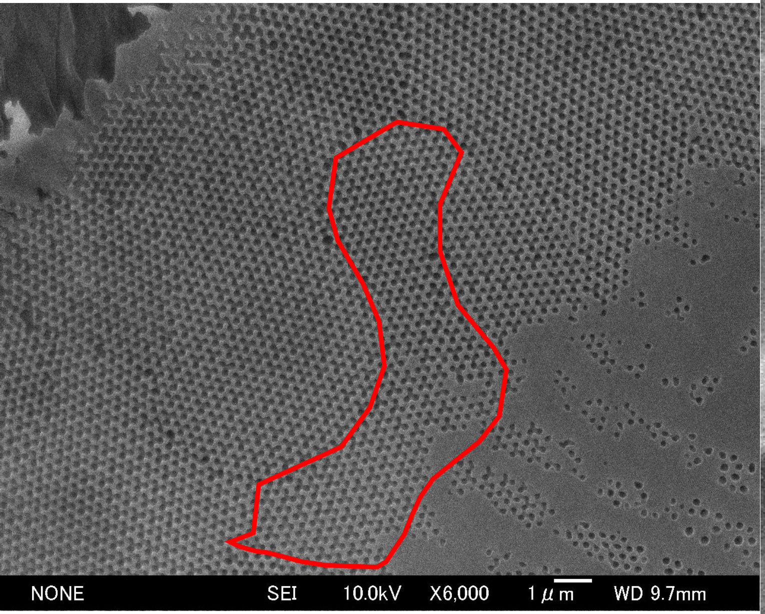

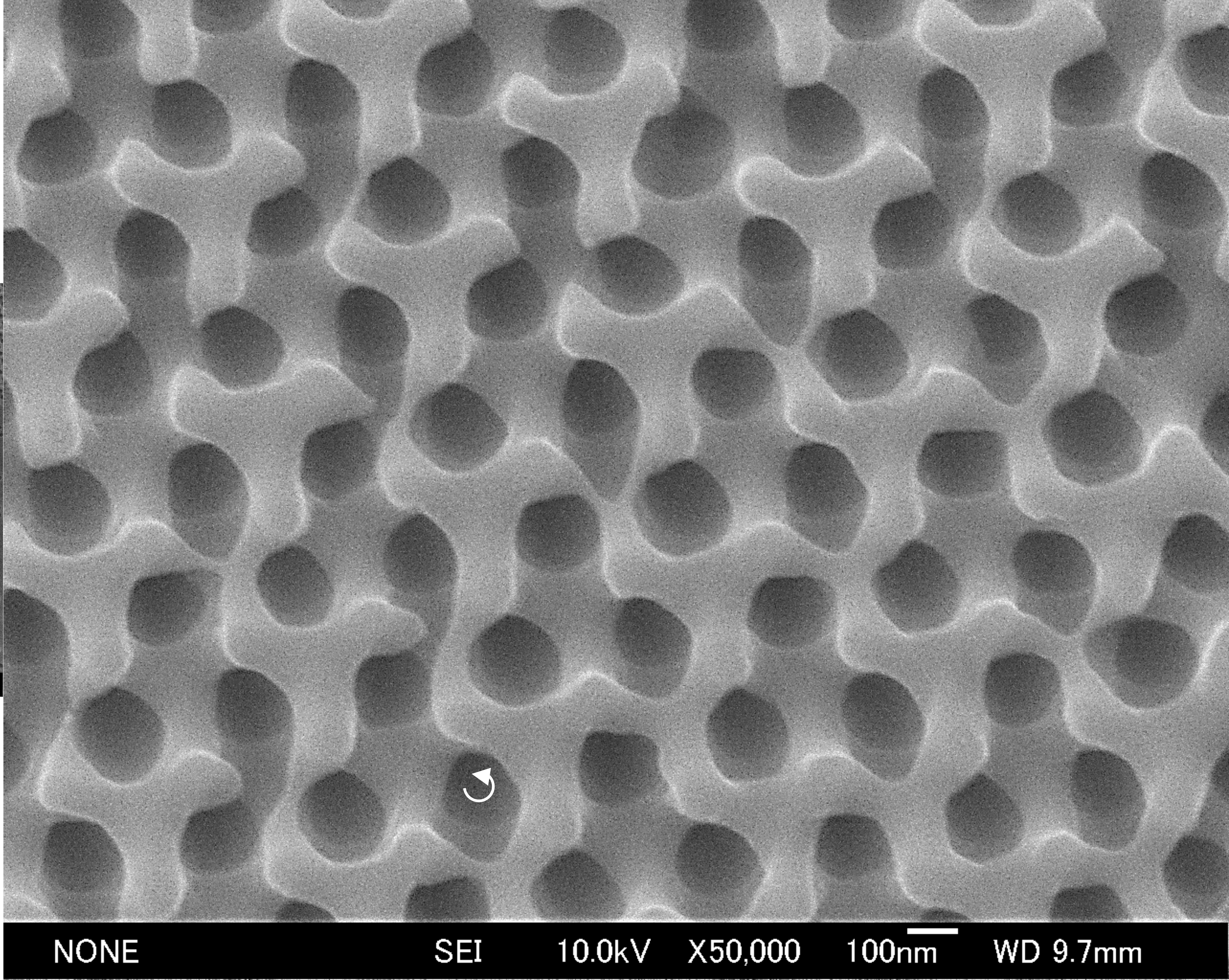

specimen No. 2
scale No. 6
domain No. 11
LH

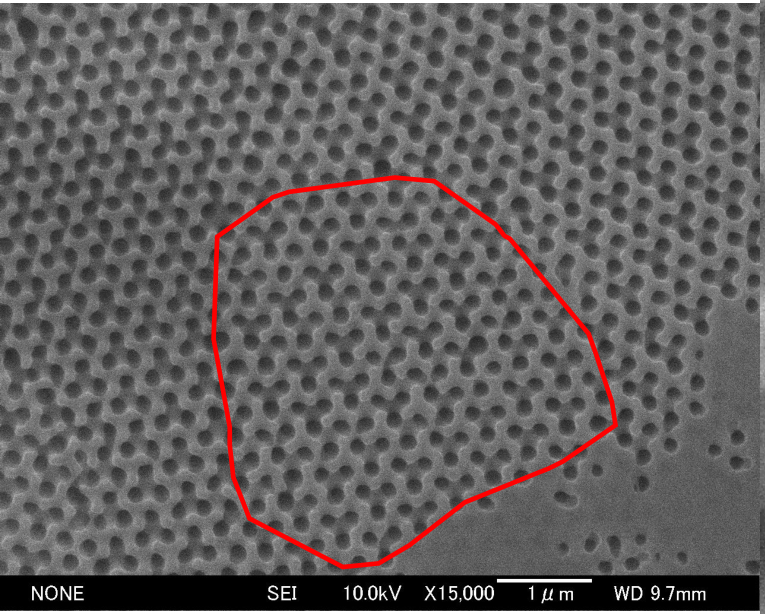

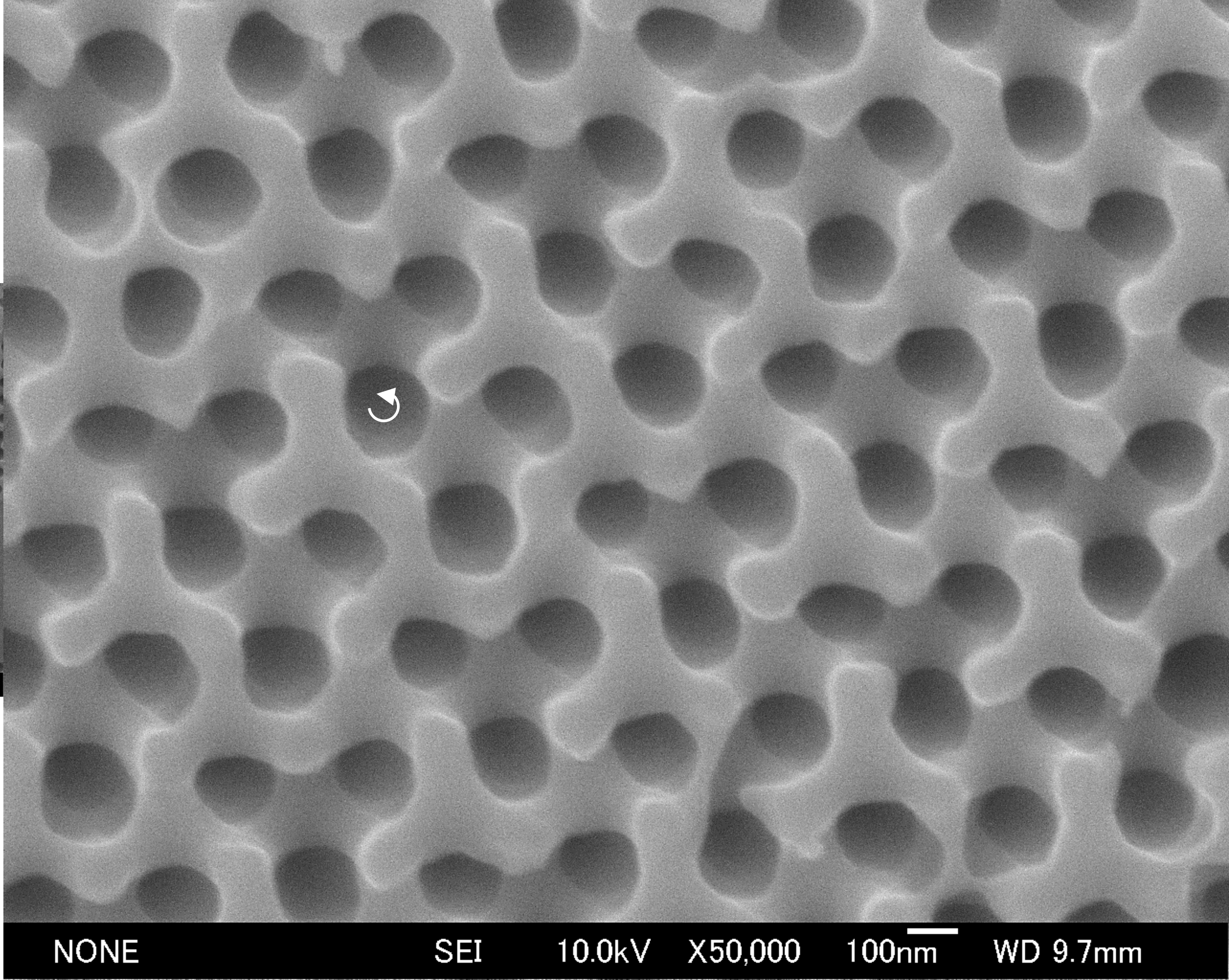

specimen No. 2
scale No. 6
domain No. 12
LH

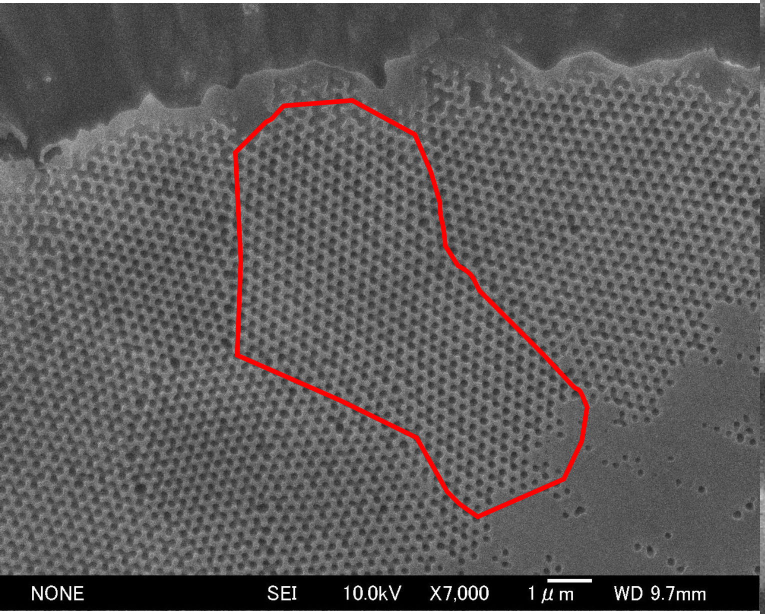

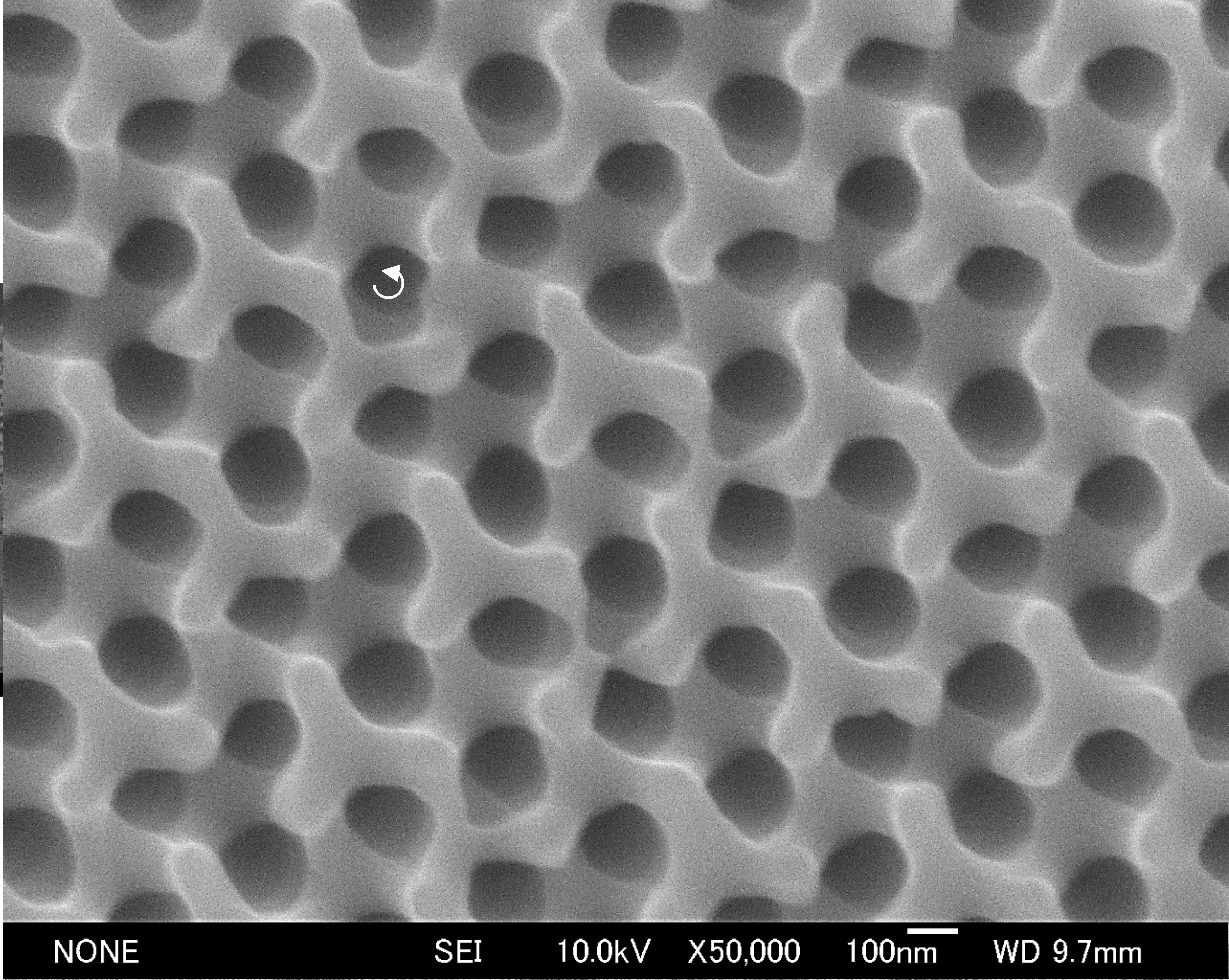

specimen No. 2
scale No. 6
domain No. 13
LH

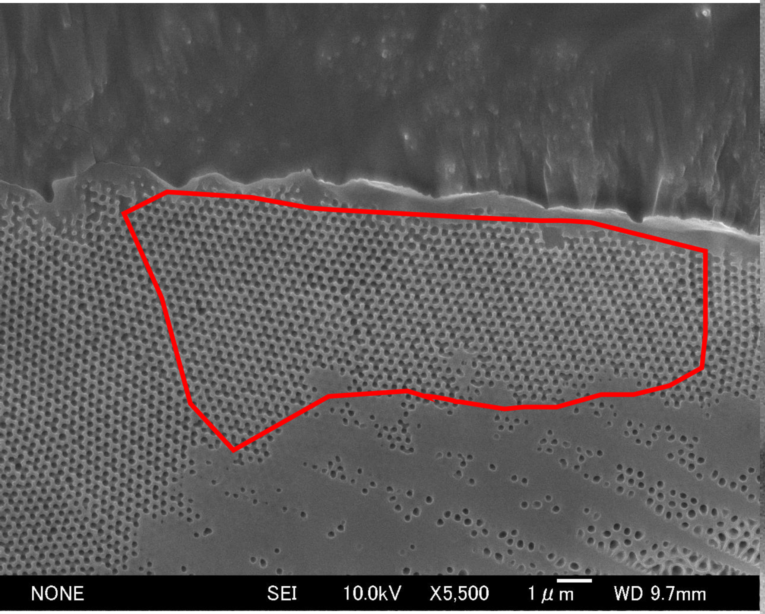

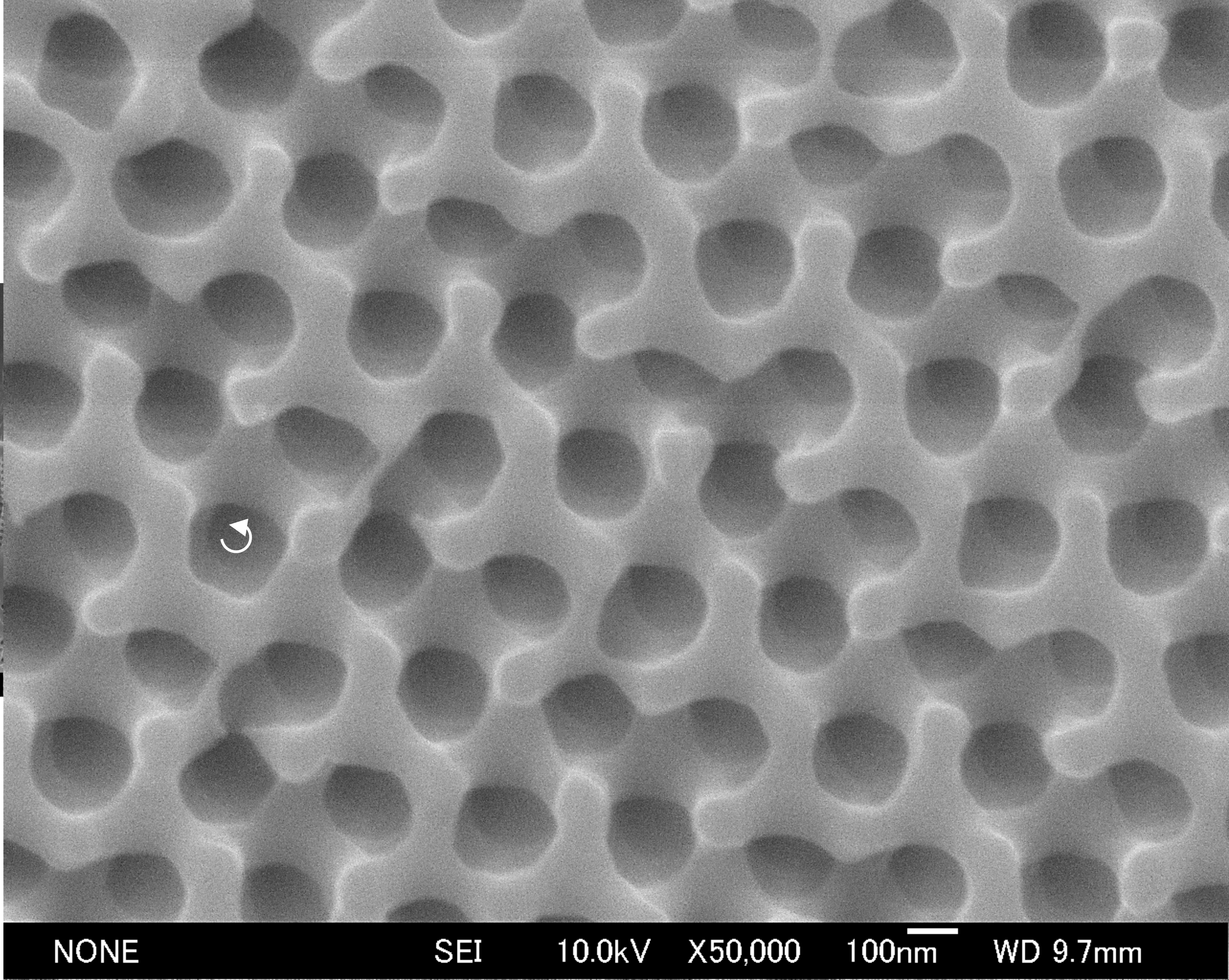

specimen No. 2
scale No. 7

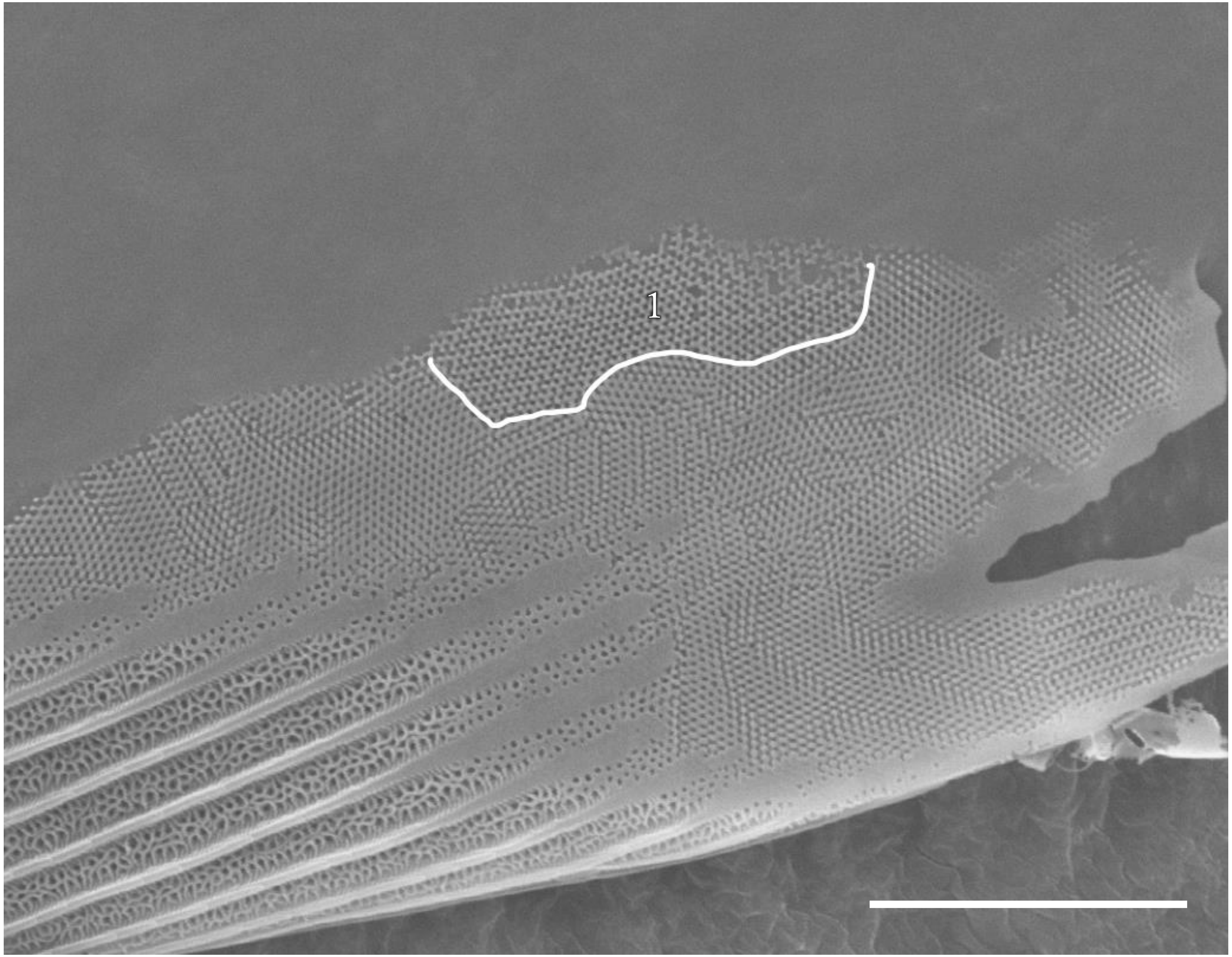

10 µm

specimen No. 2
scale No. 7
domain No. 1
LH

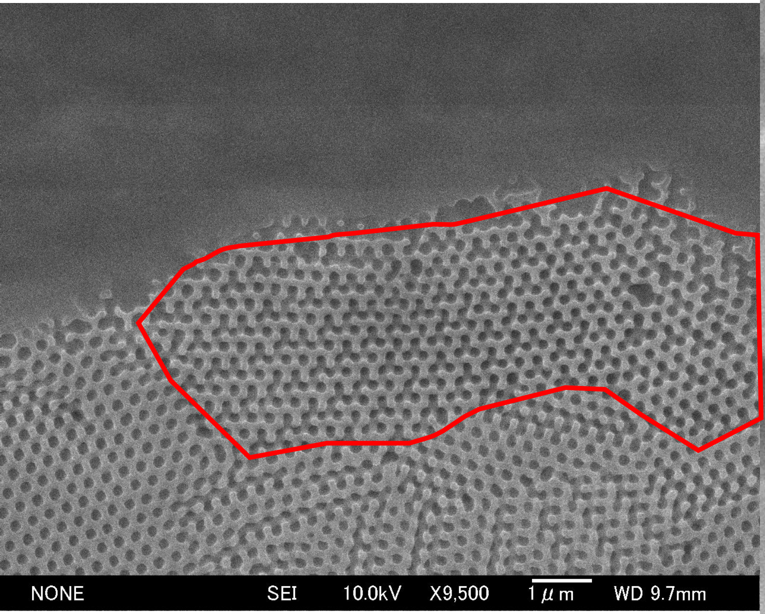

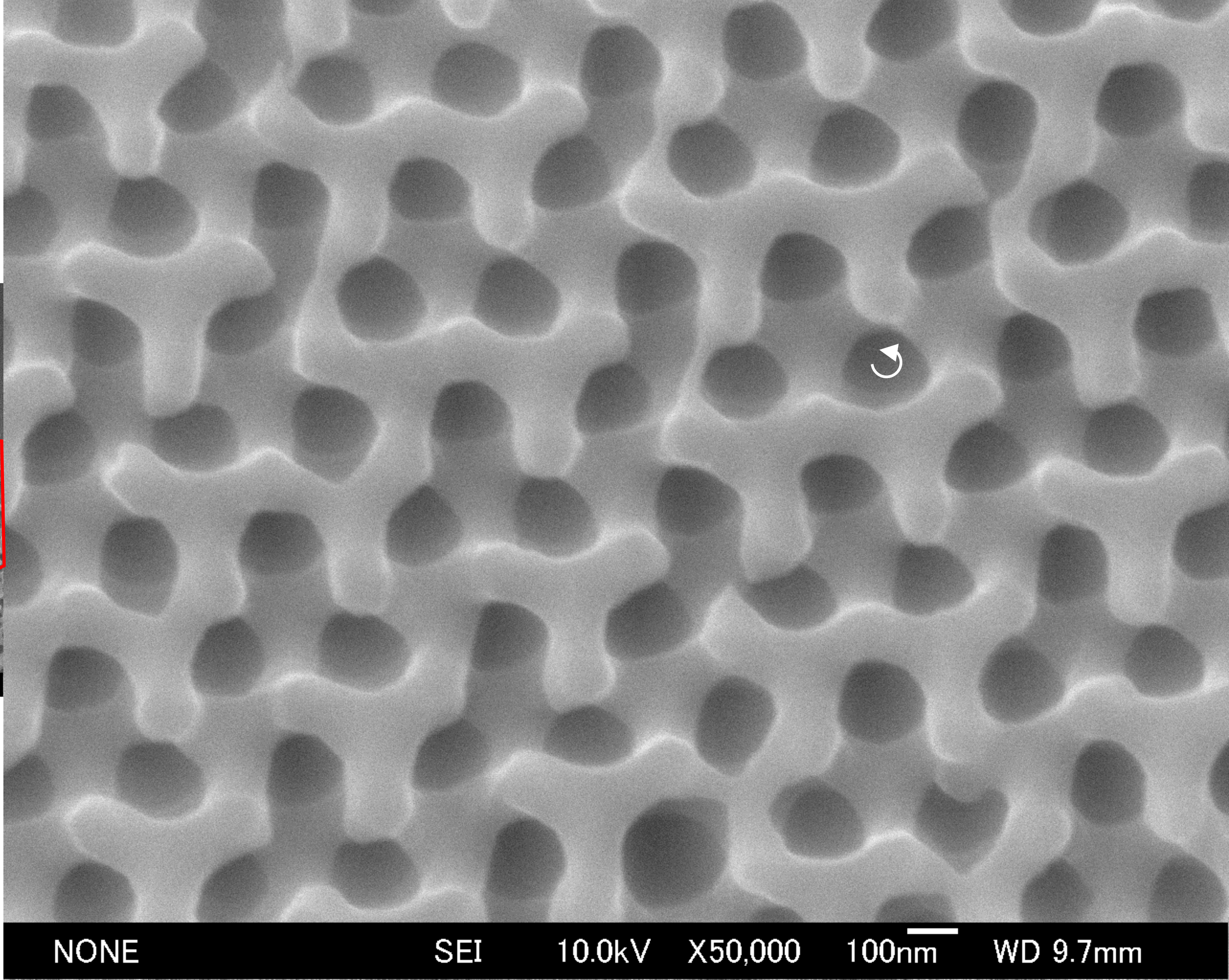

Supplement: Supplementary file 2 — Supplementary Information 2. [file 41598_2025_5750_MOESM2_ESM.pdf]
